# Supplementary material for: Age and sex effects on advanced white matter microstructure measures in 15,628 older adults: A UK biobank study
Source: Brain Imaging Behav. 2021 Sep 18;15(6):2813–23. doi: 10.1007/s11682-021-00548-y (PMC8761720; doi:10.1007/s11682-021-00548-y)
Supplement: Supplementary file 1 — Supplementary file1 (PDF 31.4 MB) [file 11682_2021_548_MOESM1_ESM.pdf]

Age and Sex Effects on Advanced White Matter Microstructure Measures in 15,628 Older Adults: A UK Biobank Study

Supplementary Methods

Participants

The UK Biobank is a publicly available dataset of community-based middle-aged and older adults residing in the United Kingdom, described in greater detail elsewhere (Miller et al., 2016). In the current study, we analyzed available cross-sectional data from UK Biobank participants who had usable dMRI scans collected on a single scanner. Neuroimaging data collection for the UK Biobank is on-going, and all data included here was collected in Cheadle, Manchester prior to the COVID-19 pandemic. Subjects were only included in the current sample if their chromosomal sex was identical to their sex as recorded elsewhere in the UK Biobank (i.e., participant sex as reported by the subject or as recorded in the subject's medical records). Additionally, participants were required to have complete nuisance covariate data (see *Statistical Analyses*), and their dMRI data was required to pass quality control procedures (see *MRI Acquisition and Processing*). Our final sample included data from a total of 15,628 UK Biobank subjects aged 45-80 years, with an approximately equal number of males and females (47.6% male, 52.4% female). Additional demographic information is presented in Table S1.

MRI Acquisition and Processing

Diffusion-weighted MRI scans were acquired and preprocessed as described previously (Alfaro-Almagro et al., 2018; Miller et al., 2016). Briefly, all dMRI images included in the current study were collected on a single Siemens Skyra 3T scanner with a 32-channel headcoil using a standard ("monopolar") Stejskal-Tanner pulse sequence and consisted of two diffusion-weighted shells (b=1000 and 2000 s/mm^2) with 50 distinct diffusion-encoding directions each, for a total of 100 directions (voxel dimensions = 2mm^3 isotropic, field of view = 104x104x72, acquisition time = 7 minutes). Preprocessing consisted of correction for eddy currents, head motion, and outlier slices using FSL's Eddy tool, followed by gradient distortion correction (Alfaro-Almagro et al., 2018;

Andersson & Sotiropoulos, 2015; Andersson & Sotiropoulos, 2016). In the current study, white matter metrics were derived using four dMRI reconstruction models: DTI, TDF, NODDI, and MAPMRI. Each reconstruction method is further described below (see *Reconstruction Models*). For single-shell dMRI data (b=1000 s/mm^2, 50 diffusion-encoding directions), the conventional model, DTI, was fit by the UK Biobank using FSL's DTIFIT (Alfaro-Almagro et al., 2018). The advanced model, TDF, was fit in-house using custom scripts (https://resource.loni.usc.edu/resources/downloads/tensor-distribution-function-code/; Nir et al., 2017). Metrics derived from DTI included fractional anisotropy (FA^{DTI}), mean diffusivity (MD), axial diffusivity (AD), and radial diffusivity (RD). TDF was used to derive an advanced measure of fractional anisotropy (FA^{TDF}). For multi-shell dMRI data (b=1000 and 2000 s/mm^2, 100 diffusion-encoding directions total), the advanced models NODDI and Laplacian-regularized MAPMRI were fit by the UK Biobank and in-house using the AMICO tool and DIPY, respectively (Daducci et al., 2015; Fick, Wassermann, Caruyer, & Deriche, 2016; Garyfallidis et al., 2014). The following white matter indices were calculated using NODDI: orientation dispersion (OD), intra-cellular volume fraction (ICVF), and isotropic volume fraction (ISOVF). Measures derived from MAPMRI included return-to-origin probability (RTOP), return-to-axis probability (RTAP), and return-to-plane probability (RTPP).

Diffusion-weighted MRI metrics were projected in-house to a standard white matter skeleton using publicly available ENIGMA protocols (http://enigma.ini.usc.edu/protocols/dti-protocols; Jahanshad et al., 2013). Briefly, each subject's whole-brain FA^{DTI} map was nonlinearly registered to the ENIGMA FA^{DTI} template using ANTs's symmetric image normalization (SyN) method (Avants, Epstein, Grossman, & Gee, 2008), and the resulting transformation was applied to the maps for each metric. White matter metrics were projected onto the ENIGMA template skeleton based on the FA^{DTI} metric using FSL's tract-based spatial statistics (TBSS; Smith et al., 2006). Major white matter regions of interest (ROIs) were labeled in-house using the Johns Hopkins University white matter atlas in ICBM space (ICBM-DTI-81 white matter labels atlas; Mori et al., 2008). After each processing step in the dMRI pipeline, 2D images of the output were visually inspected as part of quality assurance procedures. Subjects whose data warranted further attention based on this quality control, or by virtue of being outliers, underwent thorough visual inspection of their complete 4D dMRI scan. Participants with poor quality data (e.g., gross visual artifacts) were subsequently excluded prior to all group-level analyses.

For our planned *a priori* analyses examining age and sex effects on white matter microstructure, mean values for each dMRI metric were extracted from the whole white matter skeleton (full WM) and from the largest white matter structure in the brain, the corpus callosum (CC), consistent with prior literature (e.g., Beck et al., 2020; Jahanshad & Thompson, 2017; Pines et al., 2020). For supplementary analyses, mean values for each metric were extracted from an additional 14 white matter ROIs (Table S2).

Statistical Analyses

Primary analyses investigated the effects of age, sex, and their interaction on the full WM and CC. We used fractional polynomials (*mfp* in R; R Core Team, 2016) to flexibly model age in a non-linear manner without specifying *a priori* the relationship between age and the dependent variable (Dima et al., 2020; Frangou et al., 2020; Royston & Altman, 1994). Briefly, the best-fitting age model was found for the full WM and CC for each dMRI metric separately by assessing one- and two-term curvilinear age models with the following possible powers (Dima et al., 2020; Frangou et al., 2020; Royston & Altman, 1994): -2, -1, -0.5, 0, 0.5, 1, 2 and 3, where x^0 corresponds to $ln(x)$. In line with prior work (Salminen et al., 2019), all analyses included the following nuisance covariates: educational attainment (operationalized as "college" or "no college"), socioeconomic status (quantified using the Townsend Deprivation Index; Townsend, Phillimore, & Beattie, 1988), waist-hip ratio, and population structure (measured using the first 4 principal components obtained from the UK Biobank's genetic ancestry analysis; Bycroft et al., 2017). Effect sizes were calculated as the variance explained separately by age, sex, and their interaction. For instance, the effect size for age was computed as the difference in variance (change in R^2) between two models: one which included age in addition to sex and nuisance covariates, and one which only included sex and nuisance covariates. The overall R^2 for each full model is reported in the Supplementary Material.

For completeness, supplemental analyses used stricter statistical thresholds ($p < 0.01$ and $p < 0.001$) and characterized age and sex effects on an additional 14 white matter ROIs across the brain (Table S2) by using the statistical approach described above; a false discovery rate (FDR) of 5% was applied to correct for multiple comparisons across the 14 supplemental ROIs assessed (Benjamini & Hochberg, 1995). To confirm all results were robust to statistical approach, we followed previously published fractional polynomial

recommendations to repeat analyses after discretizing the continuous variable of interest (Royston & Sauerbrei, 2004). For these supplemental analyses, we binarized age by splitting participants into two groups: $\geq$ 60 years old and < 60 years old; this binarization threshold was selected based on previous large-scale neuroimaging studies examining aging (Dima et al., 2020; Frangou et al., 2020).

Sex-stratified centile reference curves were created for each white matter region and dMRI metric. Specifically, these normative charts modeled the fractional polynomial age terms determined above by using quantile regression (*quantreg* in R) to calculate the following percentiles: 5, 25, 50, 75, and 95.

Reconstruction Models

Diffusion Tensor Imaging (DTI). DTI models a single fiber orientation by fitting a single diffusion tensor, or ellipsoid, at each voxel of the brain in single-shell dMRI data (Basser, Mattiello, & Lebihan, 1994). DTI assumes a 3D Gaussian diffusion process, which is fit using six independent tensor parameters (3 eigenvalues and 3 eigenvectors) that together describe the shape and orientation of the tensor. Here we used DTI to create maps of tensor-derived fractional anisotropy (FA^{DTI}), which represents the degree of diffusion anisotropy within a fiber bundle (Jones, 2008).

$$FA = \sqrt{\frac{3}{2}} \frac{\sqrt{(\lambda_1 - <\lambda>)^2 + (\lambda_2 - <\lambda>)^2 + (\lambda_3 - <\lambda>)^2}}{\sqrt{\lambda_1^2 + \lambda_2^2 + \lambda_3^2}}$$

The eigenvalues are represented as λ_1, λ_2, λ_3, and the average diffusivity in all directions, or mean diffusivity (MD), is represented as $< \lambda >$. The diffusion parallel to the primary axis of diffusion, or axial diffusivity (AD), is defined as λ_1, the primary (largest) eigenvalue. Diffusion perpendicular to the primary diffusion axis, or radial diffusivity (RD), is calculated as the average of λ_2 and λ_3, the second and third eigenvalues. In the current study, we assessed the metrics FA^{DTI}, MD, AD, and RD.

Tensor Distribution Function (TDF). TDF models single-shell dMRI data by using a probabilistic mixture of tensors, which allows for the reconstruction of multiple underlying fiber populations, together with a distribution

of weights (Leow et al., 2009; Nir et al., 2017). For each voxel, the TDF is calculated as the probability distribution function *P*(D(θ, λ))* of all feasible Gaussian tensors *D(θ, λ).* The tensor orientation distribution function (TOD) is then computed as the marginal density function of the TDF.

$$TOD(\theta) = \int_{\lambda} P(D(\theta, \lambda))\, d\lambda$$

Eigenvalues are calculated for each spherical angle θ by computing the expected value of each eigenvalue λ along θ. From this, the advanced fractional anisotropy measure FA^{TDF} is calculated.

$$FA^{TDF} = \int TOD(\theta) * FA(\theta)\, d\theta$$

$$FA(\theta) = \frac{\sqrt{(\lambda'_1(\theta) - \lambda'_2(\theta))^2 + (\lambda'_1(\theta) - \lambda'_3(\theta))^2 + (\lambda'_2(\theta) - \lambda'_3(\theta))^2}}{2[\lambda'_1(\theta)^2 + \lambda'_2(\theta)^2 + \lambda'_3(\theta)^2]}$$

$$\lambda'_i(\theta) = \frac{\int P(D(\theta, \lambda))\lambda_i\, d\lambda}{\int P(D(\theta, \lambda))\, d\lambda}$$

In the current study, we included FA^{TDF} in the examined dMRI metrics.

Neurite Orientation Dispersion and Density Imaging (NODDI). NODDI is a multi-compartment model applied to multi-shell dMRI data that models the complete dMRI signal $E(\mathbf{q})$ as consisting of intra-cellular, extra-cellular, and isotropic water components; each of these components uniquely affects diffusion in the environment and results in a separate dMRI signal (Zhang, Schneider, Wheeler-Kingshott, & Alexander, 2012).

$$E(\mathbf{q}) = v_{iso}E_{iso}(\mathbf{q}) + (1 - v_{iso})[v_{ic}E_{ic}(\mathbf{q}) + v_{ec}E_{ec}(\mathbf{q})]$$

The dMRI signal of the intra-cellular, extra-cellular, and isotropic compartments are represented here as E_{ic}, E_{ec}, E_{iso}, respectively. The corresponding volume fractions are v_{ic}, v_{ec}, v_{iso}, where $v_{ec} = 1 - v_{ic}$. E_{ic} is modeled as a set of dispersed cylinders of zero radius, where the dispersion is defined by a Watson distribution. E_{ec} represents a dispersed mixture of Gaussian anisotropic diffusion, and E_{iso} represents isotropic Gaussian diffusion (Daducci et al., 2015; Zhang et al., 2012). In the current study, we investigated orientation dispersion (OD), intra-cellular volume fraction (ICVF), and isotropic volume fraction (ISOVF) (Daducci et al., 2015; Zhang et al., 2012).

Mean Apparent Propagator MRI (MAPMRI). MAPMRI is a diffusion propagator-based model applied to multi-shell dMRI data that estimates water diffusion without requiring *a priori* assumptions regarding the underlying tissue (Fick et al., 2016; Ozarslan et al., 2013). Specifically, MAPMRI models the acquired dMRI signal $E(\mathbf{q})$ as a set of continuous orthogonal basis functions which together represent the space $E(\mathbf{q};\, \mathbf{c})$. In this formulation, the diffusion signal is represented as the basis coefficients c and the q-space wave vector $\mathbf{q} = |\mathbf{q}|\mathbf{g}$, where the gradient direction $\mathbf{g}$ is related to the b-value as $|\mathbf{q}| = \sqrt{b/(\Delta - \delta/3)}/2\pi$. We describe the estimation of the basis coefficients $\mathbf{c}$ here, with the formulation of the MAPMRI basis functions detailed elsewhere (Fick et al., 2016). Briefly, the fitting of $\mathbf{c}$ is regularized such that $\hat{E}(\mathbf{q};\, \mathbf{c})$ uses Laplacian regularization to smoothly interpolate between the measured q-space points, and λ represents the regularization weight (Fick et al., 2016).

$$\mathrm{argmin}_{\mathrm{c}} \int_{\mathbb{R}^3} \left[E(\mathbf{q}) - \hat{E}(\mathbf{q};\mathbf{c})\right]^2 d\mathbf{q} + \lambda \int_{\mathbb{R}^3} \left[\nabla^2 \hat{E}(\mathbf{q};\mathbf{c})\right]^2 d\mathbf{q}$$

After $\mathbf{c}$ is determined, the MAPMRI basis represents both the dMRI signal and the diffusion propagator. In the current study, we considered the q-space indices return-to-origin probability (RTOP), return-to-axis probability (RTAP), and return-to-plane probability (RTPP) (Fick et al., 2016; Ozarslan et al., 2013).

Supplementary Tables and Figures

Table S1. Mean and Standard Deviation of Sample Descriptives

	Male	Female	*P*-Value
Sample Size	7442	8186	-
Age (Years)	63.6 ± 7.6	62.2 ± 7.3	< 0.001
Waist/Hip Ratio	0.92 ± 0.06	0.81 ± 0.06	< 0.001
Socioeconomic Status (Townsend Index)	-2.1 ± 2.64	-2.0 ± 2.6	*ns*
Education (College / No College)	5388 / 2054	5154 / 3032	< 0.001

Table S2. White Matter Regions of Interest Analyzed from the Johns Hopkins University (JHU) Atlas

Abbreviation	Region of Interest Name
CGC	Cingulum (cingulate)
CGH	Cingulum (hippocampal)
CR	*Corona radiata*
CST	Corticospinal tract
EC	External capsule
FX	Fornix (body)
FXST	Fornix *(crus)* / *stria terminalis*
IC	Internal capsule
PTR	Posterior thalamic radiation
SFO	Superior fronto-occpital fasciculus
SLF	Superior longitudinal fasiculus
SS	*Sagittal stratum*
TAP	Tapetum
UNC	Uncinate fasciculus

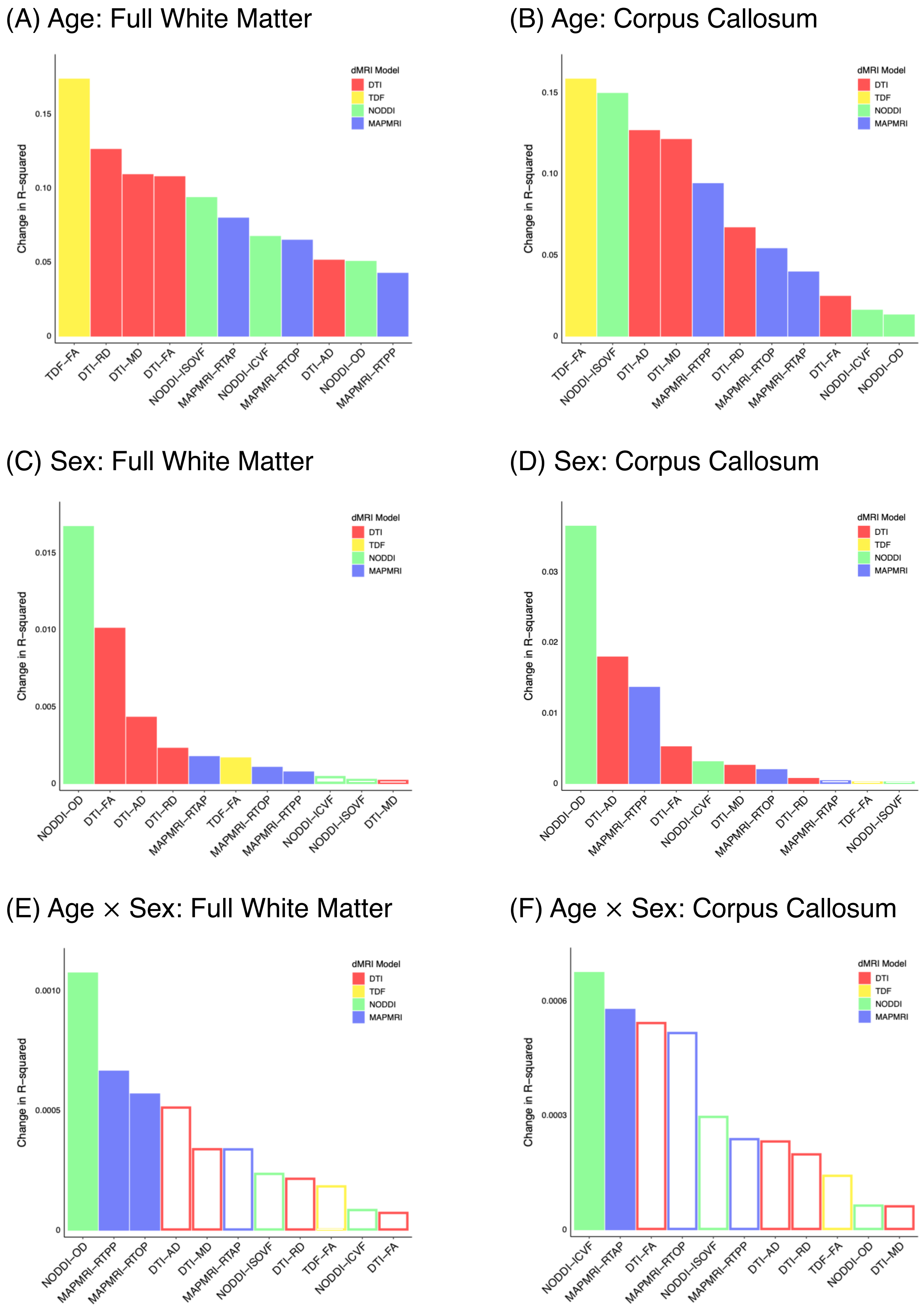

Figure S1. Effect of age (A-B), participant sex (C-D), and their interaction (E-F) on full white matter and corpus callosum microstructure. Age was modeled as a continuous variable using fractional polynomials. Filled bars indicate a significant association at $p<0.01$, whereas hollow bars indicate the association did not attain statistical significance at $p<0.01$.

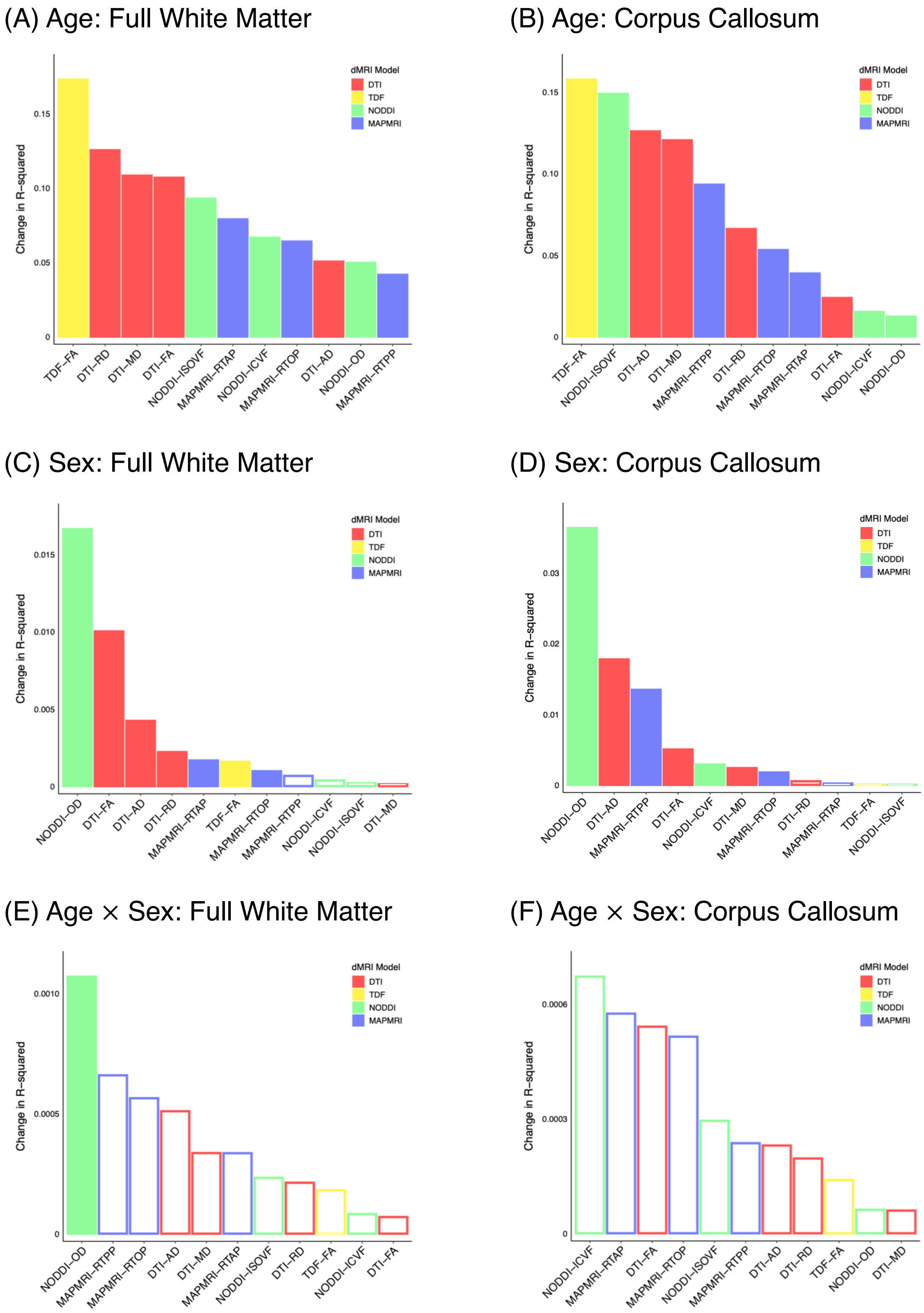

Figure S2. Effect of age (A-B), participant sex (C-D), and their interaction (E-F) on full white matter and corpus callosum microstructure. Age was modeled as a continuous variable using fractional polynomials. Filled bars indicate a significant association at $p<0.001$, whereas hollow bars indicate the association did not attain statistical significance at $p<0.001$.

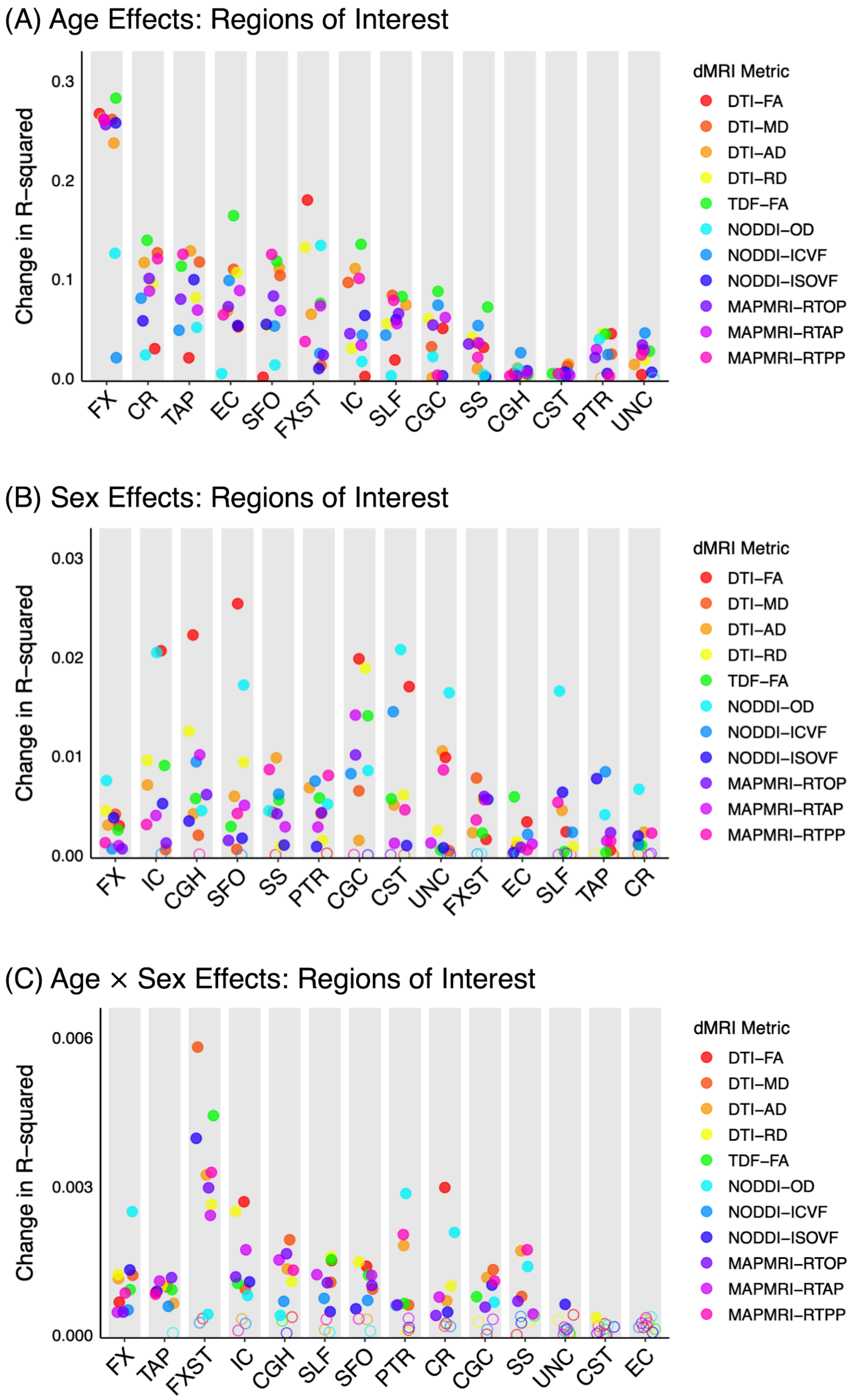

Figure S3. Effect of age (A), participant sex (B), and their interaction (C) on white matter microstructure when modeling age with fractional polynomials. Filled circles indicate the association was significant at $p<0.05$ after FDR-correction for the number of regions. Regions are ordered by the number of significant metrics, followed by the mean effect size. CGC = cingulum (cingulate), CGH = cingulum (hippocampal), CR = *corona radiata*, CST = corticospinal tract, EC = external capsule, FX = fornix (body), FXST = fornix (*crus*) / *stria terminalis*, IC = internal capsule, PTR = posterior thalamic radiation, SFO = superior fronto-occpital fasciculus, SLF = superior longitudinal fasiculus, SS = *sagittal stratum*, TAP = tapetum, UNC = uncinate fasciculus.

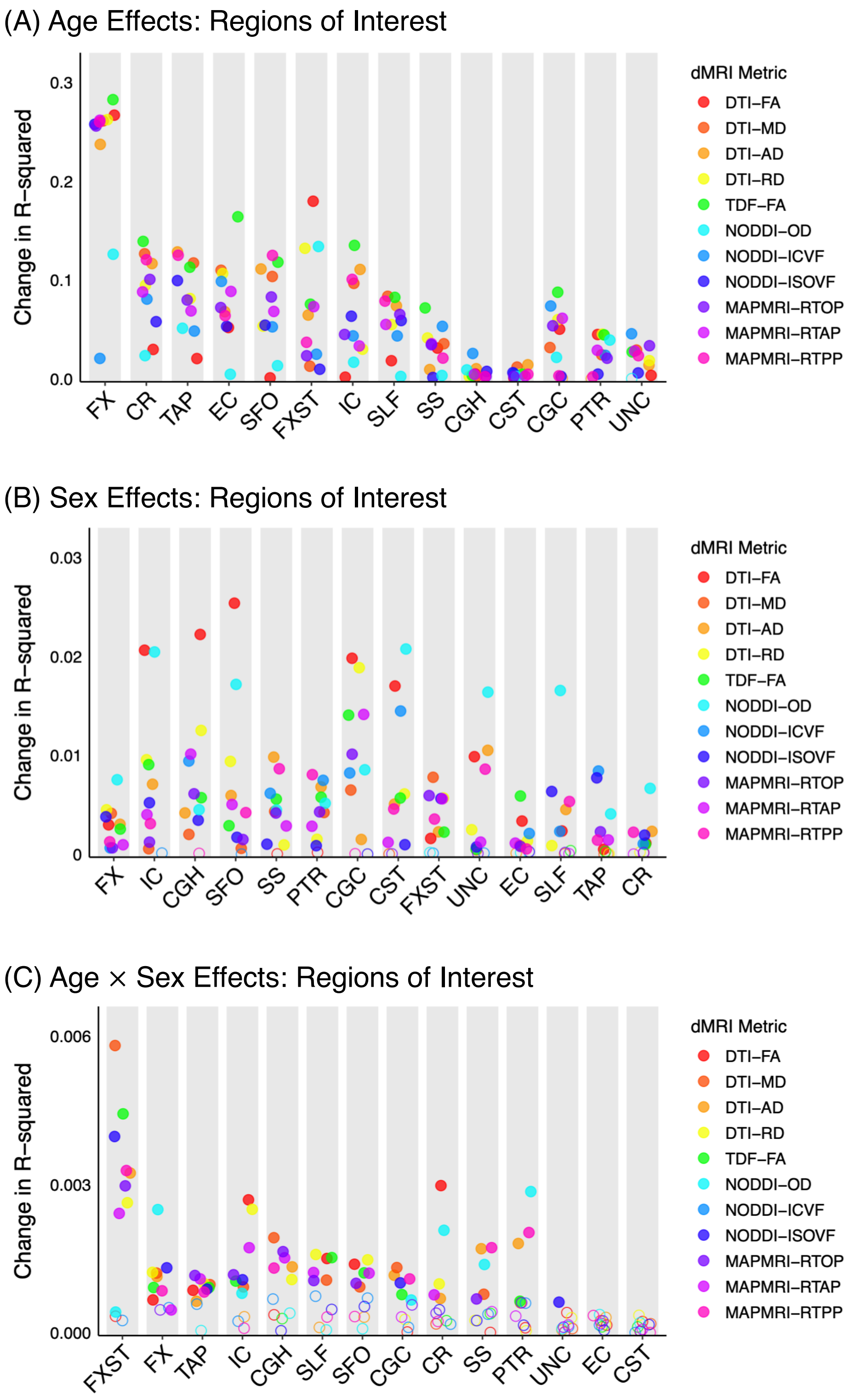

Figure S4. Effect of age (A), participant sex (B), and their interaction (C) on white matter microstructure when modeling age with fractional polynomials. Filled circles indicate the association was significant at $p<0.01$ after FDR-correction for the number of regions. Regions are ordered by the number of significant metrics, followed by the mean effect size. CGC = cingulum (cingulate), CGH = cingulum (hippocampal), CR = *corona radiata*, CST = corticospinal tract, EC = external capsule, FX = fornix (body), FXST = fornix (*crus*) / *stria terminalis*, IC = internal capsule, PTR = posterior thalamic radiation, SFO = superior fronto-occpital fasciculus, SLF = superior longitudinal fasiculus, SS = *sagittal stratum*, TAP = tapetum, UNC = uncinate fasciculus.

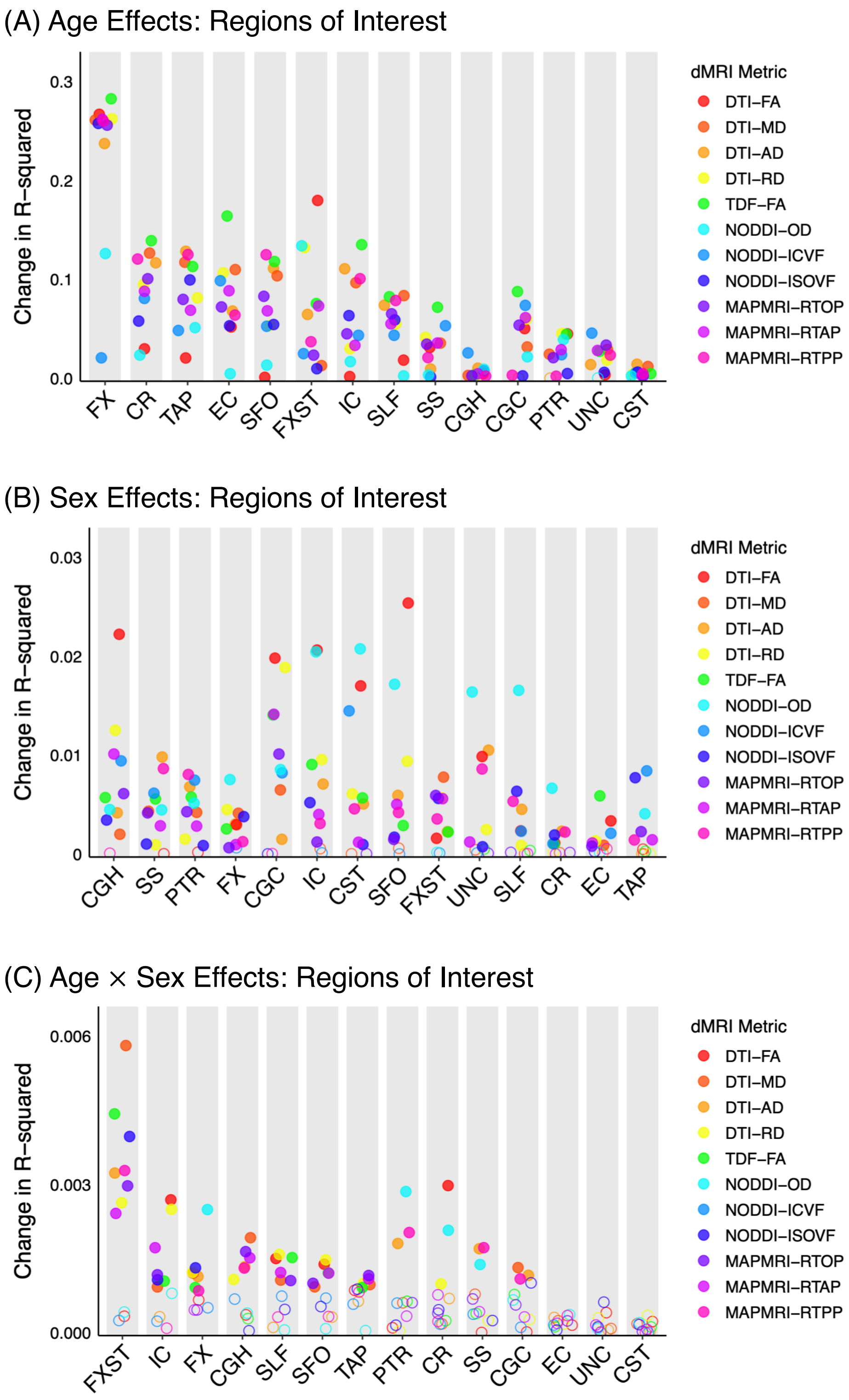

Figure S5. Effect of age (A), participant sex (B), and their interaction (C) on white matter microstructure when modeling age with fractional polynomials. Filled circles indicate the association was significant at $p<0.001$ after FDR-correction for the number of regions. Regions are ordered by the number of significant metrics, followed by the mean effect size. CGC = cingulum (cingulate), CGH = cingulum (hippocampal), CR = *corona radiata*, CST = corticospinal tract, EC = external capsule, FX = fornix (body), FXST = fornix (*crus*) / *stria terminalis*, IC = internal capsule, PTR = posterior thalamic radiation, SFO = superior fronto-occpital fasciculus, SLF = superior longitudinal fasiculus, SS = *sagittal stratum*, TAP = tapetum, UNC = uncinate fasciculus.

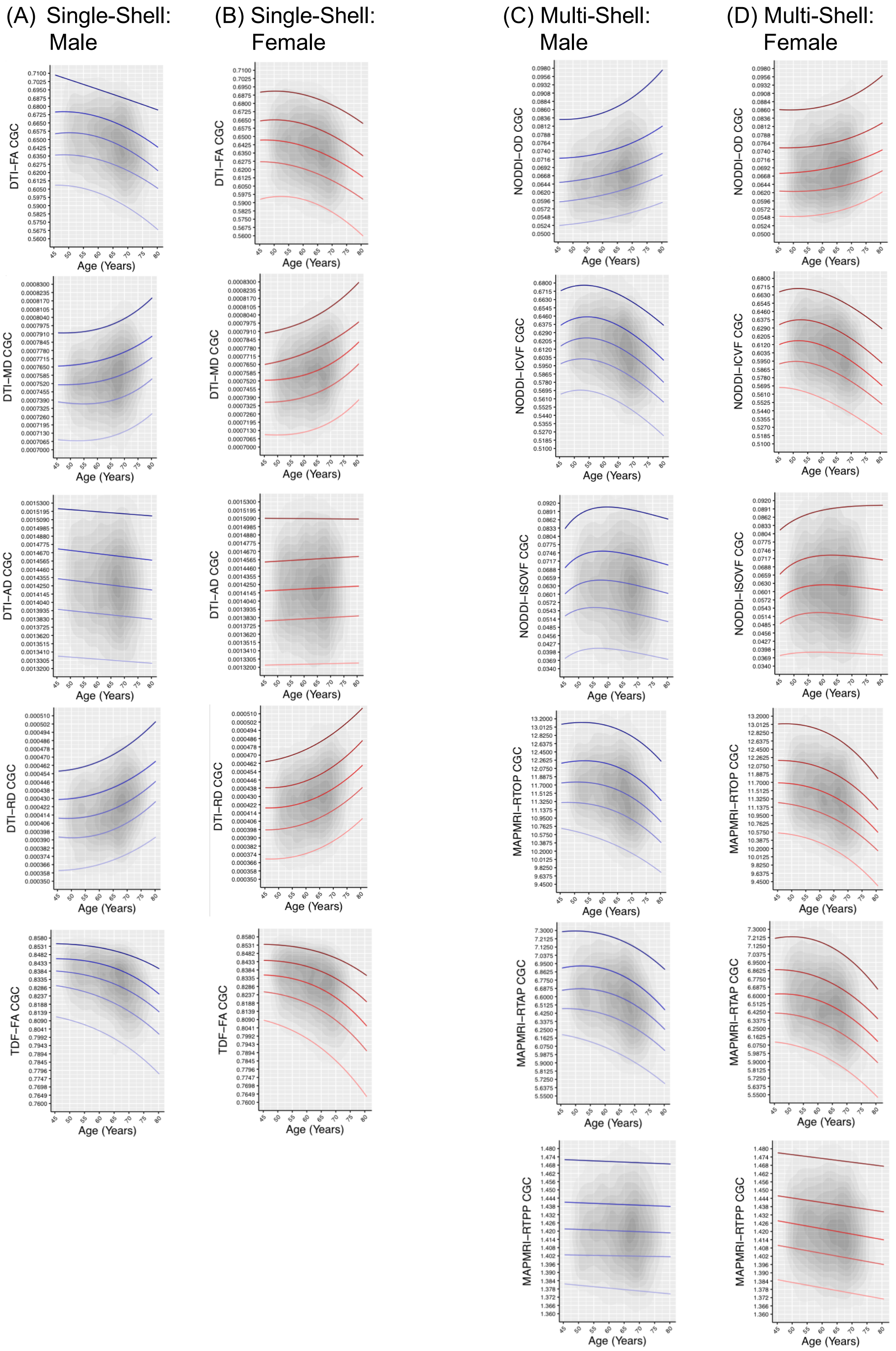

Figure S6. Normative centile reference curves calculated for the cingulum (cingulate) for single-shell dMRI metrics in (A) males and (B) females, and multi-shell dMRI metrics in (C) males and (D) females. Solid colored lines, ordered from lightest to darkest, indicate the following centiles: 5th, 25th, 50th, 75th, 95th; blue lines indicate male participants, and red lines indicate female participants. Gray overlay reflects kernel density (darker=greater degree of data point overlap). CGC = cingulum (cingulate).

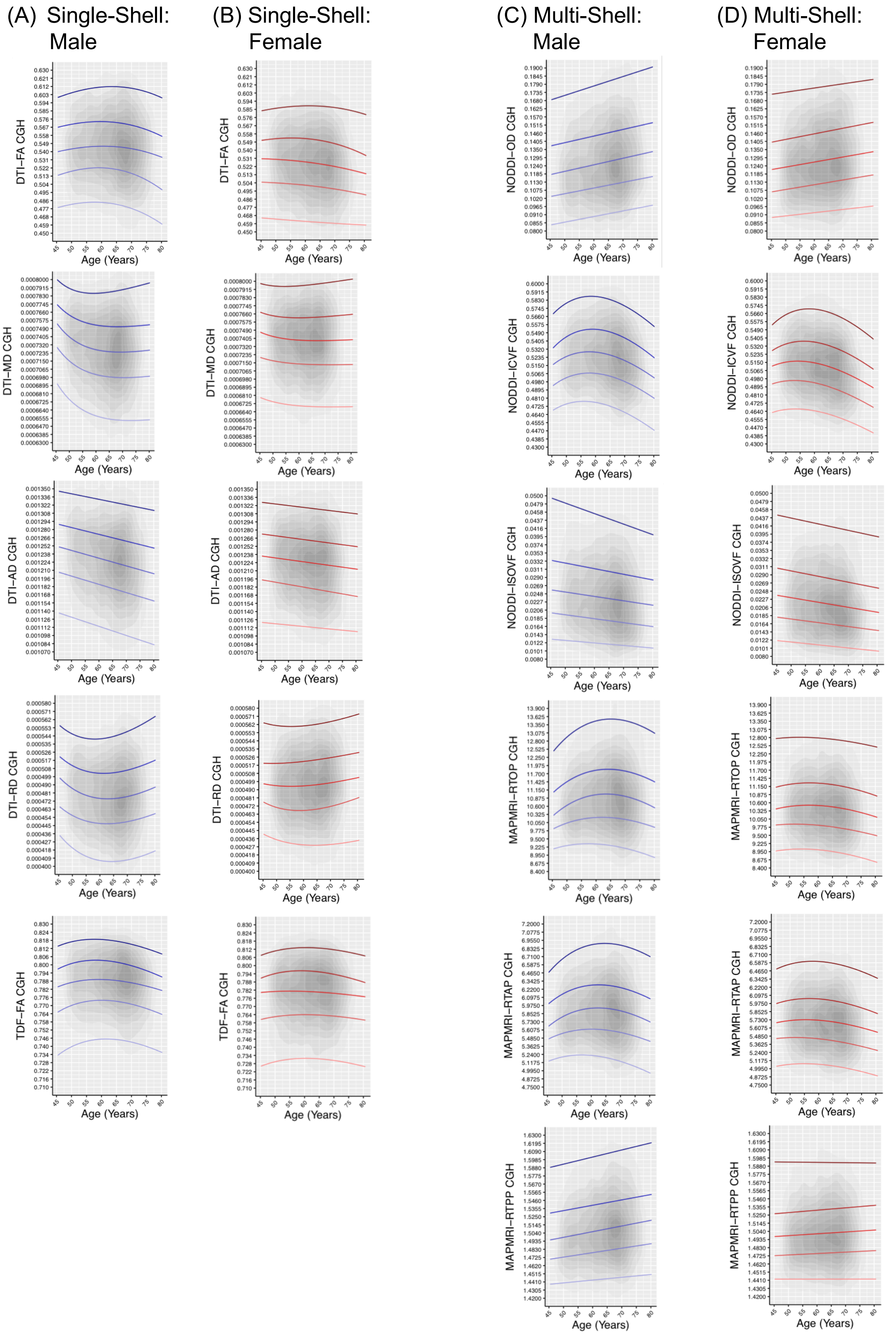

Figure S7. Normative centile reference curves calculated for the cingulum (hippocampal) for single-shell dMRI metrics in (A) males and (B) females, and multi-shell dMRI metrics in (C) males and (D) females. Solid colored lines, ordered from lightest to darkest, indicate the following centiles: 5th, 25th, 50th, 75th, 95th; blue lines indicate male participants, and red lines indicate female participants. Gray overlay reflects kernel density (darker=greater degree of data point overlap). CGH = cingulum (hippocampal).

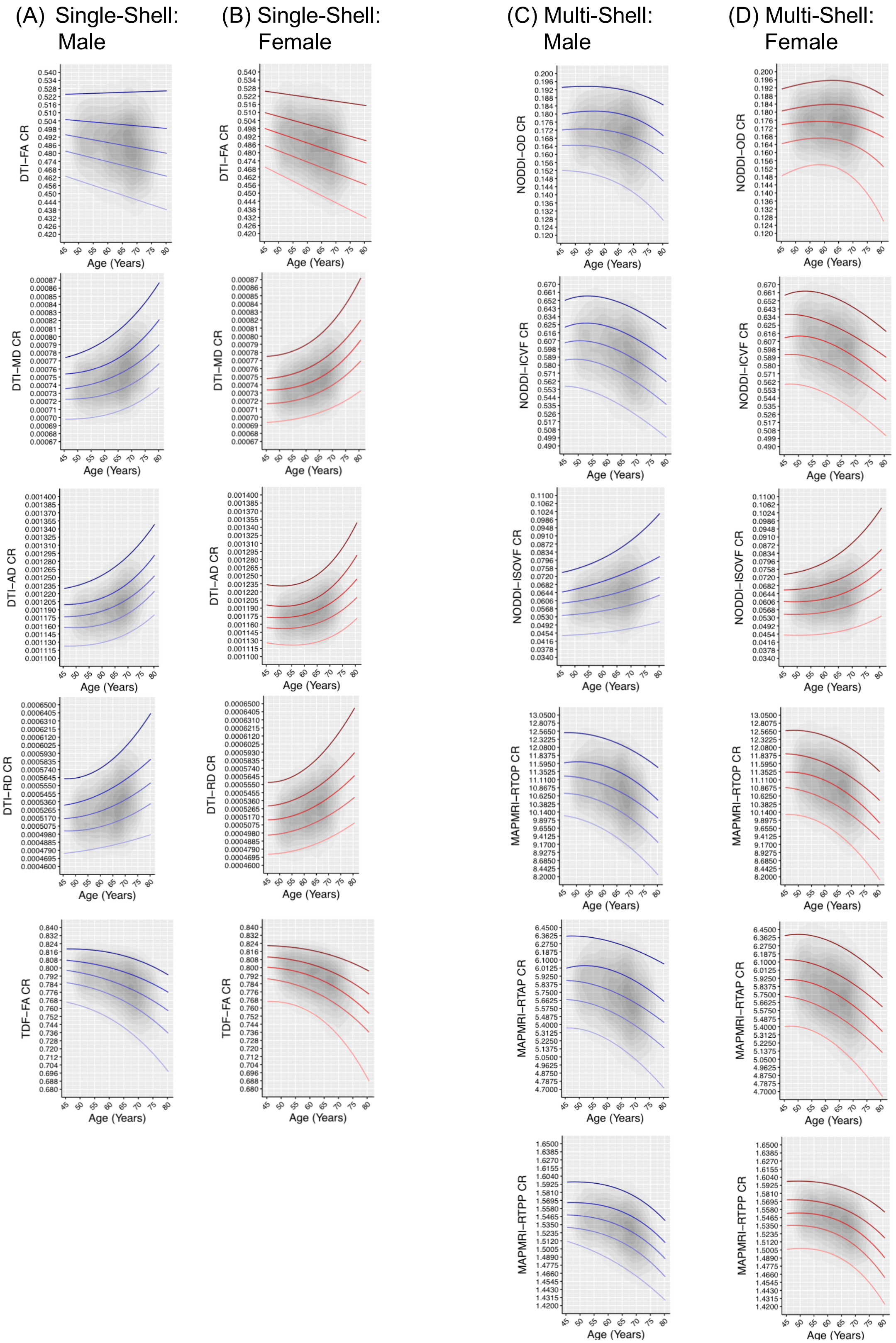

Figure S8. Normative centile reference curves calculated for the *corona radiata* for single-shell dMRI metrics in (A) males and (B) females, and multi-shell dMRI metrics in (C) males and (D) females. Solid colored lines, ordered from lightest to darkest, indicate the following centiles: 5th, 25th, 50th, 75th, 95th; blue lines indicate male participants, and red lines indicate female participants. Gray overlay reflects kernel density (darker=greater degree of data point overlap). CR = *corona radiata*.

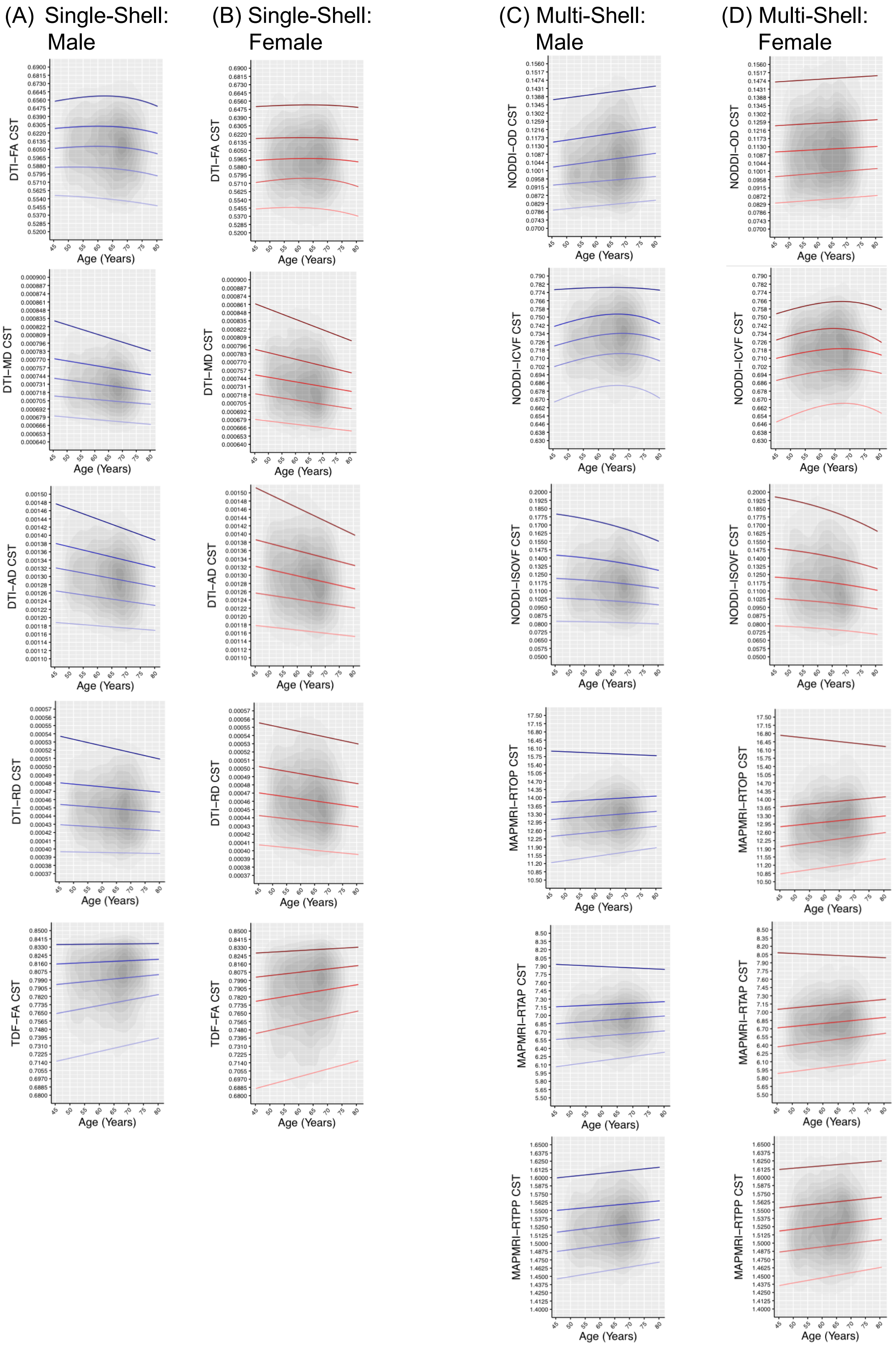

Figure S9. Normative centile reference curves calculated for the corticospinal tract for single-shell dMRI metrics in (A) males and (B) females, and multi-shell dMRI metrics in (C) males and (D) females. Solid colored lines, ordered from lightest to darkest, indicate the following centiles: 5th, 25th, 50th, 75th, 95th; blue lines indicate male participants, and red lines indicate female participants. Gray overlay reflects kernel density (darker=greater degree of data point overlap). CST = corticospinal tract.

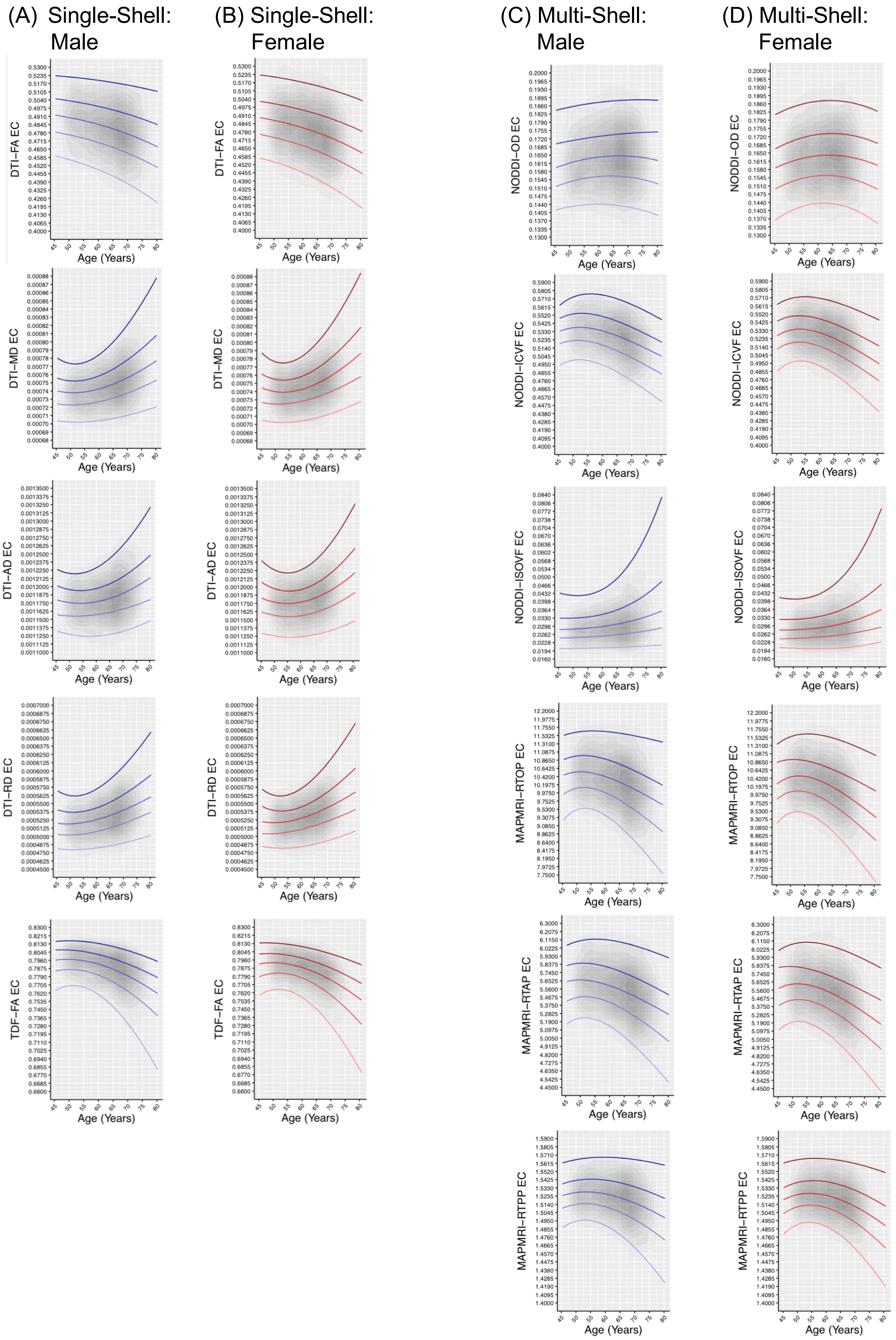

Figure S10. Normative centile reference curves calculated for the external capsule for single-shell dMRI metrics in (A) males and (B) females, and multi-shell dMRI metrics in (C) males and (D) females. Solid colored lines, ordered from lightest to darkest, indicate the following centiles: 5th, 25th, 50th, 75th, 95th; blue lines indicate male participants, and red lines indicate female participants. Gray overlay reflects kernel density (darker=greater degree of data point overlap). EC = external capsule.

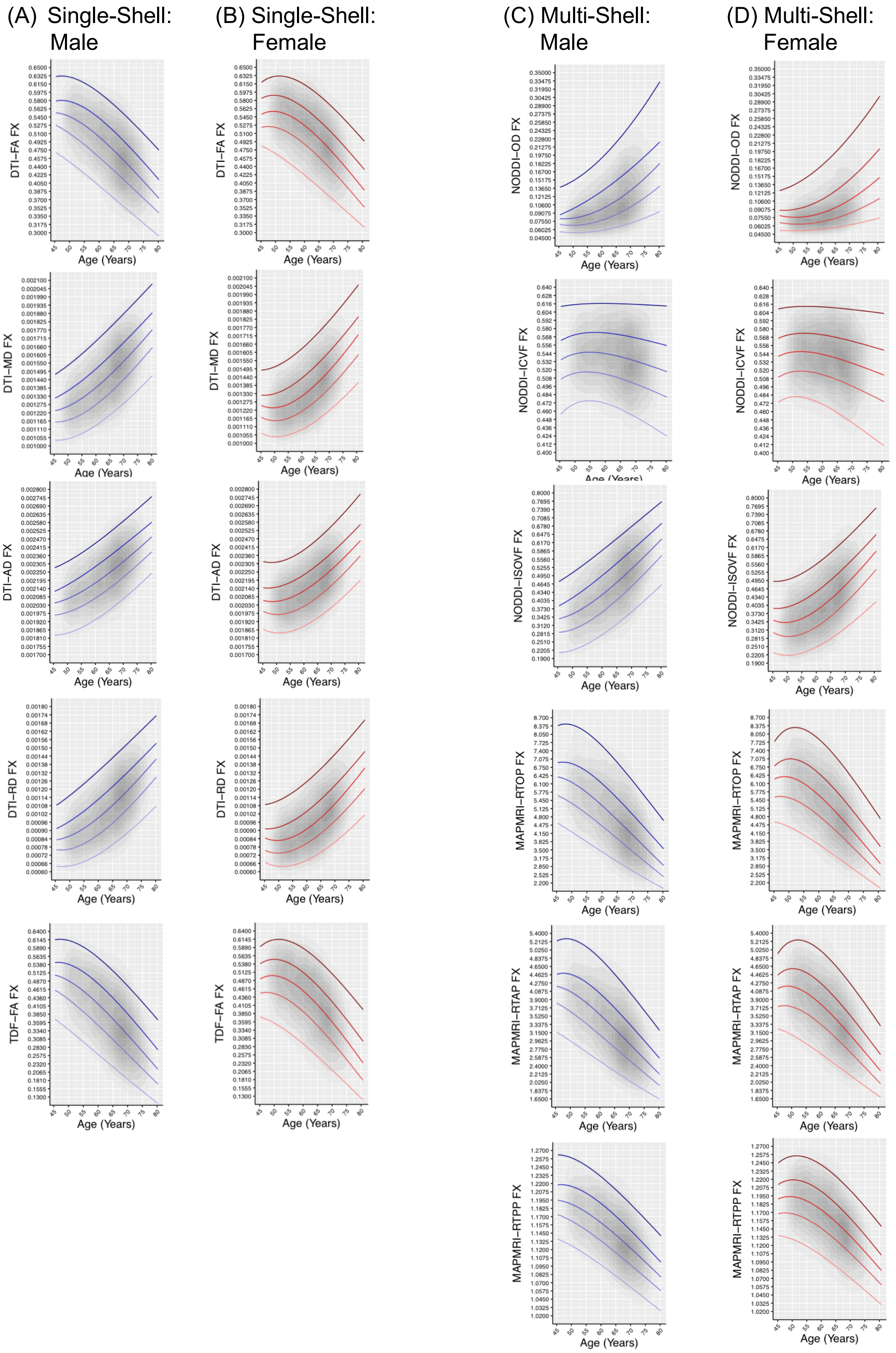

Figure S11. Normative centile reference curves calculated for the fornix (body) for single-shell dMRI metrics in (A) males and (B) females, and multi-shell dMRI metrics in (C) males and (D) females. Solid colored lines, ordered from lightest to darkest, indicate the following centiles: 5th, 25th, 50th, 75th, 95th; blue lines indicate male participants, and red lines indicate female participants. Gray overlay reflects kernel density (darker=greater degree of data point overlap). FX = fornix (body).

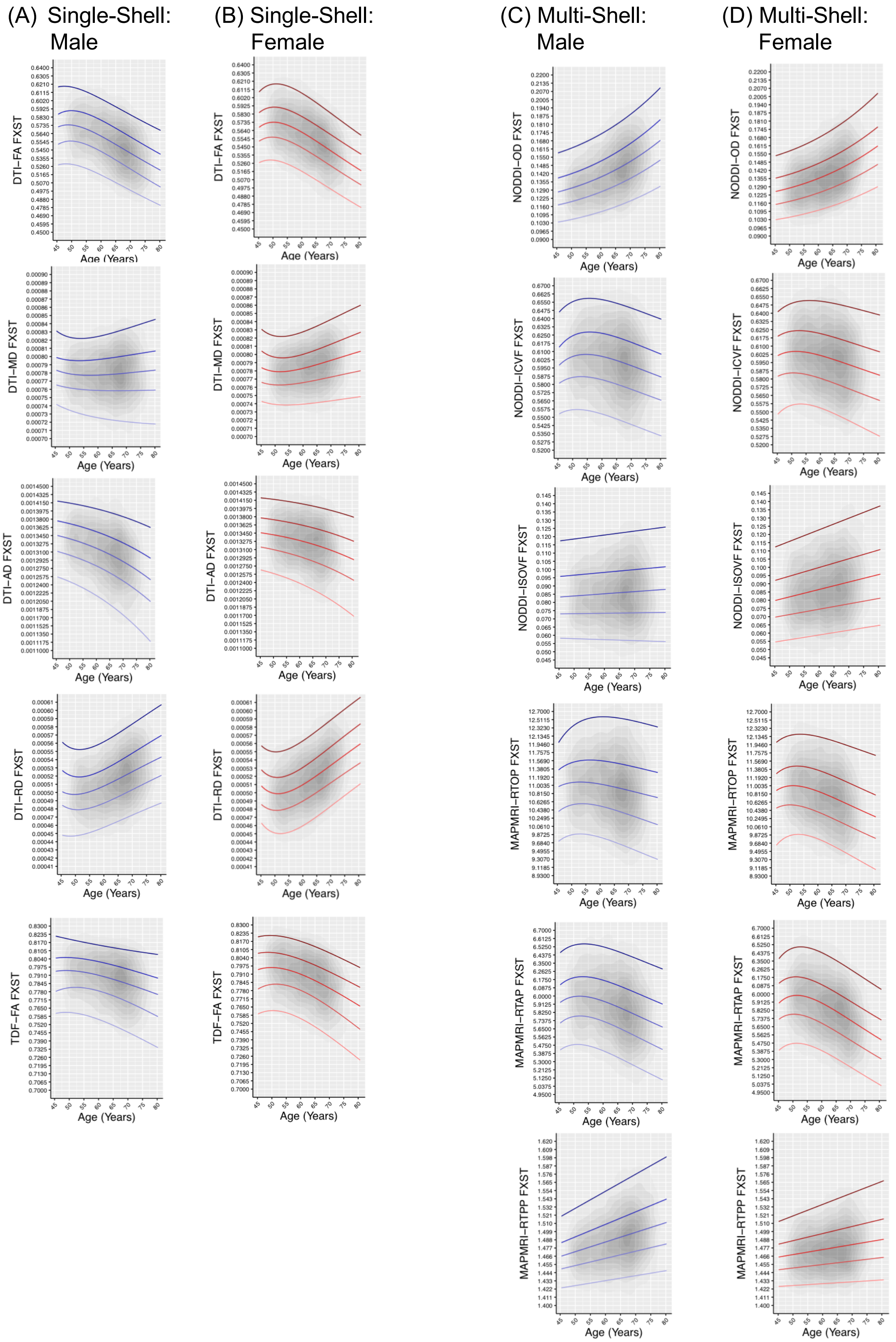

Figure S12. Normative centile reference curves calculated for the fornix (*crus*) / *stria terminalis* for single-shell dMRI metrics in (A) males and (B) females, and multi-shell dMRI metrics in (C) males and (D) females. Solid colored lines, ordered from lightest to darkest, indicate the following centiles: 5th, 25th, 50th, 75th, 95th; blue lines indicate male participants, and red lines indicate female participants. Gray overlay reflects kernel density (darker=greater degree of data point overlap). FXST = fornix (*crus*) / *stria terminalis*.

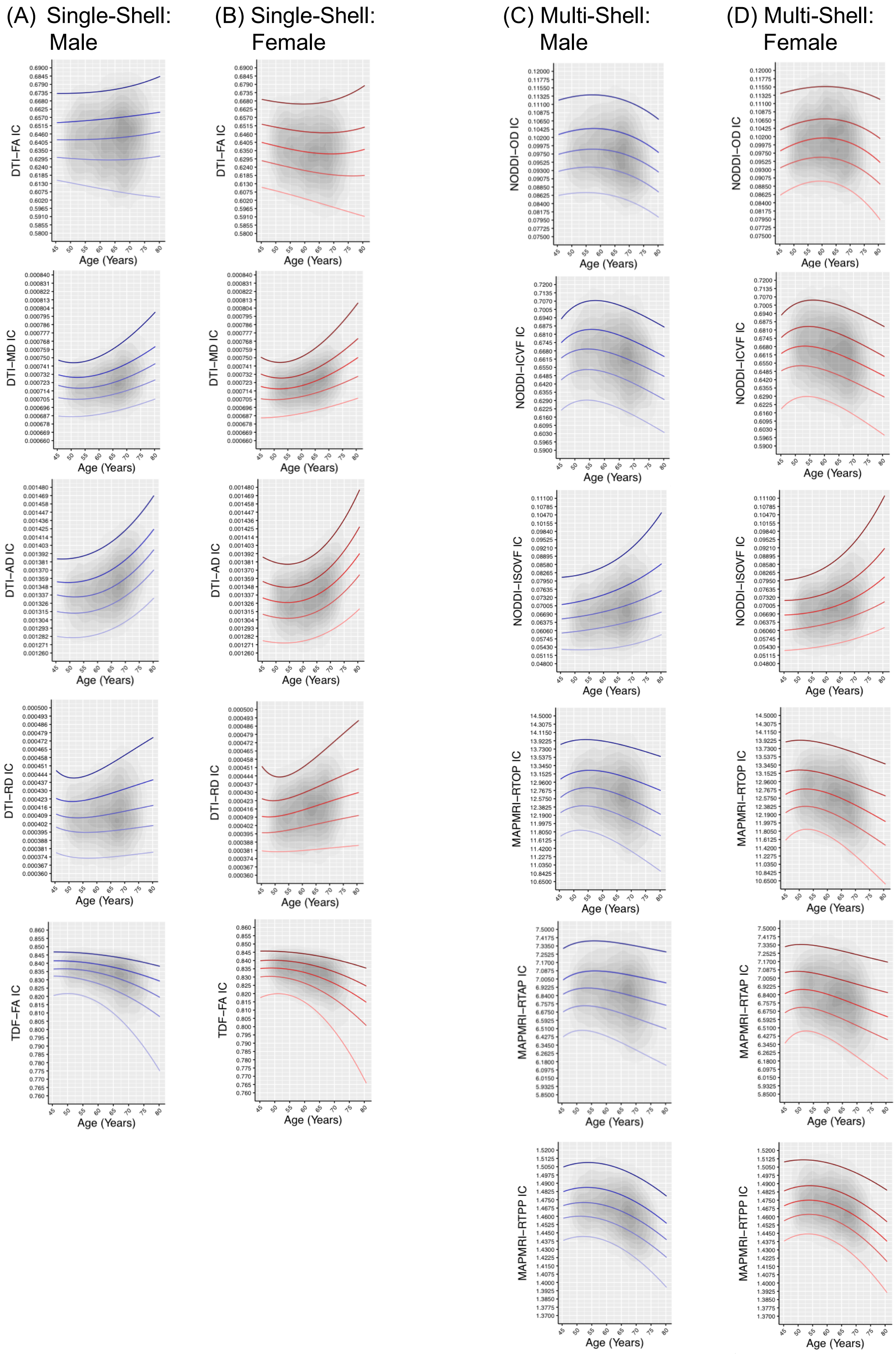

Figure S13. Normative centile reference curves calculated for the internal capsule for single-shell dMRI metrics in (A) males and (B) females, and multi-shell dMRI metrics in (C) males and (D) females. Solid colored lines, ordered from lightest to darkest, indicate the following centiles: 5th, 25th, 50th, 75th, 95th; blue lines indicate male participants, and red lines indicate female participants. Gray overlay reflects kernel density (darker=greater degree of data point overlap). IC = internal capsule.

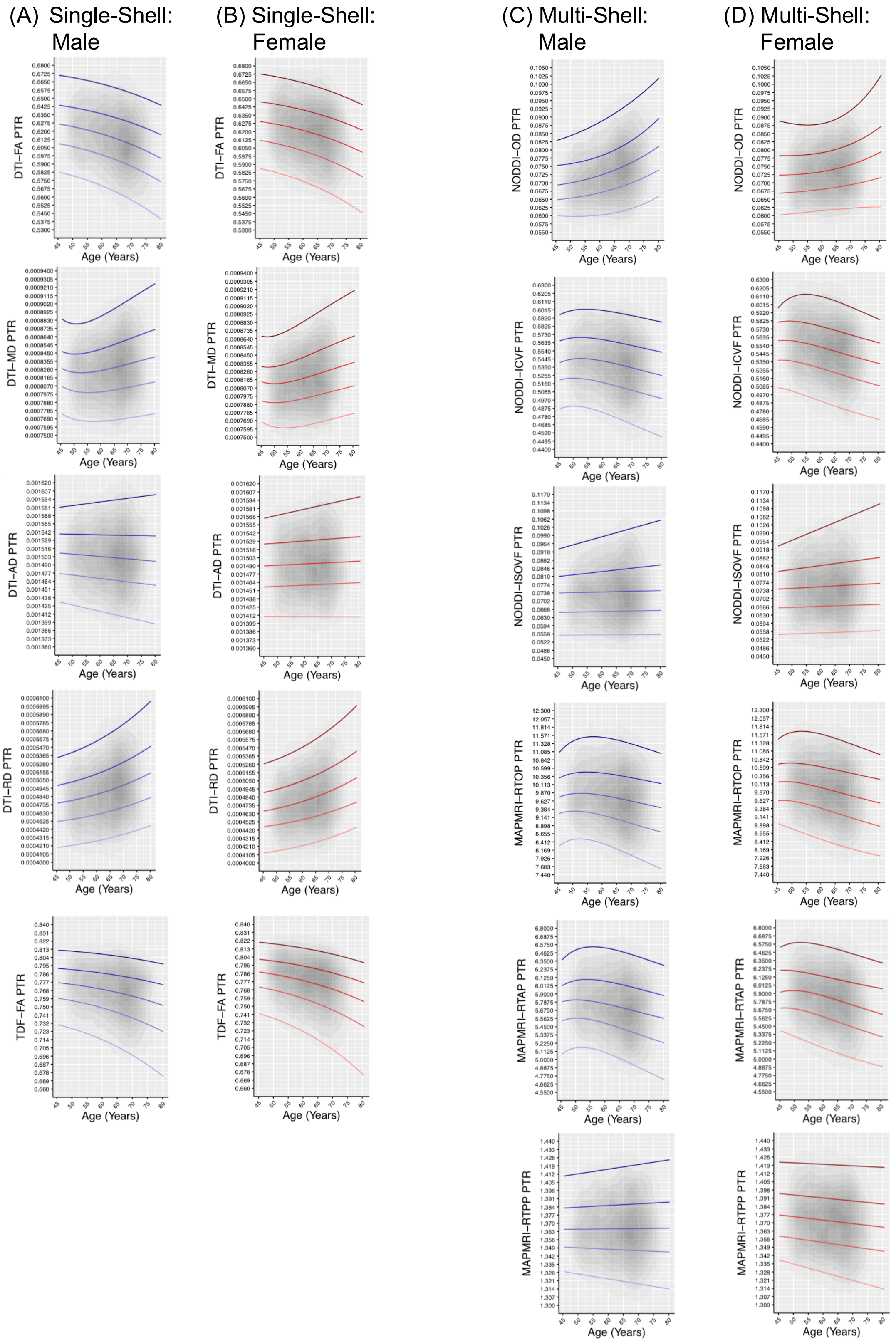

Figure S14. Normative centile reference curves calculated for the posterior thalamic radiation for single-shell dMRI metrics in (A) males and (B) females, and multi-shell dMRI metrics in (C) males and (D) females. Solid colored lines, ordered from lightest to darkest, indicate the following centiles: 5th, 25th, 50th, 75th, 95th; blue lines indicate male participants, and red lines indicate female participants. Gray overlay reflects kernel density (darker=greater degree of data point overlap). PTR = posterior thalamic radiation.

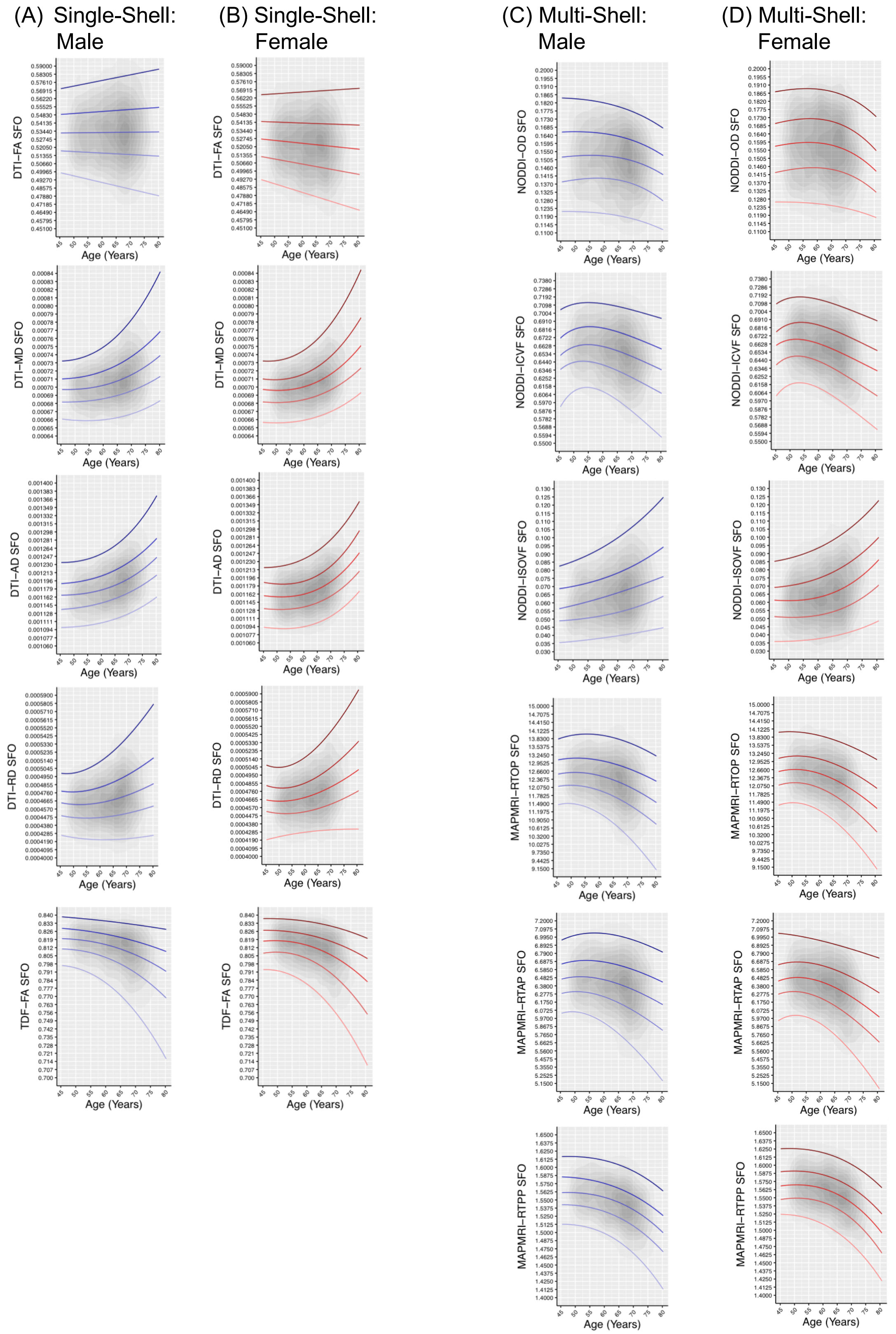

Figure S15. Normative centile reference curves calculated for the superior fronto-occpital fasciculus for single-shell dMRI metrics in (A) males and (B) females, and multi-shell dMRI metrics in (C) males and (D) females. Solid colored lines, ordered from lightest to darkest, indicate the following centiles: 5th, 25th, 50th, 75th, 95th; blue lines indicate male participants, and red lines indicate female participants. Gray overlay reflects kernel density (darker=greater degree of data point overlap). SFO = superior fronto-occpital fasciculus.

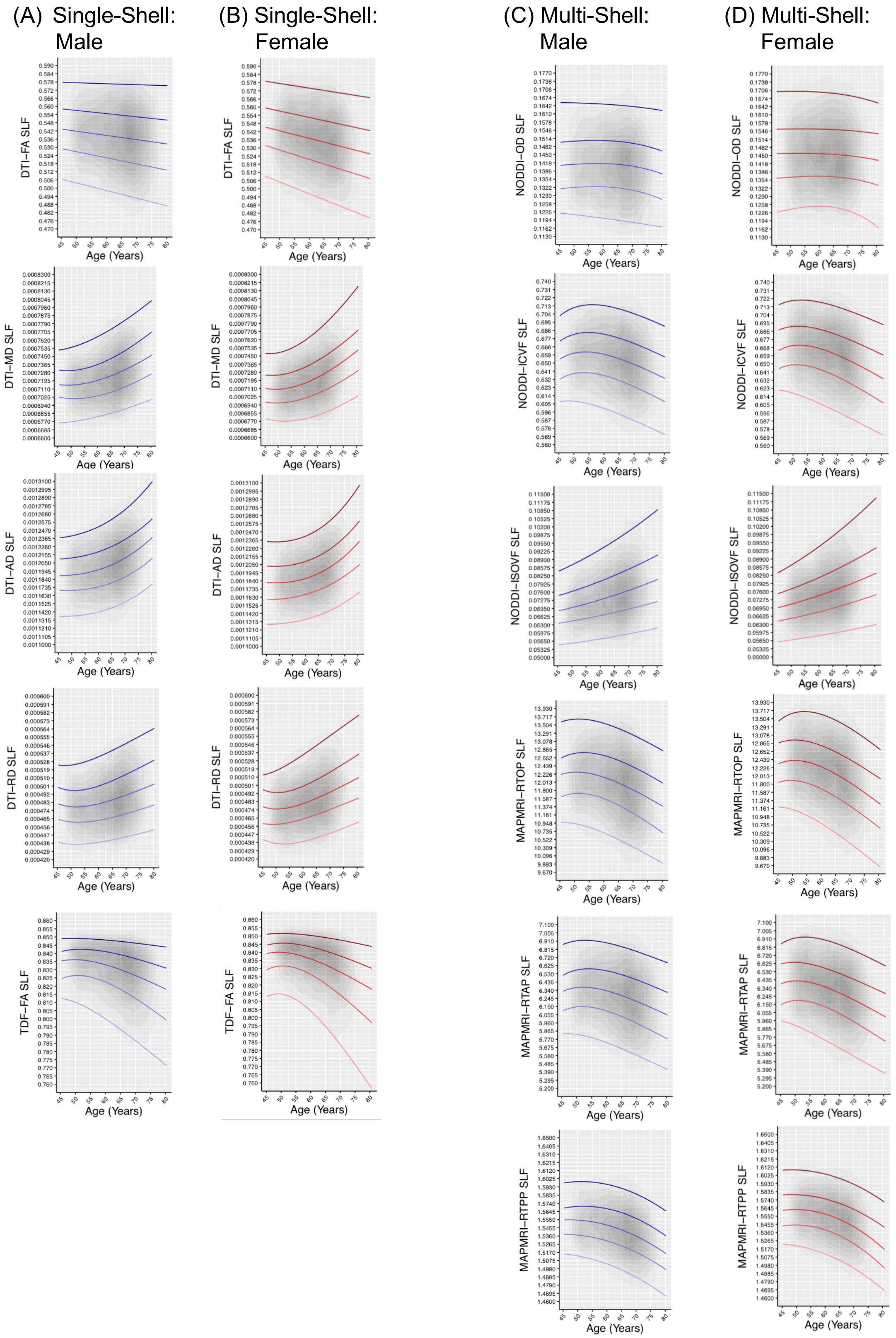

Figure S16. Normative centile reference curves calculated for the superior longitudinal fasciculus for single-shell dMRI metrics in (A) males and (B) females, and multi-shell dMRI metrics in (C) males and (D) females. Solid colored lines, ordered from lightest to darkest, indicate the following centiles: 5th, 25th, 50th, 75th, 95th; blue lines indicate male participants, and red lines indicate female participants. Gray overlay reflects kernel density (darker=greater degree of data point overlap). SLF = superior longitudinal fasciculus.

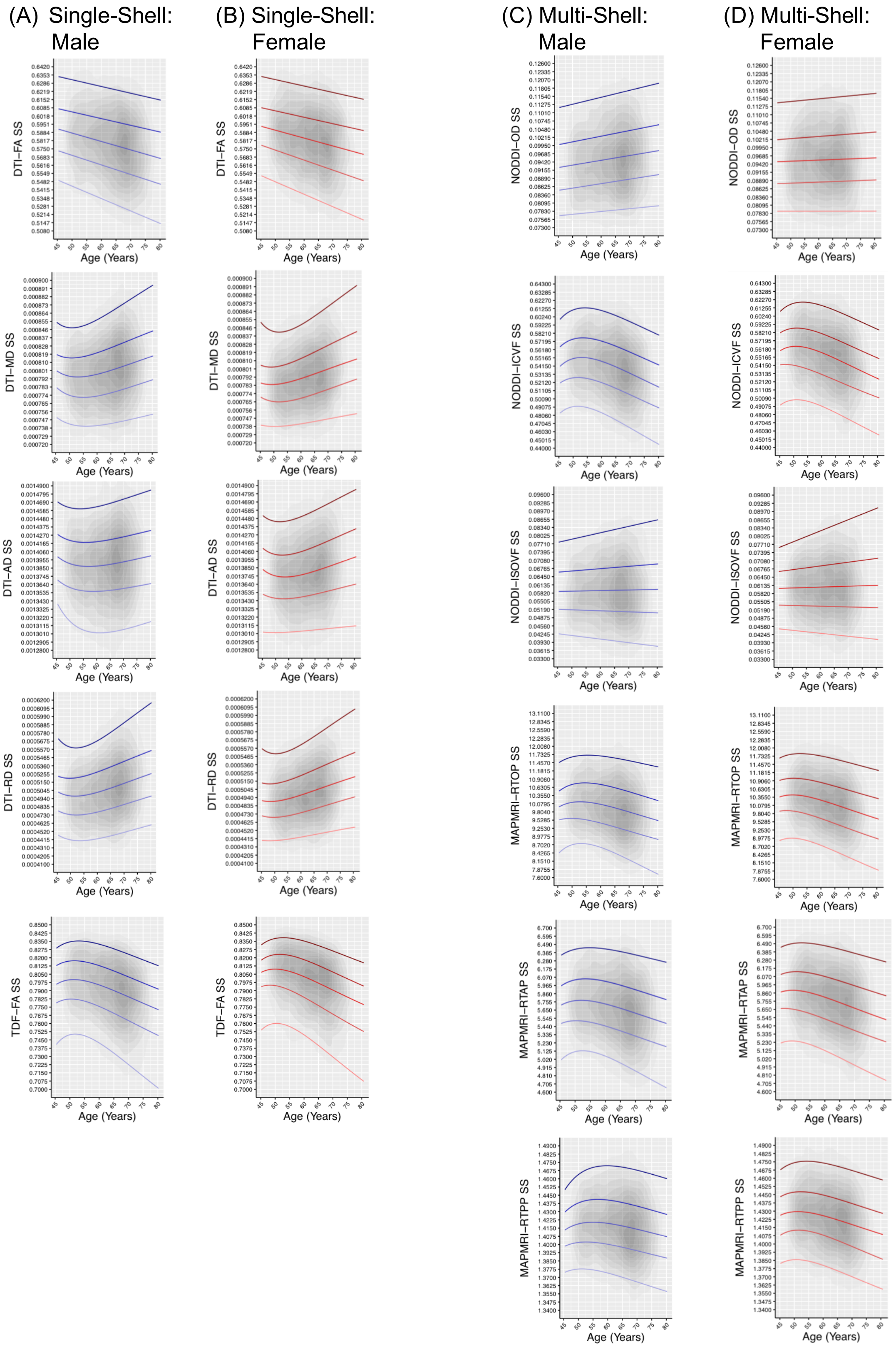

Figure S17. Normative centile reference curves calculated for the *sagittal stratum* for single-shell dMRI metrics in (A) males and (B) females, and multi-shell dMRI metrics in (C) males and (D) females. Solid colored lines, ordered from lightest to darkest, indicate the following centiles: 5th, 25th, 50th, 75th, 95th; blue lines indicate male participants, and red lines indicate female participants. Gray overlay reflects kernel density (darker=greater degree of data point overlap). SS = *sagittal stratum*.

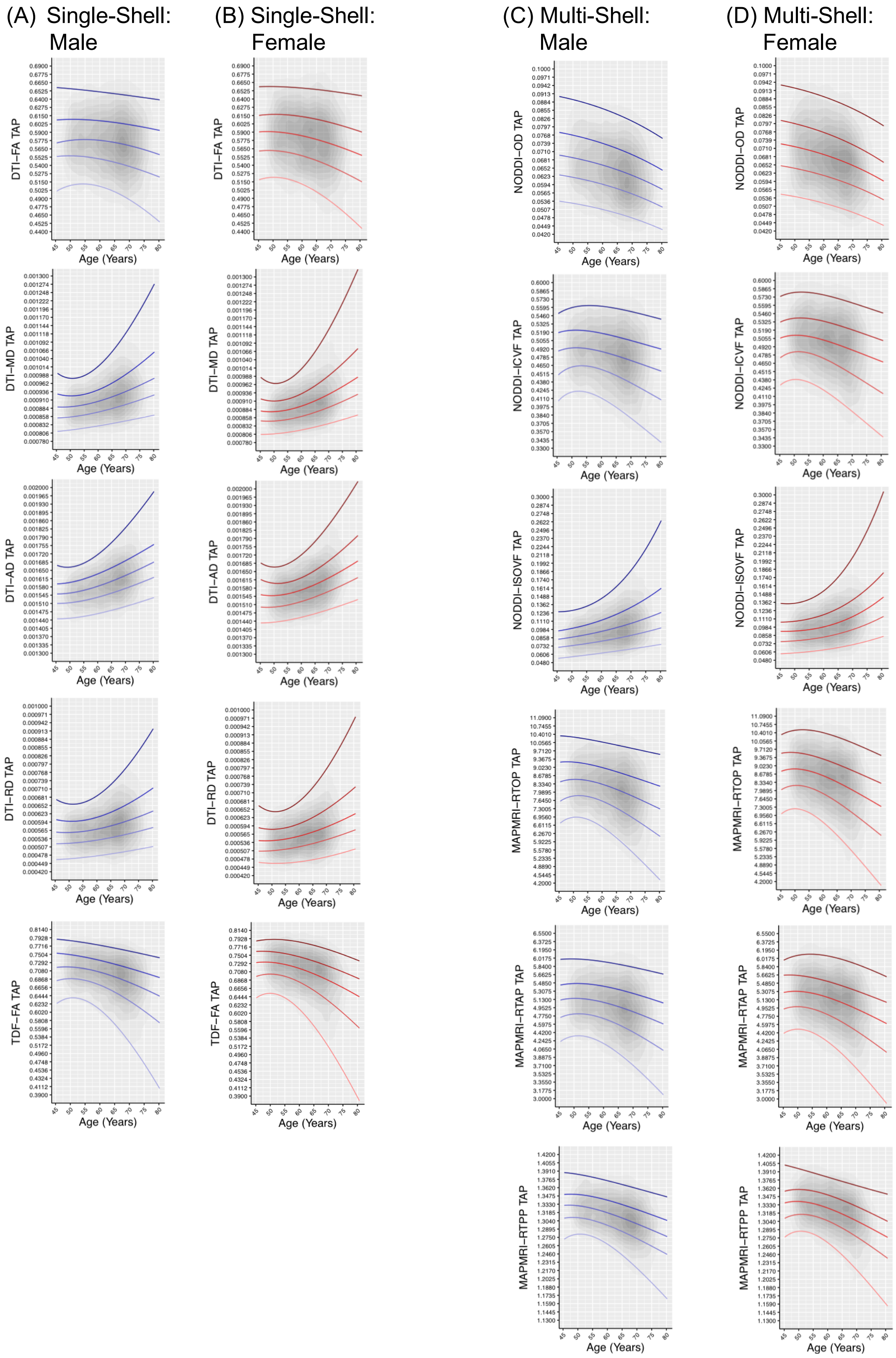

Figure S18. Normative centile reference curves calculated for the tapetum for single-shell dMRI metrics in (A) males and (B) females, and multi-shell dMRI metrics in (C) males and (D) females. Solid colored lines, ordered from lightest to darkest, indicate the following centiles: 5th, 25th, 50th, 75th, 95th; blue lines indicate male participants, and red lines indicate female participants. Gray overlay reflects kernel density (darker=greater degree of data point overlap). TAP = tapetum.

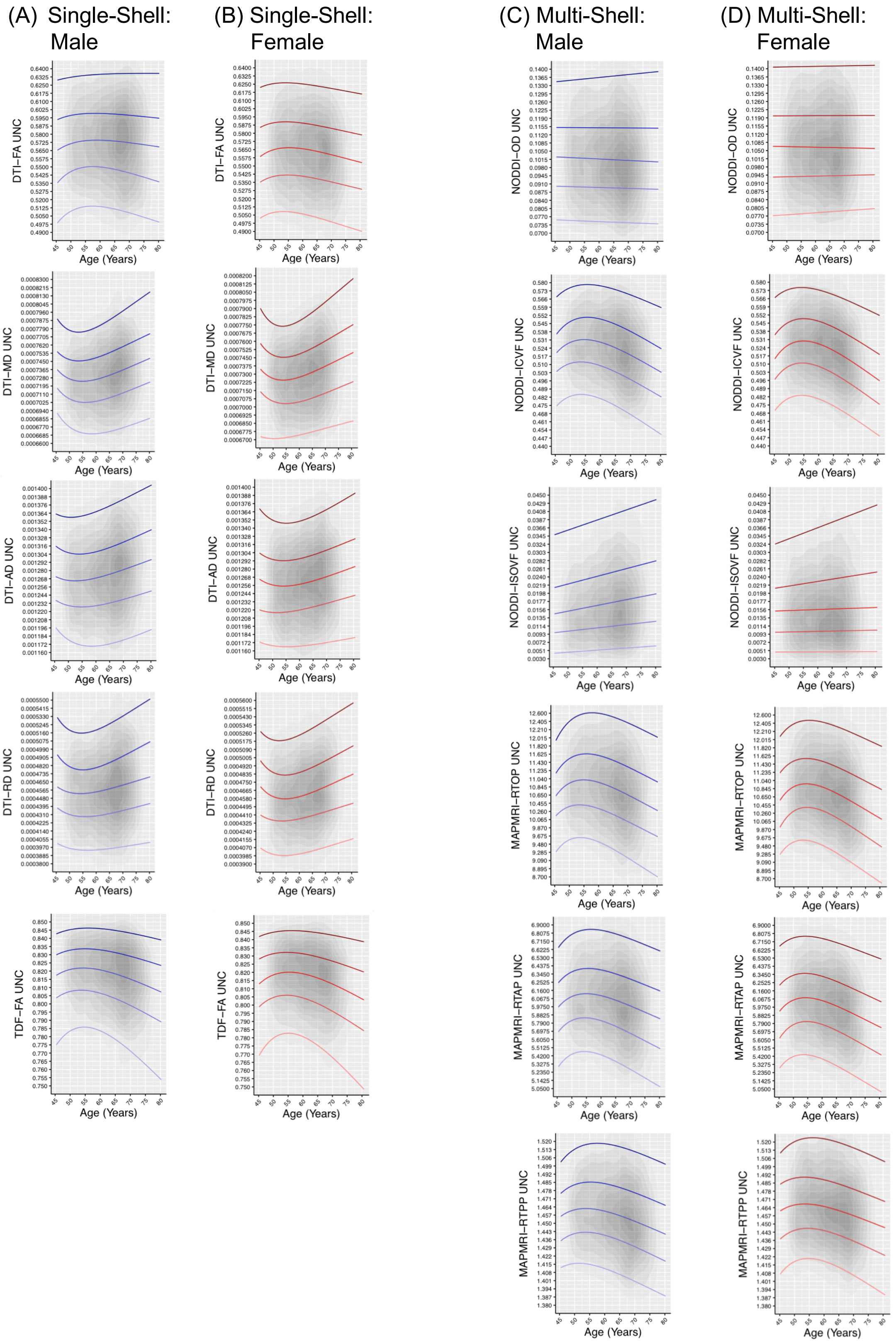

Figure S19. Normative centile reference curves calculated for the uncinate fasciculus for single-shell dMRI metrics in (A) males and (B) females, and multi-shell dMRI metrics in (C) males and (D) females. Solid colored lines, ordered from lightest to darkest, indicate the following centiles: 5th, 25th, 50th, 75th, 95th; blue lines indicate male participants, and red lines indicate female participants. Gray overlay reflects kernel density (darker=greater degree of data point overlap). UNC = uncinate fasciculus.

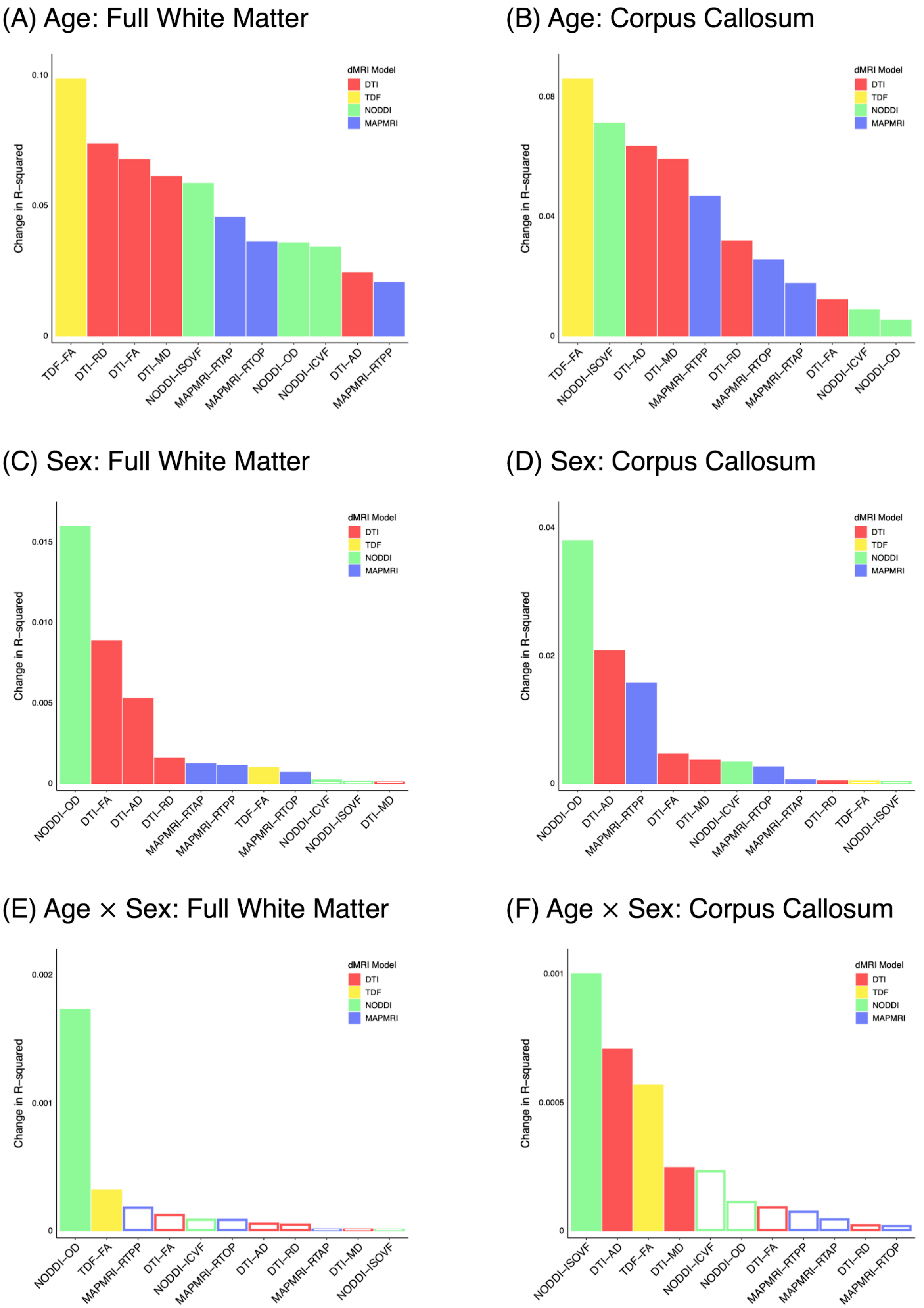

Figure S20. Effect of age (A-B), participant sex (C-D), and their interaction (E-F) on full white matter and corpus callosum white matter microstructure. Age was modeled as a discrete variable by splitting participants into two age groups (≥ 60 years or < 60 years). Filled bars indicate a significant association (*p*<0.05), whereas hollow bars indicate the association did not attain statistical significance.

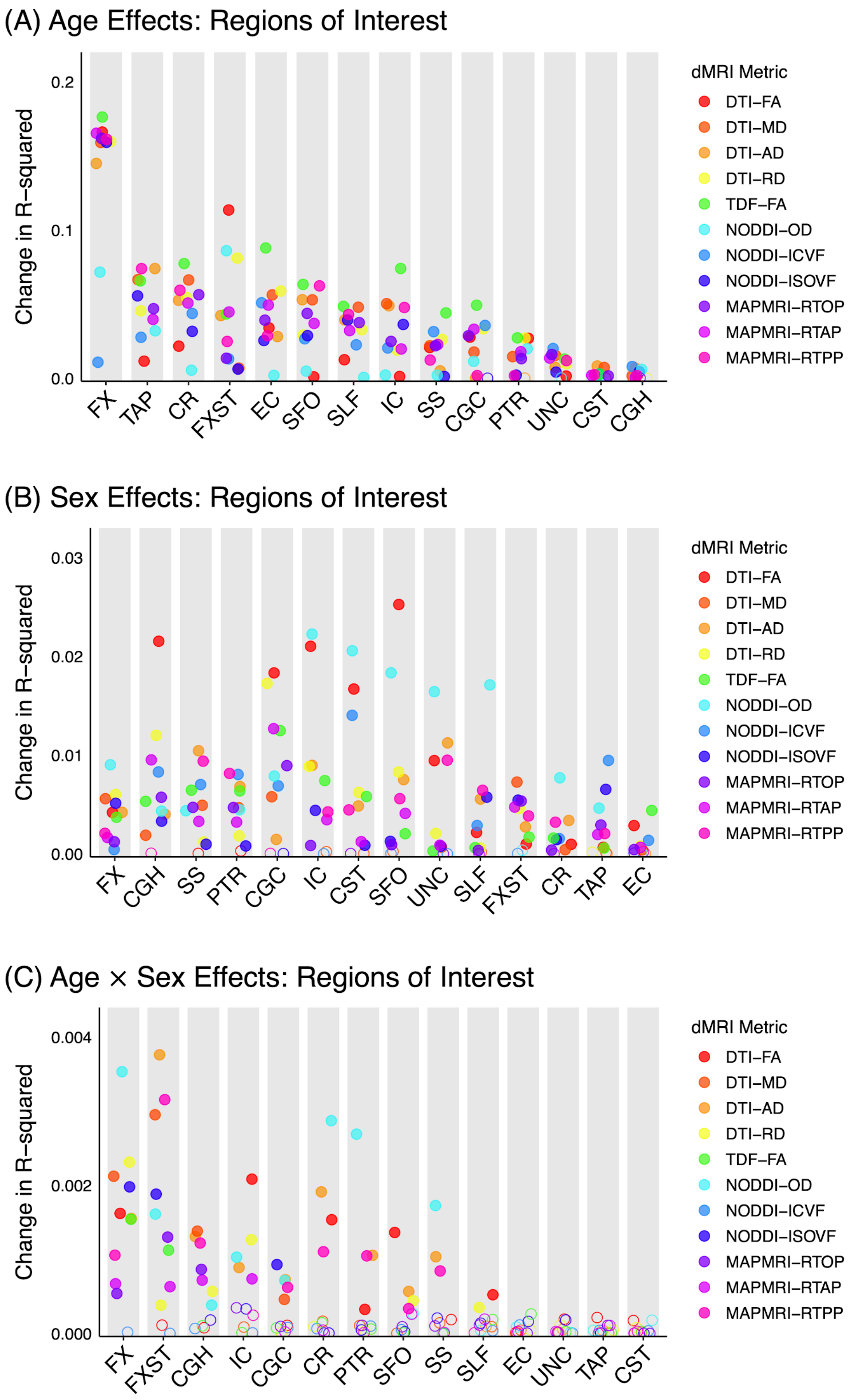

Figure S21. Effect of age (A), participant sex (B), and their interaction (C) on white matter microstructure when modeling age by splitting participants into two age groups (≥ 60 years or < 60 years). Filled circles indicate the association was significant at *p*<0.05 after FDR-correction for the number of regions. Regions are ordered by the number of significant metrics, followed by the mean effect size. CGC = cingulum (cingulate), CGH = cingulum (hippocampal), CR = *corona radiata*, CST = corticospinal tract, EC = external capsule, FX = fornix (body), FXST = fornix (*crus*) / *stria terminalis*, IC = internal capsule, PTR = posterior thalamic radiation, SFO = superior fronto-occpital fasciculus, SLF = superior longitudinal fasiculus, SS = *sagittal stratum*, TAP = tapetum, UNC = uncinate fasciculus.

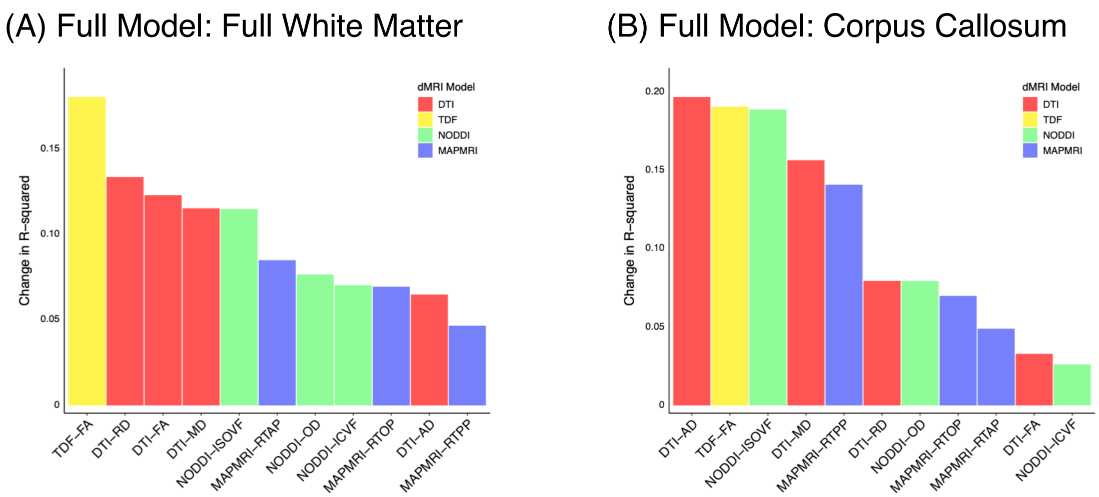

Figure S22. Total variance explained by the full model including age, sex, their interaction, and nuisance covariates. Age was modeled as a continuous variable using fractional polynomials. Filled bars indicate the full model attained statistical significance (p<0.05).

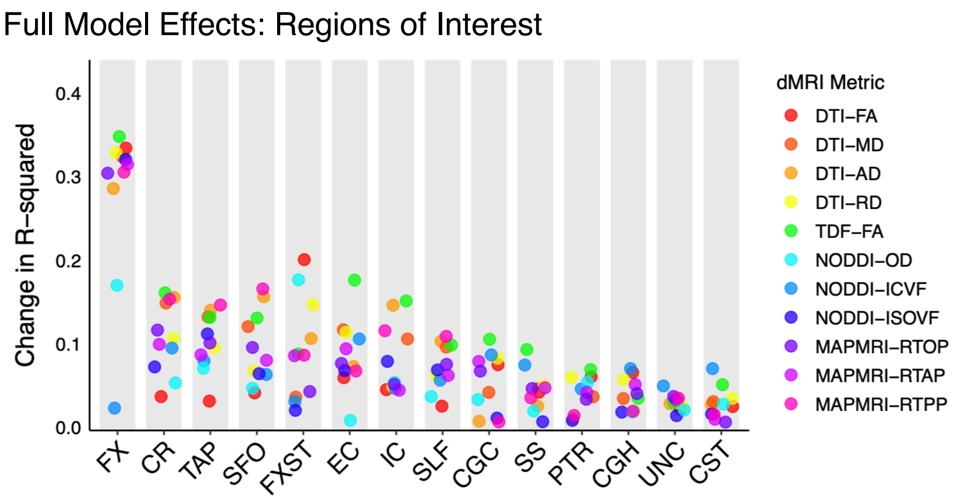

Figure S23. Total variance explained by the full model including age, sex, their interaction, and nuisance covariates when modeling age with fractional polynomials. Filled circles indicate the full model was significant (*p*<0.05) after FDR-correction for the number of regions. Regions are ordered by the number of significant metrics, followed by the mean effect size. CGC = cingulum (cingulate), CGH = cingulum (hippocampal), CR = *corona radiata*, CST = corticospinal tract, EC = external capsule, FX = fornix (body), FXST = fornix (*crus*) / *stria terminalis*, IC = internal capsule, PTR = posterior thalamic radiation, SFO = superior fronto-occpital fasciculus, SLF = superior longitudinal fasiculus, SS = *sagittal stratum*, TAP = tapetum, UNC = uncinate fasciculus

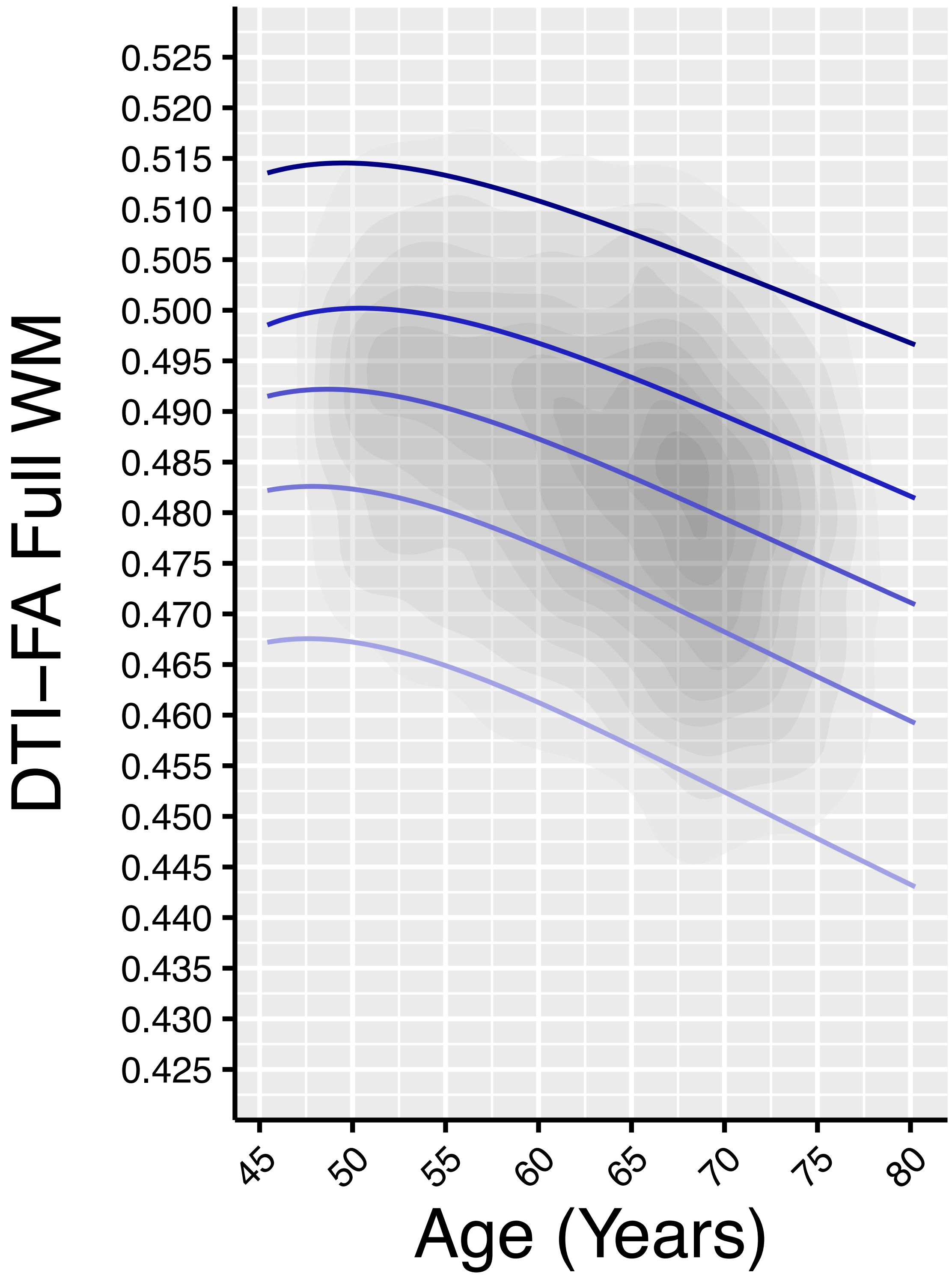

Figure S24. Full size normative centile reference curves calculated for the full white matter for DTI-FA in males. Solid colored lines, ordered from lightest to darkest, indicate the following centiles: 5th, 25th, 50th, 75th, 95th. Gray overlay reflects kernel density (darker=greater degree of data point overlap). WM = white matter.

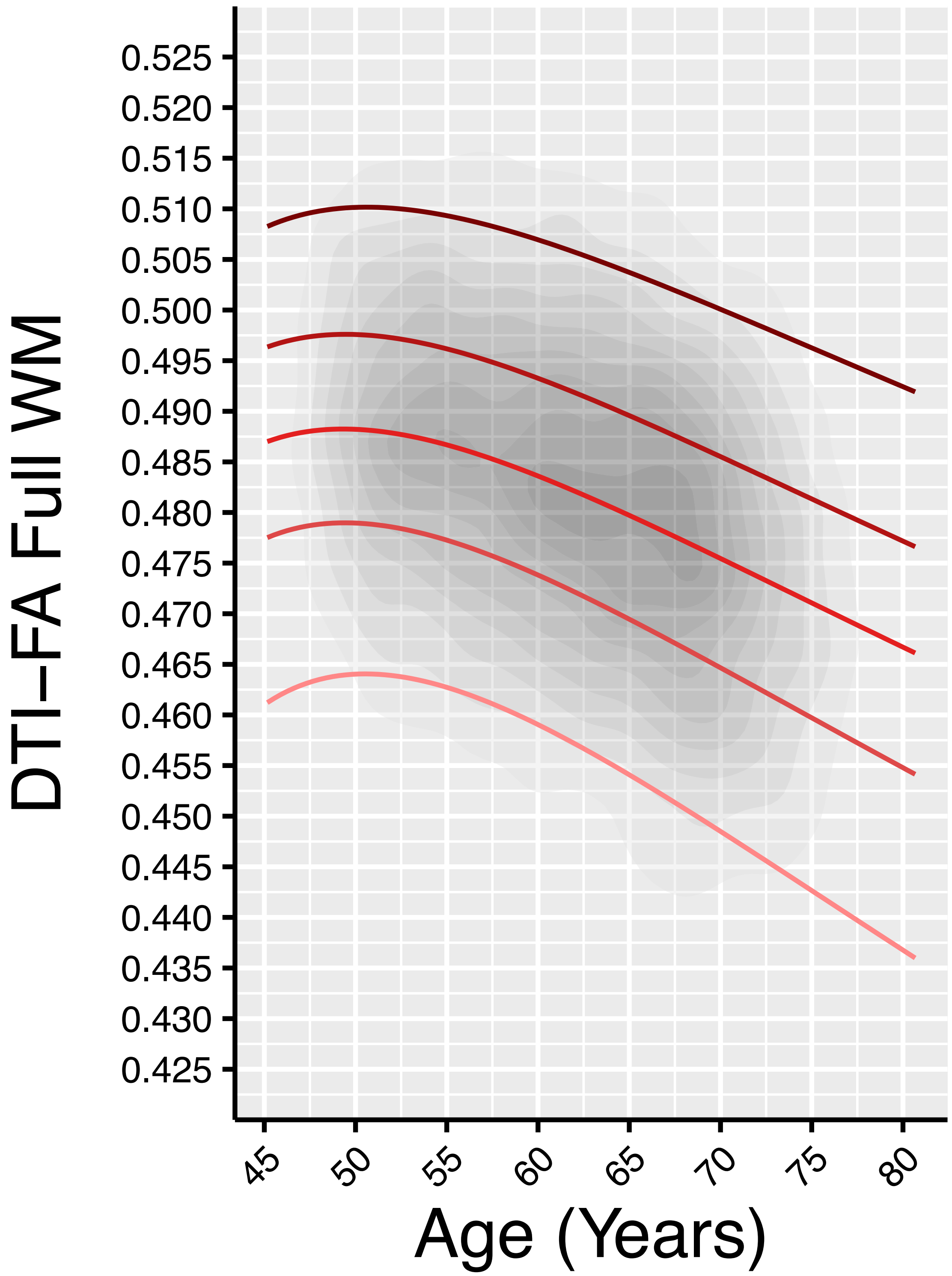

Figure S25. Full size normative centile reference curves calculated for the full white matter for DTI-FA in females. Solid colored lines, ordered from lightest to darkest, indicate the following centiles: 5th, 25th, 50th, 75th, 95th. Gray overlay reflects kernel density (darker=greater degree of data point overlap). WM = white matter.

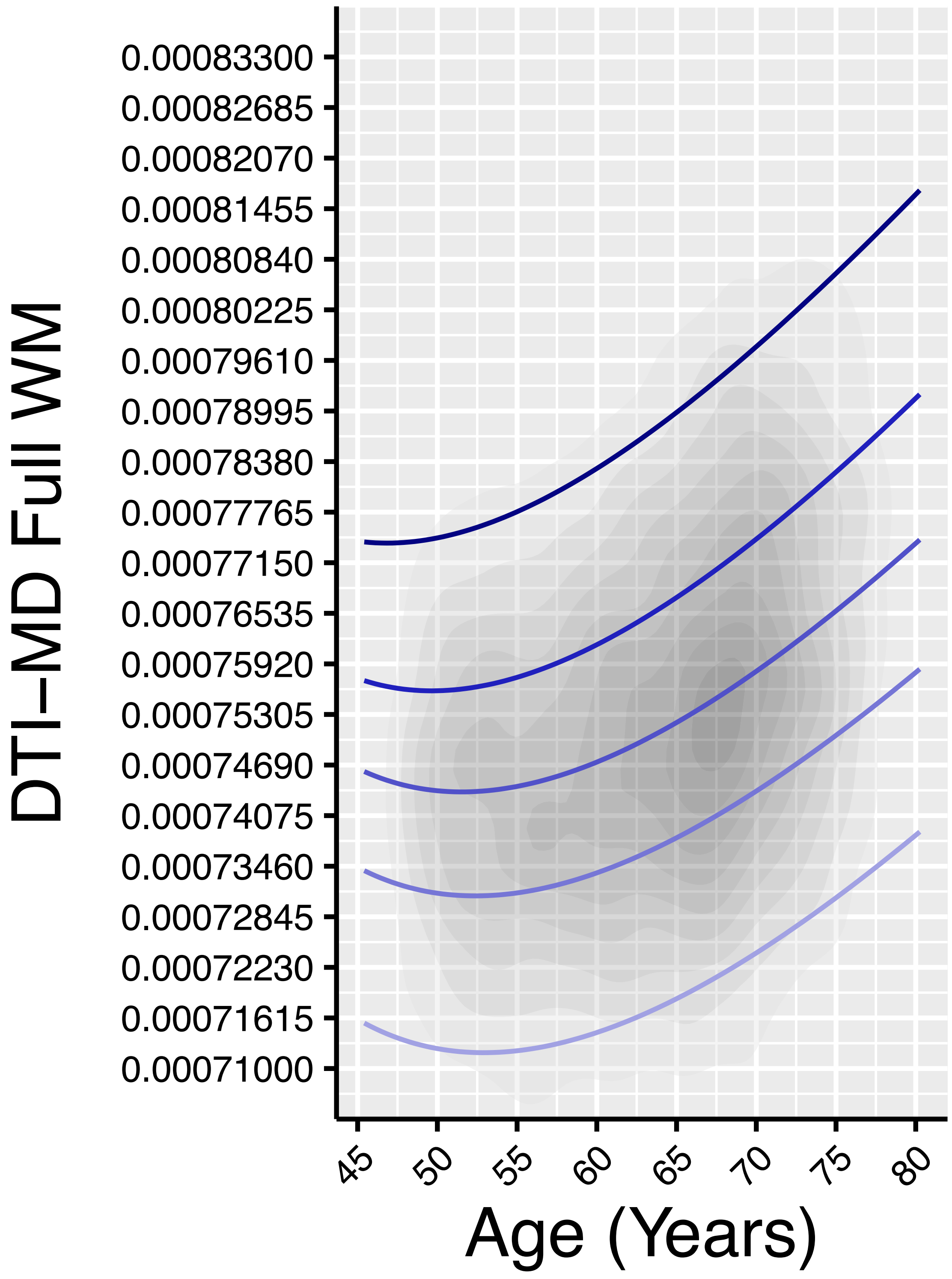

Figure S26. Full size normative centile reference curves calculated for the full white matter for DTI-MD in males. Solid colored lines, ordered from lightest to darkest, indicate the following centiles: 5th, 25th, 50th, 75th, 95th. Gray overlay reflects kernel density (darker=greater degree of data point overlap). WM = white matter.

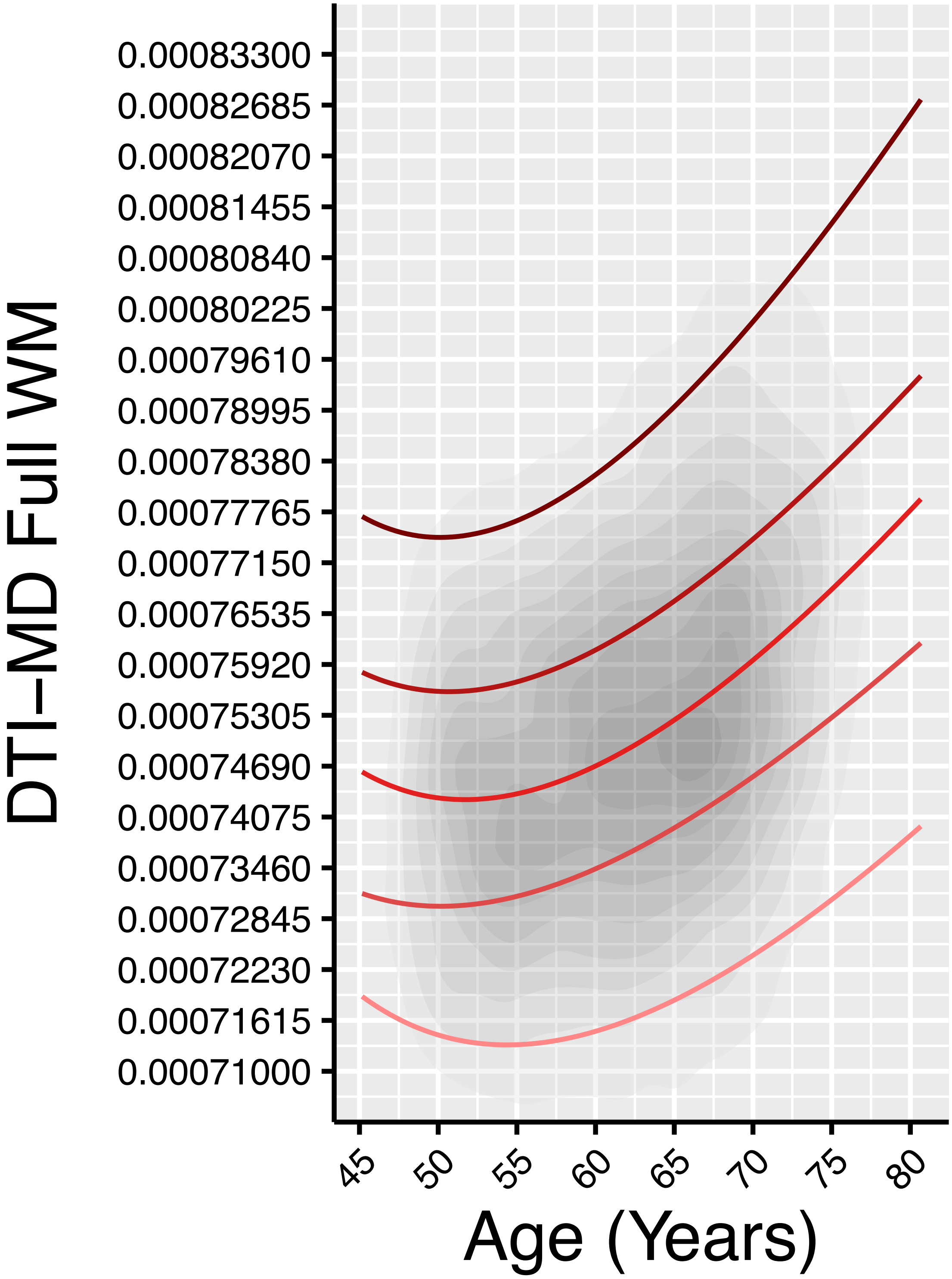

Figure S27. Full size normative centile reference curves calculated for the full white matter for DTI-MD in females. Solid colored lines, ordered from lightest to darkest, indicate the following centiles: 5th, 25th, 50th, 75th, 95th. Gray overlay reflects kernel density (darker=greater degree of data point overlap). WM = white matter.

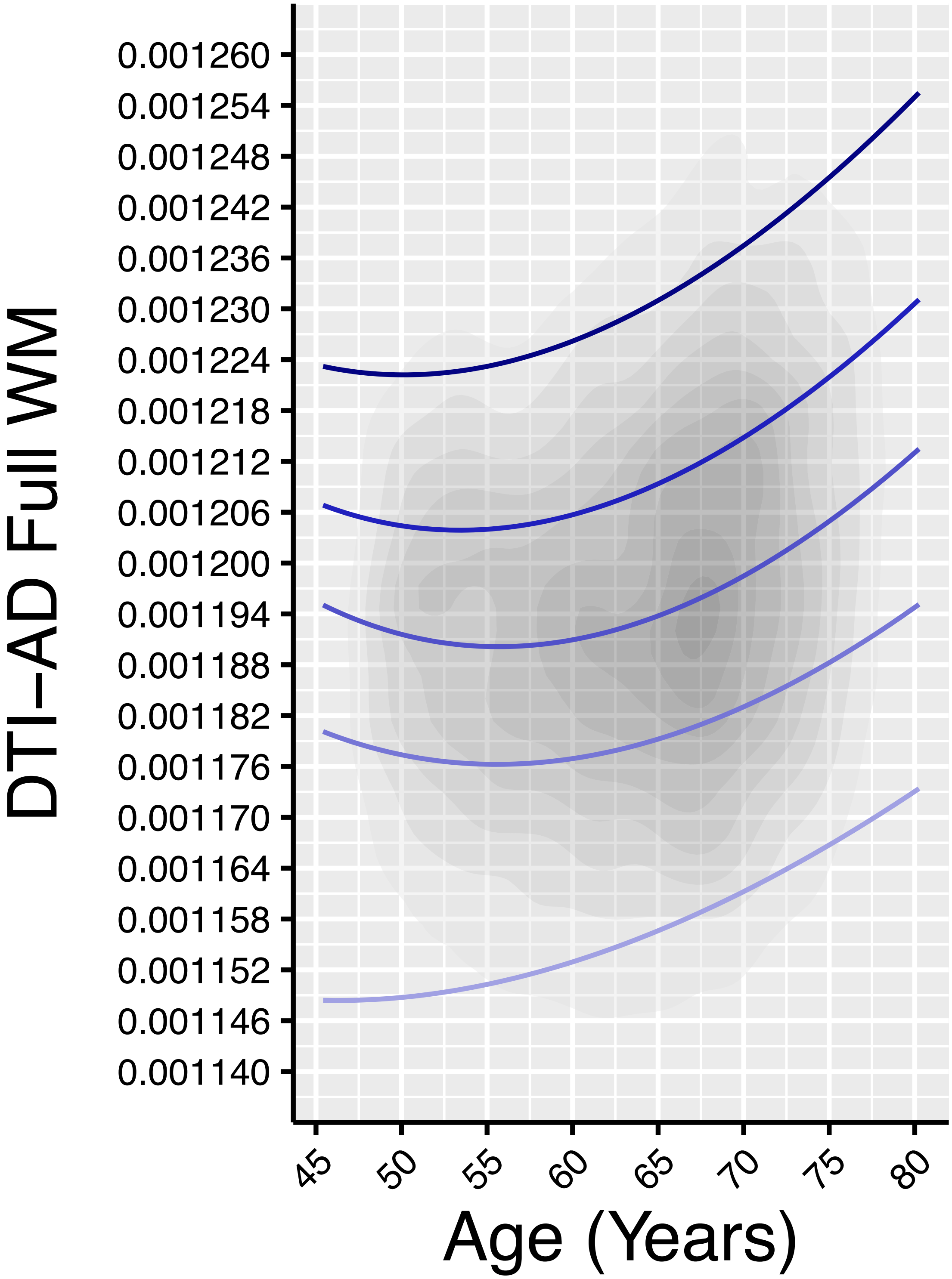

Figure S28. Full size normative centile reference curves calculated for the full white matter for DTI-AD in males. Solid colored lines, ordered from lightest to darkest, indicate the following centiles: 5th, 25th, 50th, 75th, 95th. Gray overlay reflects kernel density (darker=greater degree of data point overlap). WM = white matter.

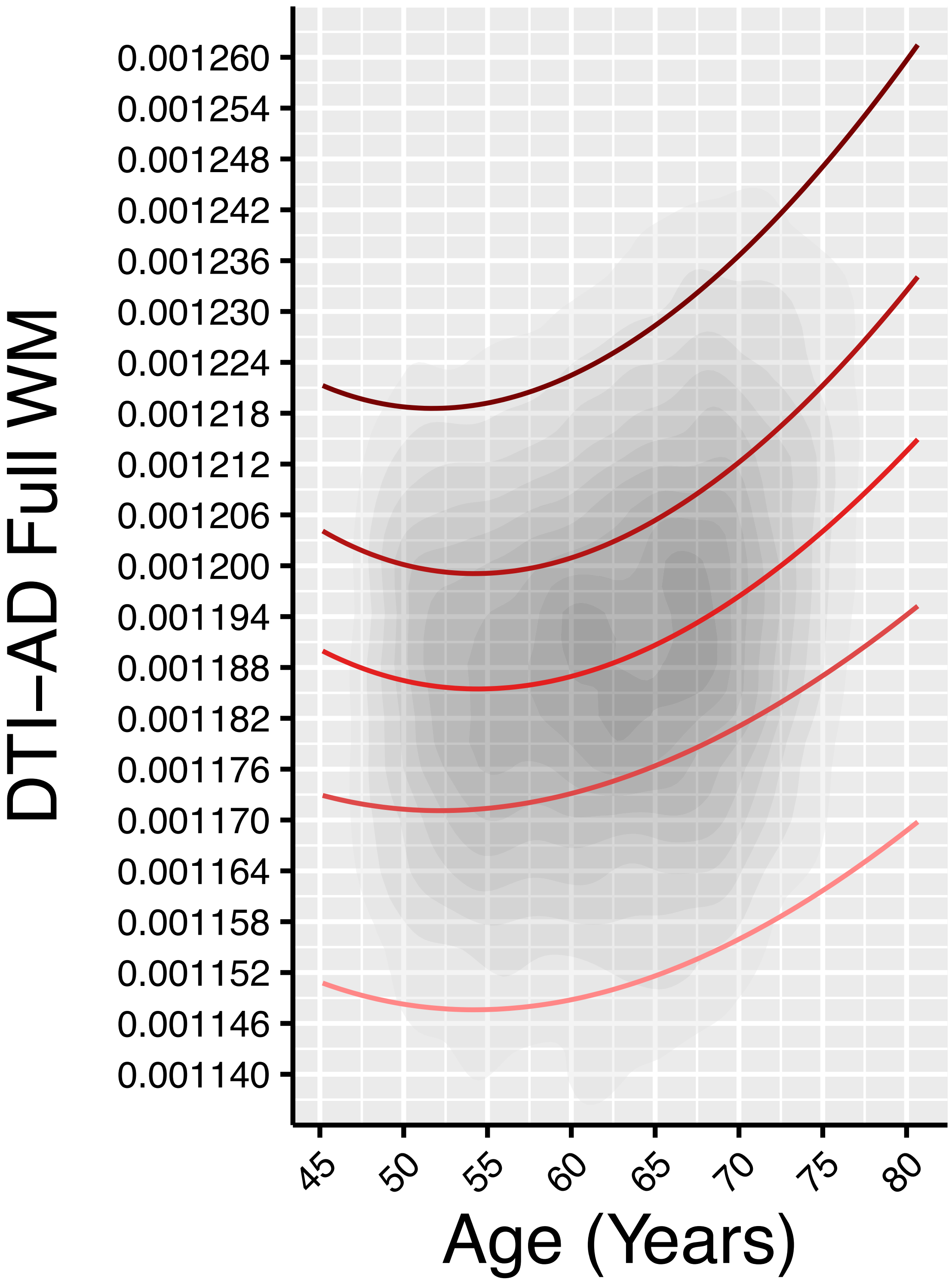

Figure S29. Full size normative centile reference curves calculated for the full white matter for DTI-AD in females. Solid colored lines, ordered from lightest to darkest, indicate the following centiles: 5th, 25th, 50th, 75th, 95th. Gray overlay reflects kernel density (darker=greater degree of data point overlap). WM = white matter.

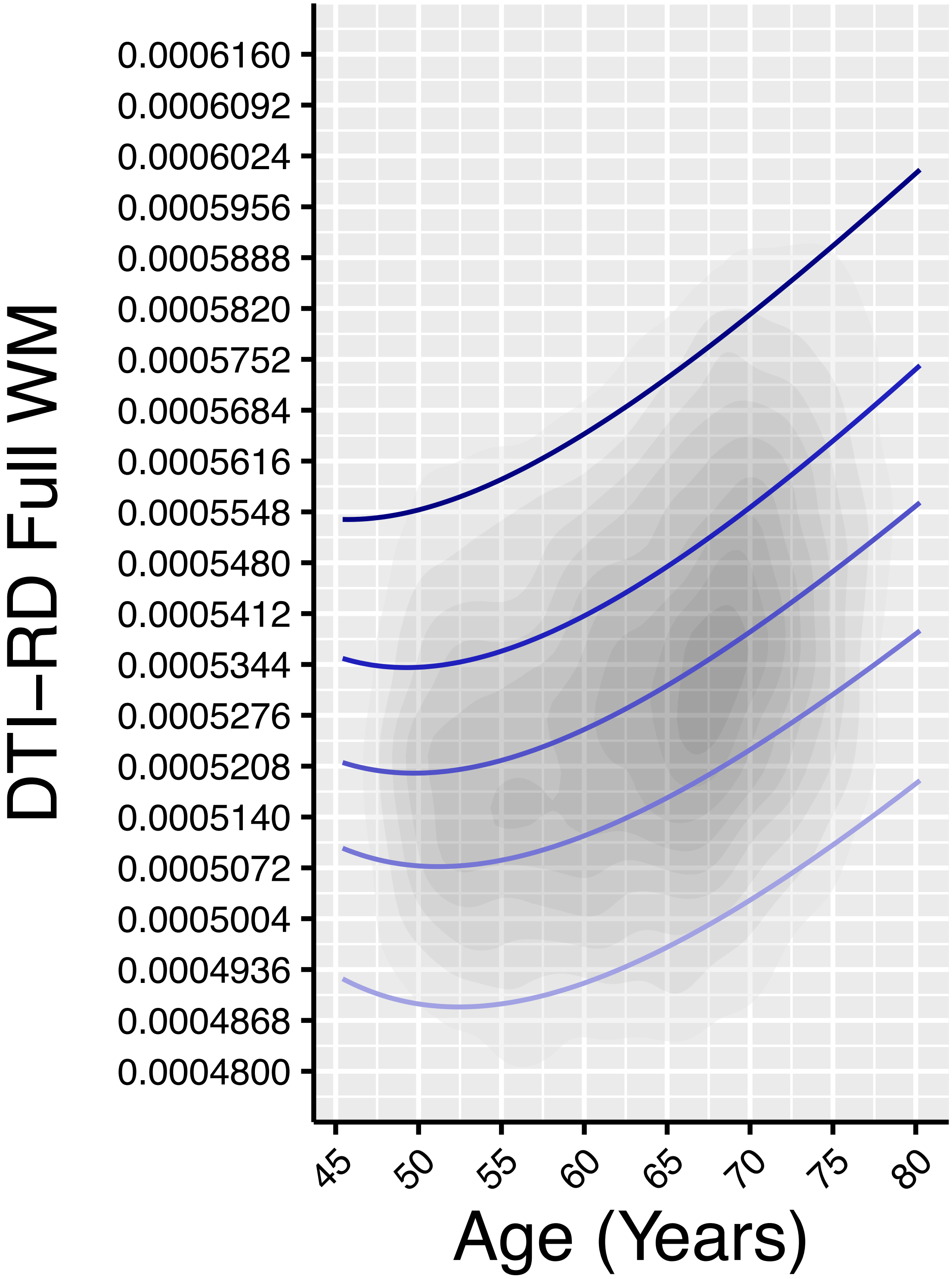

Figure S30. Full size normative centile reference curves calculated for the full white matter for DTI-RD in males. Solid colored lines, ordered from lightest to darkest, indicate the following centiles: 5th, 25th, 50th, 75th, 95th. Gray overlay reflects kernel density (darker=greater degree of data point overlap). WM = white matter.

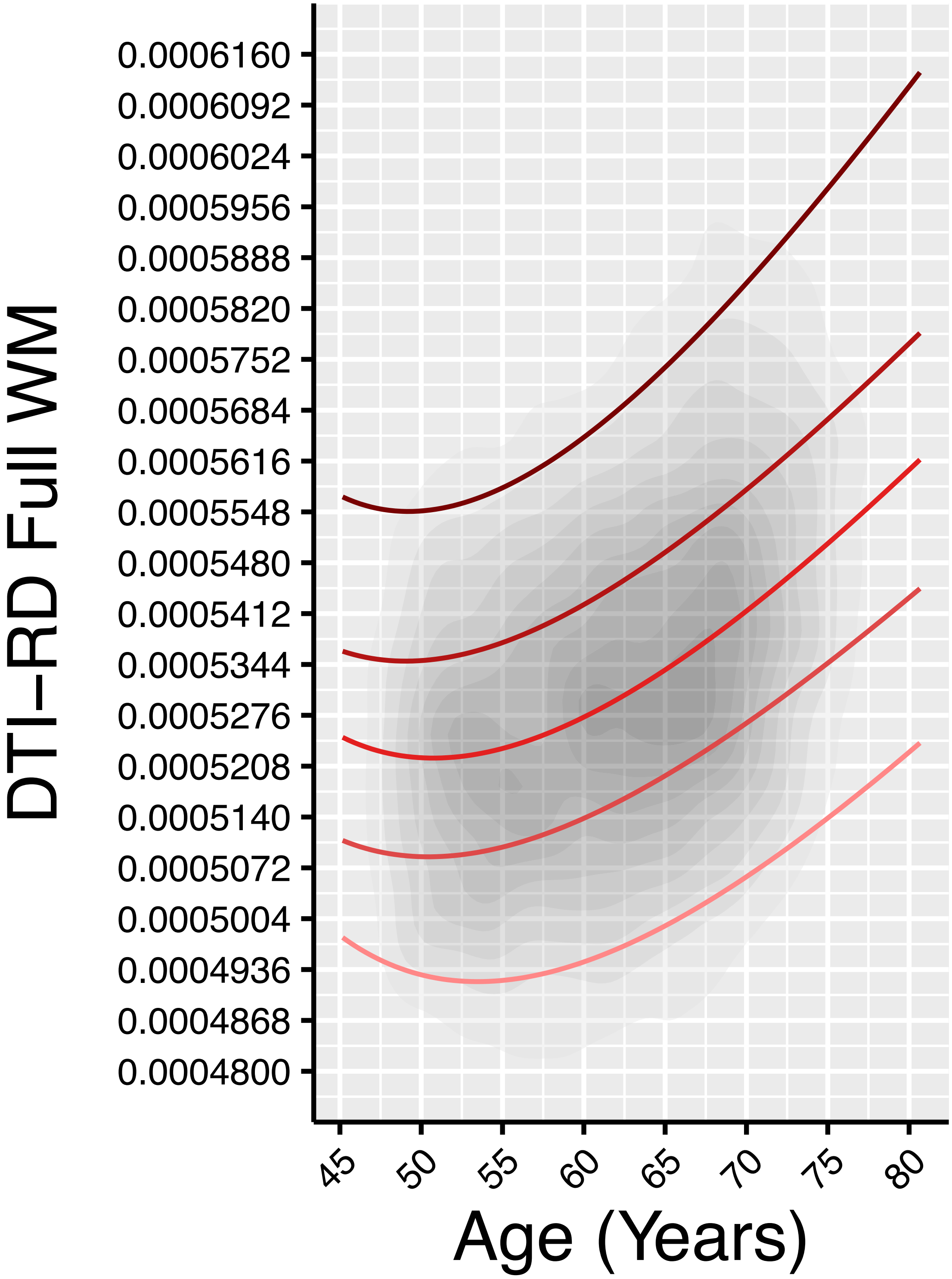

Figure S31. Full size normative centile reference curves calculated for the full white matter for DTI-RD in females. Solid colored lines, ordered from lightest to darkest, indicate the following centiles: 5th, 25th, 50th, 75th, 95th. Gray overlay reflects kernel density (darker=greater degree of data point overlap). WM = white matter.

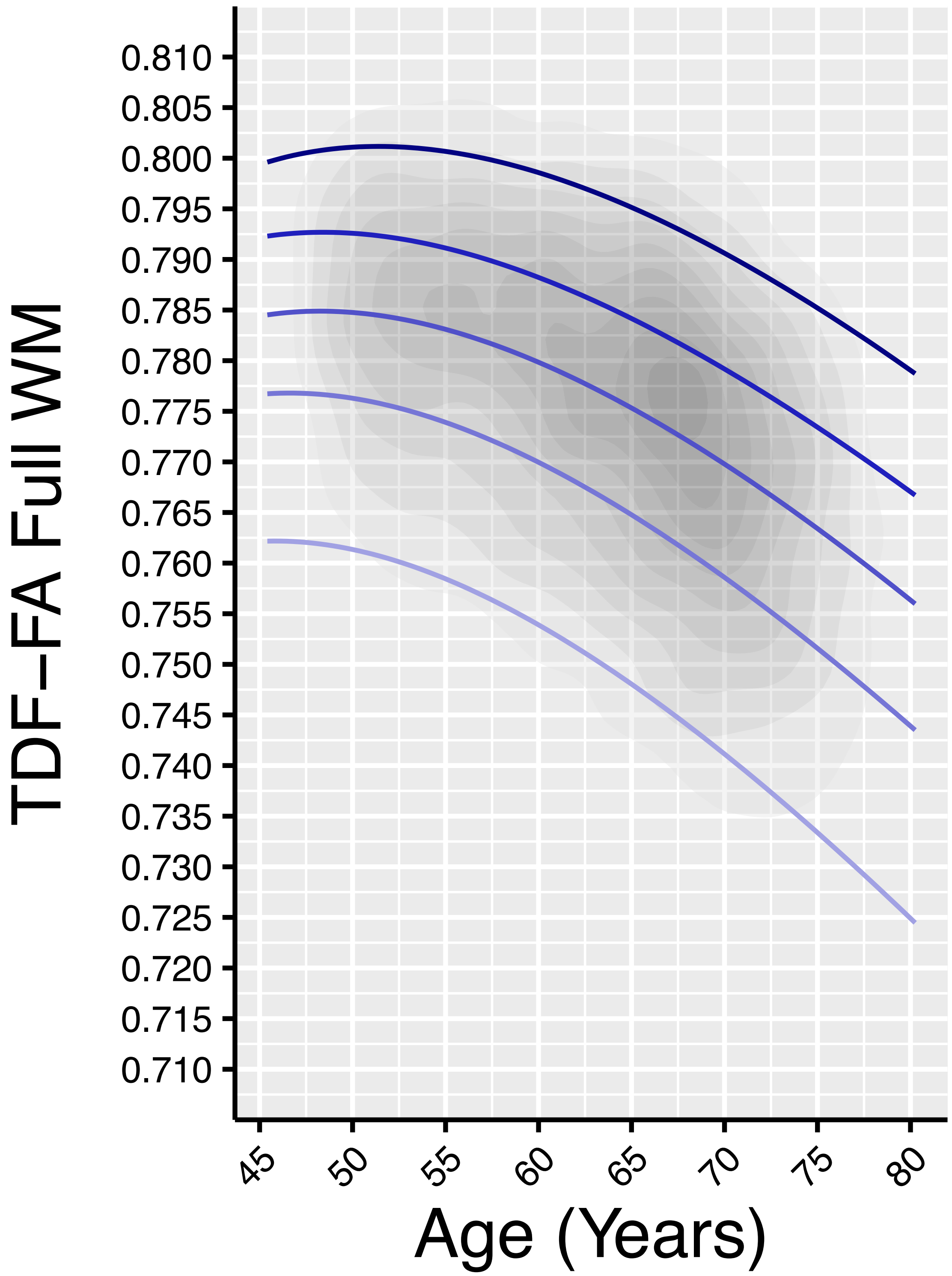

Figure S32. Full size normative centile reference curves calculated for the full white matter for TDF-FA in males. Solid colored lines, ordered from lightest to darkest, indicate the following centiles: 5th, 25th, 50th, 75th, 95th. Gray overlay reflects kernel density (darker=greater degree of data point overlap). WM = white matter.

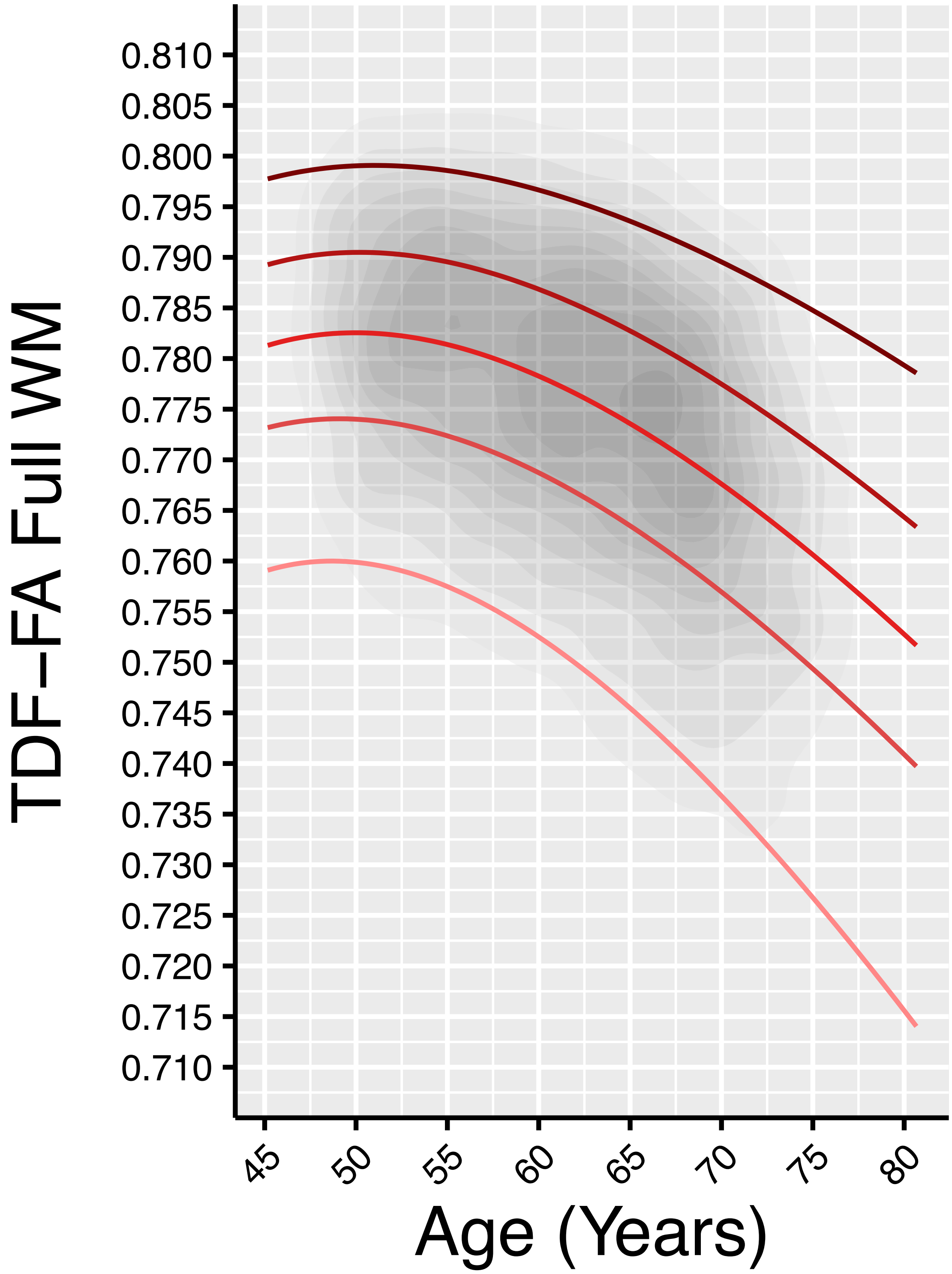

Figure S33. Full size normative centile reference curves calculated for the full white matter for TDF-FA in females. Solid colored lines, ordered from lightest to darkest, indicate the following centiles: 5th, 25th, 50th, 75th, 95th. Gray overlay reflects kernel density (darker=greater degree of data point overlap). WM = white matter.

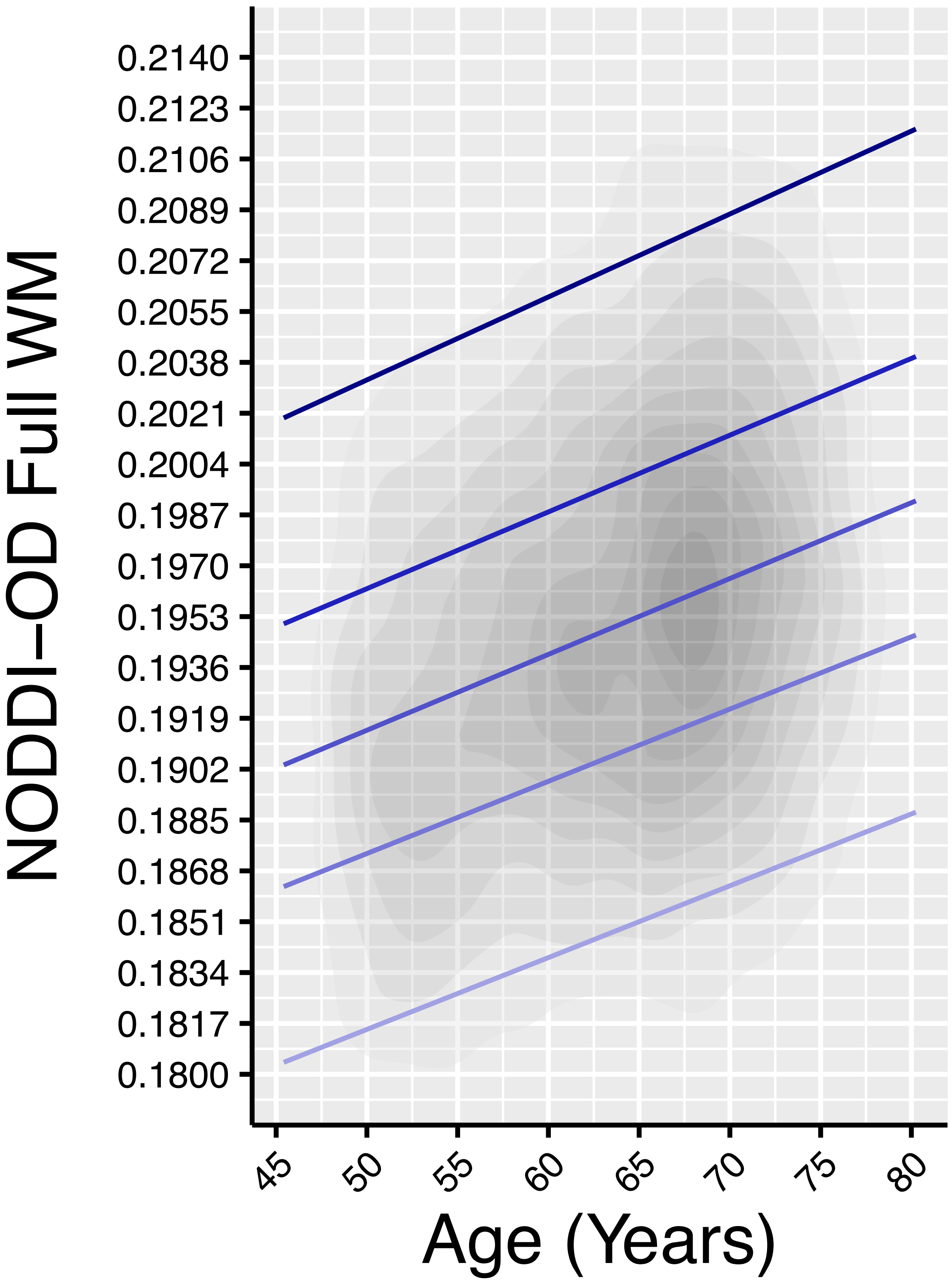

Figure S34. Full size normative centile reference curves calculated for the full white matter for NODDI-OD in males. Solid colored lines, ordered from lightest to darkest, indicate the following centiles: 5th, 25th, 50th, 75th, 95th. Gray overlay reflects kernel density (darker=greater degree of data point overlap). WM = white matter.

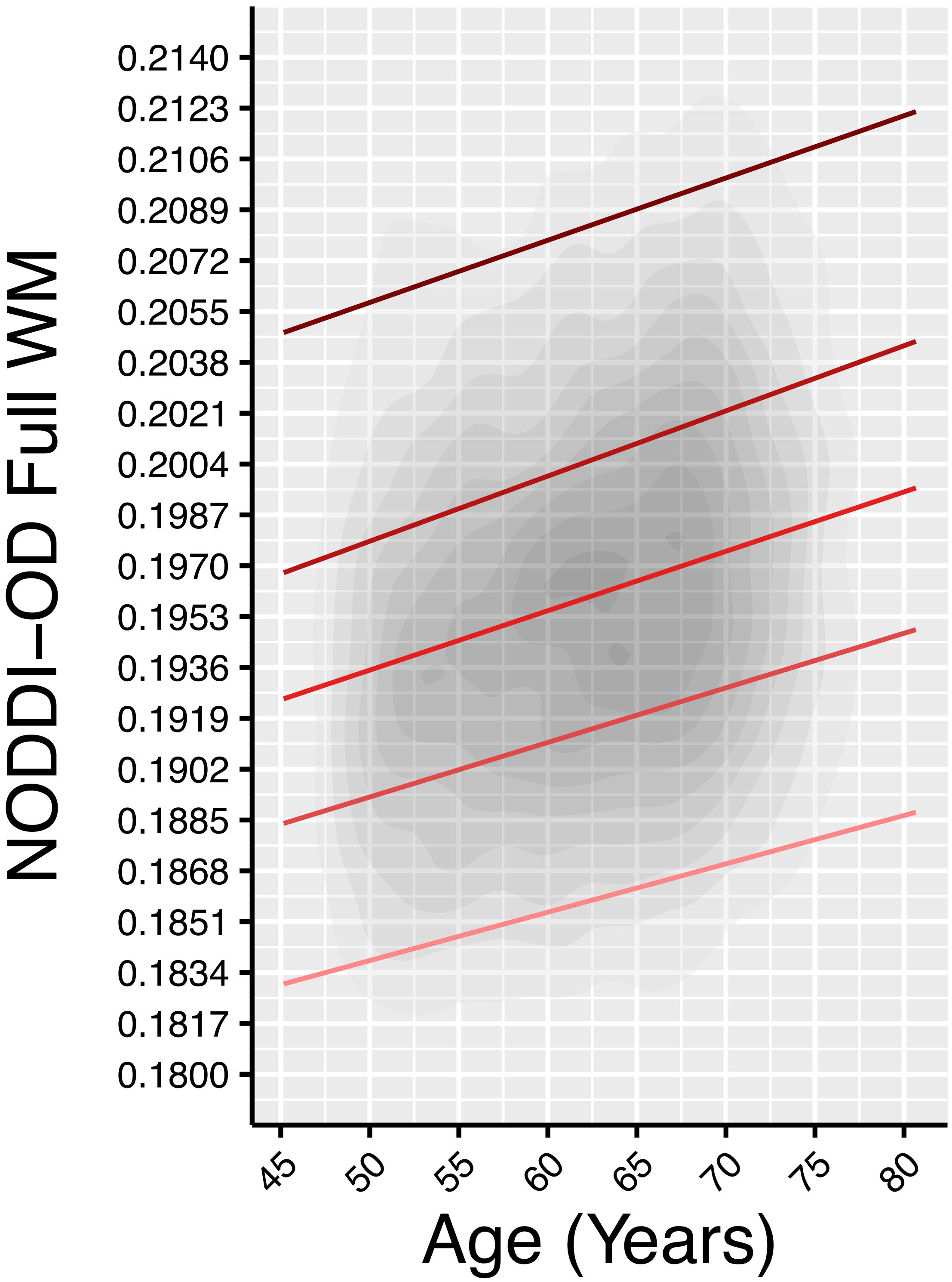

Figure S35. Full size normative centile reference curves calculated for the full white matter for NODDI-OD in females. Solid colored lines, ordered from lightest to darkest, indicate the following centiles: 5th, 25th, 50th, 75th, 95th. Gray overlay reflects kernel density (darker=greater degree of data point overlap). WM = white matter.

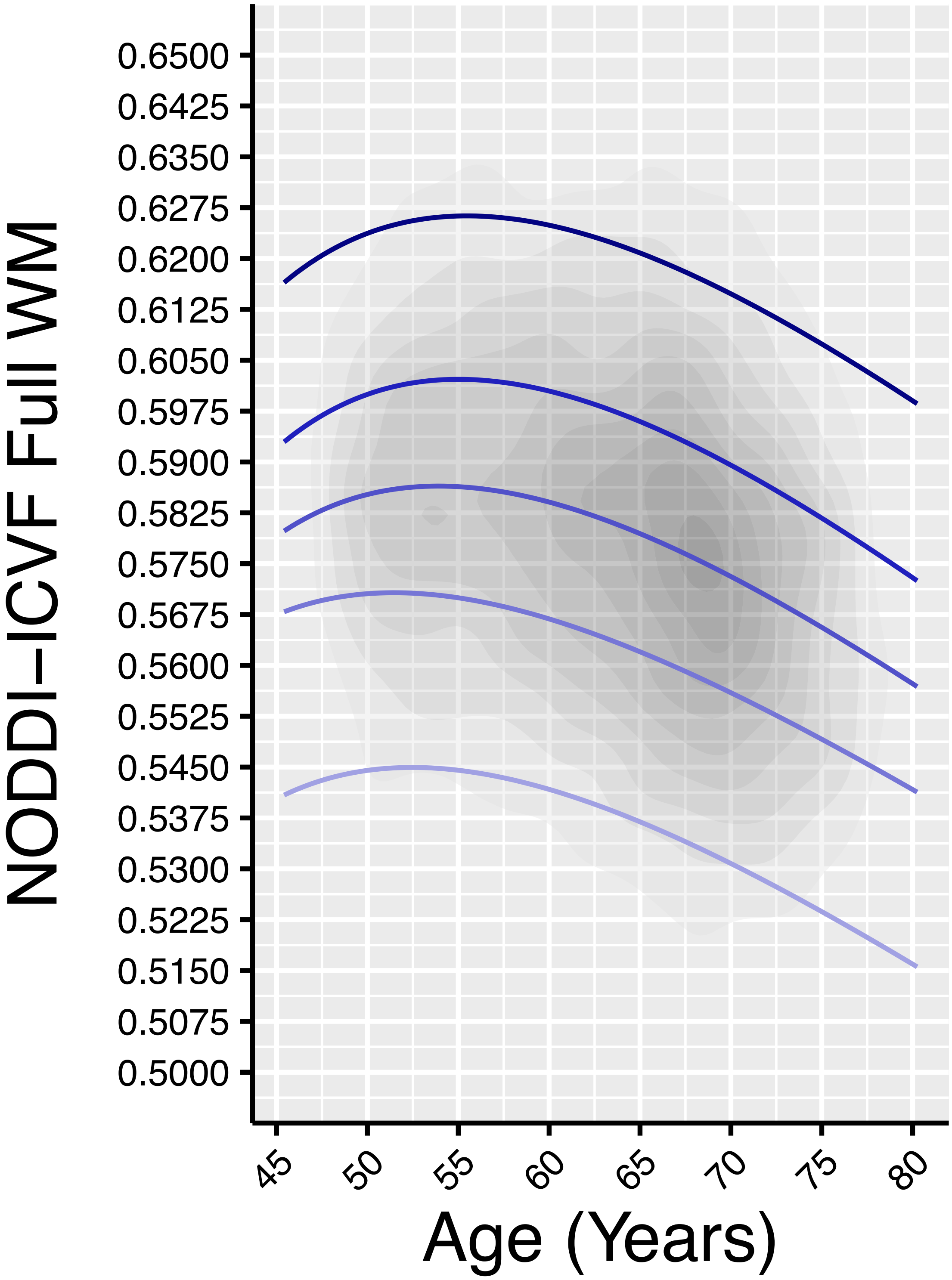

Figure S36. Full size normative centile reference curves calculated for the full white matter for NODDI-ICVF in males. Solid colored lines, ordered from lightest to darkest, indicate the following centiles: 5th, 25th, 50th, 75th, 95th. Gray overlay reflects kernel density (darker=greater degree of data point overlap). WM = white matter.

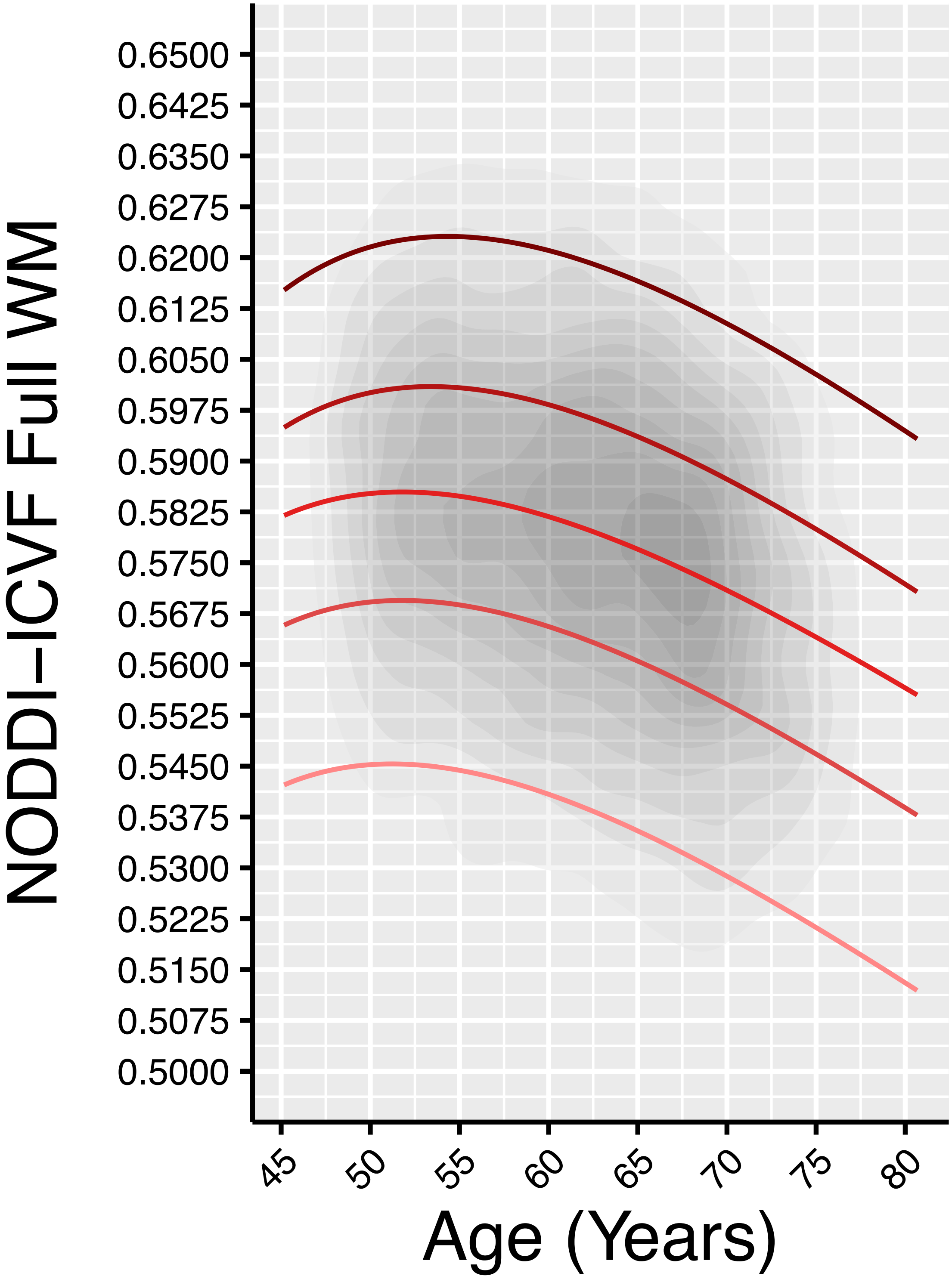

Figure S37. Full size normative centile reference curves calculated for the full white matter for NODDI-ICVF in females. Solid colored lines, ordered from lightest to darkest, indicate the following centiles: 5th, 25th, 50th, 75th, 95th. Gray overlay reflects kernel density (darker=greater degree of data point overlap). WM = white matter.

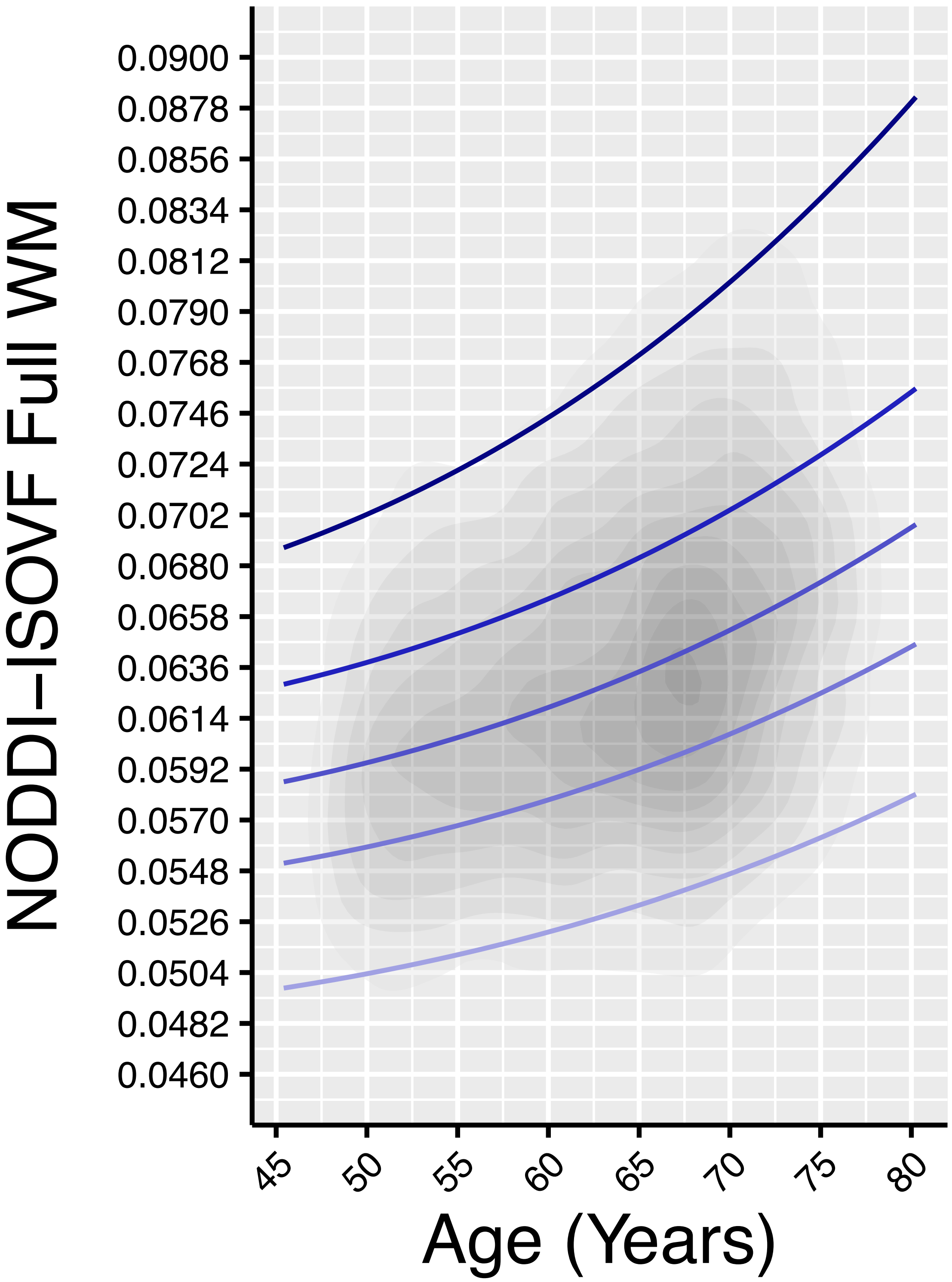

Figure S38. Full size normative centile reference curves calculated for the full white matter for NODDI-ISOVF in males. Solid colored lines, ordered from lightest to darkest, indicate the following centiles: 5th, 25th, 50th, 75th, 95th. Gray overlay reflects kernel density (darker=greater degree of data point overlap). WM = white matter.

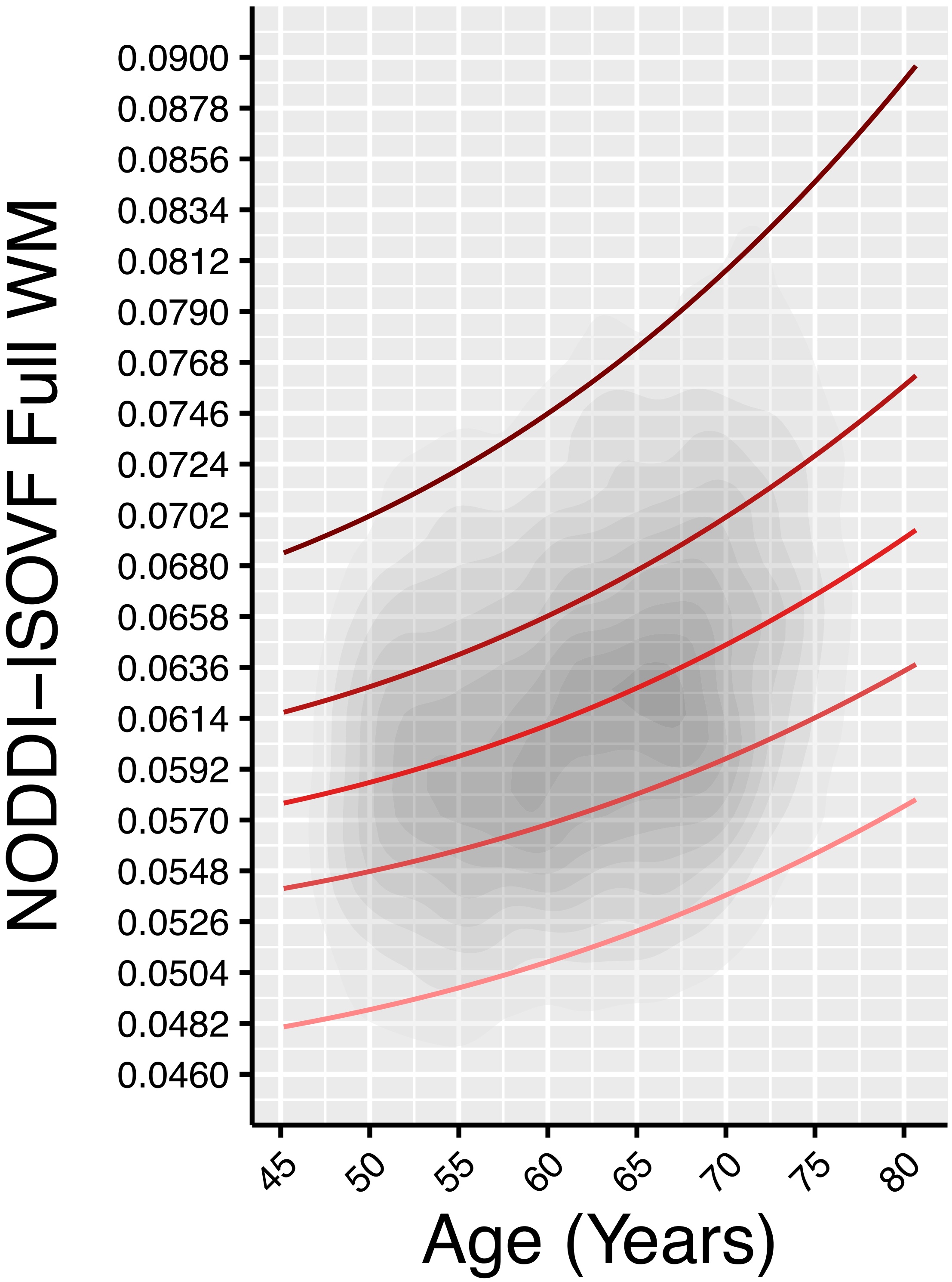

Figure S39. Full size normative centile reference curves calculated for the full white matter for NODDI-ISOVF in females. Solid colored lines, ordered from lightest to darkest, indicate the following centiles: 5th, 25th, 50th, 75th, 95th. Gray overlay reflects kernel density (darker=greater degree of data point overlap). WM = white matter.

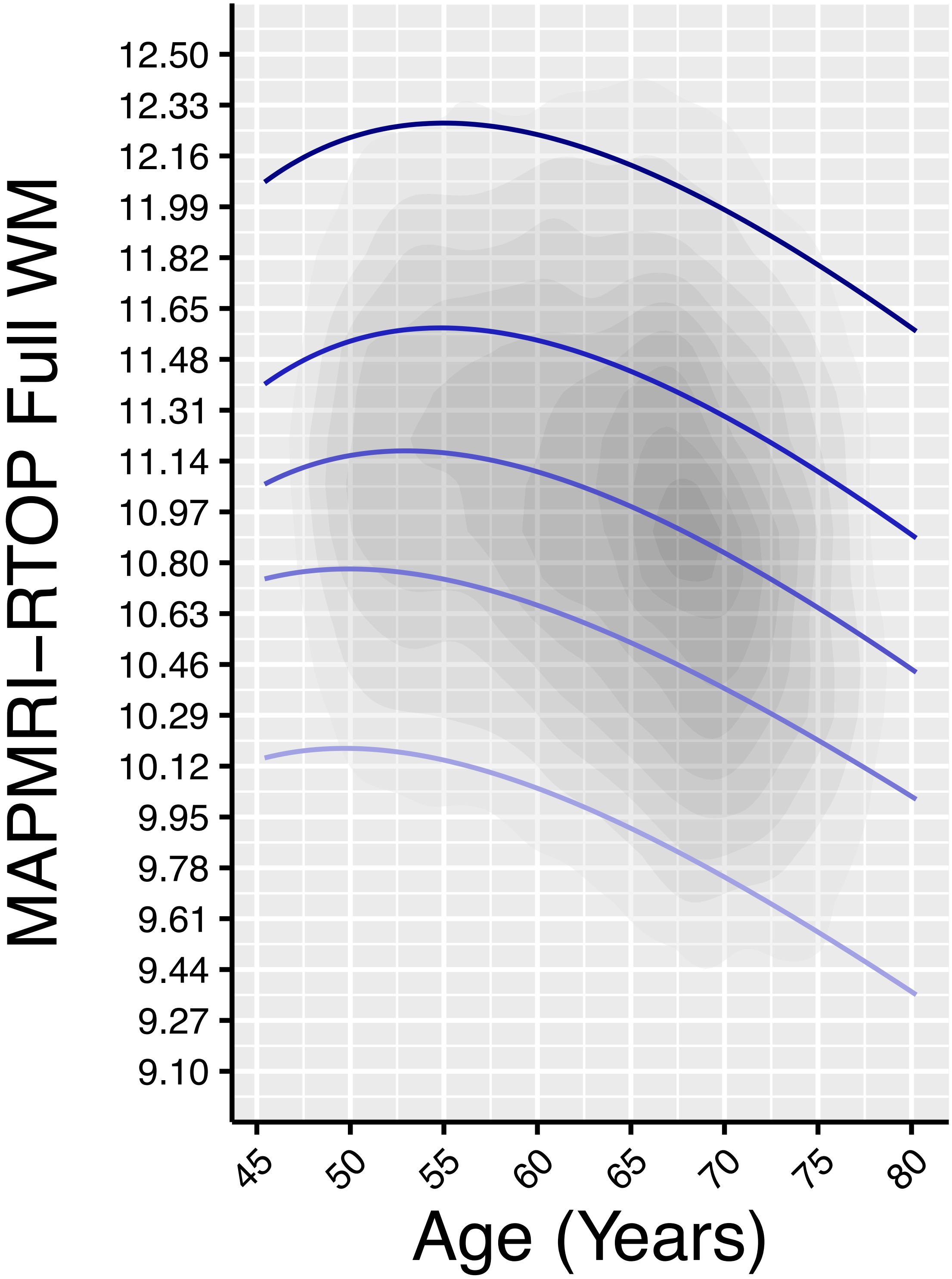

Figure S40. Full size normative centile reference curves calculated for the full white matter for MAPMRI-RTOP in males. Solid colored lines, ordered from lightest to darkest, indicate the following centiles: 5th, 25th, 50th, 75th, 95th. Gray overlay reflects kernel density (darker=greater degree of data point overlap). WM = white matter.

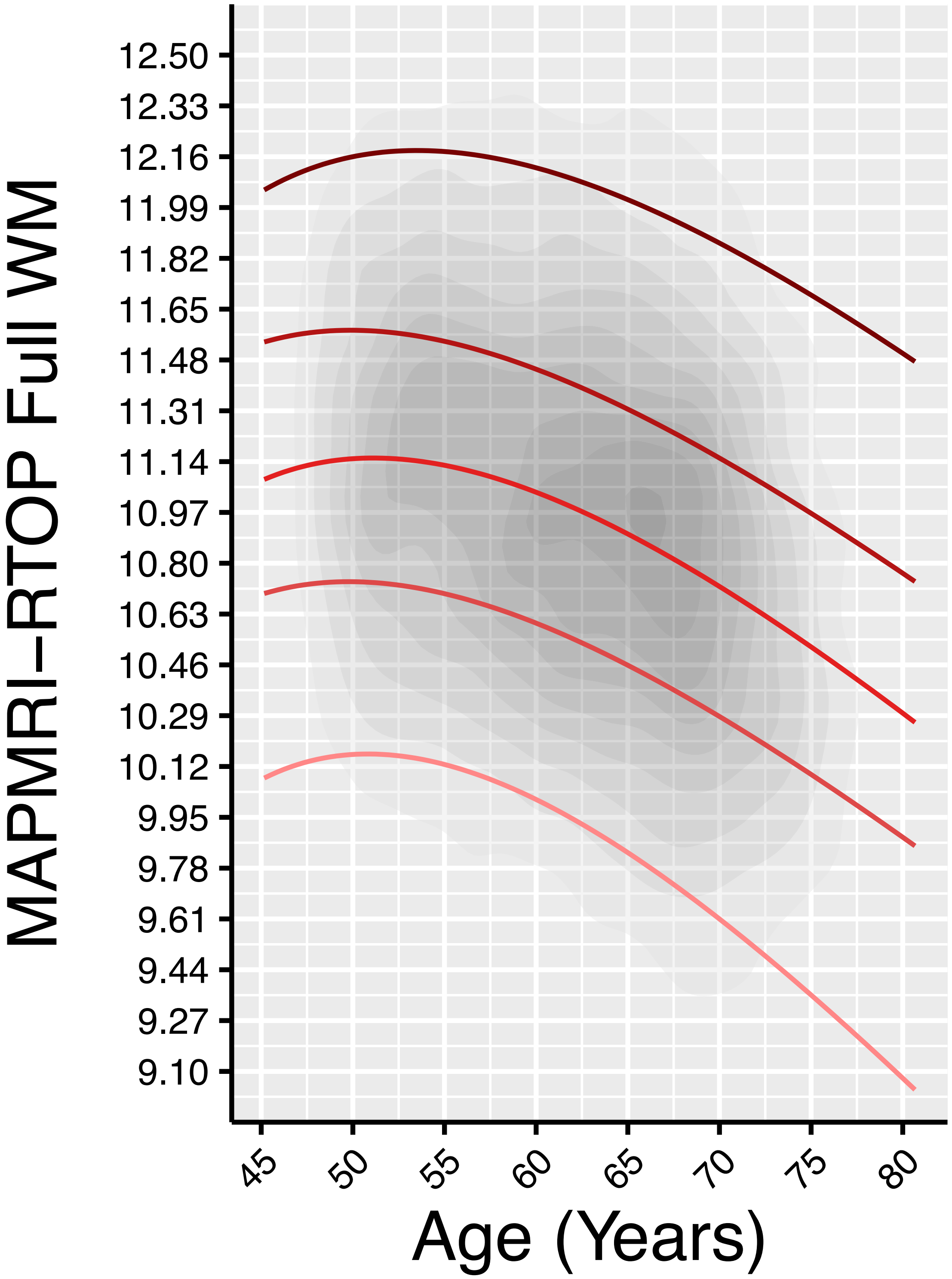

Figure S41. Full size normative centile reference curves calculated for the full white matter for MAPMRI-RTOP in females. Solid colored lines, ordered from lightest to darkest, indicate the following centiles: 5th, 25th, 50th, 75th, 95th. Gray overlay reflects kernel density (darker=greater degree of data point overlap). WM = white matter.

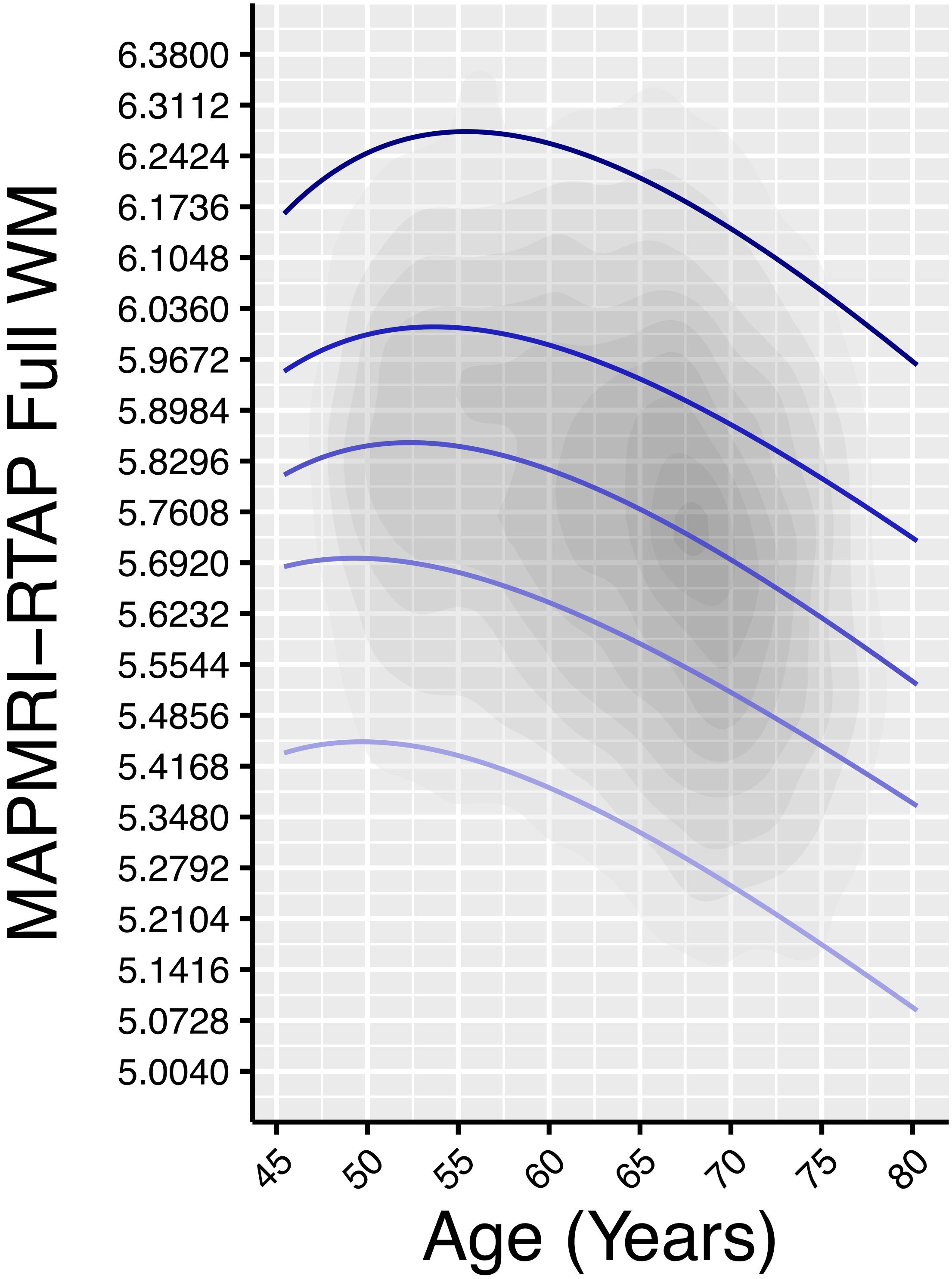

Figure S42. Full size normative centile reference curves calculated for the full white matter for MAPMRI-RTAP in males. Solid colored lines, ordered from lightest to darkest, indicate the following centiles: 5th, 25th, 50th, 75th, 95th. Gray overlay reflects kernel density (darker=greater degree of data point overlap). WM = white matter.

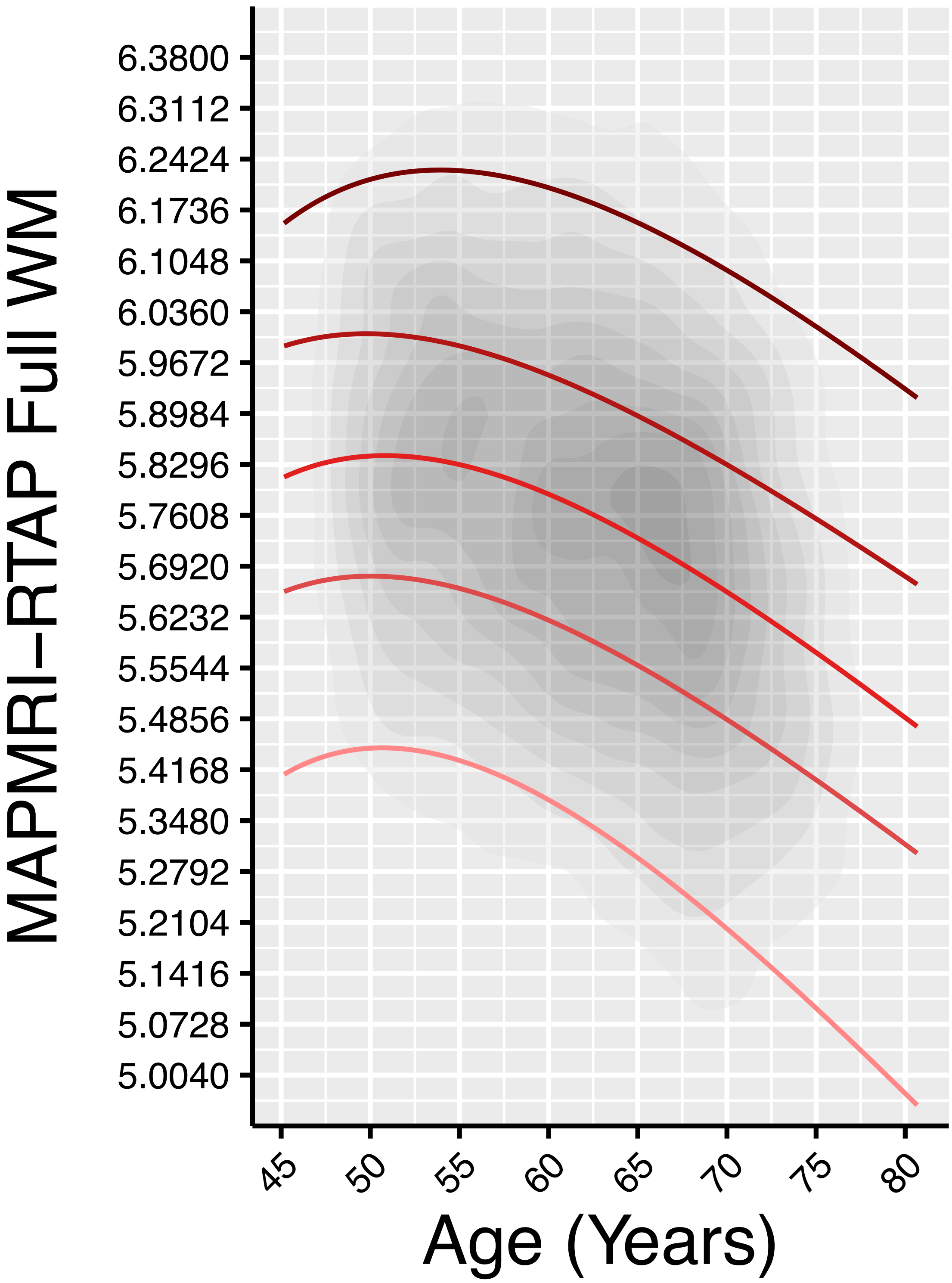

Figure S43. Full size normative centile reference curves calculated for the full white matter for MAPMRI-RTAP in females. Solid colored lines, ordered from lightest to darkest, indicate the following centiles: 5th, 25th, 50th, 75th, 95th. Gray overlay reflects kernel density (darker=greater degree of data point overlap). WM = white matter.

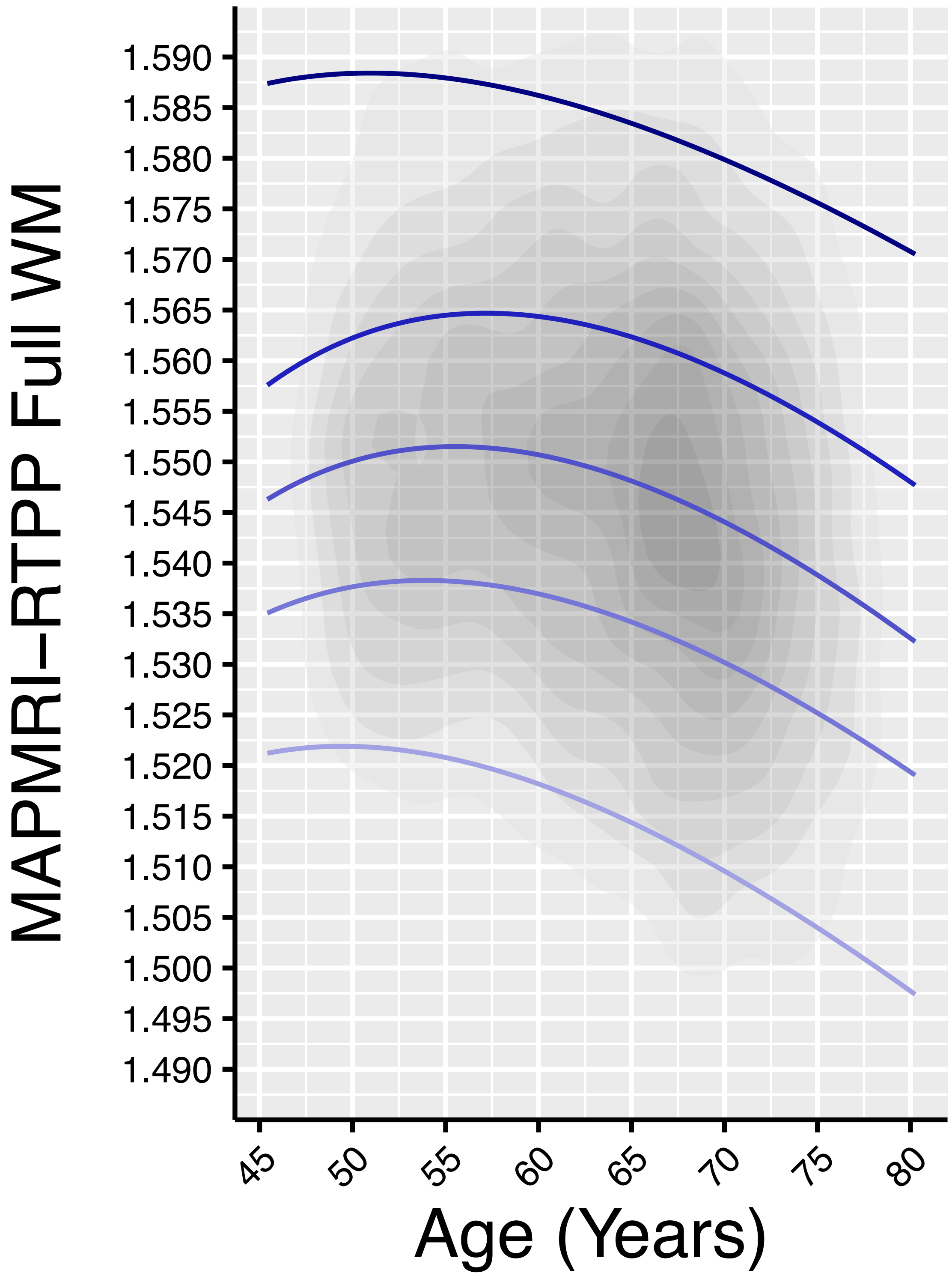

Figure S44. Full size normative centile reference curves calculated for the full white matter for MAPMRI-RTPP in males. Solid colored lines, ordered from lightest to darkest, indicate the following centiles: 5th, 25th, 50th, 75th, 95th. Gray overlay reflects kernel density (darker=greater degree of data point overlap). WM = white matter.

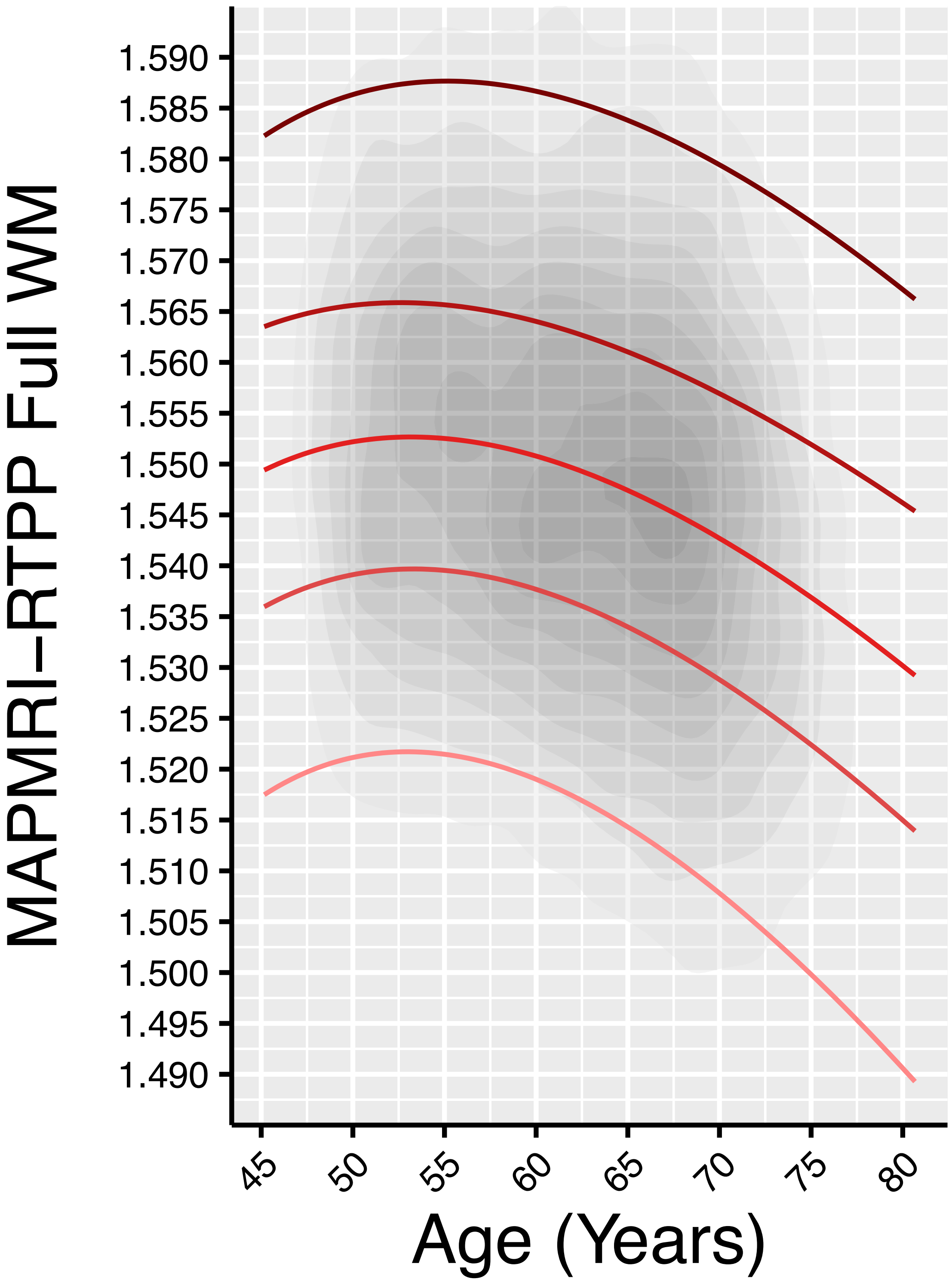

Figure S45. Full size normative centile reference curves calculated for the full white matter for MAPMRI-RTPP in females. Solid colored lines, ordered from lightest to darkest, indicate the following centiles: 5th, 25th, 50th, 75th, 95th. Gray overlay reflects kernel density (darker=greater degree of data point overlap). WM = white matter.

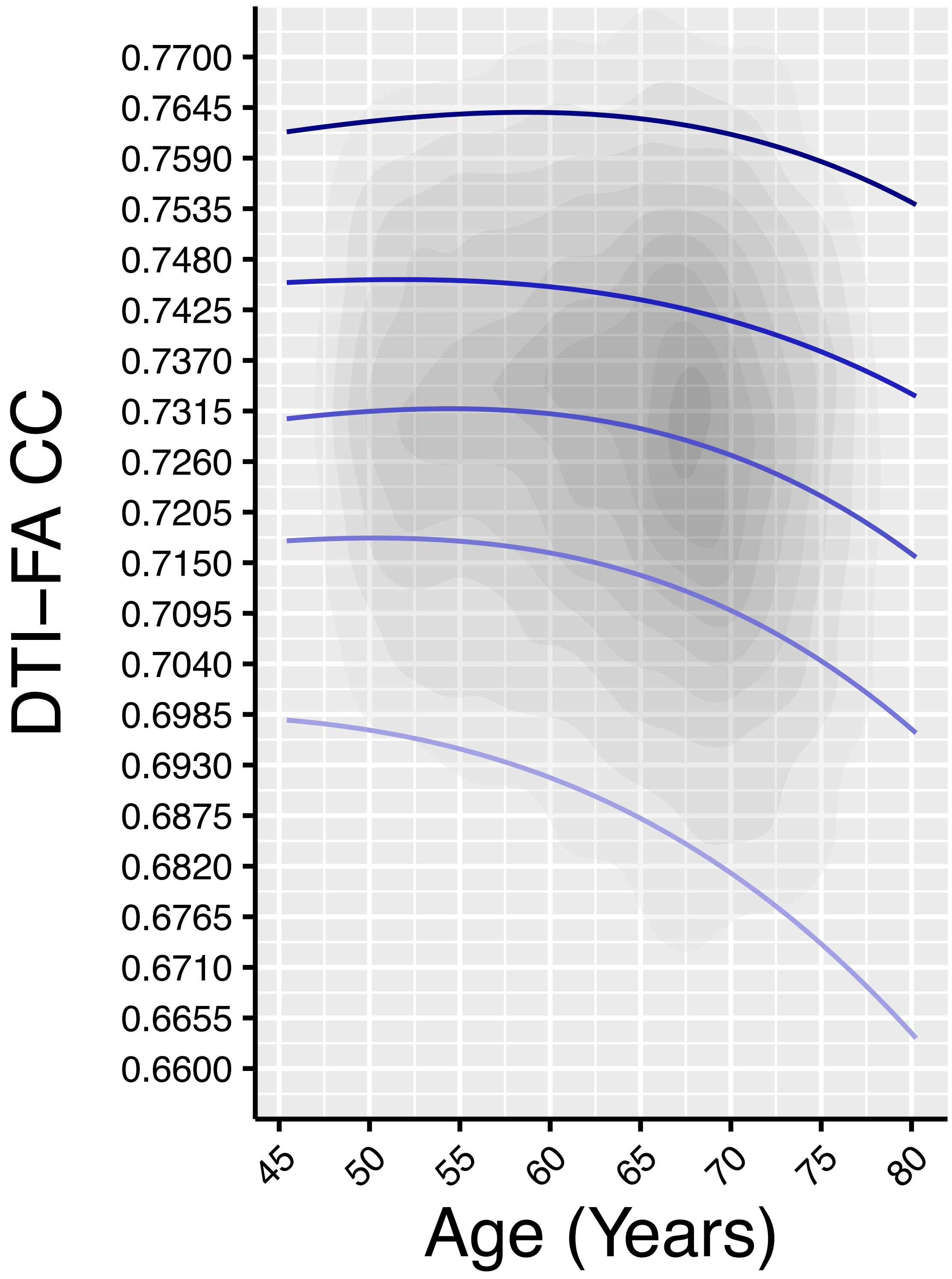

Figure S46. Full size normative centile reference curves calculated for the corpus callosum for DTI-FA in males. Solid colored lines, ordered from lightest to darkest, indicate the following centiles: 5th, 25th, 50th, 75th, 95th. Gray overlay reflects kernel density (darker=greater degree of data point overlap). CC = corpus callosum.

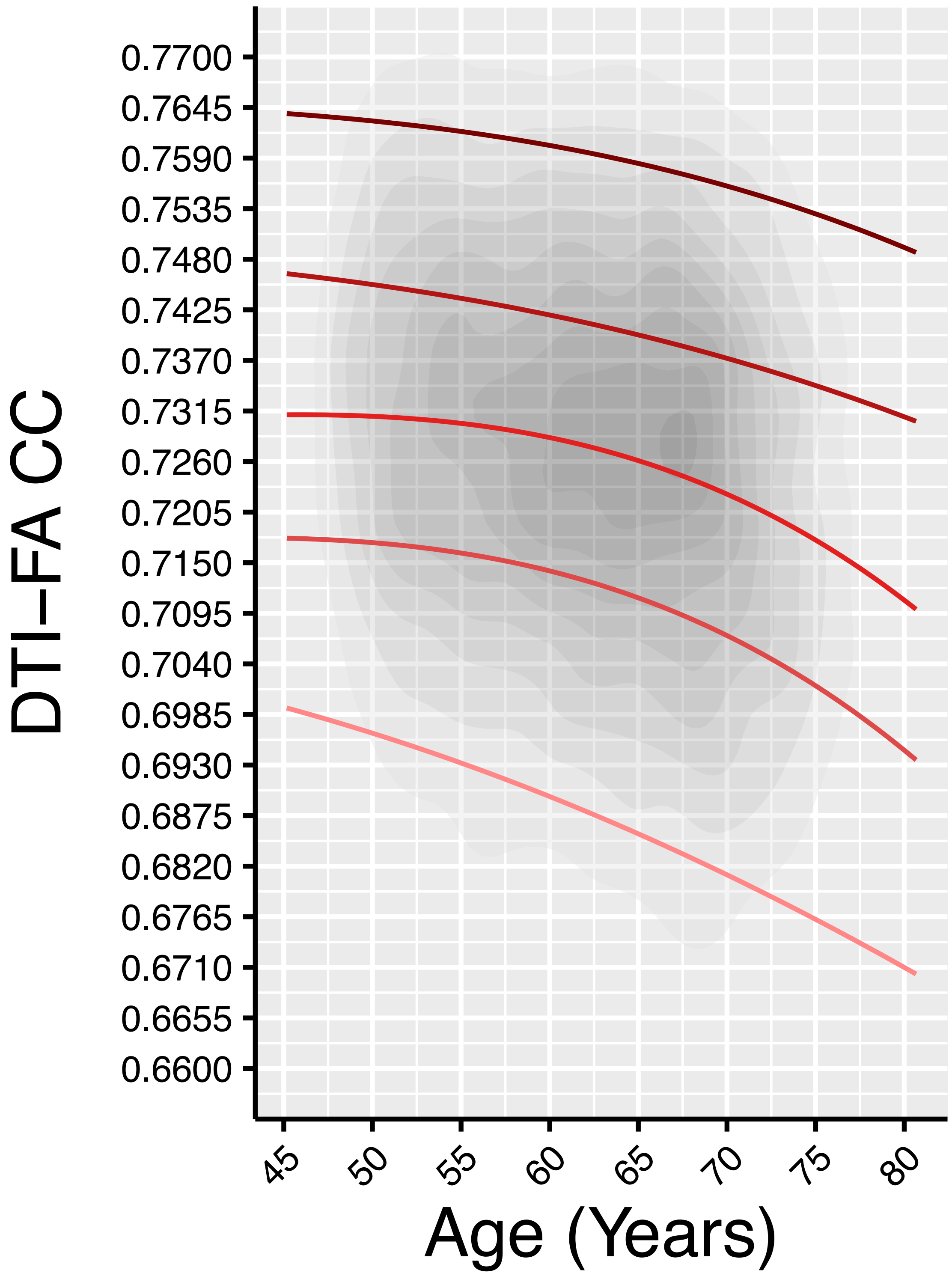

Figure S47. Full size normative centile reference curves calculated for the corpus callosum for DTI-FA in females. Solid colored lines, ordered from lightest to darkest, indicate the following centiles: 5th, 25th, 50th, 75th, 95th. Gray overlay reflects kernel density (darker=greater degree of data point overlap). CC = corpus callosum.

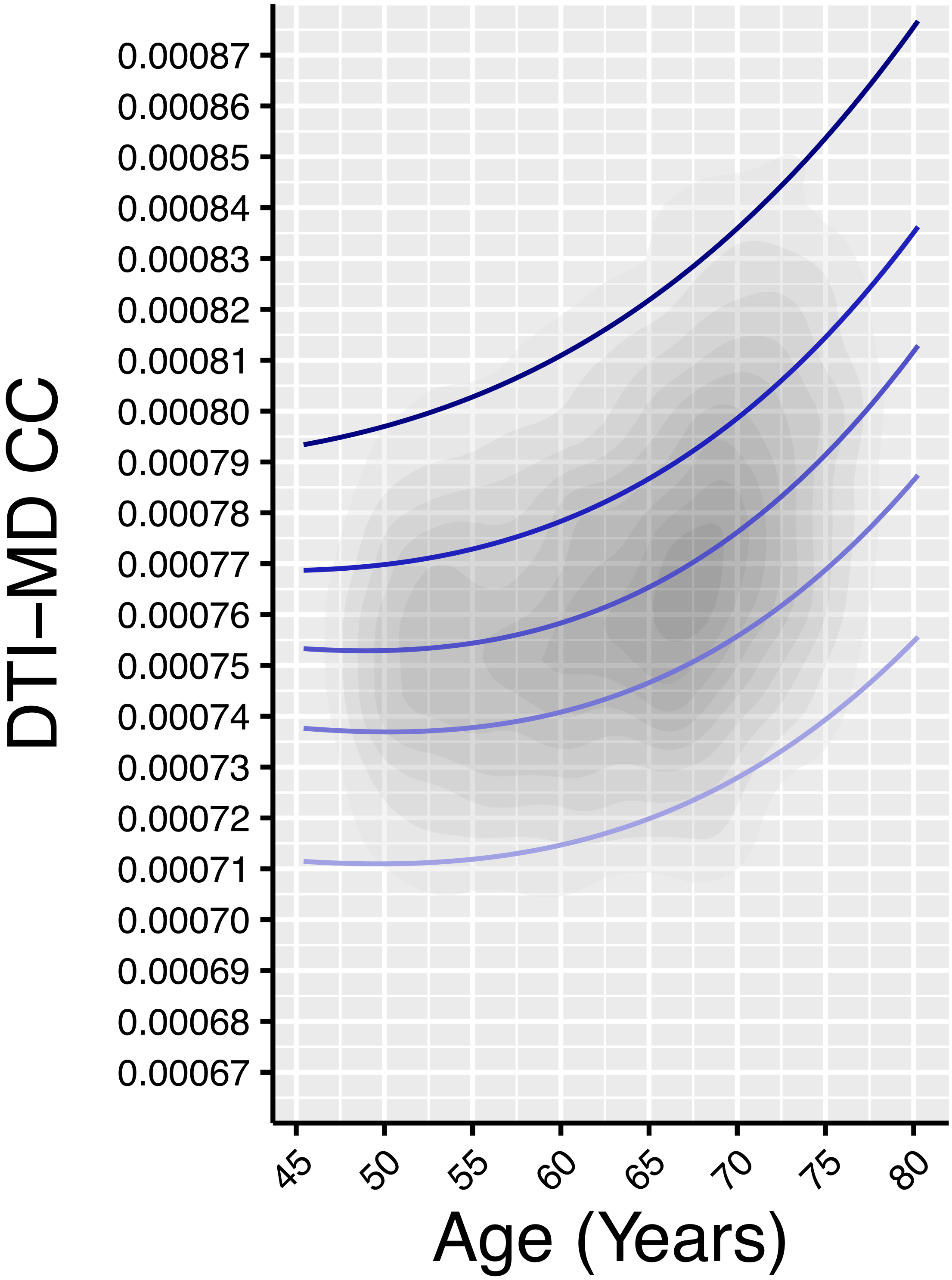

Figure S48. Full size normative centile reference curves calculated for the corpus callosum for DTI-MD in males. Solid colored lines, ordered from lightest to darkest, indicate the following centiles: 5th, 25th, 50th, 75th, 95th. Gray overlay reflects kernel density (darker=greater degree of data point overlap). CC = corpus callosum.

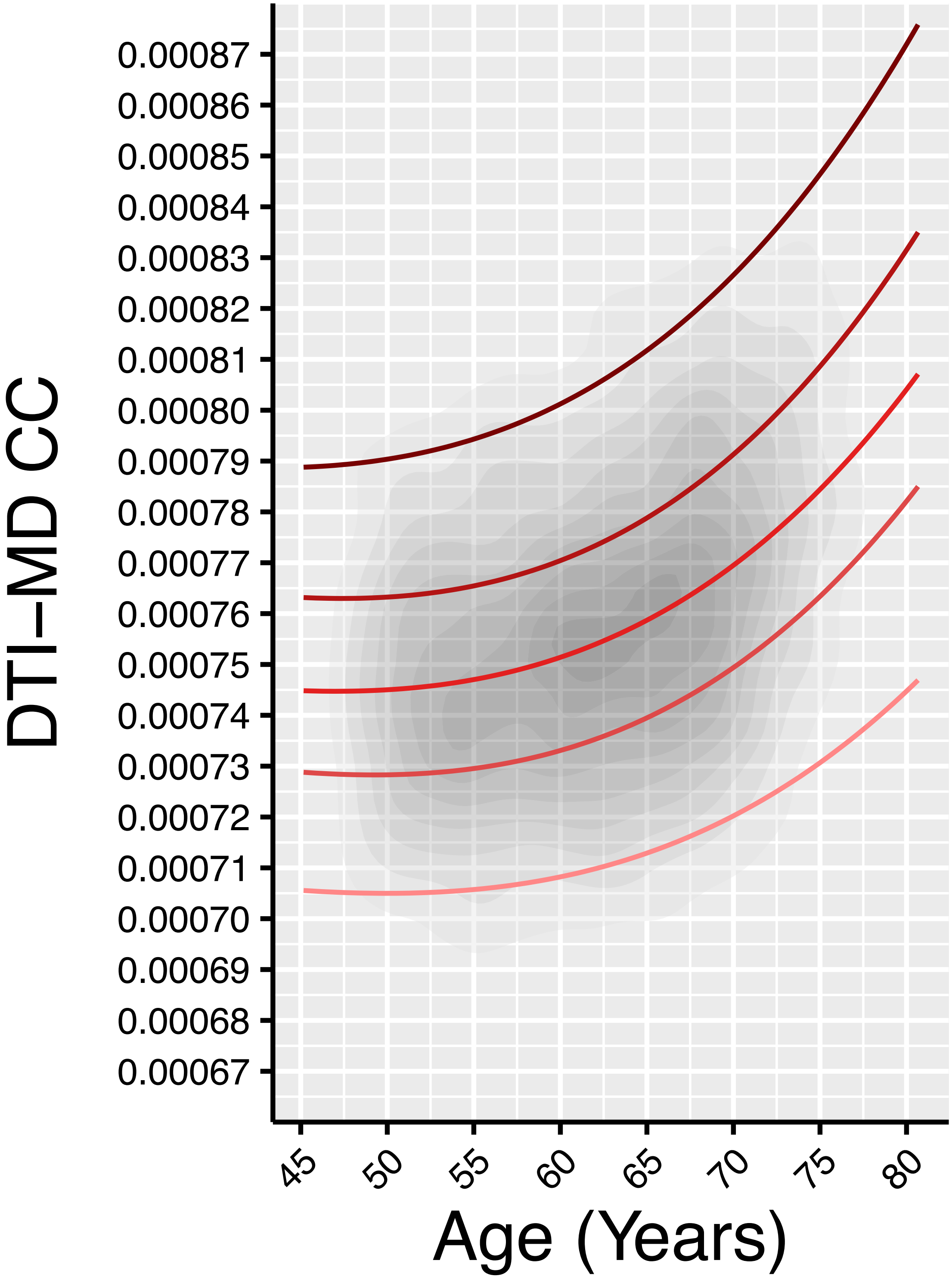

Figure S49. Full size normative centile reference curves calculated for the corpus callosum for DTI-MD in females. Solid colored lines, ordered from lightest to darkest, indicate the following centiles: 5th, 25th, 50th, 75th, 95th. Gray overlay reflects kernel density (darker=greater degree of data point overlap). CC = corpus callosum.

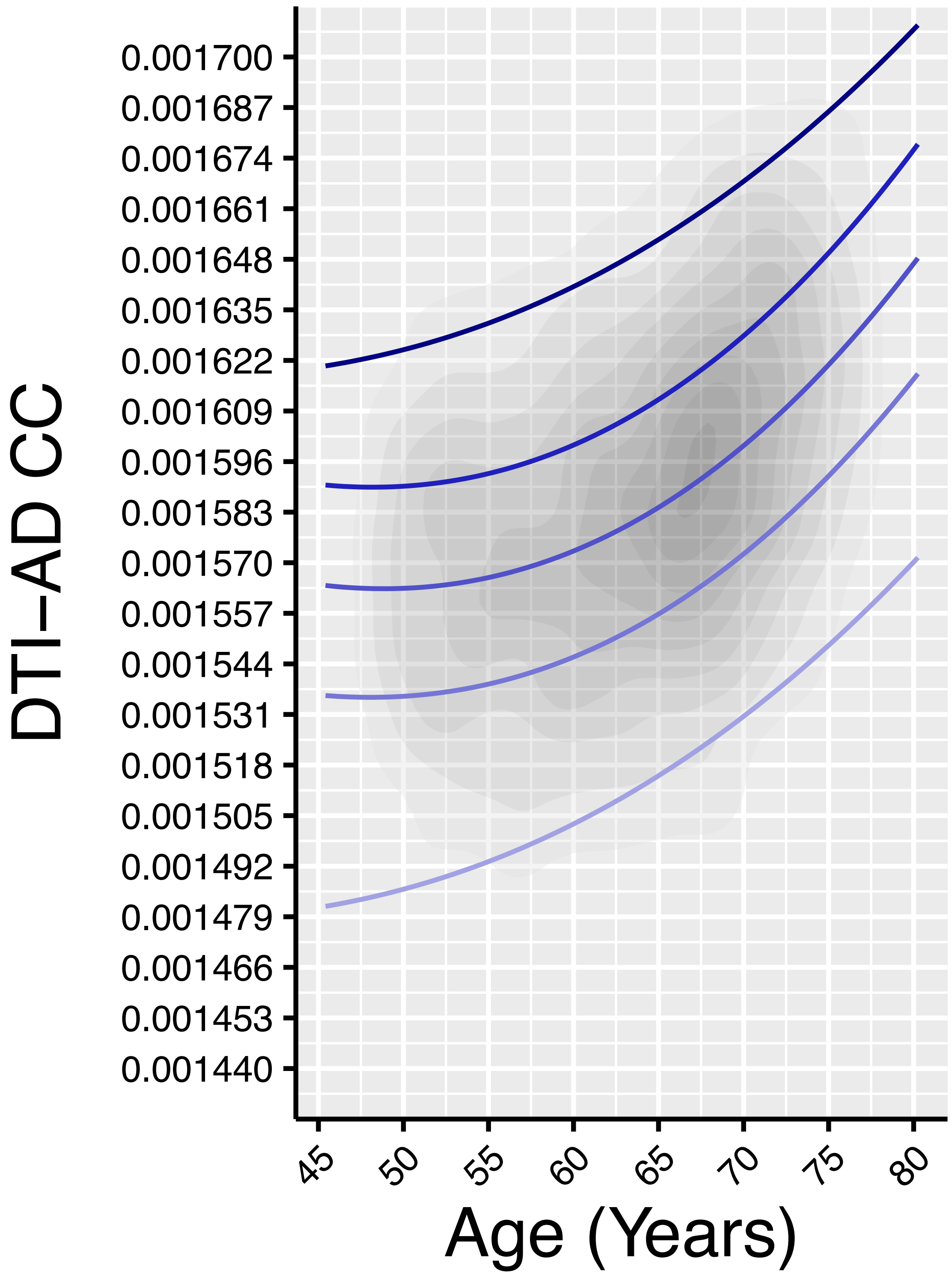

Figure S50. Full size normative centile reference curves calculated for the corpus callosum for DTI-AD in males. Solid colored lines, ordered from lightest to darkest, indicate the following centiles: 5th, 25th, 50th, 75th, 95th. Gray overlay reflects kernel density (darker=greater degree of data point overlap). CC = corpus callosum.

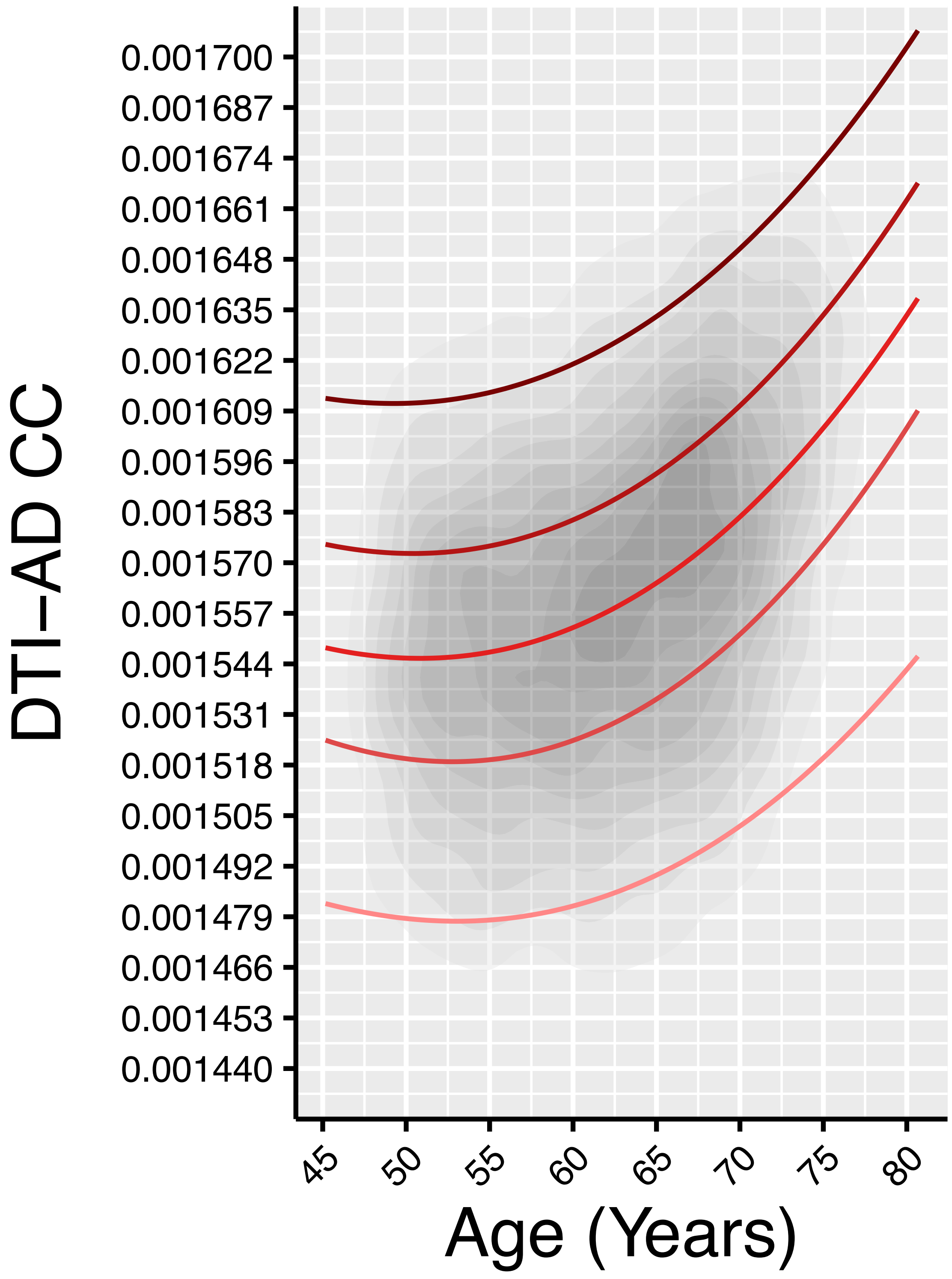

Figure S51. Full size normative centile reference curves calculated for the corpus callosum for DTI-AD in females. Solid colored lines, ordered from lightest to darkest, indicate the following centiles: 5th, 25th, 50th, 75th, 95th. Gray overlay reflects kernel density (darker=greater degree of data point overlap). CC = corpus callosum.

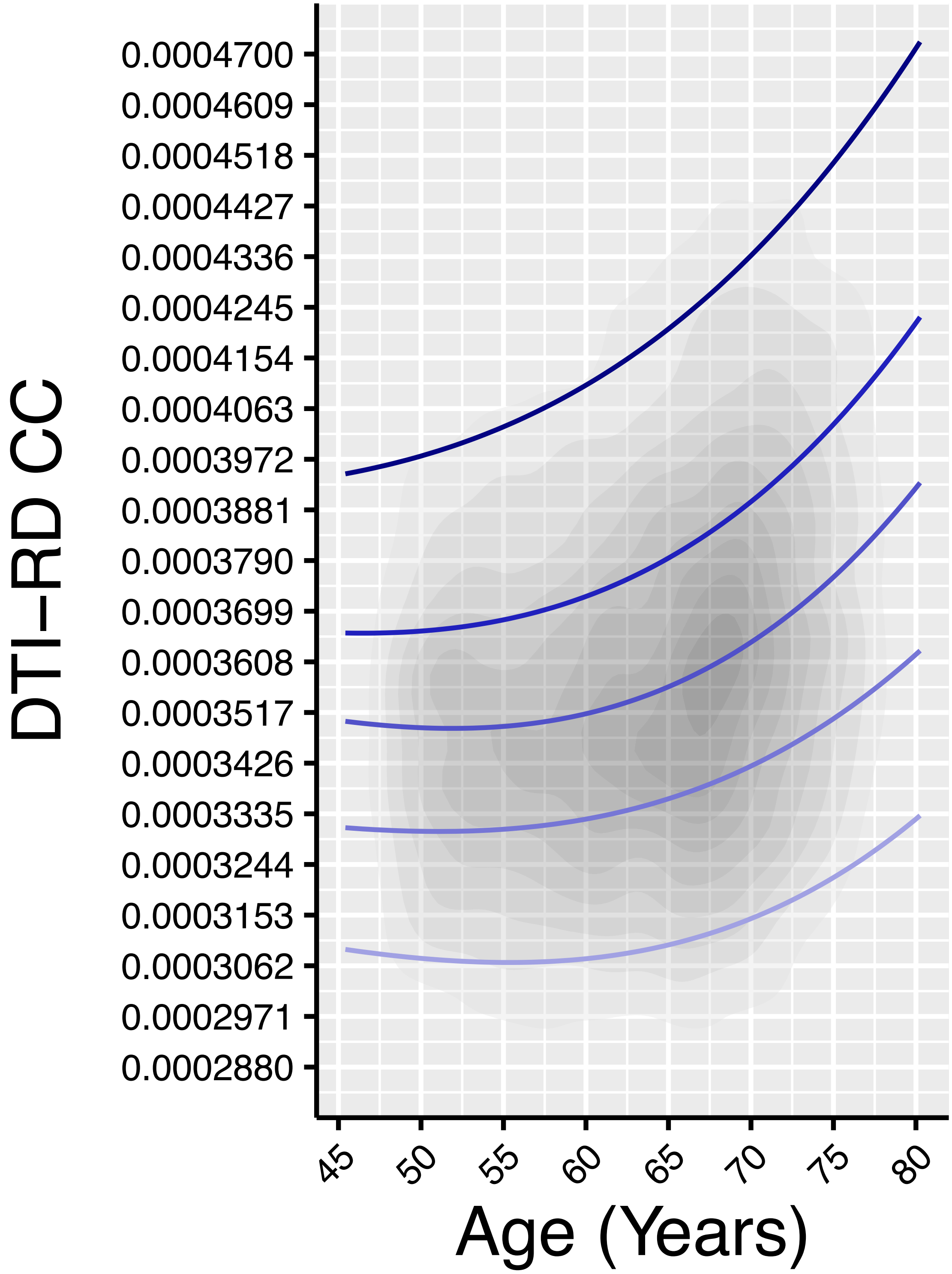

Figure S52. Full size normative centile reference curves calculated for the corpus callosum for DTI-RD in males. Solid colored lines, ordered from lightest to darkest, indicate the following centiles: 5th, 25th, 50th, 75th, 95th. Gray overlay reflects kernel density (darker=greater degree of data point overlap). CC = corpus callosum.

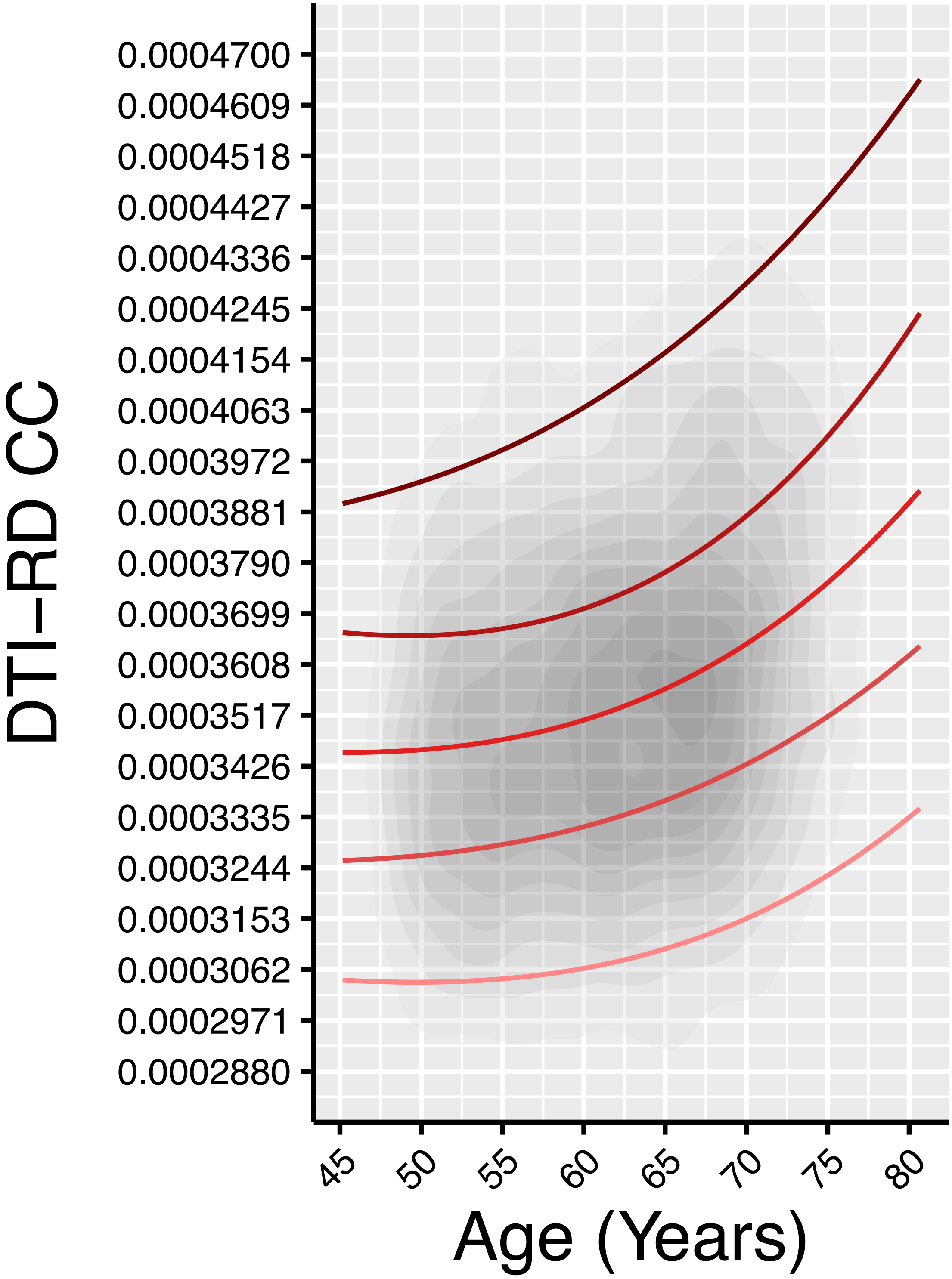

Figure S53. Full size normative centile reference curves calculated for the corpus callosum for DTI-RD in females. Solid colored lines, ordered from lightest to darkest, indicate the following centiles: 5th, 25th, 50th, 75th, 95th. Gray overlay reflects kernel density (darker=greater degree of data point overlap). CC = corpus callosum.

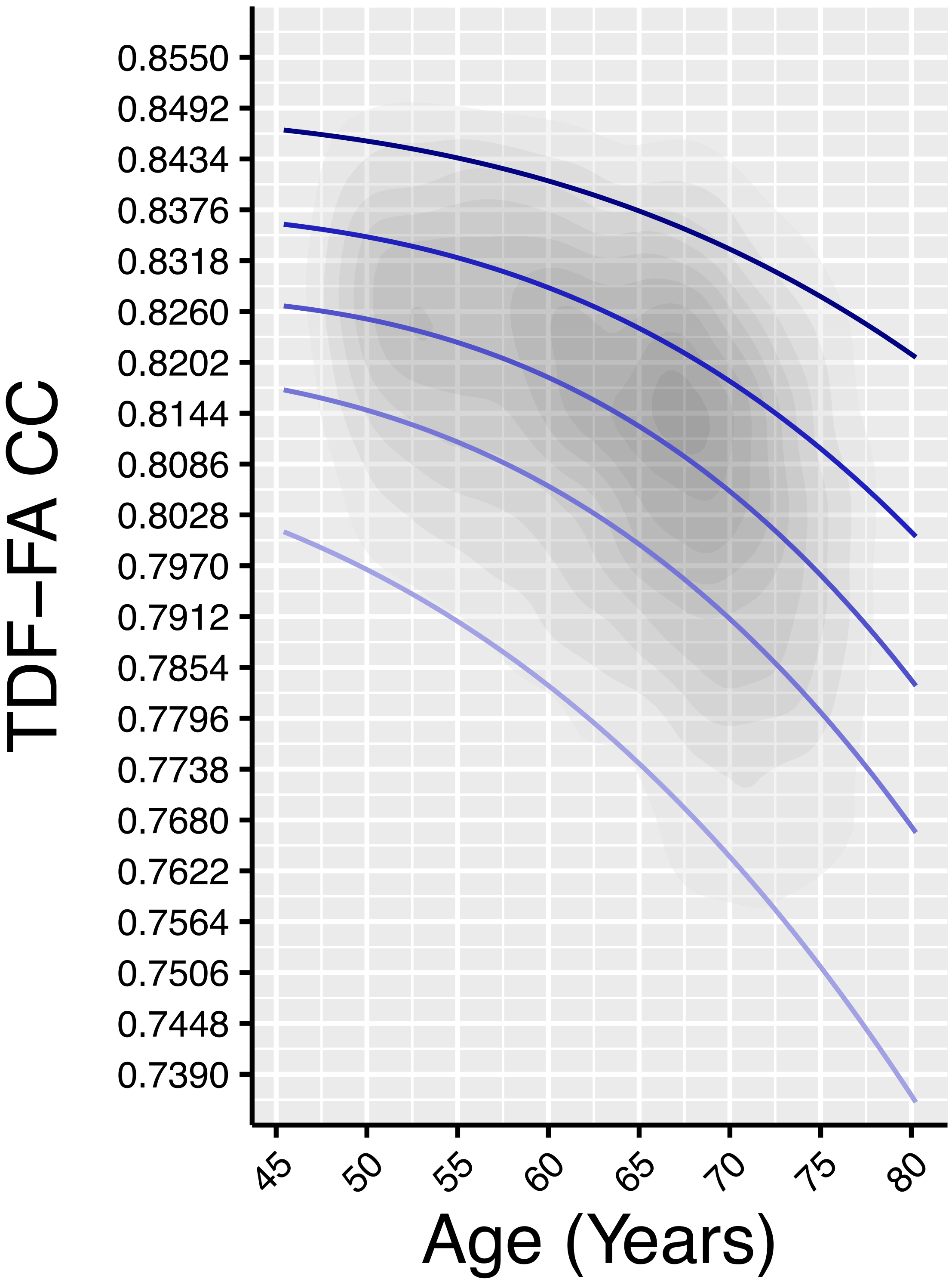

Figure S54. Full size normative centile reference curves calculated for the corpus callosum for TDF-FA in males. Solid colored lines, ordered from lightest to darkest, indicate the following centiles: 5th, 25th, 50th, 75th, 95th. Gray overlay reflects kernel density (darker=greater degree of data point overlap). CC = corpus callosum.

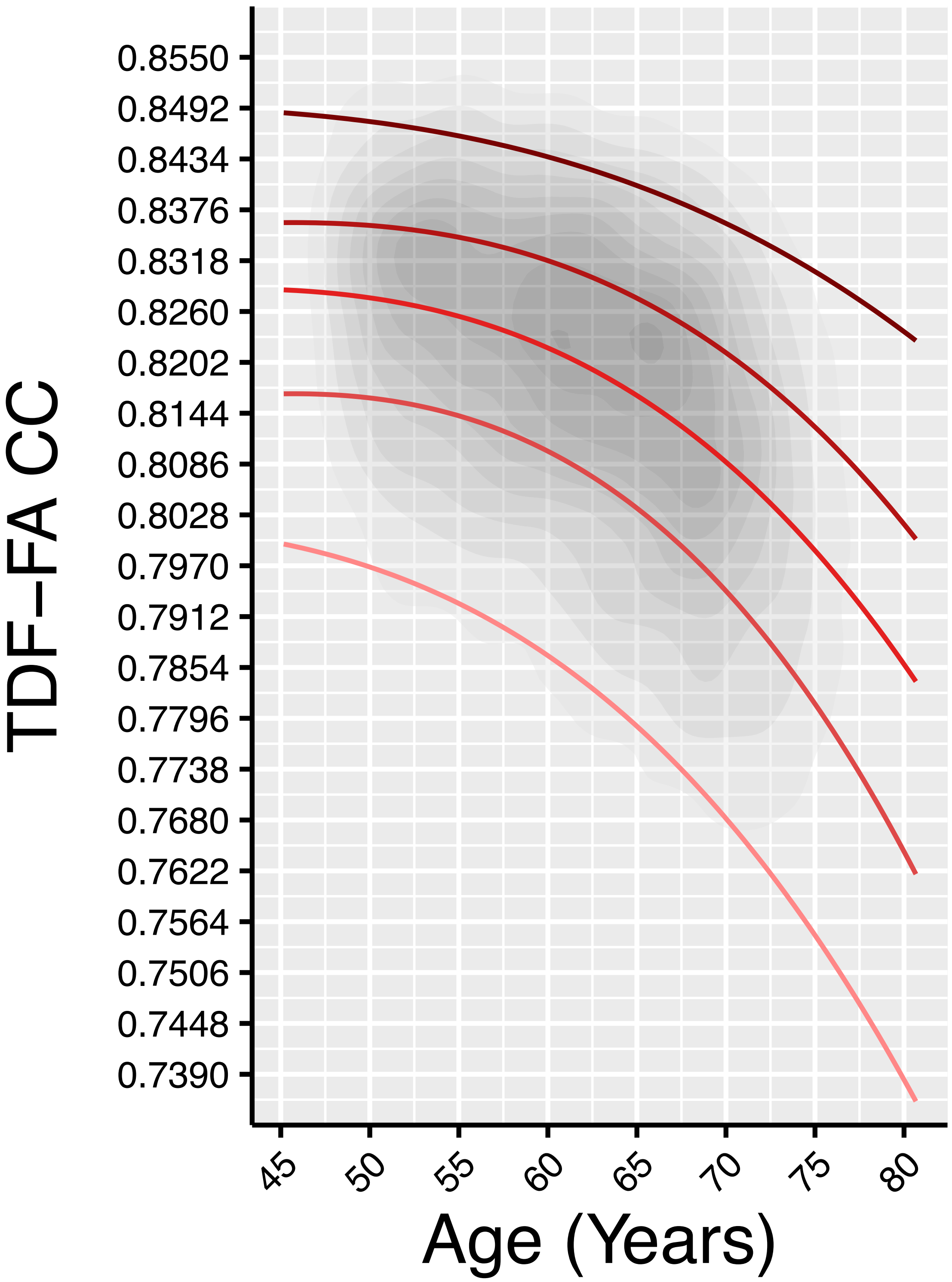

Figure S55. Full size normative centile reference curves calculated for the corpus callosum for TDF-FA in females. Solid colored lines, ordered from lightest to darkest, indicate the following centiles: 5th, 25th, 50th, 75th, 95th. Gray overlay reflects kernel density (darker=greater degree of data point overlap). CC = corpus callosum.

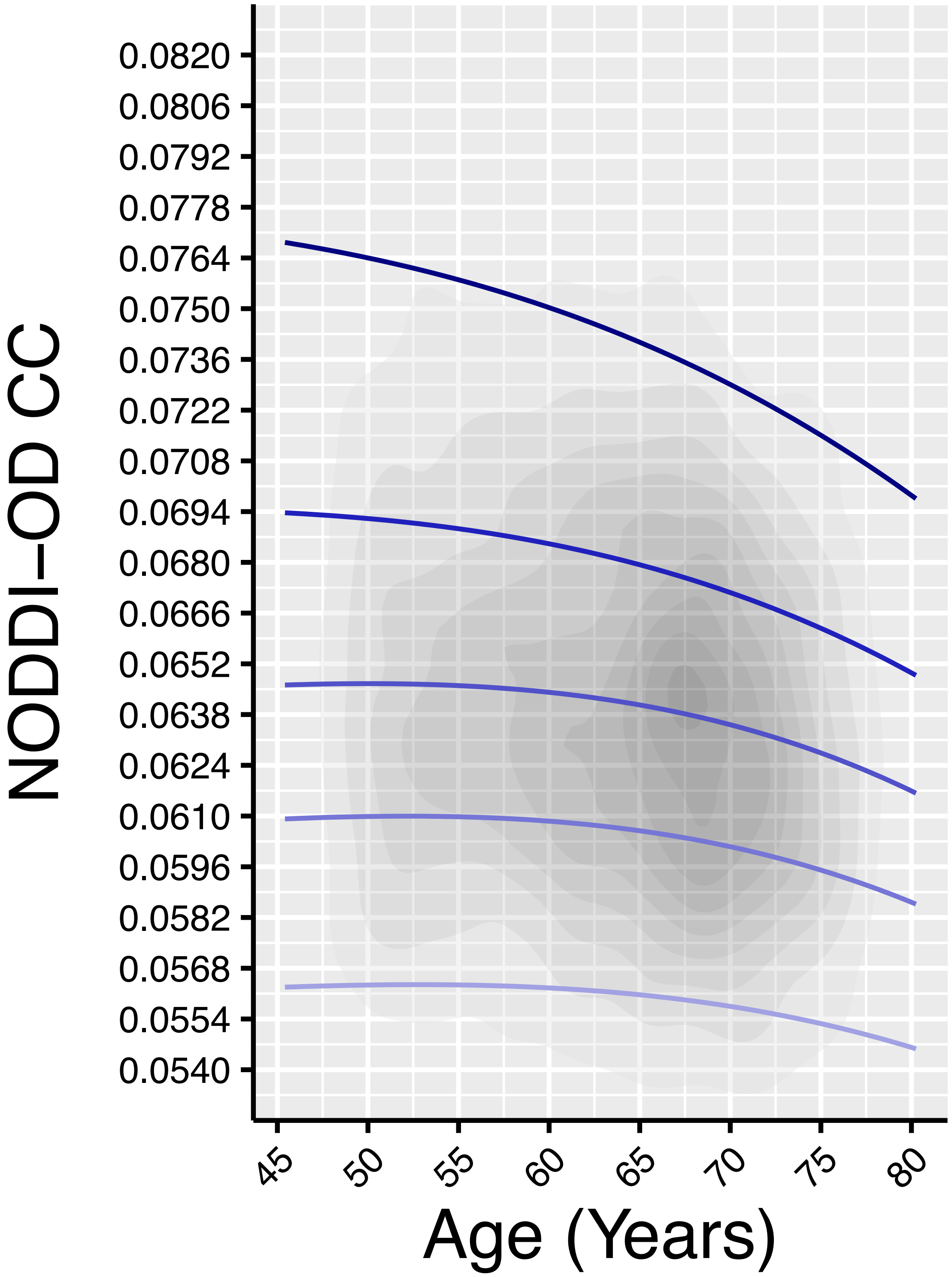

Figure S56. Full size normative centile reference curves calculated for the corpus callosum for NODDI-OD in males. Solid colored lines, ordered from lightest to darkest, indicate the following centiles: 5th, 25th, 50th, 75th, 95th. Gray overlay reflects kernel density (darker=greater degree of data point overlap). CC = corpus callosum.

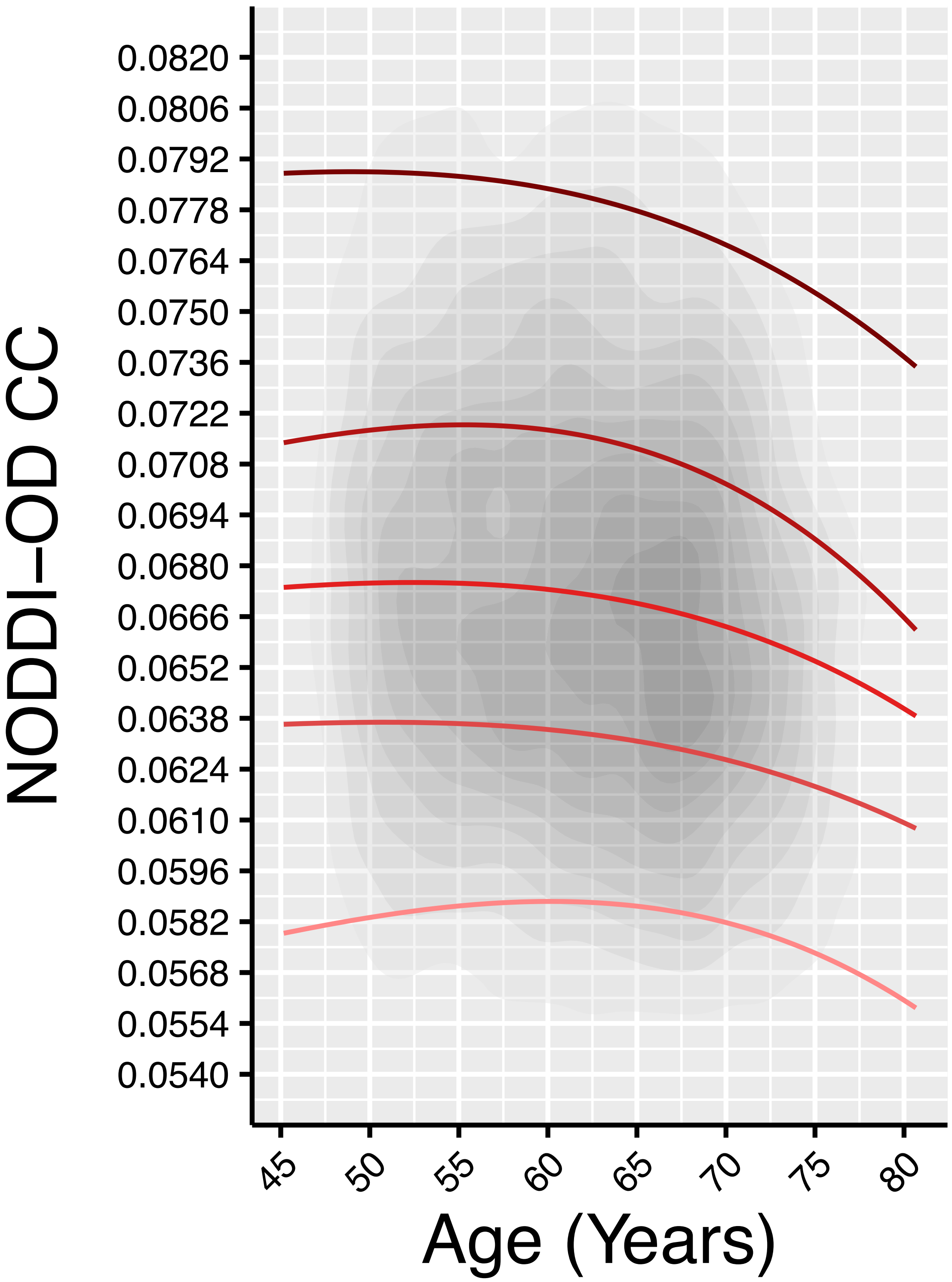

Figure S57. Full size normative centile reference curves calculated for the corpus callosum for NODDI-OD in females. Solid colored lines, ordered from lightest to darkest, indicate the following centiles: 5th, 25th, 50th, 75th, 95th. Gray overlay reflects kernel density (darker=greater degree of data point overlap). CC = corpus callosum.

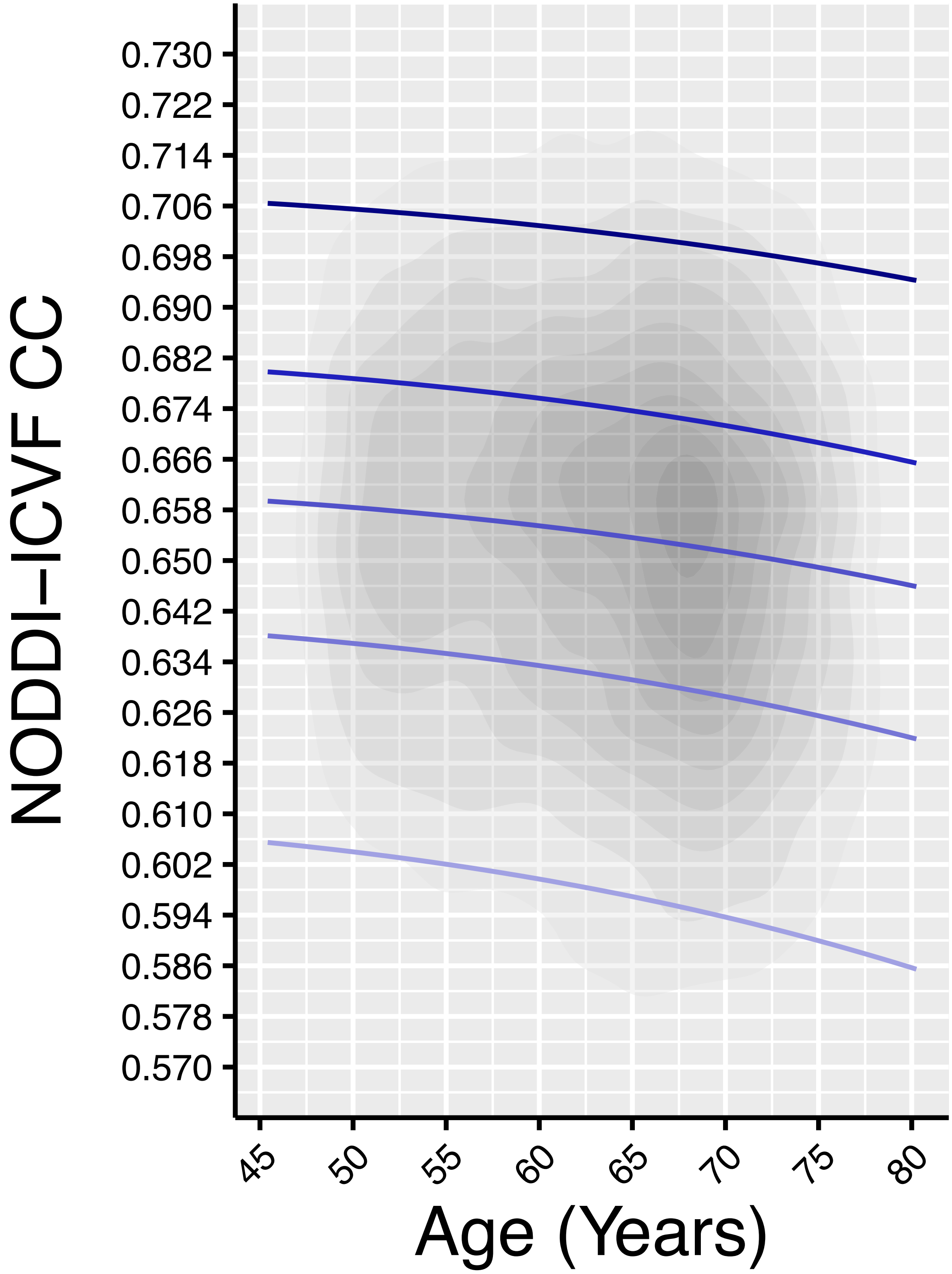

Figure S58. Full size normative centile reference curves calculated for the corpus callosum for NODDI-ICVF in males. Solid colored lines, ordered from lightest to darkest, indicate the following centiles: 5th, 25th, 50th, 75th, 95th. Gray overlay reflects kernel density (darker=greater degree of data point overlap). CC = corpus callosum.

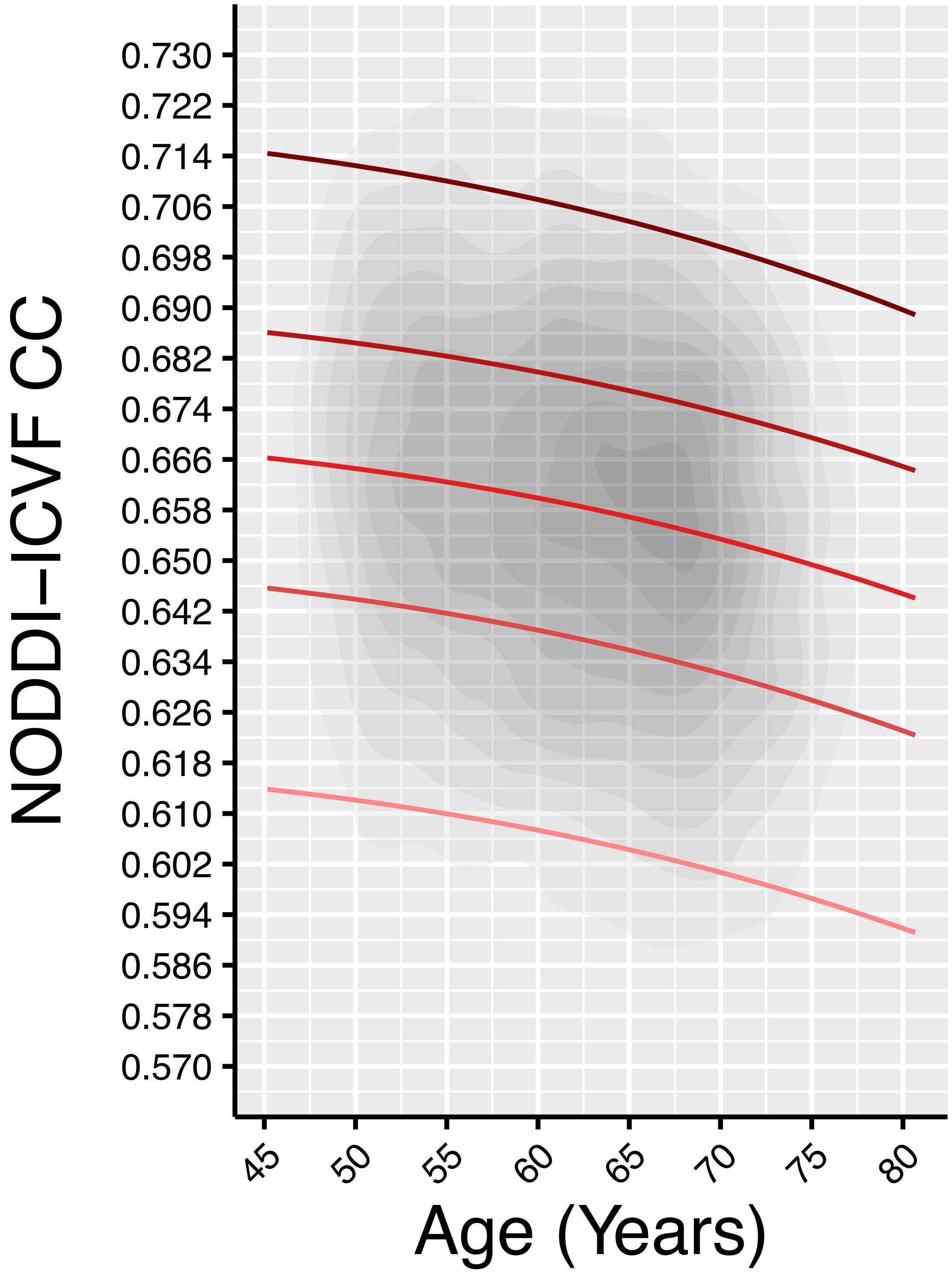

Figure S59. Full size normative centile reference curves calculated for the corpus callosum for NODDI-ICVF in females. Solid colored lines, ordered from lightest to darkest, indicate the following centiles: 5th, 25th, 50th, 75th, 95th. Gray overlay reflects kernel density (darker=greater degree of data point overlap). CC = corpus callosum.

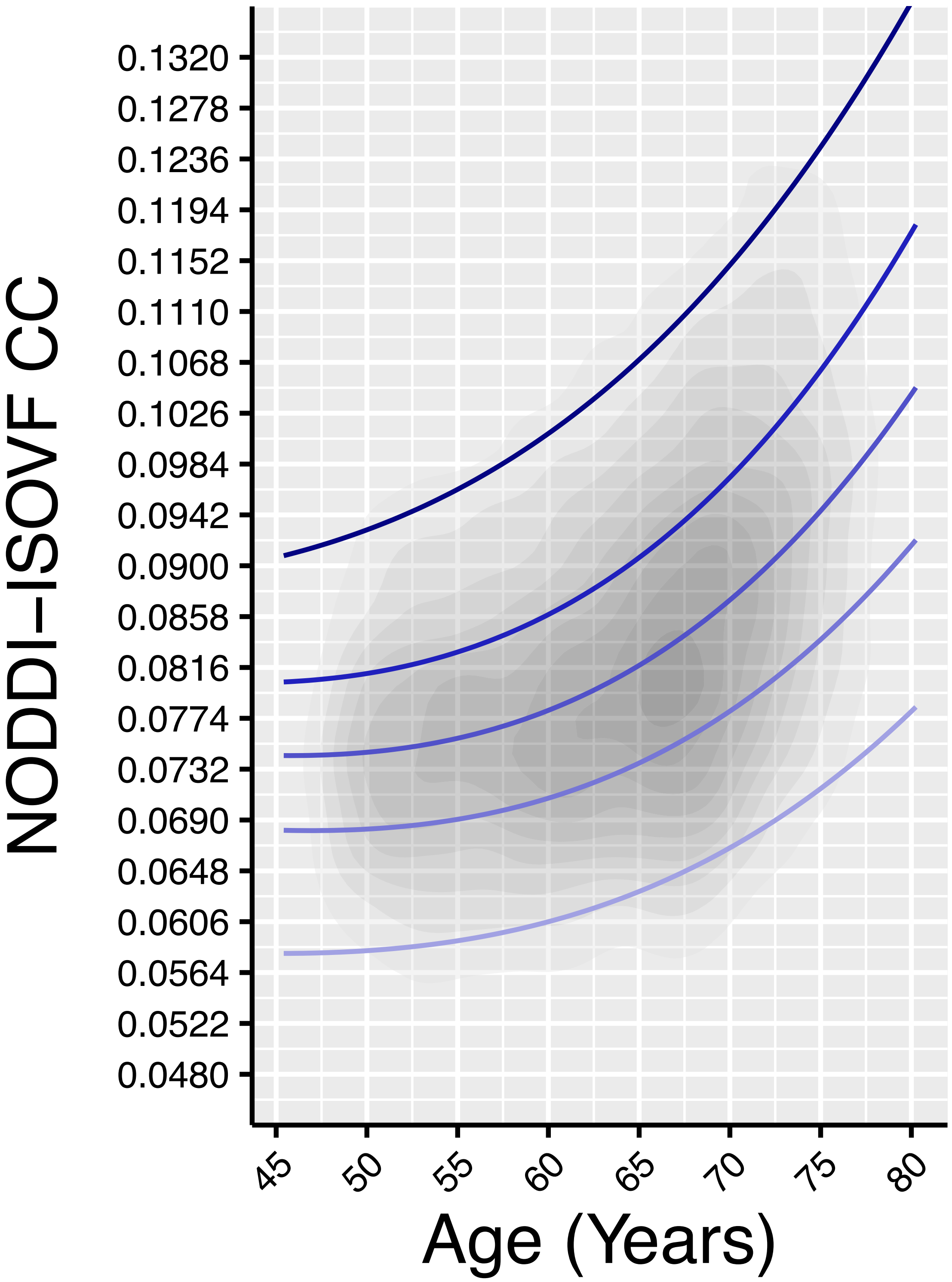

Figure S60. Full size normative centile reference curves calculated for the corpus callosum for NODDI-ISOVF in males. Solid colored lines, ordered from lightest to darkest, indicate the following centiles: 5th, 25th, 50th, 75th, 95th. Gray overlay reflects kernel density (darker=greater degree of data point overlap). CC = corpus callosum.

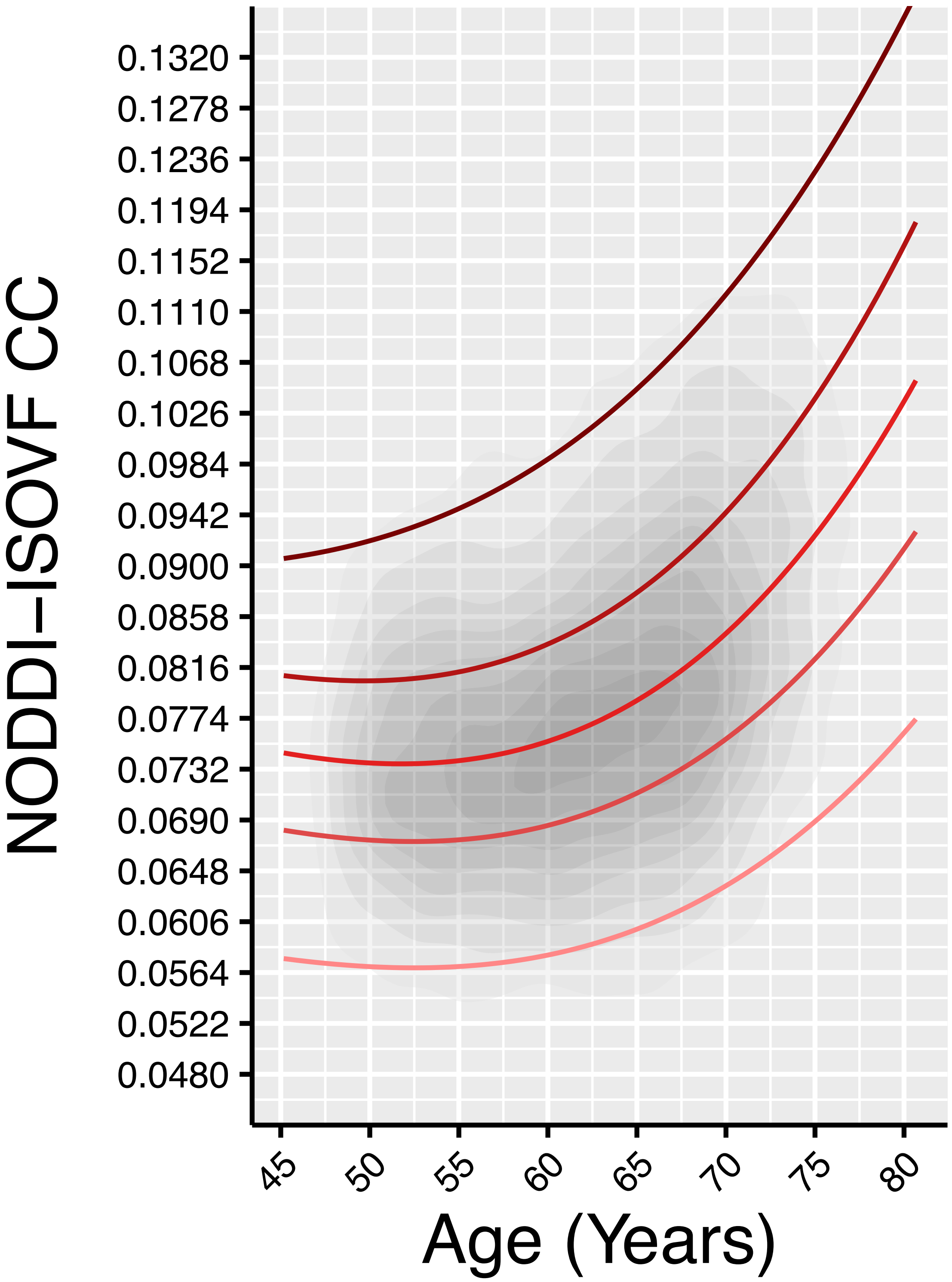

Figure S61. Full size normative centile reference curves calculated for the corpus callosum for NODDI-ISOVF in females. Solid colored lines, ordered from lightest to darkest, indicate the following centiles: 5th, 25th, 50th, 75th, 95th. Gray overlay reflects kernel density (darker=greater degree of data point overlap). CC = corpus callosum.

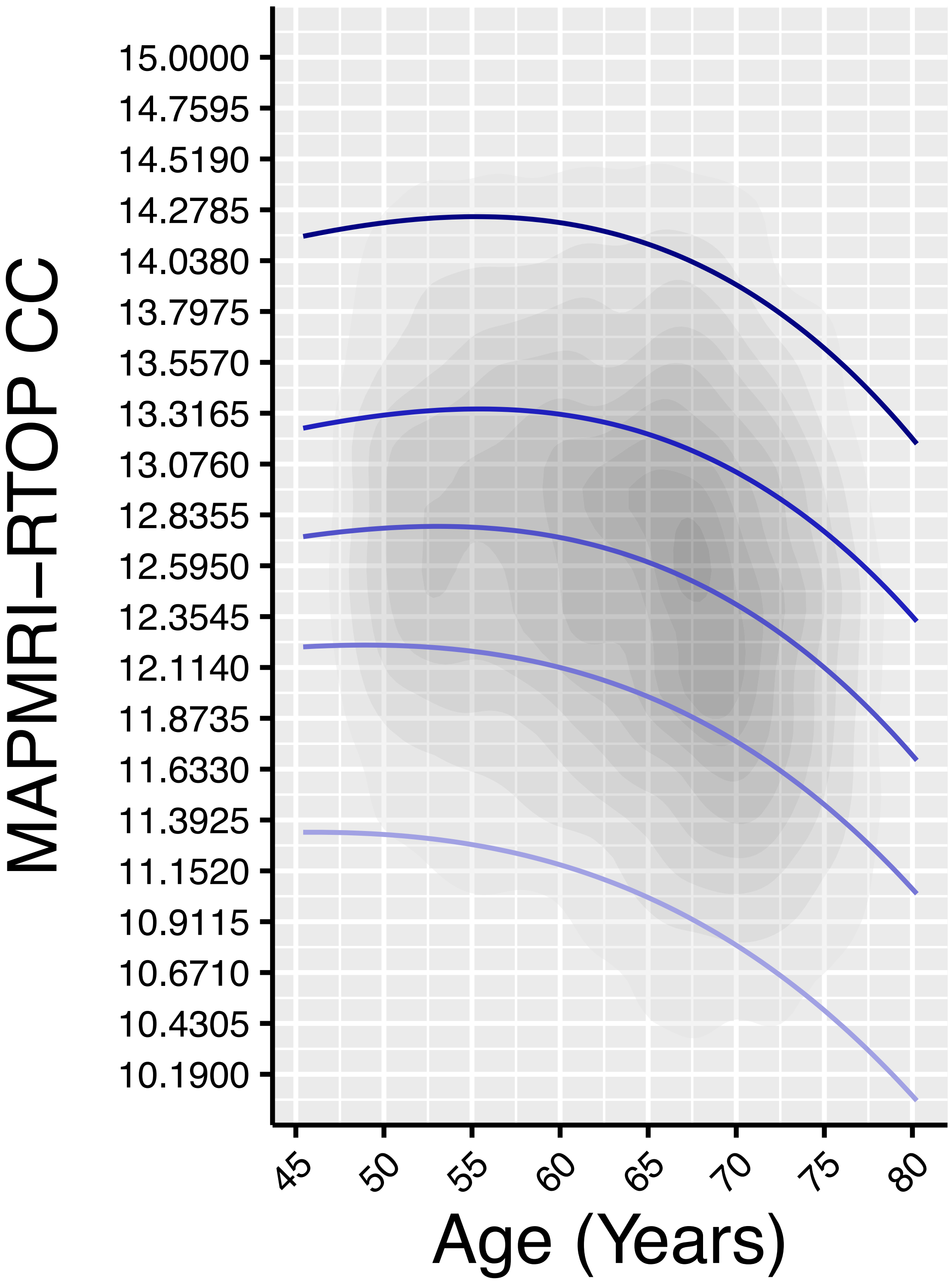

Figure S62. Full size normative centile reference curves calculated for the corpus callosum for MAPMRI-RTOP in males. Solid colored lines, ordered from lightest to darkest, indicate the following centiles: 5th, 25th, 50th, 75th, 95th. Gray overlay reflects kernel density (darker=greater degree of data point overlap). CC = corpus callosum.

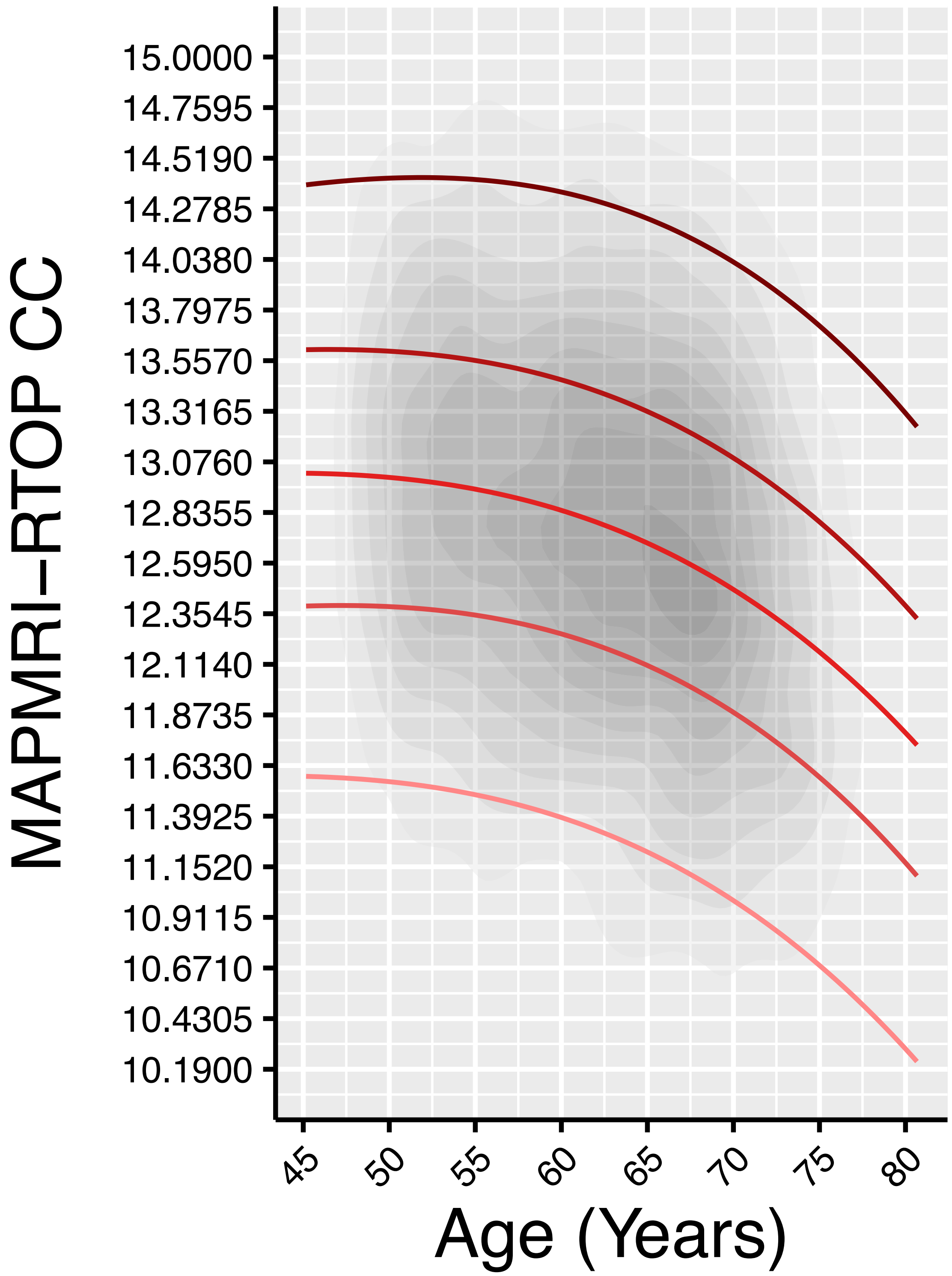

Figure S63. Full size normative centile reference curves calculated for the corpus callosum for MAPMRI-RTOP in females. Solid colored lines, ordered from lightest to darkest, indicate the following centiles: 5th, 25th, 50th, 75th, 95th. Gray overlay reflects kernel density (darker=greater degree of data point overlap). CC = corpus callosum.

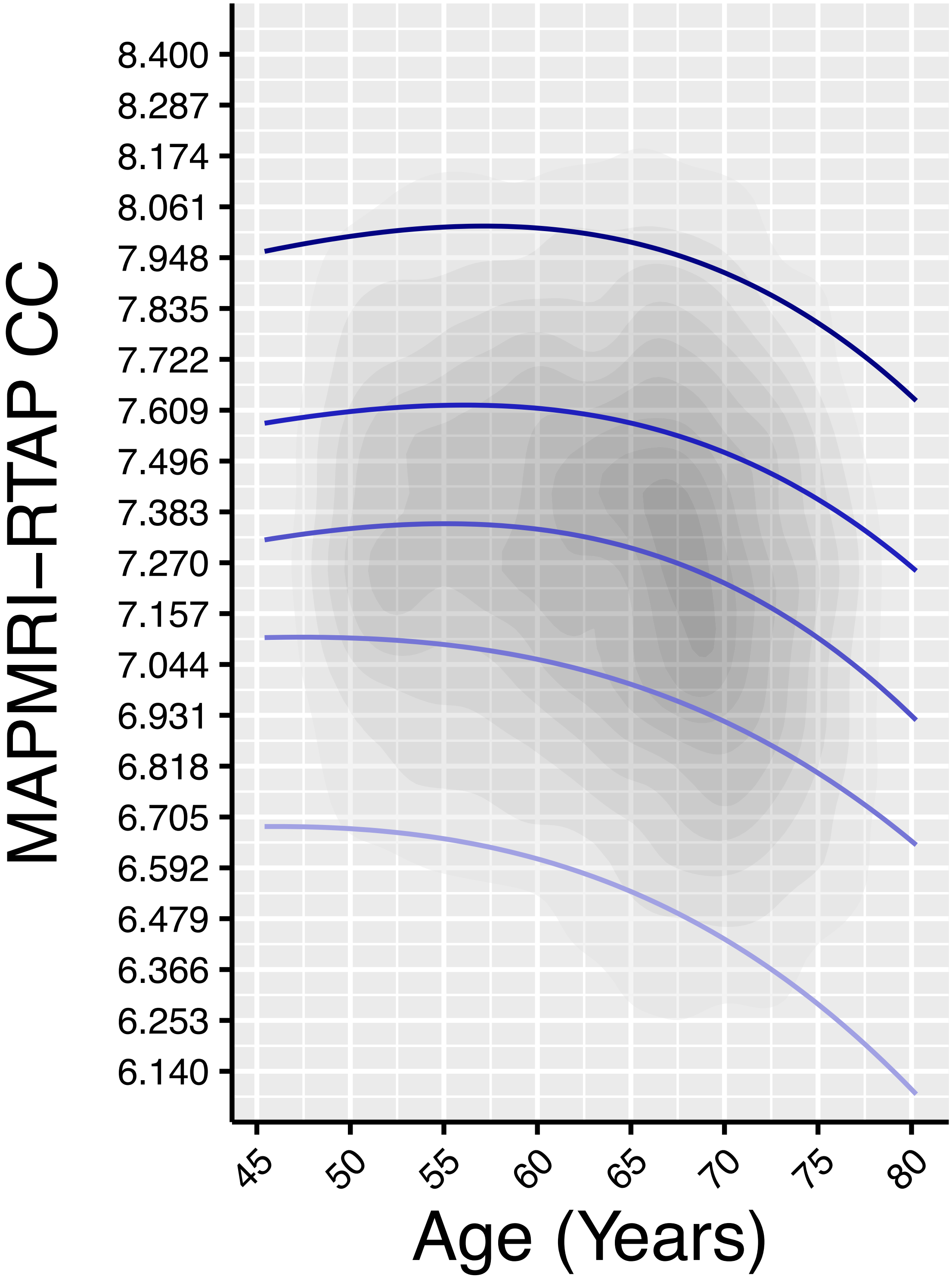

Figure S64. Full size normative centile reference curves calculated for the corpus callosum for MAPMRI-RTAP in males. Solid colored lines, ordered from lightest to darkest, indicate the following centiles: 5th, 25th, 50th, 75th, 95th. Gray overlay reflects kernel density (darker=greater degree of data point overlap). CC = corpus callosum.

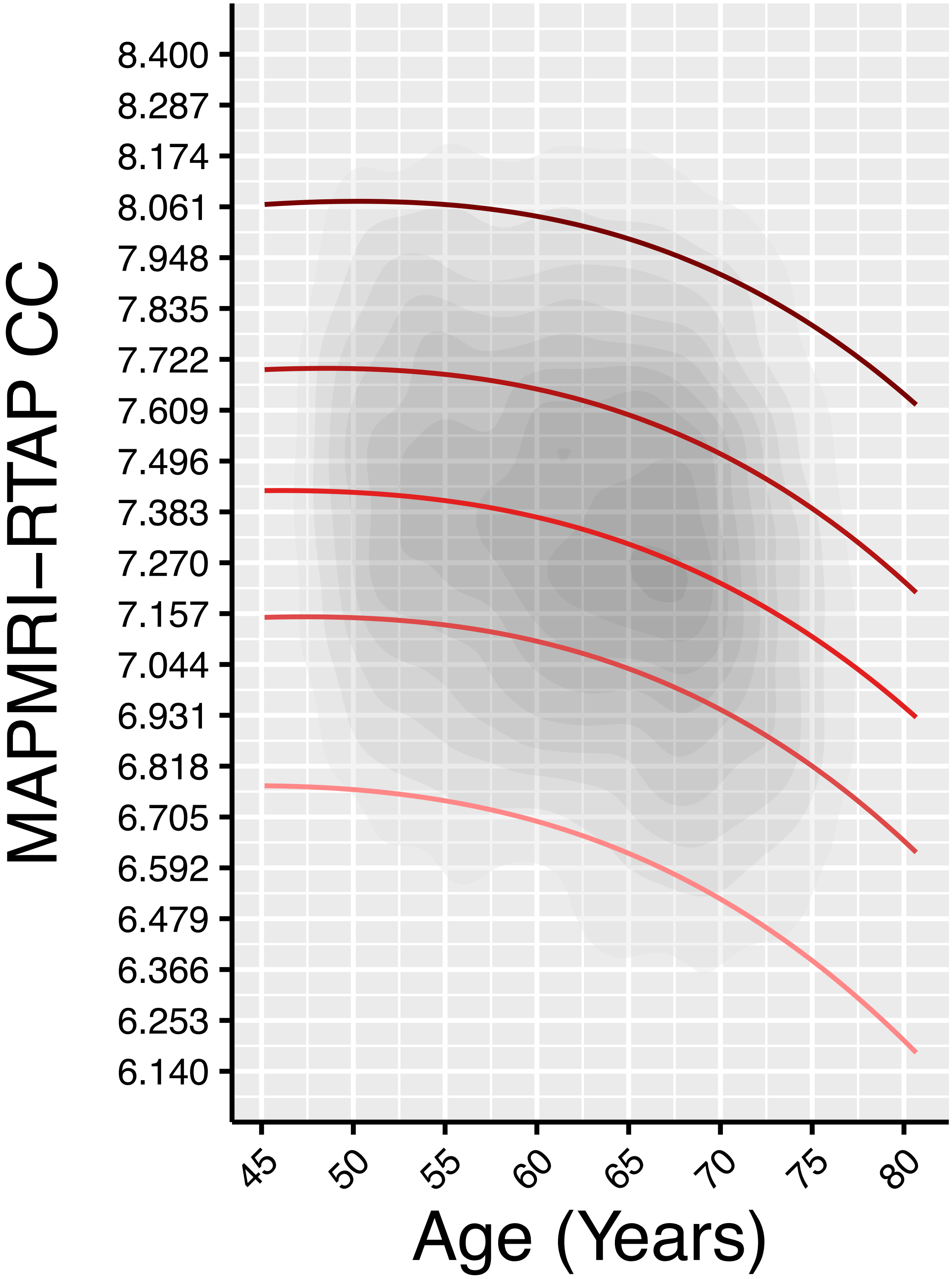

Figure S65. Full size normative centile reference curves calculated for the corpus callosum for MAPMRI-RTAP in females. Solid colored lines, ordered from lightest to darkest, indicate the following centiles: 5th, 25th, 50th, 75th, 95th. Gray overlay reflects kernel density (darker=greater degree of data point overlap). CC = corpus callosum.

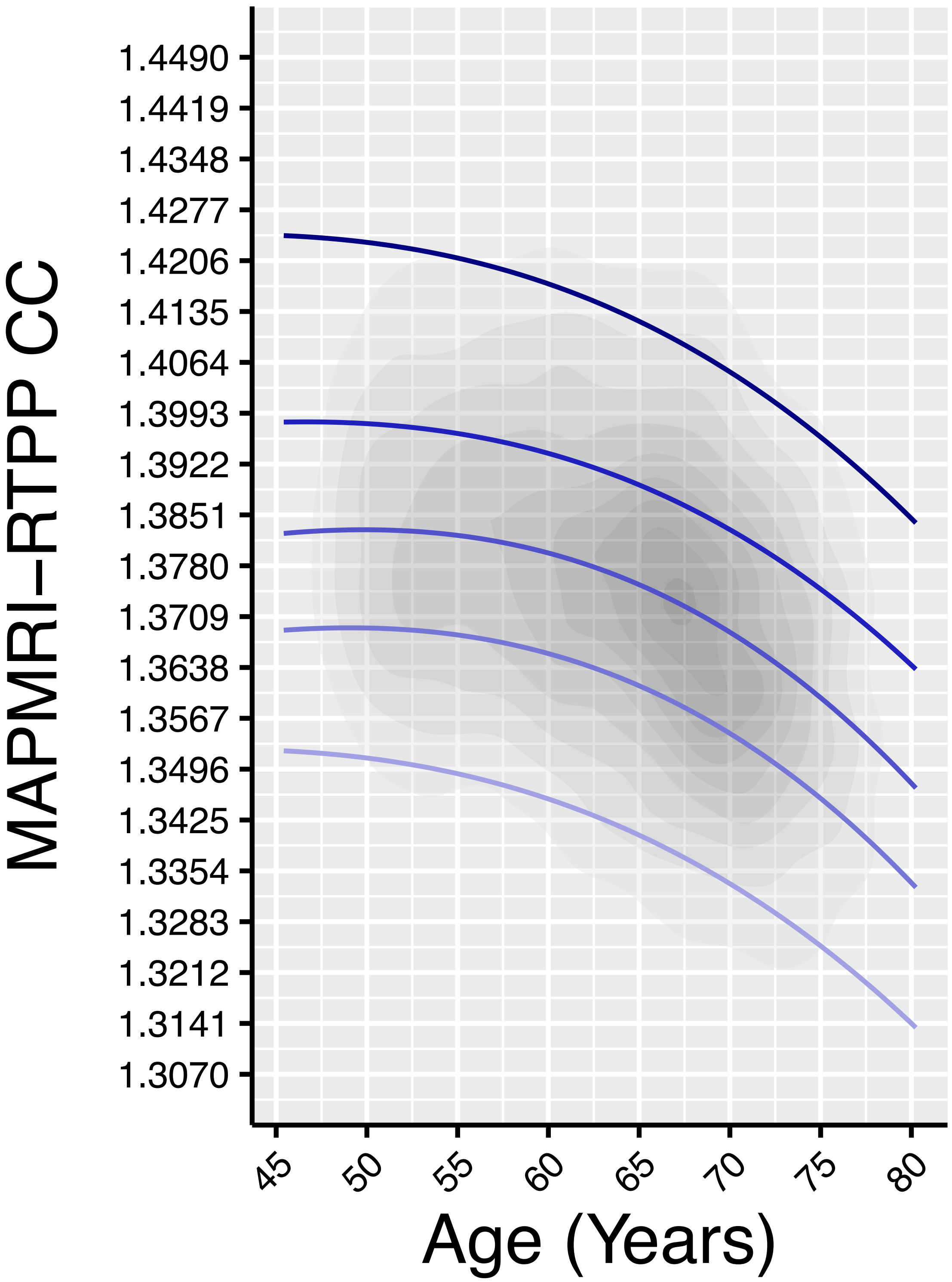

Figure S66. Full size normative centile reference curves calculated for the corpus callosum for MAPMRI-RTPP in males. Solid colored lines, ordered from lightest to darkest, indicate the following centiles: 5th, 25th, 50th, 75th, 95th. Gray overlay reflects kernel density (darker=greater degree of data point overlap). CC = corpus callosum.

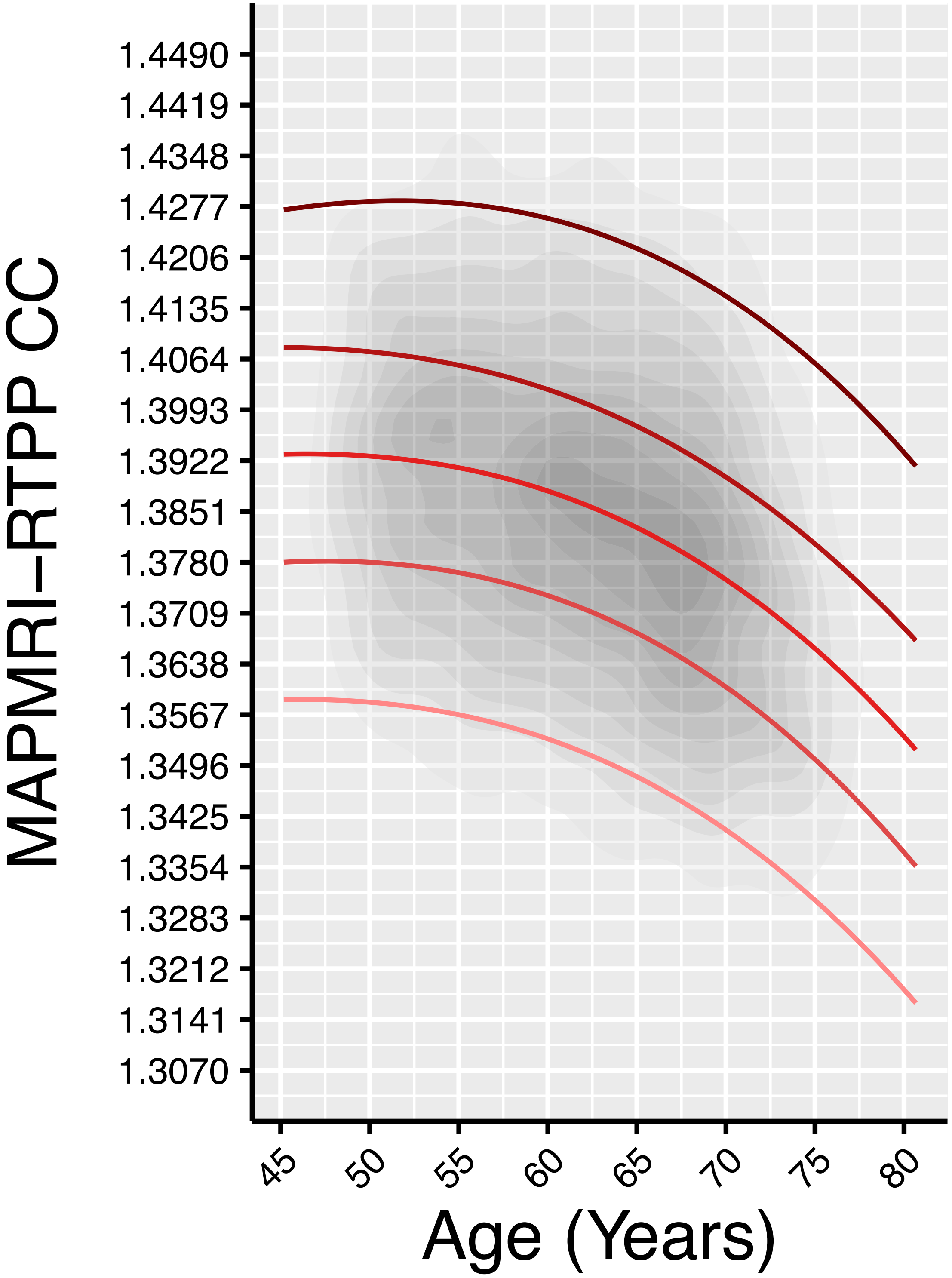

Figure S67. Full size normative centile reference curves calculated for the corpus callosum for MAPMRI-RTPP in females. Solid colored lines, ordered from lightest to darkest, indicate the following centiles: 5th, 25th, 50th, 75th, 95th. Gray overlay reflects kernel density (darker=greater degree of data point overlap). CC = corpus callosum.

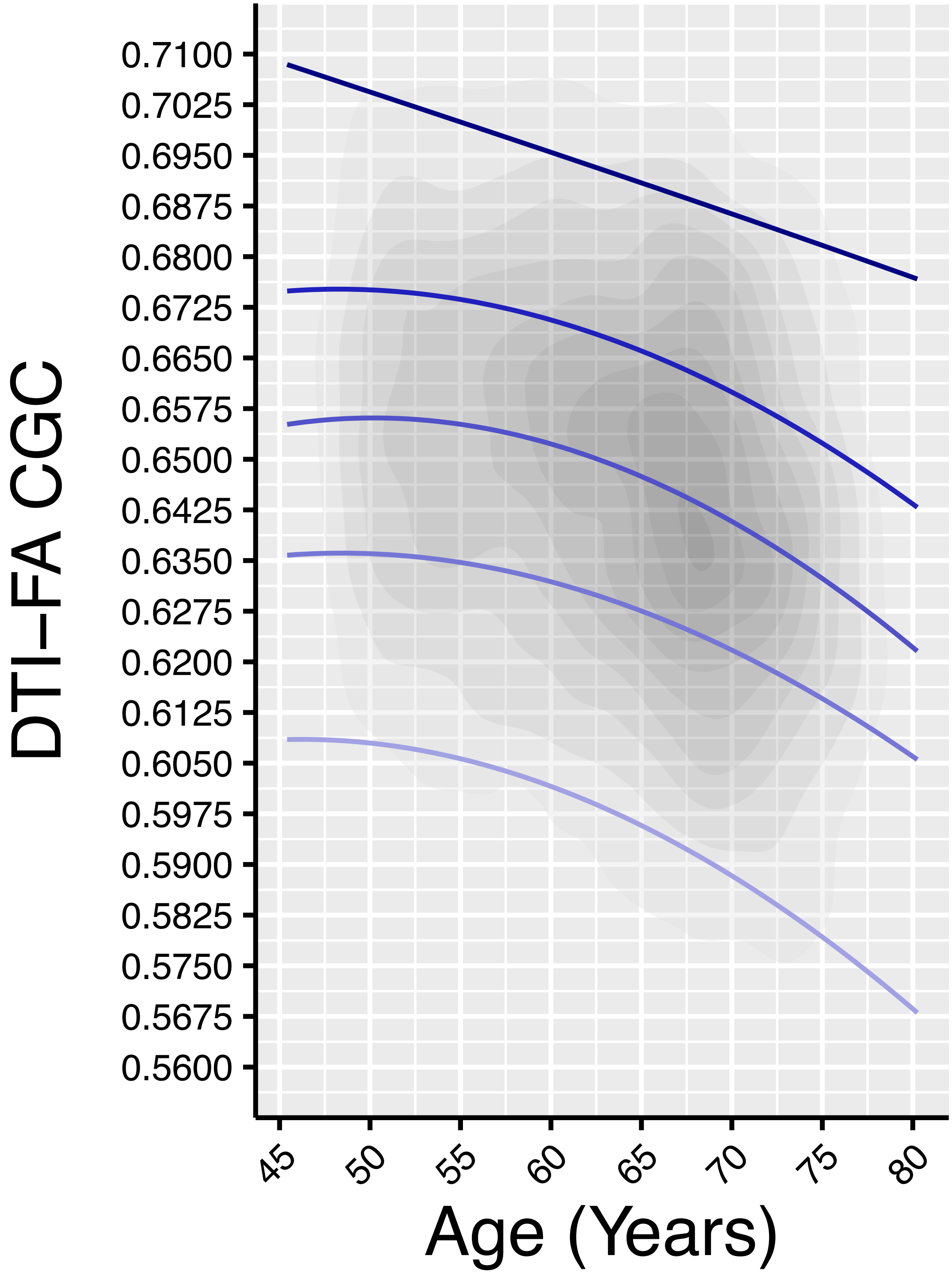

Figure S68. Full size normative centile reference curves calculated for the cingulum (cingulate) for DTI-FA in males. Solid colored lines, ordered from lightest to darkest, indicate the following centiles: 5th, 25th, 50th, 75th, 95th. Gray overlay reflects kernel density (darker=greater degree of data point overlap). CGC = cingulum (cingulate).

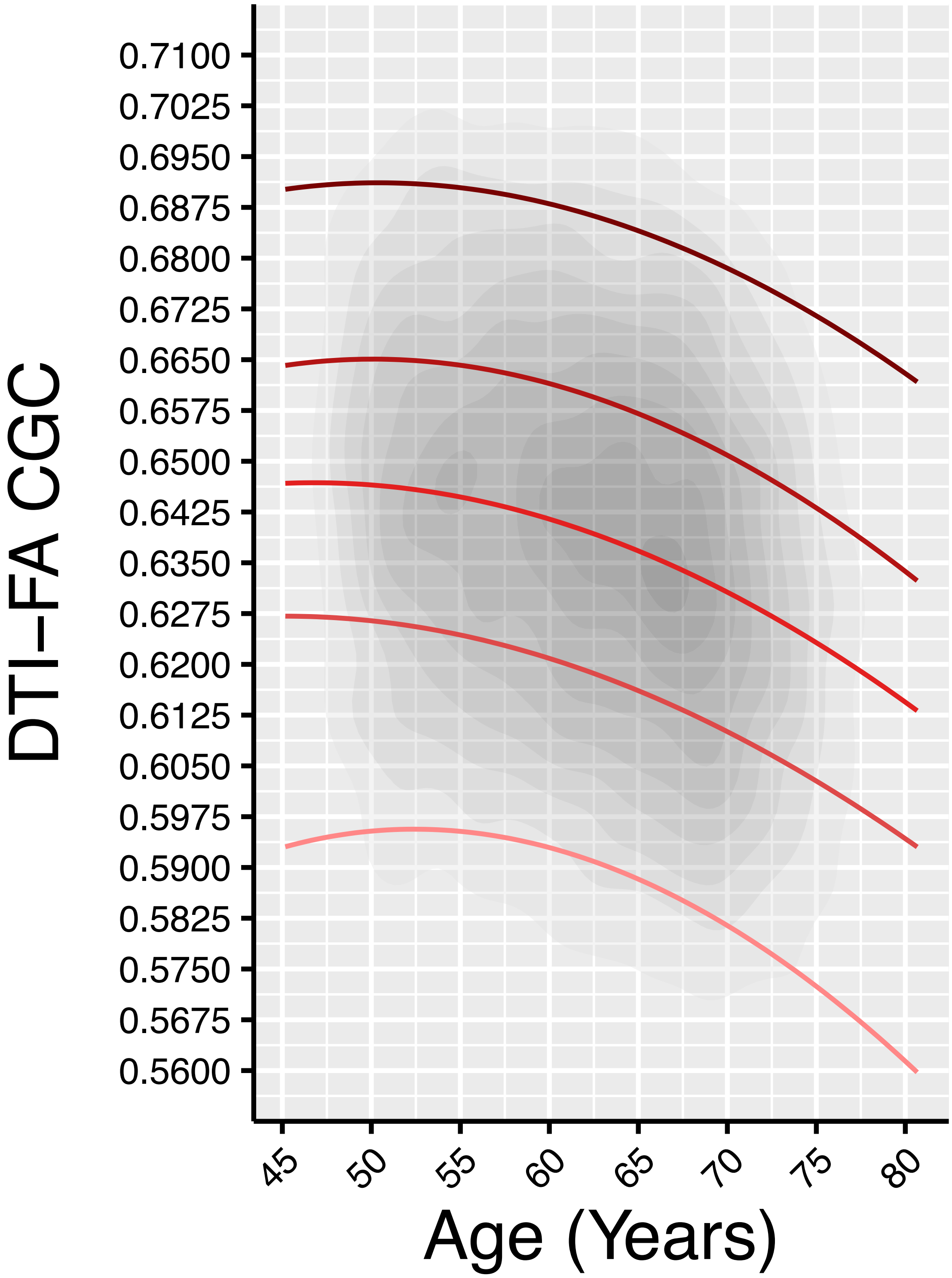

Figure S69. Full size normative centile reference curves calculated for the cingulum (cingulate) for DTI-FA in females. Solid colored lines, ordered from lightest to darkest, indicate the following centiles: 5th, 25th, 50th, 75th, 95th. Gray overlay reflects kernel density (darker=greater degree of data point overlap). CGC = cingulum (cingulate).

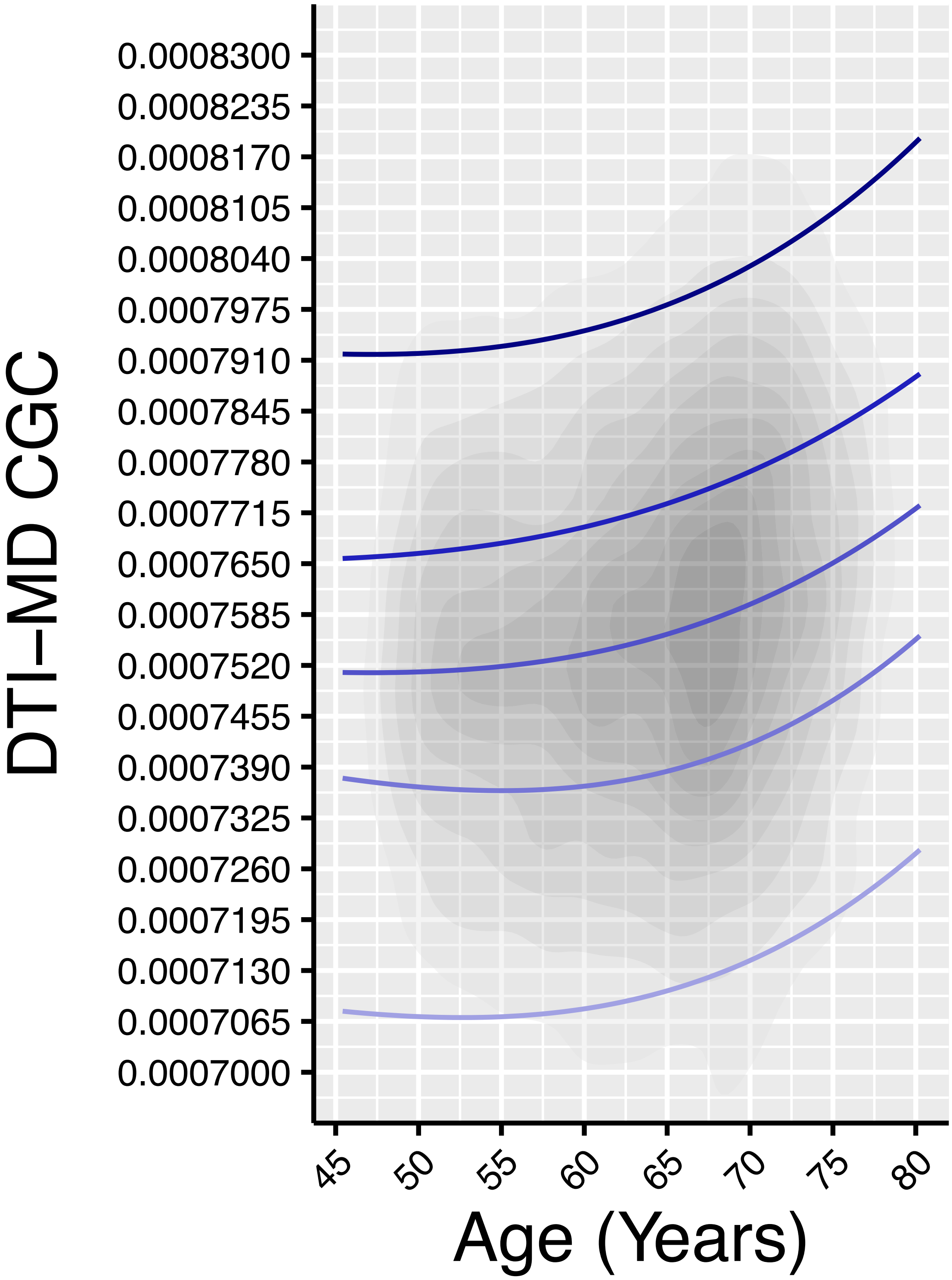

Figure S70. Full size normative centile reference curves calculated for the cingulum (cingulate) for DTI-MD in males. Solid colored lines, ordered from lightest to darkest, indicate the following centiles: 5th, 25th, 50th, 75th, 95th. Gray overlay reflects kernel density (darker=greater degree of data point overlap). CGC = cingulum (cingulate).

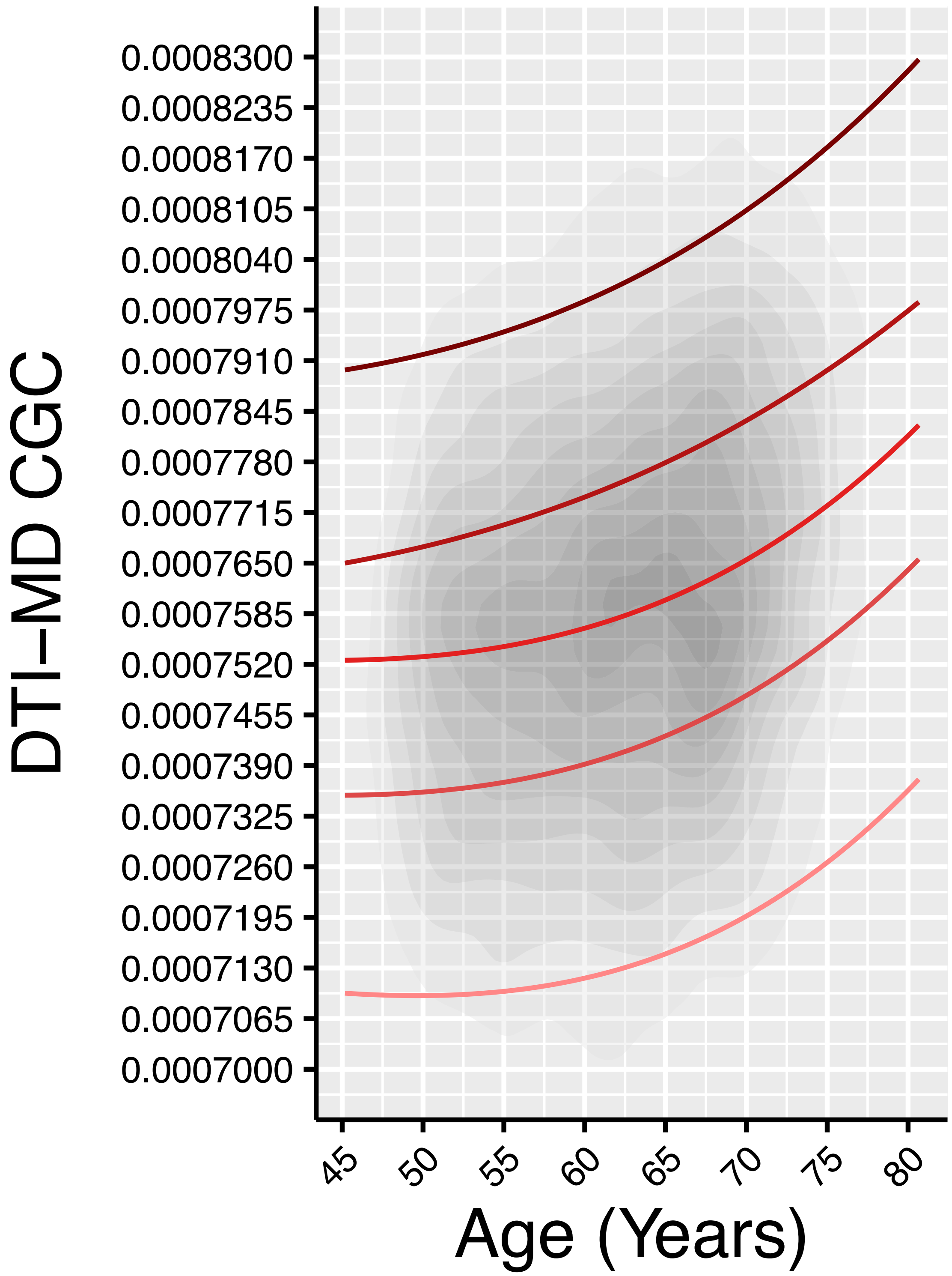

Figure S71. Full size normative centile reference curves calculated for the cingulum (cingulate) for DTI-MD in females. Solid colored lines, ordered from lightest to darkest, indicate the following centiles: 5th, 25th, 50th, 75th, 95th. Gray overlay reflects kernel density (darker=greater degree of data point overlap). CGC = cingulum (cingulate).

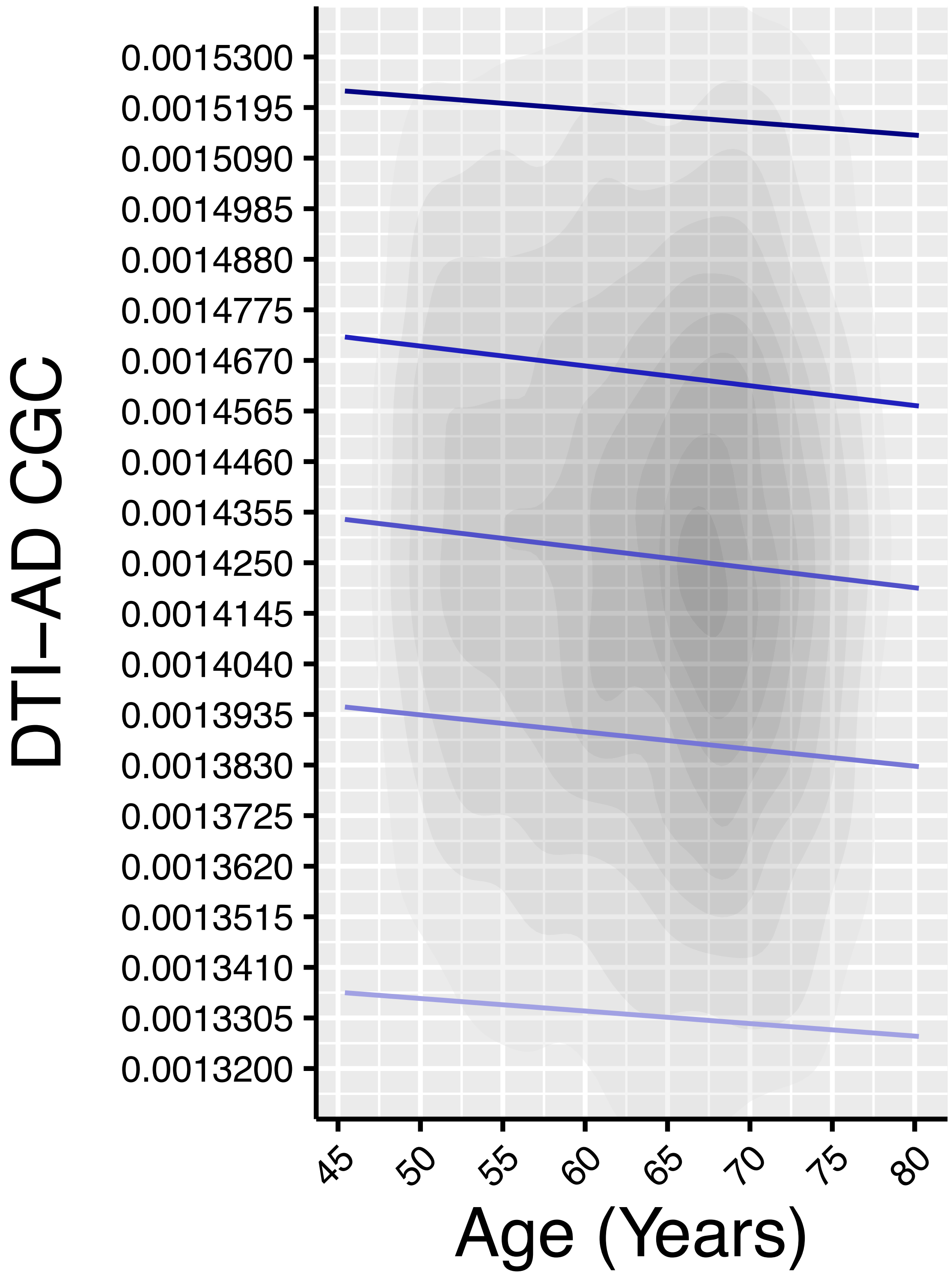

Figure S72. Full size normative centile reference curves calculated for the cingulum (cingulate) for DTI-AD in males. Solid colored lines, ordered from lightest to darkest, indicate the following centiles: 5th, 25th, 50th, 75th, 95th. Gray overlay reflects kernel density (darker=greater degree of data point overlap). CGC = cingulum (cingulate).

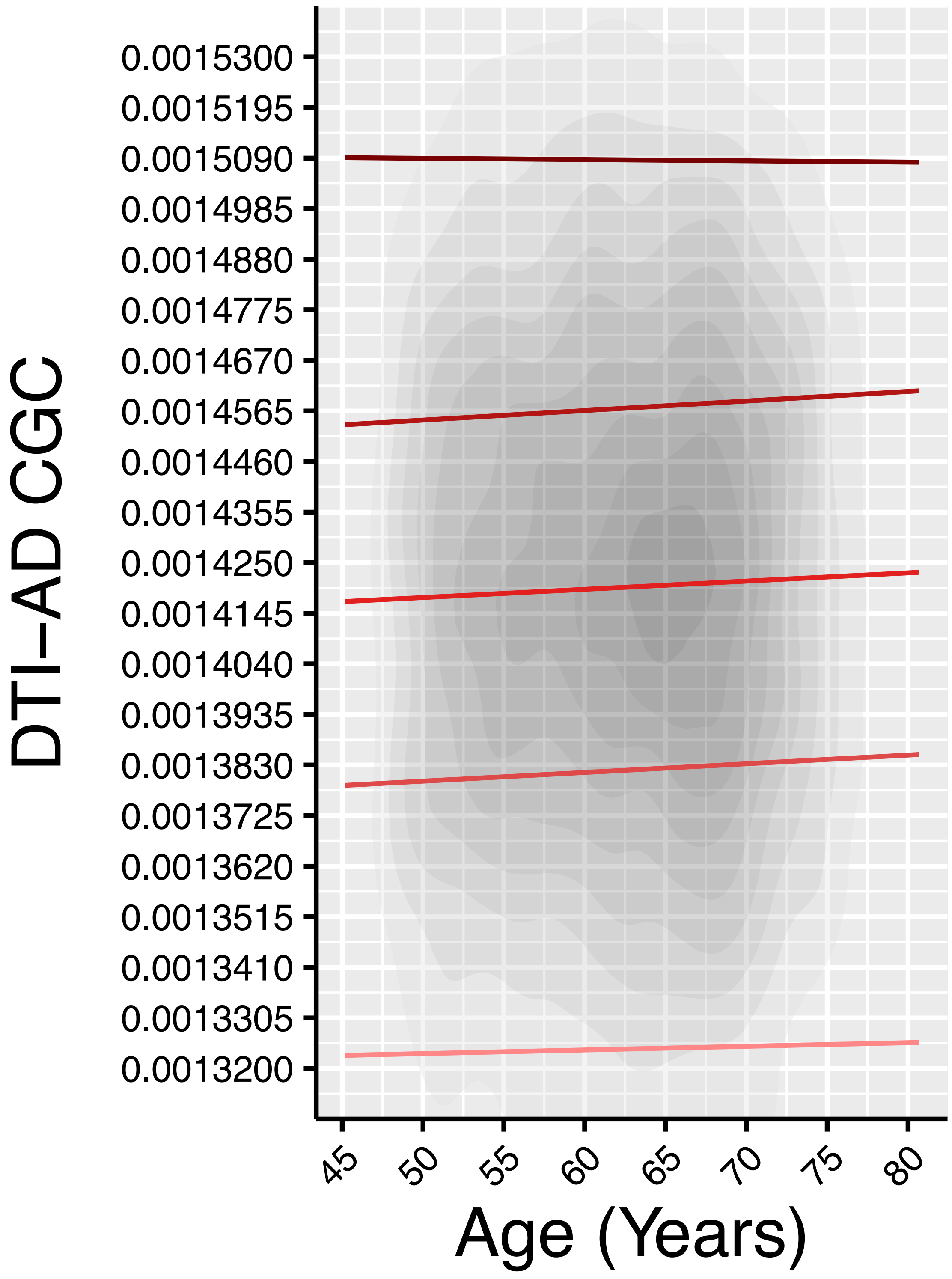

Figure S73. Full size normative centile reference curves calculated for the cingulum (cingulate) for DTI-AD in females. Solid colored lines, ordered from lightest to darkest, indicate the following centiles: 5th, 25th, 50th, 75th, 95th. Gray overlay reflects kernel density (darker=greater degree of data point overlap). CGC = cingulum (cingulate).

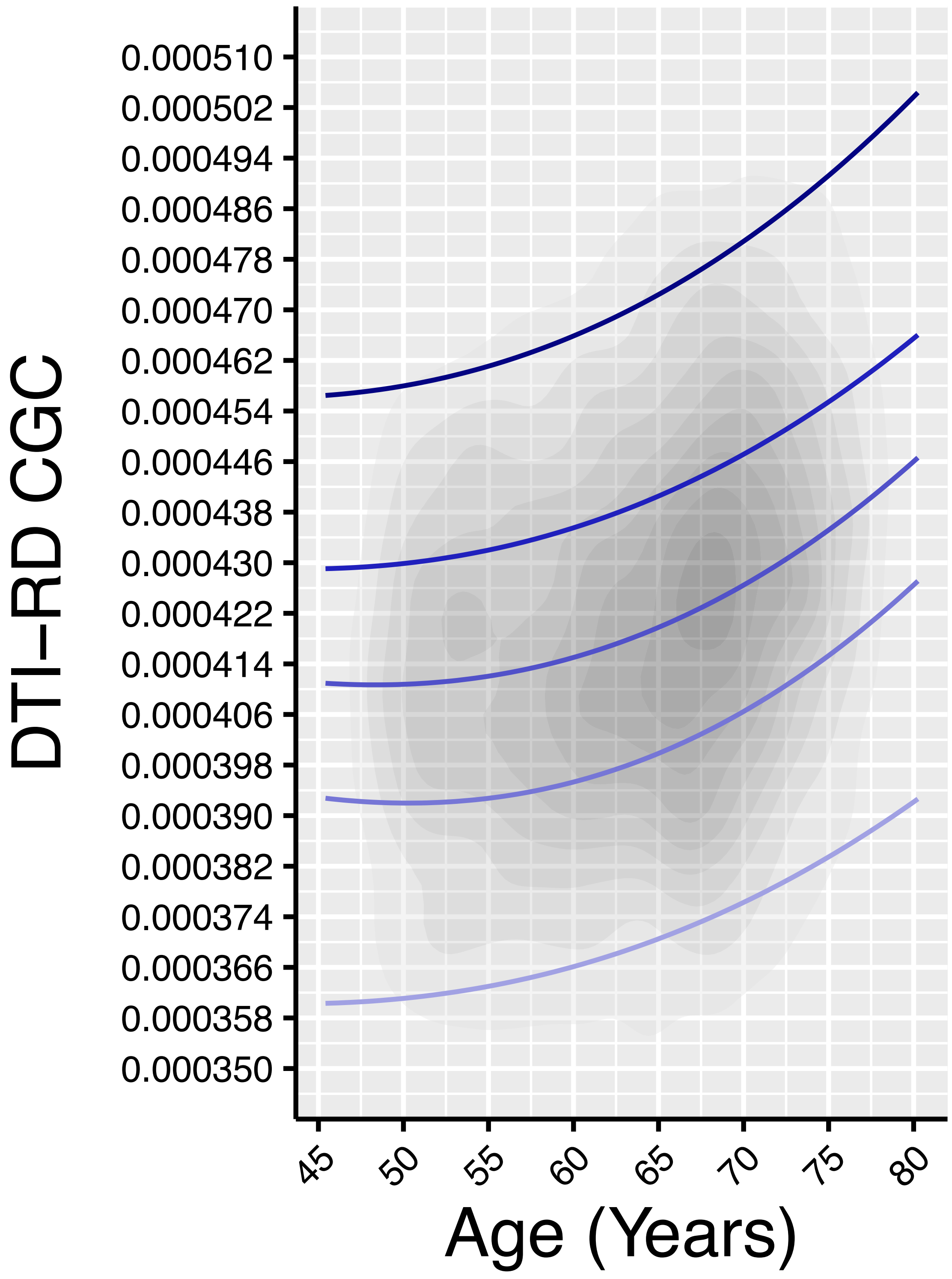

Figure S74. Full size normative centile reference curves calculated for the cingulum (cingulate) for DTI-RD in males. Solid colored lines, ordered from lightest to darkest, indicate the following centiles: 5th, 25th, 50th, 75th, 95th. Gray overlay reflects kernel density (darker=greater degree of data point overlap). CGC = cingulum (cingulate).

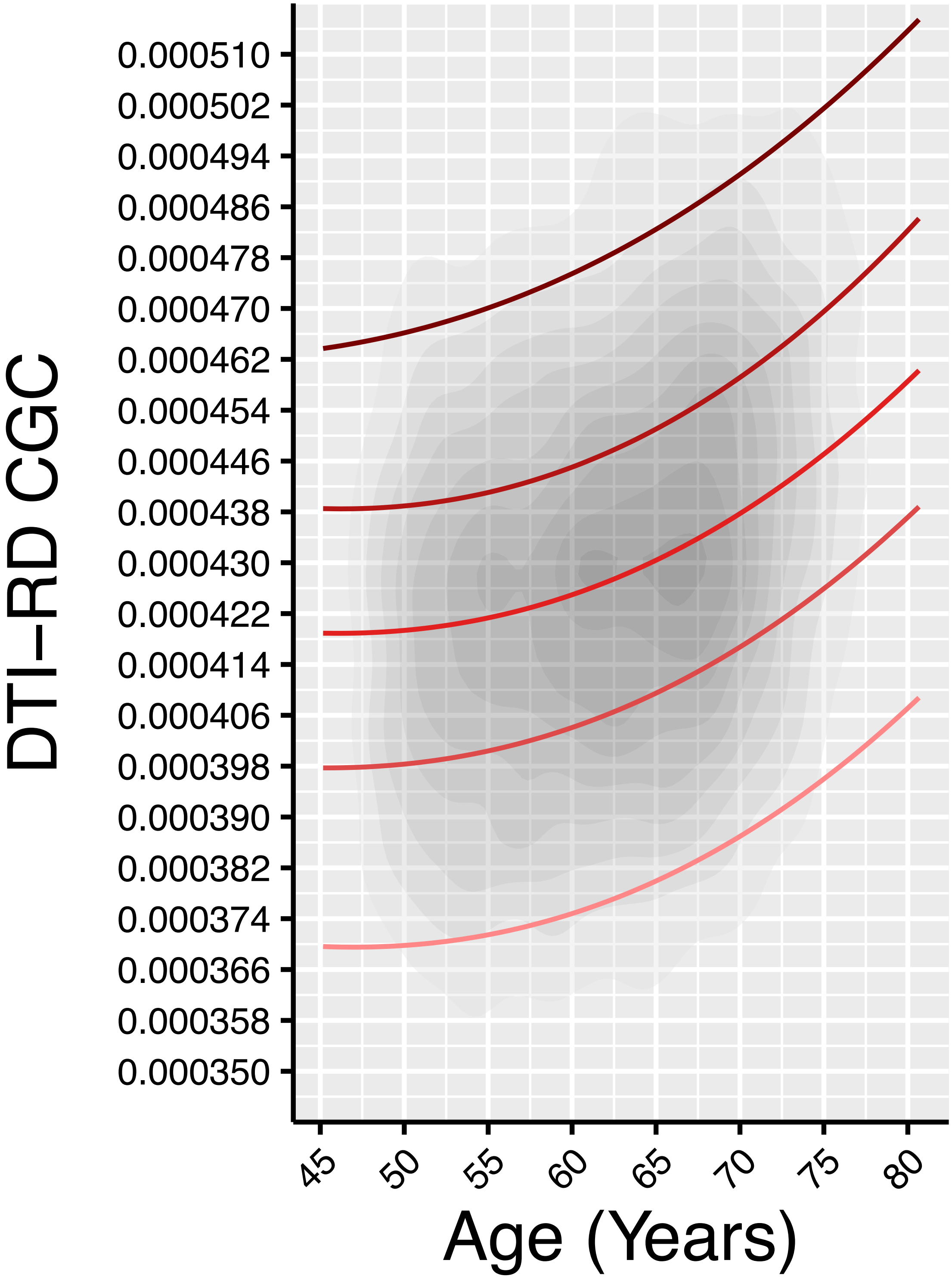

Figure S75. Full size normative centile reference curves calculated for the cingulum (cingulate) for DTI-RD in females. Solid colored lines, ordered from lightest to darkest, indicate the following centiles: 5th, 25th, 50th, 75th, 95th. Gray overlay reflects kernel density (darker=greater degree of data point overlap). CGC = cingulum (cingulate).

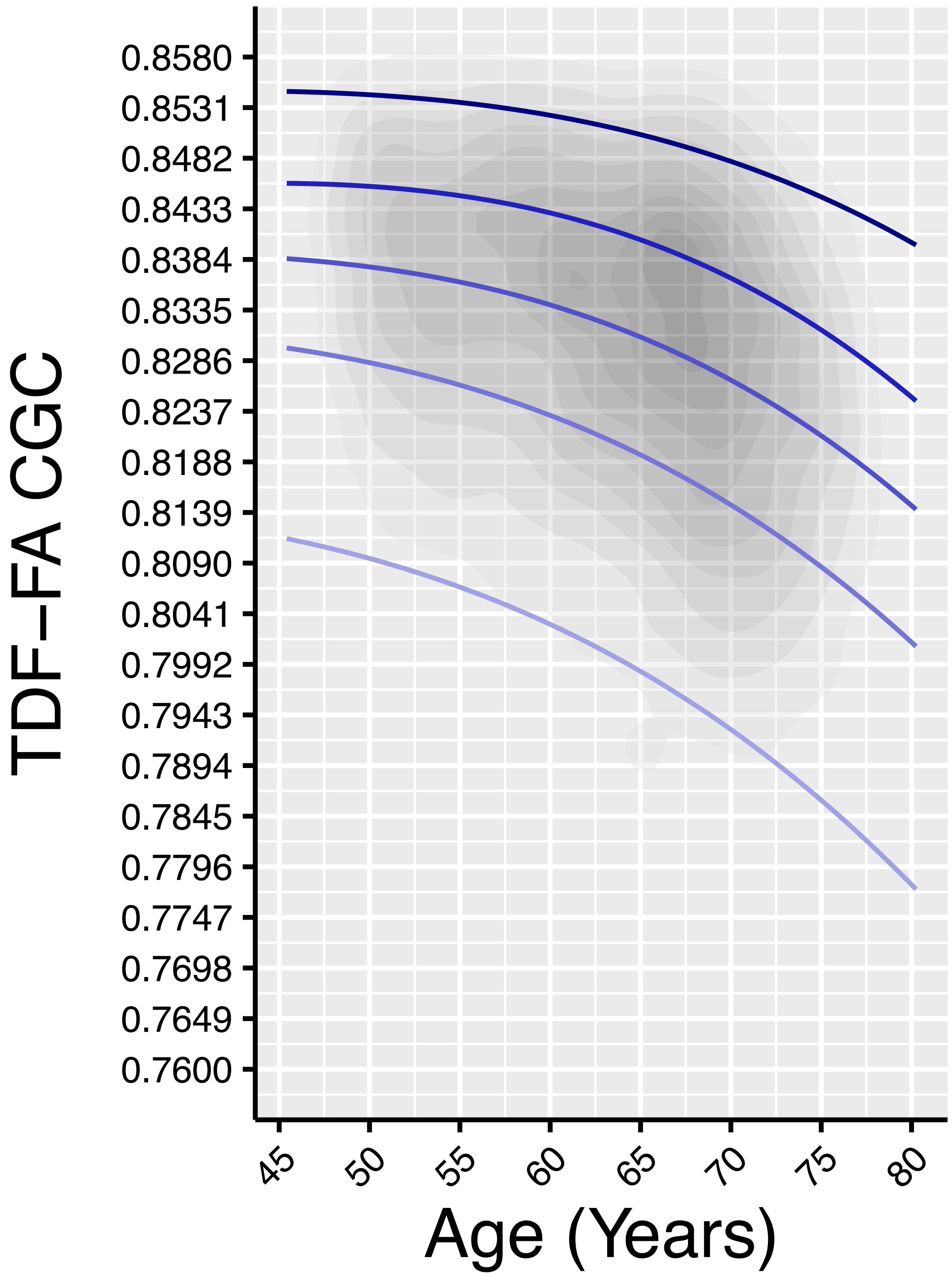

Figure S76. Full size normative centile reference curves calculated for the cingulum (cingulate) for TDF-FA in males. Solid colored lines, ordered from lightest to darkest, indicate the following centiles: 5th, 25th, 50th, 75th, 95th. Gray overlay reflects kernel density (darker=greater degree of data point overlap). CGC = cingulum (cingulate).

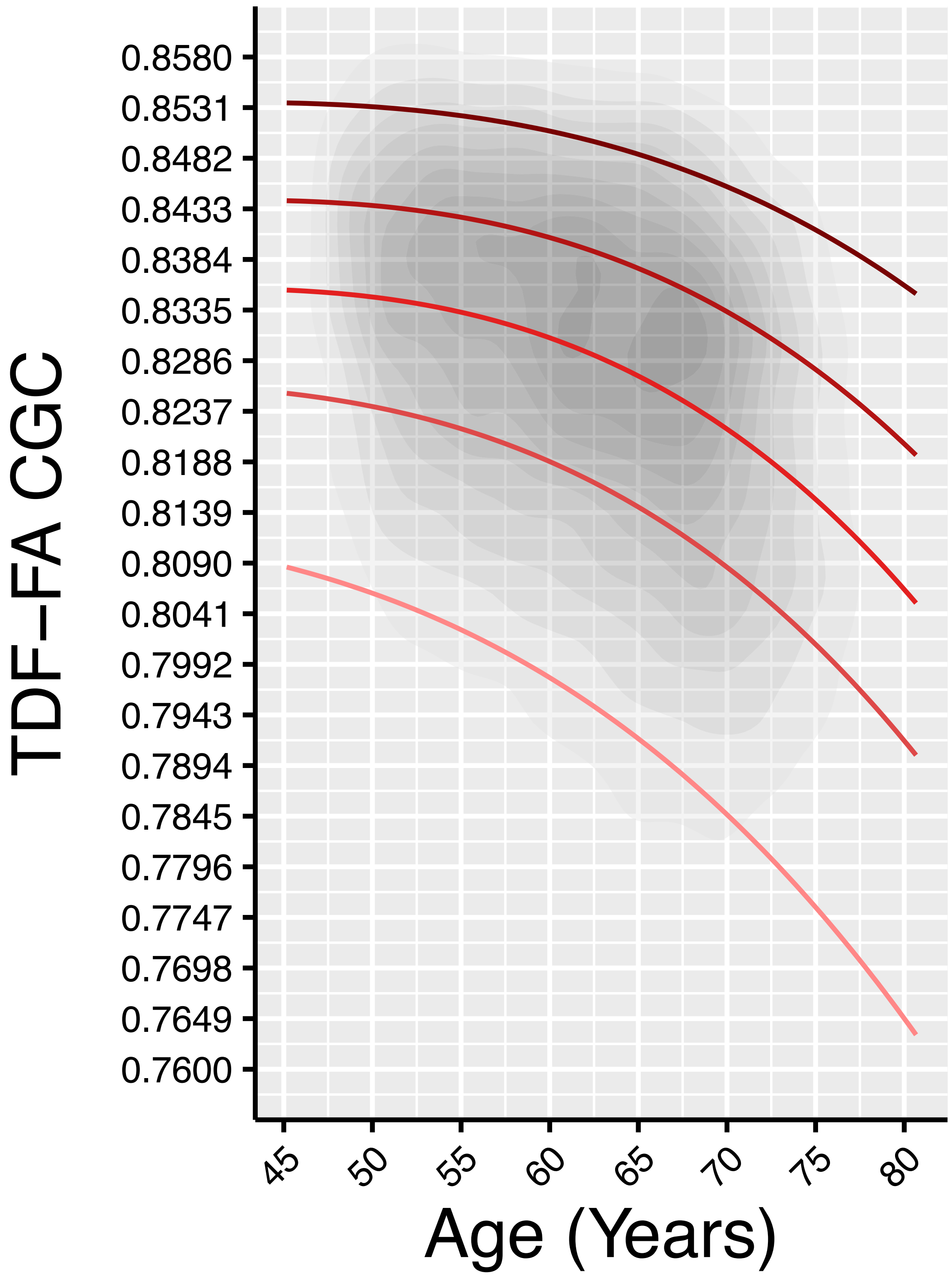

Figure S77. Full size normative centile reference curves calculated for the cingulum (cingulate) for TDF-FA in females. Solid colored lines, ordered from lightest to darkest, indicate the following centiles: 5th, 25th, 50th, 75th, 95th. Gray overlay reflects kernel density (darker=greater degree of data point overlap). CGC = cingulum (cingulate).

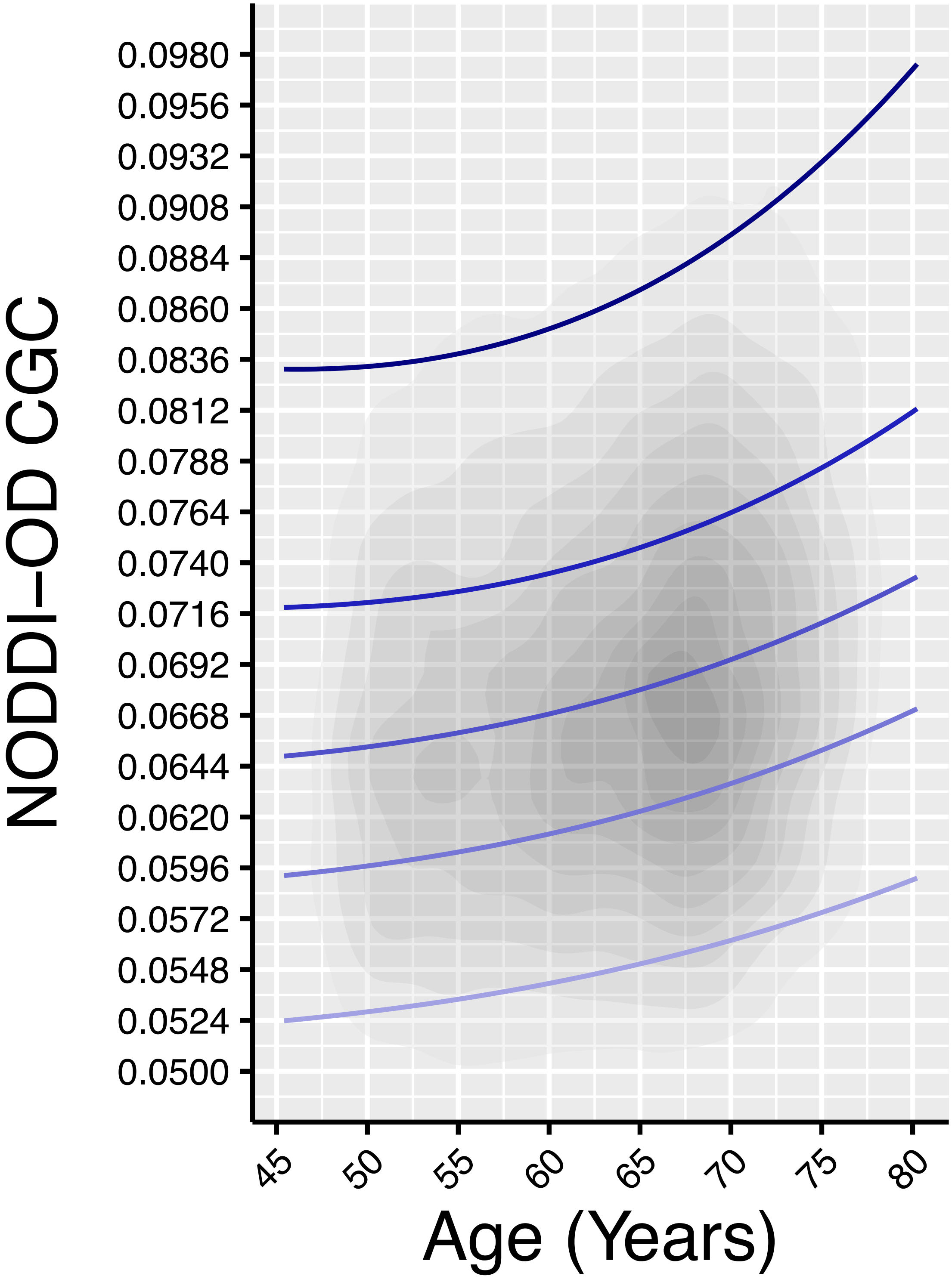

Figure S78. Full size normative centile reference curves calculated for the cingulum (cingulate) for NODDI-OD in males. Solid colored lines, ordered from lightest to darkest, indicate the following centiles: 5th, 25th, 50th, 75th, 95th. Gray overlay reflects kernel density (darker=greater degree of data point overlap). CGC = cingulum (cingulate).

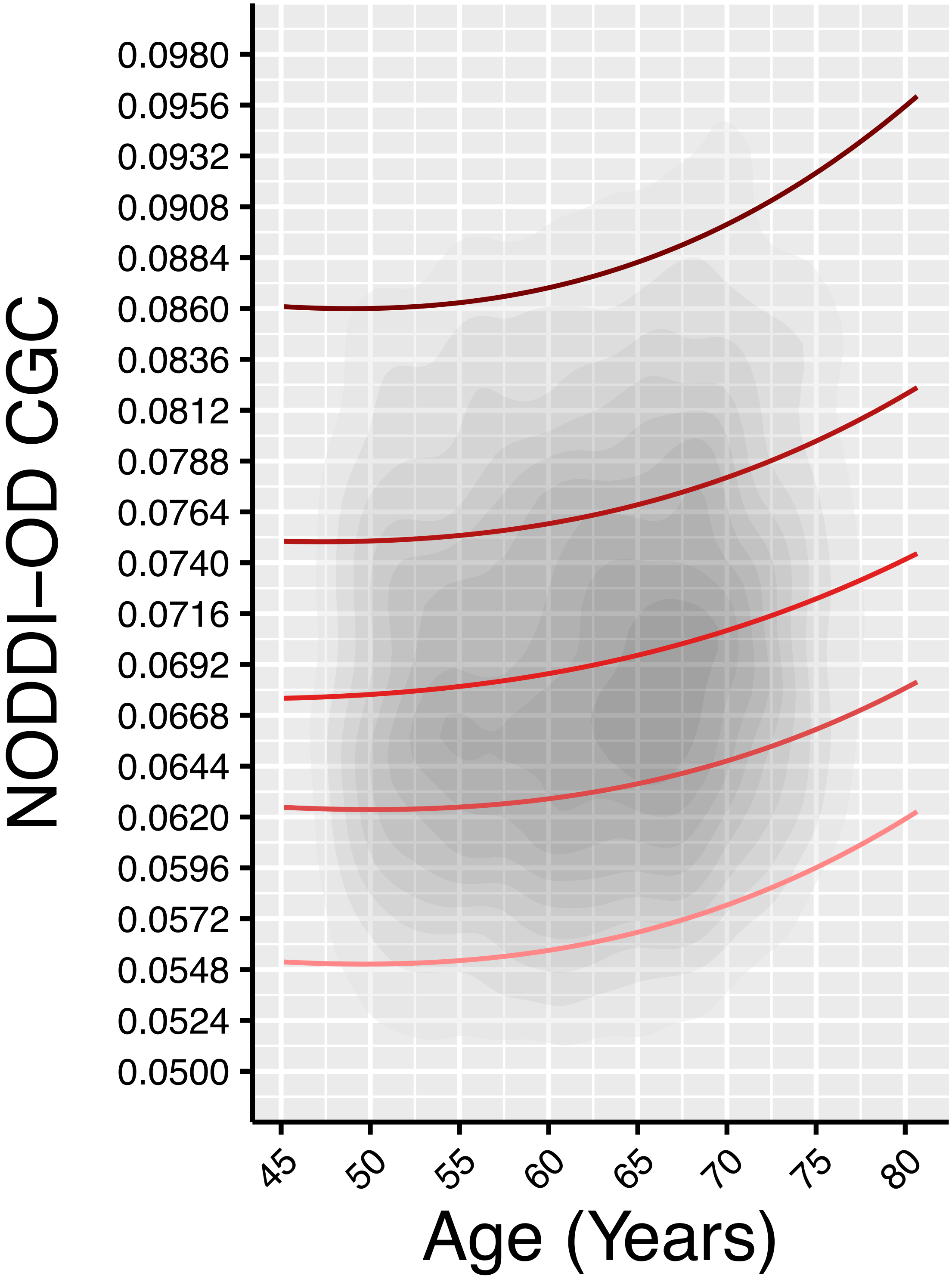

Figure S79. Full size normative centile reference curves calculated for the cingulum (cingulate) for NODDI-OD in females. Solid colored lines, ordered from lightest to darkest, indicate the following centiles: 5th, 25th, 50th, 75th, 95th. Gray overlay reflects kernel density (darker=greater degree of data point overlap). CGC = cingulum (cingulate).

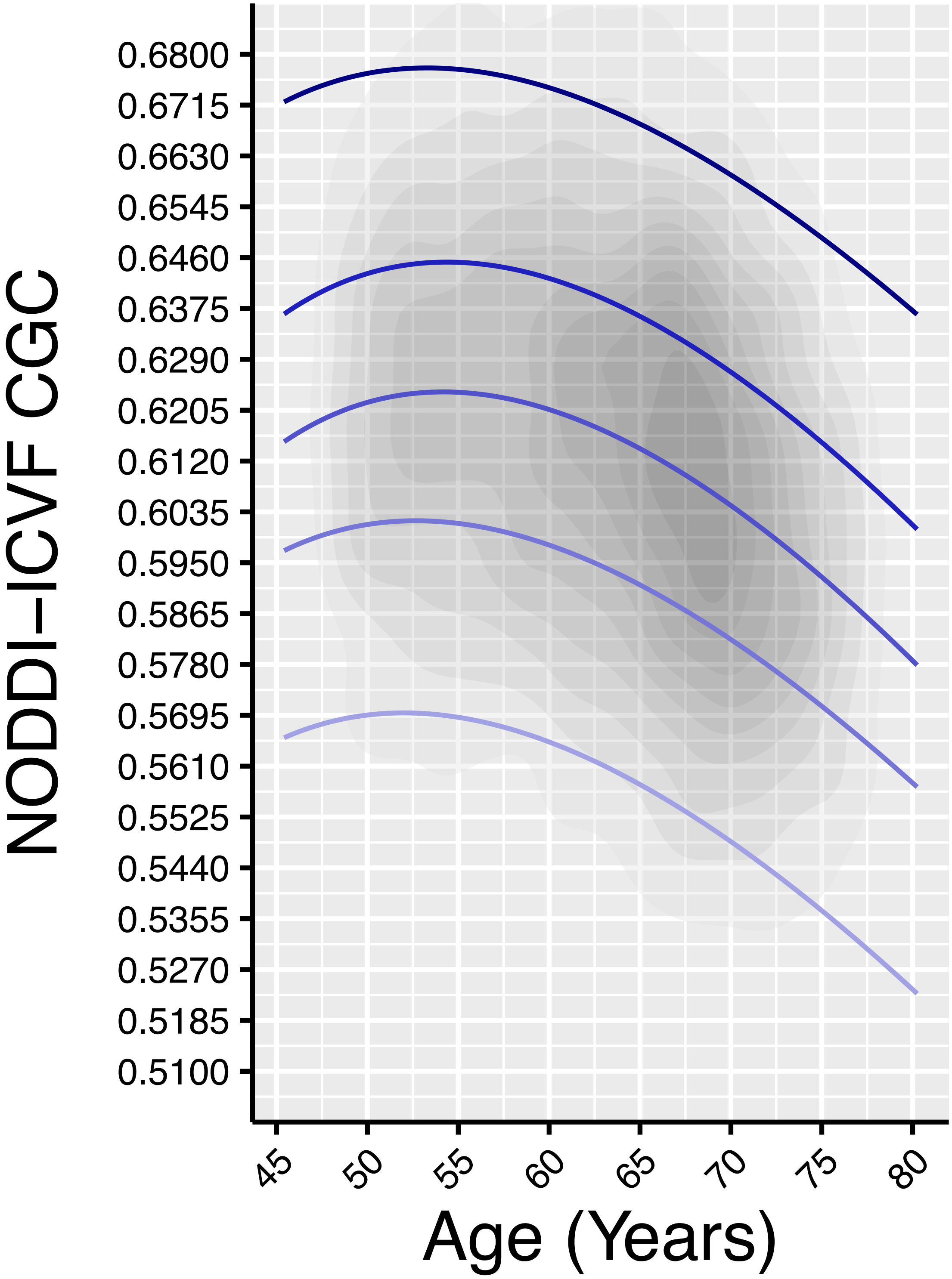

Figure S80. Full size normative centile reference curves calculated for the cingulum (cingulate) for NODDI-ICVF in males. Solid colored lines, ordered from lightest to darkest, indicate the following centiles: 5th, 25th, 50th, 75th, 95th. Gray overlay reflects kernel density (darker=greater degree of data point overlap). CGC = cingulum (cingulate).

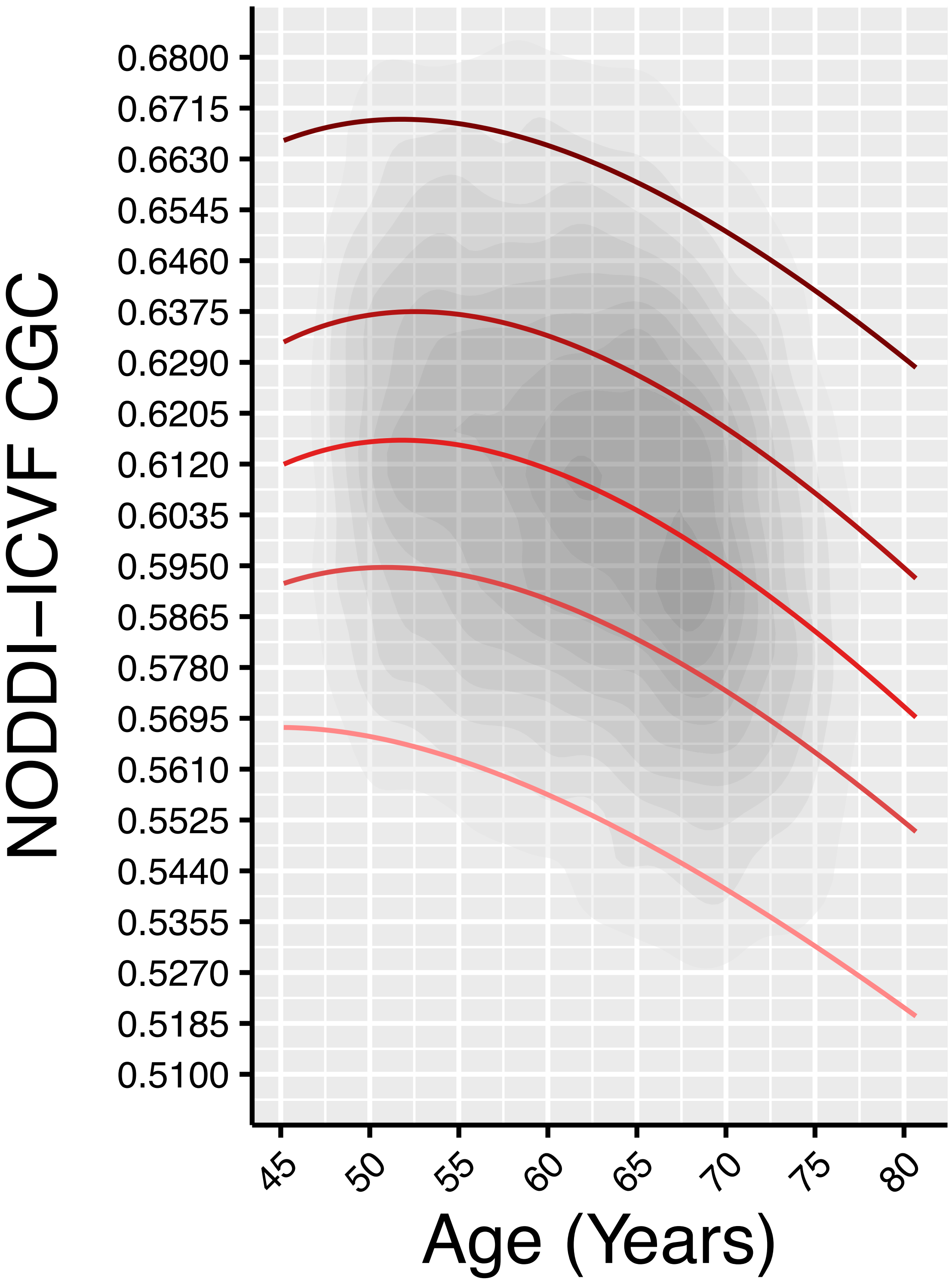

Figure S81. Full size normative centile reference curves calculated for the cingulum (cingulate) for NODDI-ICVF in females. Solid colored lines, ordered from lightest to darkest, indicate the following centiles: 5th, 25th, 50th, 75th, 95th. Gray overlay reflects kernel density (darker=greater degree of data point overlap). CGC = cingulum (cingulate).

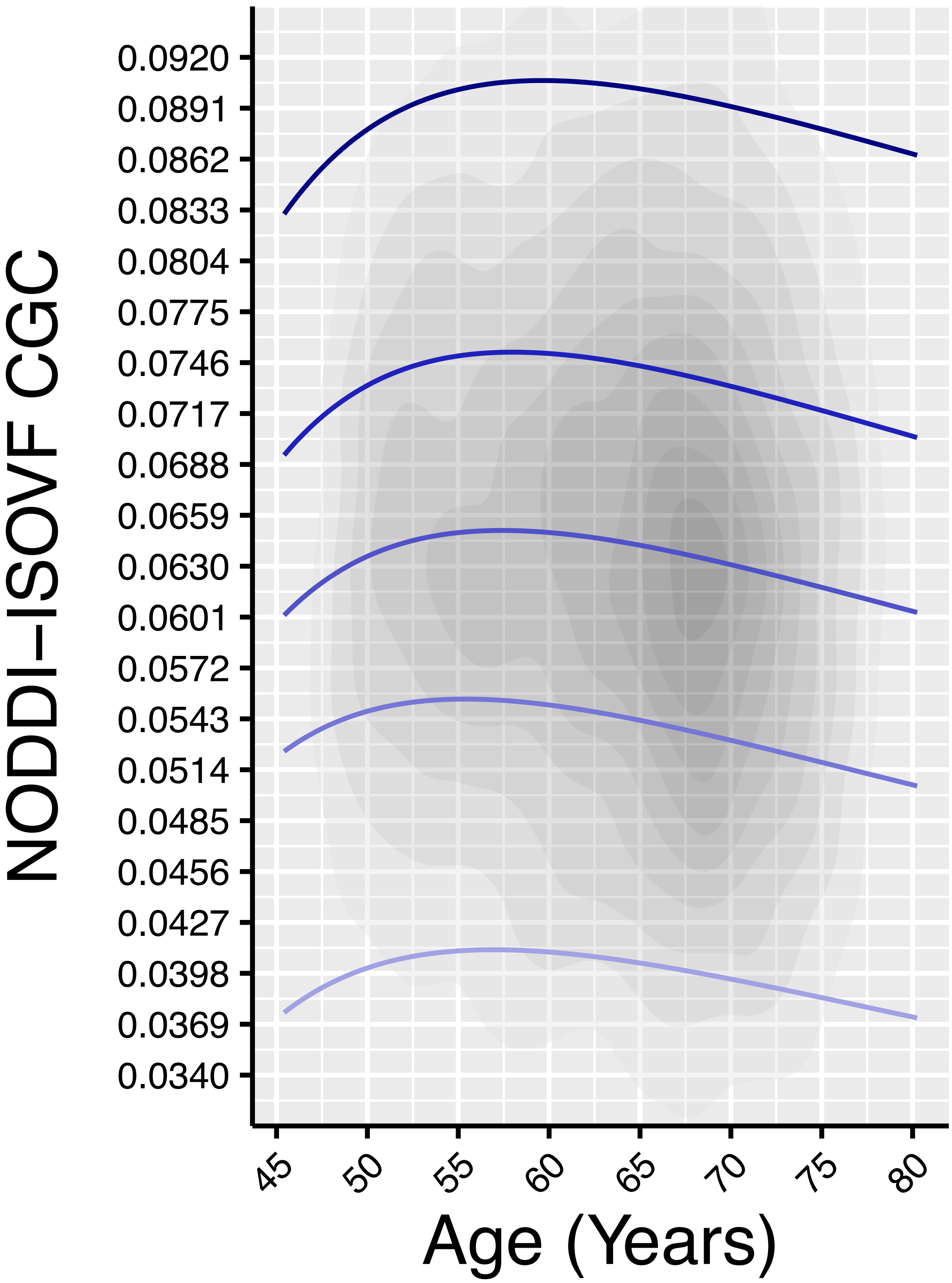

Figure S82. Full size normative centile reference curves calculated for the cingulum (cingulate) for NODDI-ISOVF in males. Solid colored lines, ordered from lightest to darkest, indicate the following centiles: 5th, 25th, 50th, 75th, 95th. Gray overlay reflects kernel density (darker=greater degree of data point overlap). CGC = cingulum (cingulate).

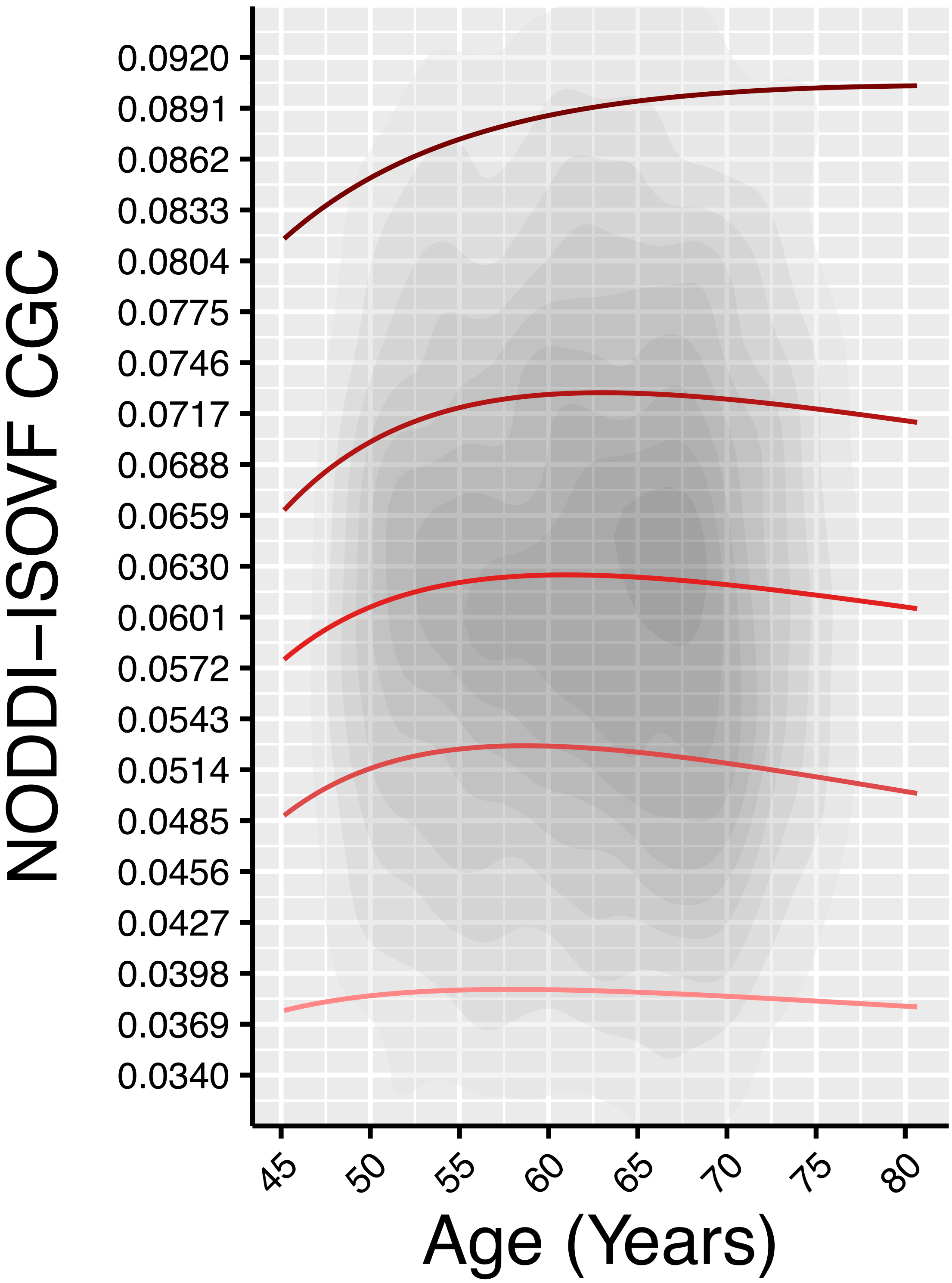

Figure S83. Full size normative centile reference curves calculated for the cingulum (cingulate) for NODDI-ISOVF in females. Solid colored lines, ordered from lightest to darkest, indicate the following centiles: 5th, 25th, 50th, 75th, 95th. Gray overlay reflects kernel density (darker=greater degree of data point overlap). CGC = cingulum (cingulate).

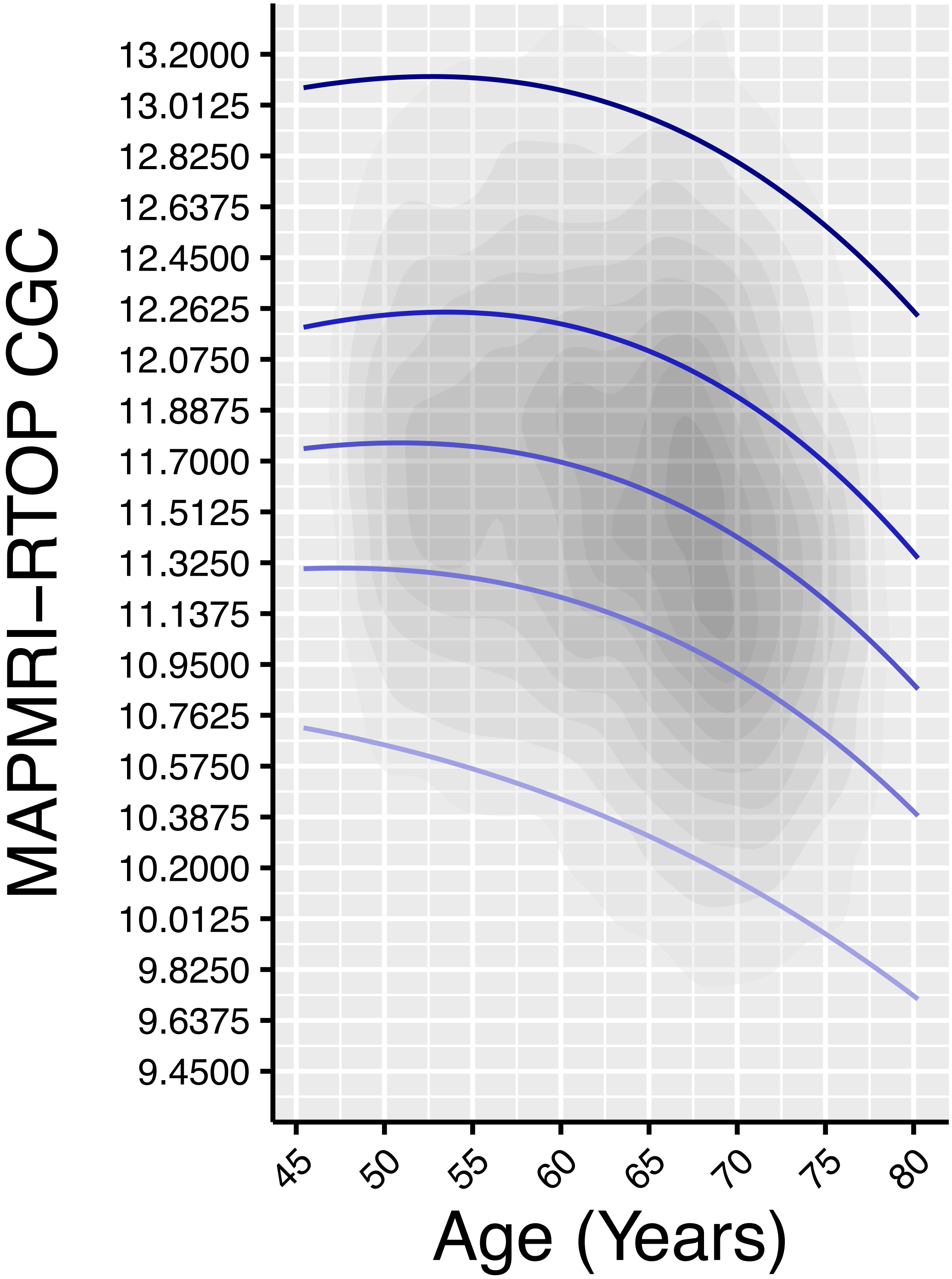

Figure S84. Full size normative centile reference curves calculated for the cingulum (cingulate) for MAPMRI-RTOP in males. Solid colored lines, ordered from lightest to darkest, indicate the following centiles: 5th, 25th, 50th, 75th, 95th. Gray overlay reflects kernel density (darker=greater degree of data point overlap). CGC = cingulum (cingulate).

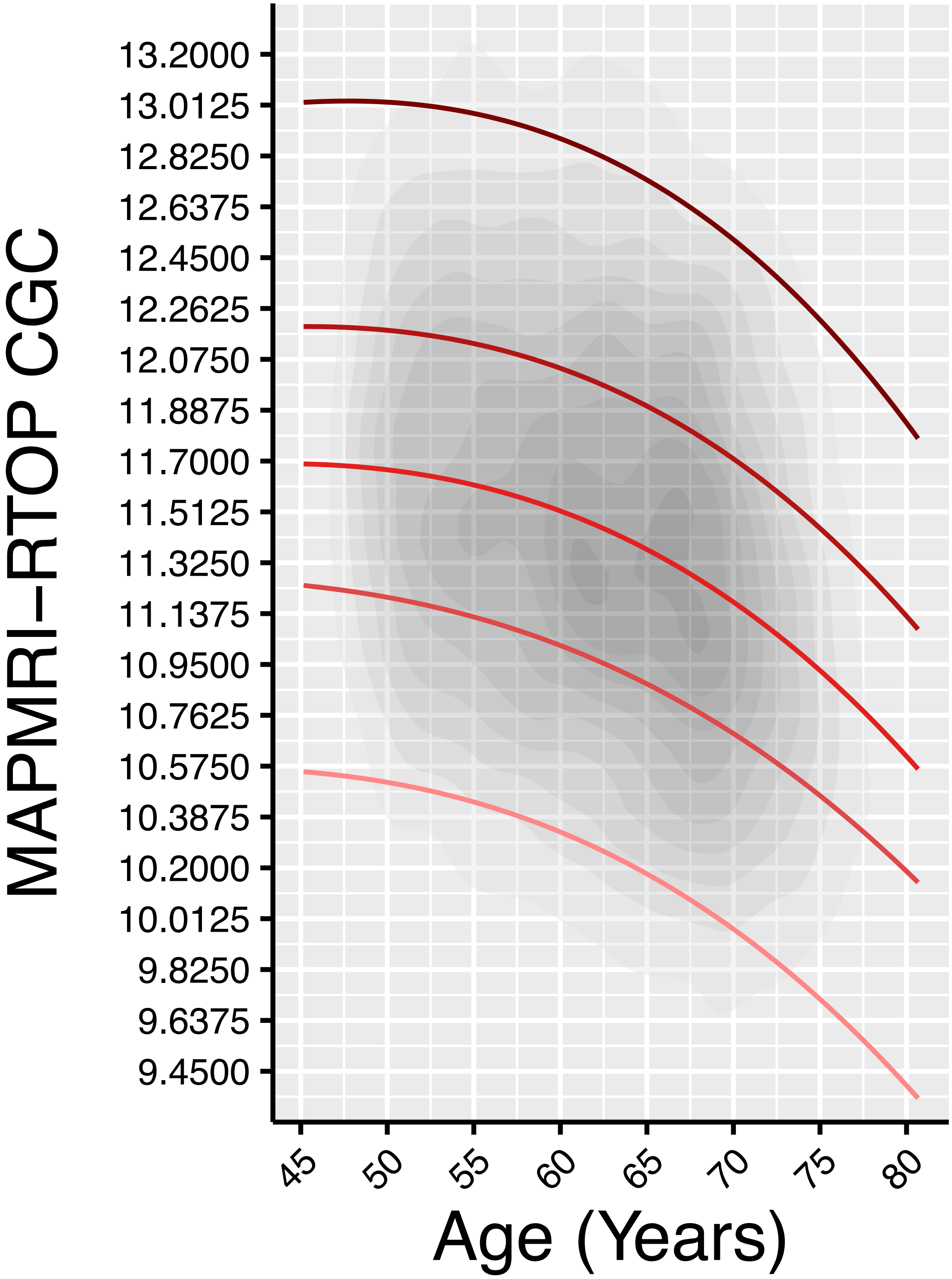

Figure S85. Full size normative centile reference curves calculated for the cingulum (cingulate) for MAPMRI-RTOP in females. Solid colored lines, ordered from lightest to darkest, indicate the following centiles: 5th, 25th, 50th, 75th, 95th. Gray overlay reflects kernel density (darker=greater degree of data point overlap). CGC = cingulum (cingulate).

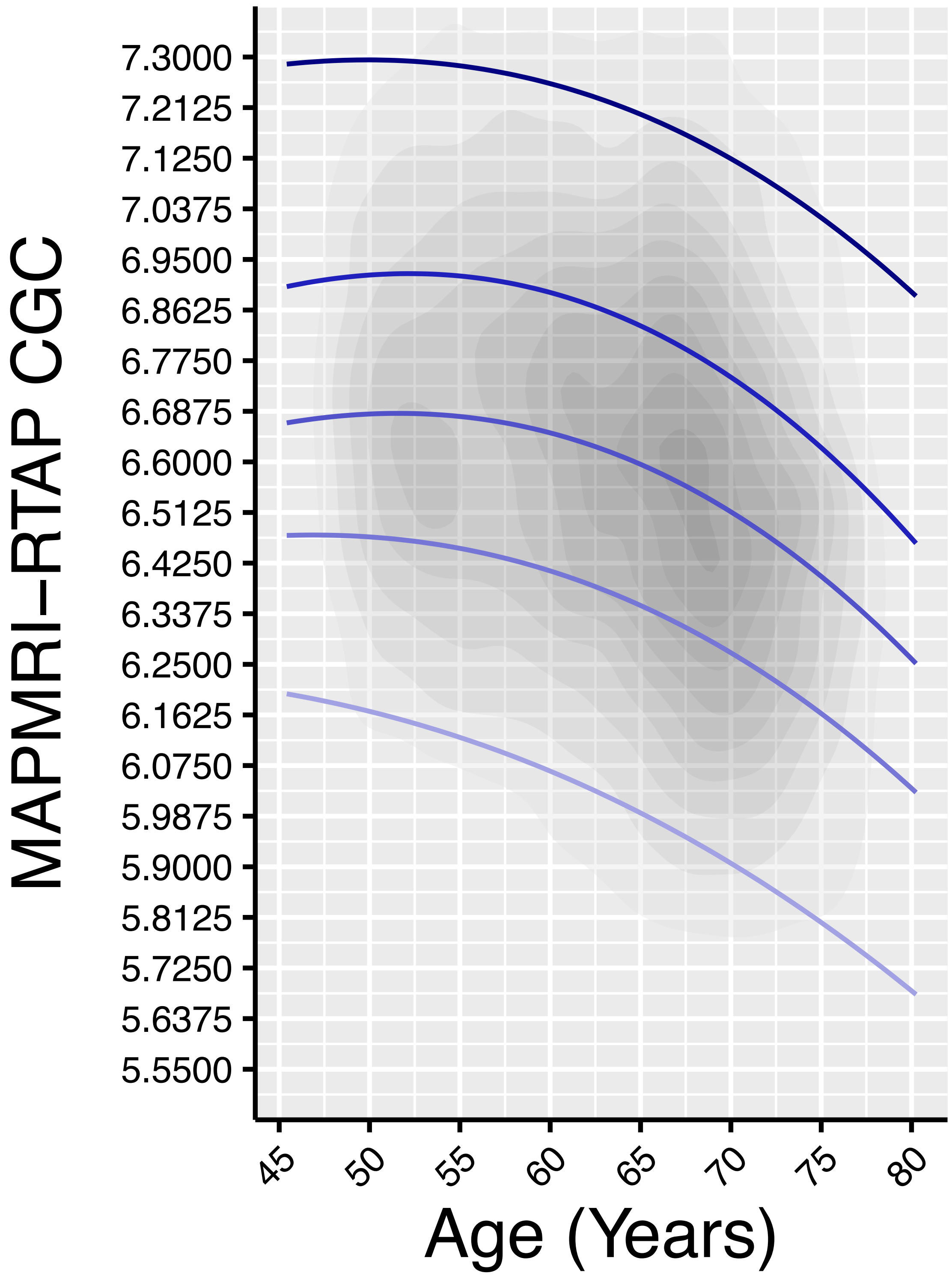

Figure S86. Full size normative centile reference curves calculated for the cingulum (cingulate) for MAPMRI-RTAP in males. Solid colored lines, ordered from lightest to darkest, indicate the following centiles: 5th, 25th, 50th, 75th, 95th. Gray overlay reflects kernel density (darker=greater degree of data point overlap). CGC = cingulum (cingulate).

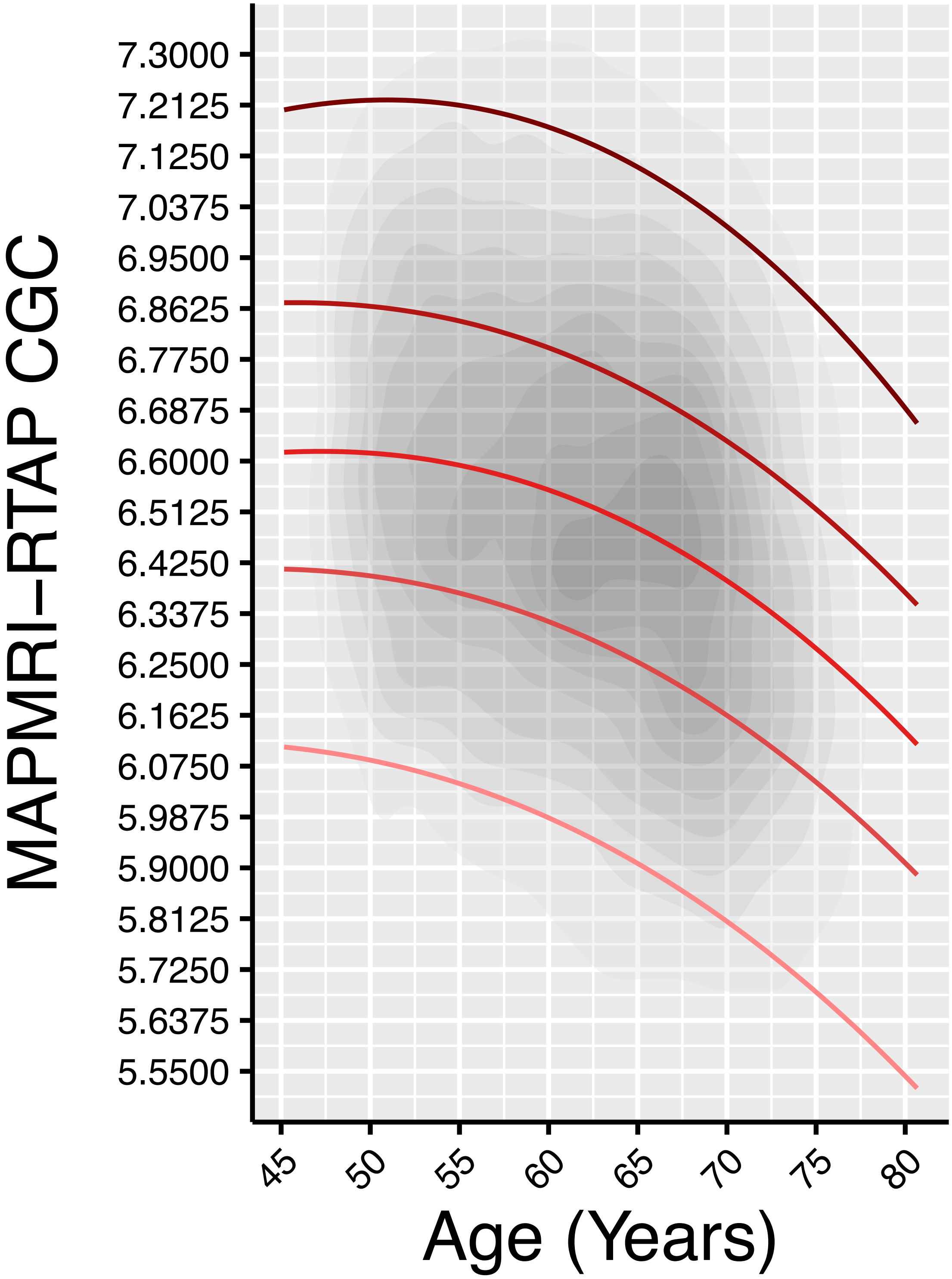

Figure S87. Full size normative centile reference curves calculated for the cingulum (cingulate) for MAPMRI-RTAP in females. Solid colored lines, ordered from lightest to darkest, indicate the following centiles: 5th, 25th, 50th, 75th, 95th. Gray overlay reflects kernel density (darker=greater degree of data point overlap). CGC = cingulum (cingulate).

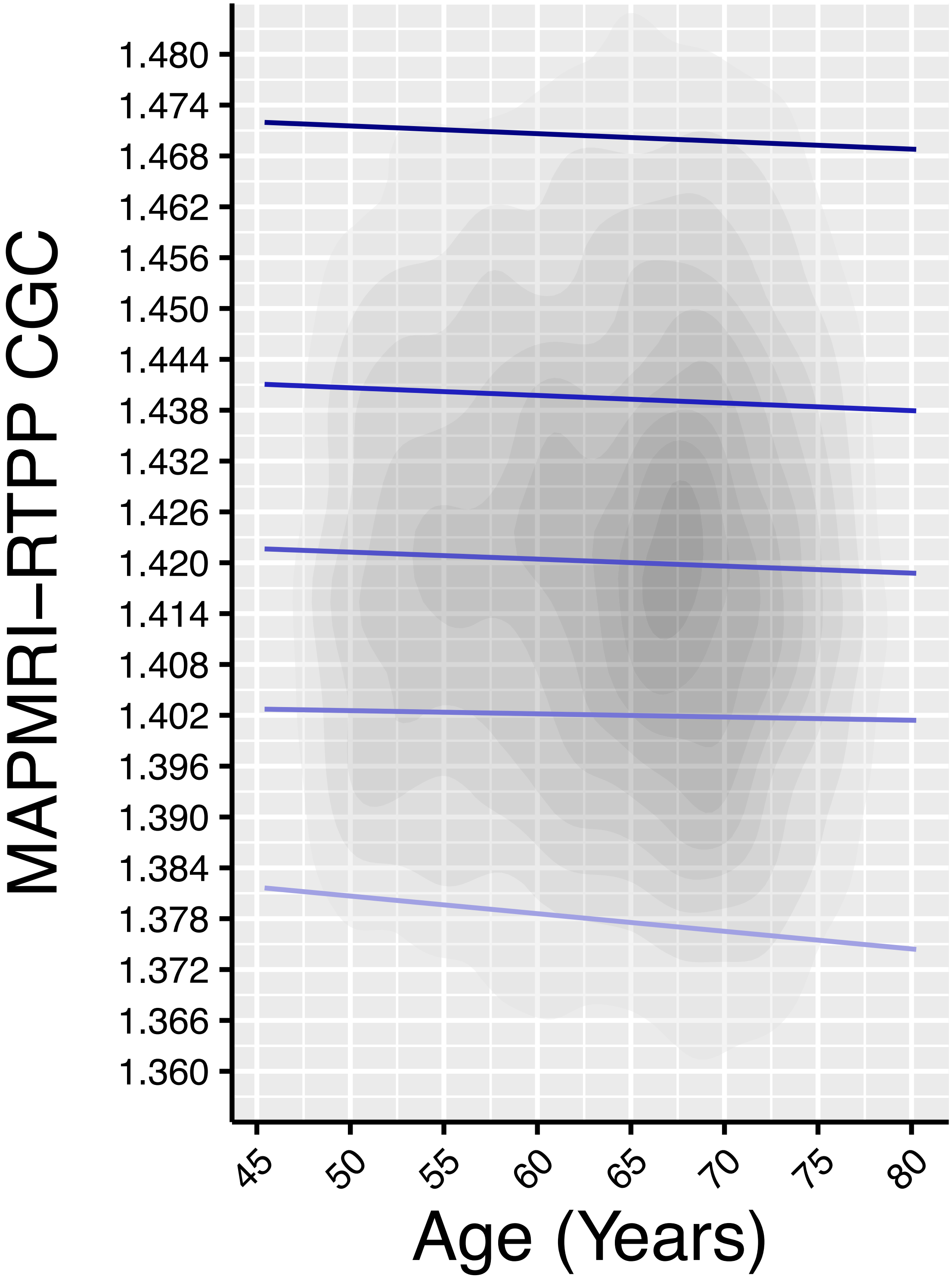

Figure S88. Full size normative centile reference curves calculated for the cingulum (cingulate) for MAPMRI-RTPP in males. Solid colored lines, ordered from lightest to darkest, indicate the following centiles: 5th, 25th, 50th, 75th, 95th. Gray overlay reflects kernel density (darker=greater degree of data point overlap). CGC = cingulum (cingulate).

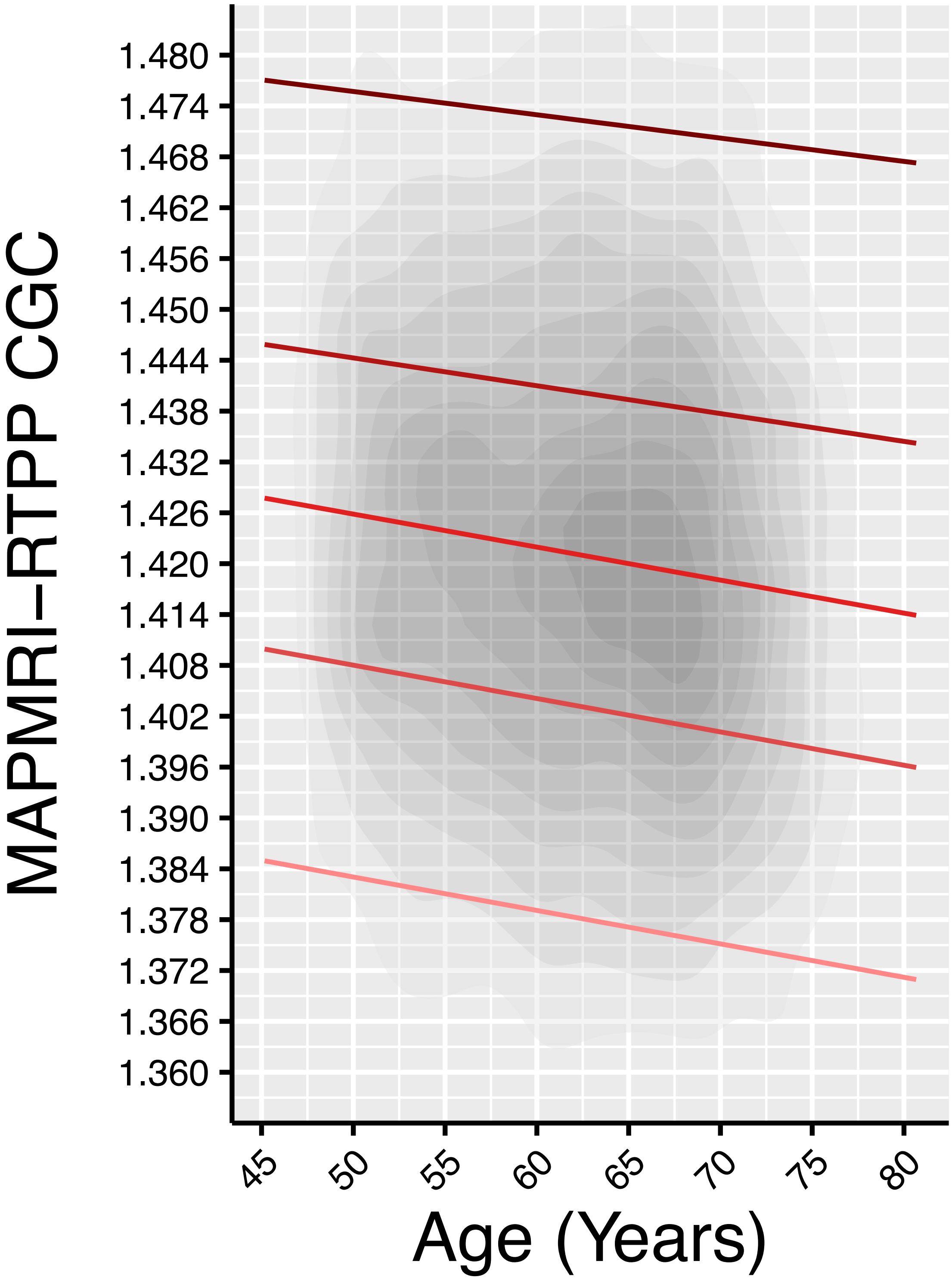

Figure S89. Full size normative centile reference curves calculated for the cingulum (cingulate) for MAPMRI-RTPP in females. Solid colored lines, ordered from lightest to darkest, indicate the following centiles: 5th, 25th, 50th, 75th, 95th. Gray overlay reflects kernel density (darker=greater degree of data point overlap). CGC = cingulum (cingulate).

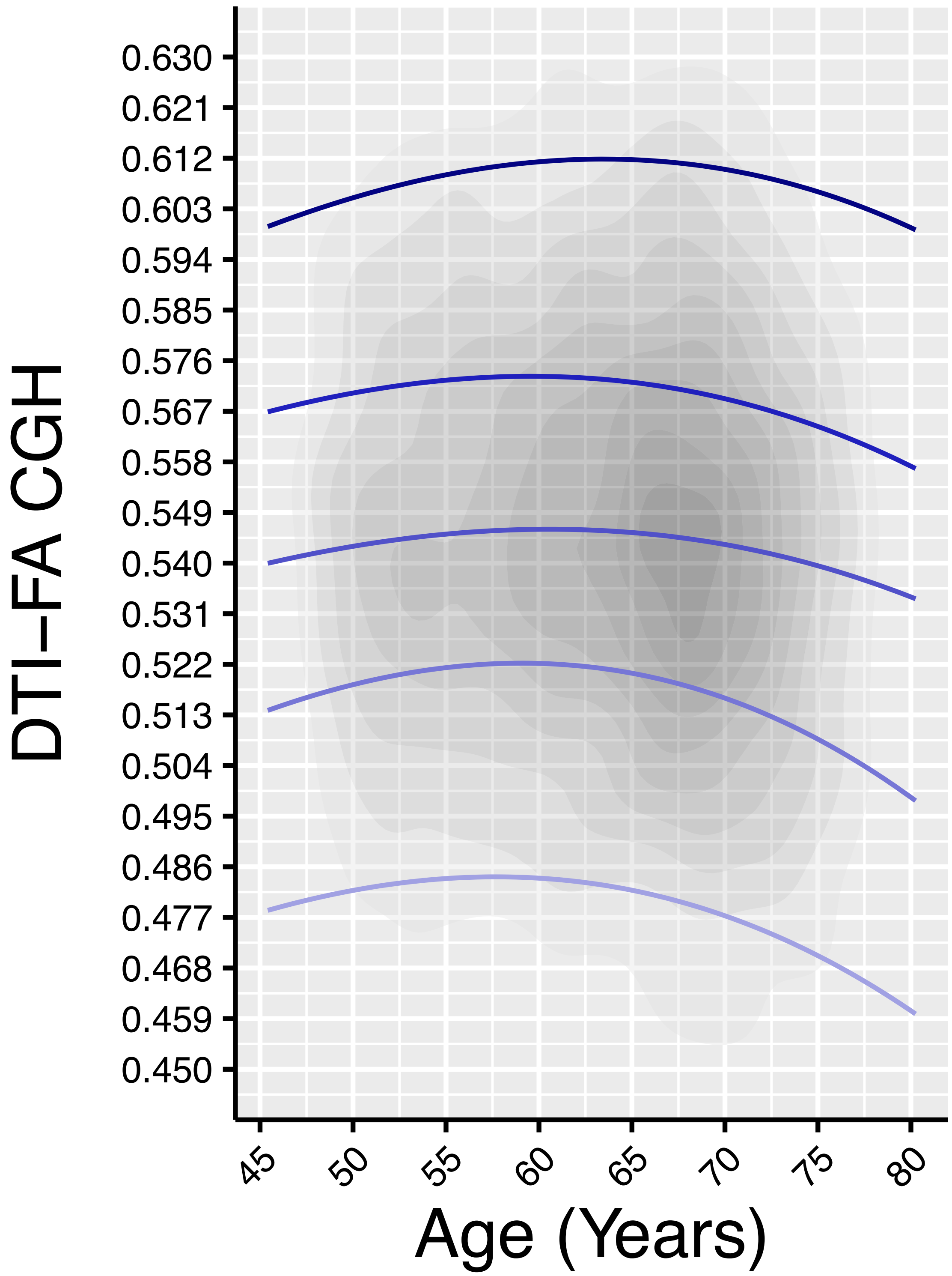

Figure S90. Full size normative centile reference curves calculated for the cingulum (hippocampal) for DTI-FA in males. Solid colored lines, ordered from lightest to darkest, indicate the following centiles: 5th, 25th, 50th, 75th, 95th. Gray overlay reflects kernel density (darker=greater degree of data point overlap). CGH = cingulum (hippocampal).

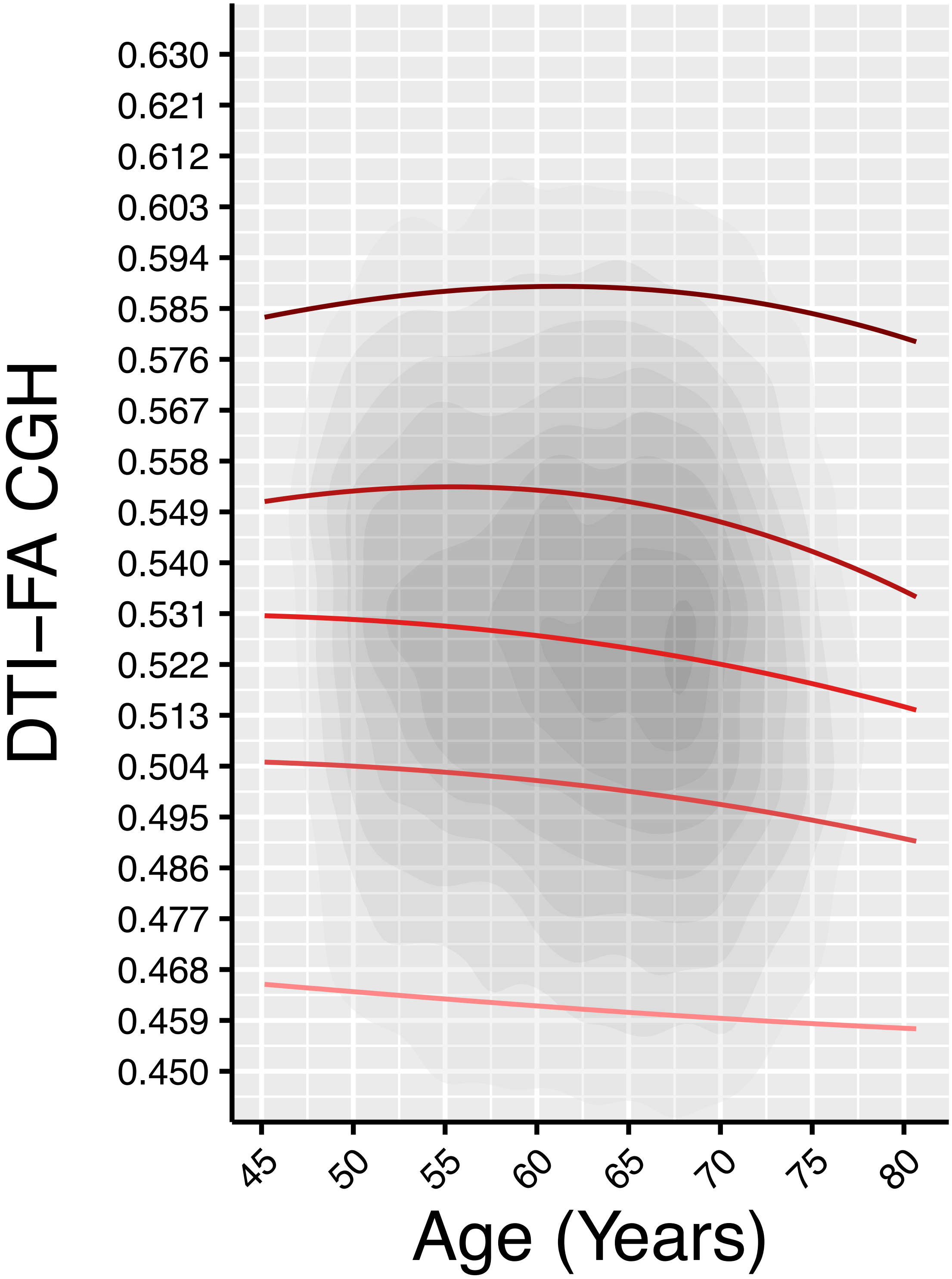

Figure S91. Full size normative centile reference curves calculated for the cingulum (hippocampal) for DTI-FA in females. Solid colored lines, ordered from lightest to darkest, indicate the following centiles: 5th, 25th, 50th, 75th, 95th. Gray overlay reflects kernel density (darker=greater degree of data point overlap). CGH = cingulum (hippocampal).

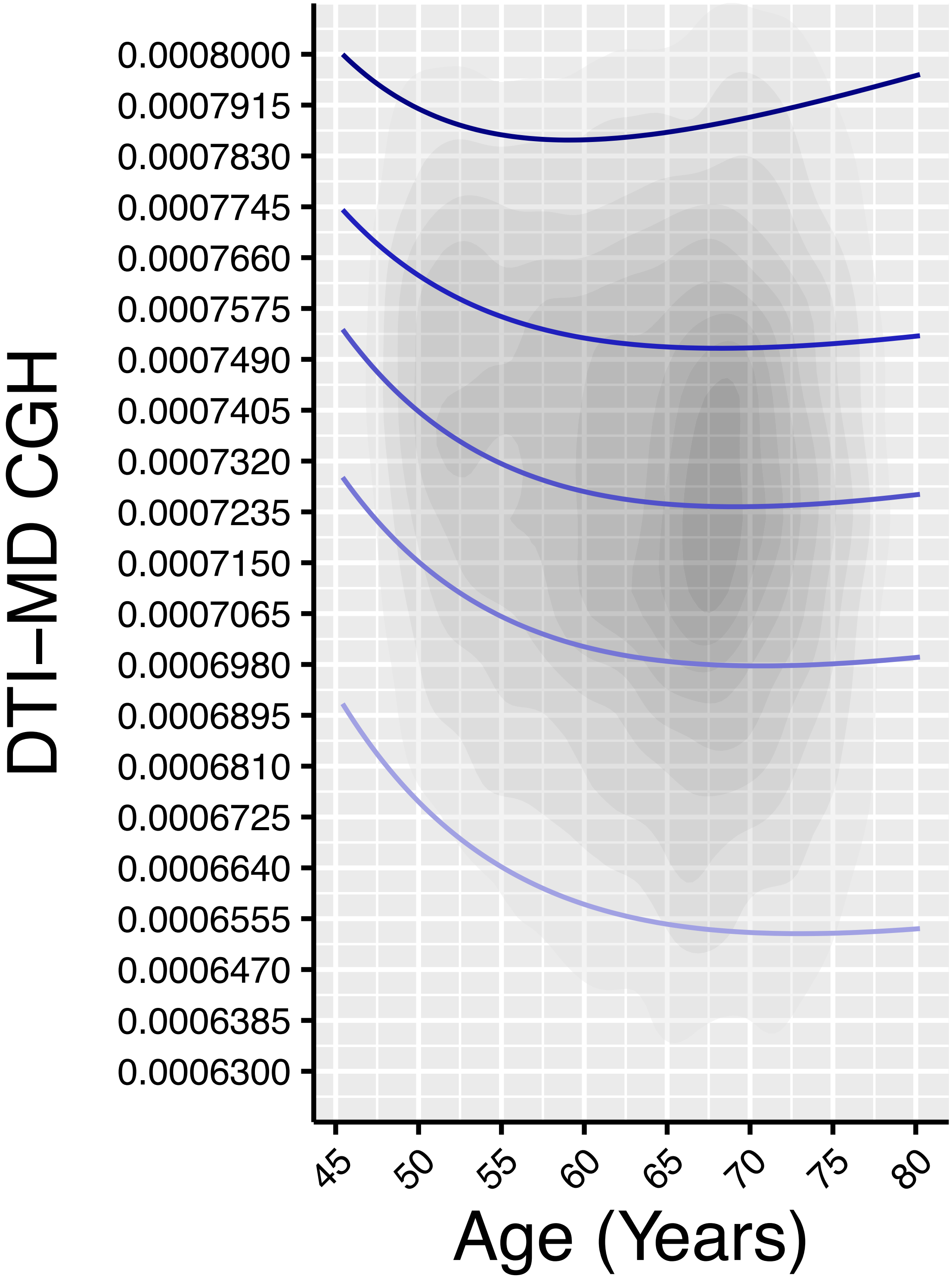

Figure S92. Full size normative centile reference curves calculated for the cingulum (hippocampal) for DTI-MD in males. Solid colored lines, ordered from lightest to darkest, indicate the following centiles: 5th, 25th, 50th, 75th, 95th. Gray overlay reflects kernel density (darker=greater degree of data point overlap). CGH = cingulum (hippocampal).

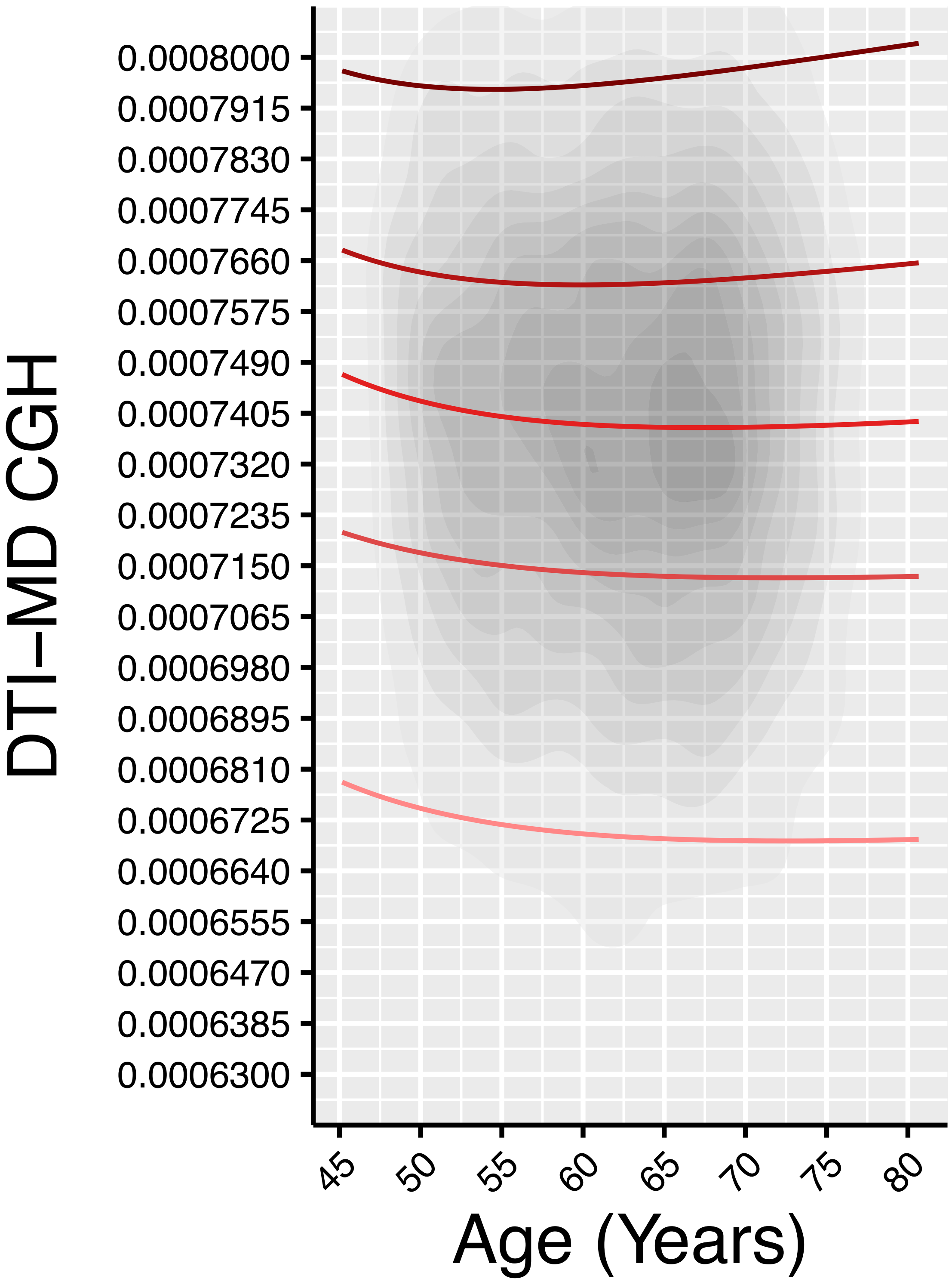

Figure S93. Full size normative centile reference curves calculated for the cingulum (hippocampal) for DTI-MD in females. Solid colored lines, ordered from lightest to darkest, indicate the following centiles: 5th, 25th, 50th, 75th, 95th. Gray overlay reflects kernel density (darker=greater degree of data point overlap). CGH = cingulum (hippocampal).

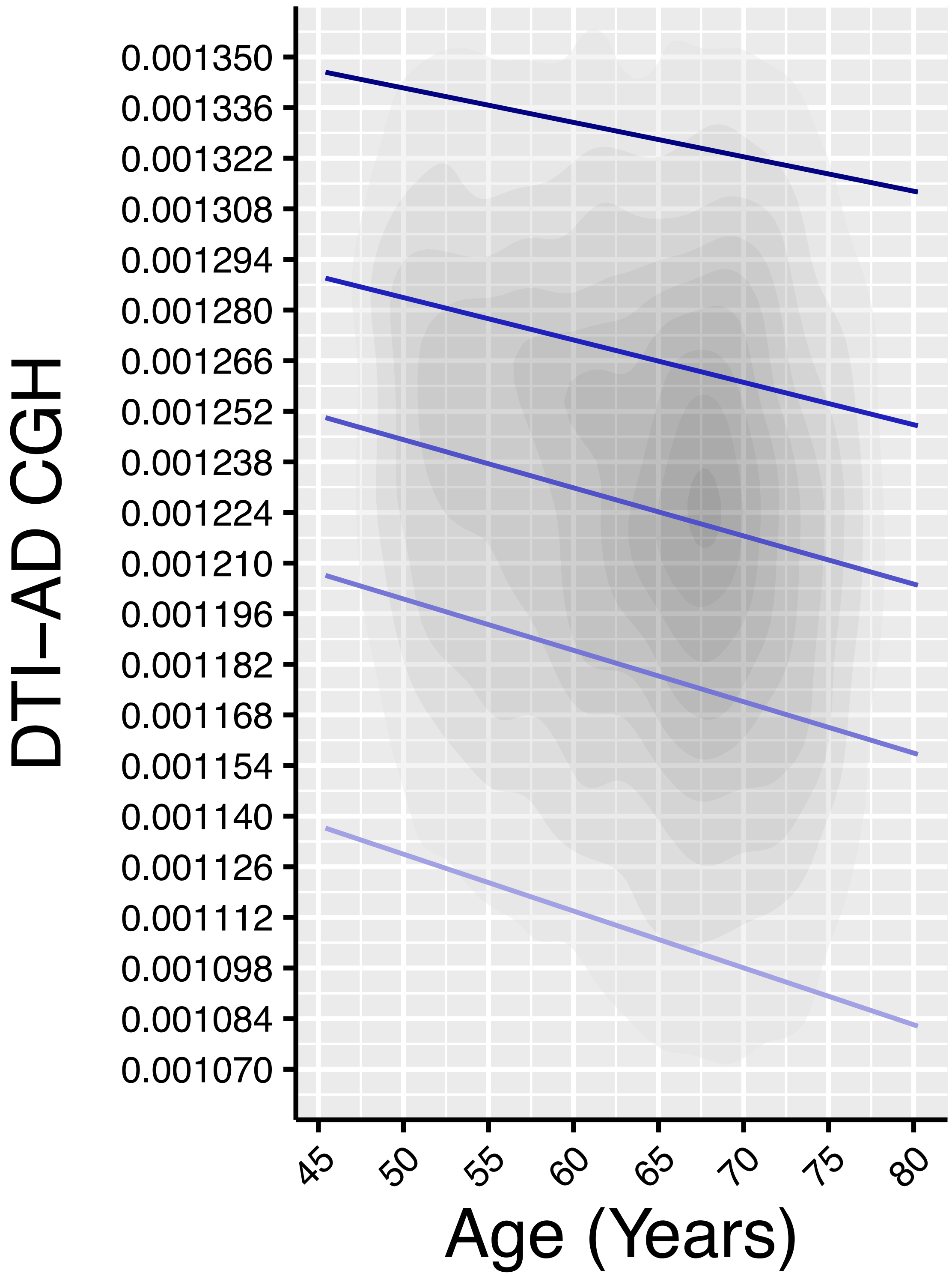

Figure S94. Full size normative centile reference curves calculated for the cingulum (hippocampal) for DTI-AD in males. Solid colored lines, ordered from lightest to darkest, indicate the following centiles: 5th, 25th, 50th, 75th, 95th. Gray overlay reflects kernel density (darker=greater degree of data point overlap). CGH = cingulum (hippocampal).

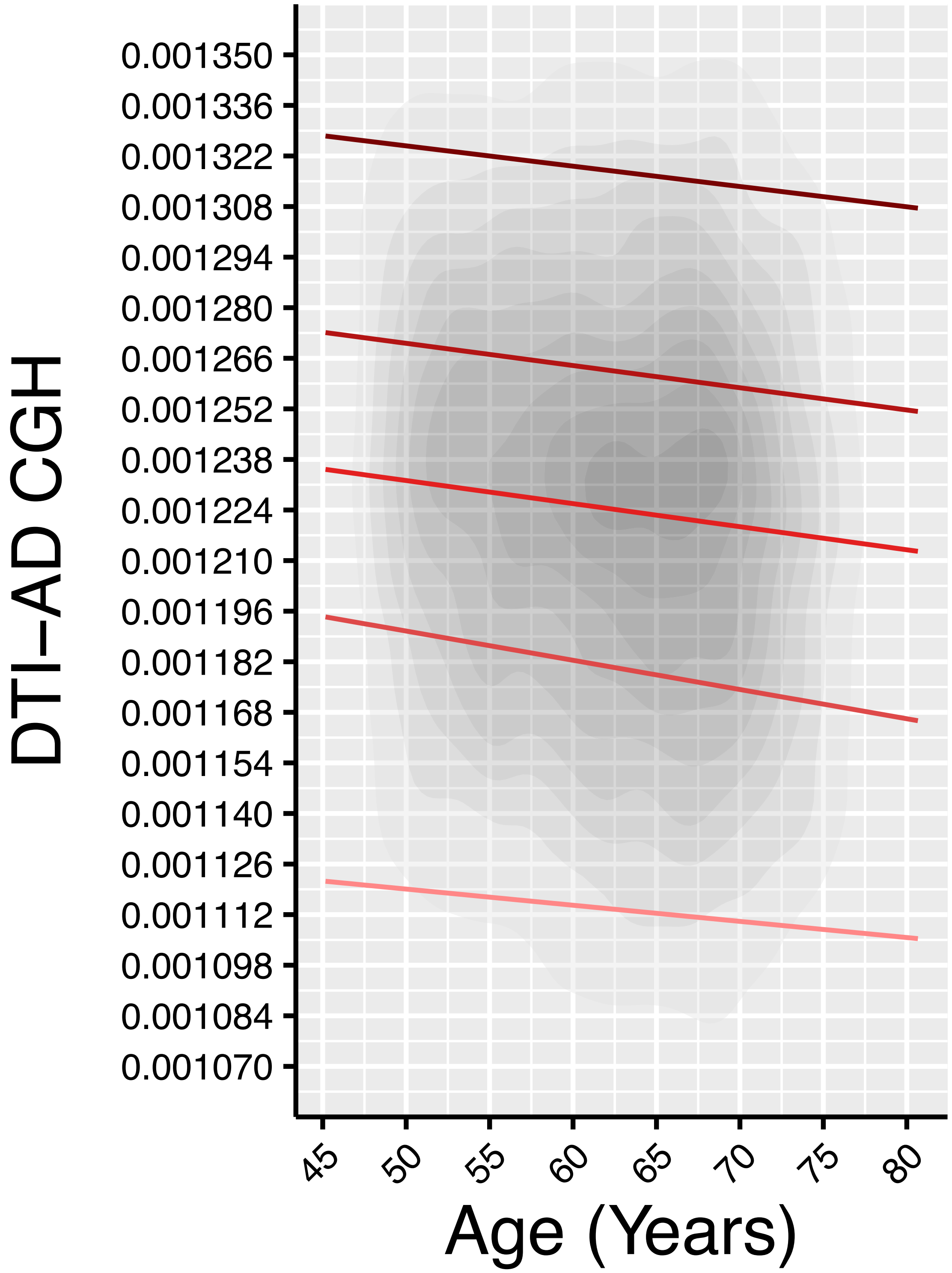

Figure S95. Full size normative centile reference curves calculated for the cingulum (hippocampal) for DTI-AD in females. Solid colored lines, ordered from lightest to darkest, indicate the following centiles: 5th, 25th, 50th, 75th, 95th. Gray overlay reflects kernel density (darker=greater degree of data point overlap). CGH = cingulum (hippocampal).

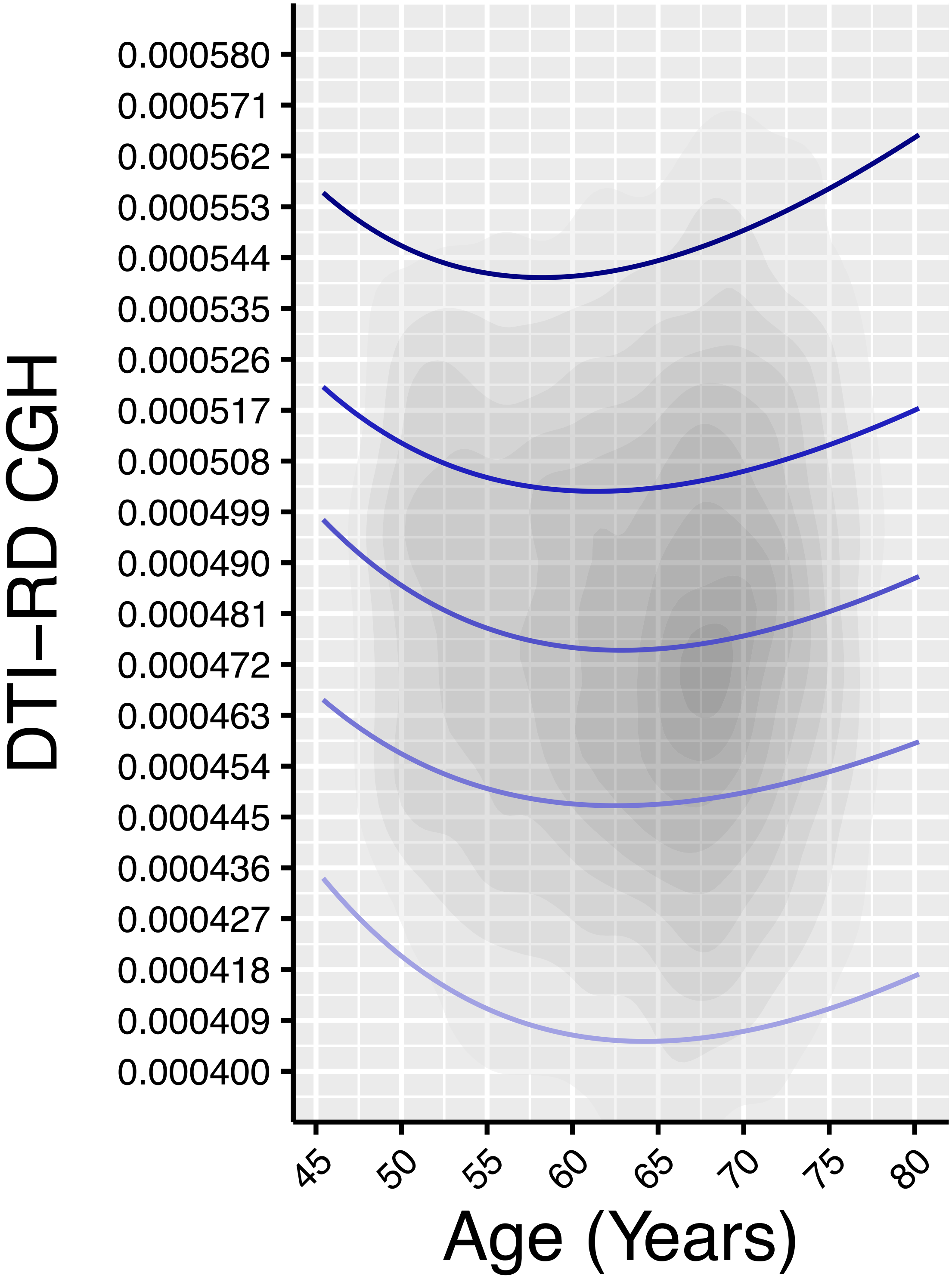

Figure S96. Full size normative centile reference curves calculated for the cingulum (hippocampal) for DTI-RD in males. Solid colored lines, ordered from lightest to darkest, indicate the following centiles: 5th, 25th, 50th, 75th, 95th. Gray overlay reflects kernel density (darker=greater degree of data point overlap). CGH = cingulum (hippocampal).

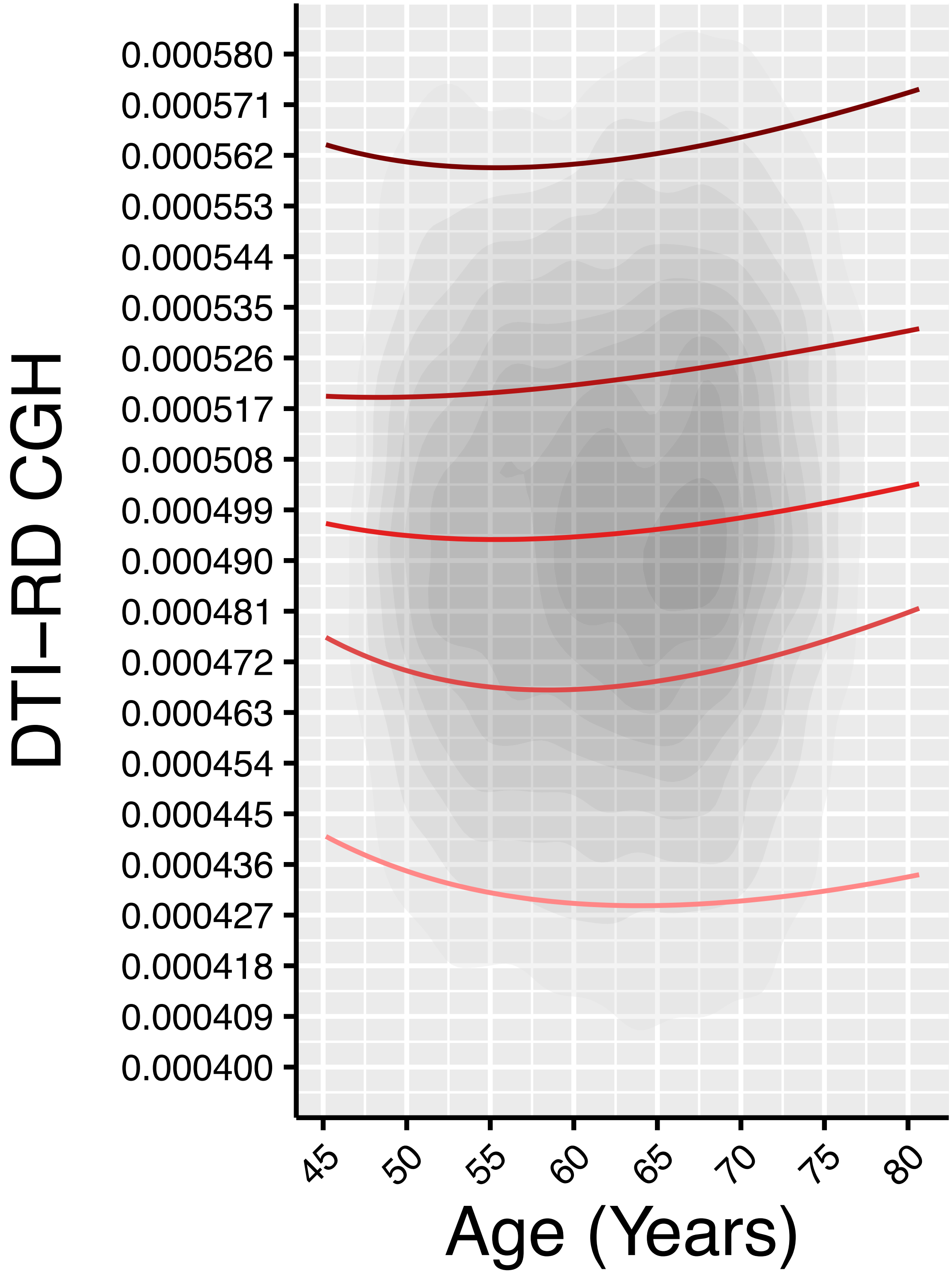

Figure S97. Full size normative centile reference curves calculated for the cingulum (hippocampal) for DTI-RD in females. Solid colored lines, ordered from lightest to darkest, indicate the following centiles: 5th, 25th, 50th, 75th, 95th. Gray overlay reflects kernel density (darker=greater degree of data point overlap). CGH = cingulum (hippocampal).

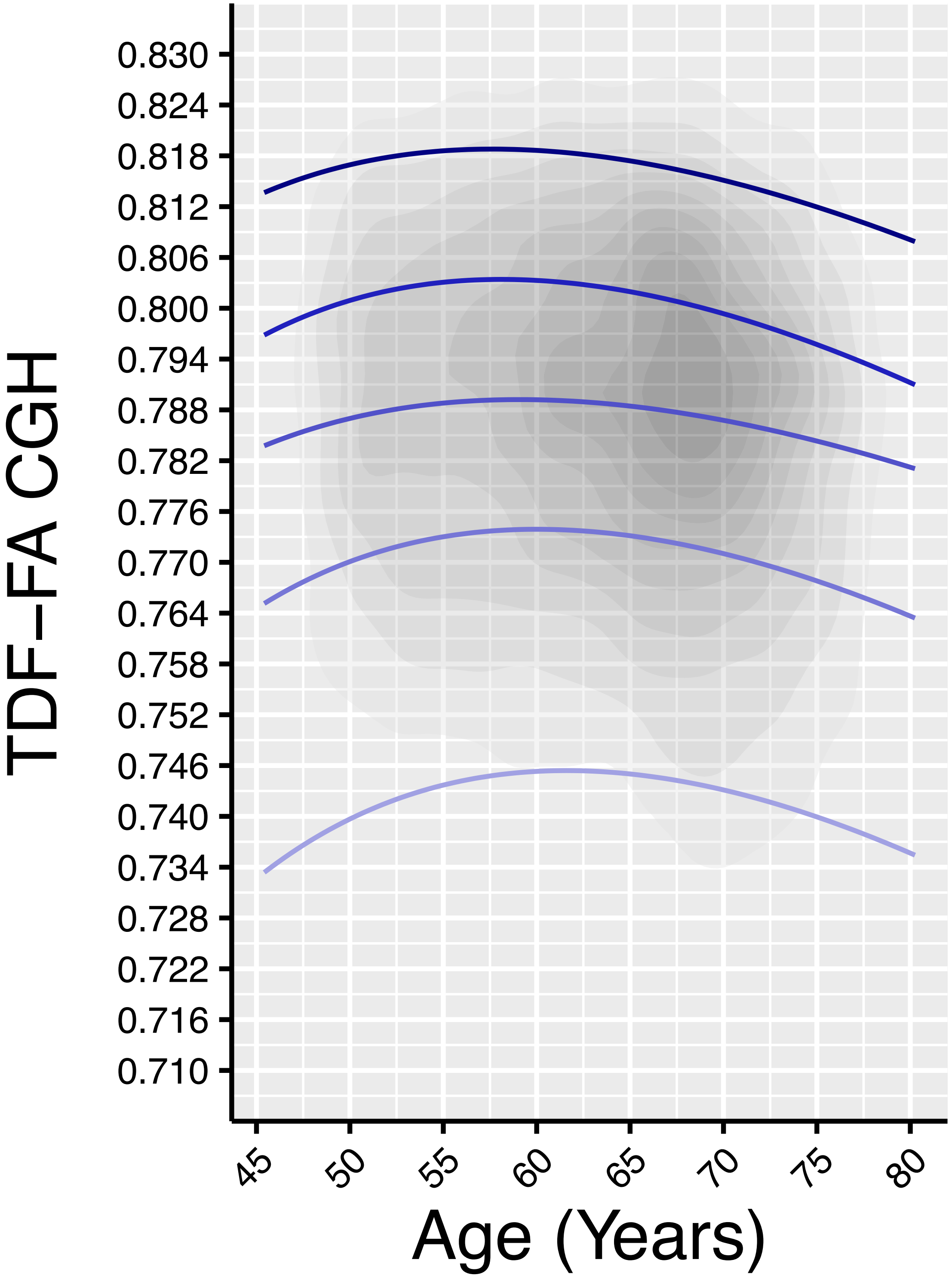

Figure S98. Full size normative centile reference curves calculated for the cingulum (hippocampal) for TDF-FA in males. Solid colored lines, ordered from lightest to darkest, indicate the following centiles: 5th, 25th, 50th, 75th, 95th. Gray overlay reflects kernel density (darker=greater degree of data point overlap). CGH = cingulum (hippocampal).

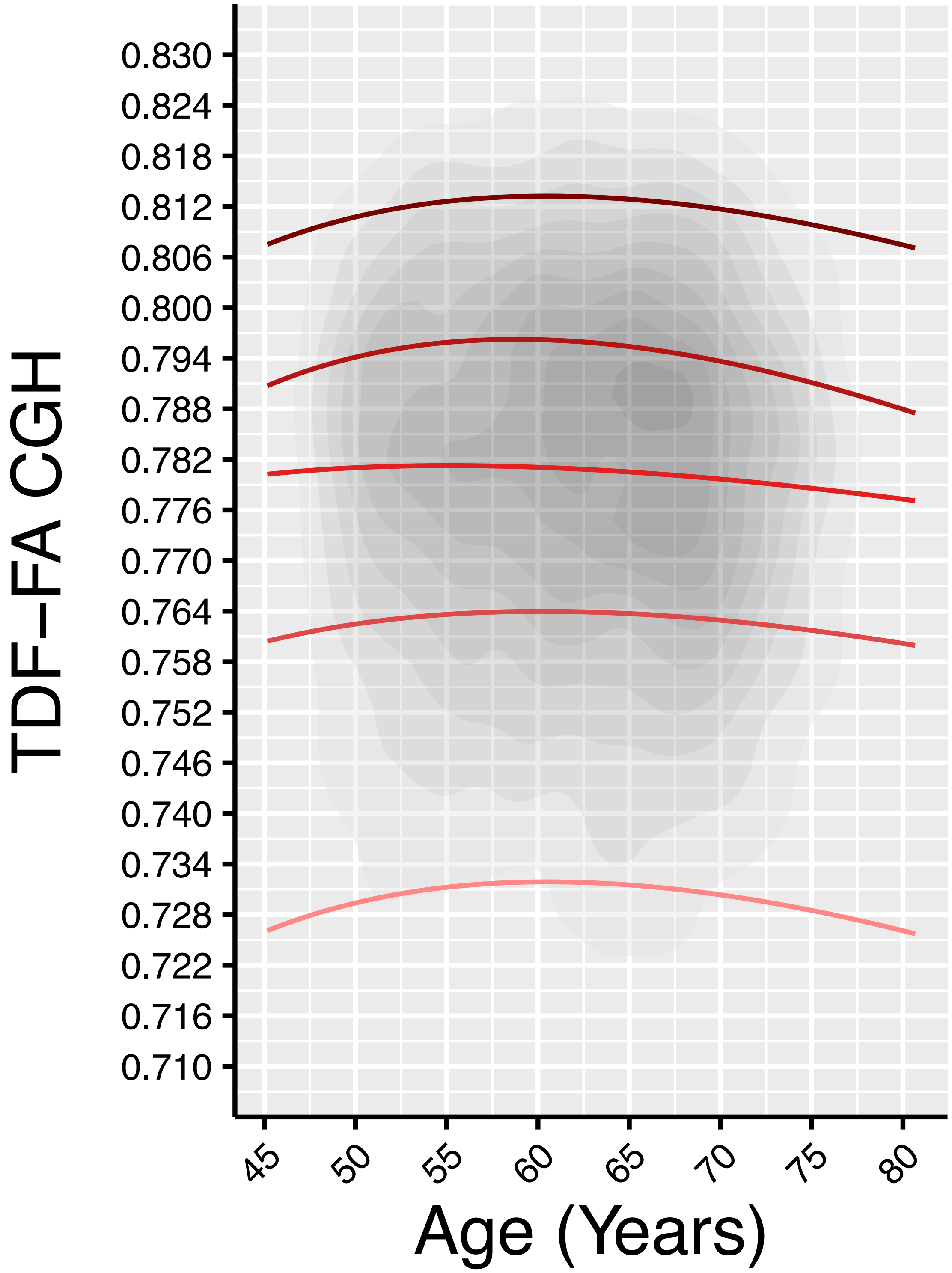

Figure S99. Full size normative centile reference curves calculated for the cingulum (hippocampal) for TDF-FA in females. Solid colored lines, ordered from lightest to darkest, indicate the following centiles: 5th, 25th, 50th, 75th, 95th. Gray overlay reflects kernel density (darker=greater degree of data point overlap). CGH = cingulum (hippocampal).

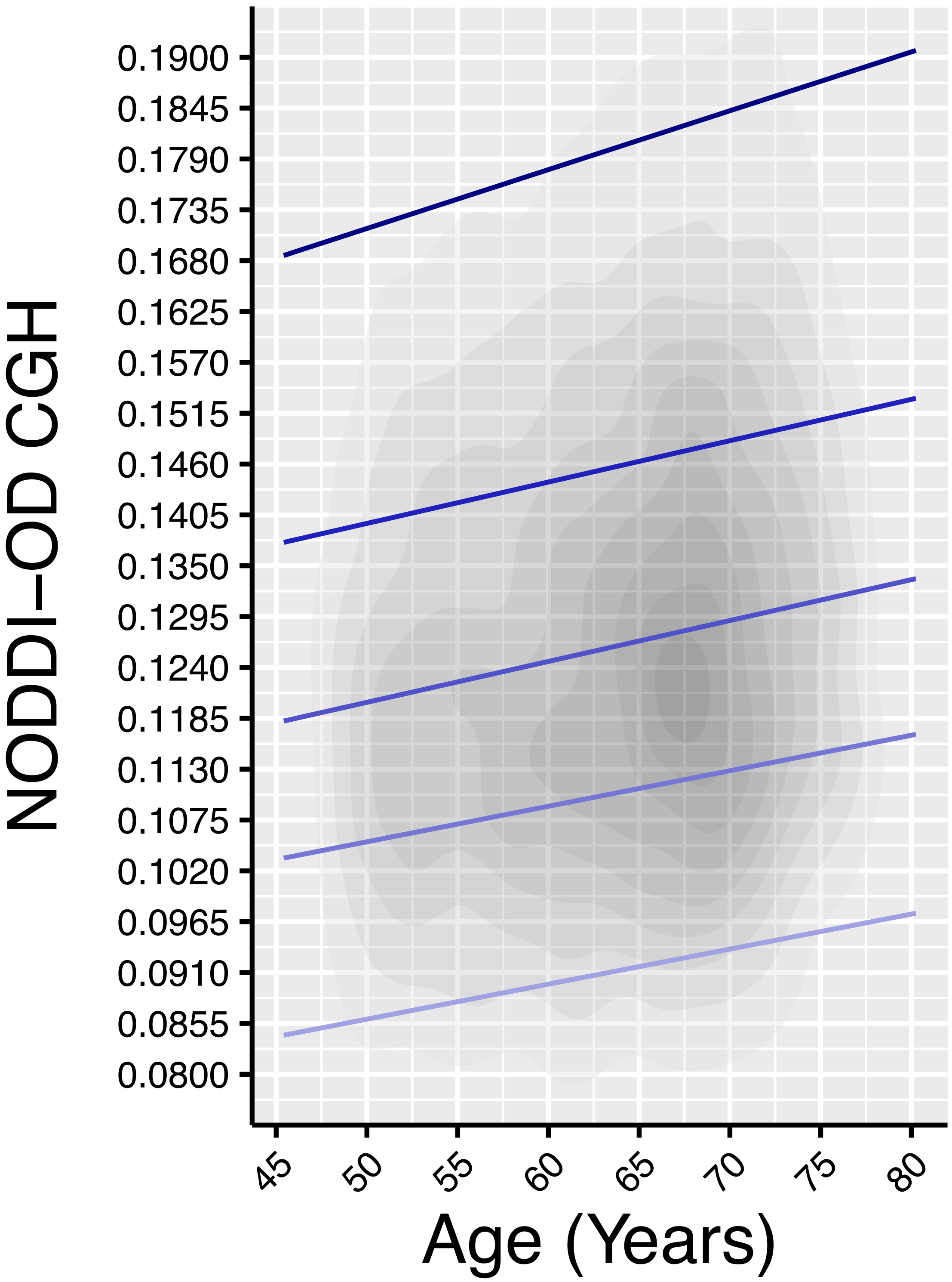

Figure S100. Full size normative centile reference curves calculated for the cingulum (hippocampal) for NODDI-OD in males. Solid colored lines, ordered from lightest to darkest, indicate the following centiles: 5th, 25th, 50th, 75th, 95th. Gray overlay reflects kernel density (darker=greater degree of data point overlap). CGH = cingulum (hippocampal).

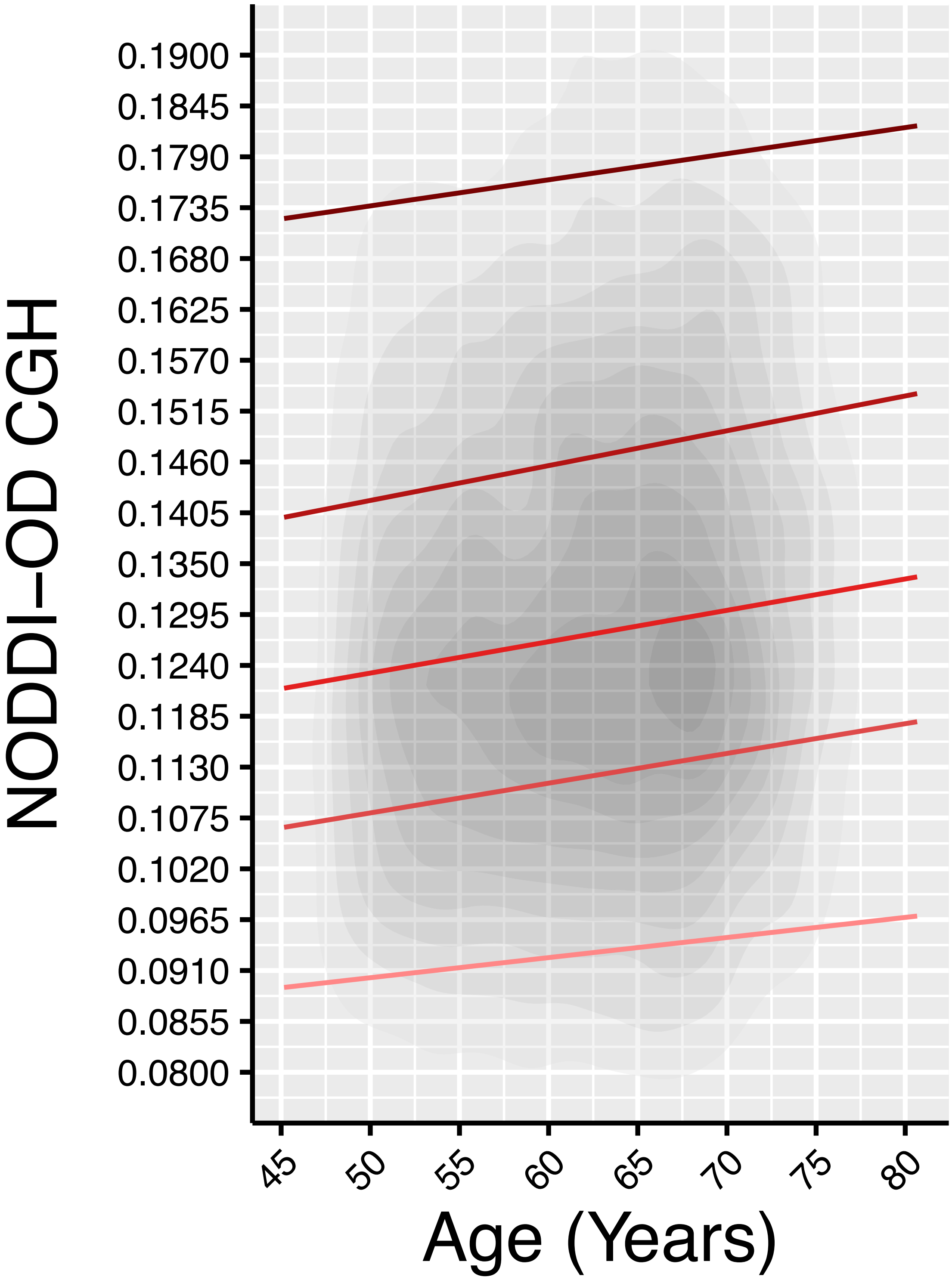

Figure S101. Full size normative centile reference curves calculated for the cingulum (hippocampal) for NODDI-OD in females. Solid colored lines, ordered from lightest to darkest, indicate the following centiles: 5th, 25th, 50th, 75th, 95th. Gray overlay reflects kernel density (darker=greater degree of data point overlap). CGH = cingulum (hippocampal).

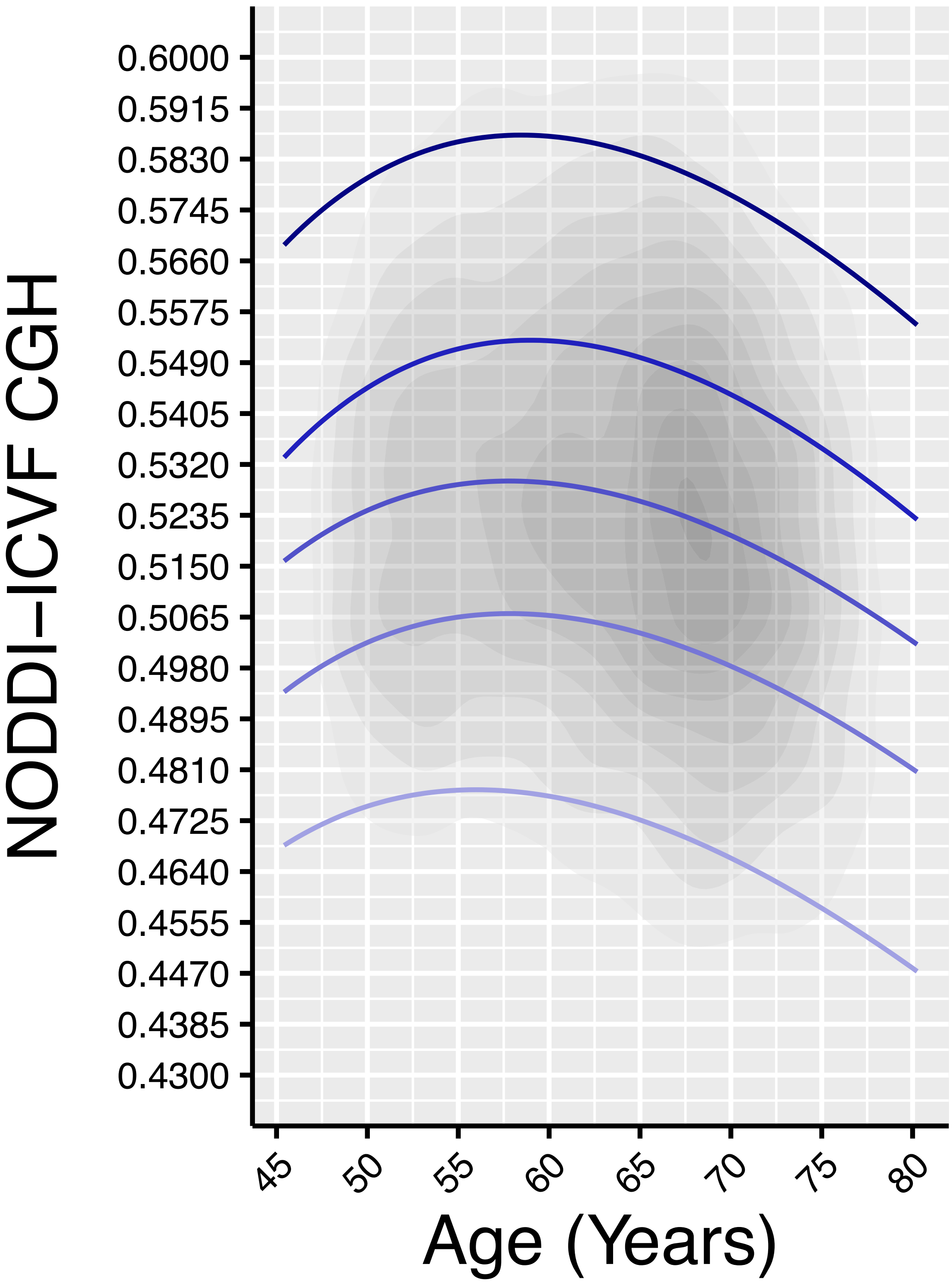

Figure S102. Full size normative centile reference curves calculated for the cingulum (hippocampal) for NODDI-ICVF in males. Solid colored lines, ordered from lightest to darkest, indicate the following centiles: 5th, 25th, 50th, 75th, 95th. Gray overlay reflects kernel density (darker=greater degree of data point overlap). CGH = cingulum (hippocampal).

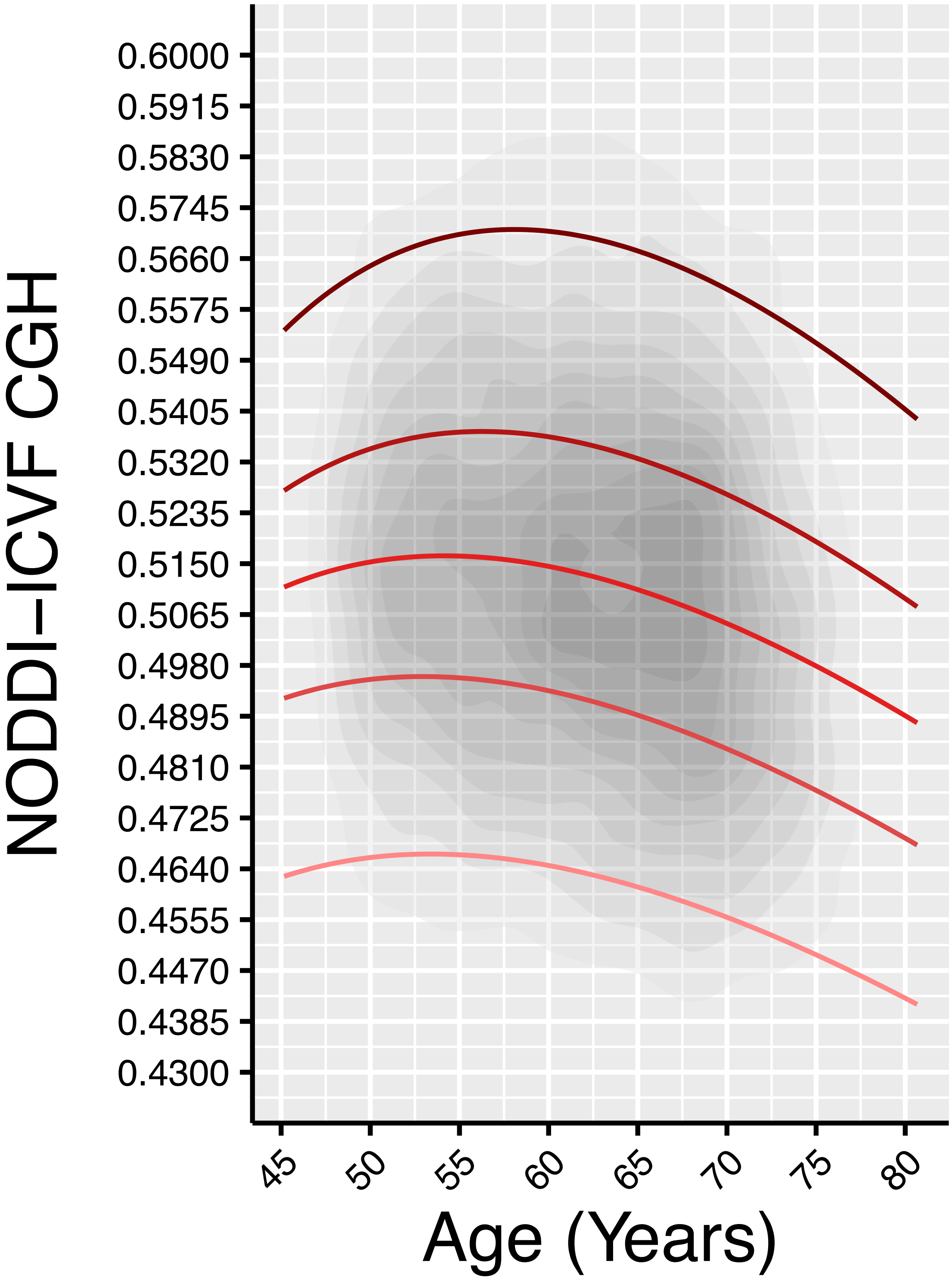

Figure S103. Full size normative centile reference curves calculated for the cingulum (hippocampal) for NODDI-ICVF in females. Solid colored lines, ordered from lightest to darkest, indicate the following centiles: 5th, 25th, 50th, 75th, 95th. Gray overlay reflects kernel density (darker=greater degree of data point overlap). CGH = cingulum (hippocampal).

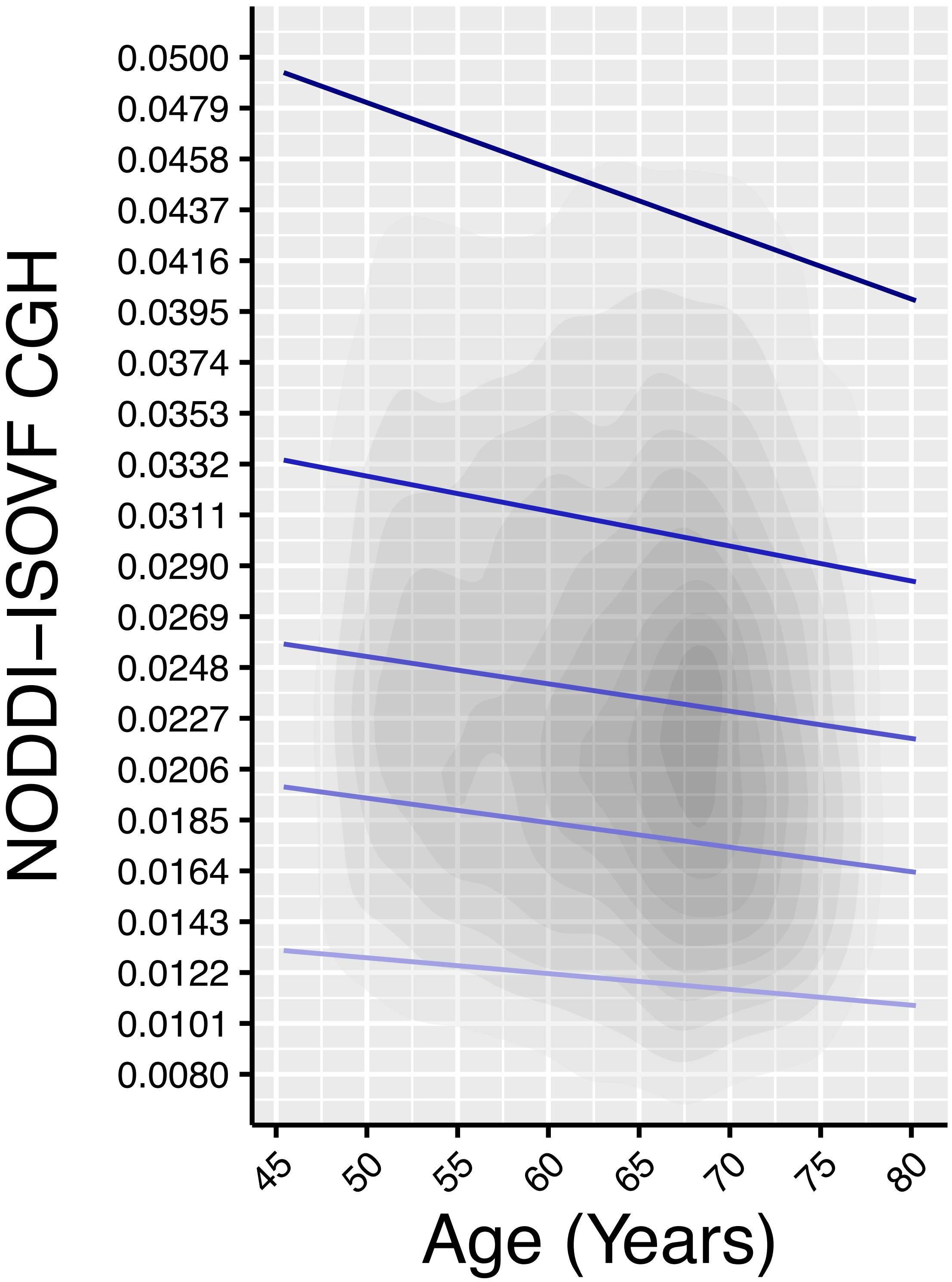

Figure S104. Full size normative centile reference curves calculated for the cingulum (hippocampal) for NODDI-ISOVF in males. Solid colored lines, ordered from lightest to darkest, indicate the following centiles: 5th, 25th, 50th, 75th, 95th. Gray overlay reflects kernel density (darker=greater degree of data point overlap). CGH = cingulum (hippocampal).

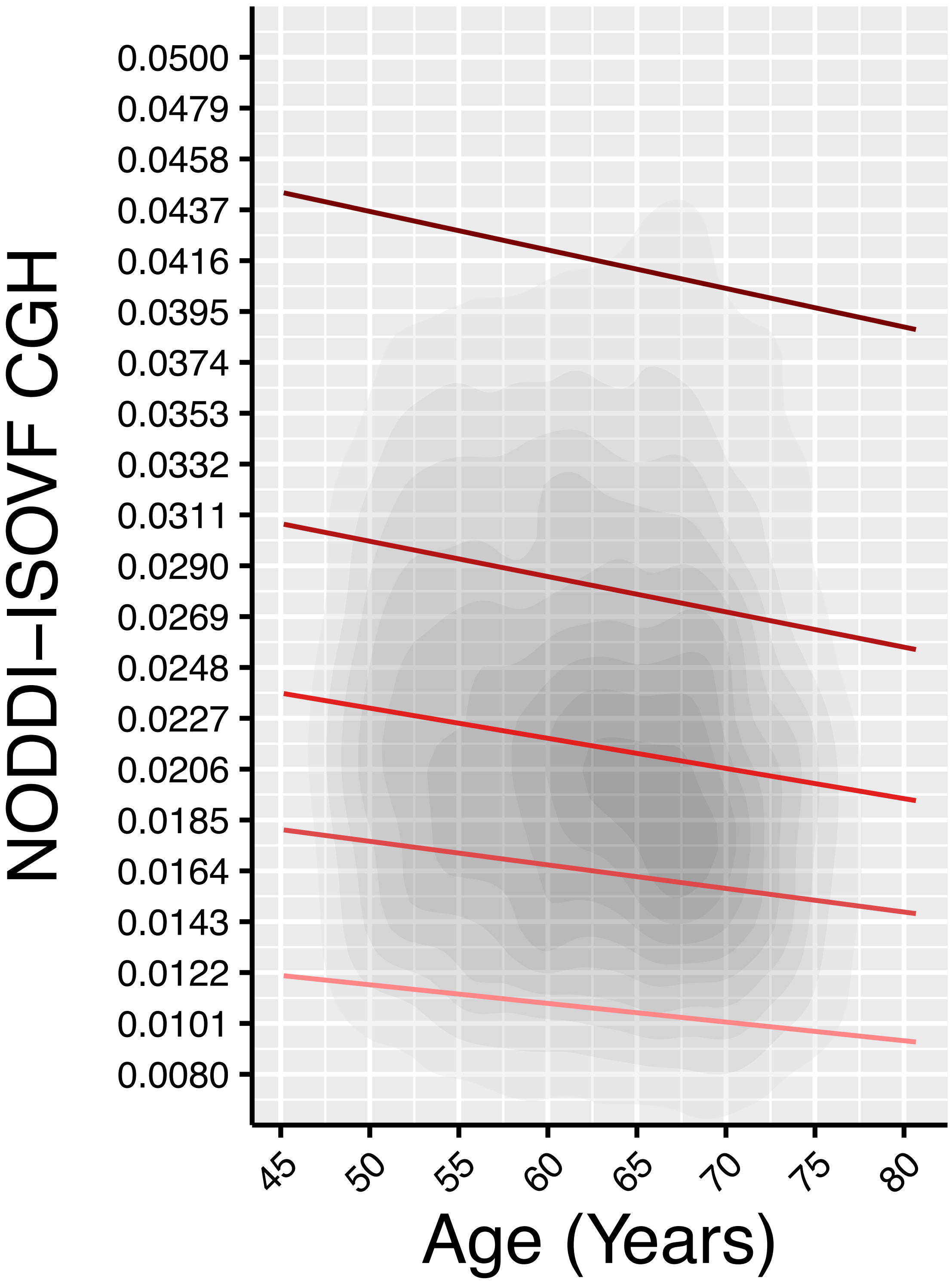

Figure S105. Full size normative centile reference curves calculated for the cingulum (hippocampal) for NODDI-ISOVF in females. Solid colored lines, ordered from lightest to darkest, indicate the following centiles: 5th, 25th, 50th, 75th, 95th. Gray overlay reflects kernel density (darker=greater degree of data point overlap). CGH = cingulum (hippocampal).

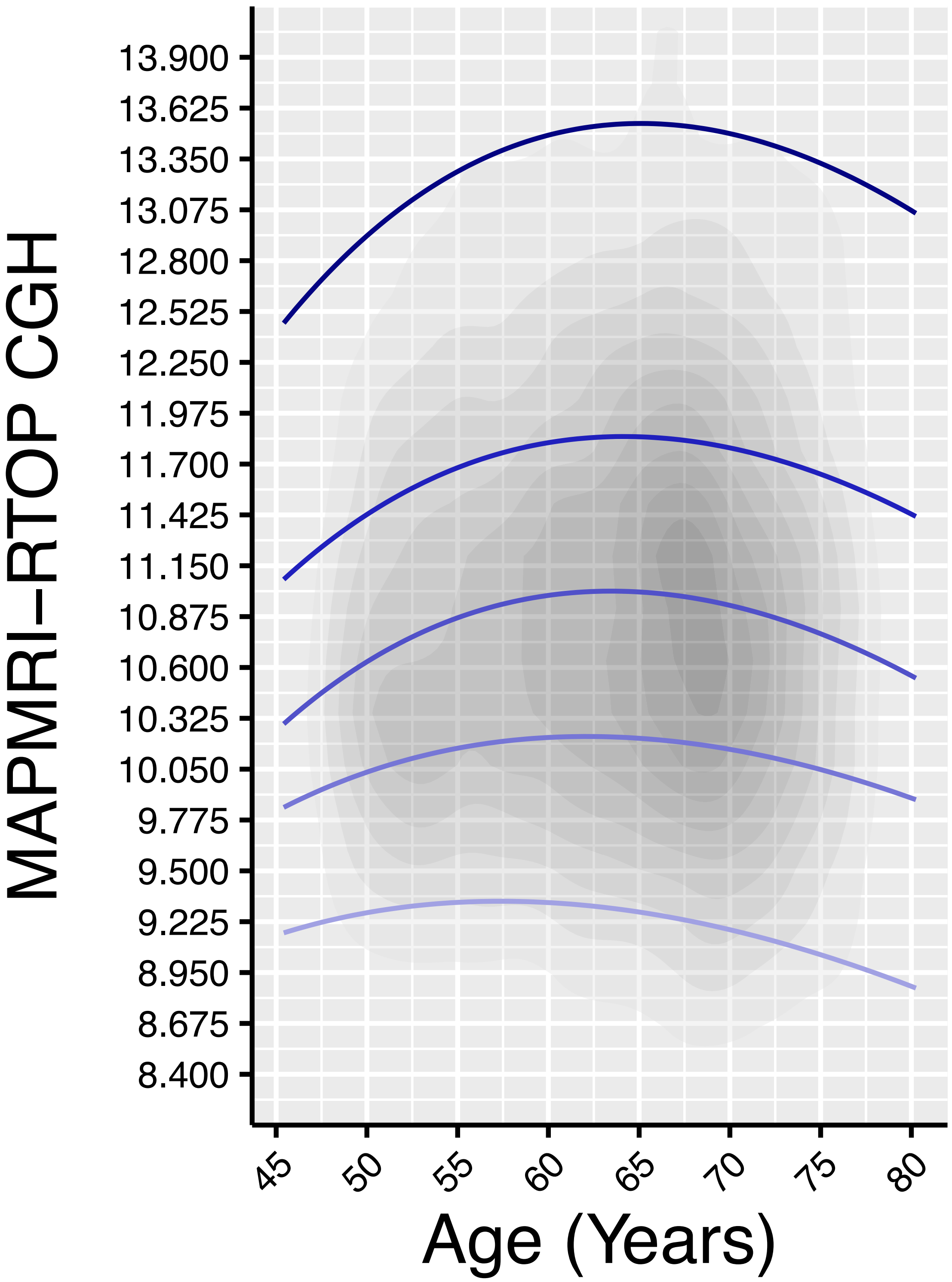

Figure S106. Full size normative centile reference curves calculated for the cingulum (hippocampal) for MAPMRI-RTOP in males. Solid colored lines, ordered from lightest to darkest, indicate the following centiles: 5th, 25th, 50th, 75th, 95th. Gray overlay reflects kernel density (darker=greater degree of data point overlap). CGH = cingulum (hippocampal).

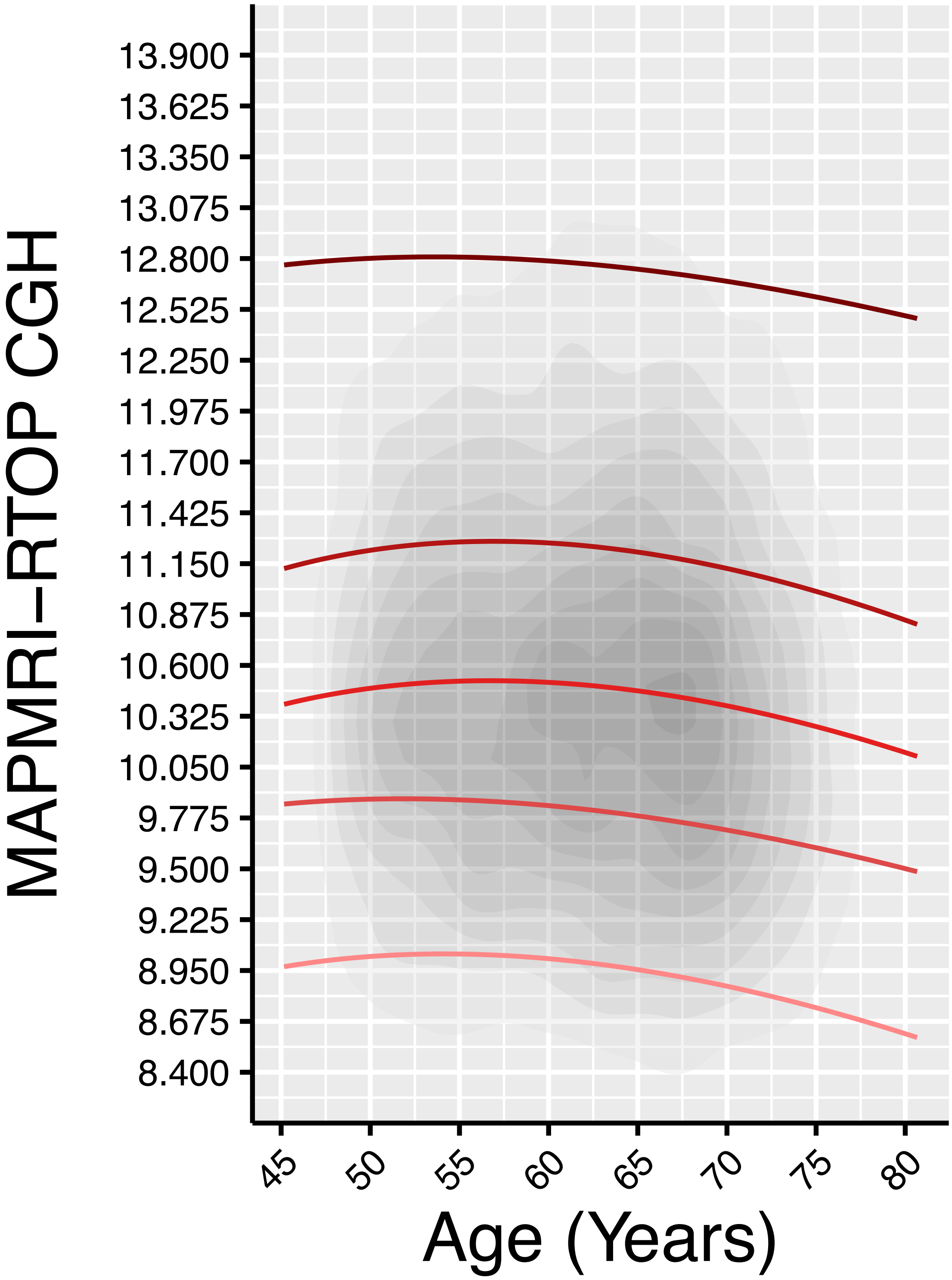

Figure S107. Full size normative centile reference curves calculated for the cingulum (hippocampal) for MAPMRI-RTOP in females. Solid colored lines, ordered from lightest to darkest, indicate the following centiles: 5th, 25th, 50th, 75th, 95th. Gray overlay reflects kernel density (darker=greater degree of data point overlap). CGH = cingulum (hippocampal).

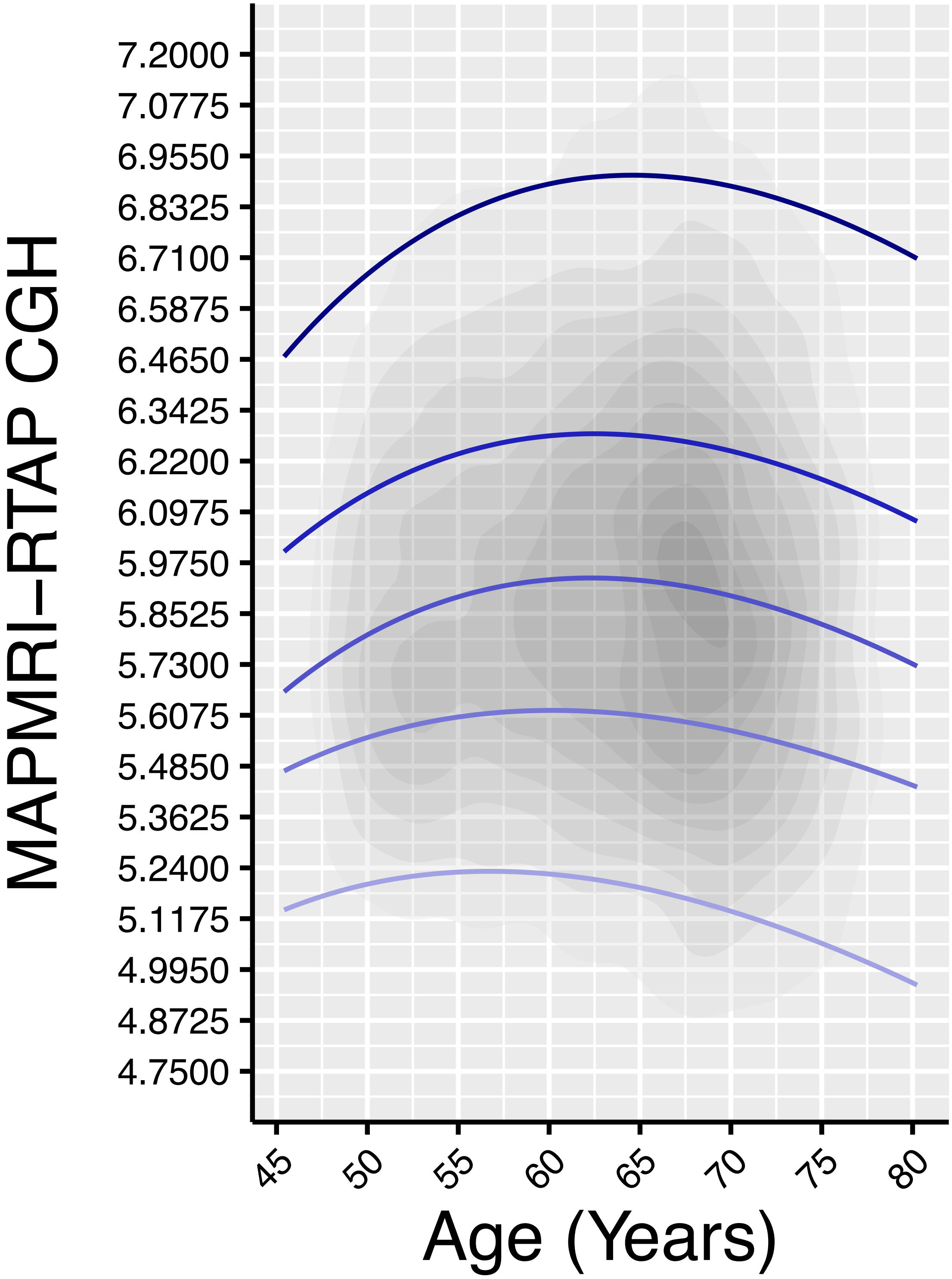

Figure S108. Full size normative centile reference curves calculated for the cingulum (hippocampal) for MAPMRI-RTAP in males. Solid colored lines, ordered from lightest to darkest, indicate the following centiles: 5th, 25th, 50th, 75th, 95th. Gray overlay reflects kernel density (darker=greater degree of data point overlap). CGH = cingulum (hippocampal).

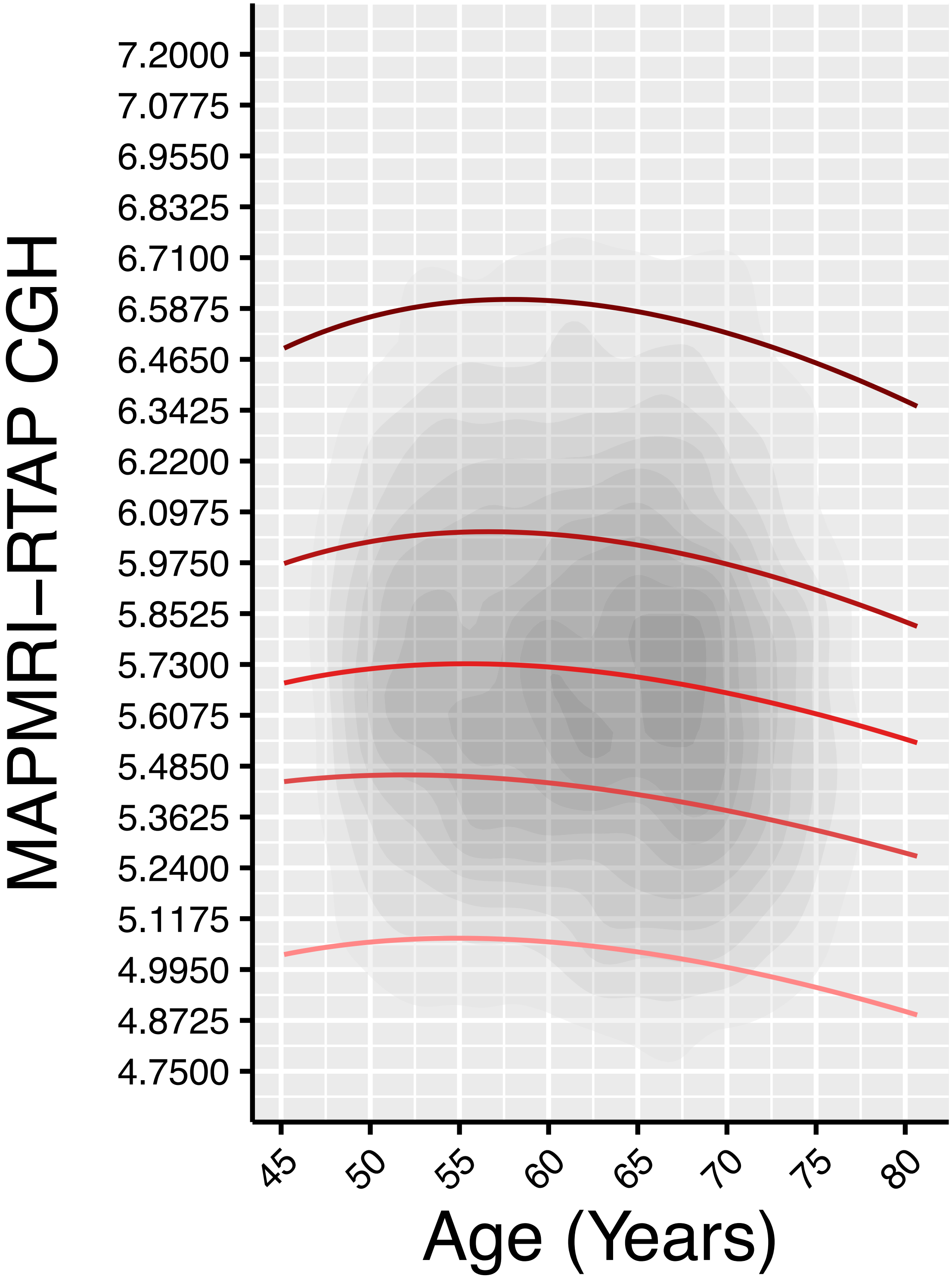

Figure S109. Full size normative centile reference curves calculated for the cingulum (hippocampal) for MAPMRI-RTAP in females. Solid colored lines, ordered from lightest to darkest, indicate the following centiles: 5th, 25th, 50th, 75th, 95th. Gray overlay reflects kernel density (darker=greater degree of data point overlap). CGH = cingulum (hippocampal).

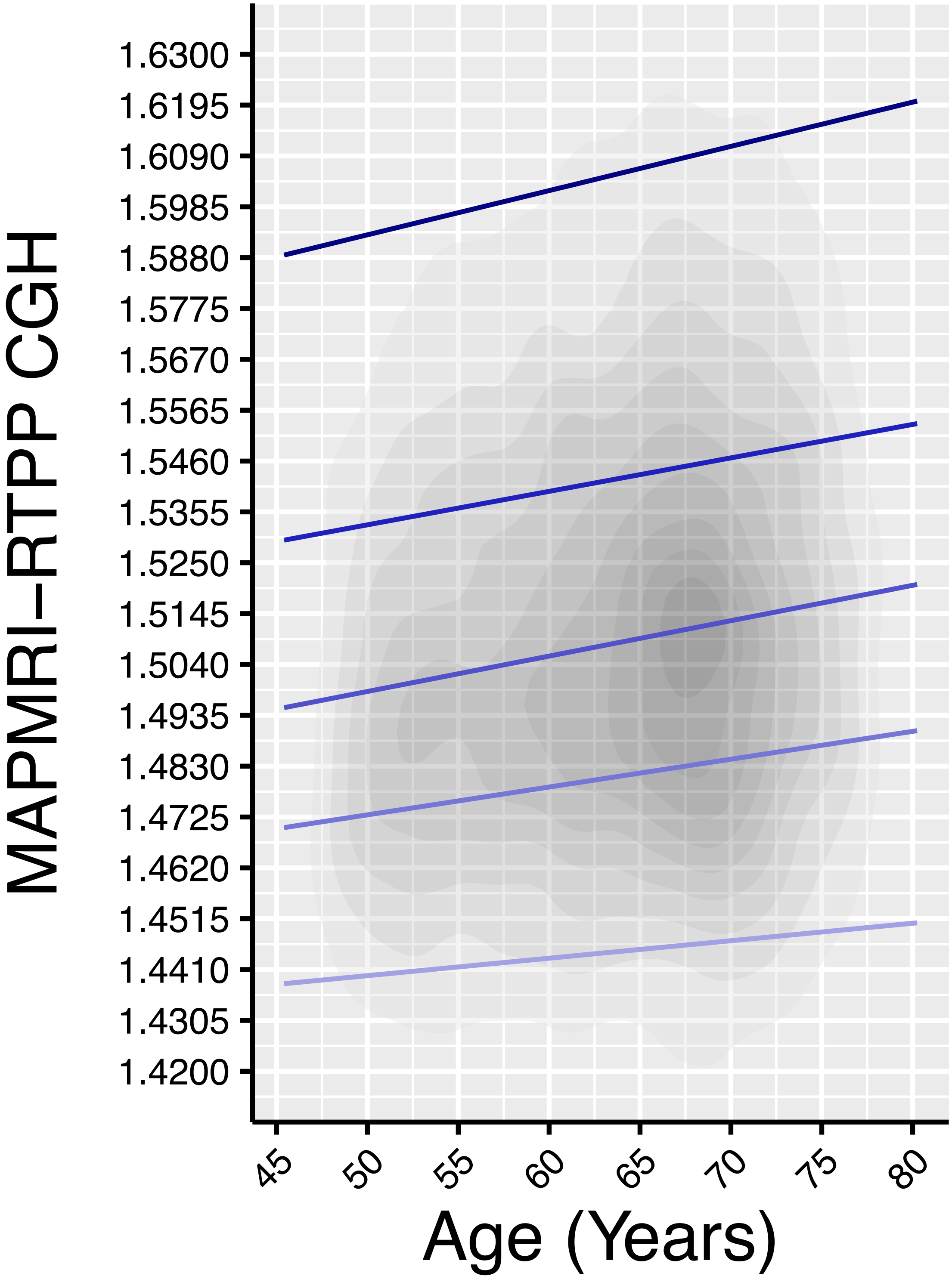

Figure S110. Full size normative centile reference curves calculated for the cingulum (hippocampal) for MAPMRI-RTPP in males. Solid colored lines, ordered from lightest to darkest, indicate the following centiles: 5th, 25th, 50th, 75th, 95th. Gray overlay reflects kernel density (darker=greater degree of data point overlap). CGH = cingulum (hippocampal).

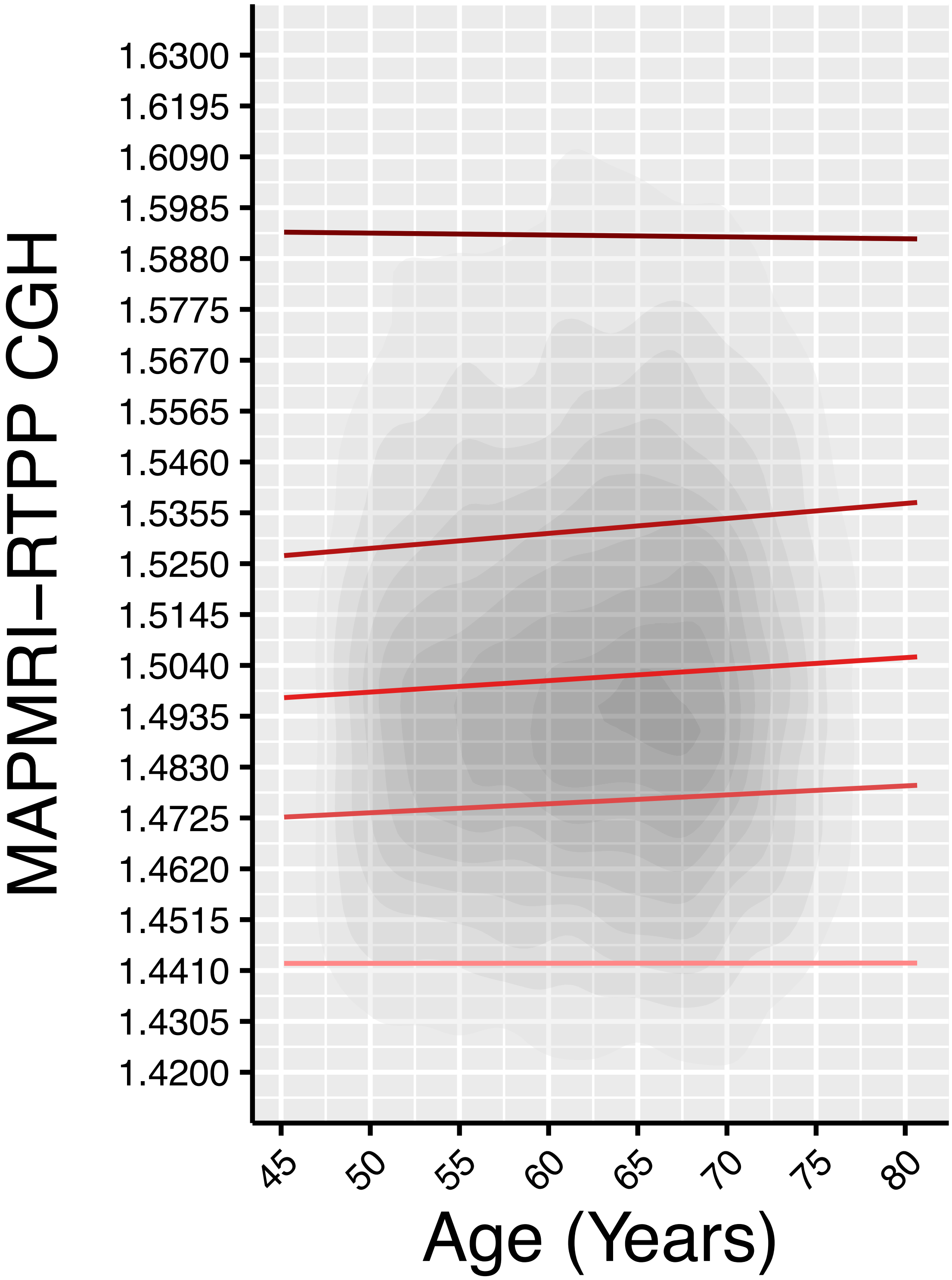

Figure S111. Full size normative centile reference curves calculated for the cingulum (hippocampal) for MAPMRI-RTPP in females. Solid colored lines, ordered from lightest to darkest, indicate the following centiles: 5th, 25th, 50th, 75th, 95th. Gray overlay reflects kernel density (darker=greater degree of data point overlap). CGH = cingulum (hippocampal).

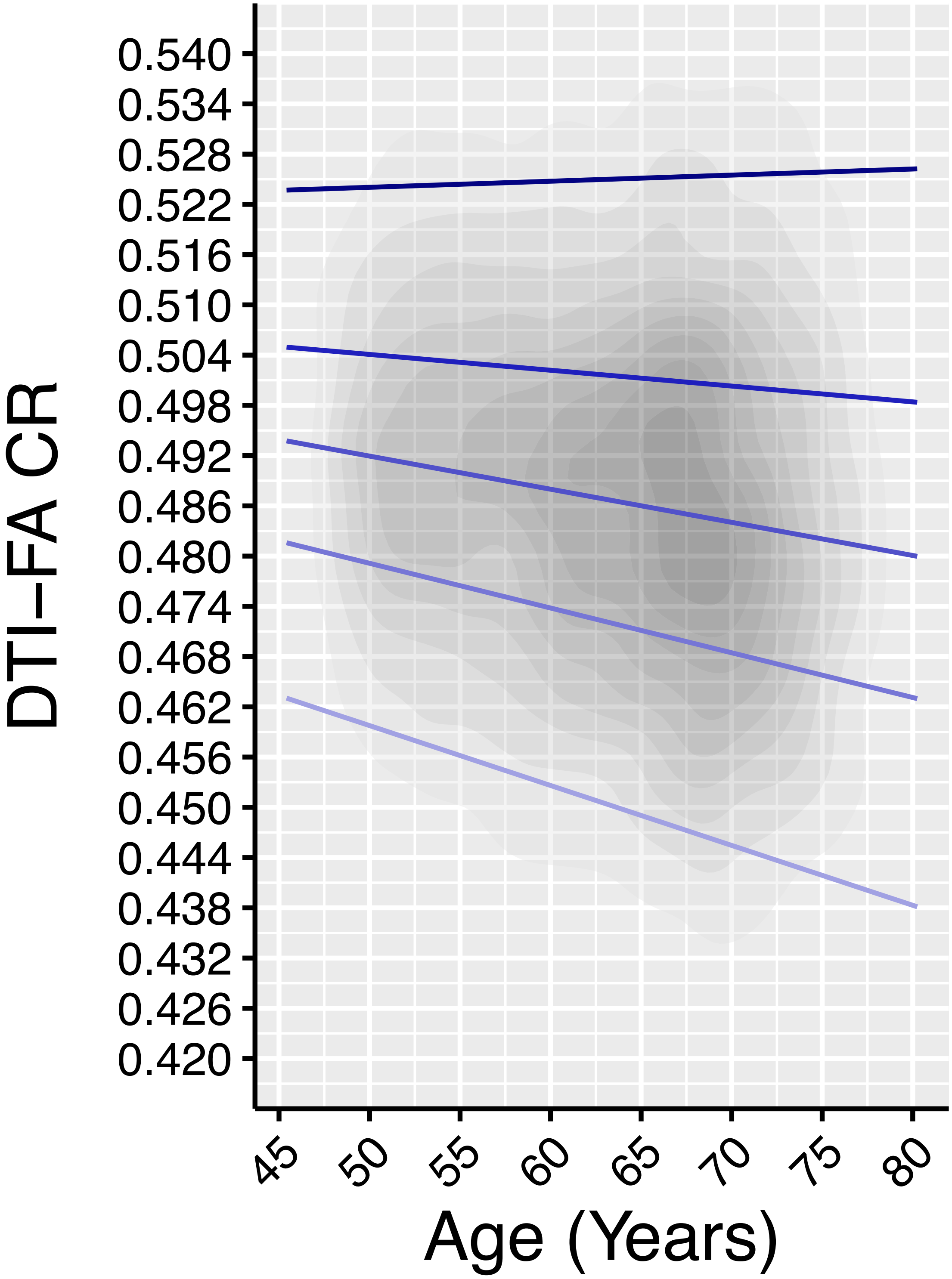

Figure S112. Full size normative centile reference curves calculated for the *corona radiata* for DTI-FA in males. Solid colored lines, ordered from lightest to darkest, indicate the following centiles: 5th, 25th, 50th, 75th, 95th. Gray overlay reflects kernel density (darker=greater degree of data point overlap). CR = *corona radiata.*

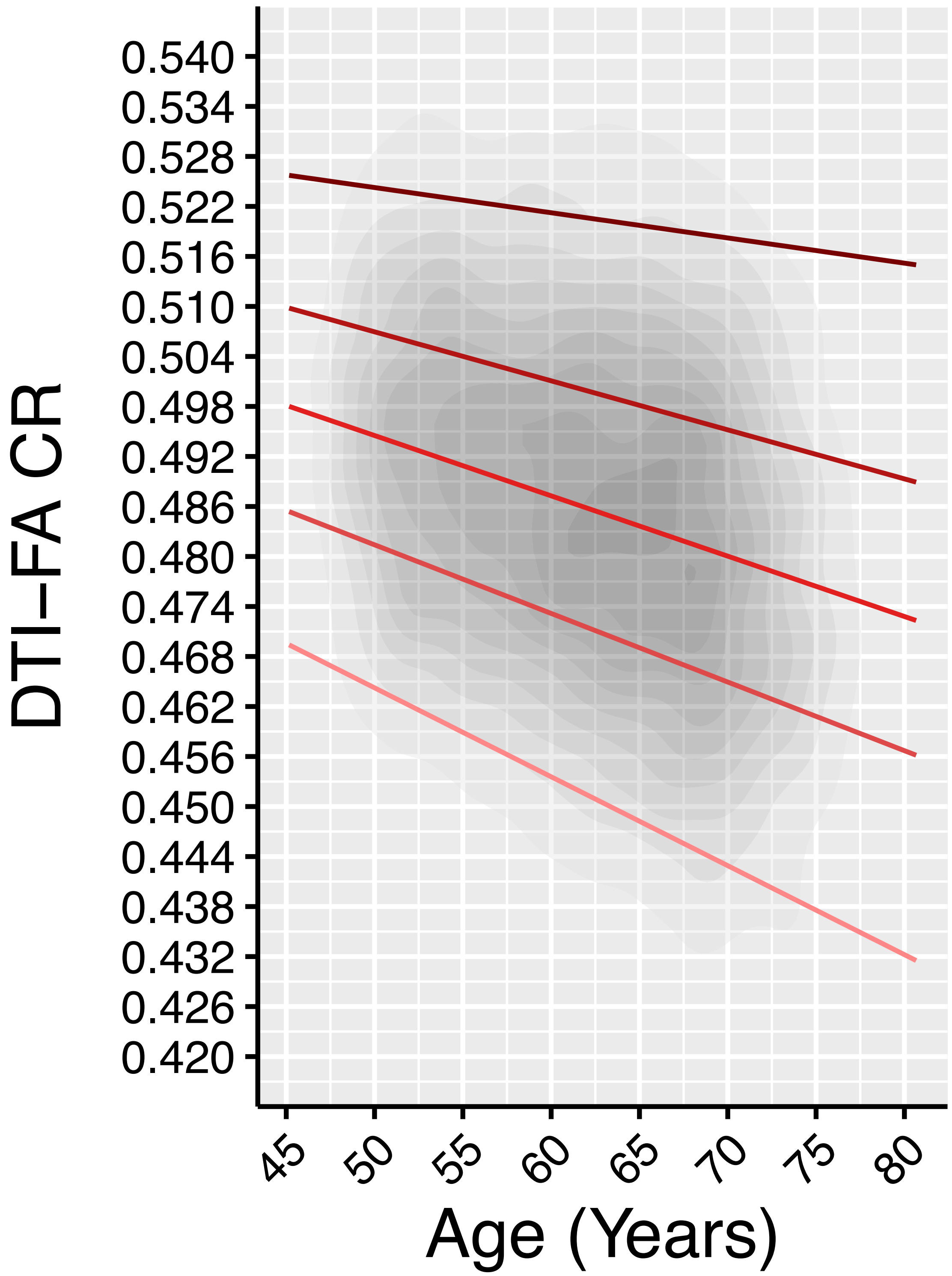

Figure S113. Full size normative centile reference curves calculated for the *corona radiata* for DTI-FA in females. Solid colored lines, ordered from lightest to darkest, indicate the following centiles: 5th, 25th, 50th, 75th, 95th. Gray overlay reflects kernel density (darker=greater degree of data point overlap). CR = *corona radiata.*

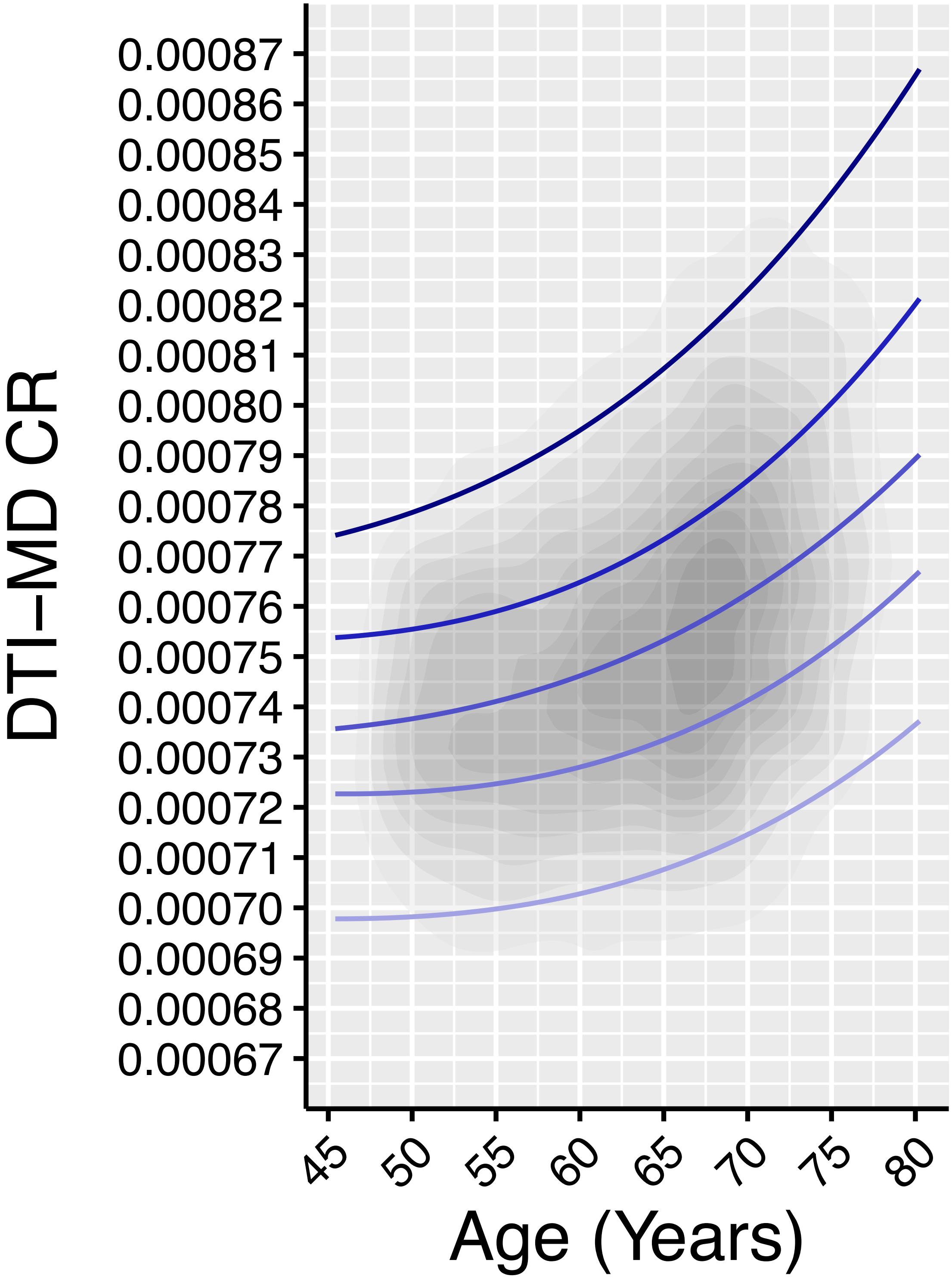

Figure S114. Full size normative centile reference curves calculated for the *corona radiata* for DTI-MD in males. Solid colored lines, ordered from lightest to darkest, indicate the following centiles: 5th, 25th, 50th, 75th, 95th. Gray overlay reflects kernel density (darker=greater degree of data point overlap). CR = *corona radiata***.**

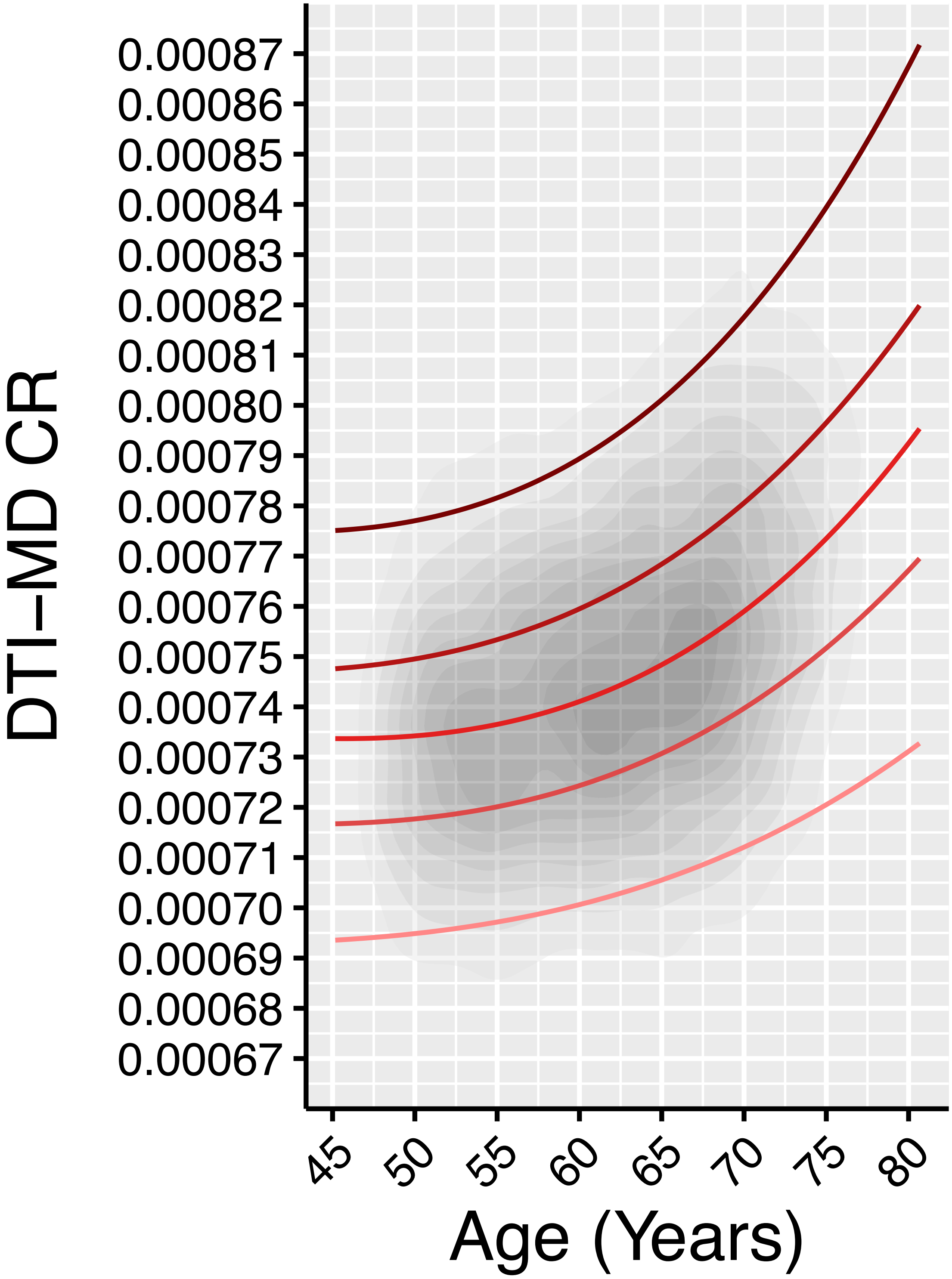

Figure S115. Full size normative centile reference curves calculated for the *corona radiata* for DTI-MD in females. Solid colored lines, ordered from lightest to darkest, indicate the following centiles: 5th, 25th, 50th, 75th, 95th. Gray overlay reflects kernel density (darker=greater degree of data point overlap). CR = *corona radiata*.

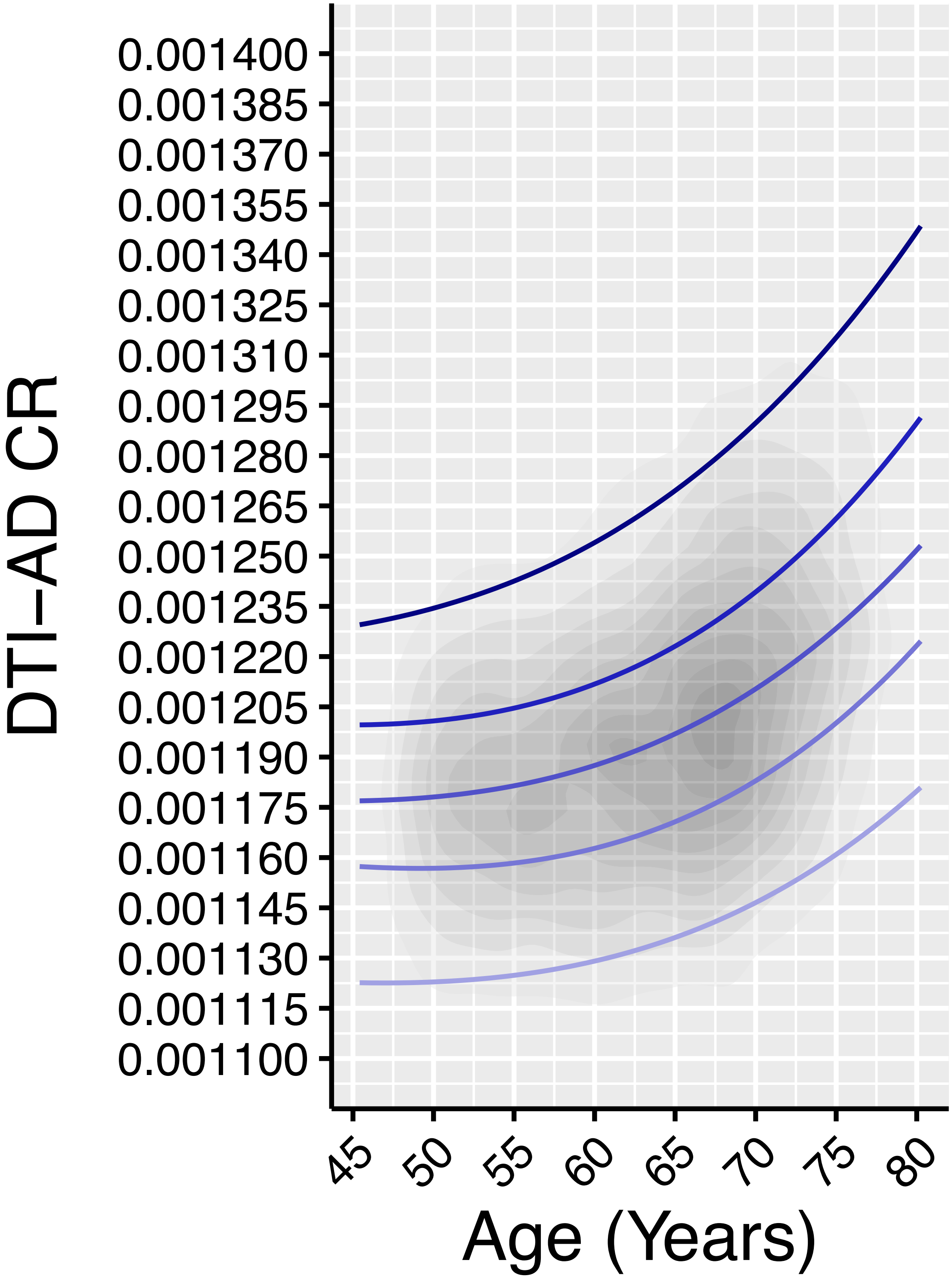

Figure S116. Full size normative centile reference curves calculated for the *corona radiata* for DTI-AD in males. Solid colored lines, ordered from lightest to darkest, indicate the following centiles: 5th, 25th, 50th, 75th, 95th. Gray overlay reflects kernel density (darker=greater degree of data point overlap). CR = *corona radiata*.

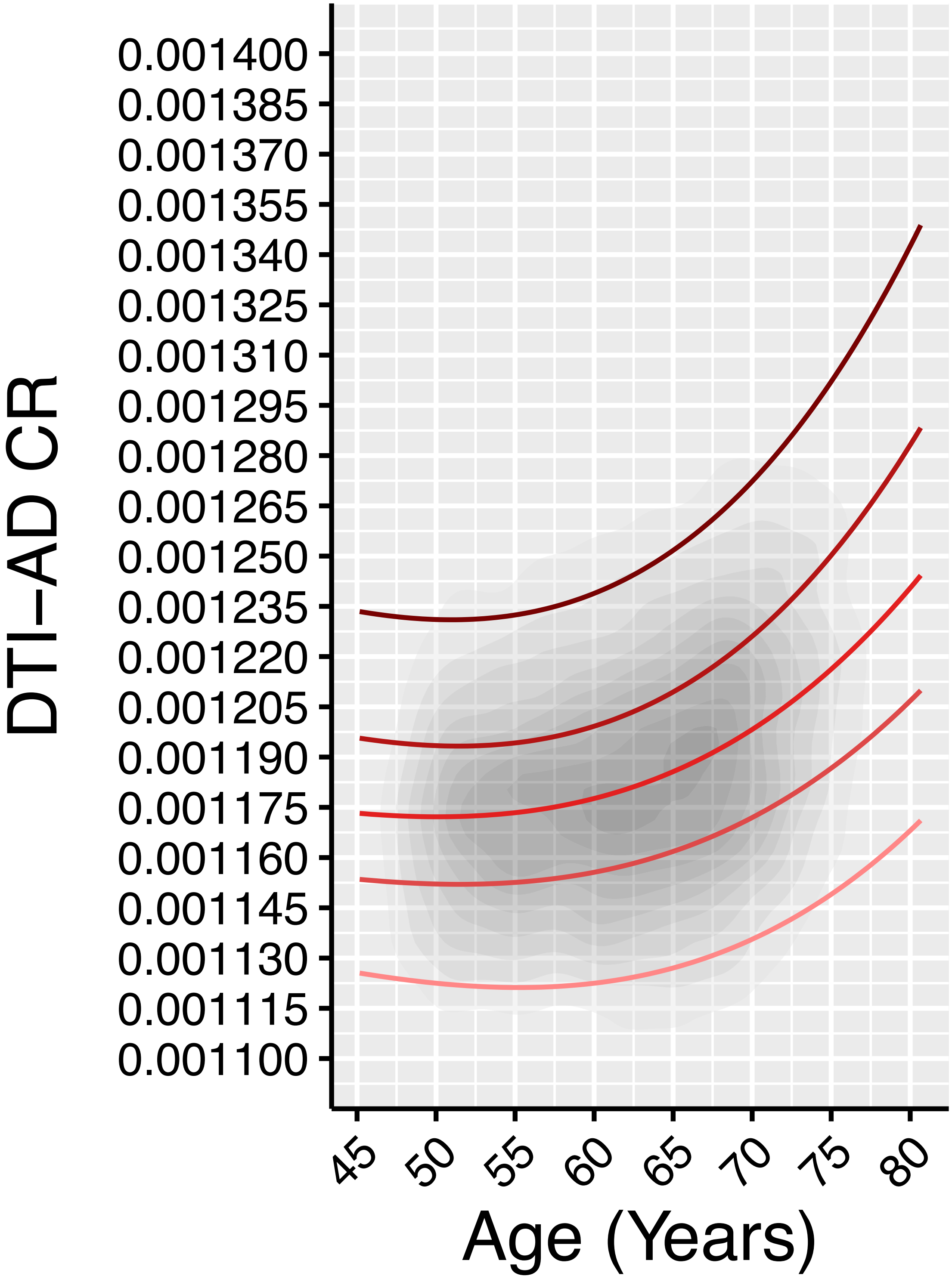

Figure S117. Full size normative centile reference curves calculated for the *corona radiata* for DTI-AD in females. Solid colored lines, ordered from lightest to darkest, indicate the following centiles: 5th, 25th, 50th, 75th, 95th. Gray overlay reflects kernel density (darker=greater degree of data point overlap). CR = *corona radiata*.

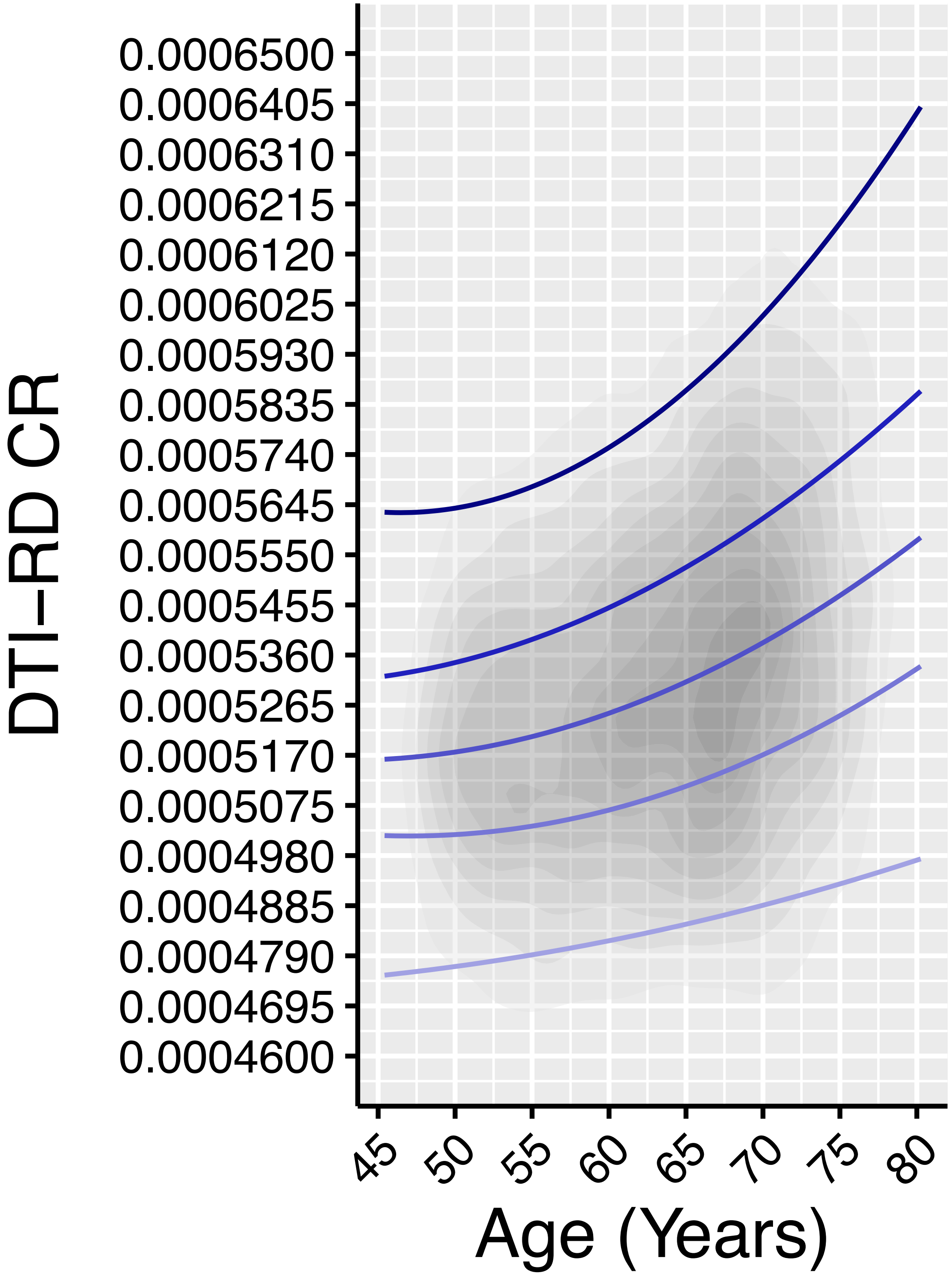

Figure S118. Full size normative centile reference curves calculated for the *corona radiata* for DTI-RD in males. Solid colored lines, ordered from lightest to darkest, indicate the following centiles: 5th, 25th, 50th, 75th, 95th. Gray overlay reflects kernel density (darker=greater degree of data point overlap). CR = *corona radiata*.

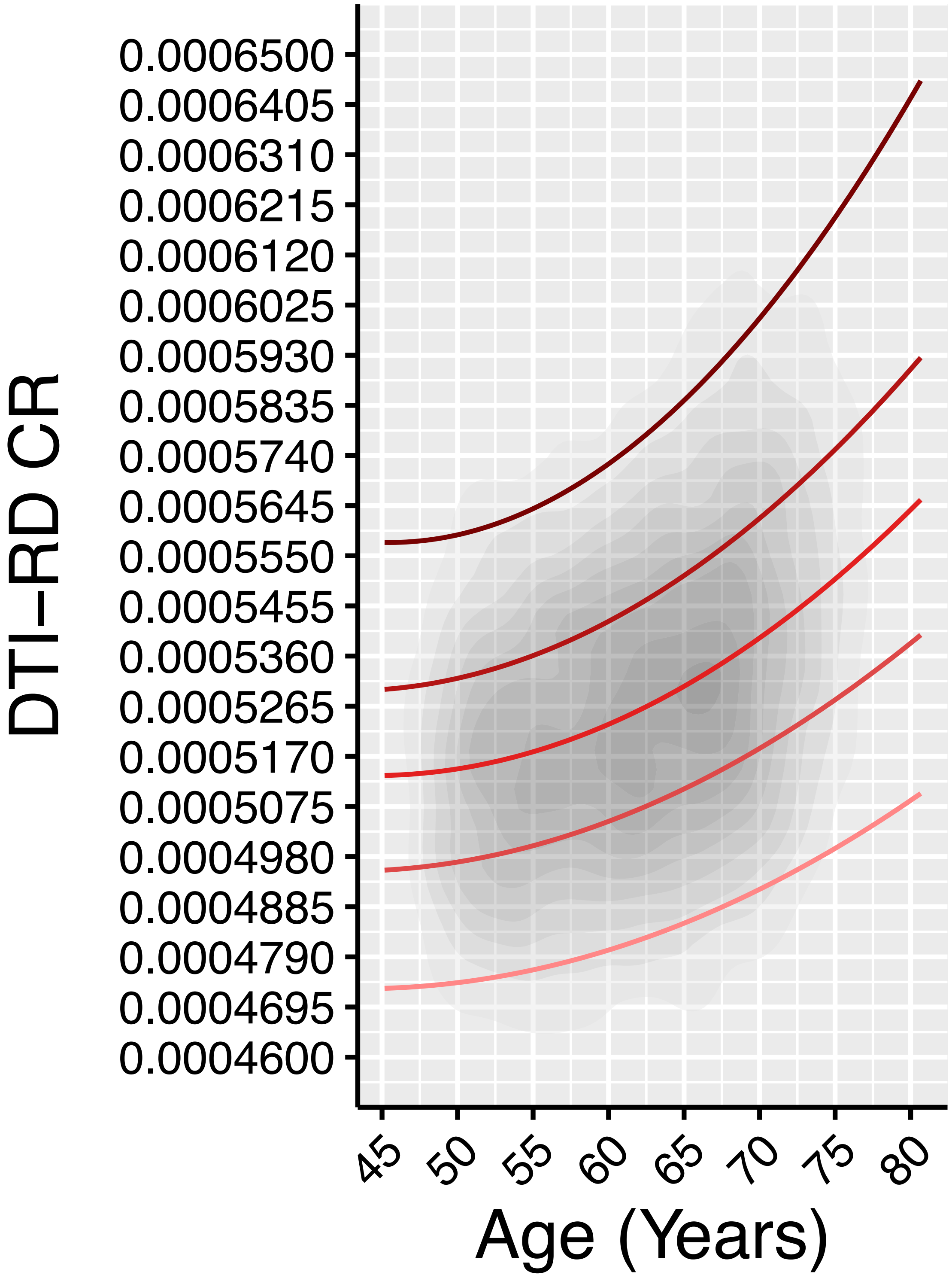

Figure S119. Full size normative centile reference curves calculated for the *corona radiata* for DTI-RD in females. Solid colored lines, ordered from lightest to darkest, indicate the following centiles: 5th, 25th, 50th, 75th, 95th. Gray overlay reflects kernel density (darker=greater degree of data point overlap). CR = *corona radiata*.

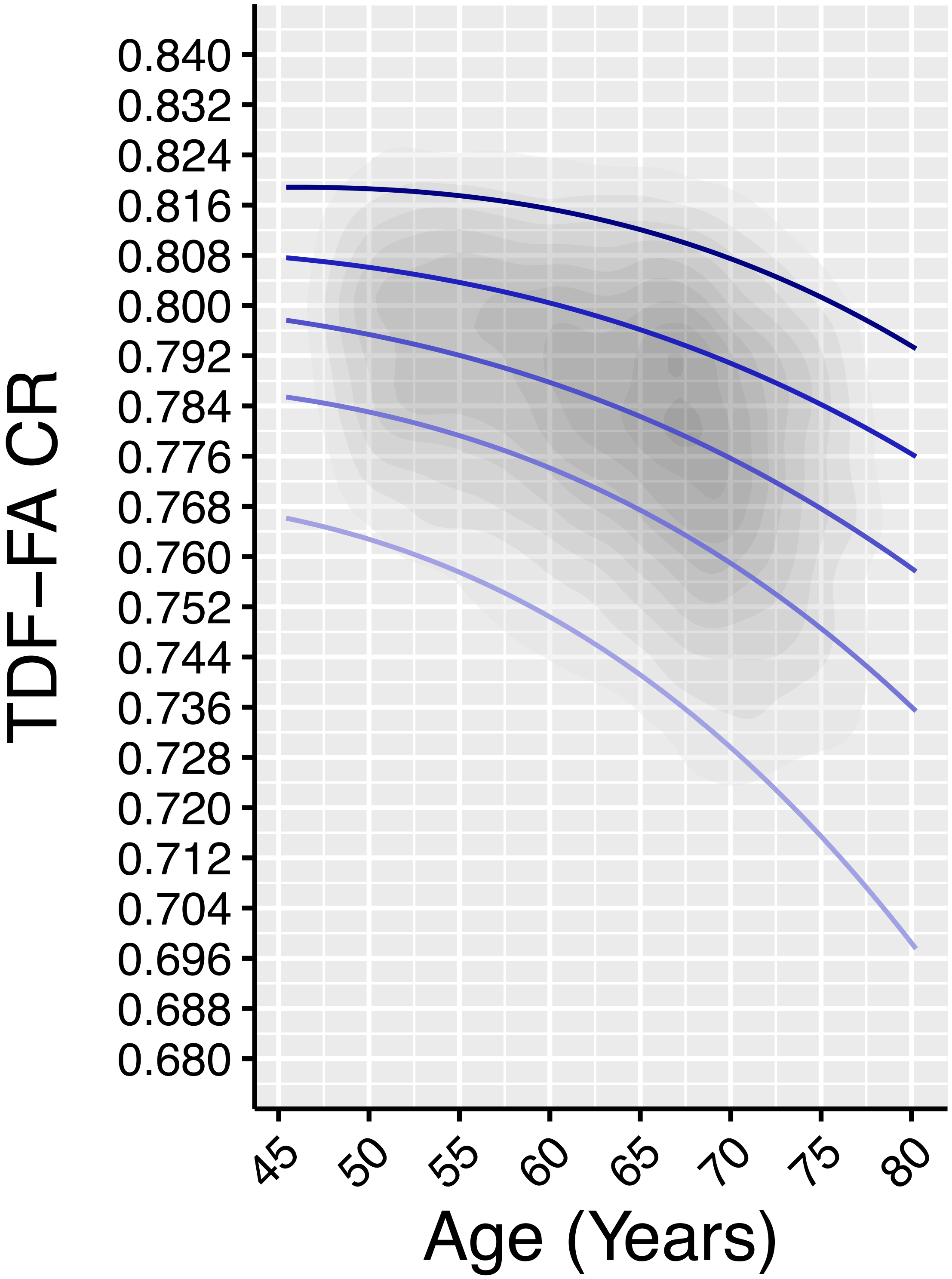

Figure S120. Full size normative centile reference curves calculated for the *corona radiata* for TDF-FA in males. Solid colored lines, ordered from lightest to darkest, indicate the following centiles: 5th, 25th, 50th, 75th, 95th. Gray overlay reflects kernel density (darker=greater degree of data point overlap). CR = *corona radiata***.**

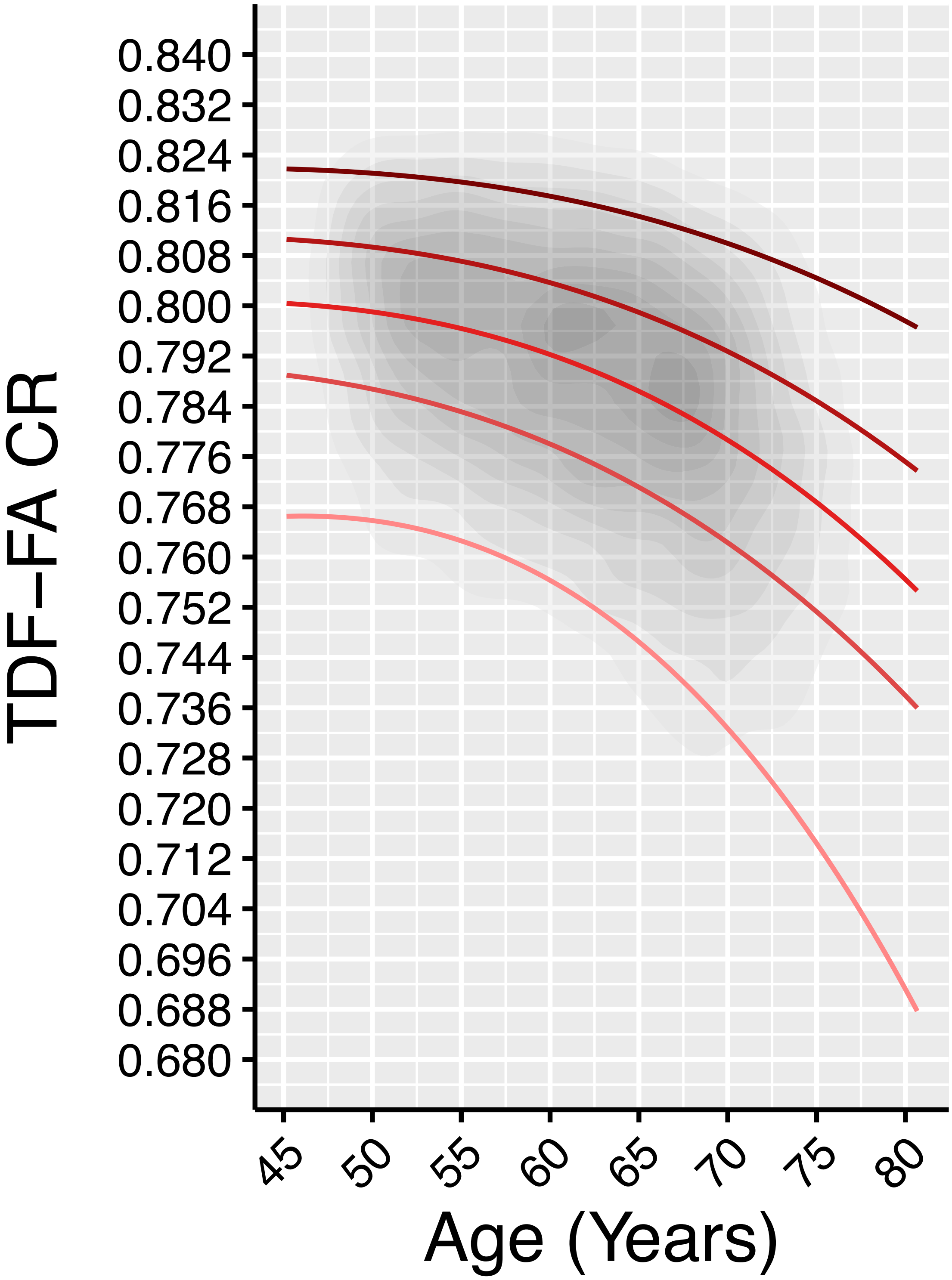

Figure S121. Full size normative centile reference curves calculated for the *corona radiata* for TDF-FA in females. Solid colored lines, ordered from lightest to darkest, indicate the following centiles: 5th, 25th, 50th, 75th, 95th. Gray overlay reflects kernel density (darker=greater degree of data point overlap). CR = *corona radiata*.

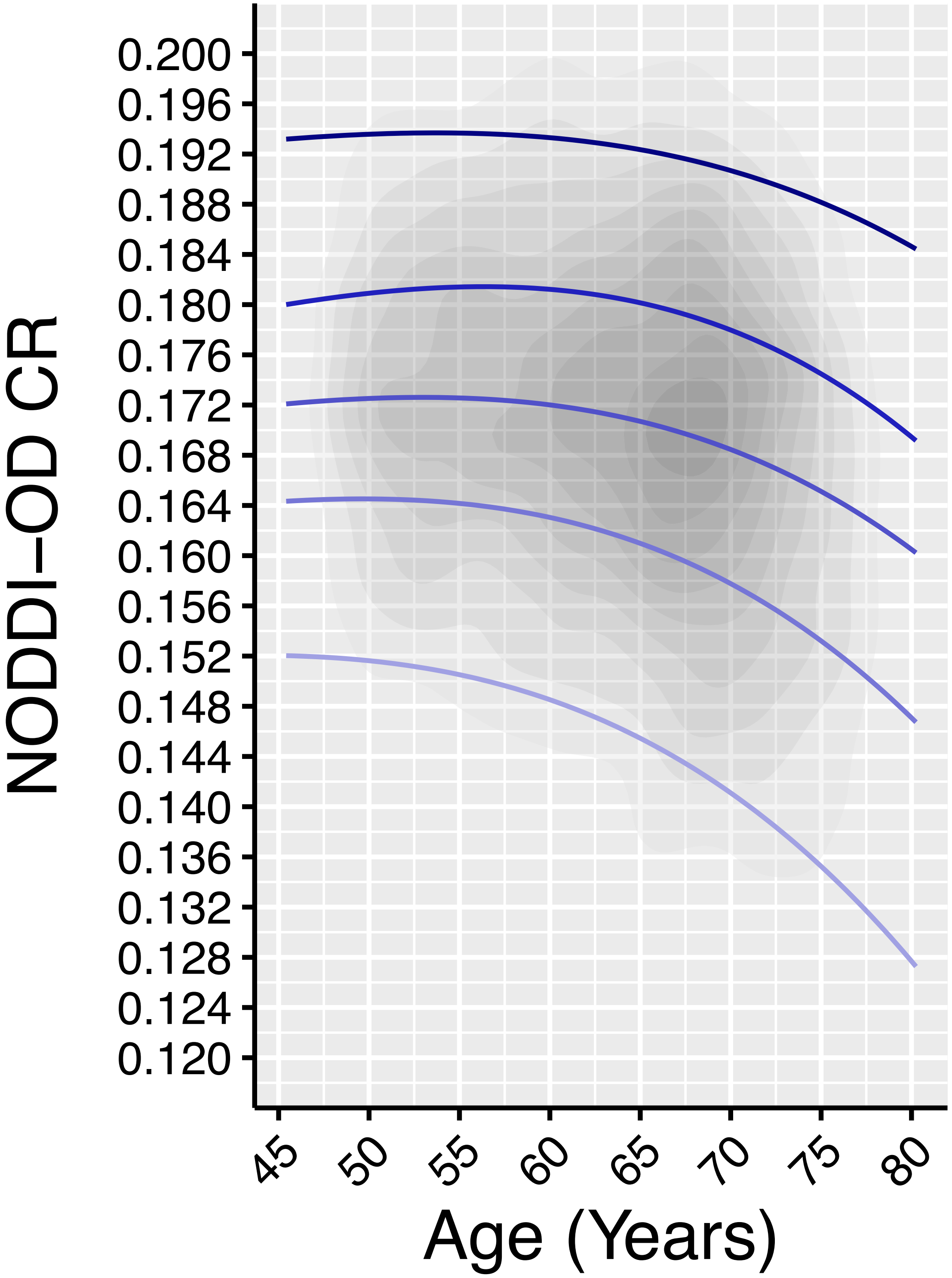

Figure S122. Full size normative centile reference curves calculated for the *corona radiata* for NODDI-OD in males. Solid colored lines, ordered from lightest to darkest, indicate the following centiles: 5th, 25th, 50th, 75th, 95th. Gray overlay reflects kernel density (darker=greater degree of data point overlap). CR = *corona radiata***.**

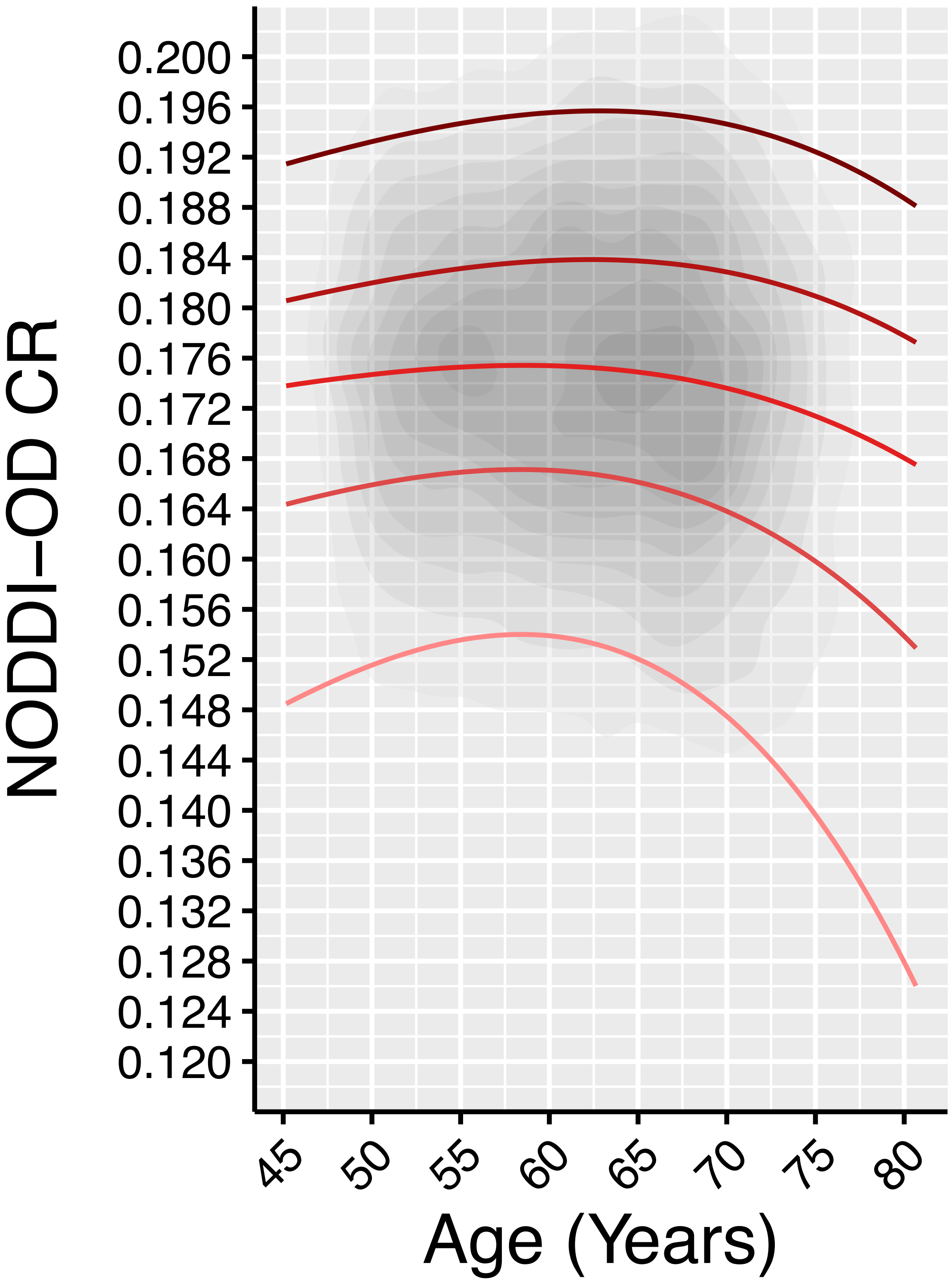

Figure S123. Full size normative centile reference curves calculated for the *corona radiata* for NODDI-OD in females. Solid colored lines, ordered from lightest to darkest, indicate the following centiles: 5th, 25th, 50th, 75th, 95th. Gray overlay reflects kernel density (darker=greater degree of data point overlap). CR = *corona radiata*.

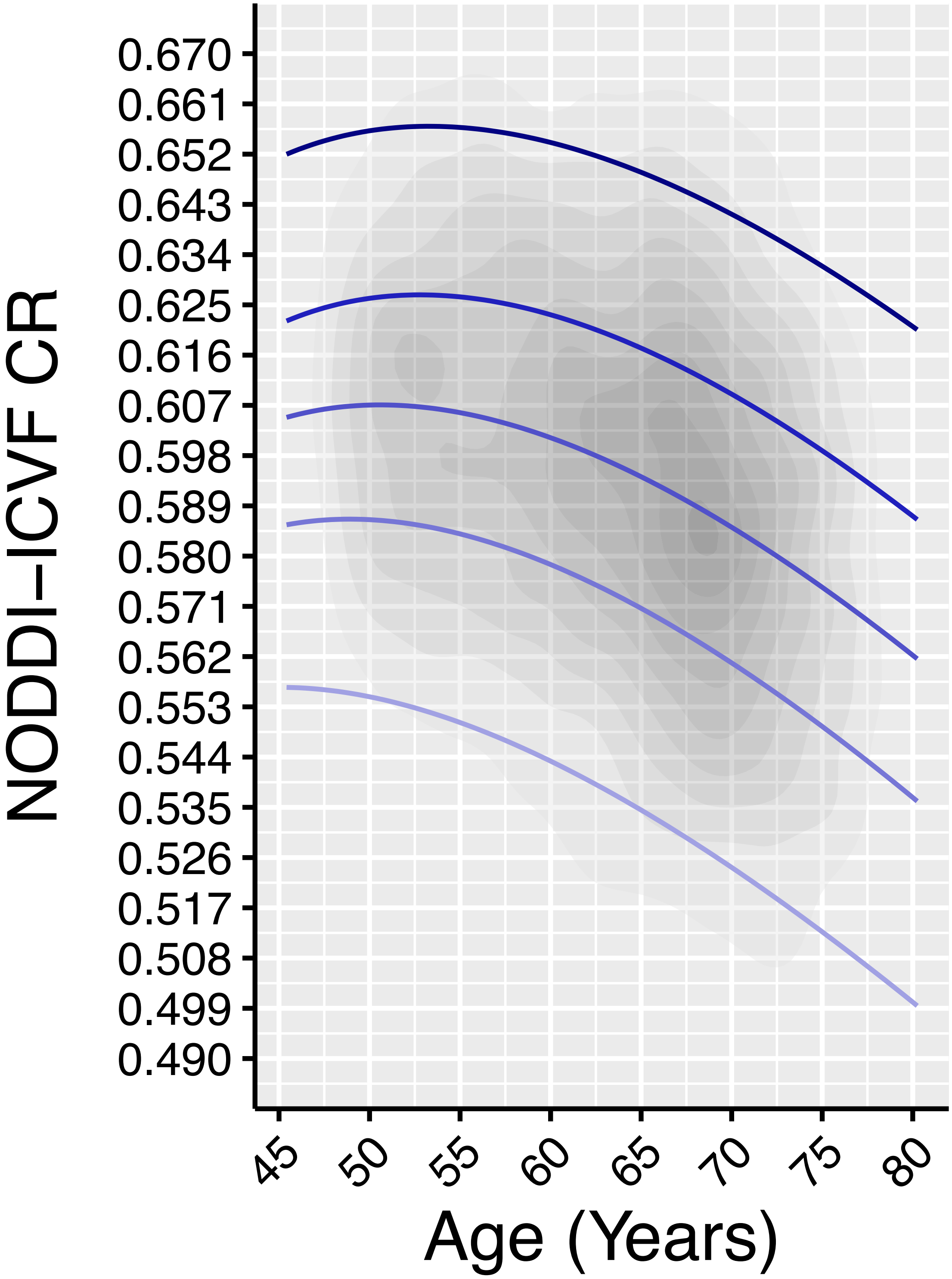

Figure S124. Full size normative centile reference curves calculated for the *corona radiata* for NODDI-ICVF in males. Solid colored lines, ordered from lightest to darkest, indicate the following centiles: 5th, 25th, 50th, 75th, 95th. Gray overlay reflects kernel density (darker=greater degree of data point overlap). CR = *corona radiata*.

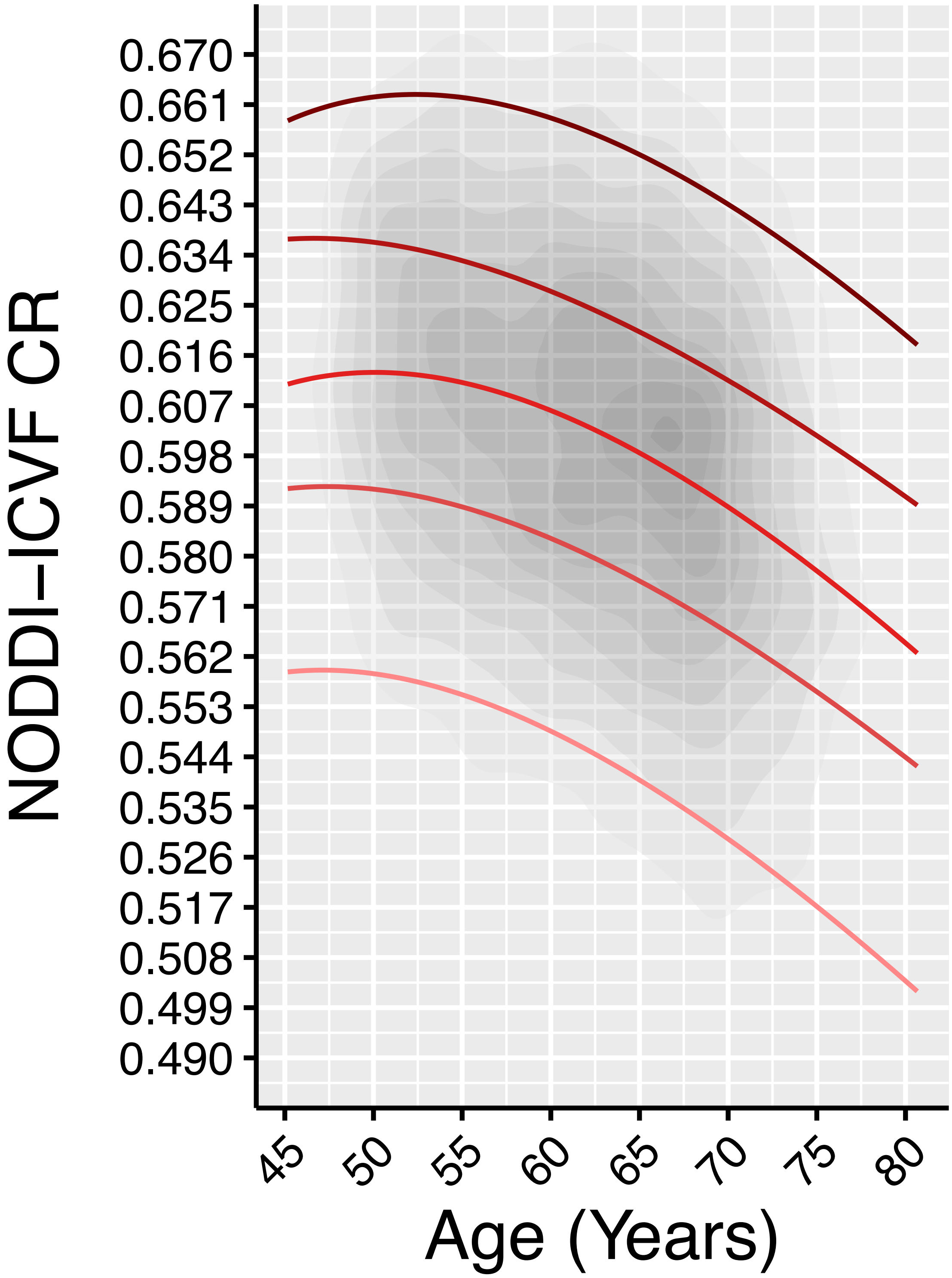

Figure S125. Full size normative centile reference curves calculated for the *corona radiata* for NODDI-ICVF in females. Solid colored lines, ordered from lightest to darkest, indicate the following centiles: 5th, 25th, 50th, 75th, 95th. Gray overlay reflects kernel density (darker=greater degree of data point overlap). CR = *corona radiata***.**

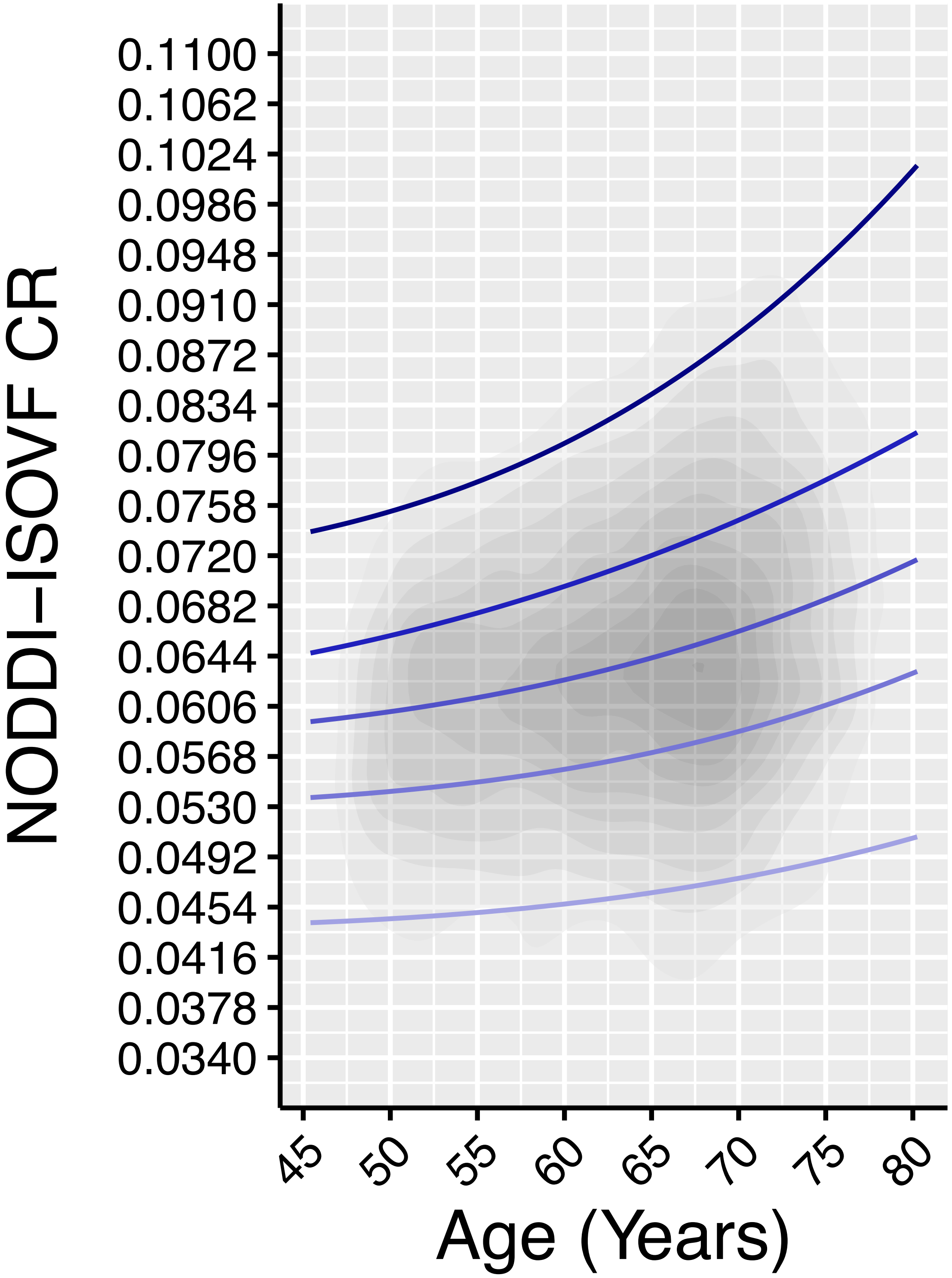

Figure S126. Full size normative centile reference curves calculated for the *corona radiata* for NODDI-ISOVF in males. Solid colored lines, ordered from lightest to darkest, indicate the following centiles: 5th, 25th, 50th, 75th, 95th. Gray overlay reflects kernel density (darker=greater degree of data point overlap). CR = *corona radiata*.

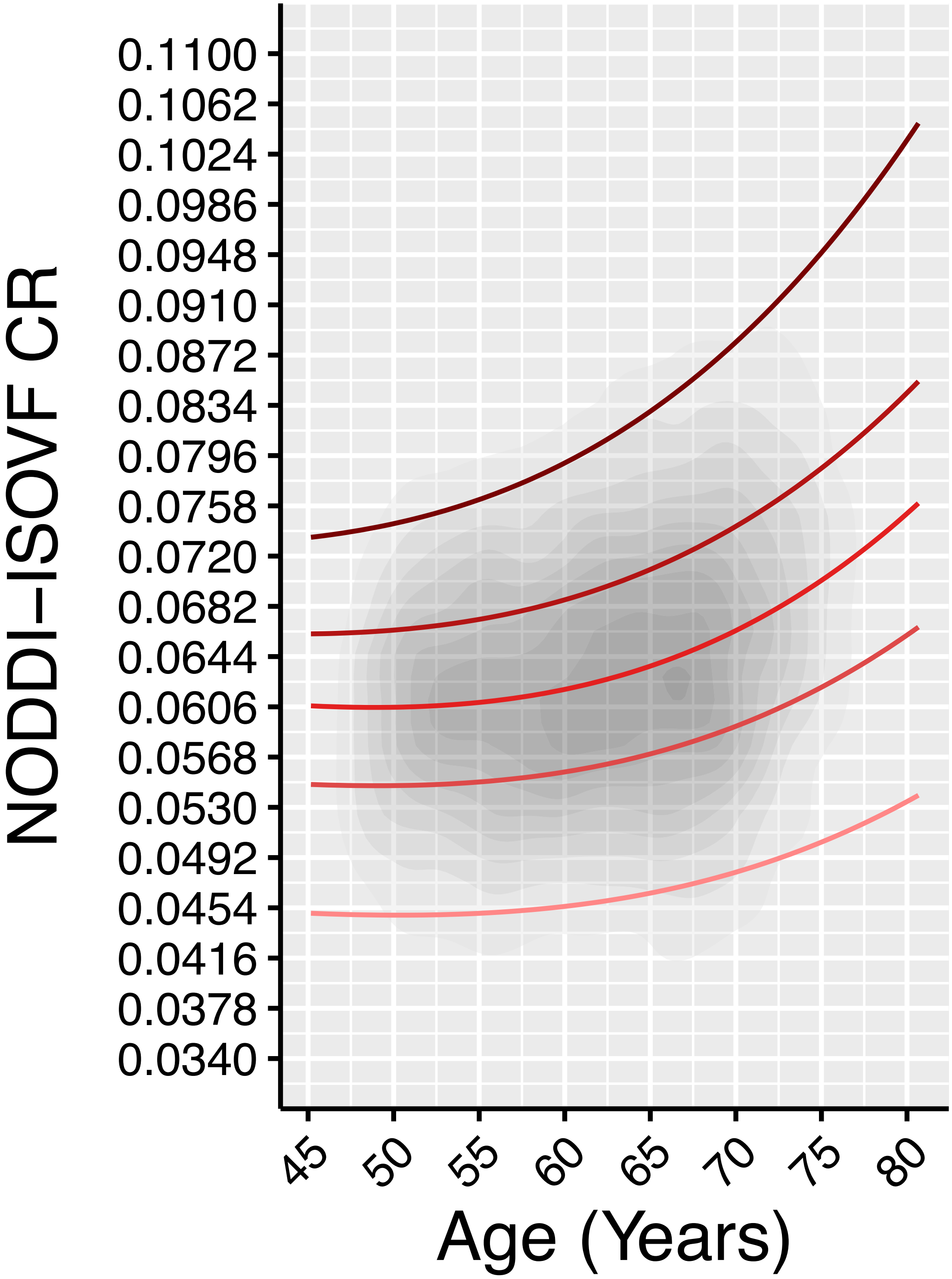

Figure S127. Full size normative centile reference curves calculated for the *corona radiata* for NODDI-ISOVF in females. Solid colored lines, ordered from lightest to darkest, indicate the following centiles: 5th, 25th, 50th, 75th, 95th. Gray overlay reflects kernel density (darker=greater degree of data point overlap). CR = *corona radiata***.**

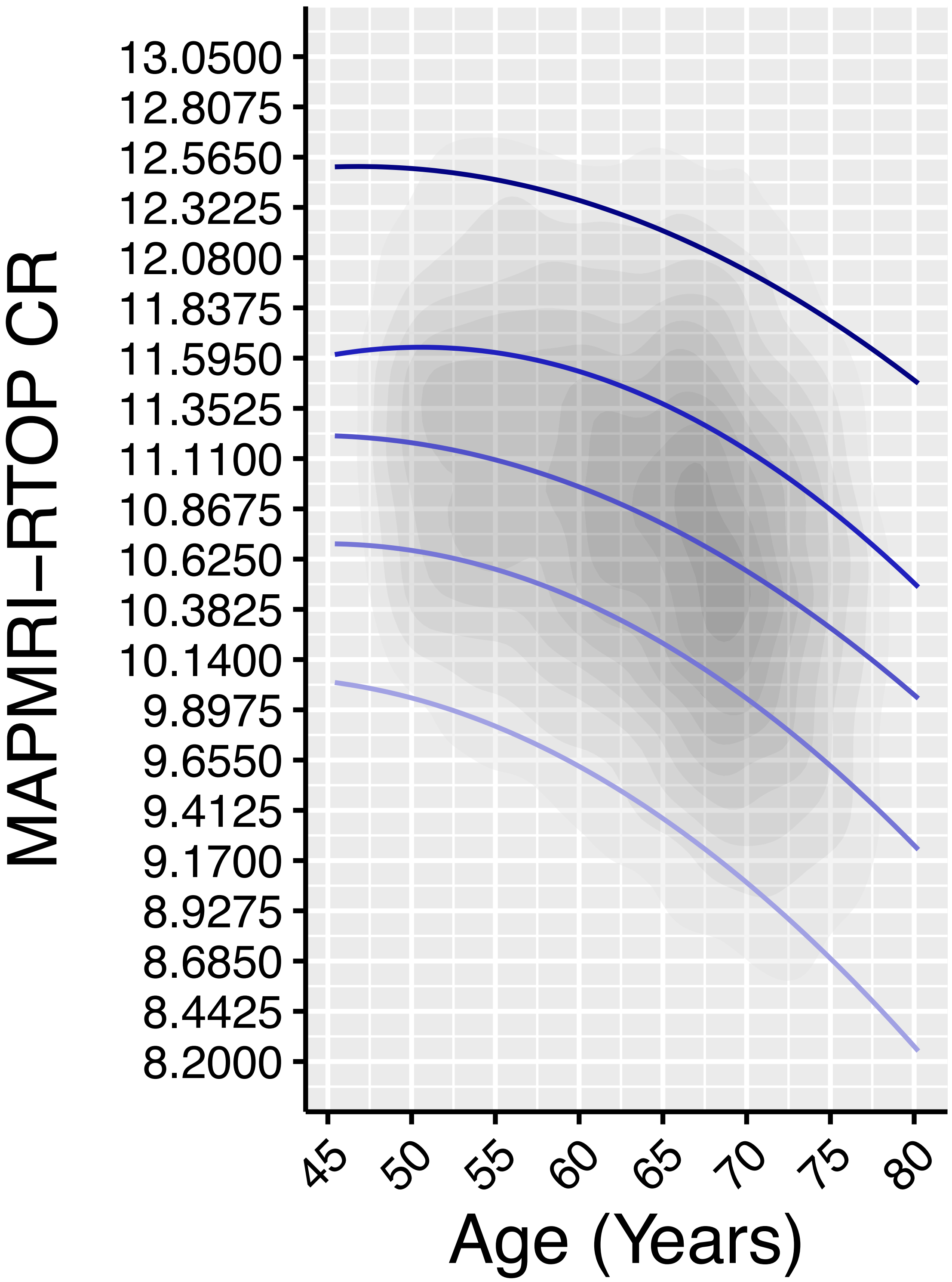

Figure S128. Full size normative centile reference curves calculated for the *corona radiata* for MAPMRI-RTOP in males. Solid colored lines, ordered from lightest to darkest, indicate the following centiles: 5th, 25th, 50th, 75th, 95th. Gray overlay reflects kernel density (darker=greater degree of data point overlap). CR = *corona radiata*.

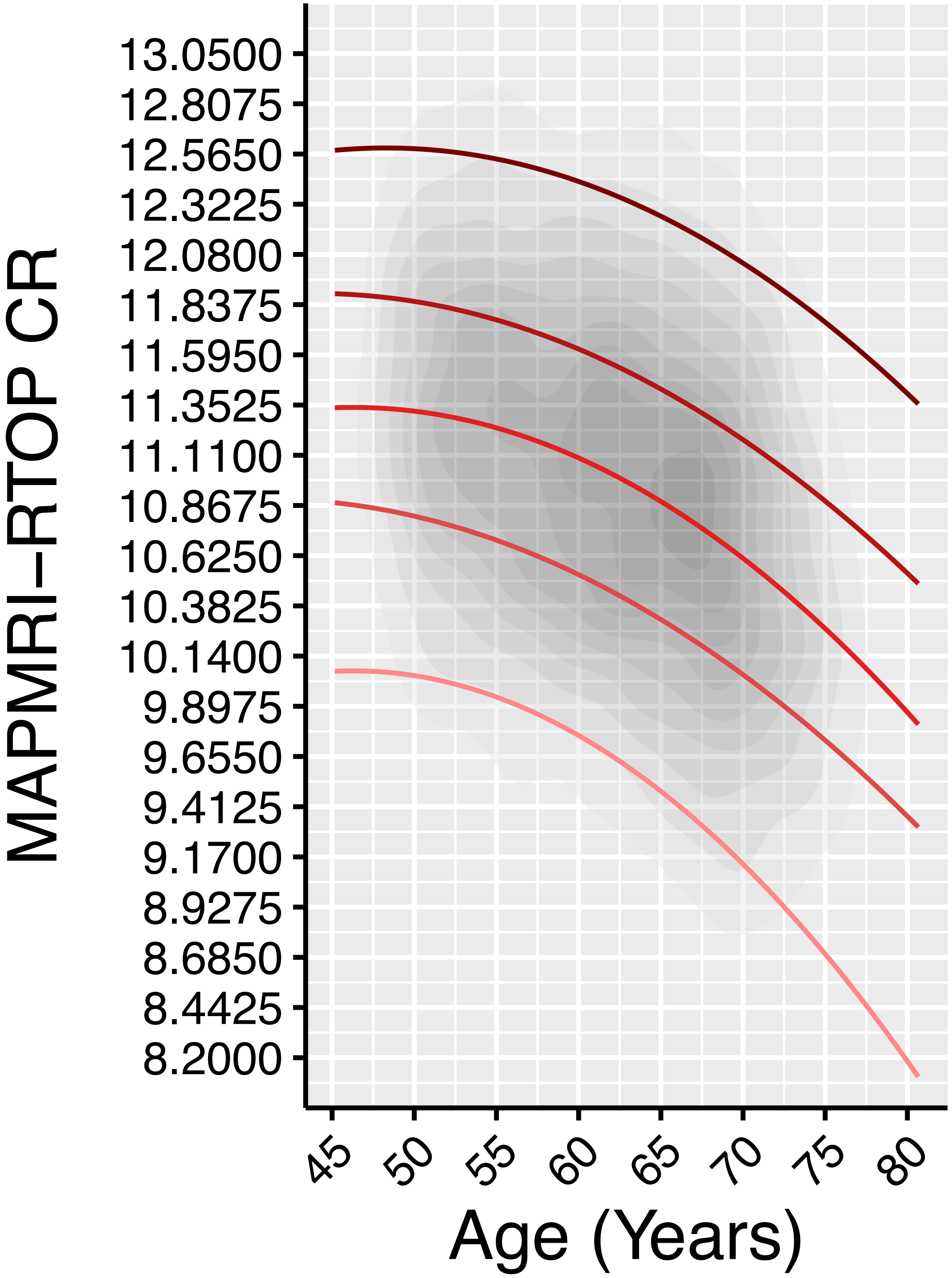

Figure S129. Full size normative centile reference curves calculated for the *corona radiata* for MAPMRI-RTOP in females. Solid colored lines, ordered from lightest to darkest, indicate the following centiles: 5th, 25th, 50th, 75th, 95th. Gray overlay reflects kernel density (darker=greater degree of data point overlap). CR = *corona radiata*.

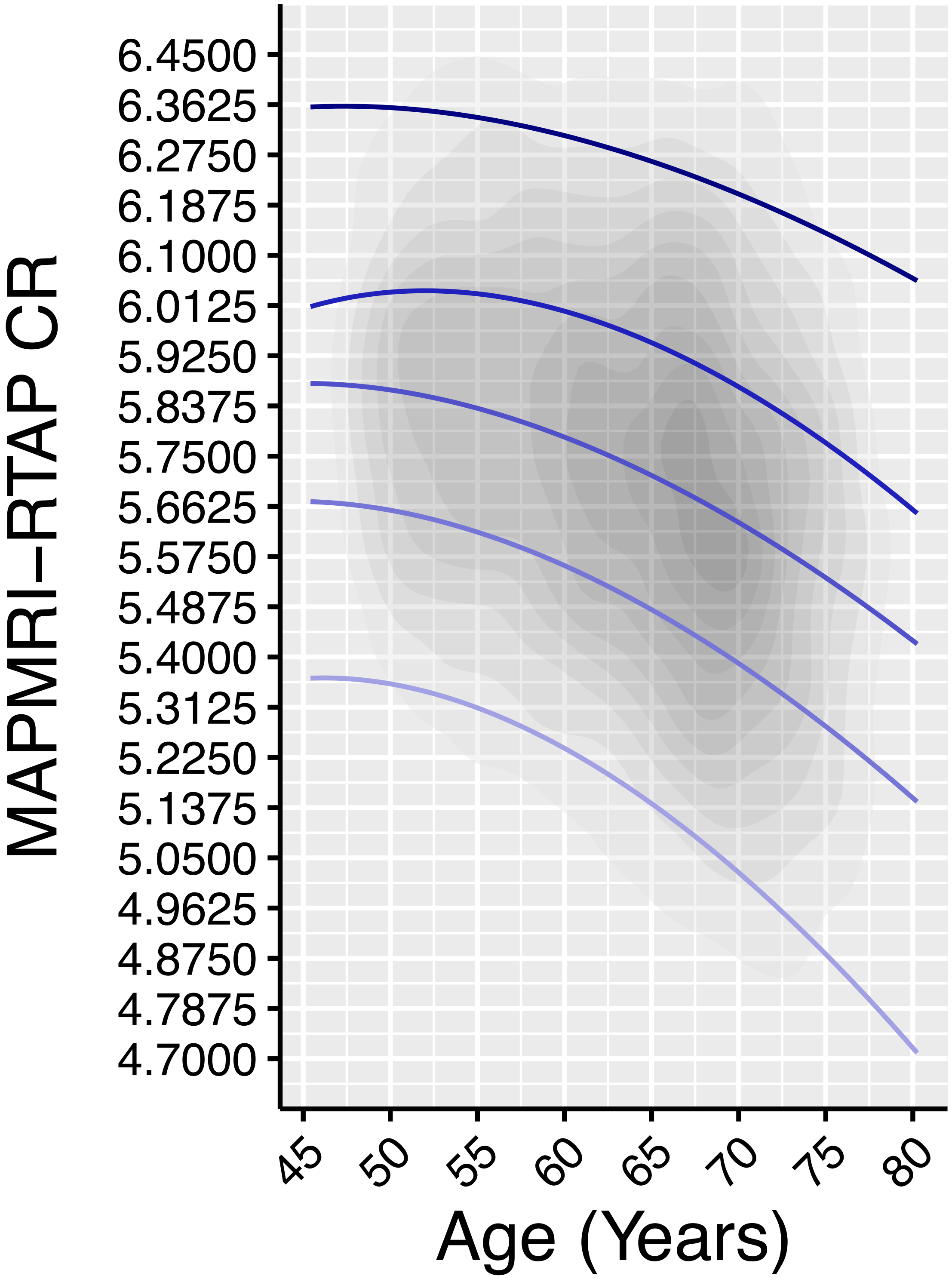

Figure S130. Full size normative centile reference curves calculated for the *corona radiata* for MAPMRI-RTAP in males. Solid colored lines, ordered from lightest to darkest, indicate the following centiles: 5th, 25th, 50th, 75th, 95th. Gray overlay reflects kernel density (darker=greater degree of data point overlap). CR = *corona radiata*.

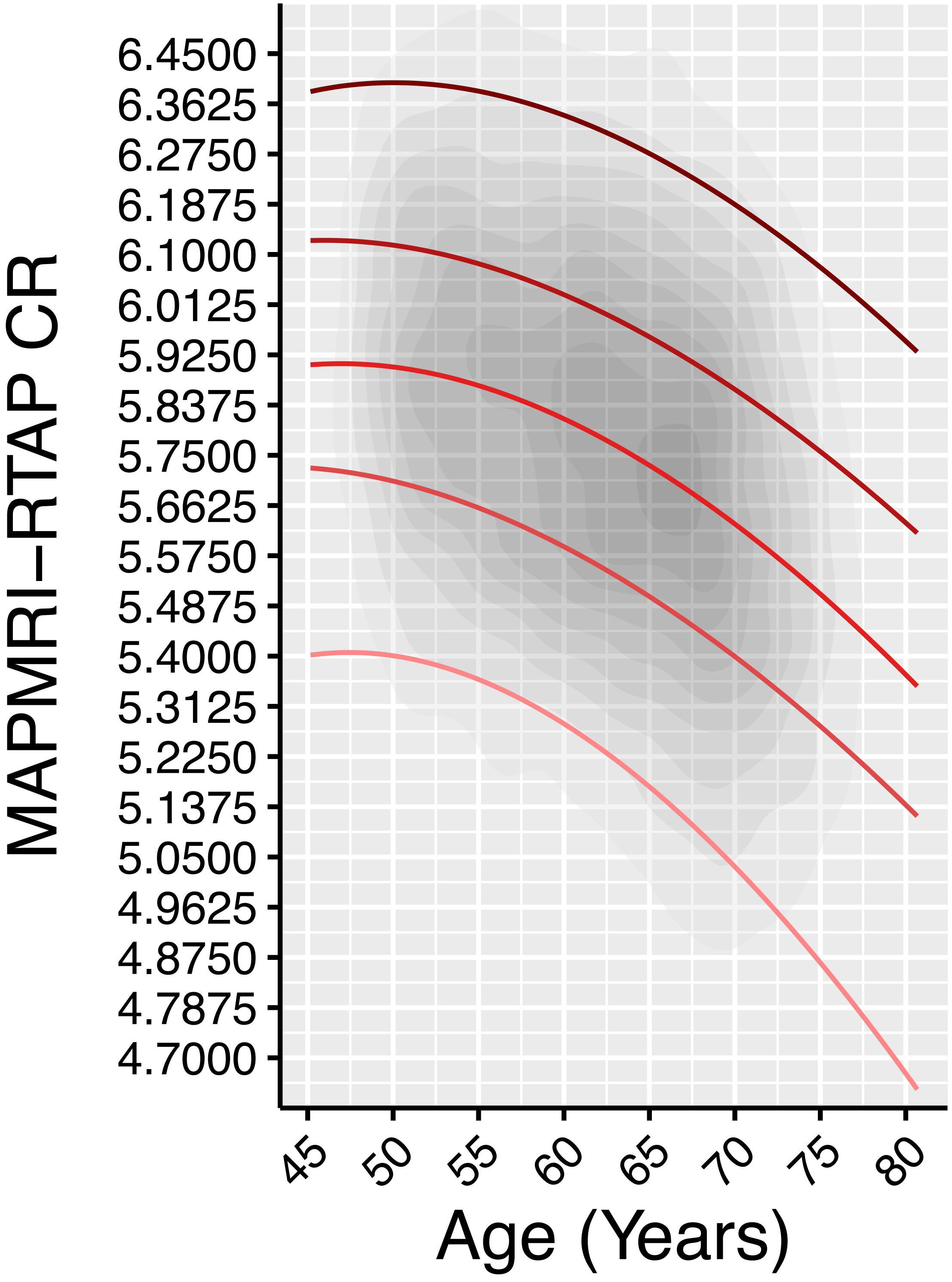

Figure S131. Full size normative centile reference curves calculated for the *corona radiata* for MAPMRI-RTAP in females. Solid colored lines, ordered from lightest to darkest, indicate the following centiles: 5th, 25th, 50th, 75th, 95th. Gray overlay reflects kernel density (darker=greater degree of data point overlap). CR = *corona radiata*.

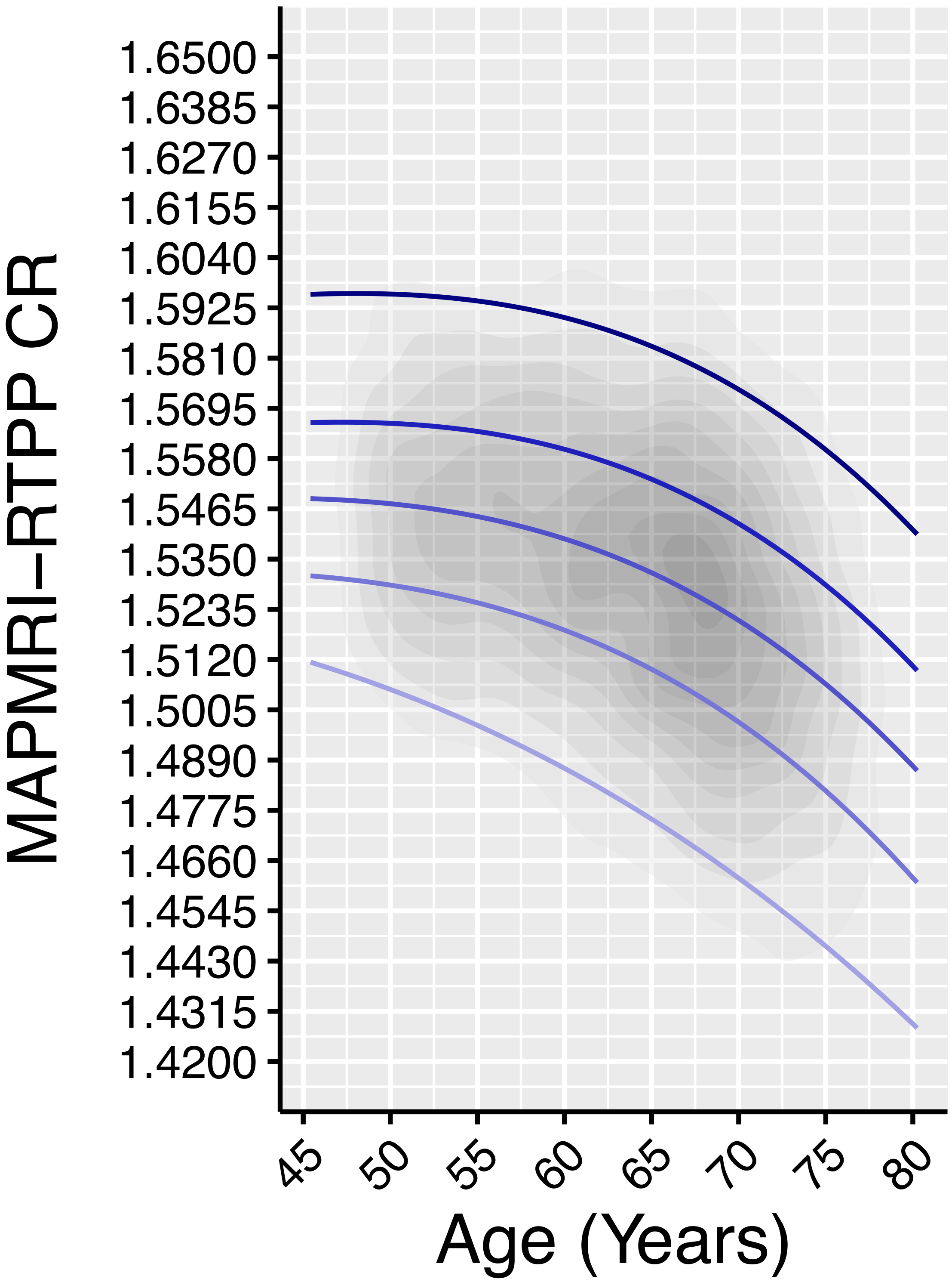

Figure S132. Full size normative centile reference curves calculated for the *corona radiata* for MAPMRI-RTPP in males. Solid colored lines, ordered from lightest to darkest, indicate the following centiles: 5th, 25th, 50th, 75th, 95th. Gray overlay reflects kernel density (darker=greater degree of data point overlap). CR = *corona radiata***.**

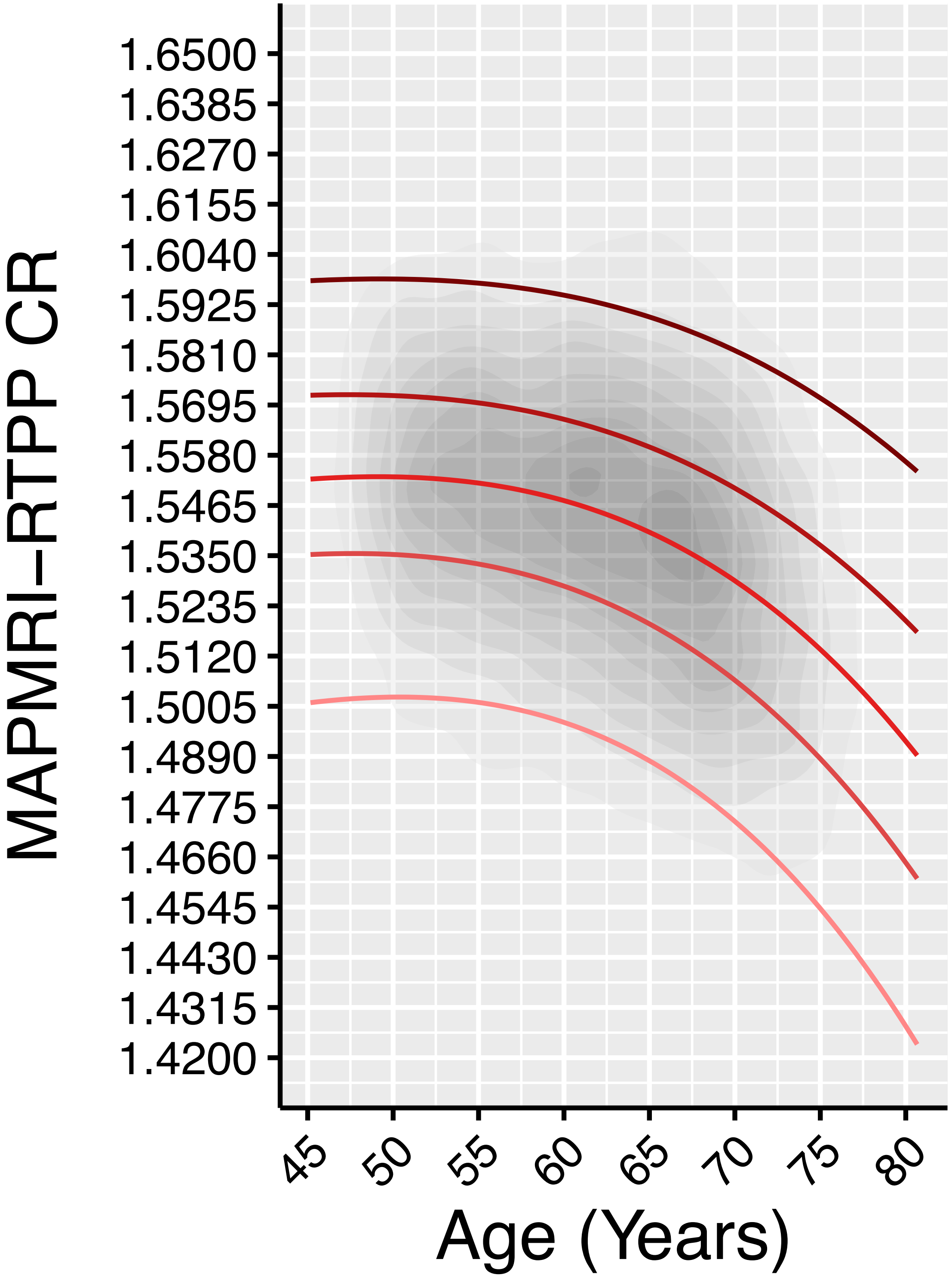

Figure S133. Full size normative centile reference curves calculated for the *corona radiata* for MAPMRI-RTPP in females. Solid colored lines, ordered from lightest to darkest, indicate the following centiles: 5th, 25th, 50th, 75th, 95th. Gray overlay reflects kernel density (darker=greater degree of data point overlap). CR = *corona radiata*

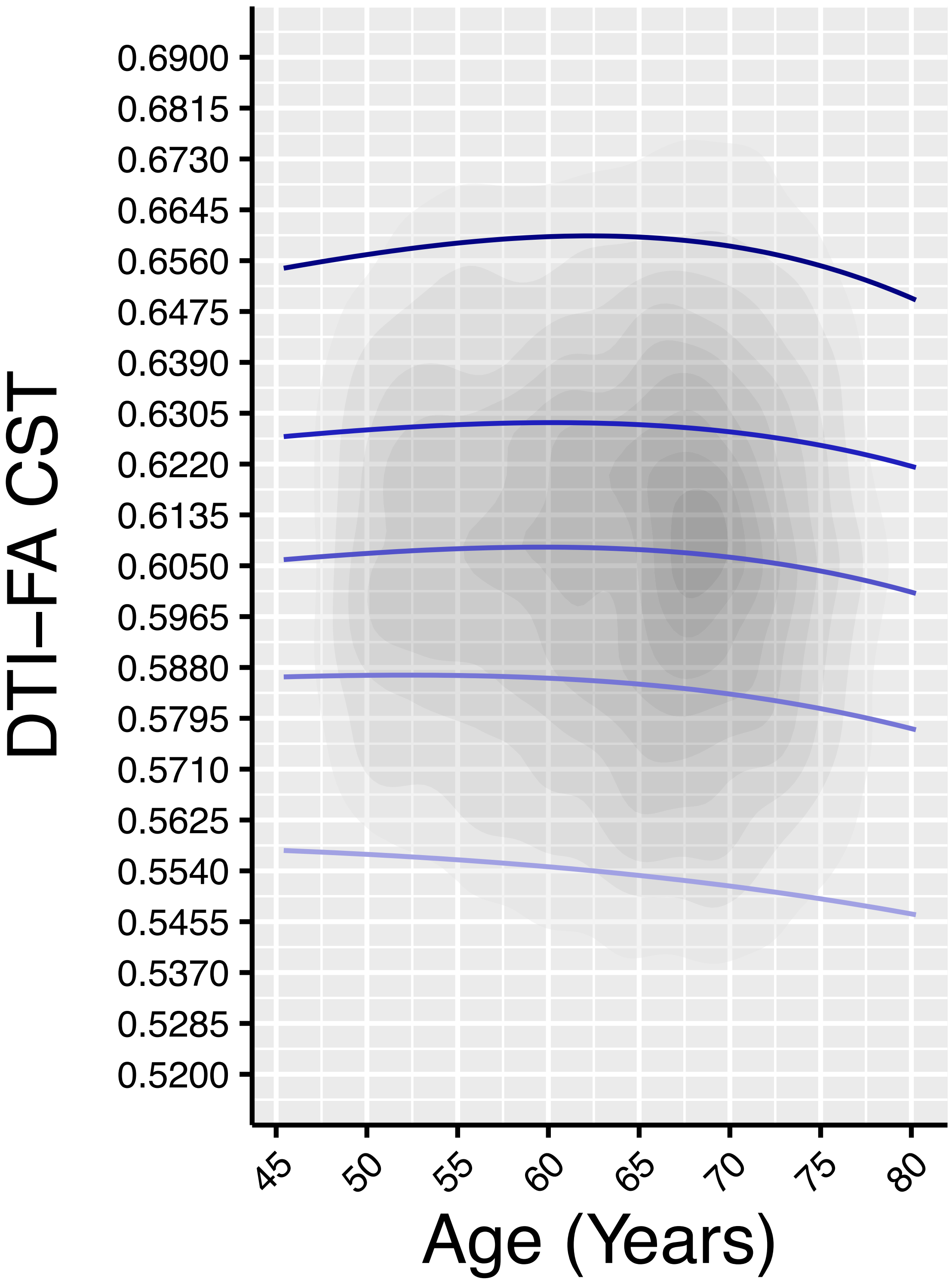

Figure S134. Full size normative centile reference curves calculated for the corticospinal tract for DTI-FA in males. Solid colored lines, ordered from lightest to darkest, indicate the following centiles: 5th, 25th, 50th, 75th, 95th. Gray overlay reflects kernel density (darker=greater degree of data point overlap). CST = corticospinal tract.

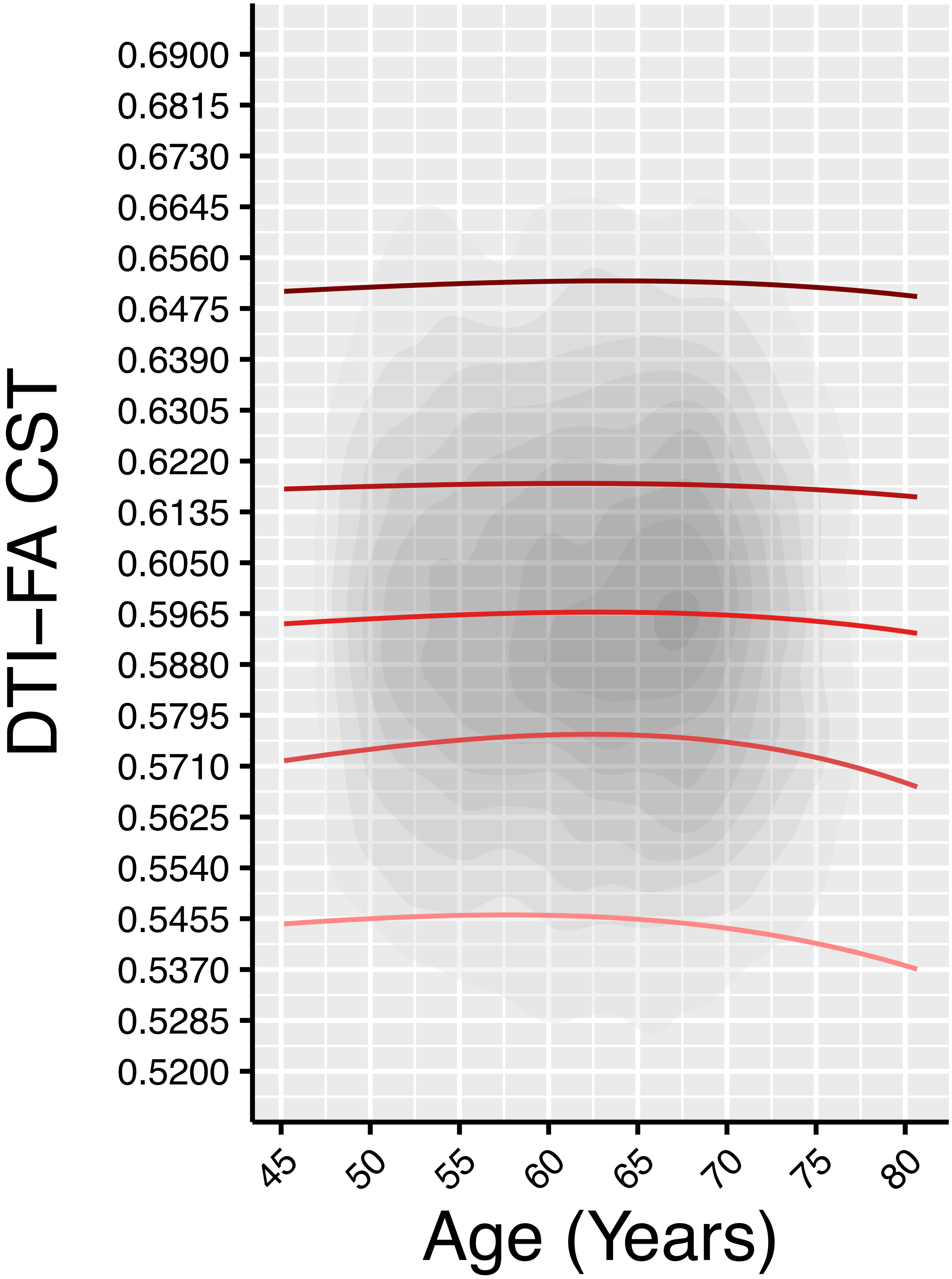

Figure S135. Full size normative centile reference curves calculated for the corticospinal tract for DTI-FA in females. Solid colored lines, ordered from lightest to darkest, indicate the following centiles: 5th, 25th, 50th, 75th, 95th. Gray overlay reflects kernel density (darker=greater degree of data point overlap). CST = corticospinal tract.

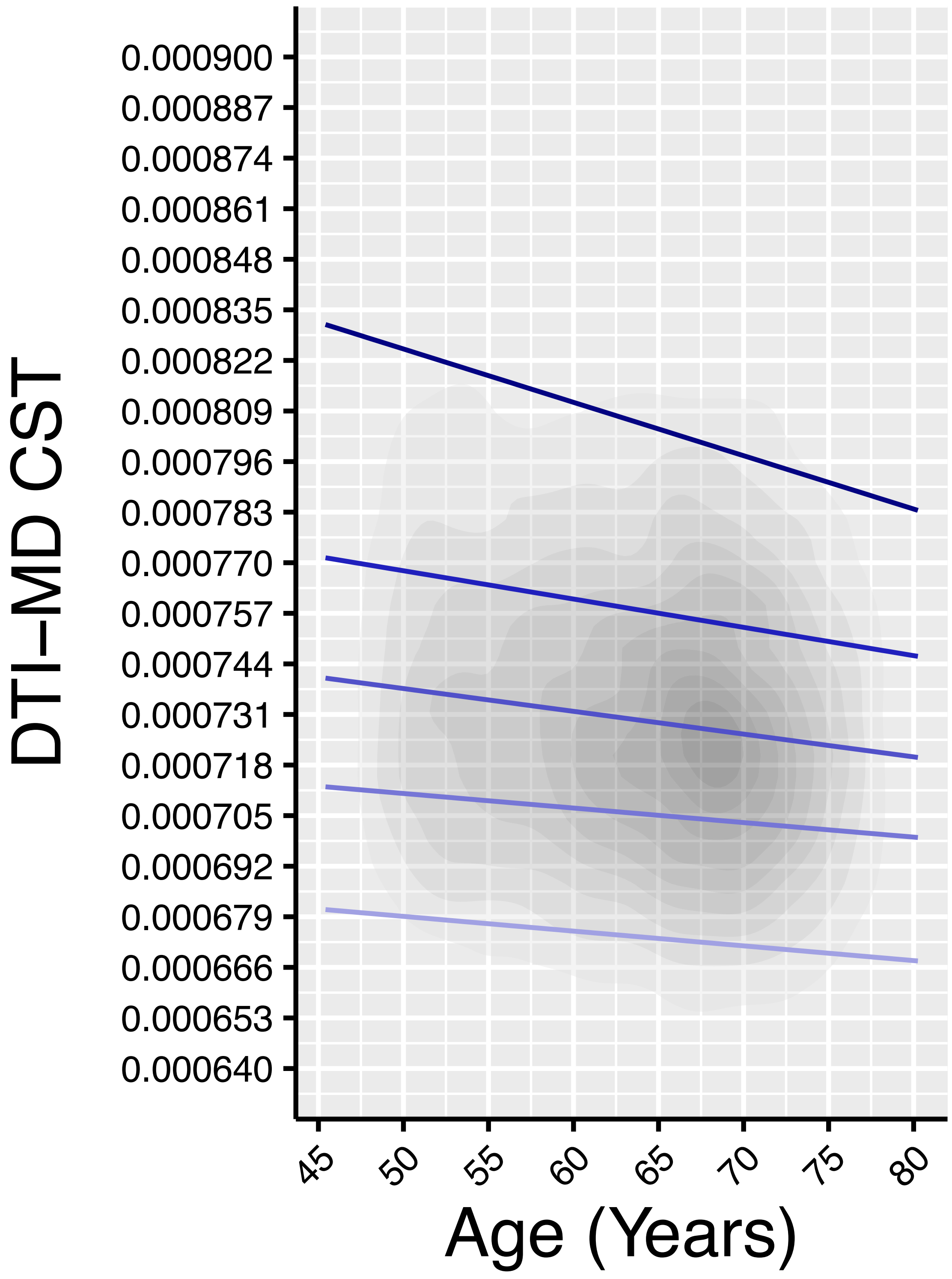

Figure S136. Full size normative centile reference curves calculated for the corticospinal tract for DTI-MD in males. Solid colored lines, ordered from lightest to darkest, indicate the following centiles: 5th, 25th, 50th, 75th, 95th. Gray overlay reflects kernel density (darker=greater degree of data point overlap). CST = corticospinal tract.

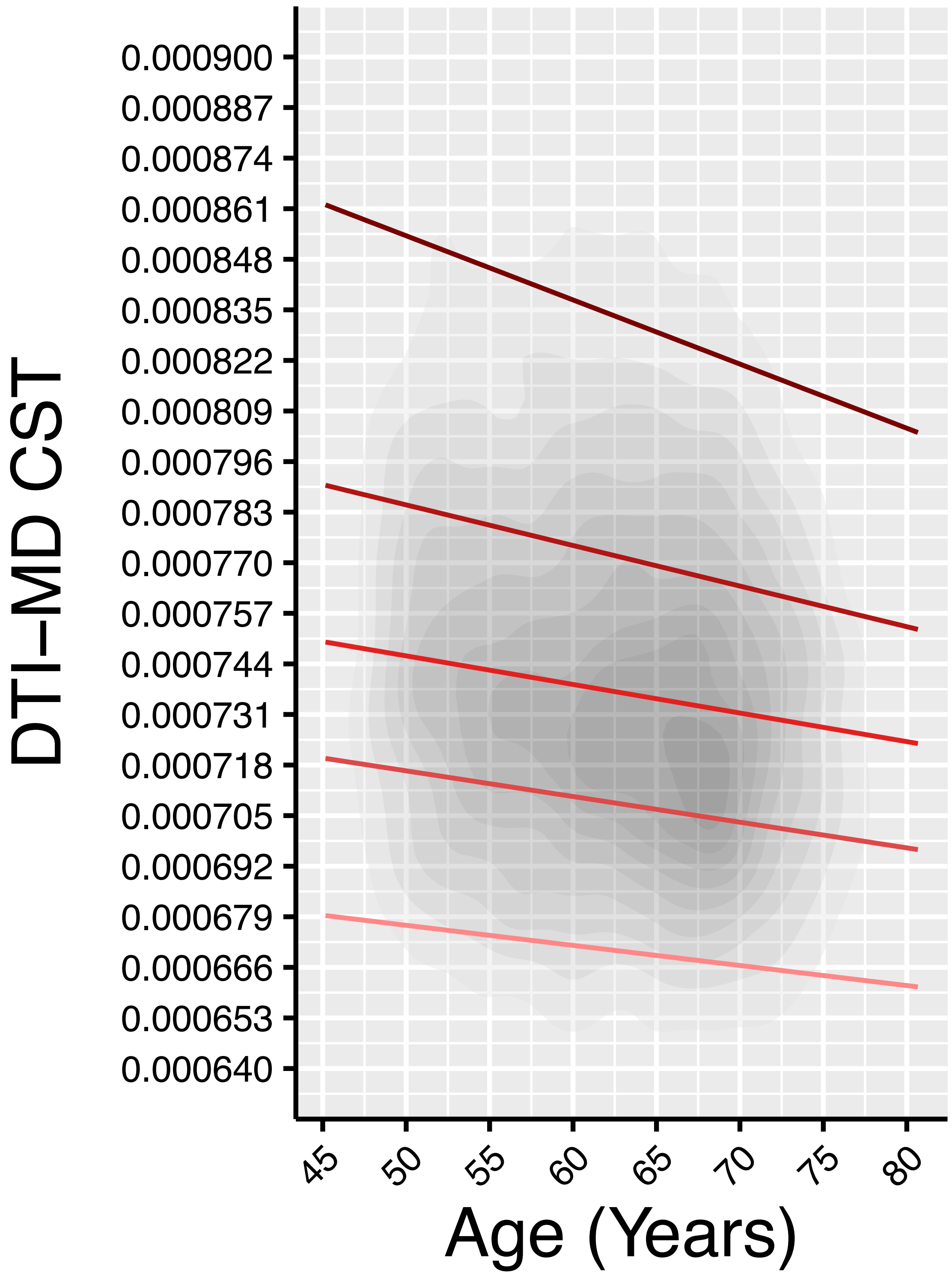

Figure S137. Full size normative centile reference curves calculated for the corticospinal tract for DTI-MD in females. Solid colored lines, ordered from lightest to darkest, indicate the following centiles: 5th, 25th, 50th, 75th, 95th. Gray overlay reflects kernel density (darker=greater degree of data point overlap). CST = corticospinal tract.

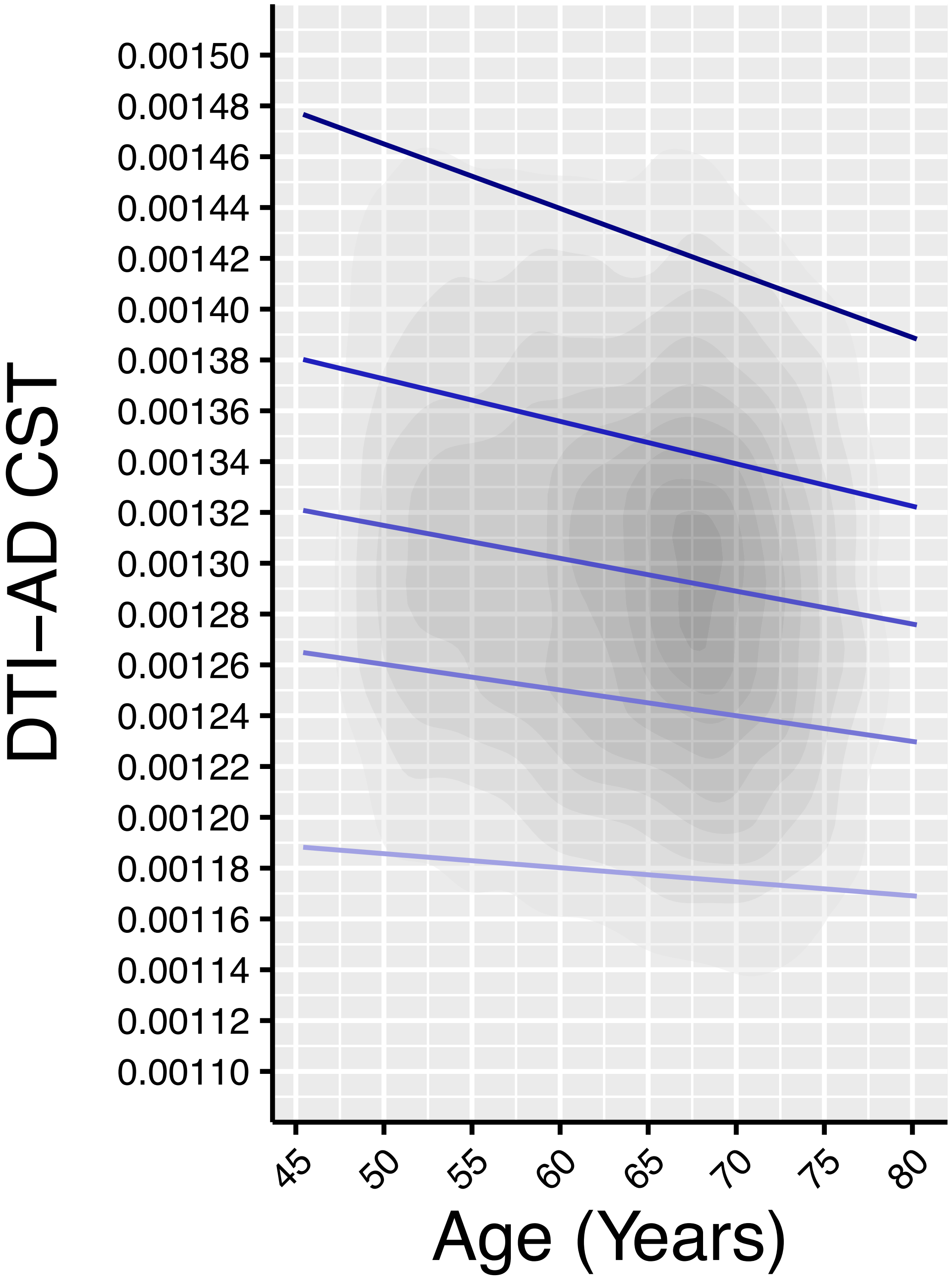

Figure S138. Full size normative centile reference curves calculated for the corticospinal tract for DTI-AD in males. Solid colored lines, ordered from lightest to darkest, indicate the following centiles: 5th, 25th, 50th, 75th, 95th. Gray overlay reflects kernel density (darker=greater degree of data point overlap). CST = corticospinal tract.

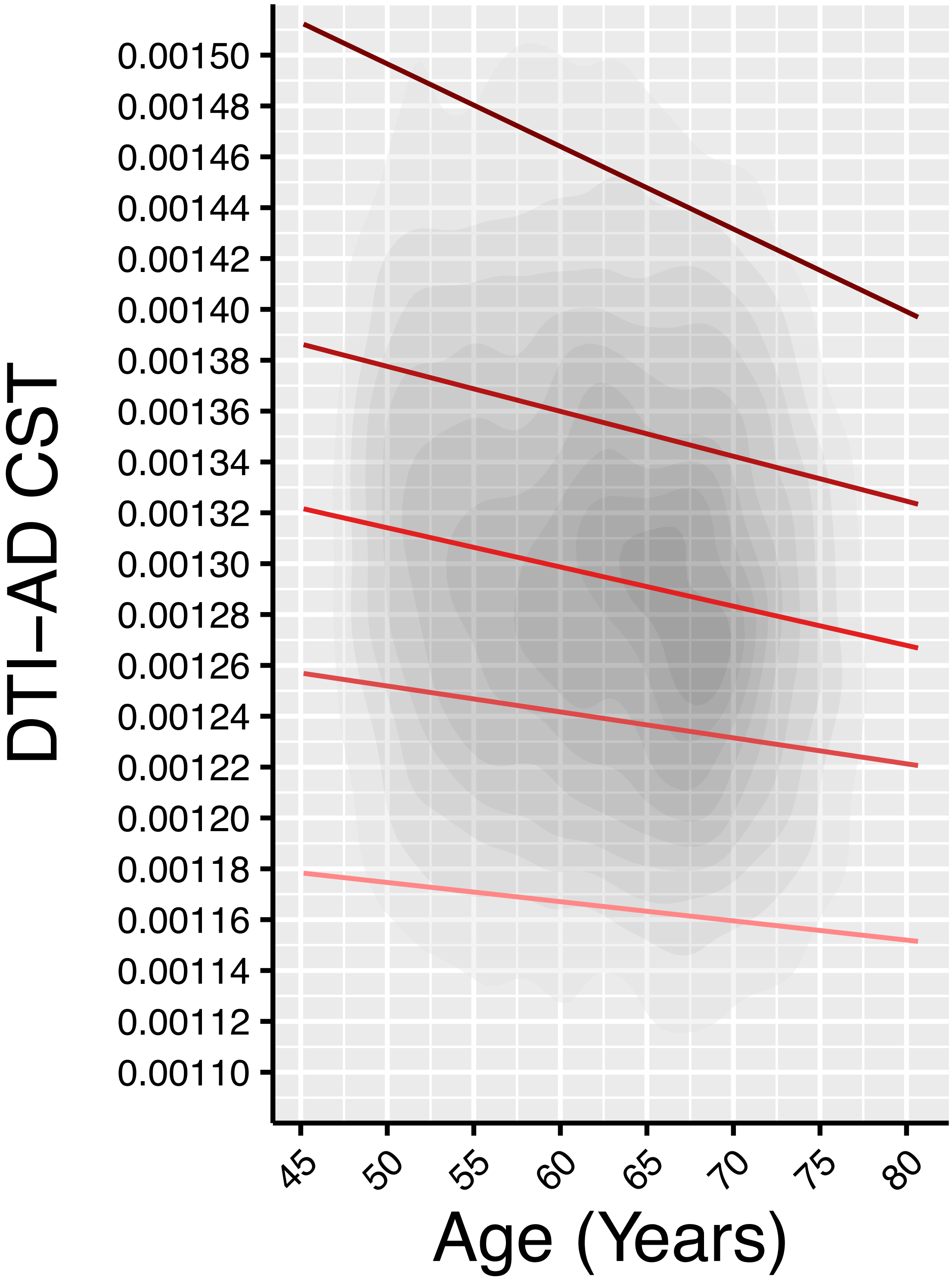

Figure S139. Full size normative centile reference curves calculated for the corticospinal tract for DTI-AD in females. Solid colored lines, ordered from lightest to darkest, indicate the following centiles: 5th, 25th, 50th, 75th, 95th. Gray overlay reflects kernel density (darker=greater degree of data point overlap). CST = corticospinal tract.

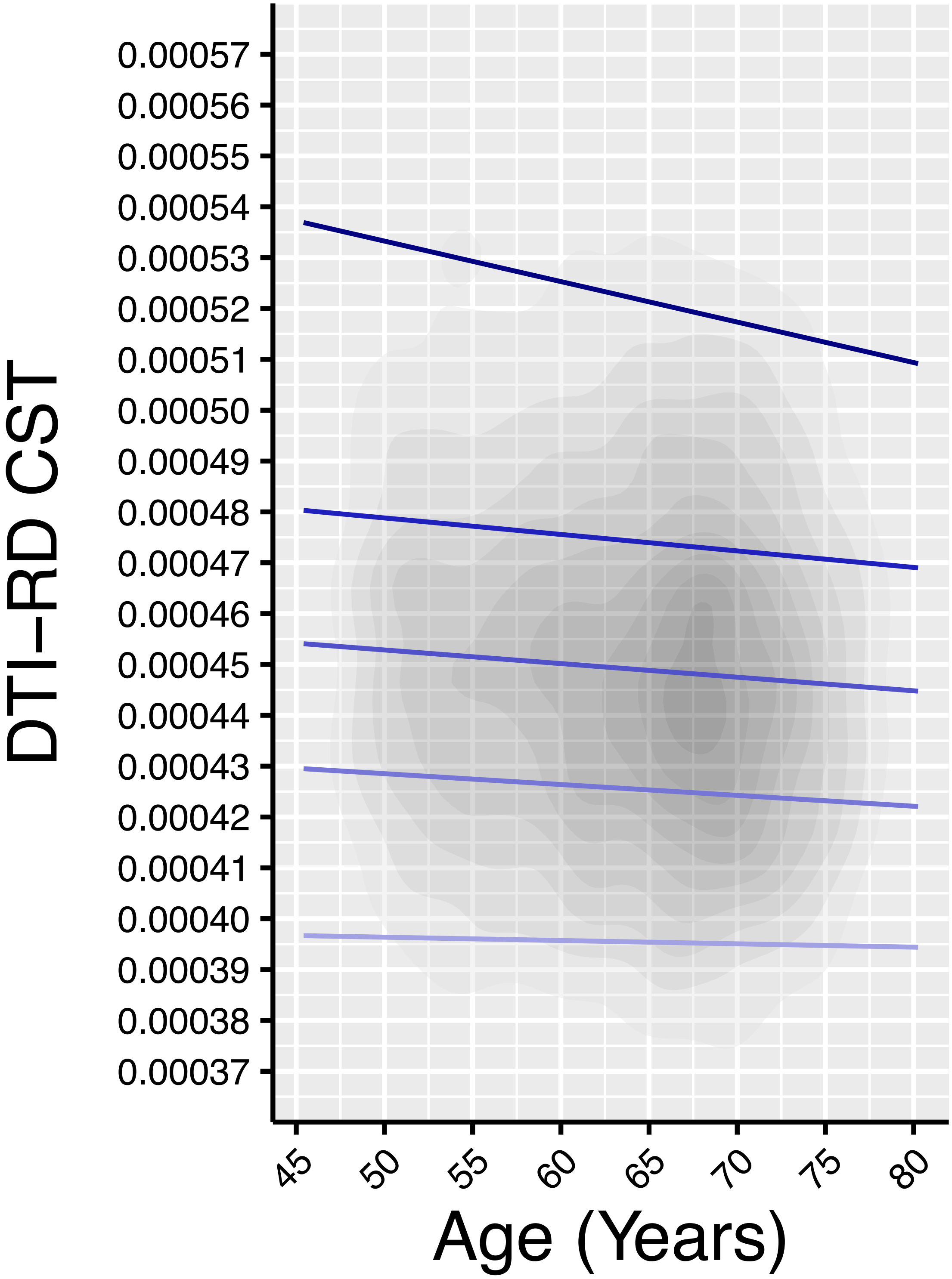

Figure S140. Full size normative centile reference curves calculated for the corticospinal tract for DTI-RD in males. Solid colored lines, ordered from lightest to darkest, indicate the following centiles: 5th, 25th, 50th, 75th, 95th. Gray overlay reflects kernel density (darker=greater degree of data point overlap). CST = corticospinal tract.

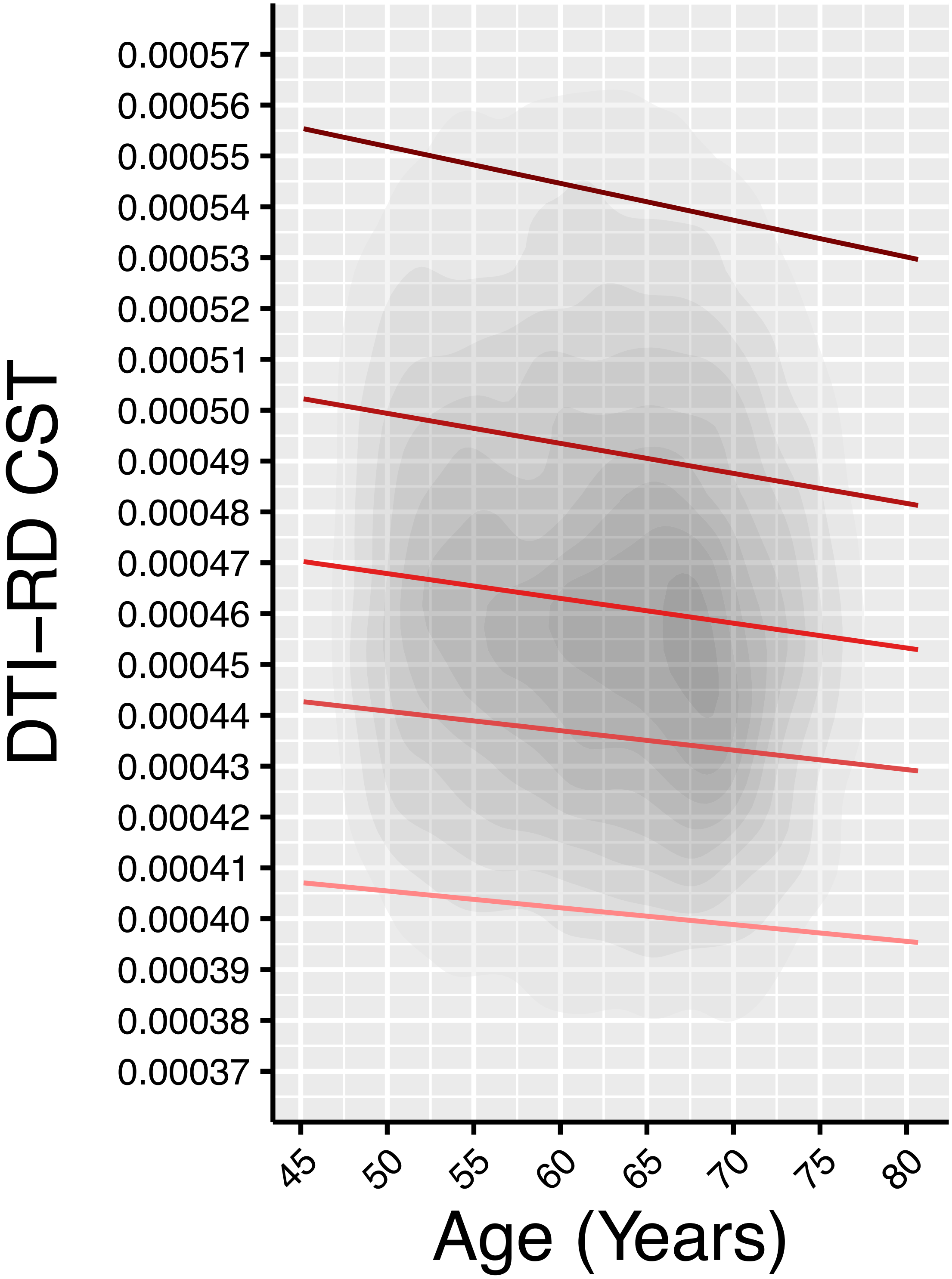

Figure S141. Full size normative centile reference curves calculated for the corticospinal tract for DTI-RD in females. Solid colored lines, ordered from lightest to darkest, indicate the following centiles: 5th, 25th, 50th, 75th, 95th. Gray overlay reflects kernel density (darker=greater degree of data point overlap). CST = corticospinal tract.

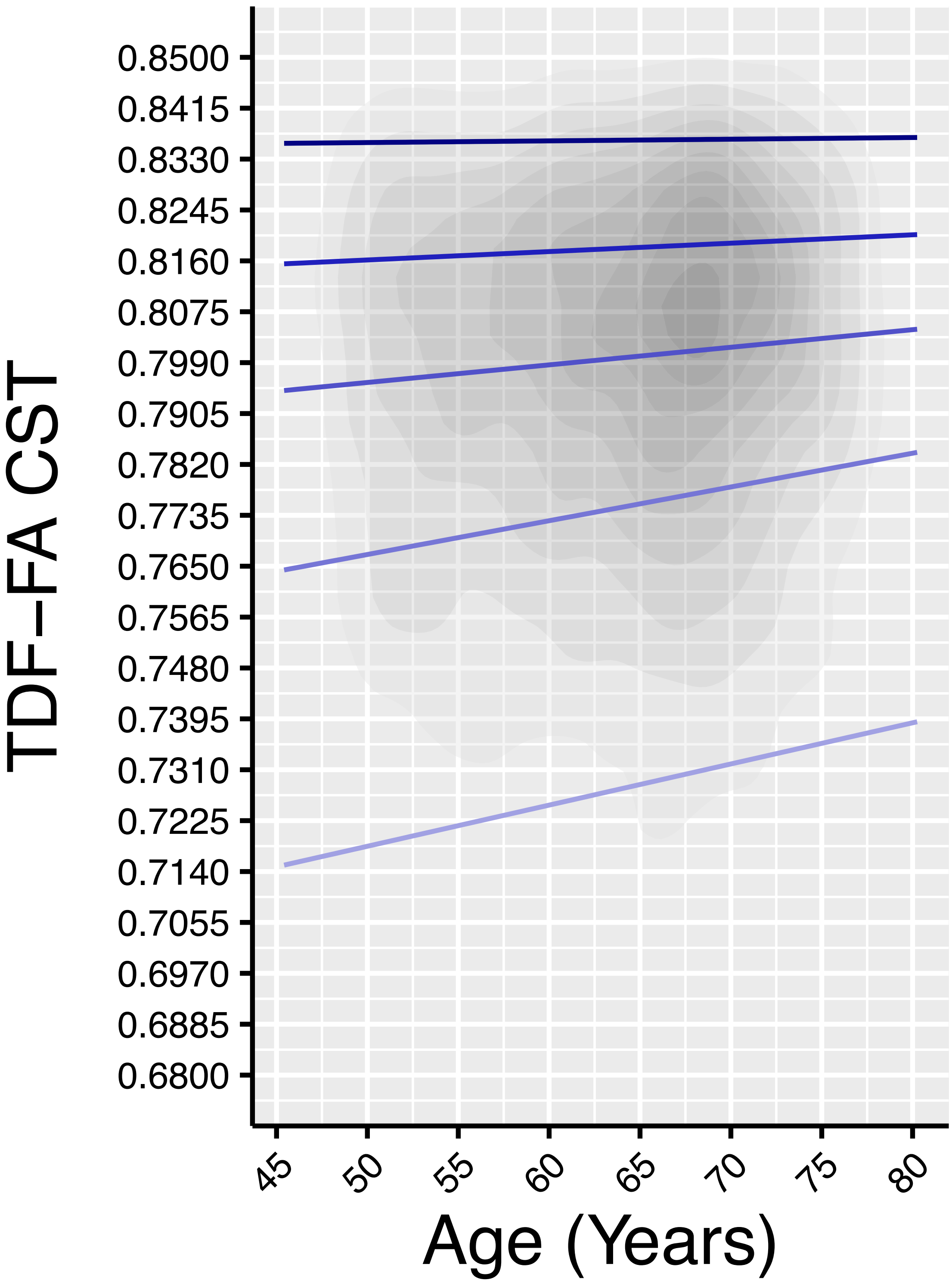

Figure S142. Full size normative centile reference curves calculated for the corticospinal tract for TDF-FA in males. Solid colored lines, ordered from lightest to darkest, indicate the following centiles: 5th, 25th, 50th, 75th, 95th. Gray overlay reflects kernel density (darker=greater degree of data point overlap). CST = corticospinal tract.

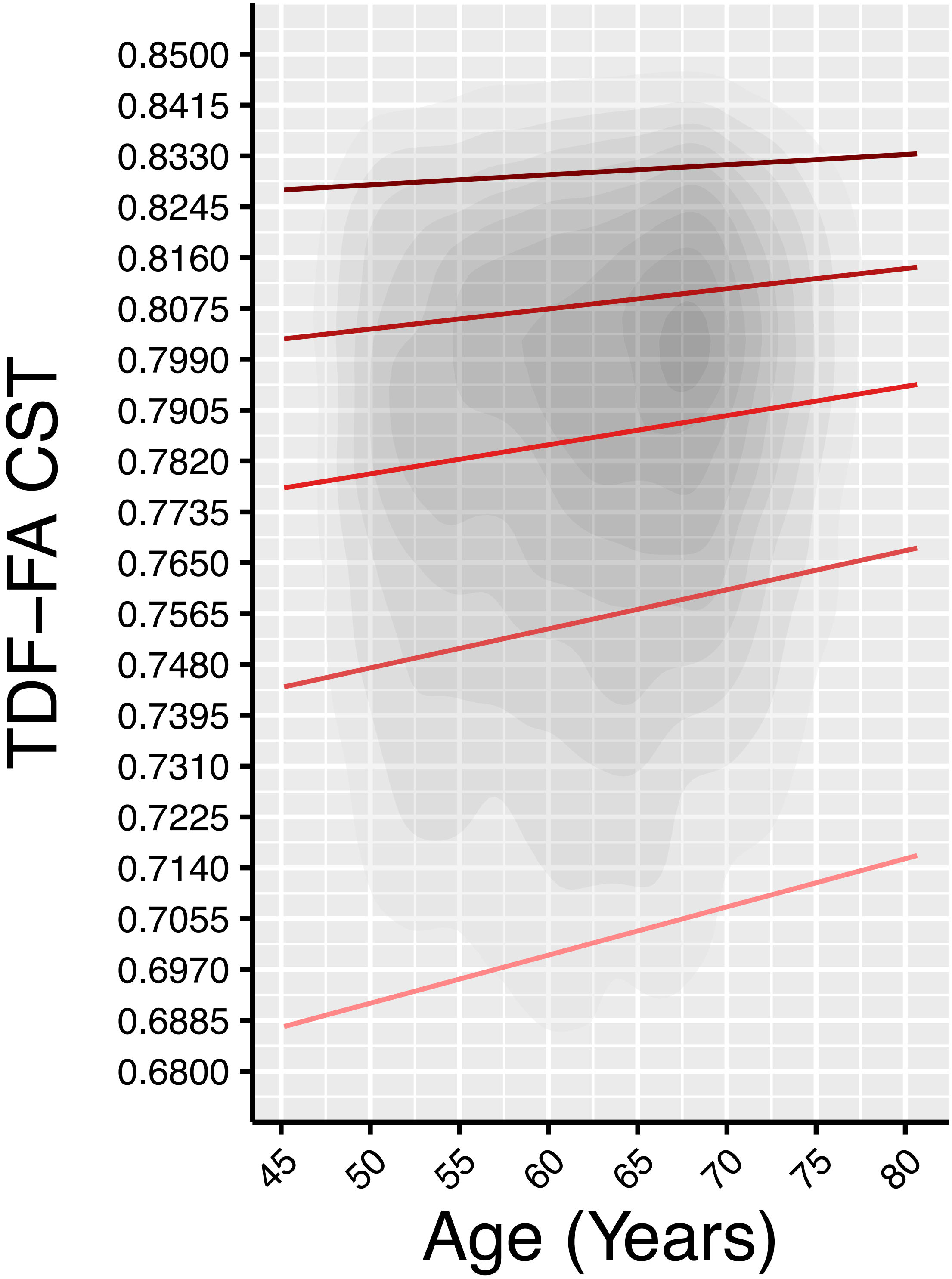

Figure S143. Full size normative centile reference curves calculated for the corticospinal tract for TDF-FA in females. Solid colored lines, ordered from lightest to darkest, indicate the following centiles: 5th, 25th, 50th, 75th, 95th. Gray overlay reflects kernel density (darker=greater degree of data point overlap). CST = corticospinal tract.

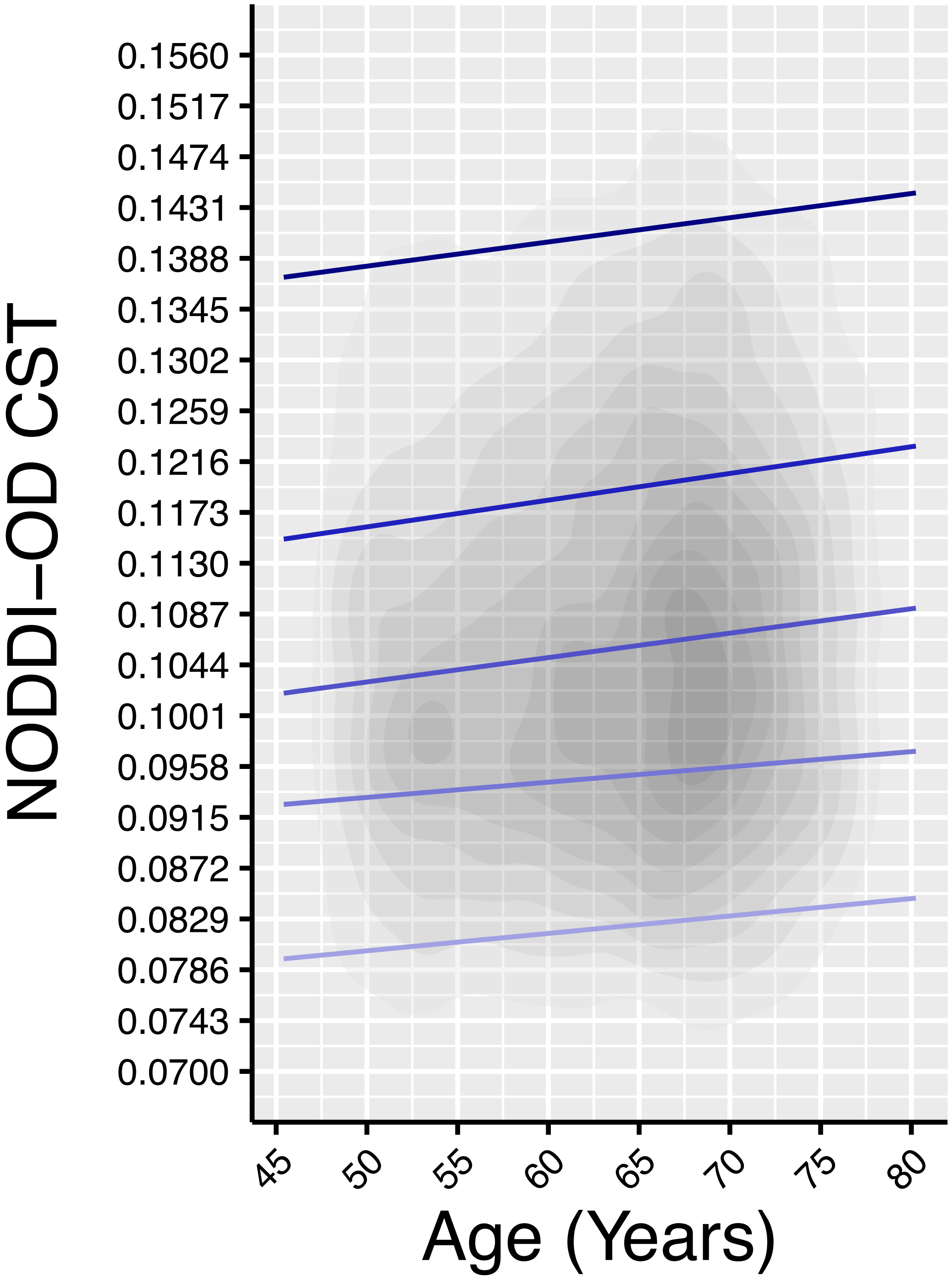

Figure S144. Full size normative centile reference curves calculated for the corticospinal tract for NODDI-OD in males. Solid colored lines, ordered from lightest to darkest, indicate the following centiles: 5th, 25th, 50th, 75th, 95th. Gray overlay reflects kernel density (darker=greater degree of data point overlap). CST = corticospinal tract.

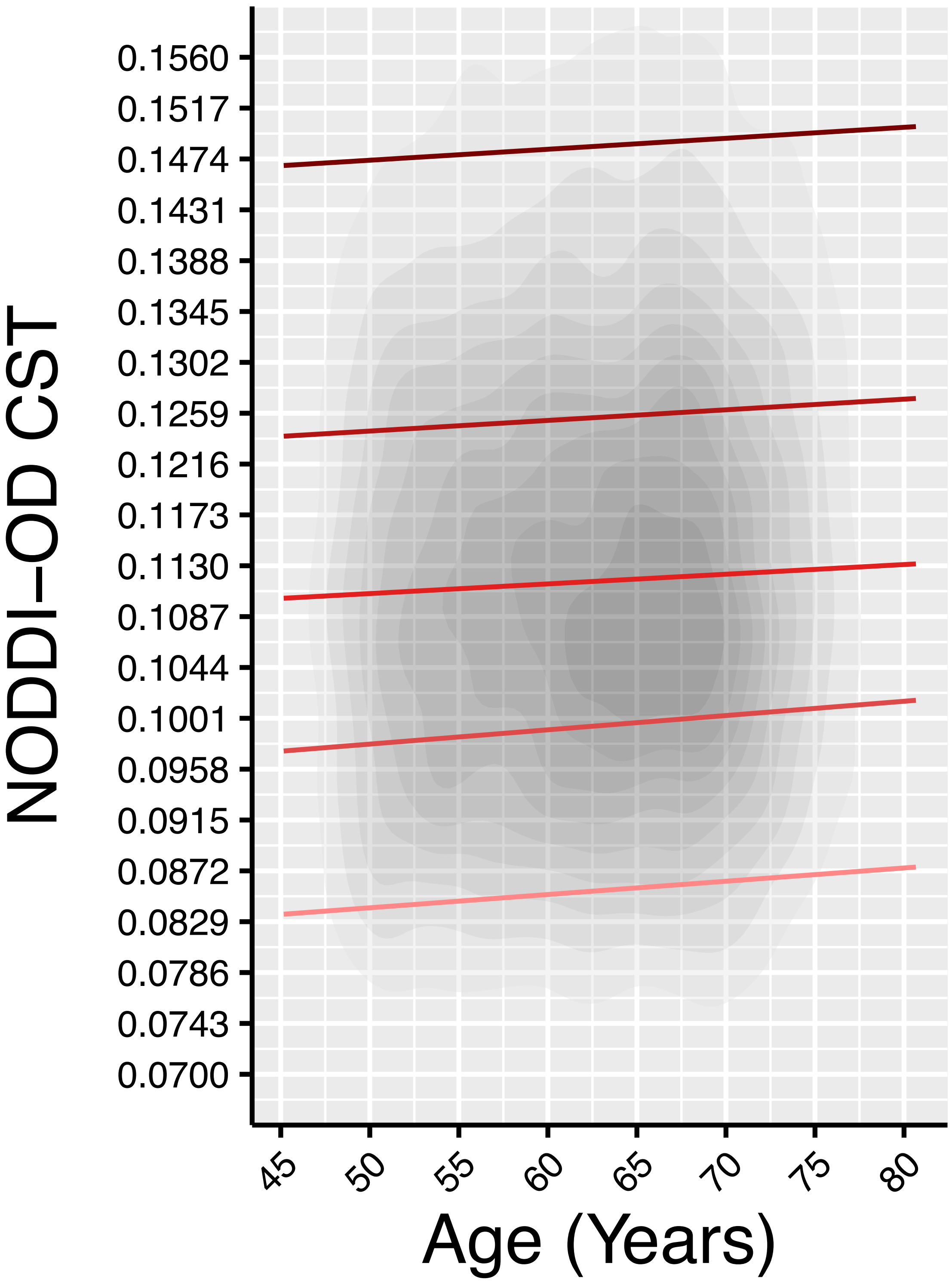

Figure S145. Full size normative centile reference curves calculated for the corticospinal tract for NODDI-OD in females. Solid colored lines, ordered from lightest to darkest, indicate the following centiles: 5th, 25th, 50th, 75th, 95th. Gray overlay reflects kernel density (darker=greater degree of data point overlap). CST = corticospinal tract.

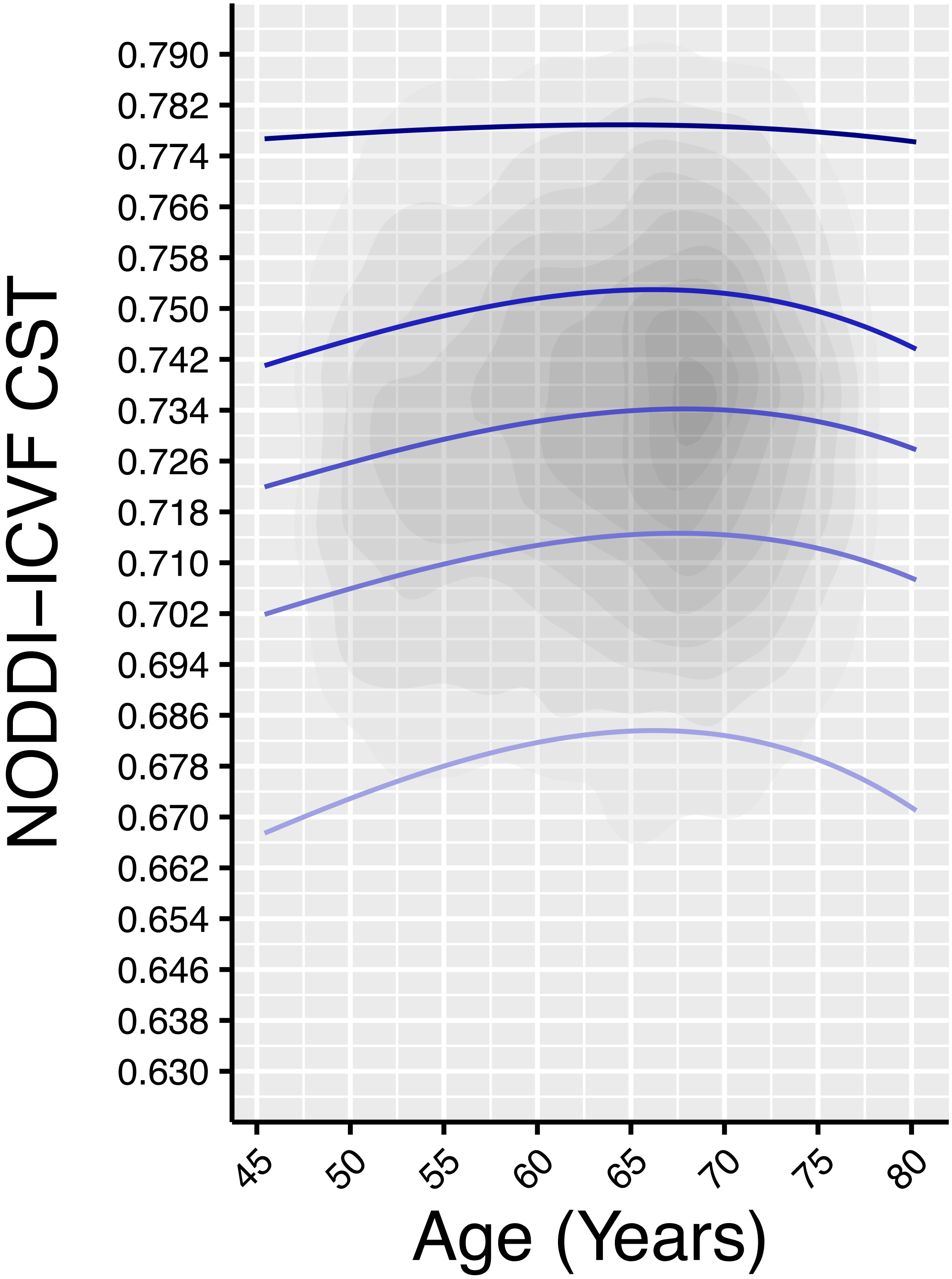

Figure S146. Full size normative centile reference curves calculated for the corticospinal tract for NODDI-ICVF in males. Solid colored lines, ordered from lightest to darkest, indicate the following centiles: 5th, 25th, 50th, 75th, 95th. Gray overlay reflects kernel density (darker=greater degree of data point overlap). CST = corticospinal tract.

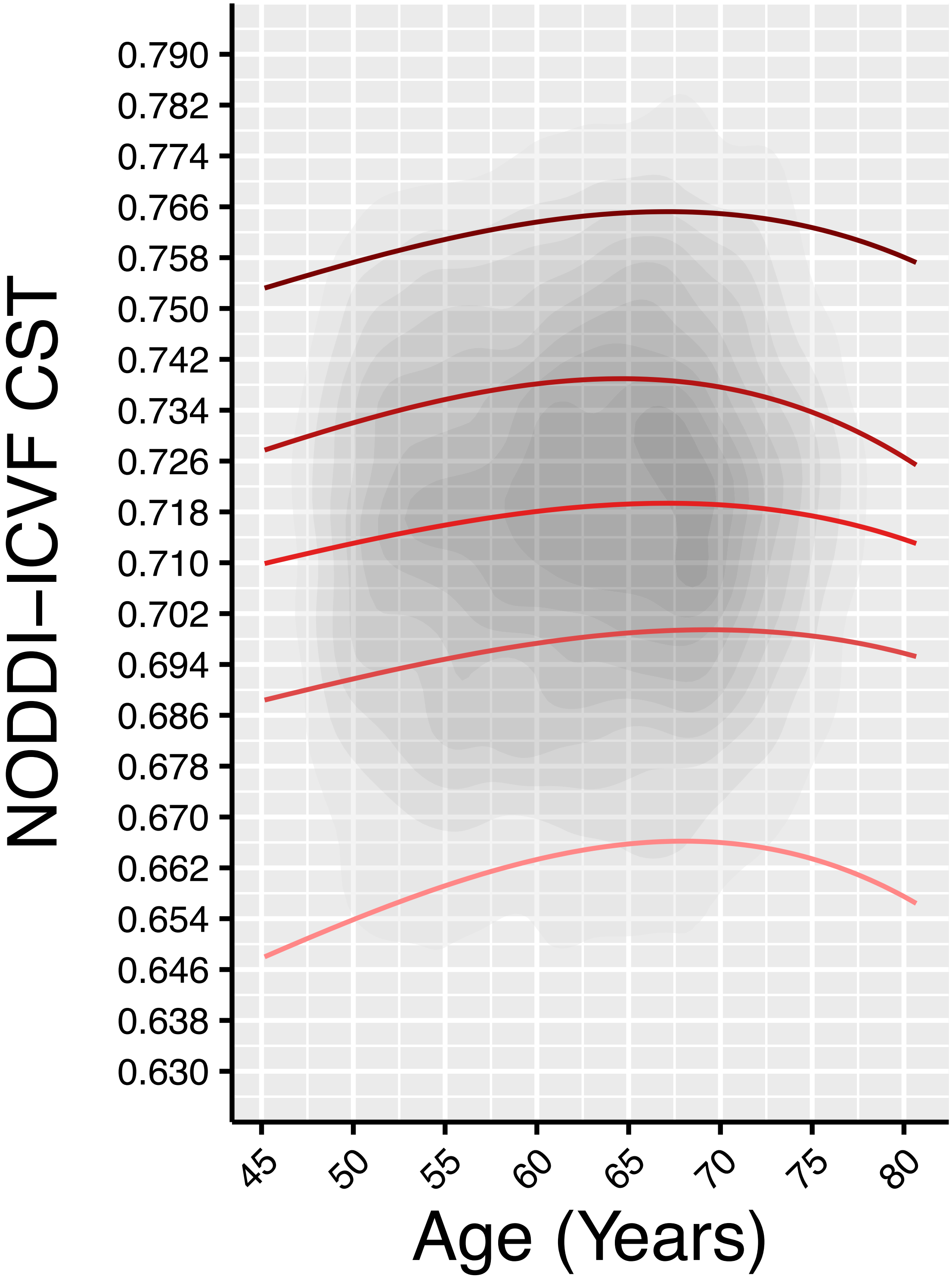

Figure S147. Full size normative centile reference curves calculated for the corticospinal tract for NODDI-ICVF in females. Solid colored lines, ordered from lightest to darkest, indicate the following centiles: 5th, 25th, 50th, 75th, 95th. Gray overlay reflects kernel density (darker=greater degree of data point overlap). CST = corticospinal tract.

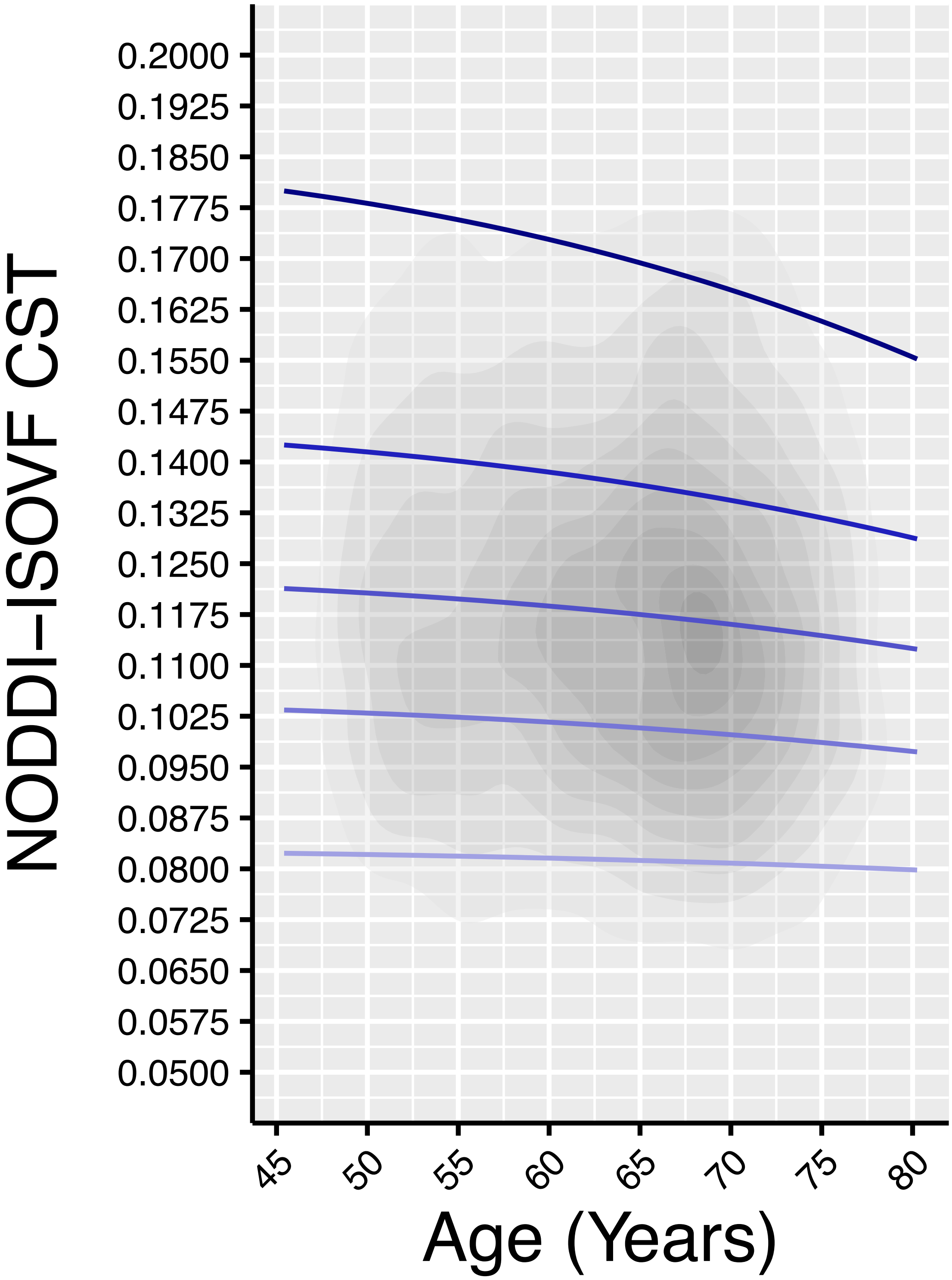

Figure S148. Full size normative centile reference curves calculated for the corticospinal tract for NODDI-ISOVF in males. Solid colored lines, ordered from lightest to darkest, indicate the following centiles: 5th, 25th, 50th, 75th, 95th. Gray overlay reflects kernel density (darker=greater degree of data point overlap). CST = corticospinal tract.

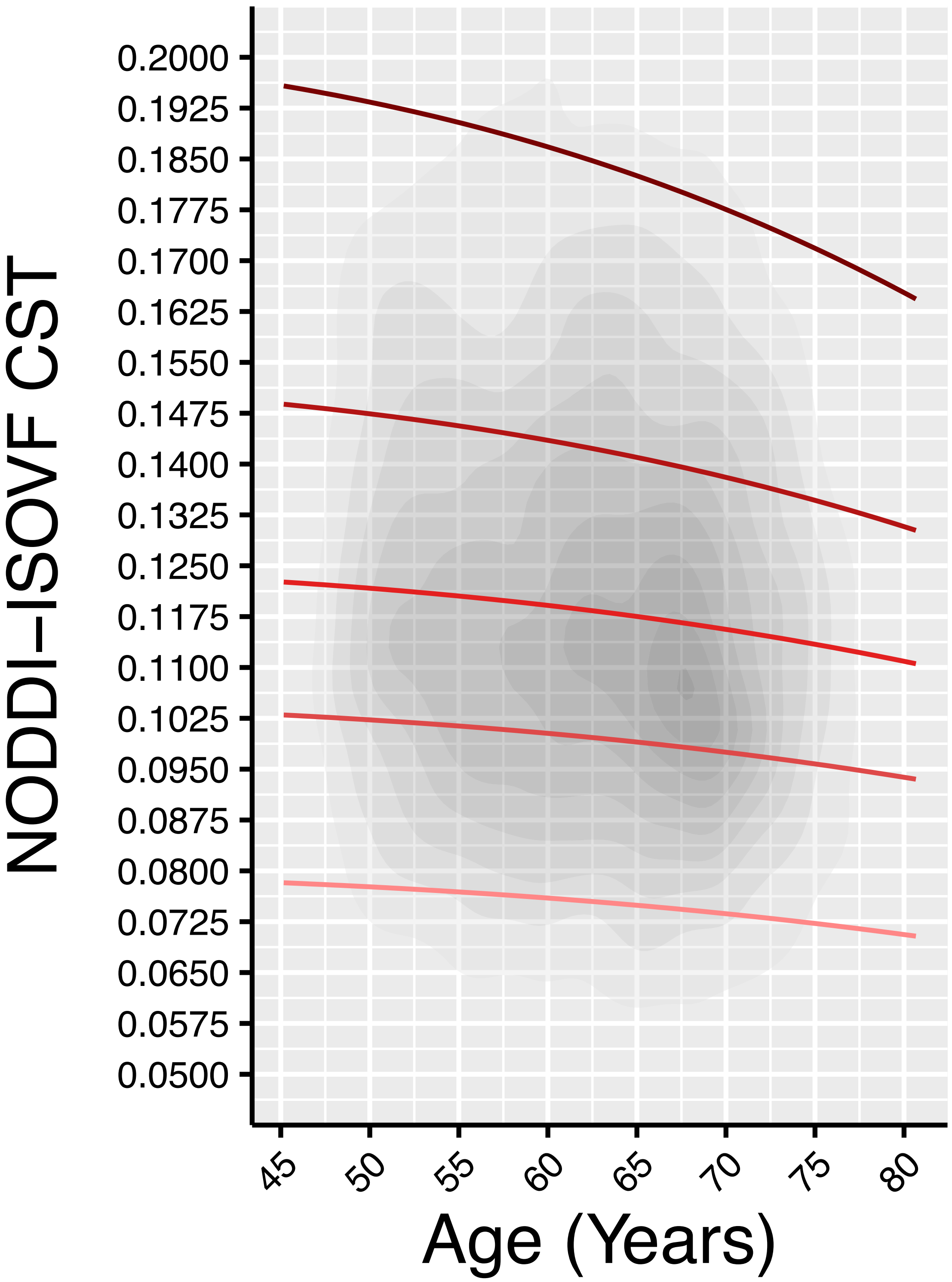

Figure S149. Full size normative centile reference curves calculated for the corticospinal tract for NODDI-ISOVF in females. Solid colored lines, ordered from lightest to darkest, indicate the following centiles: 5th, 25th, 50th, 75th, 95th. Gray overlay reflects kernel density (darker=greater degree of data point overlap). CST = corticospinal tract.

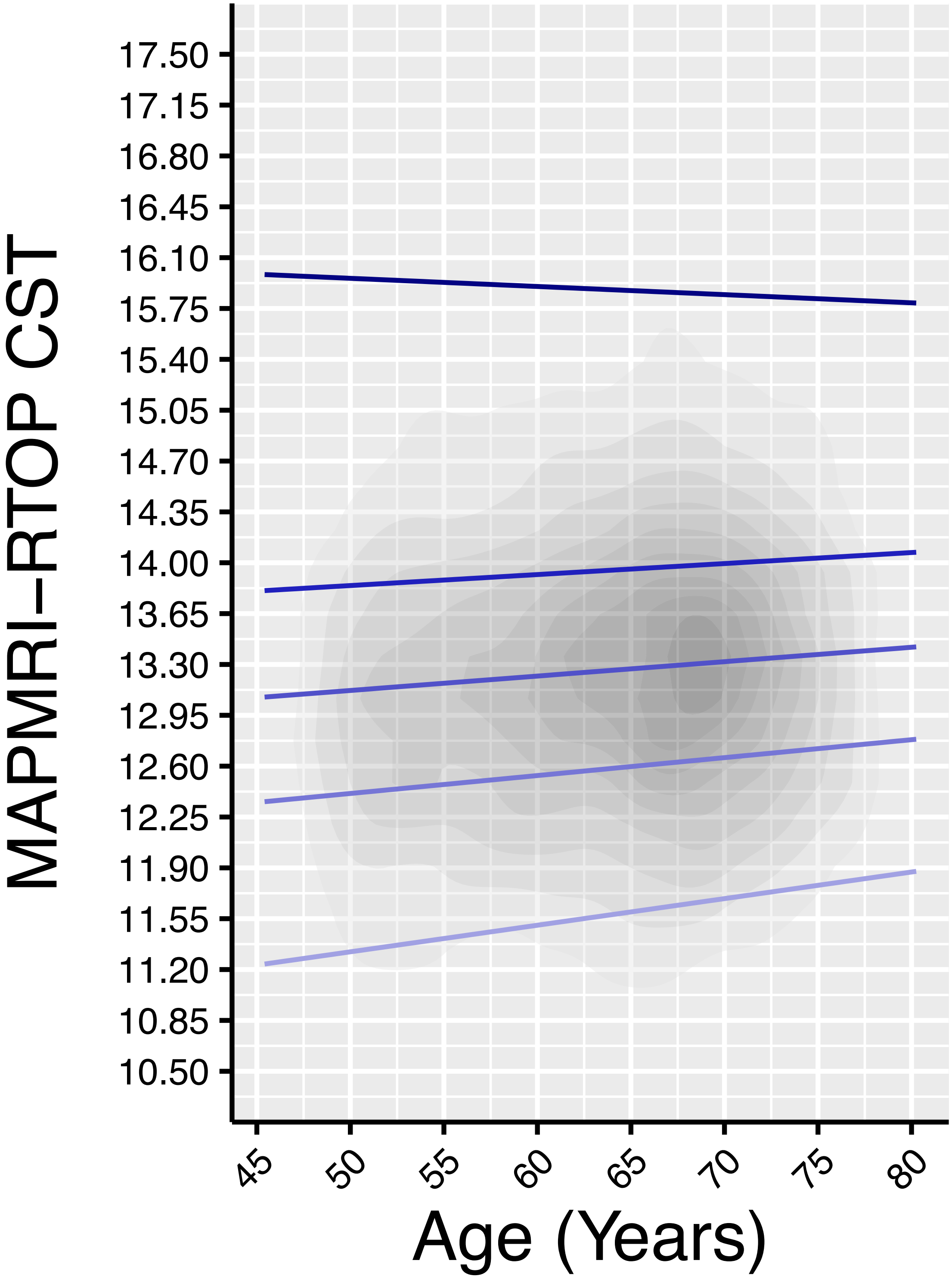

Figure S150. Full size normative centile reference curves calculated for the corticospinal tract for MAPMRI-RTOP in males. Solid colored lines, ordered from lightest to darkest, indicate the following centiles: 5th, 25th, 50th, 75th, 95th. Gray overlay reflects kernel density (darker=greater degree of data point overlap). CST = corticospinal tract.

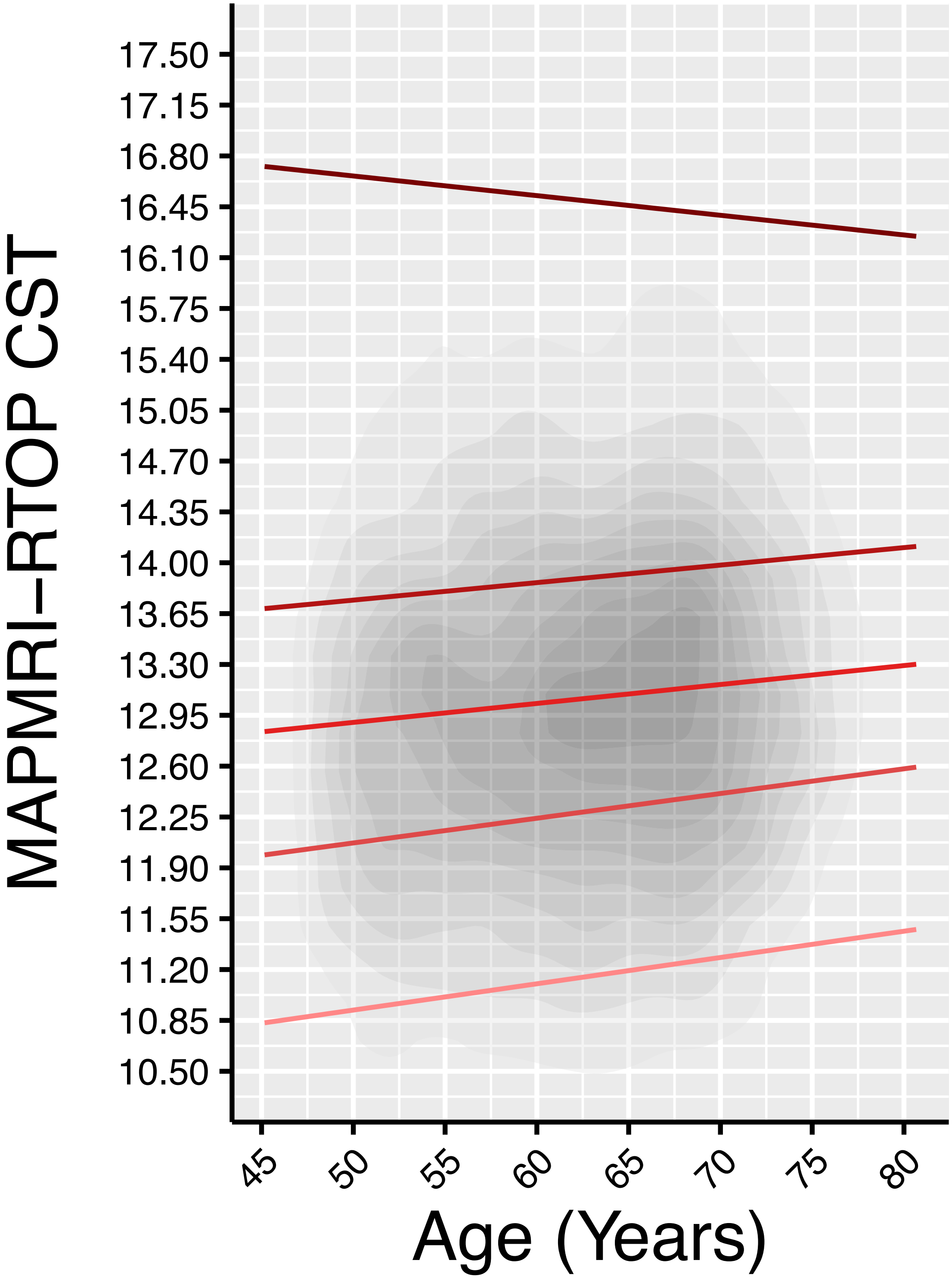

Figure S151. Full size normative centile reference curves calculated for the corticospinal tract for MAPMRI-RTOP in females. Solid colored lines, ordered from lightest to darkest, indicate the following centiles: 5th, 25th, 50th, 75th, 95th. Gray overlay reflects kernel density (darker=greater degree of data point overlap). CST = corticospinal tract.

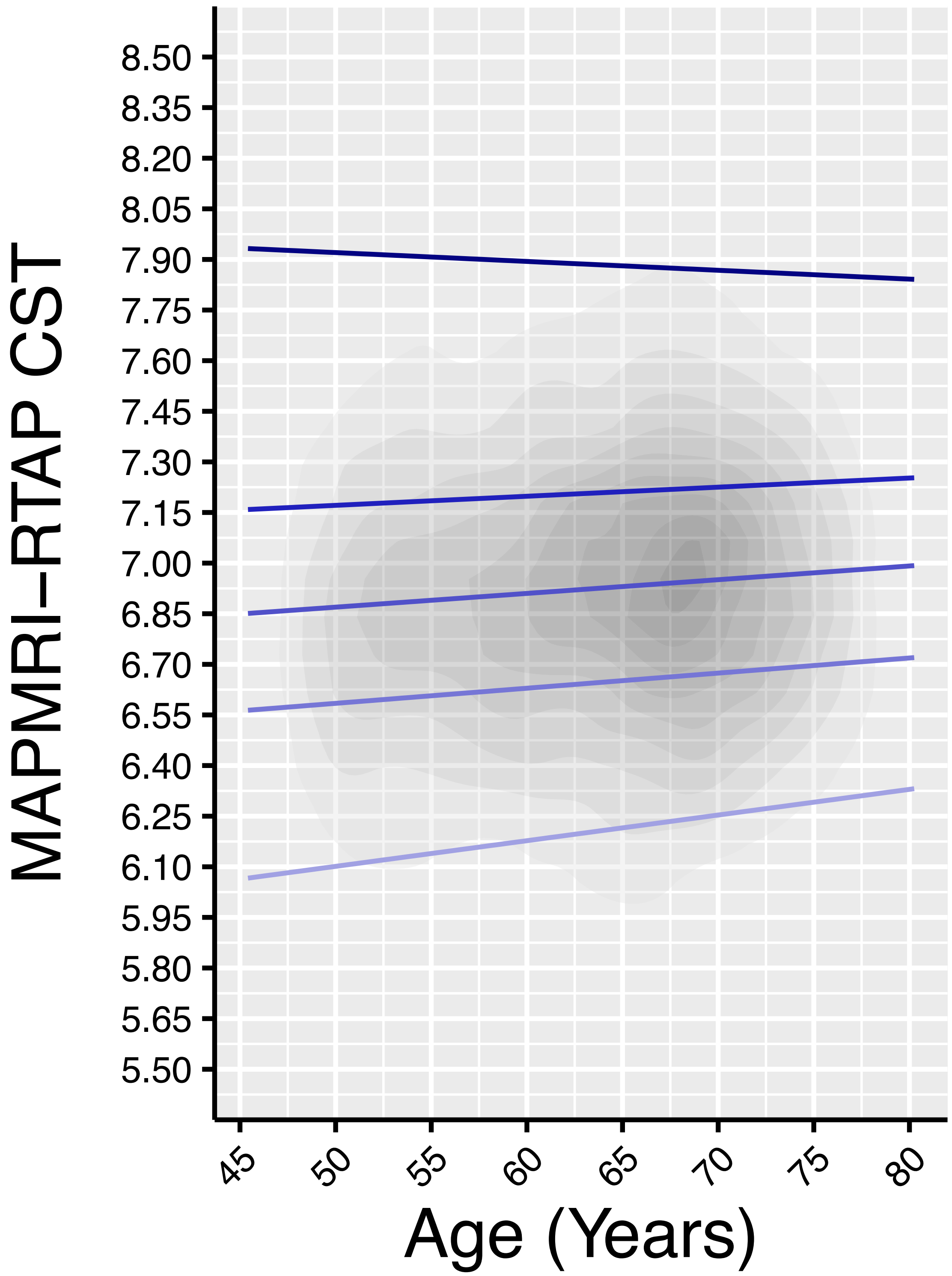

Figure S152. Full size normative centile reference curves calculated for the corticospinal tract for MAPMRI-RTAP in males. Solid colored lines, ordered from lightest to darkest, indicate the following centiles: 5th, 25th, 50th, 75th, 95th. Gray overlay reflects kernel density (darker=greater degree of data point overlap). CST = corticospinal tract.

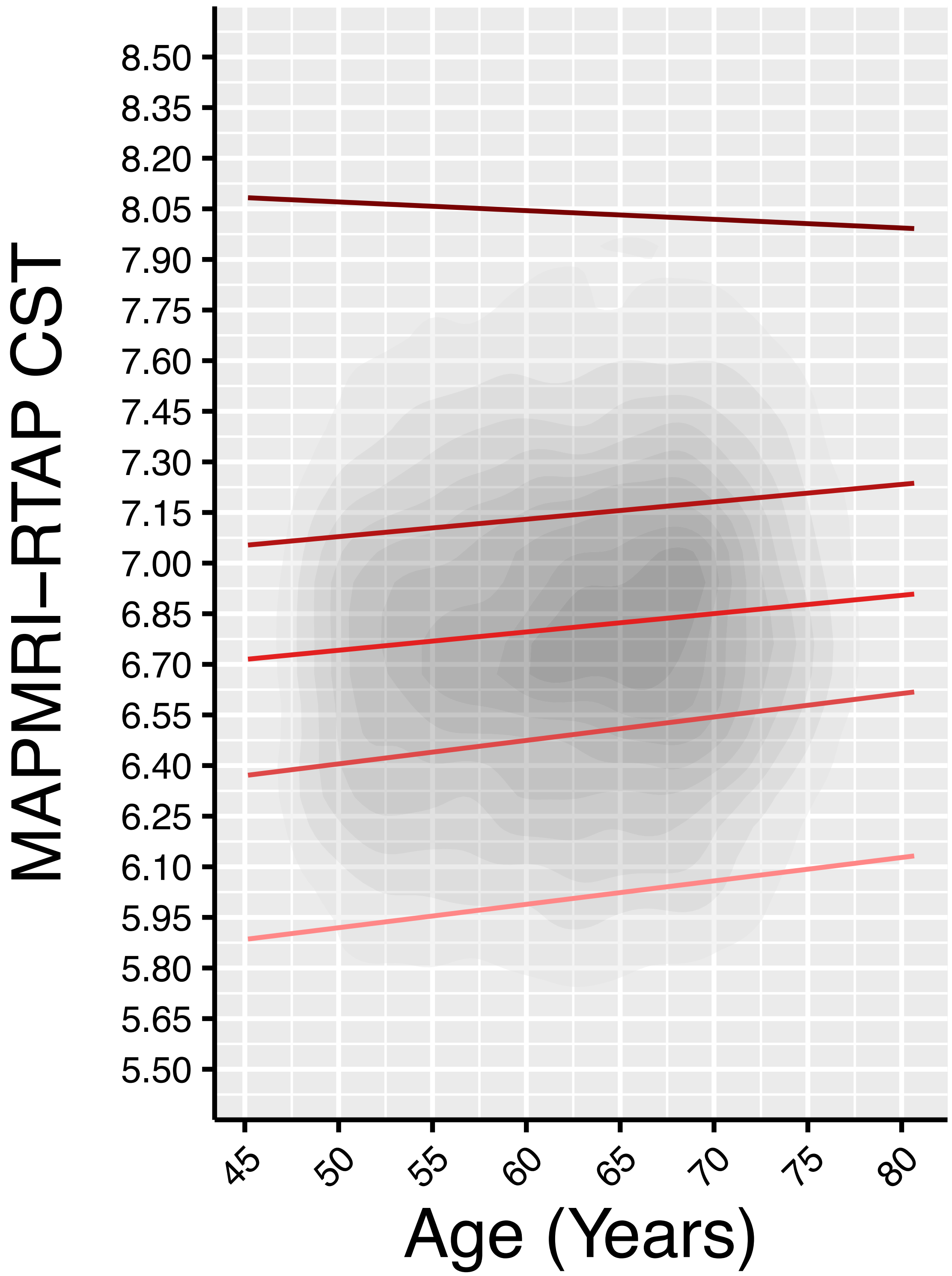

Figure S153. Full size normative centile reference curves calculated for the corticospinal tract for MAPMRI-RTAP in females. Solid colored lines, ordered from lightest to darkest, indicate the following centiles: 5th, 25th, 50th, 75th, 95th. Gray overlay reflects kernel density (darker=greater degree of data point overlap). CST = corticospinal tract.

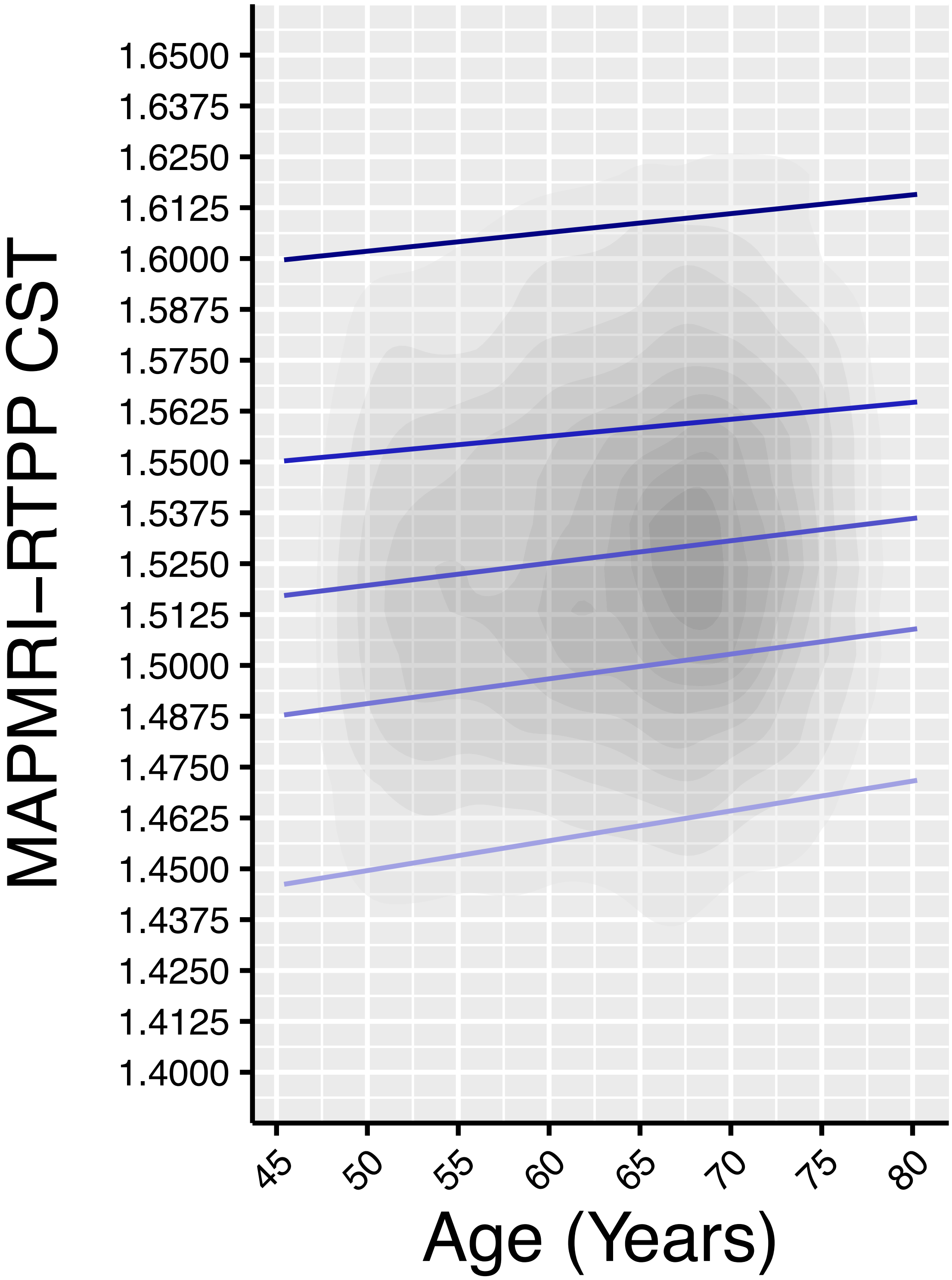

Figure S154. Full size normative centile reference curves calculated for the corticospinal tract for MAPMRI-RTPP in males. Solid colored lines, ordered from lightest to darkest, indicate the following centiles: 5th, 25th, 50th, 75th, 95th. Gray overlay reflects kernel density (darker=greater degree of data point overlap). CST = corticospinal tract.

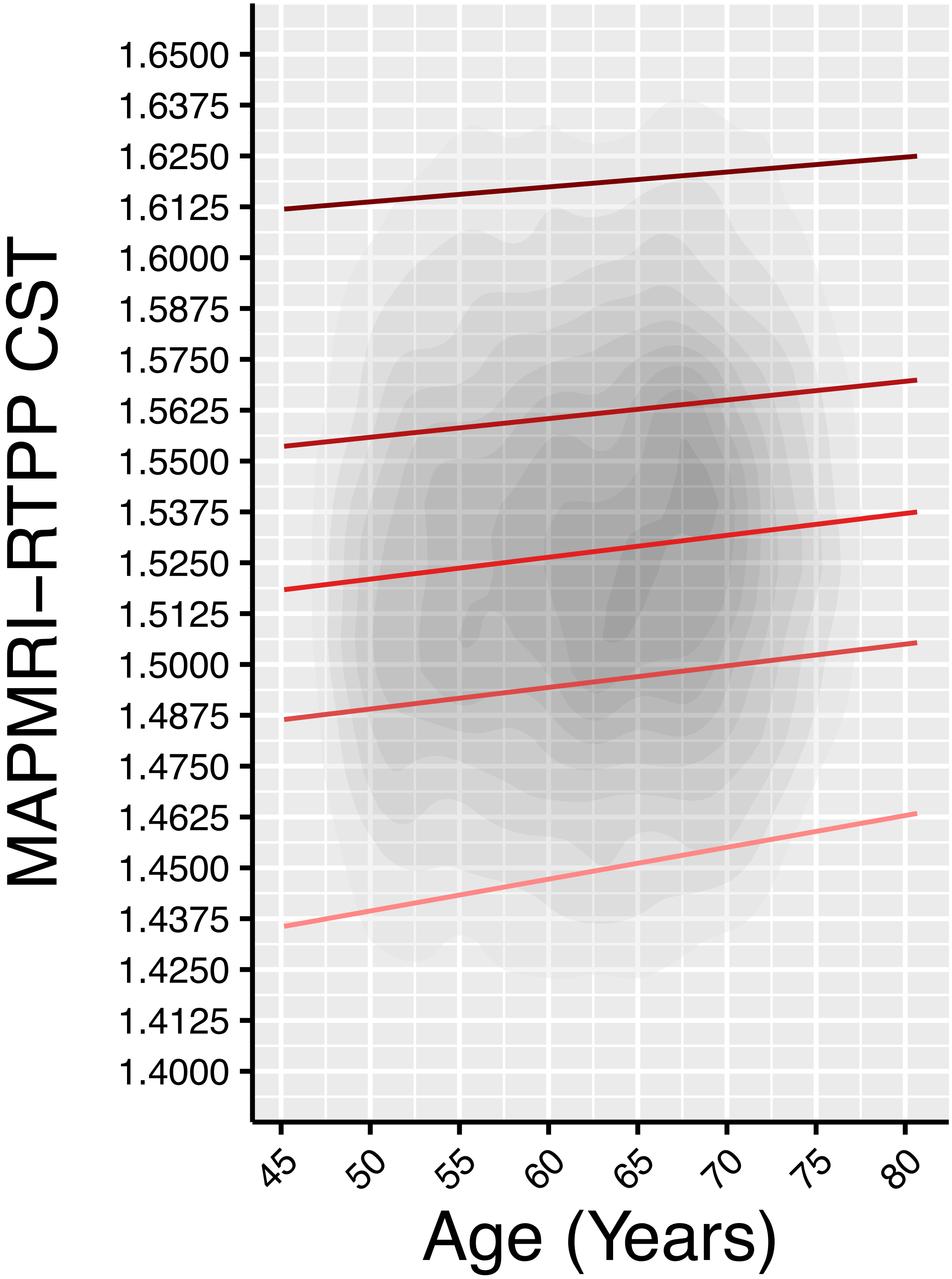

Figure S155. Full size normative centile reference curves calculated for the corticospinal tract for MAPMRI-RTPP in females. Solid colored lines, ordered from lightest to darkest, indicate the following centiles: 5th, 25th, 50th, 75th, 95th. Gray overlay reflects kernel density (darker=greater degree of data point overlap). CST = corticospinal tract.

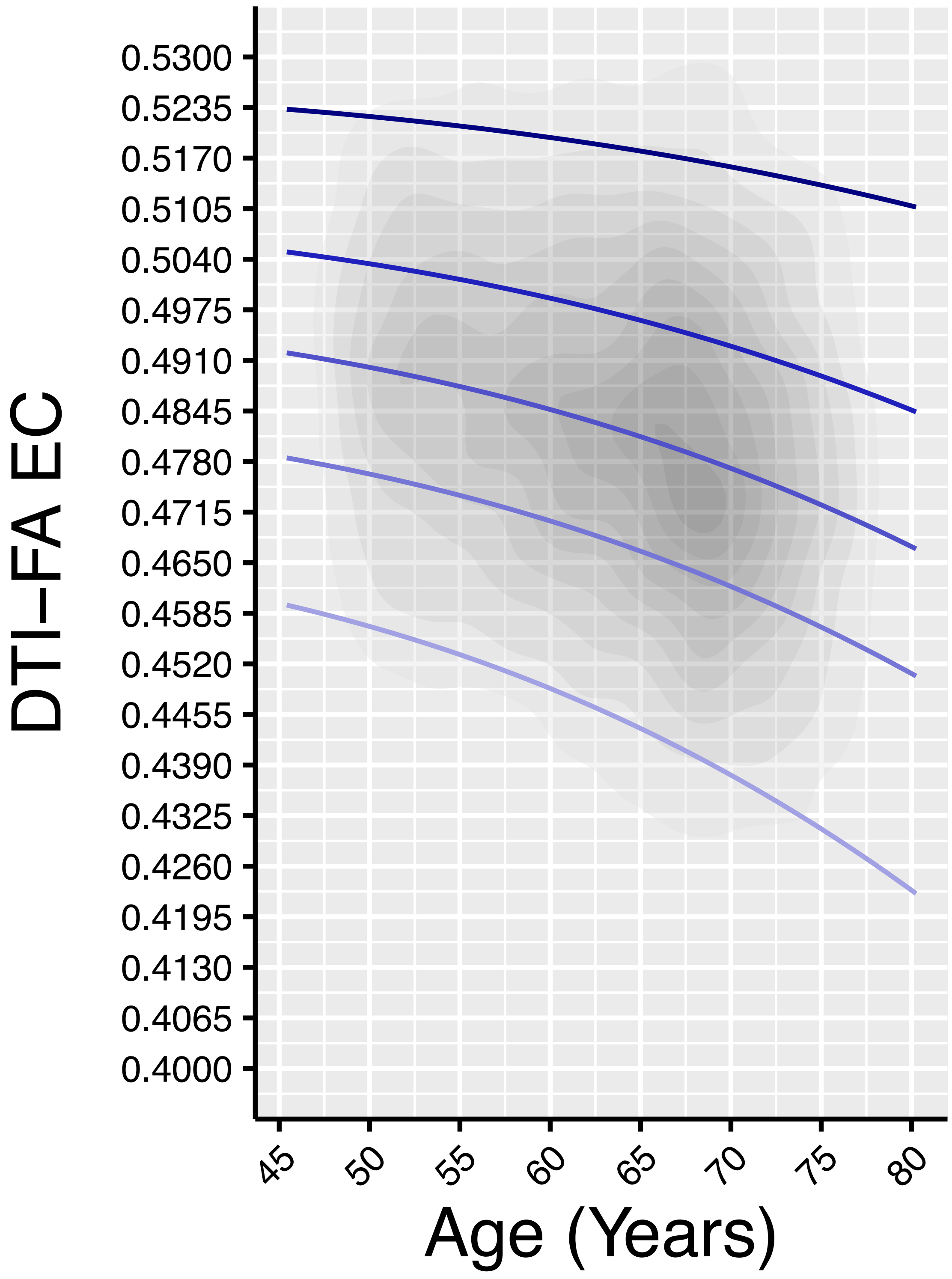

Figure S156. Full size normative centile reference curves calculated for the external capsule tract for DTI-FA in males. Solid colored lines, ordered from lightest to darkest, indicate the following centiles: 5th, 25th, 50th, 75th, 95th. Gray overlay reflects kernel density (darker=greater degree of data point overlap). EC = external capsule.

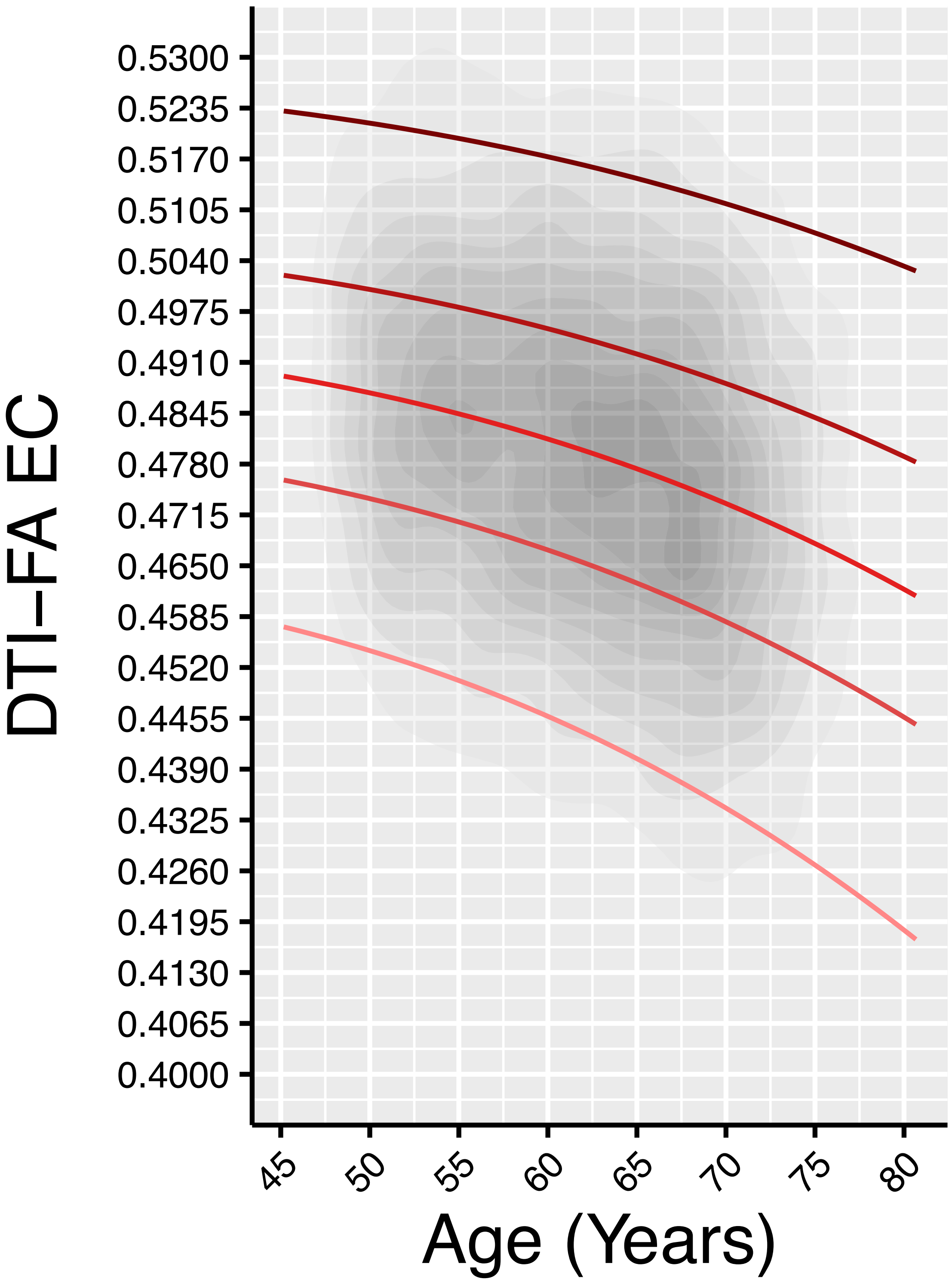

Figure S157. Full size normative centile reference curves calculated for the external capsule tract for DTI-FA in females. Solid colored lines, ordered from lightest to darkest, indicate the following centiles: 5th, 25th, 50th, 75th, 95th. Gray overlay reflects kernel density (darker=greater degree of data point overlap). EC = external capsule.

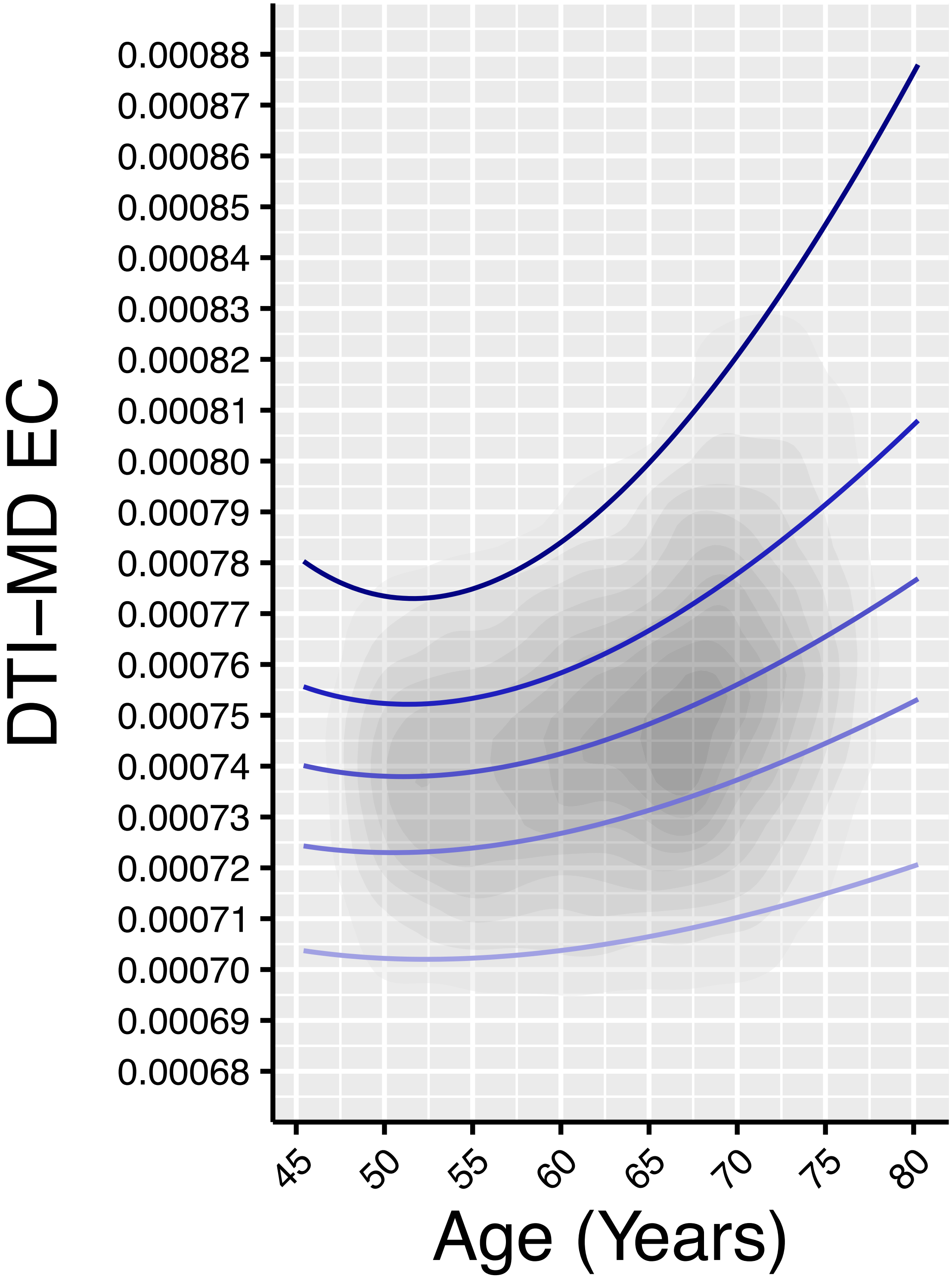

Figure S158. Full size normative centile reference curves calculated for the external capsule tract for DTI-MD in males. Solid colored lines, ordered from lightest to darkest, indicate the following centiles: 5th, 25th, 50th, 75th, 95th. Gray overlay reflects kernel density (darker=greater degree of data point overlap). EC = external capsule.

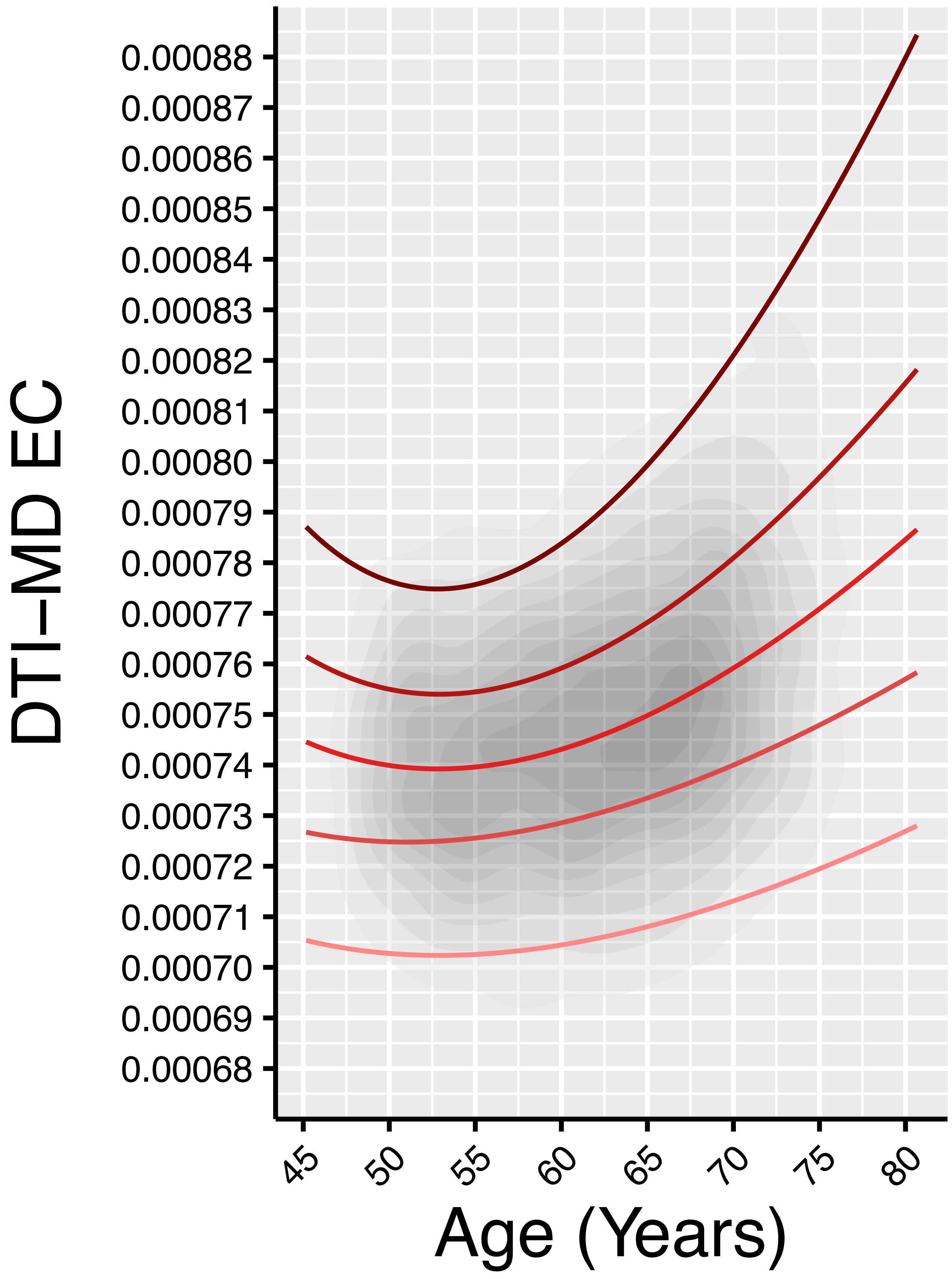

Figure S159. Full size normative centile reference curves calculated for the external capsule tract for DTI-MD in females. Solid colored lines, ordered from lightest to darkest, indicate the following centiles: 5th, 25th, 50th, 75th, 95th. Gray overlay reflects kernel density (darker=greater degree of data point overlap). EC = external capsule.

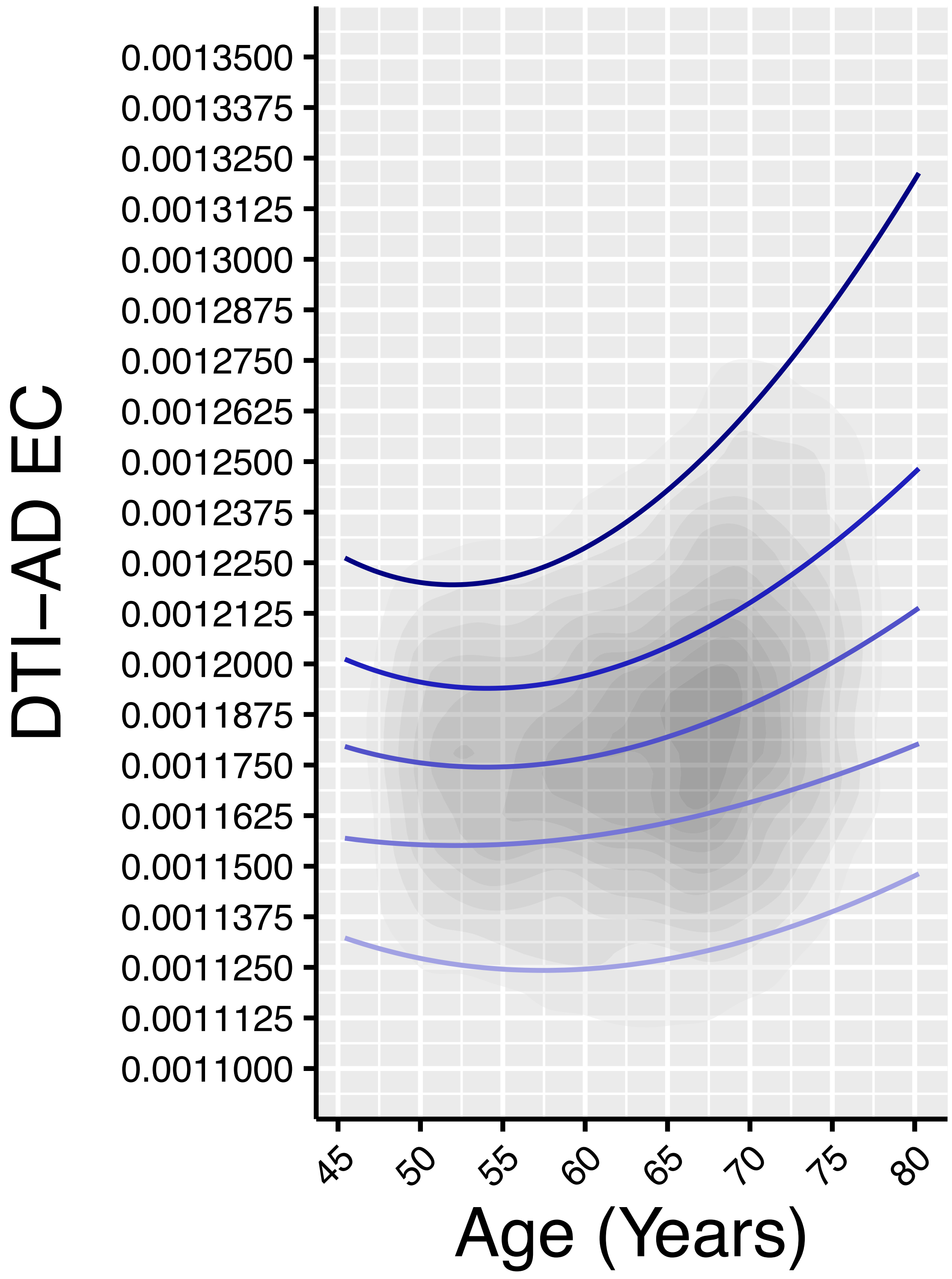

Figure S160. Full size normative centile reference curves calculated for the external capsule tract for DTI-AD in males. Solid colored lines, ordered from lightest to darkest, indicate the following centiles: 5th, 25th, 50th, 75th, 95th. Gray overlay reflects kernel density (darker=greater degree of data point overlap). EC = external capsule.

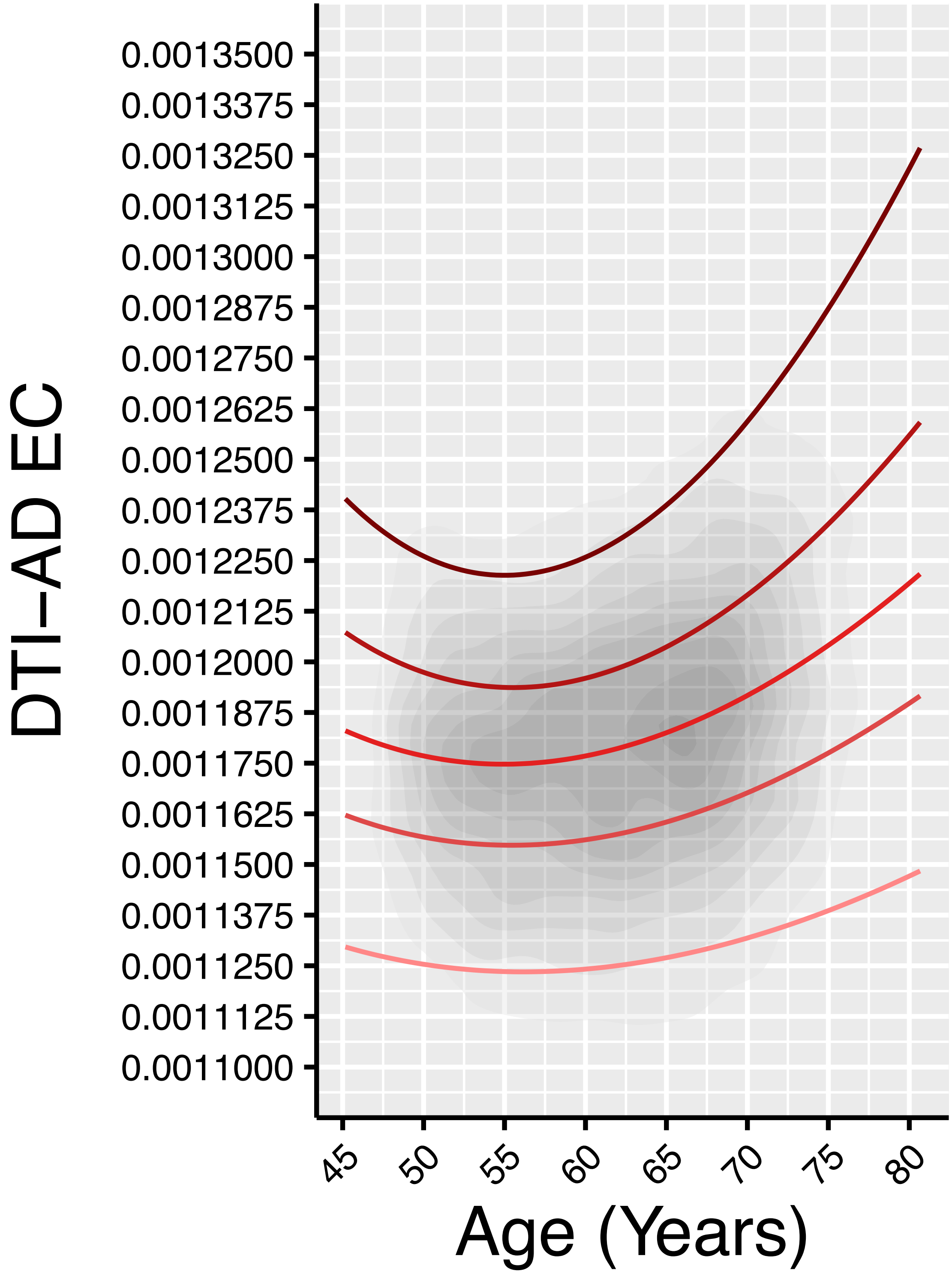

Figure S161. Full size normative centile reference curves calculated for the external capsule tract for DTI-AD in females. Solid colored lines, ordered from lightest to darkest, indicate the following centiles: 5th, 25th, 50th, 75th, 95th. Gray overlay reflects kernel density (darker=greater degree of data point overlap). EC = external capsule.

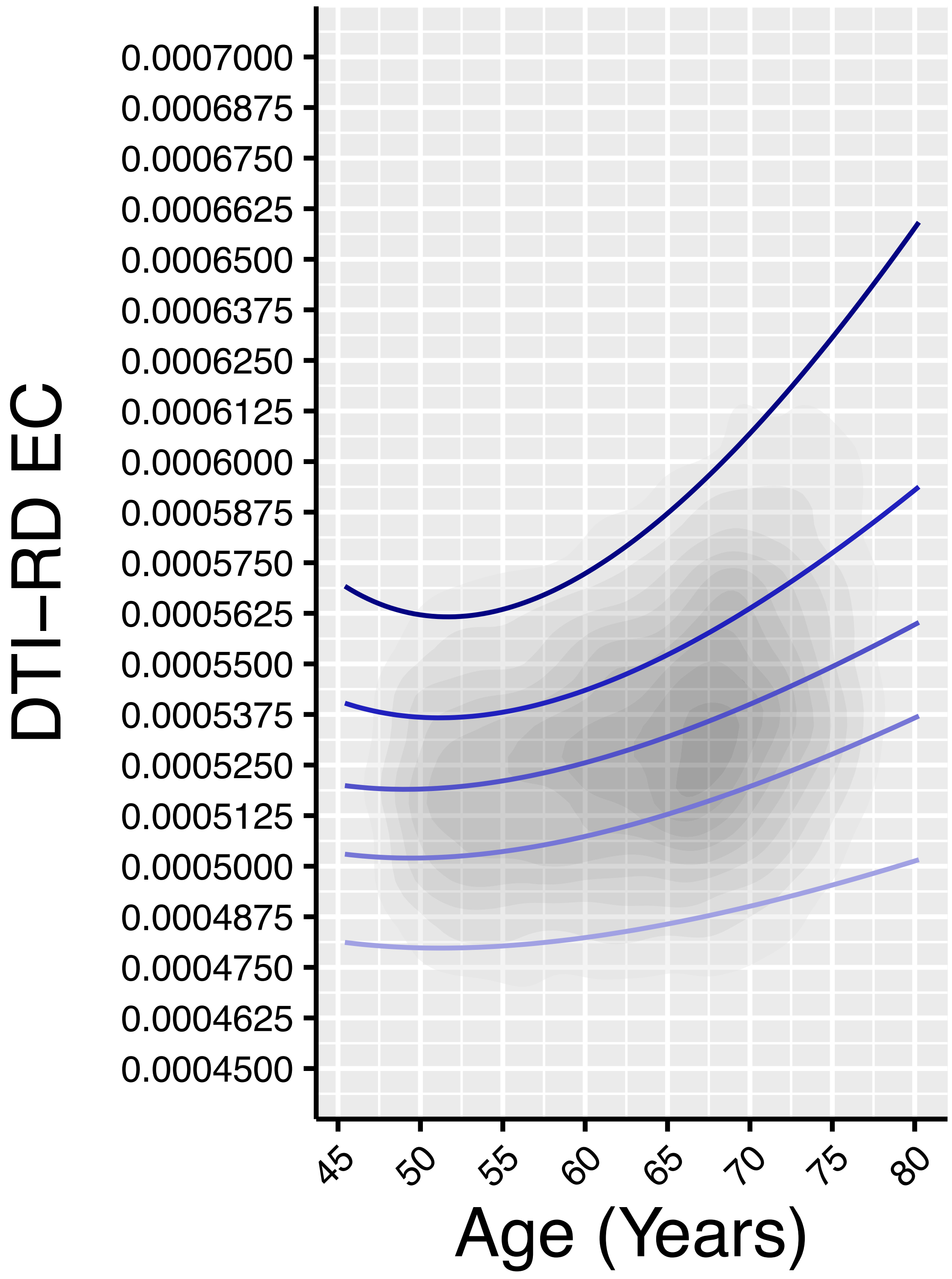

Figure S162. Full size normative centile reference curves calculated for the external capsule tract for DTI-RD in males. Solid colored lines, ordered from lightest to darkest, indicate the following centiles: 5th, 25th, 50th, 75th, 95th. Gray overlay reflects kernel density (darker=greater degree of data point overlap). EC = external capsule.

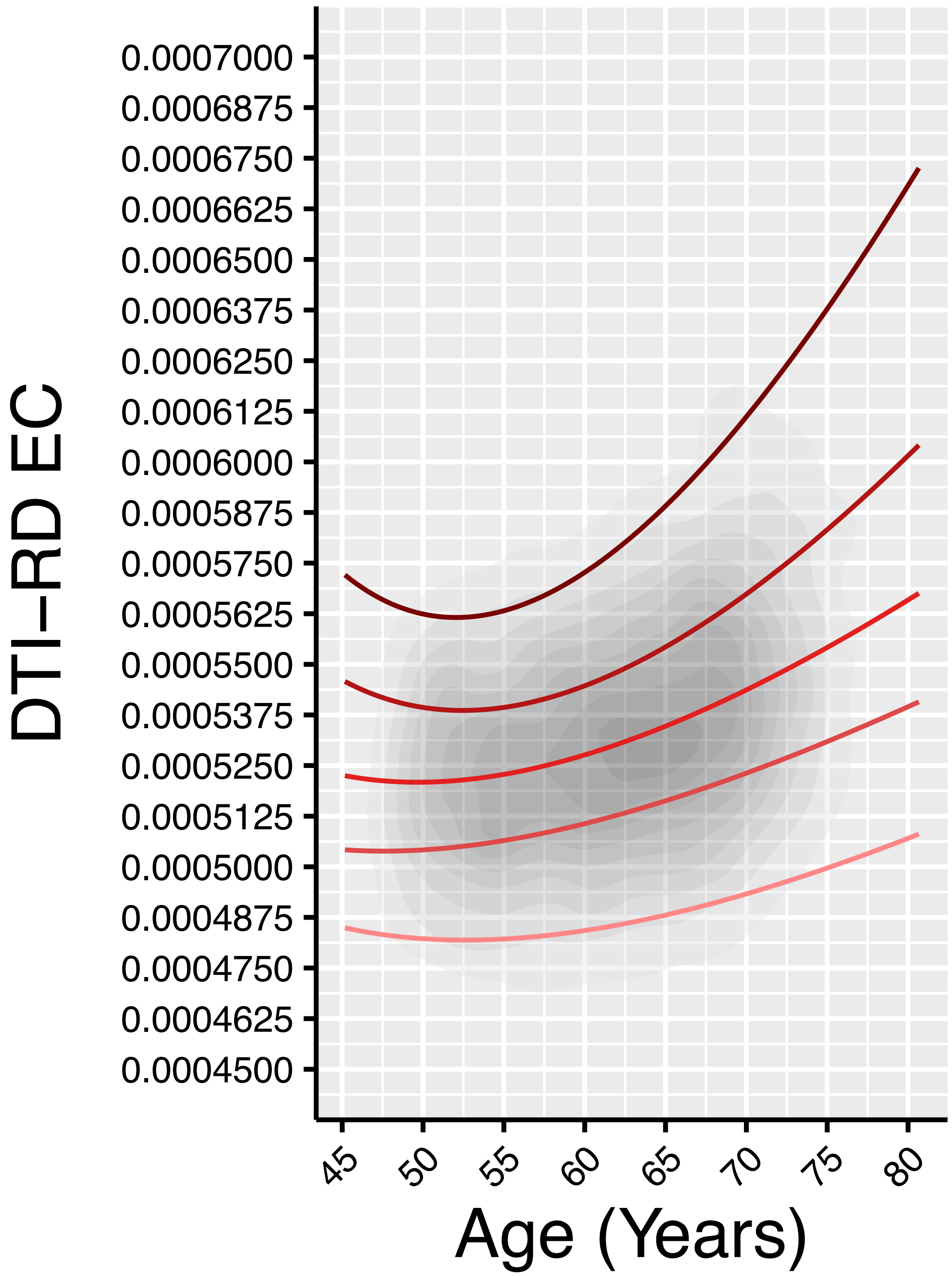

Figure S163. Full size normative centile reference curves calculated for the external capsule tract for DTI-RD in females. Solid colored lines, ordered from lightest to darkest, indicate the following centiles: 5th, 25th, 50th, 75th, 95th. Gray overlay reflects kernel density (darker=greater degree of data point overlap). EC = external capsule.

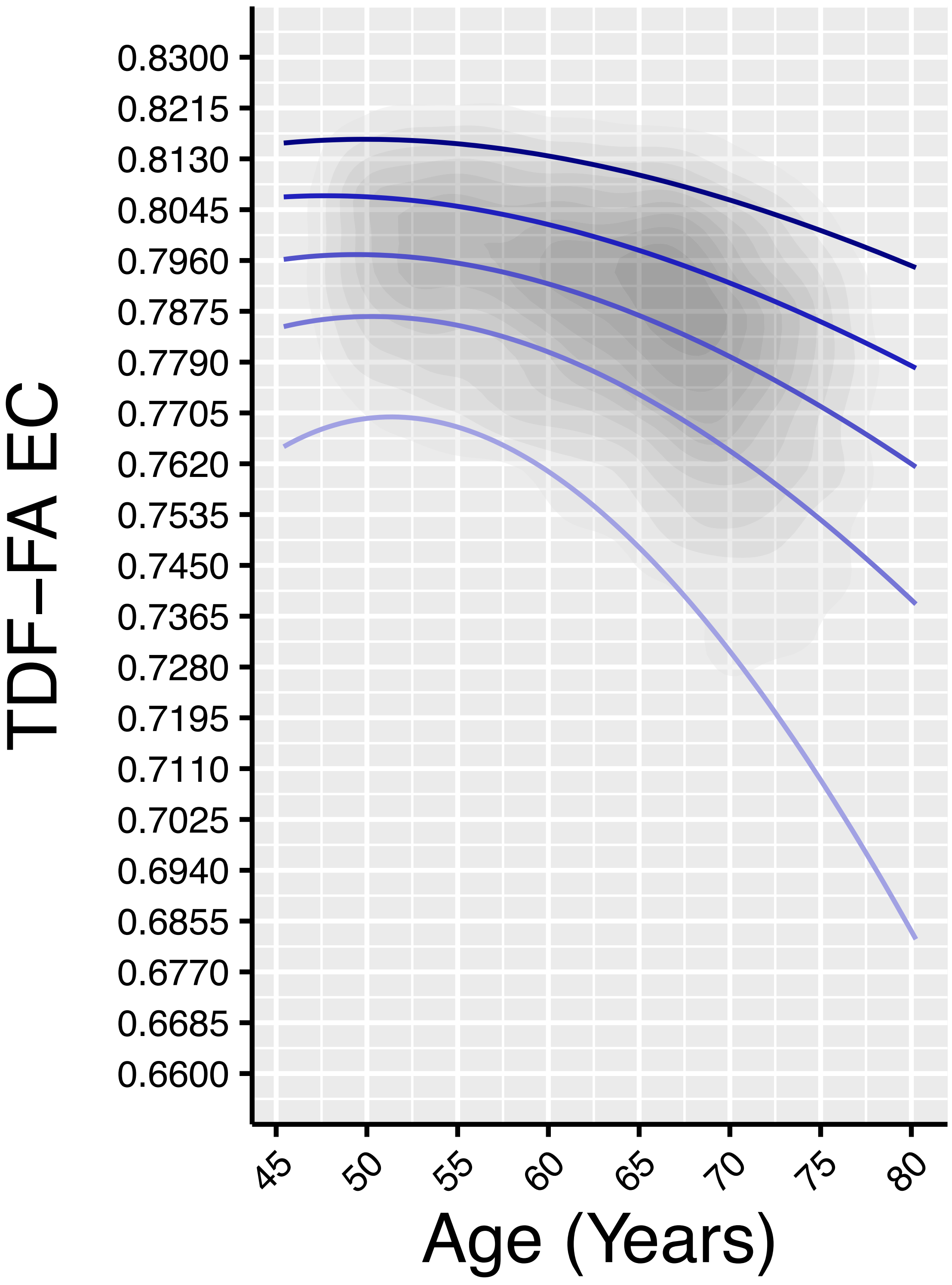

Figure S164. Full size normative centile reference curves calculated for the external capsule tract for TDF-FA in males. Solid colored lines, ordered from lightest to darkest, indicate the following centiles: 5th, 25th, 50th, 75th, 95th. Gray overlay reflects kernel density (darker=greater degree of data point overlap). EC = external capsule.

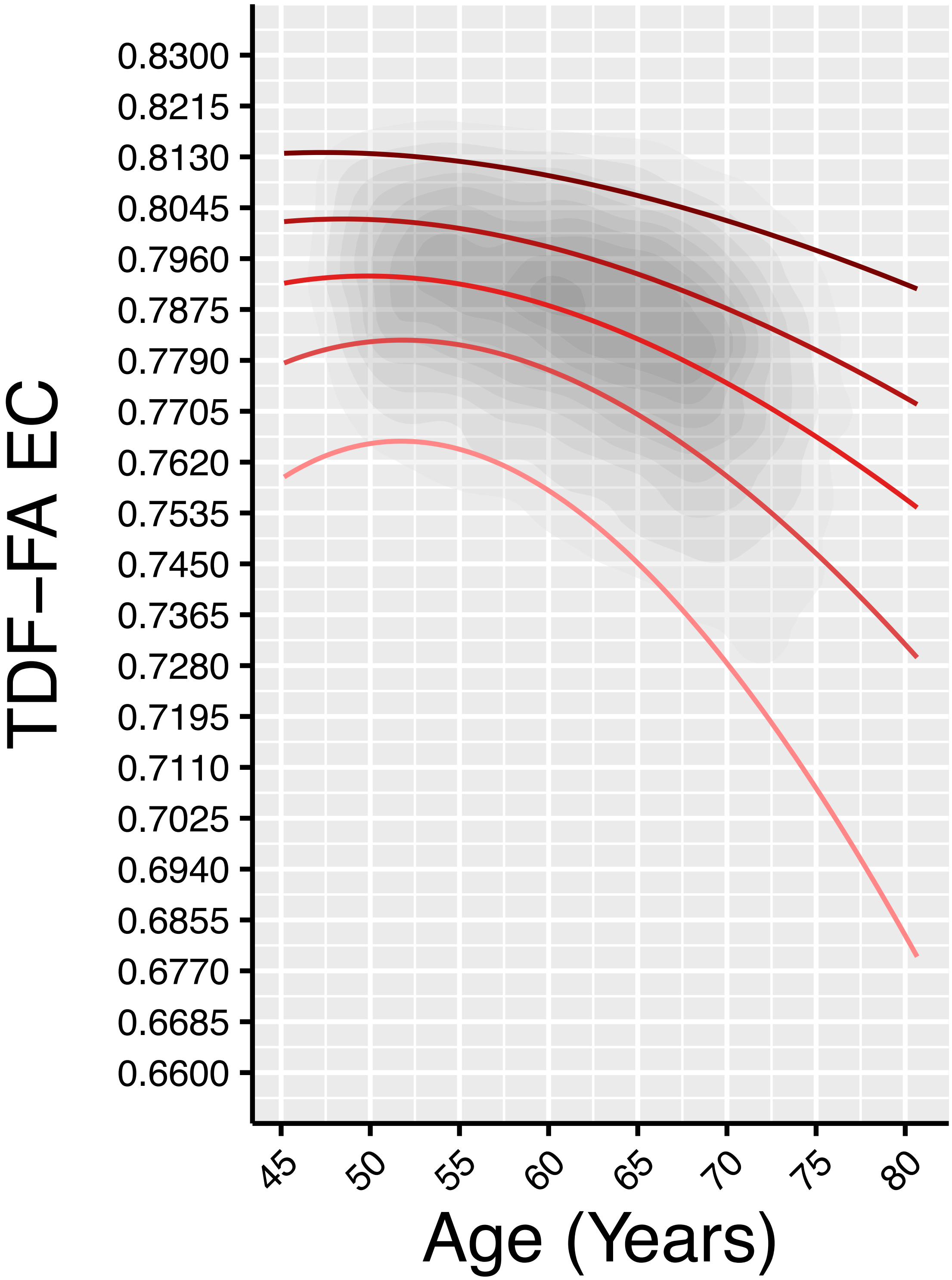

Figure S165. Full size normative centile reference curves calculated for the external capsule tract for TDF-FA in females. Solid colored lines, ordered from lightest to darkest, indicate the following centiles: 5th, 25th, 50th, 75th, 95th. Gray overlay reflects kernel density (darker=greater degree of data point overlap). EC = external capsule.

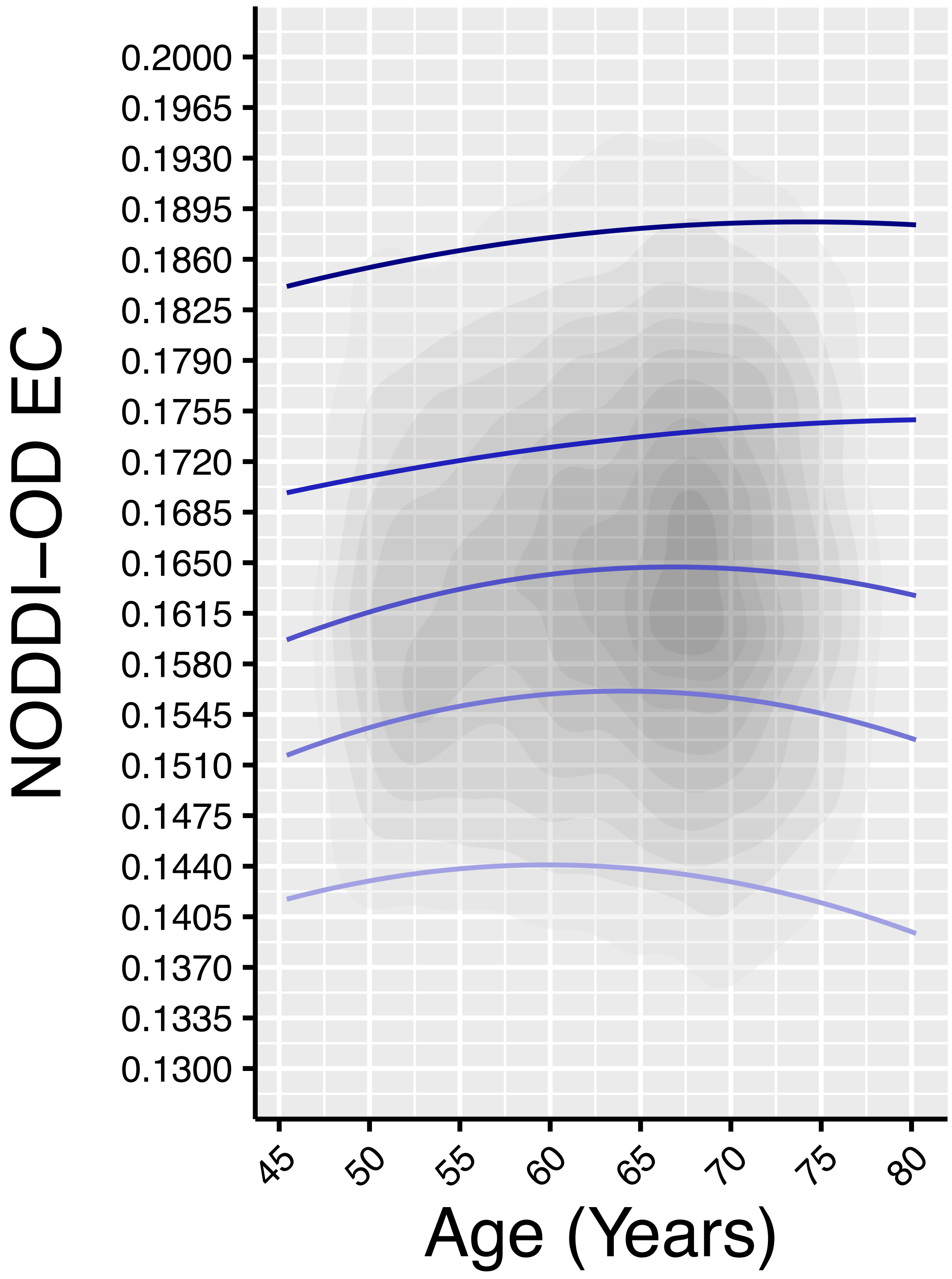

Figure S166. Full size normative centile reference curves calculated for the external capsule tract for NODDI-OD in males. Solid colored lines, ordered from lightest to darkest, indicate the following centiles: 5th, 25th, 50th, 75th, 95th. Gray overlay reflects kernel density (darker=greater degree of data point overlap). EC = external capsule.

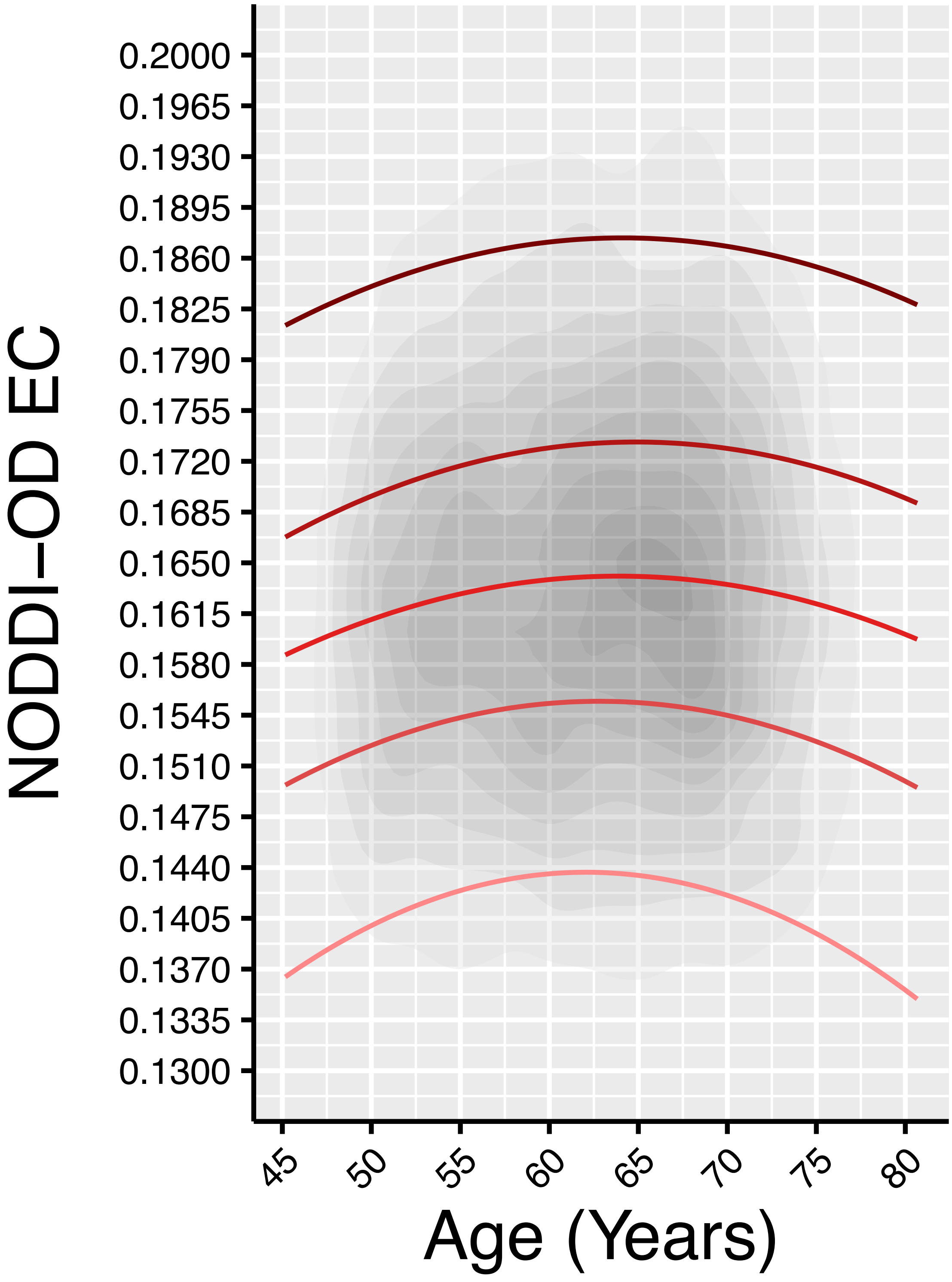

Figure S167. Full size normative centile reference curves calculated for the external capsule tract for NODDI-OD in females. Solid colored lines, ordered from lightest to darkest, indicate the following centiles: 5th, 25th, 50th, 75th, 95th. Gray overlay reflects kernel density (darker=greater degree of data point overlap). EC = external capsule.

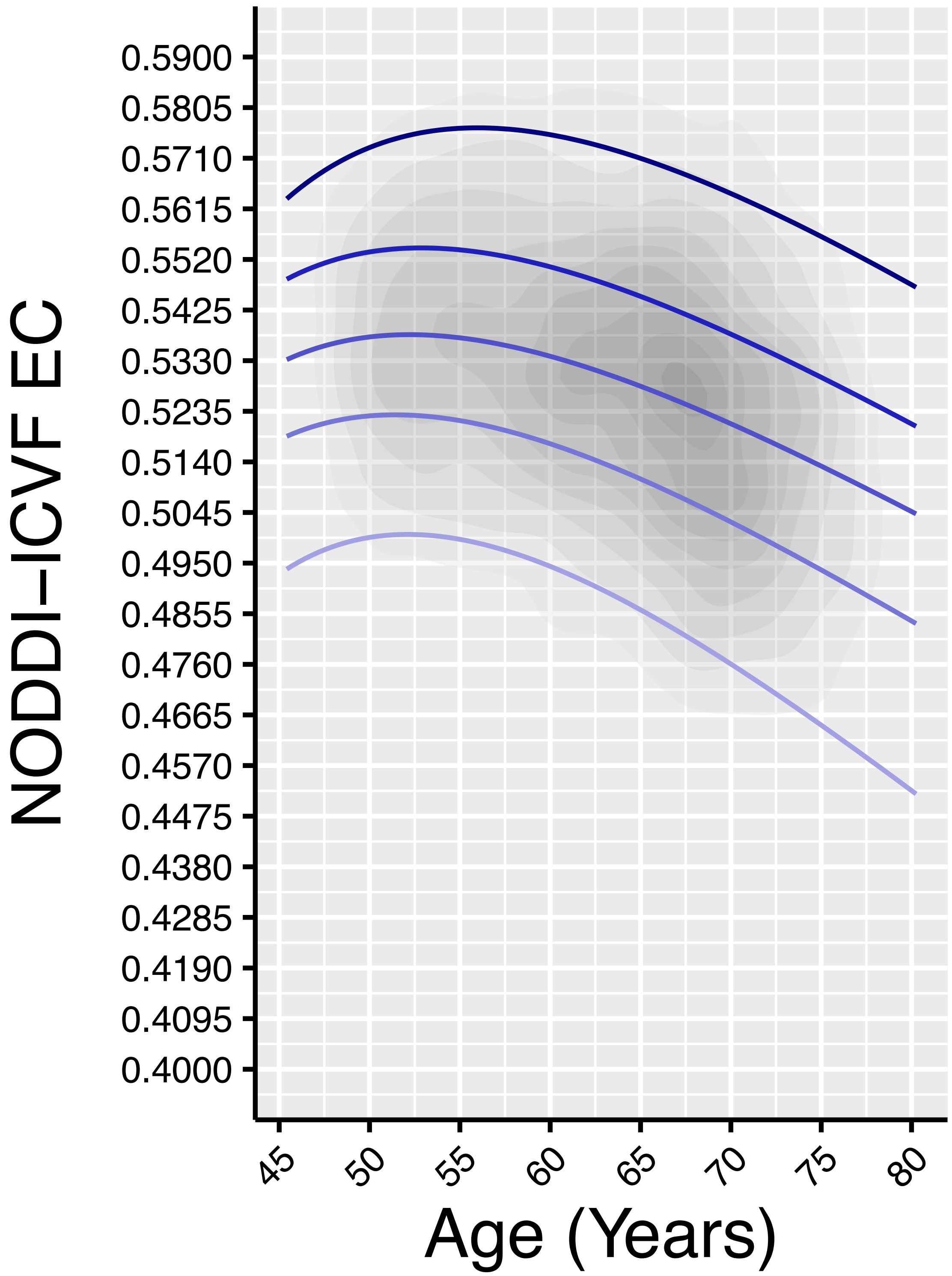

Figure S168. Full size normative centile reference curves calculated for the external capsule tract for NODDI-ICVF in males. Solid colored lines, ordered from lightest to darkest, indicate the following centiles: 5th, 25th, 50th, 75th, 95th. Gray overlay reflects kernel density (darker=greater degree of data point overlap). EC = external capsule.

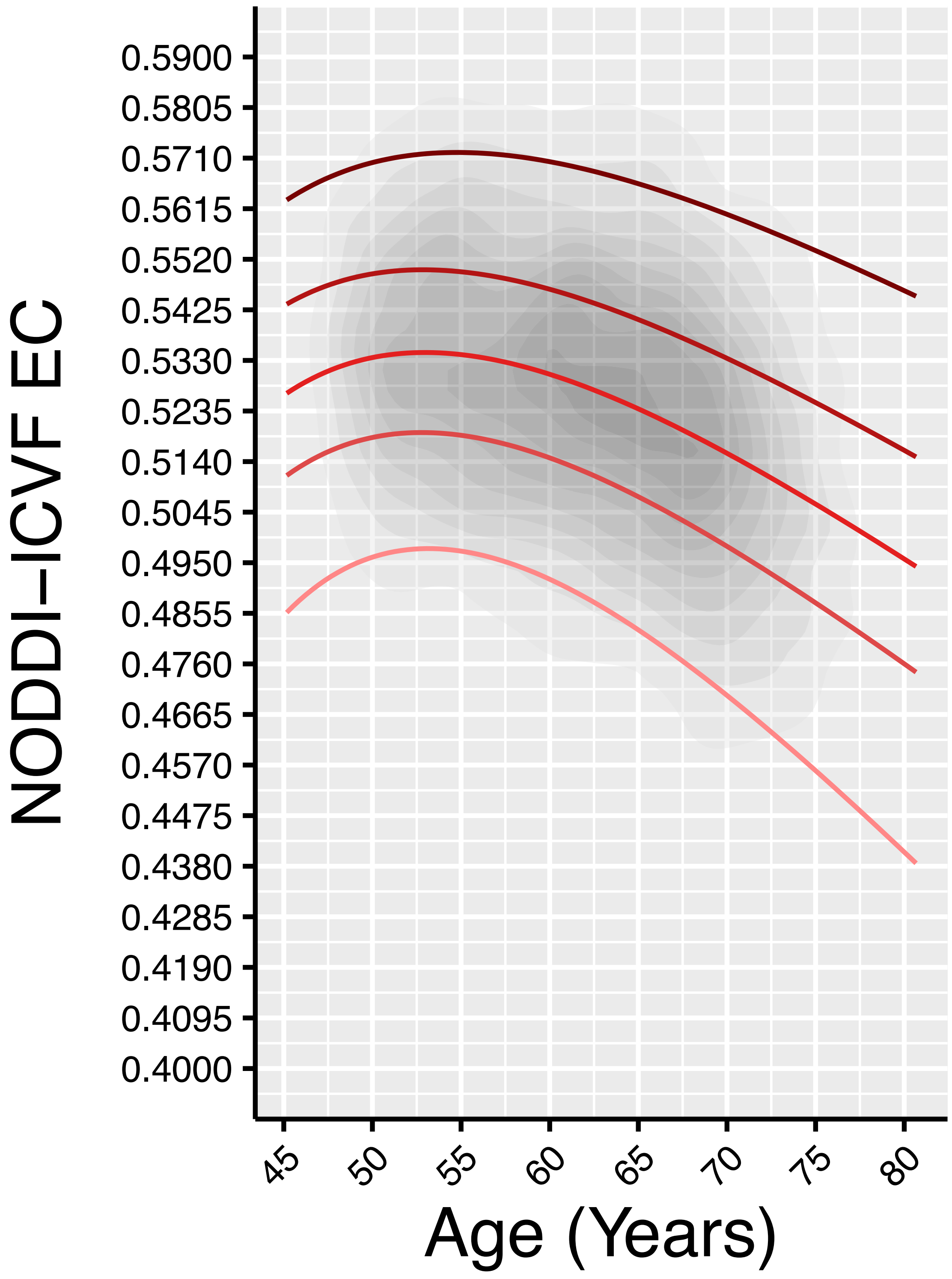

Figure S169. Full size normative centile reference curves calculated for the external capsule tract for NODDI-ICVF in females. Solid colored lines, ordered from lightest to darkest, indicate the following centiles: 5th, 25th, 50th, 75th, 95th. Gray overlay reflects kernel density (darker=greater degree of data point overlap). EC = external capsule.

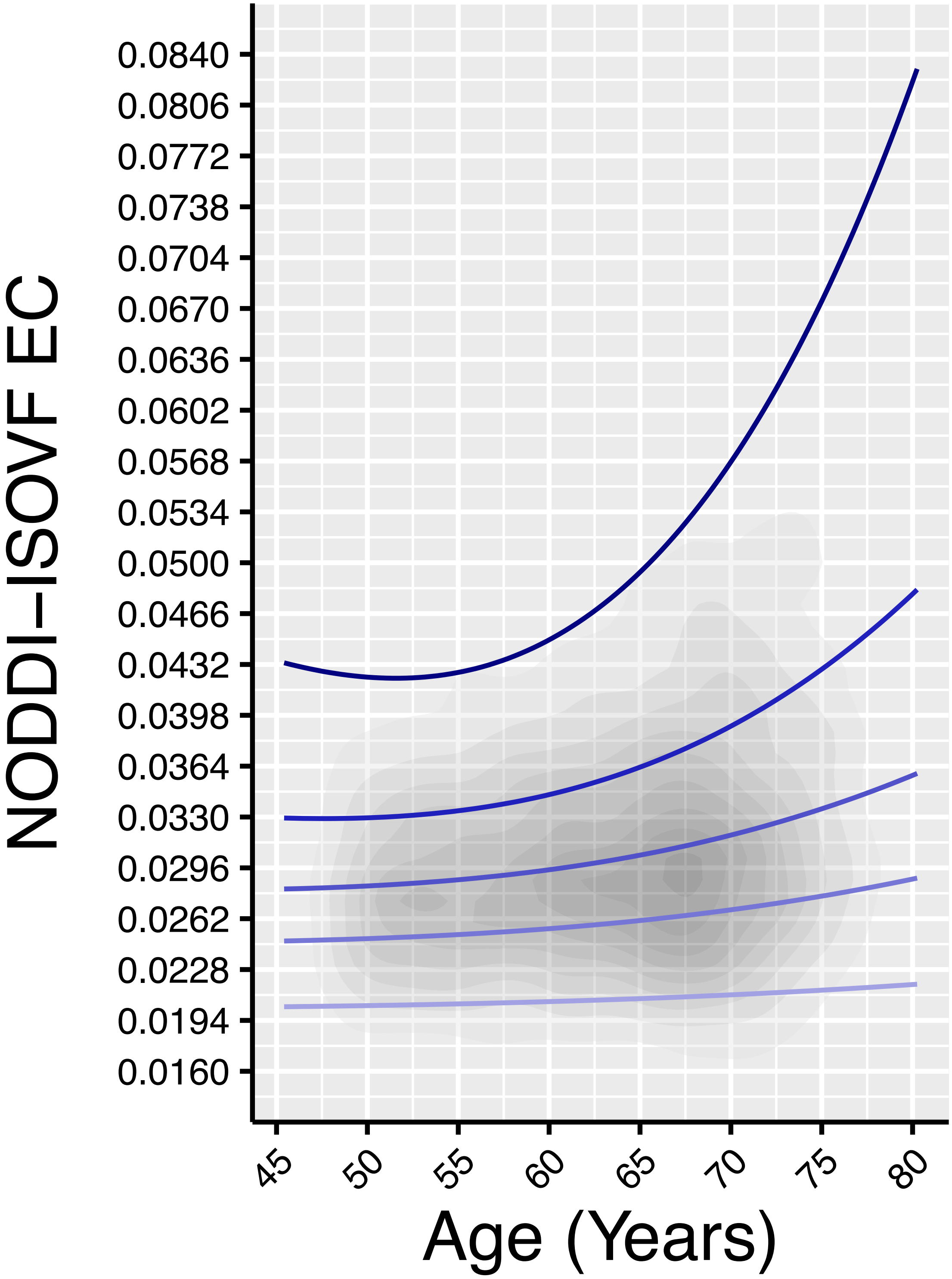

Figure S170. Full size normative centile reference curves calculated for the external capsule tract for NODDI-ISOVF in males. Solid colored lines, ordered from lightest to darkest, indicate the following centiles: 5th, 25th, 50th, 75th, 95th. Gray overlay reflects kernel density (darker=greater degree of data point overlap). EC = external capsule.

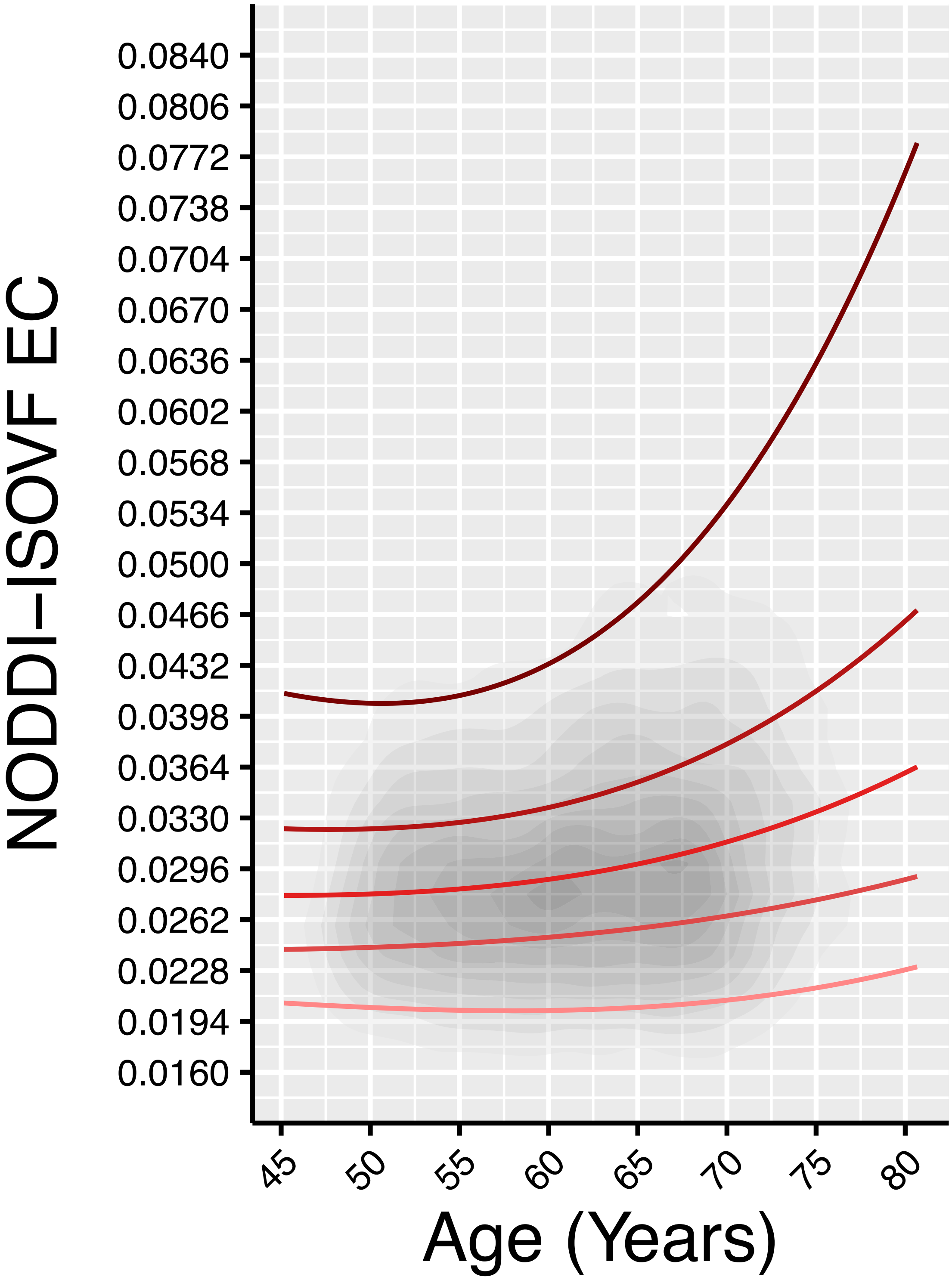

Figure S171. Full size normative centile reference curves calculated for the external capsule tract for NODDI-ISOVF in females. Solid colored lines, ordered from lightest to darkest, indicate the following centiles: 5th, 25th, 50th, 75th, 95th. Gray overlay reflects kernel density (darker=greater degree of data point overlap). EC = external capsule.

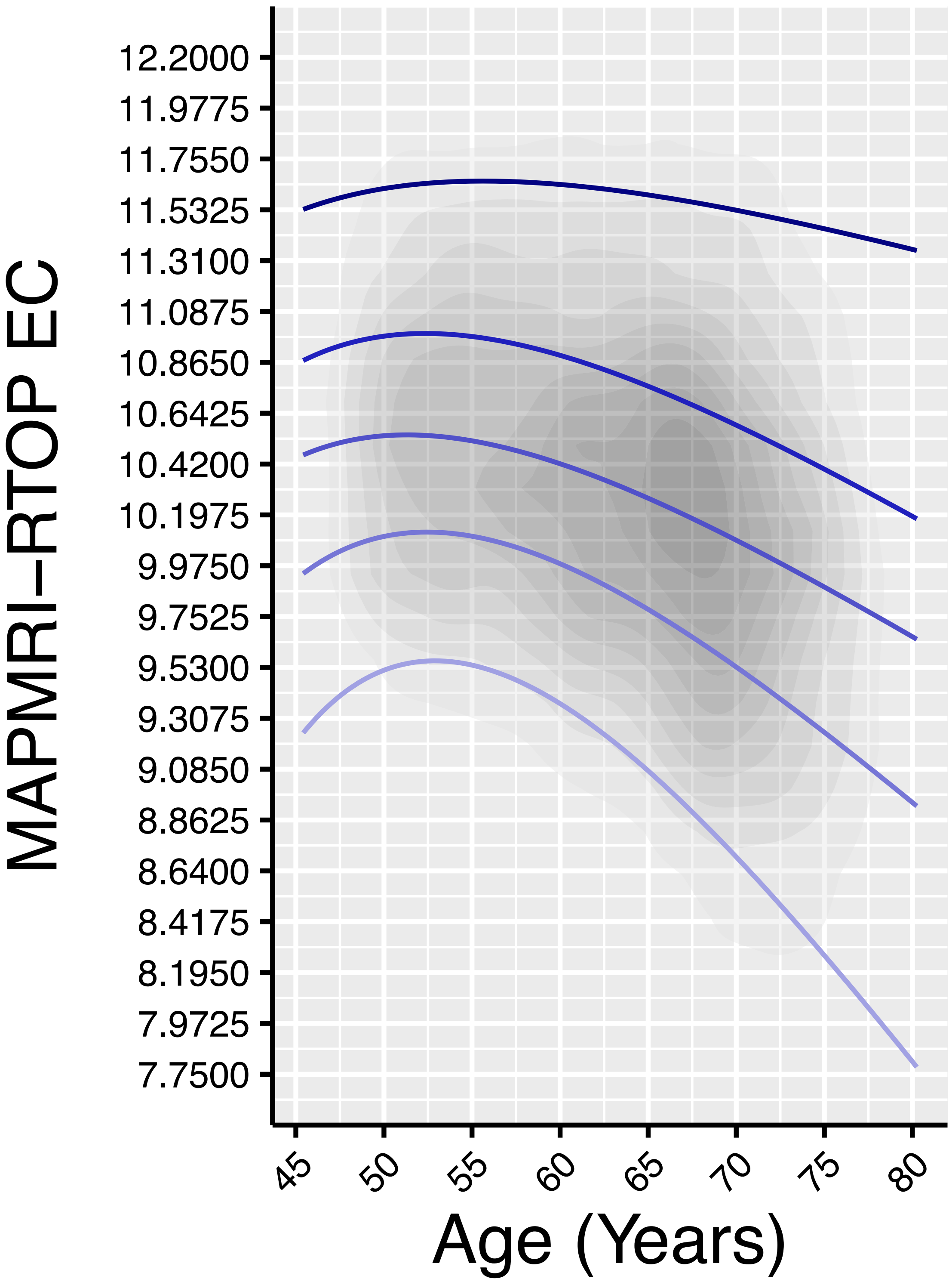

Figure S172. Full size normative centile reference curves calculated for the external capsule tract for MAPMRI-RTOP in males. Solid colored lines, ordered from lightest to darkest, indicate the following centiles: 5th, 25th, 50th, 75th, 95th. Gray overlay reflects kernel density (darker=greater degree of data point overlap). EC = external capsule.

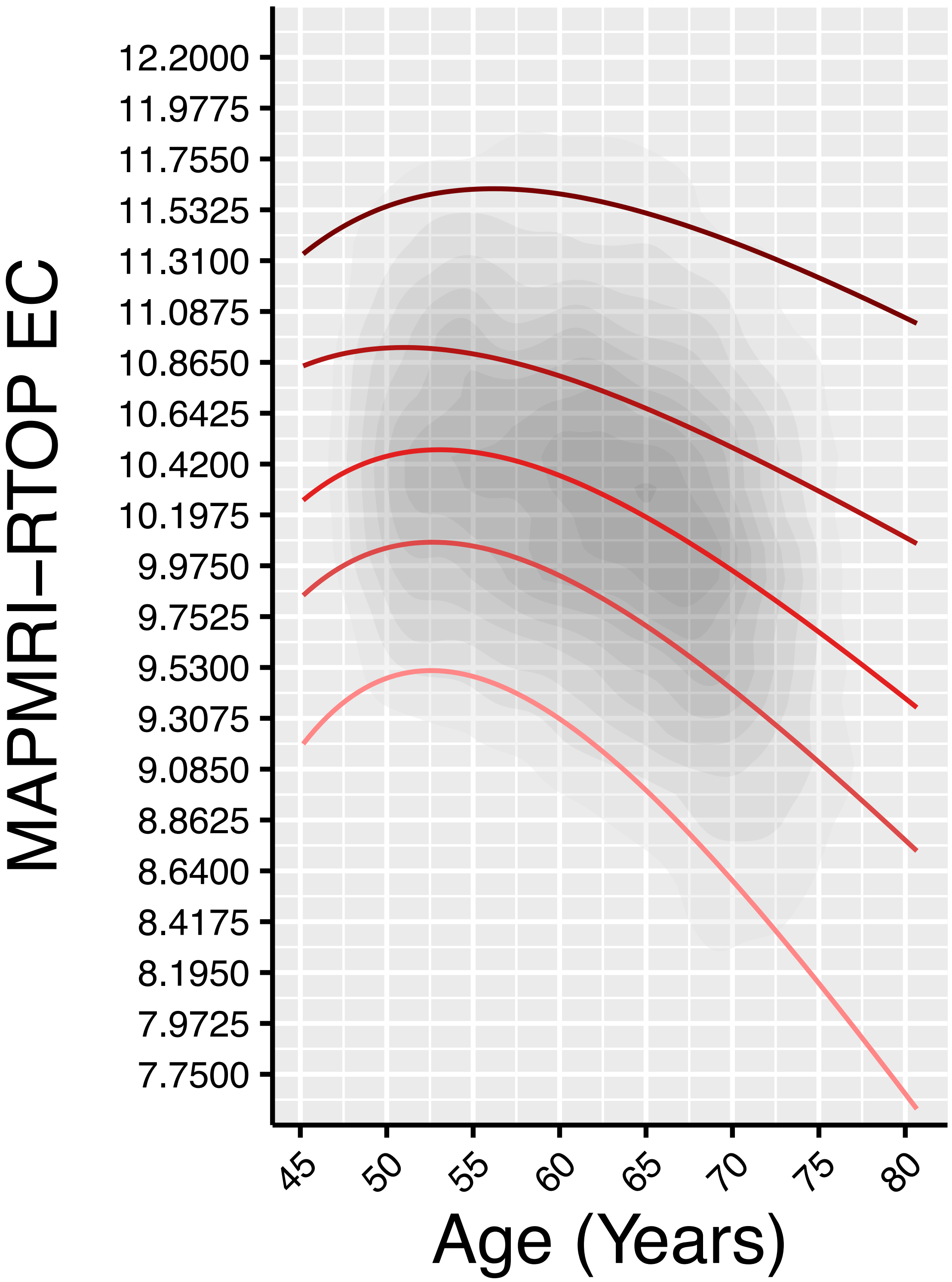

Figure S173. Full size normative centile reference curves calculated for the external capsule tract for MAPMRI-RTOP in females. Solid colored lines, ordered from lightest to darkest, indicate the following centiles: 5th, 25th, 50th, 75th, 95th. Gray overlay reflects kernel density (darker=greater degree of data point overlap). EC = external capsule.

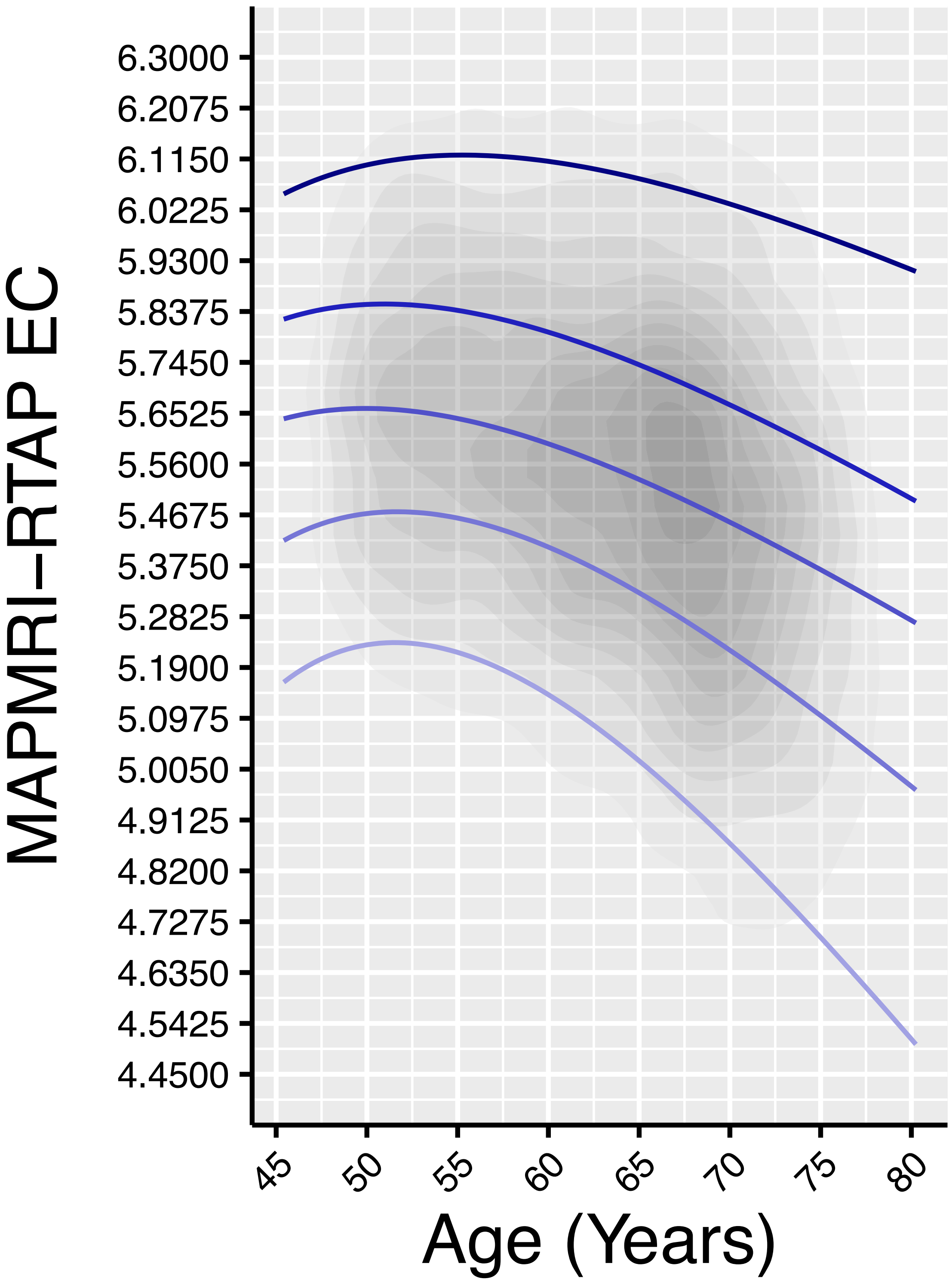

Figure S174. Full size normative centile reference curves calculated for the external capsule tract for MAPMRI-RTAP in males. Solid colored lines, ordered from lightest to darkest, indicate the following centiles: 5th, 25th, 50th, 75th, 95th. Gray overlay reflects kernel density (darker=greater degree of data point overlap). EC = external capsule.

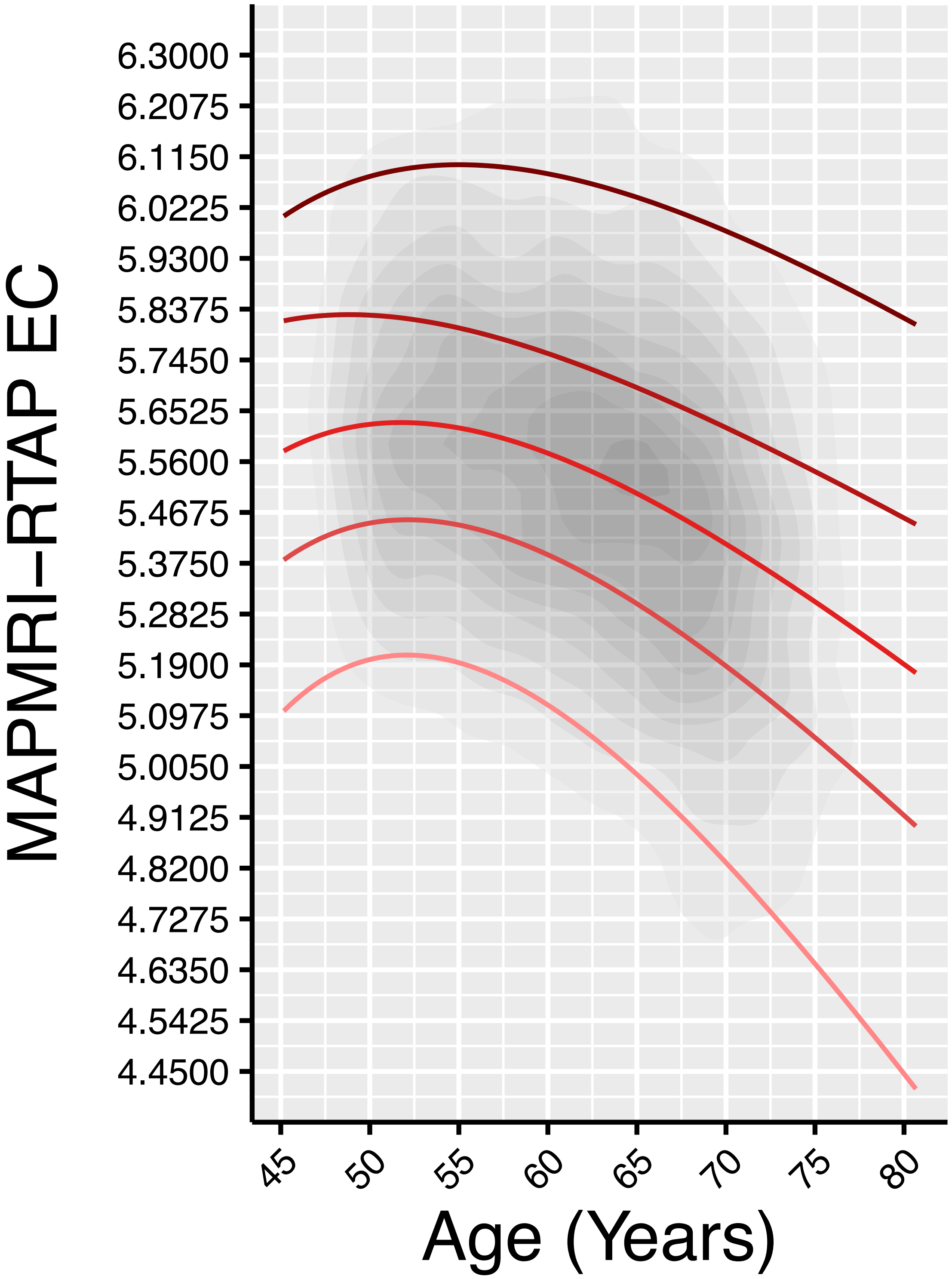

Figure S175. Full size normative centile reference curves calculated for the external capsule tract for MAPMRI-RTAP in females. Solid colored lines, ordered from lightest to darkest, indicate the following centiles: 5th, 25th, 50th, 75th, 95th. Gray overlay reflects kernel density (darker=greater degree of data point overlap). EC = external capsule.

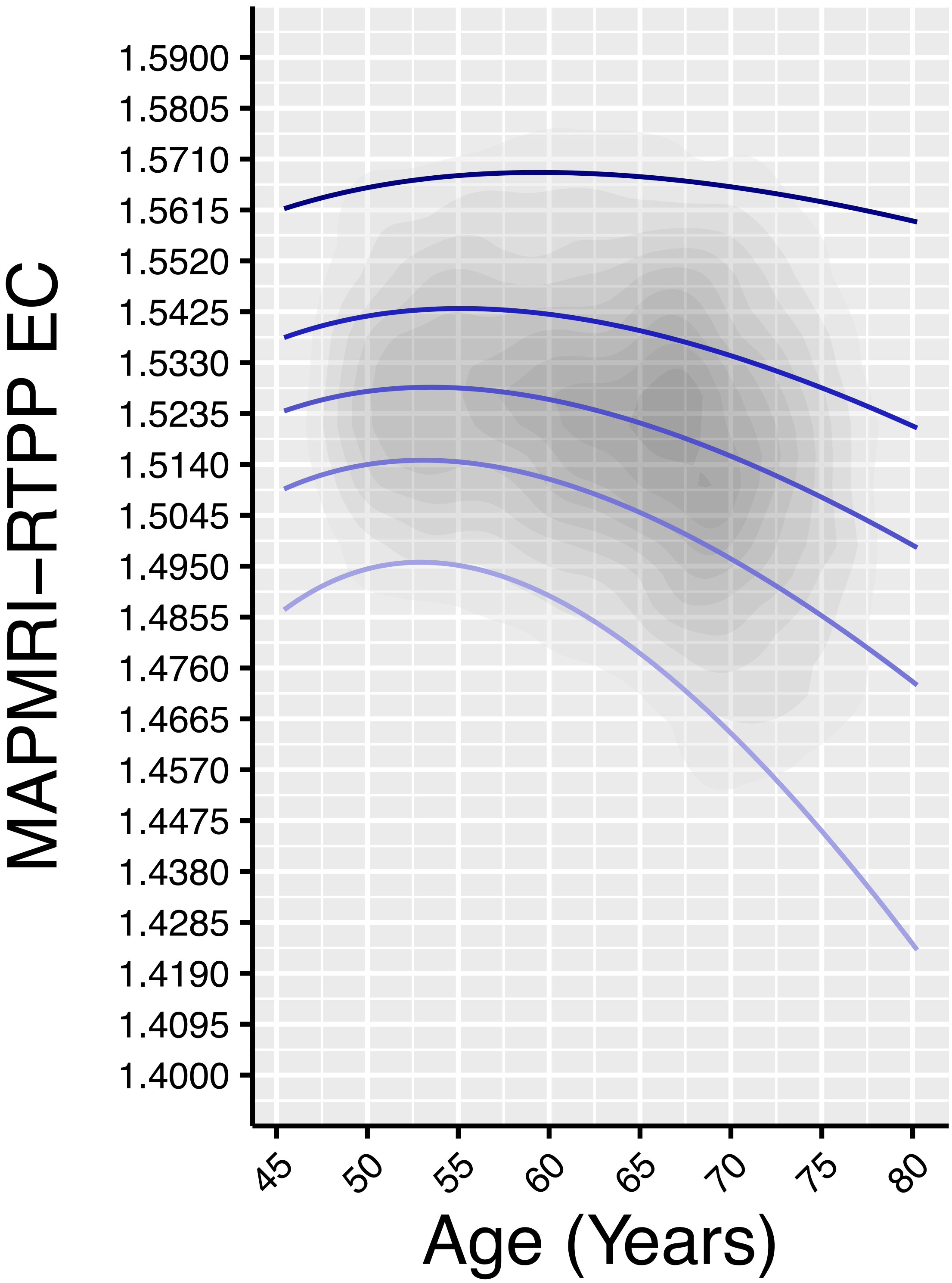

Figure S176. Full size normative centile reference curves calculated for the external capsule tract for MAPMRI-RTPP in males. Solid colored lines, ordered from lightest to darkest, indicate the following centiles: 5th, 25th, 50th, 75th, 95th. Gray overlay reflects kernel density (darker=greater degree of data point overlap). EC = external capsule.

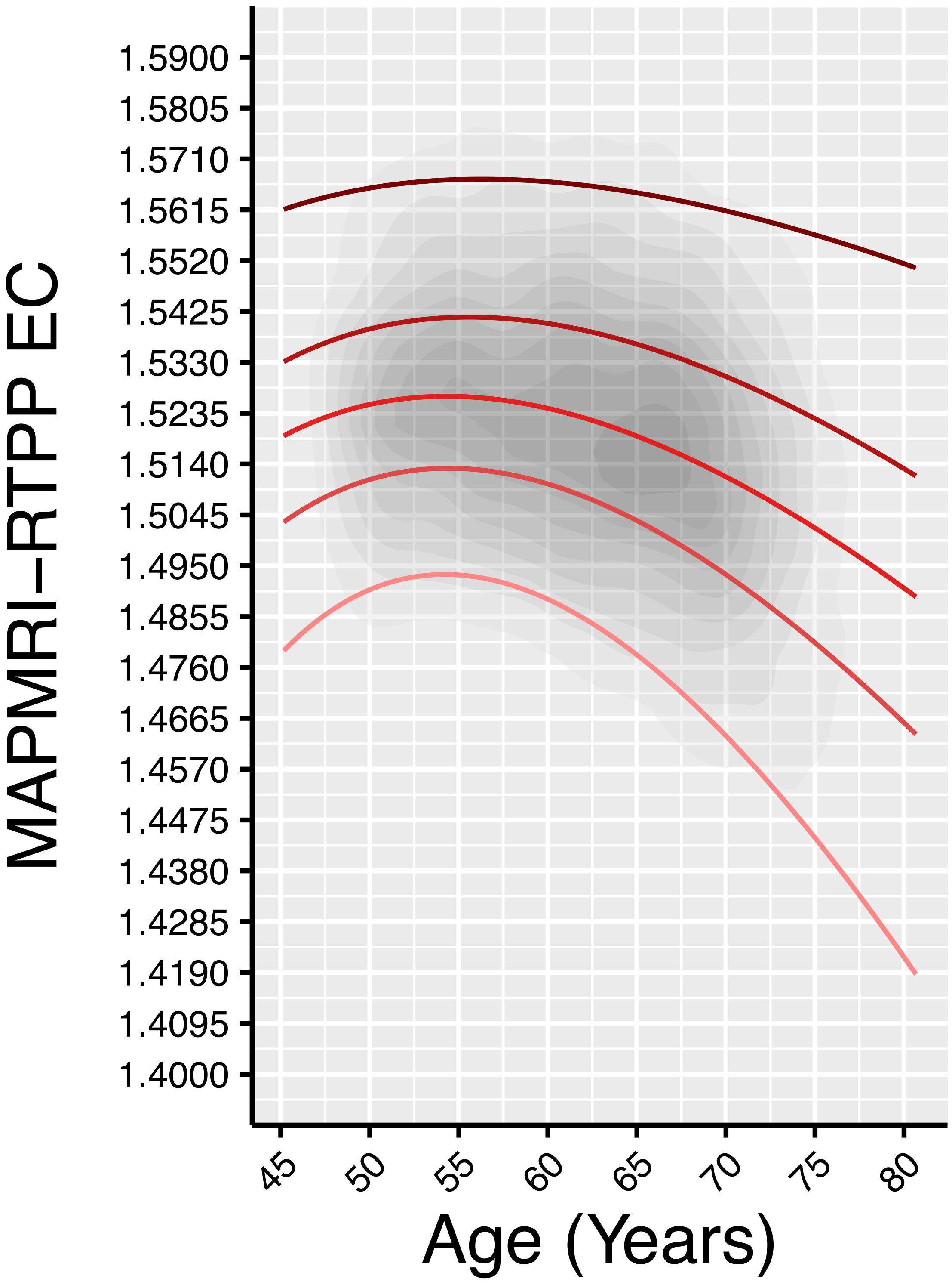

Figure S177. Full size normative centile reference curves calculated for the external capsule tract for MAPMRI-RTPP in females. Solid colored lines, ordered from lightest to darkest, indicate the following centiles: 5th, 25th, 50th, 75th, 95th. Gray overlay reflects kernel density (darker=greater degree of data point overlap). EC = external capsule.

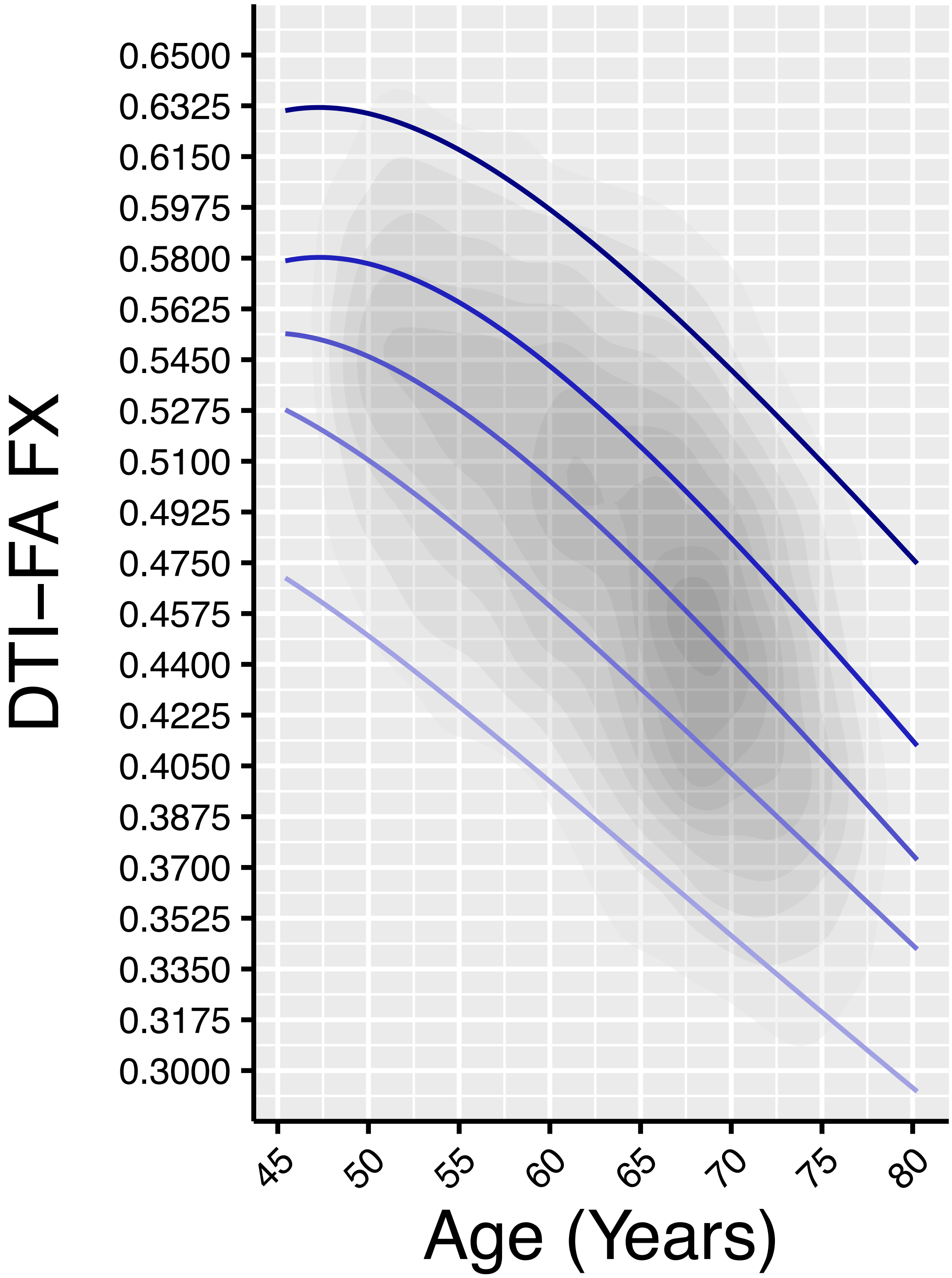

Figure S178. Full size normative centile reference curves calculated for the fornix (body) tract for DTI-FA in males. Solid colored lines, ordered from lightest to darkest, indicate the following centiles: 5th, 25th, 50th, 75th, 95th. Gray overlay reflects kernel density (darker=greater degree of data point overlap). FX = fornix (body).

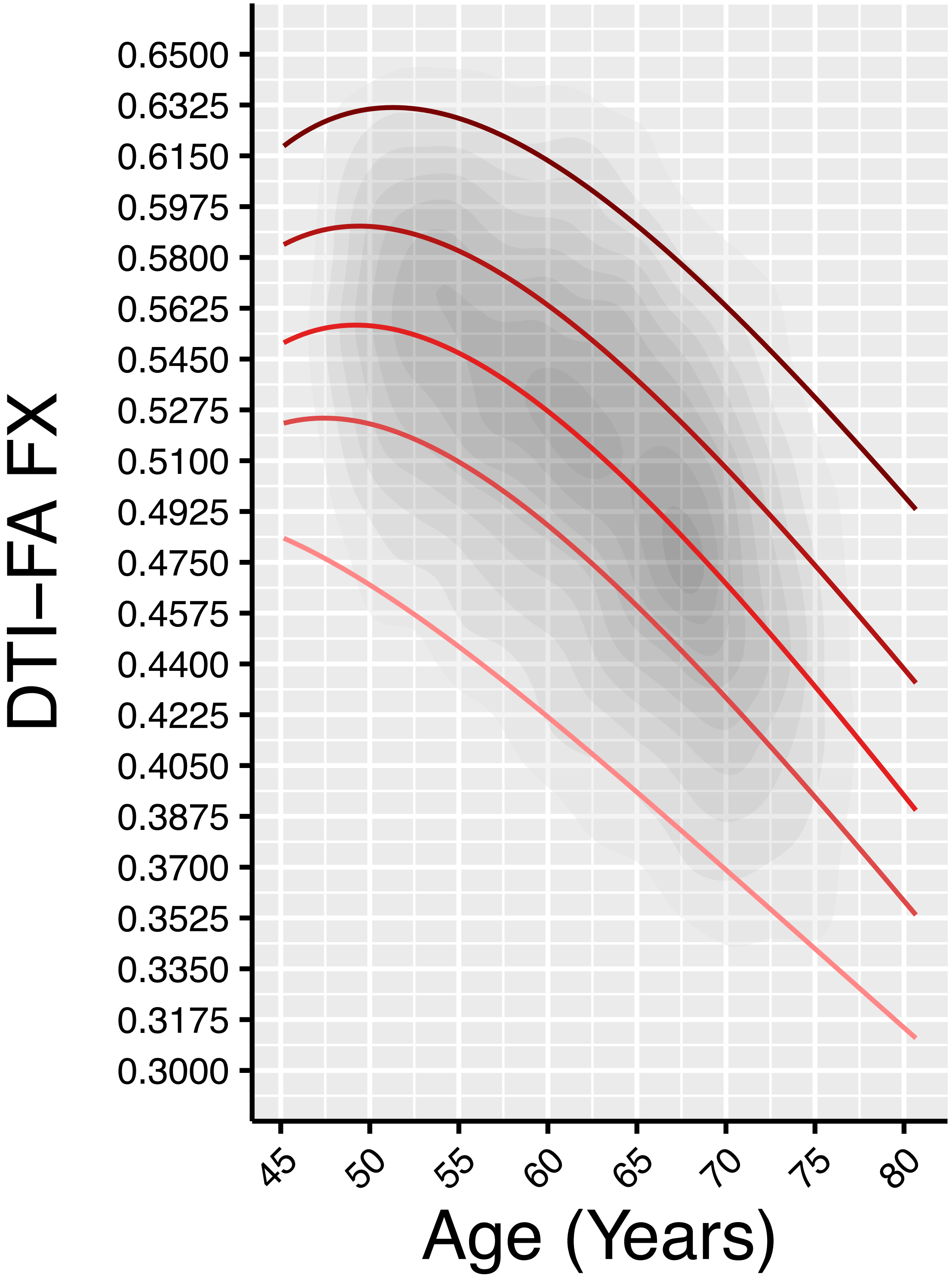

Figure S179. Full size normative centile reference curves calculated for the fornix (body) tract for DTI-FA in females. Solid colored lines, ordered from lightest to darkest, indicate the following centiles: 5th, 25th, 50th, 75th, 95th. Gray overlay reflects kernel density (darker=greater degree of data point overlap). FX = fornix (body).

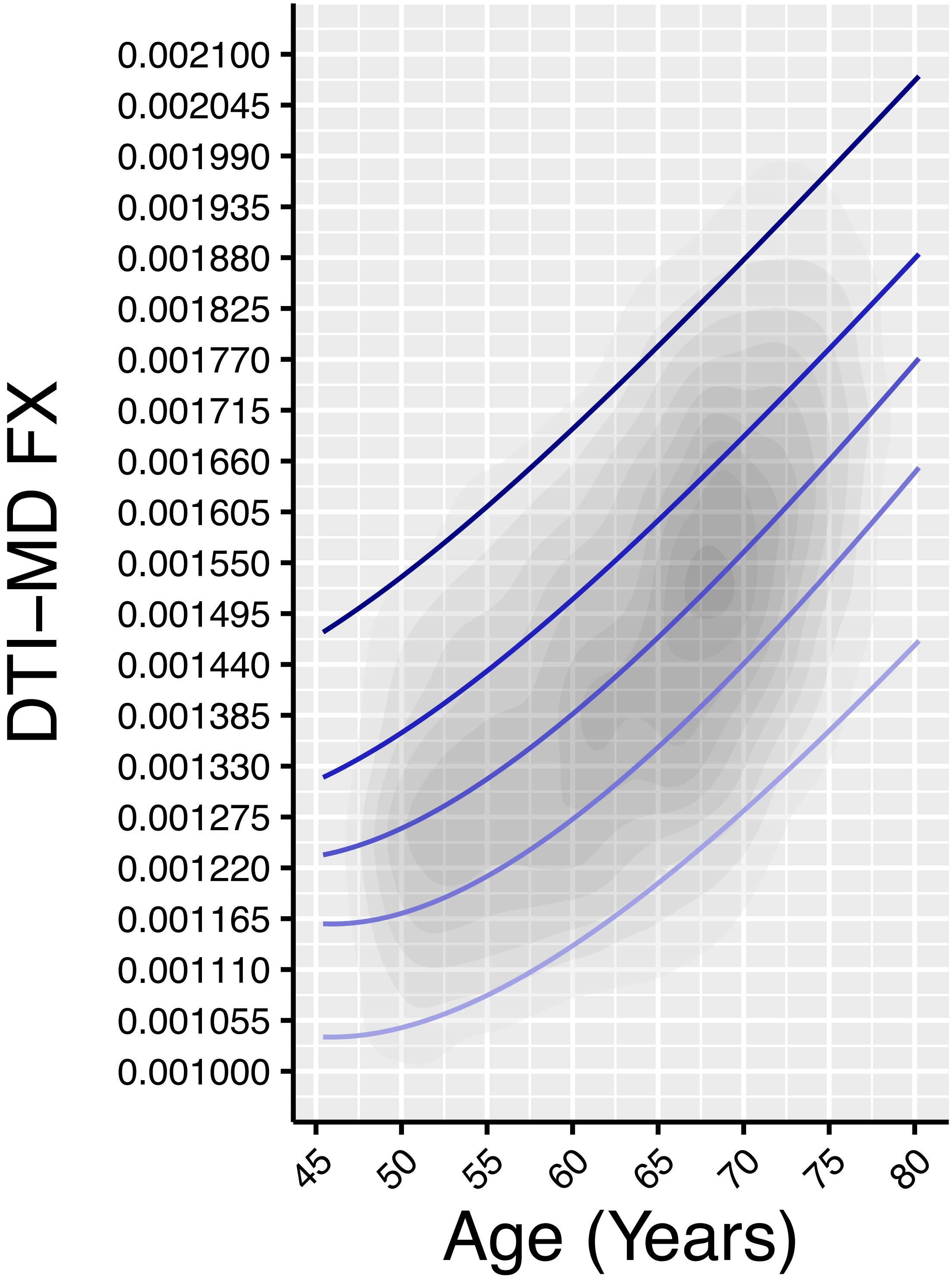

Figure S180. Full size normative centile reference curves calculated for the fornix (body) tract for DTI-MD in males. Solid colored lines, ordered from lightest to darkest, indicate the following centiles: 5th, 25th, 50th, 75th, 95th. Gray overlay reflects kernel density (darker=greater degree of data point overlap). FX = fornix (body).

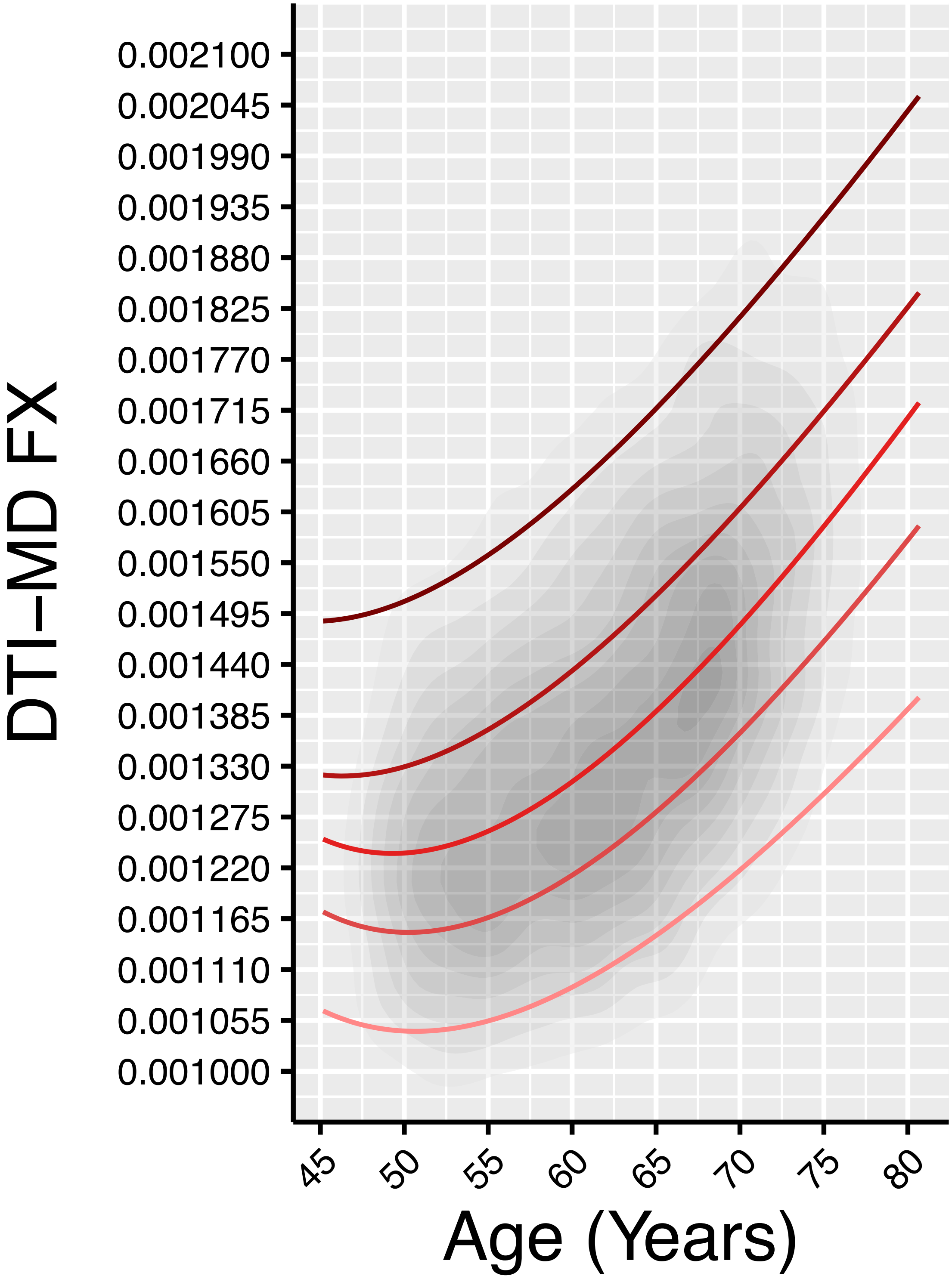

Figure S181. Full size normative centile reference curves calculated for the fornix (body) tract for DTI-MD in females. Solid colored lines, ordered from lightest to darkest, indicate the following centiles: 5th, 25th, 50th, 75th, 95th. Gray overlay reflects kernel density (darker=greater degree of data point overlap). FX = fornix (body).

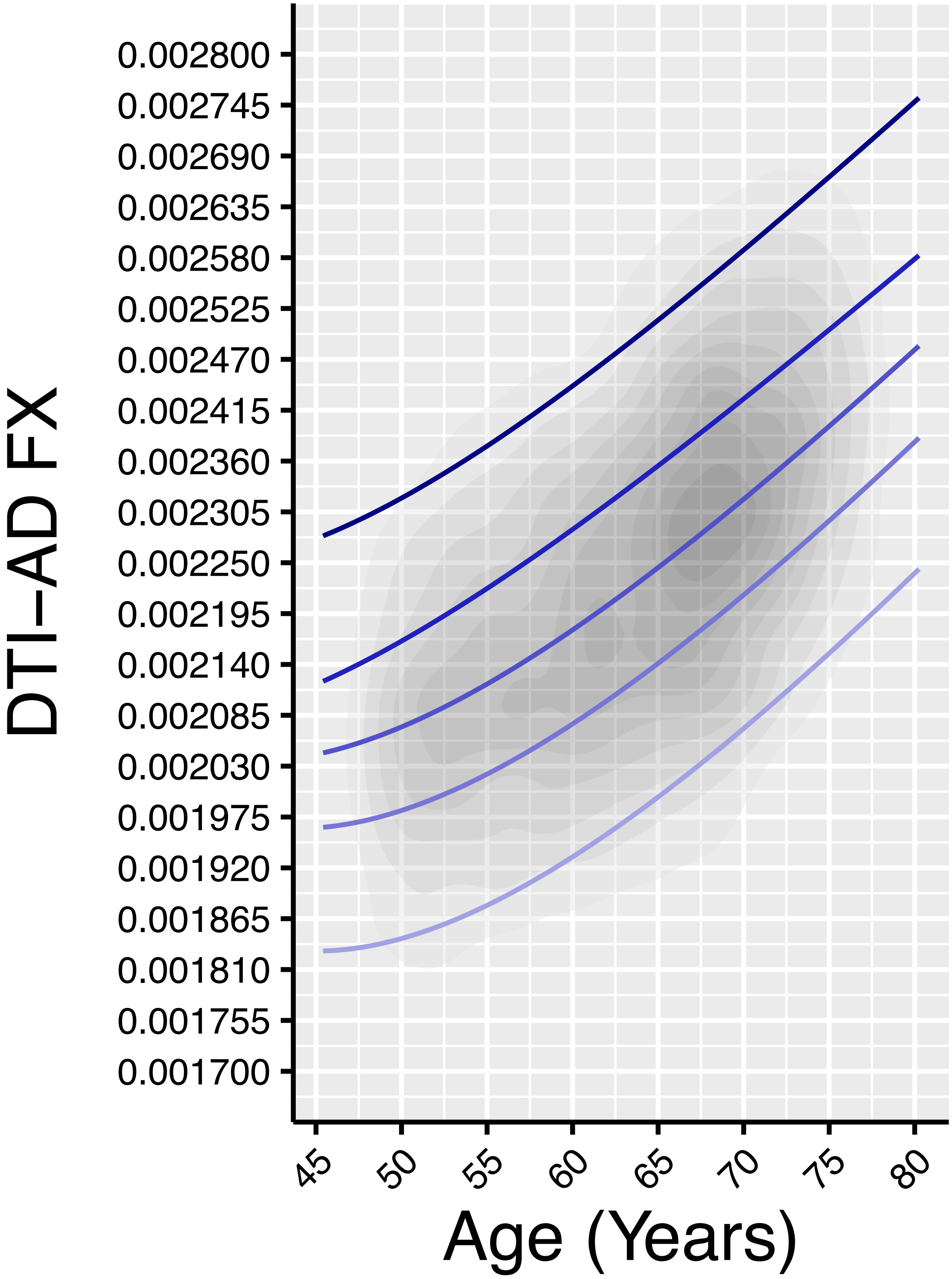

Figure S182. Full size normative centile reference curves calculated for the fornix (body) tract for DTI-AD in males. Solid colored lines, ordered from lightest to darkest, indicate the following centiles: 5th, 25th, 50th, 75th, 95th. Gray overlay reflects kernel density (darker=greater degree of data point overlap). FX = fornix (body).

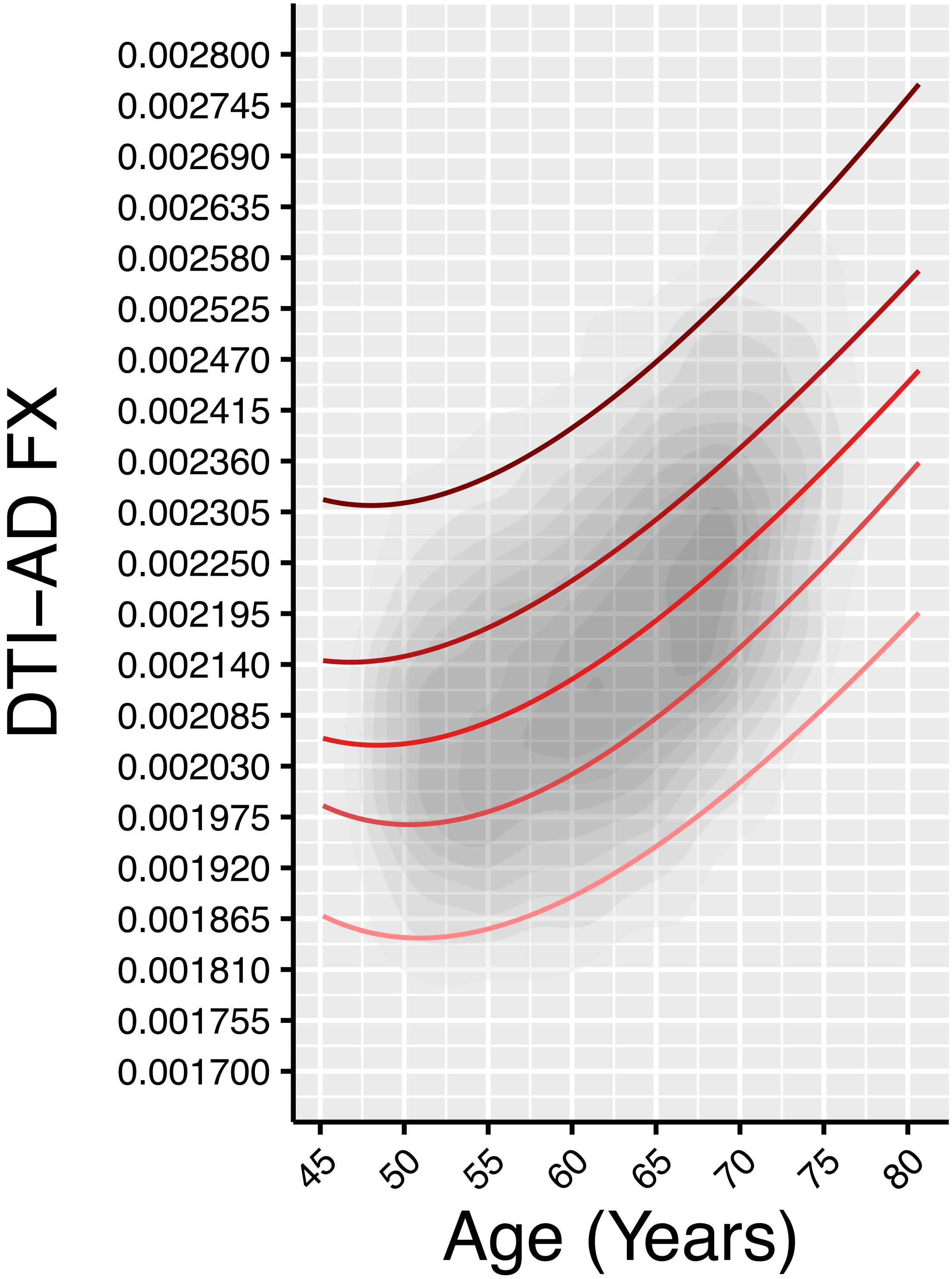

Figure S183. Full size normative centile reference curves calculated for the fornix (body) tract for DTI-AD in females. Solid colored lines, ordered from lightest to darkest, indicate the following centiles: 5th, 25th, 50th, 75th, 95th. Gray overlay reflects kernel density (darker=greater degree of data point overlap). FX = fornix (body).

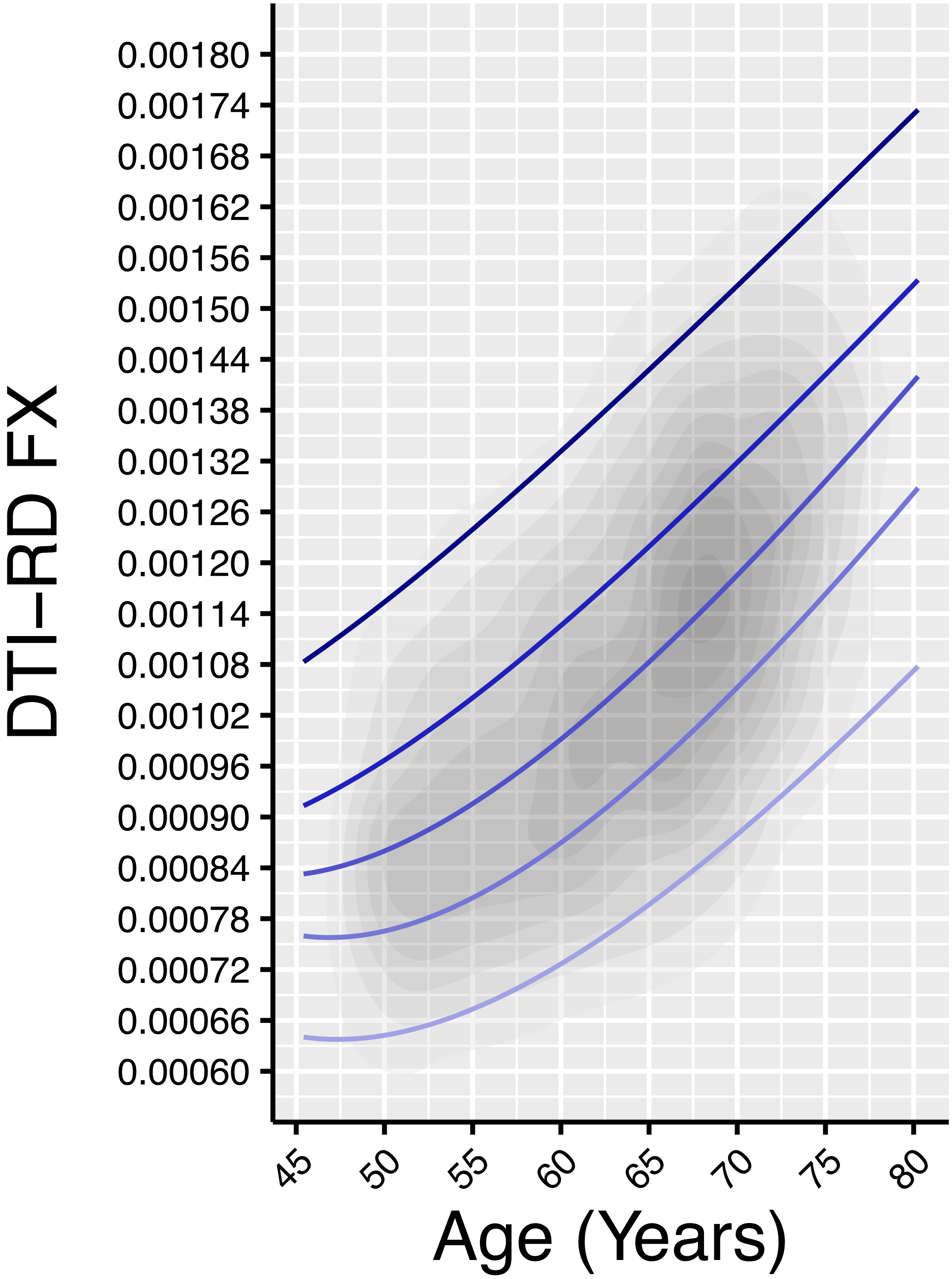

Figure S184. Full size normative centile reference curves calculated for the fornix (body) tract for DTI-RD in males. Solid colored lines, ordered from lightest to darkest, indicate the following centiles: 5th, 25th, 50th, 75th, 95th. Gray overlay reflects kernel density (darker=greater degree of data point overlap). FX = fornix (body).

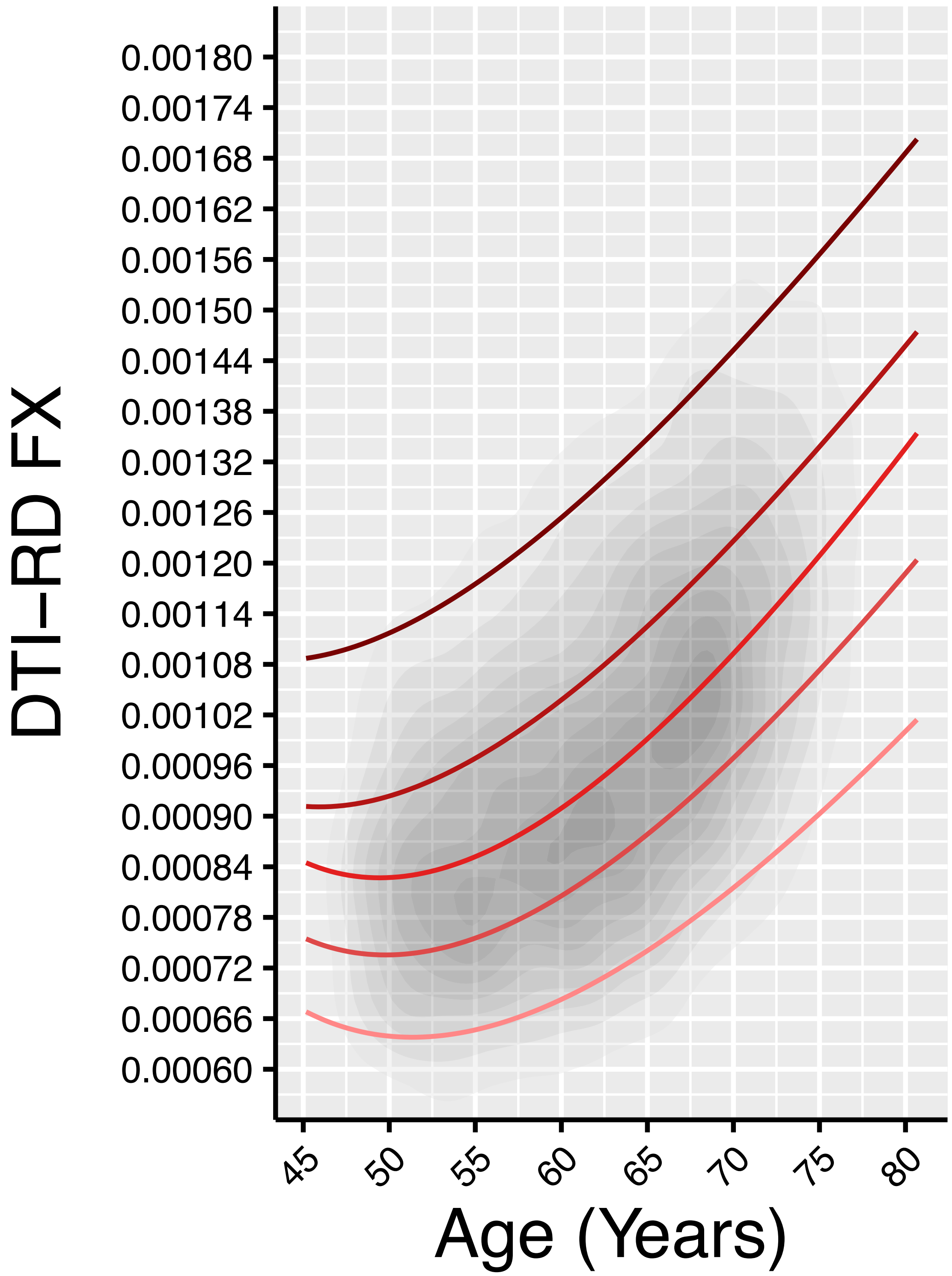

Figure S185. Full size normative centile reference curves calculated for the fornix (body) tract for DTI-RD in females. Solid colored lines, ordered from lightest to darkest, indicate the following centiles: 5th, 25th, 50th, 75th, 95th. Gray overlay reflects kernel density (darker=greater degree of data point overlap). FX = fornix (body).

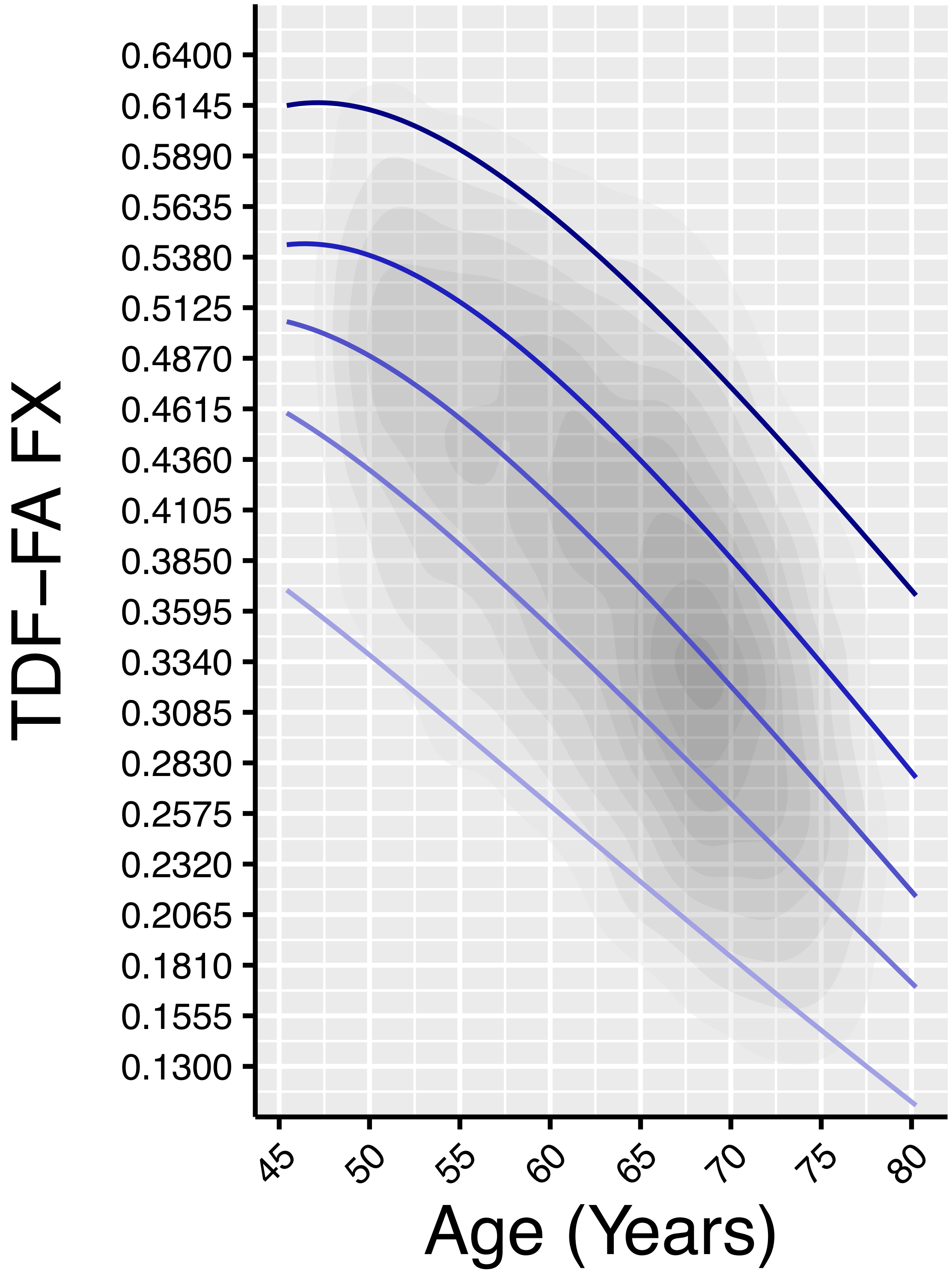

Figure S186. Full size normative centile reference curves calculated for the fornix (body) tract for TDF-FA in males. Solid colored lines, ordered from lightest to darkest, indicate the following centiles: 5th, 25th, 50th, 75th, 95th. Gray overlay reflects kernel density (darker=greater degree of data point overlap). FX = fornix (body).

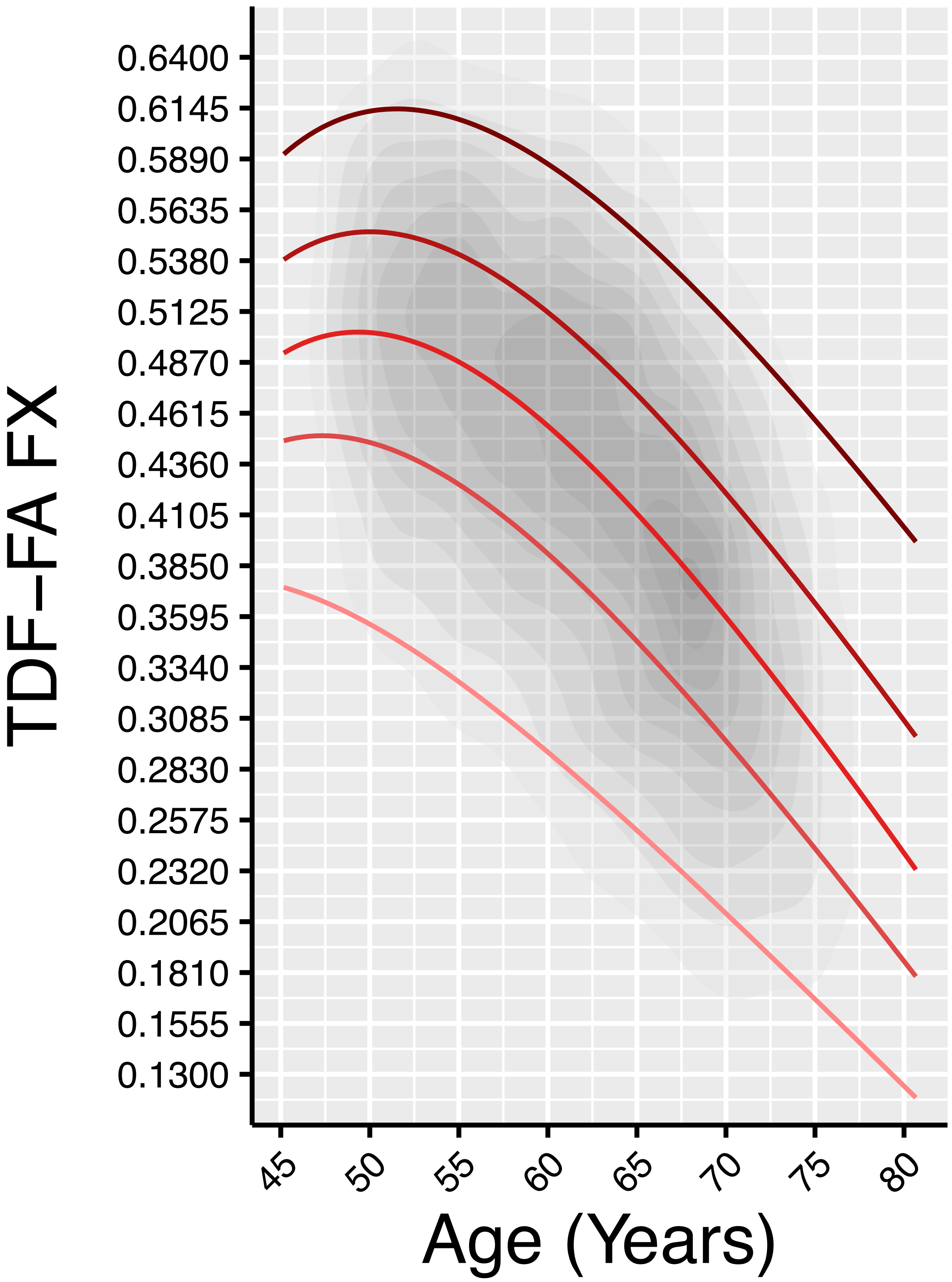

Figure S187. Full size normative centile reference curves calculated for the fornix (body) tract for TDF-FA in females. Solid colored lines, ordered from lightest to darkest, indicate the following centiles: 5th, 25th, 50th, 75th, 95th. Gray overlay reflects kernel density (darker=greater degree of data point overlap). FX = fornix (body).

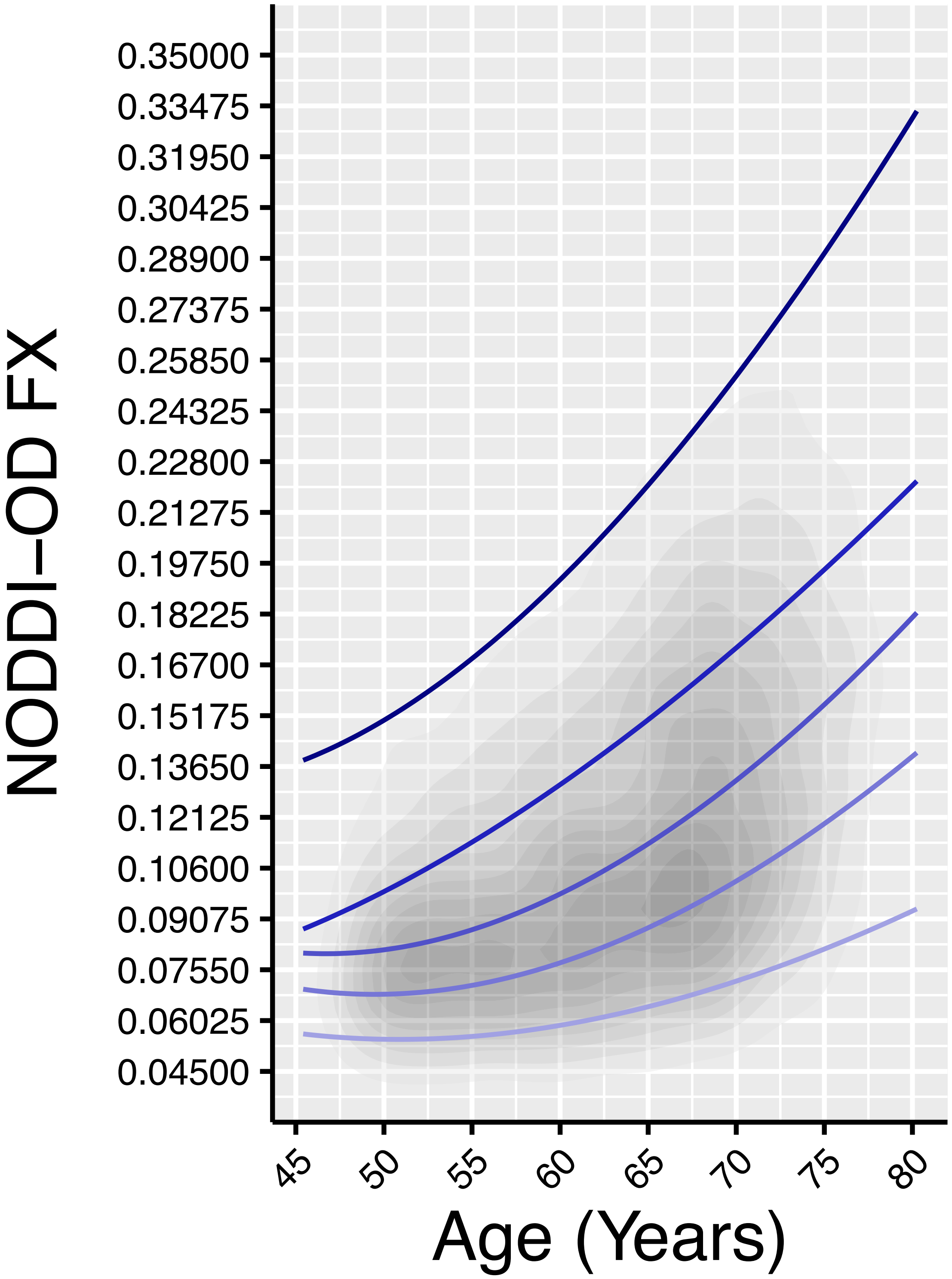

Figure S188. Full size normative centile reference curves calculated for the fornix (body) tract for NODDI-OD in males. Solid colored lines, ordered from lightest to darkest, indicate the following centiles: 5th, 25th, 50th, 75th, 95th. Gray overlay reflects kernel density (darker=greater degree of data point overlap). FX = fornix (body).

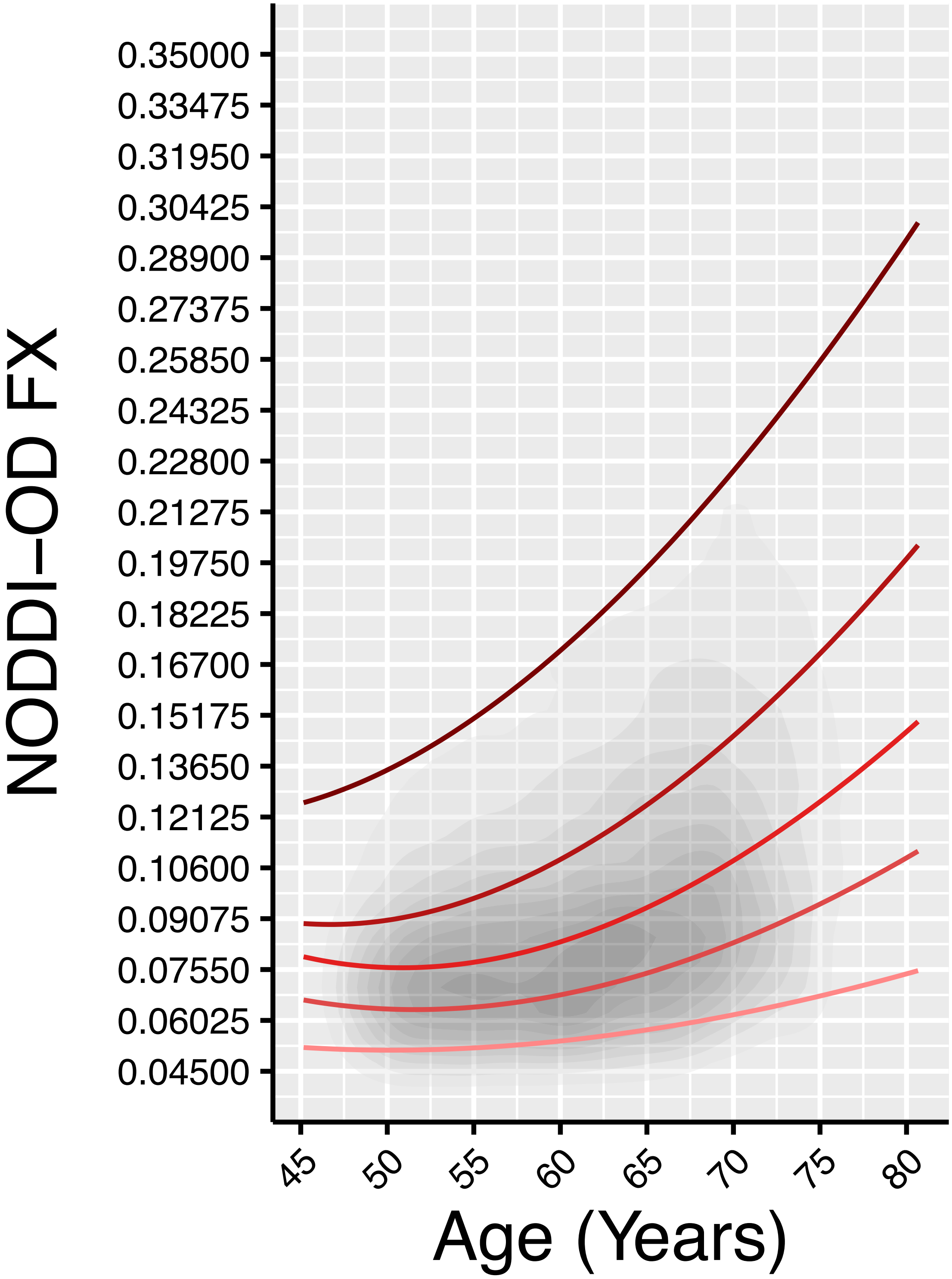

Figure S189. Full size normative centile reference curves calculated for the fornix (body) tract for NODDI-OD in females. Solid colored lines, ordered from lightest to darkest, indicate the following centiles: 5th, 25th, 50th, 75th, 95th. Gray overlay reflects kernel density (darker=greater degree of data point overlap). FX = fornix (body).

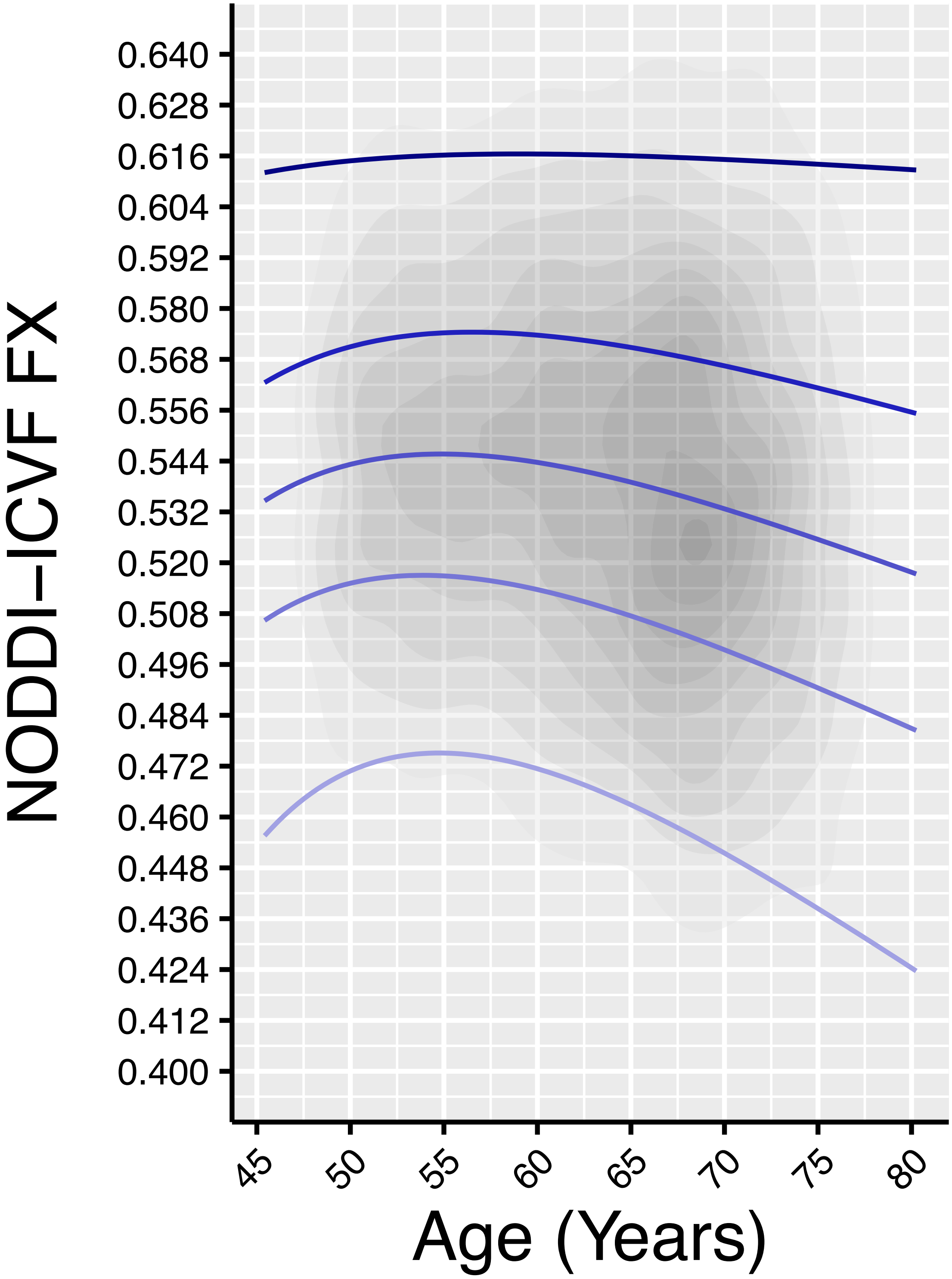

Figure S190. Full size normative centile reference curves calculated for the fornix (body) tract for NODDI-ICVF in males. Solid colored lines, ordered from lightest to darkest, indicate the following centiles: 5th, 25th, 50th, 75th, 95th. Gray overlay reflects kernel density (darker=greater degree of data point overlap). FX = fornix (body).

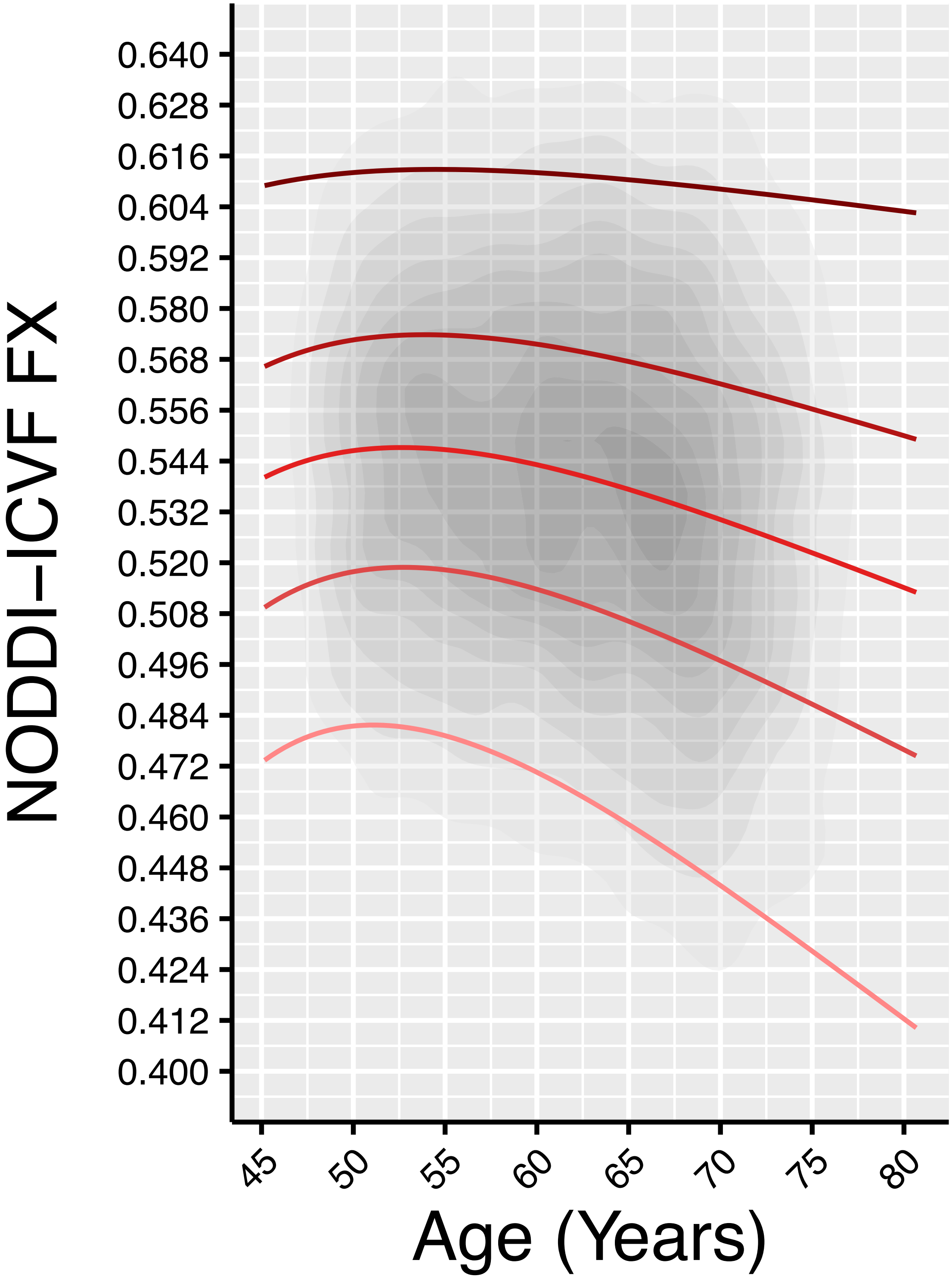

Figure S191. Full size normative centile reference curves calculated for the fornix (body) tract for NODDI-ICVF in females. Solid colored lines, ordered from lightest to darkest, indicate the following centiles: 5th, 25th, 50th, 75th, 95th. Gray overlay reflects kernel density (darker=greater degree of data point overlap). FX = fornix (body).

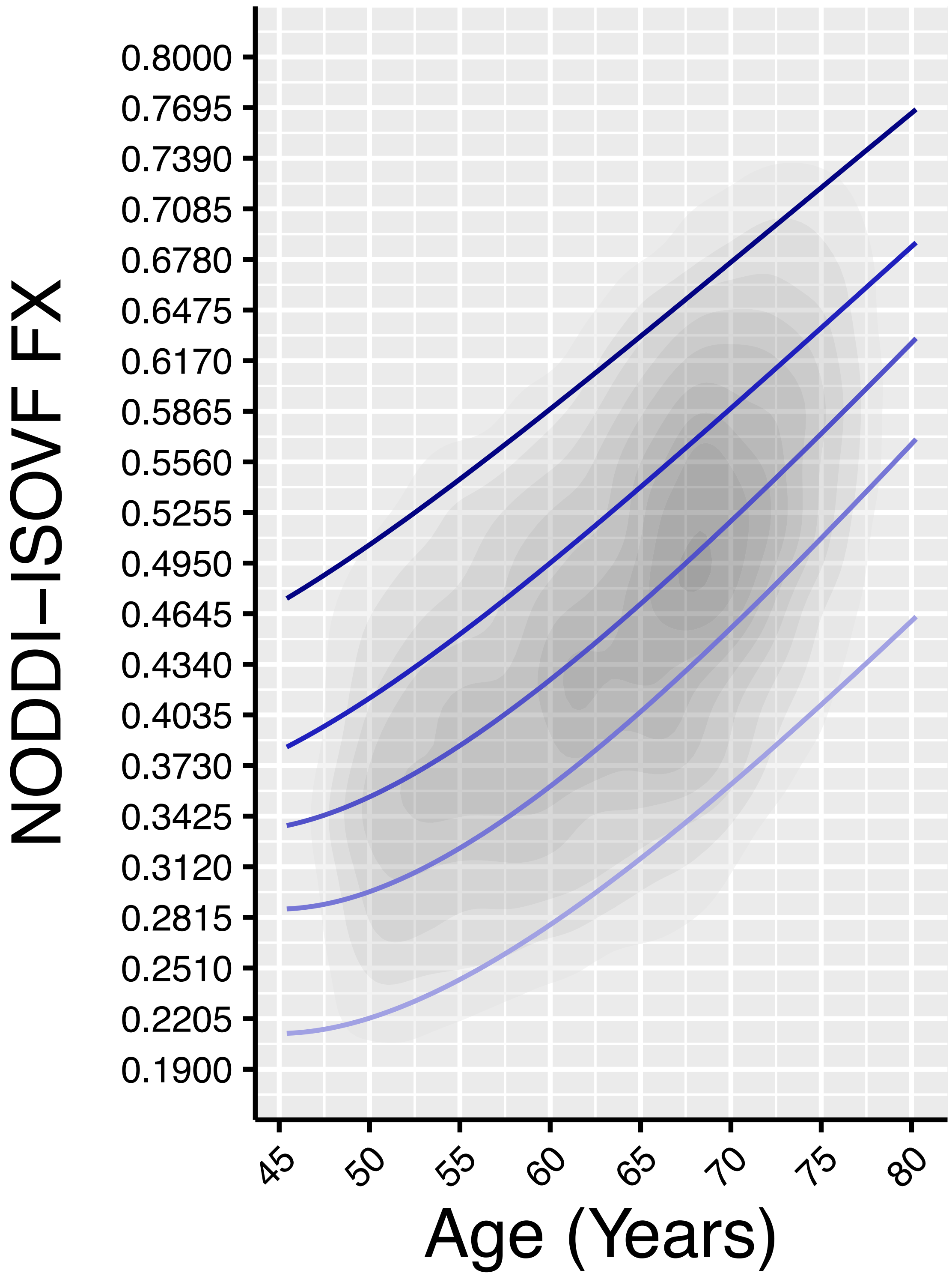

Figure S192. Full size normative centile reference curves calculated for the fornix (body) tract for NODDI-ISOVF in males. Solid colored lines, ordered from lightest to darkest, indicate the following centiles: 5th, 25th, 50th, 75th, 95th. Gray overlay reflects kernel density (darker=greater degree of data point overlap). FX = fornix (body).

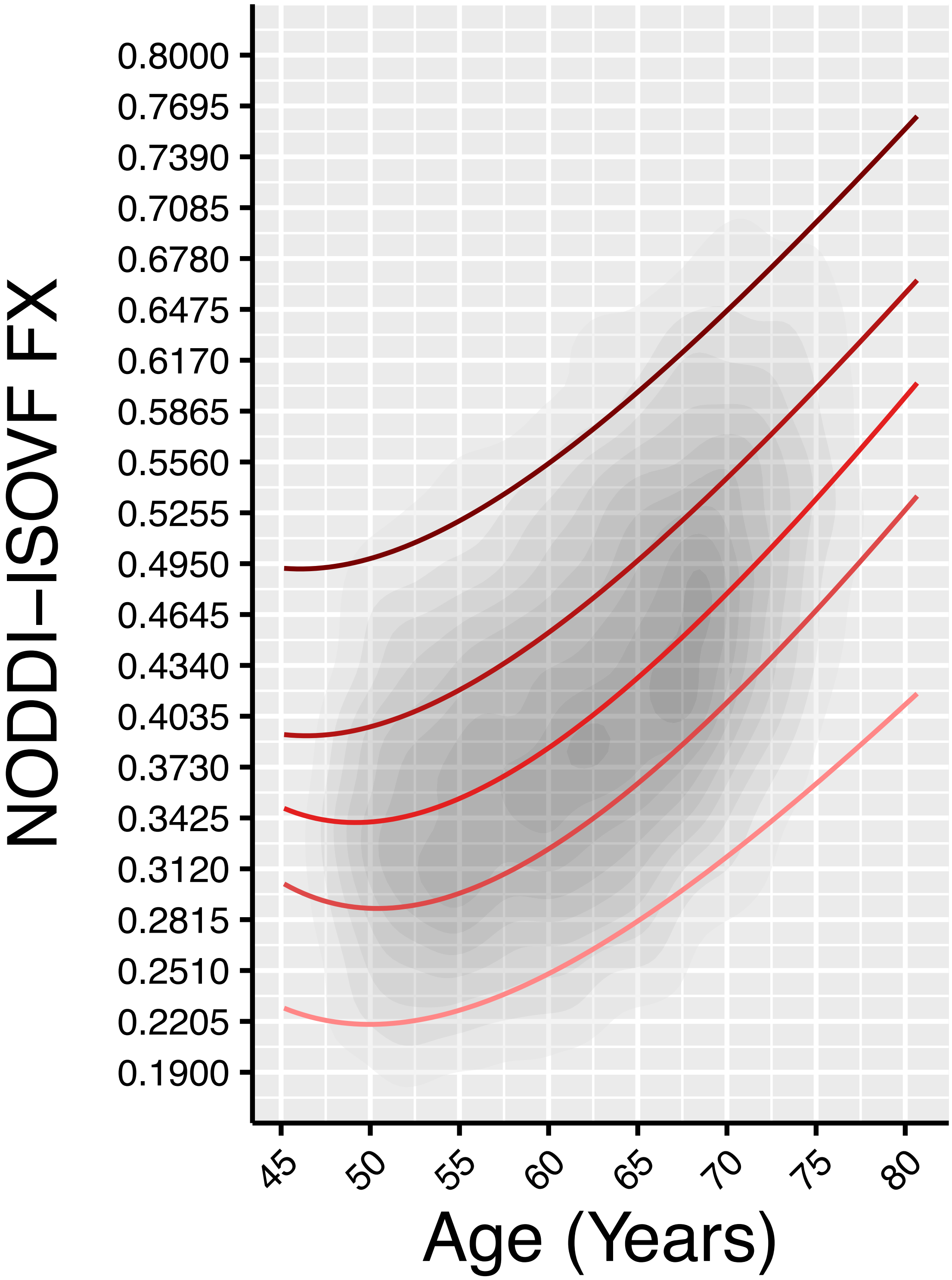

Figure S193. Full size normative centile reference curves calculated for the fornix (body) tract for NODDI-ISOVF in females. Solid colored lines, ordered from lightest to darkest, indicate the following centiles: 5th, 25th, 50th, 75th, 95th. Gray overlay reflects kernel density (darker=greater degree of data point overlap). FX = fornix (body).

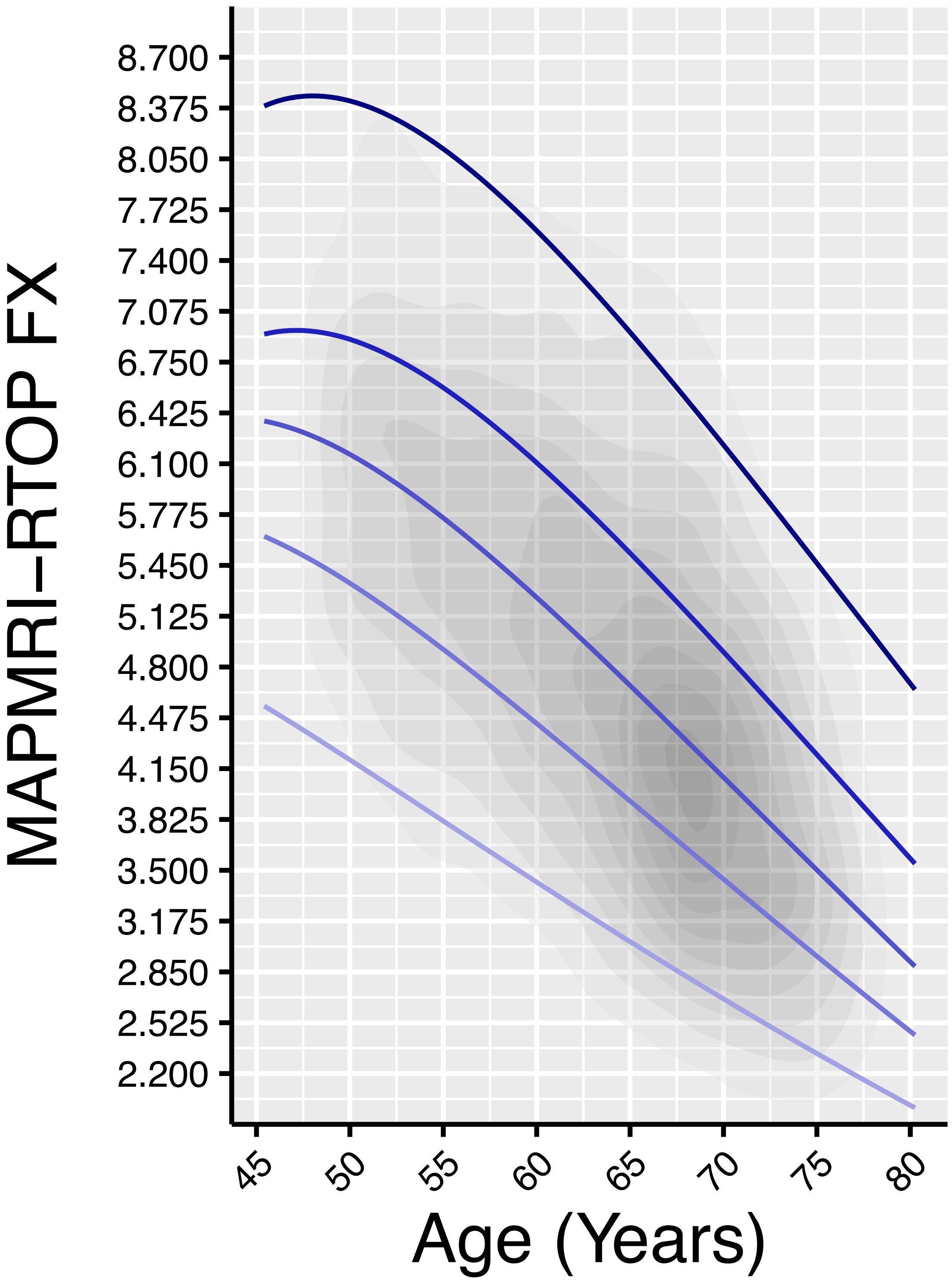

Figure S194. Full size normative centile reference curves calculated for the fornix (body) tract for MAPMRI-RTOP in males. Solid colored lines, ordered from lightest to darkest, indicate the following centiles: 5th, 25th, 50th, 75th, 95th. Gray overlay reflects kernel density (darker=greater degree of data point overlap). FX = fornix (body).

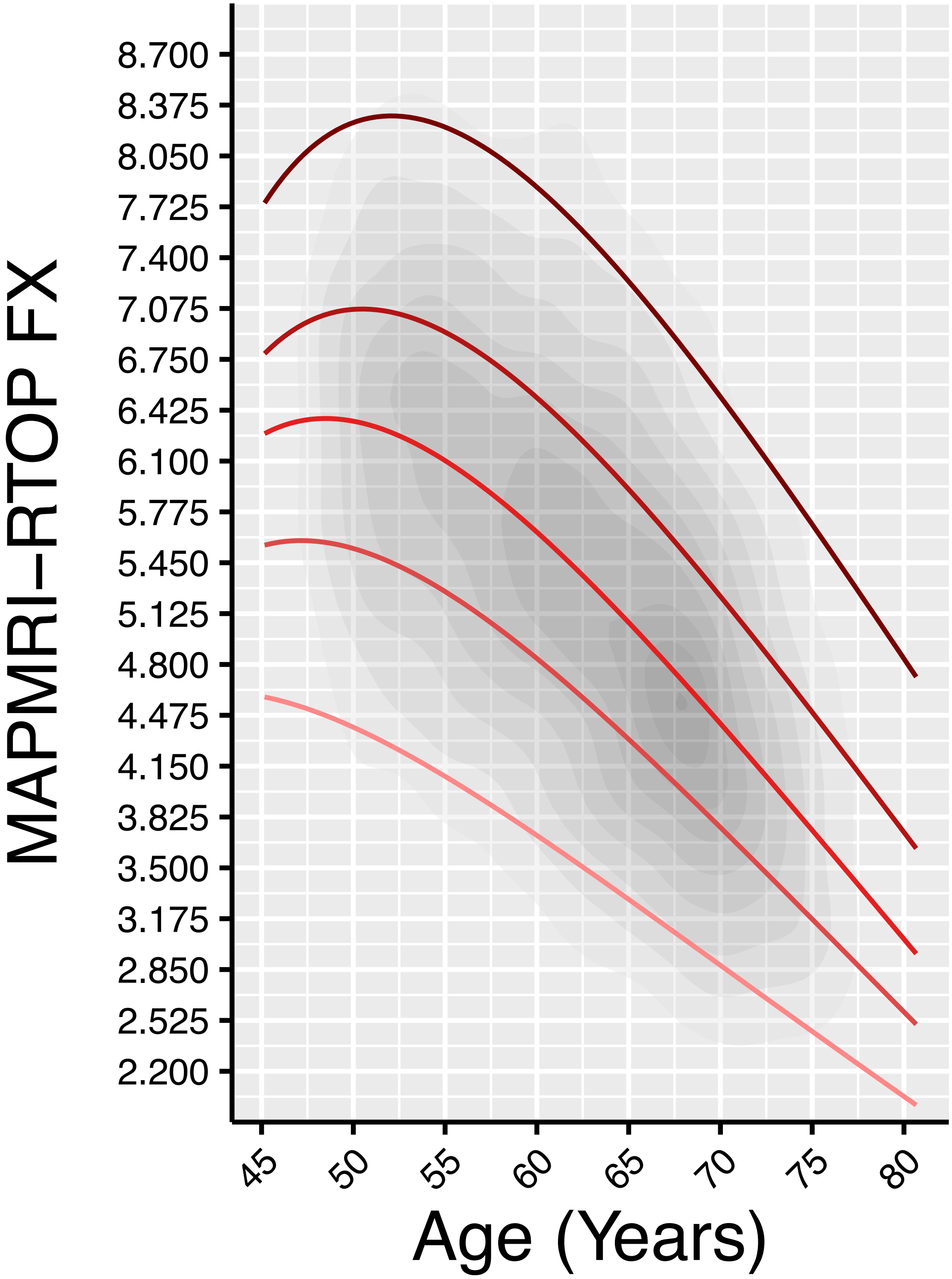

Figure S195. Full size normative centile reference curves calculated for the fornix (body) tract for MAPMRI-RTOP in females. Solid colored lines, ordered from lightest to darkest, indicate the following centiles: 5th, 25th, 50th, 75th, 95th. Gray overlay reflects kernel density (darker=greater degree of data point overlap). FX = fornix (body).

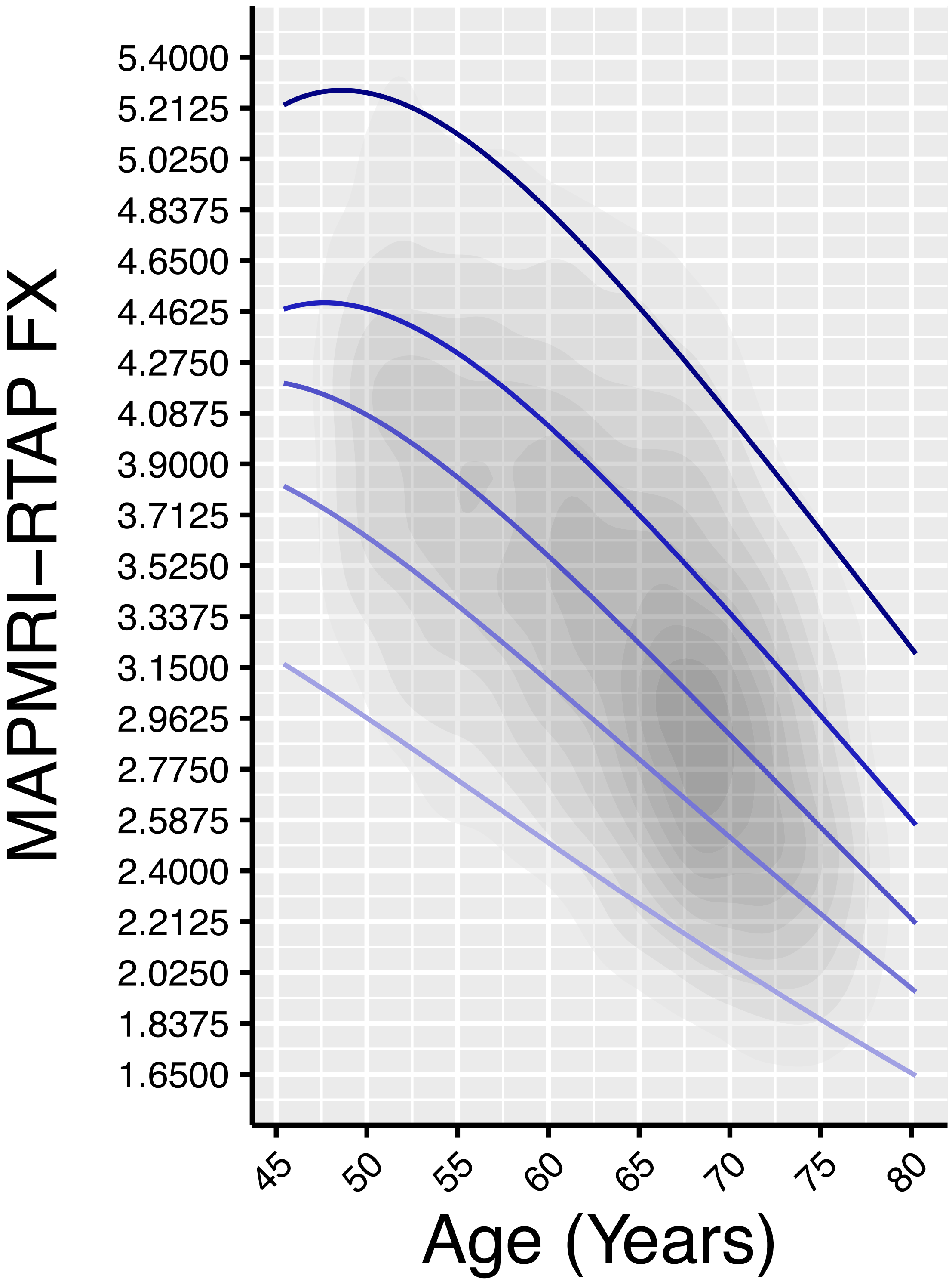

Figure S196. Full size normative centile reference curves calculated for the fornix (body) tract for MAPMRI-RTAP in males. Solid colored lines, ordered from lightest to darkest, indicate the following centiles: 5th, 25th, 50th, 75th, 95th. Gray overlay reflects kernel density (darker=greater degree of data point overlap). FX = fornix (body).

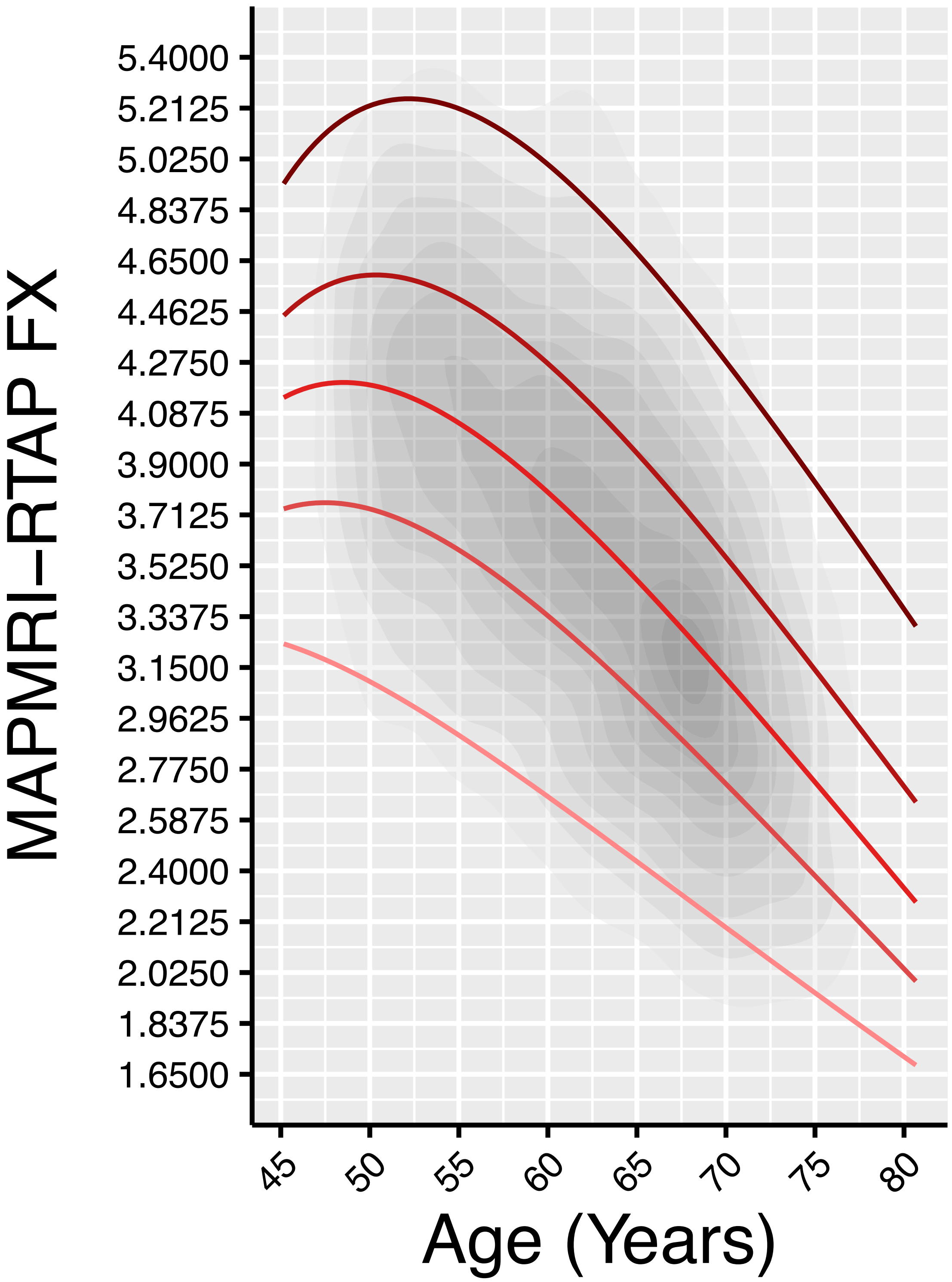

Figure S197. Full size normative centile reference curves calculated for the fornix (body) tract for MAPMRI-RTAP in females. Solid colored lines, ordered from lightest to darkest, indicate the following centiles: 5th, 25th, 50th, 75th, 95th. Gray overlay reflects kernel density (darker=greater degree of data point overlap). FX = fornix (body).

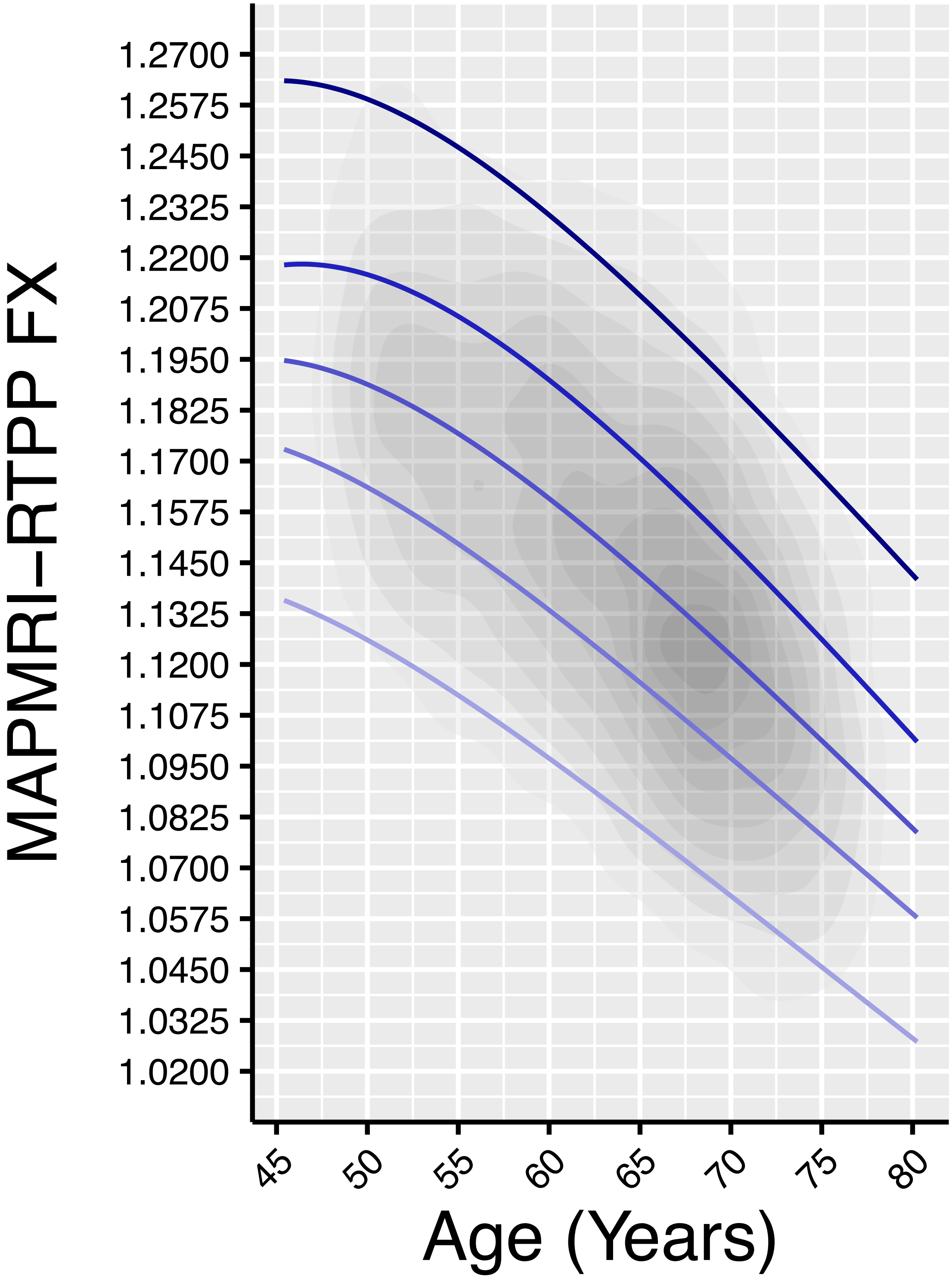

Figure S198. Full size normative centile reference curves calculated for the fornix (body) tract for MAPMRI-RTPP in males. Solid colored lines, ordered from lightest to darkest, indicate the following centiles: 5th, 25th, 50th, 75th, 95th. Gray overlay reflects kernel density (darker=greater degree of data point overlap). FX = fornix (body).

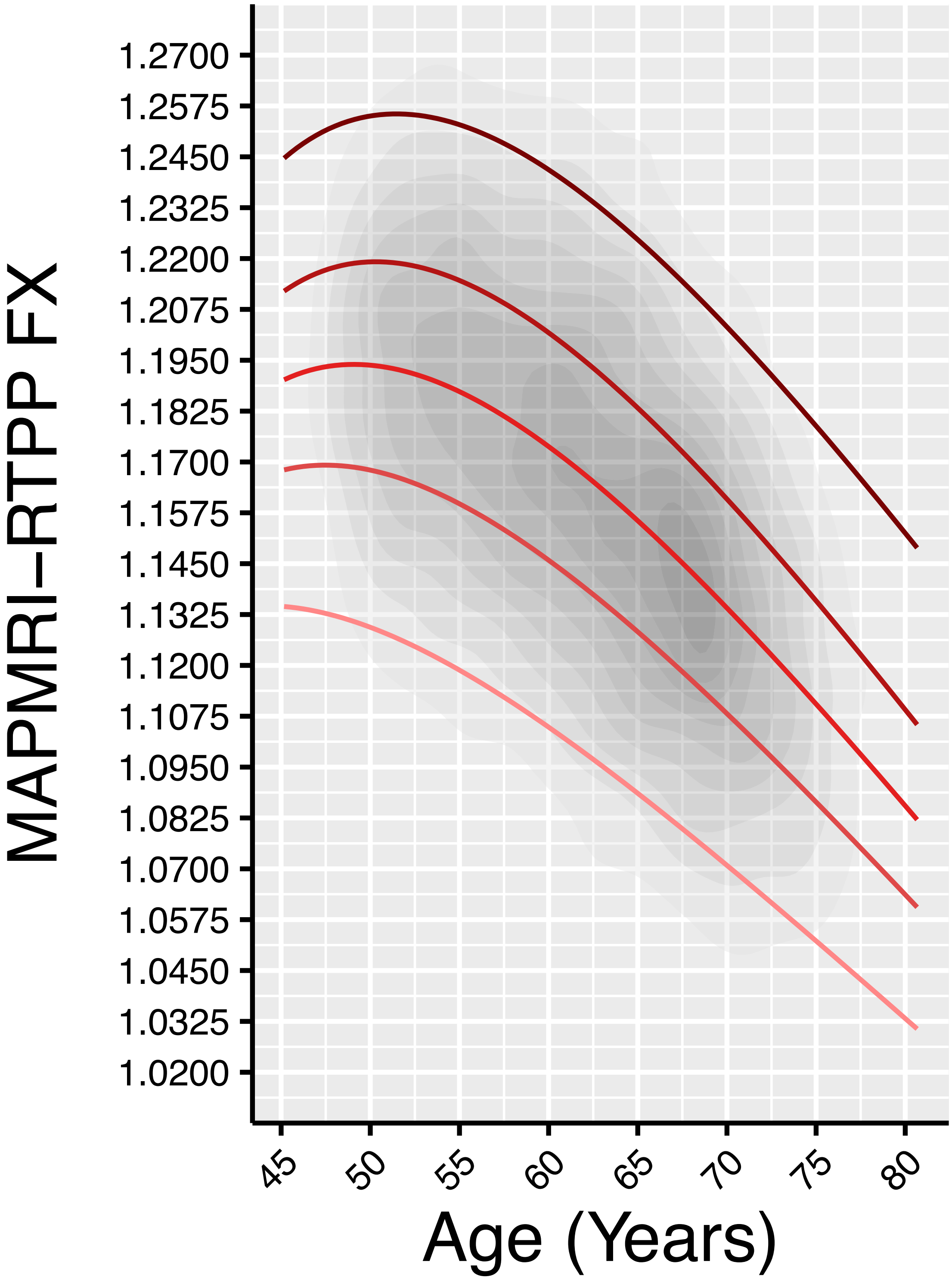

Figure S199. Full size normative centile reference curves calculated for the fornix (body) tract for MAPMRI-RTPP in females. Solid colored lines, ordered from lightest to darkest, indicate the following centiles: 5th, 25th, 50th, 75th, 95th. Gray overlay reflects kernel density (darker=greater degree of data point overlap). FX = fornix (body).

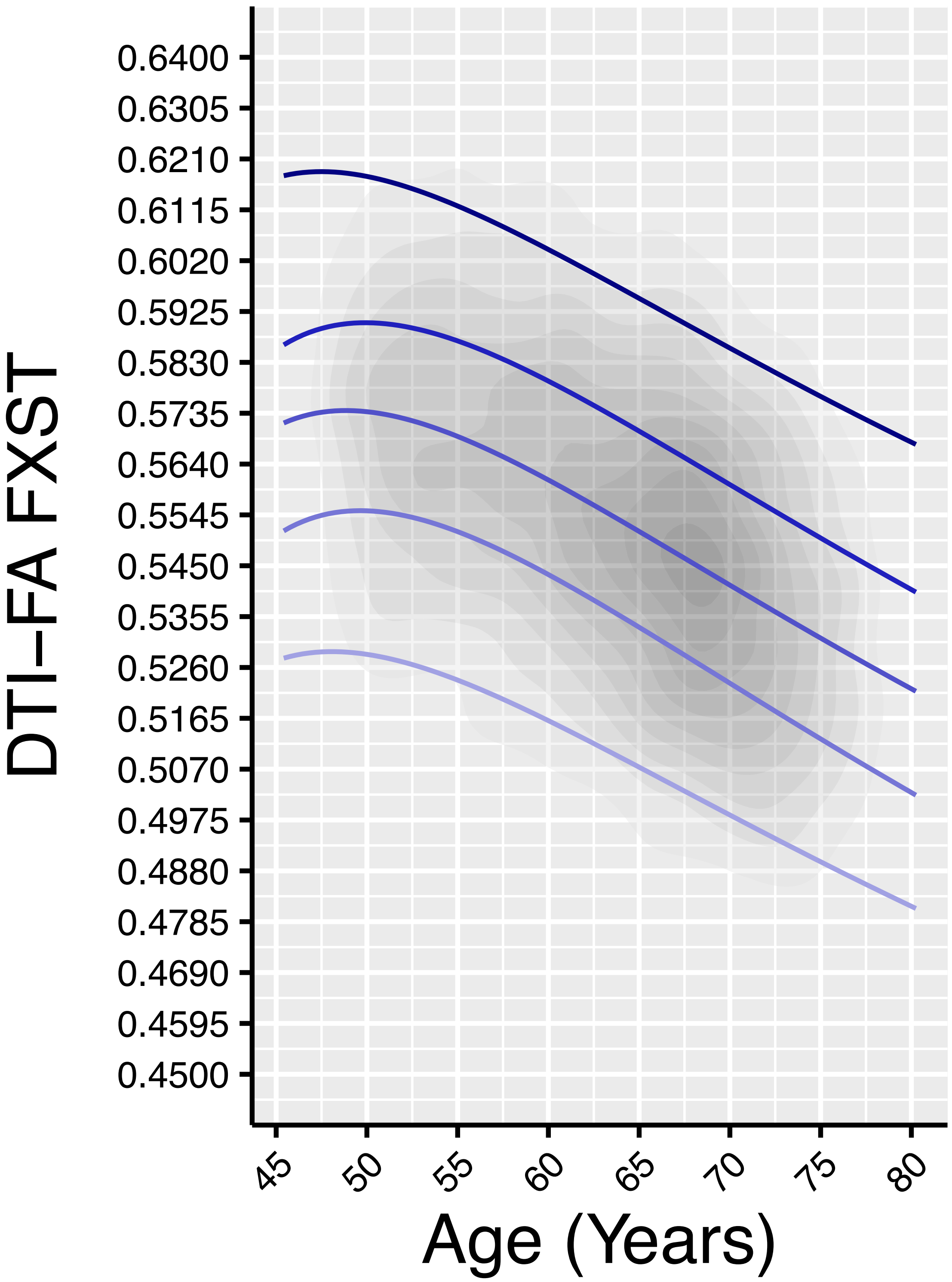

Figure S200. Full size normative centile reference curves calculated for the fornix (*crus*) / *stria terminalis* tract for DTI-FA in males. Solid colored lines, ordered from lightest to darkest, indicate the following centiles: 5th, 25th, 50th, 75th, 95th. Gray overlay reflects kernel density (darker=greater degree of data point overlap). FXST = fornix (*crus*) / *stria terminalis.*

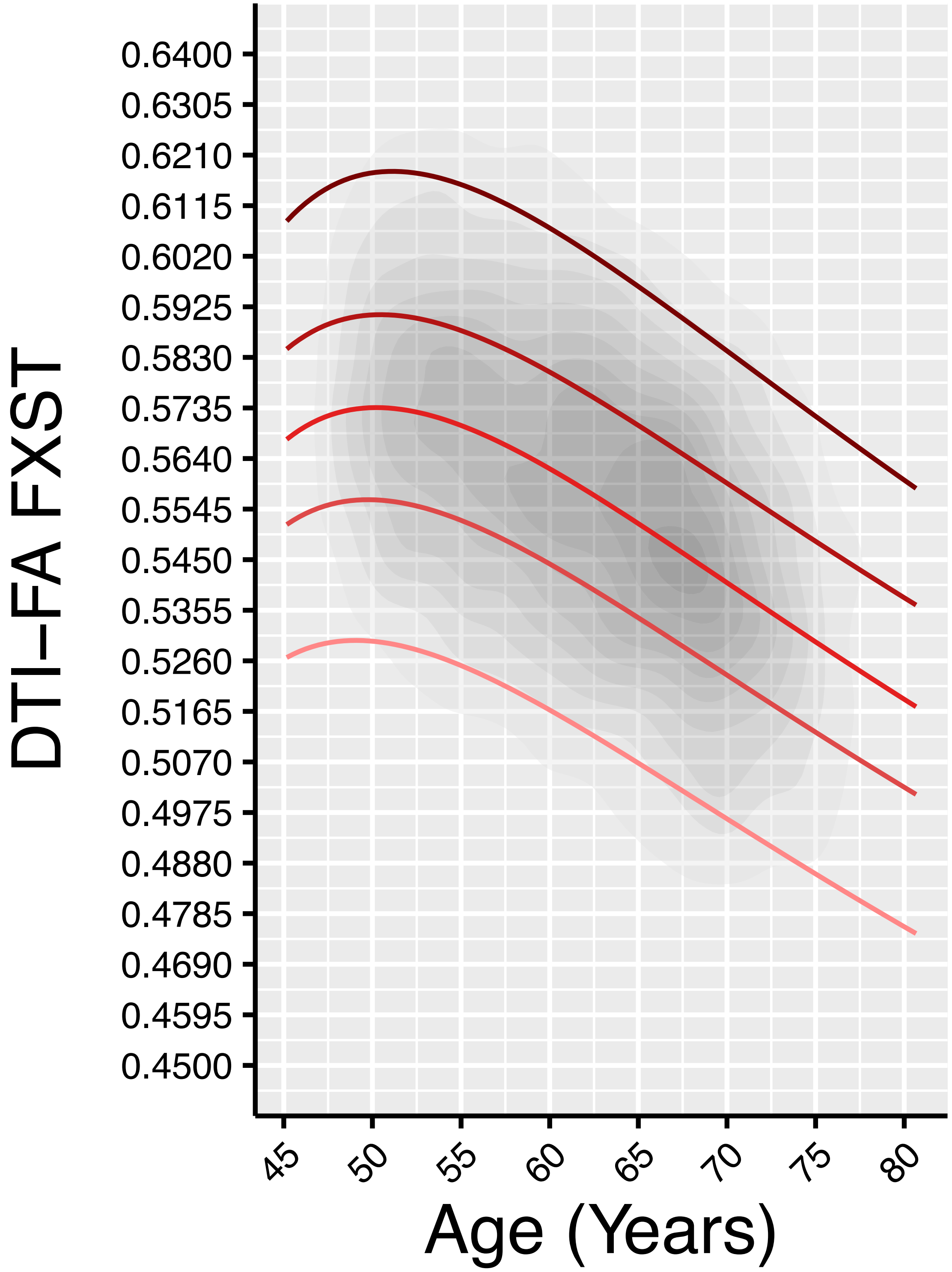

Figure S201. Full size normative centile reference curves calculated for the fornix (*crus*) / *stria terminalis* tract for DTI-FA in females. Solid colored lines, ordered from lightest to darkest, indicate the following centiles: 5th, 25th, 50th, 75th, 95th. Gray overlay reflects kernel density (darker=greater degree of data point overlap). FXST = fornix (*crus*) / *stria terminalis.*

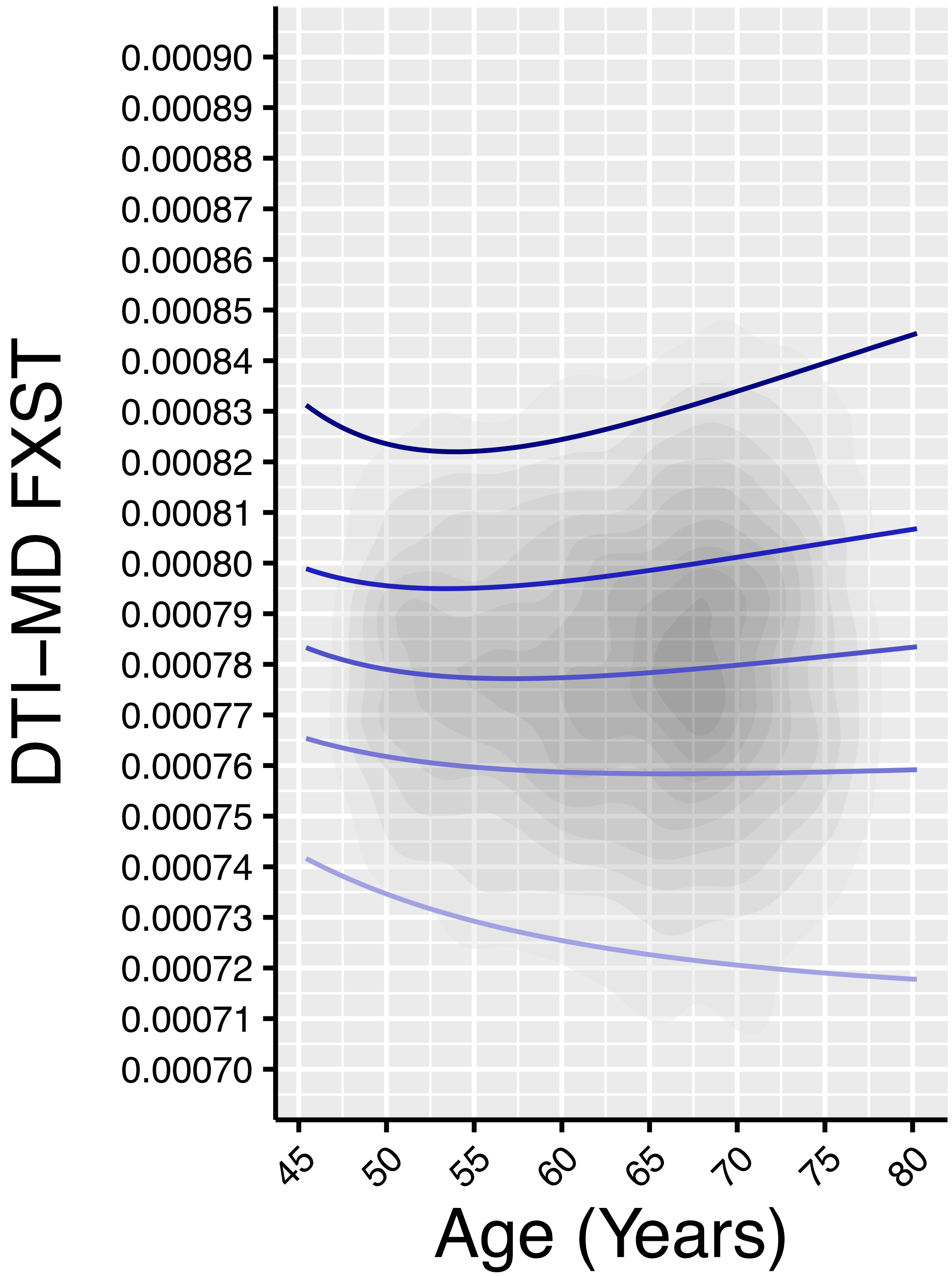

Figure S202. Full size normative centile reference curves calculated for the fornix (*crus*) / *stria terminalis* tract for DTI-MD in males. Solid colored lines, ordered from lightest to darkest, indicate the following centiles: 5th, 25th, 50th, 75th, 95th. Gray overlay reflects kernel density (darker=greater degree of data point overlap). FXST = fornix (*crus*) / *stria terminalis.*

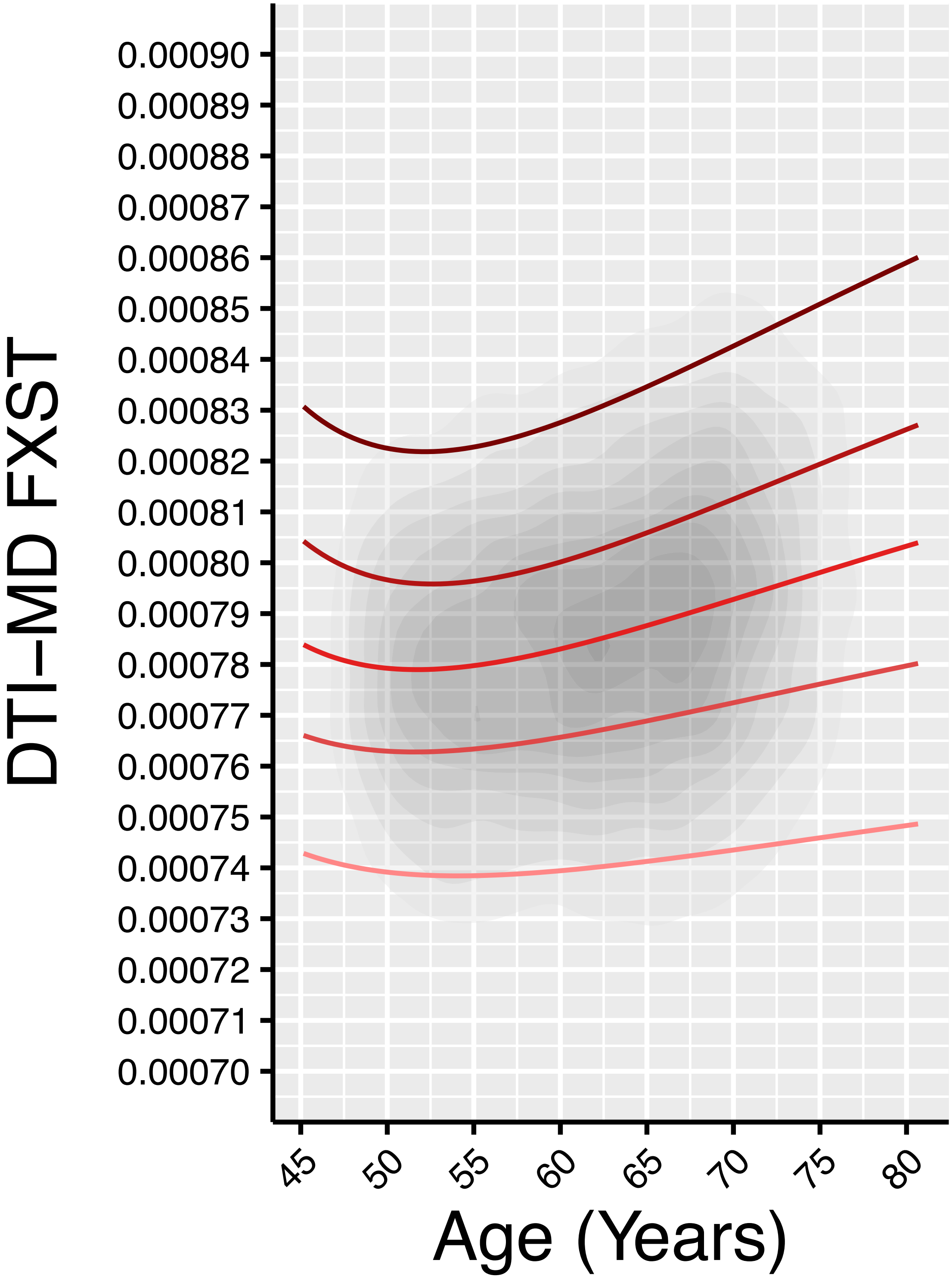

Figure S203. Full size normative centile reference curves calculated for the fornix (*crus*) / *stria terminalis* tract for DTI-MD in females. Solid colored lines, ordered from lightest to darkest, indicate the following centiles: 5th, 25th, 50th, 75th, 95th. Gray overlay reflects kernel density (darker=greater degree of data point overlap). FXST = fornix (*crus*) / *stria terminalis.*

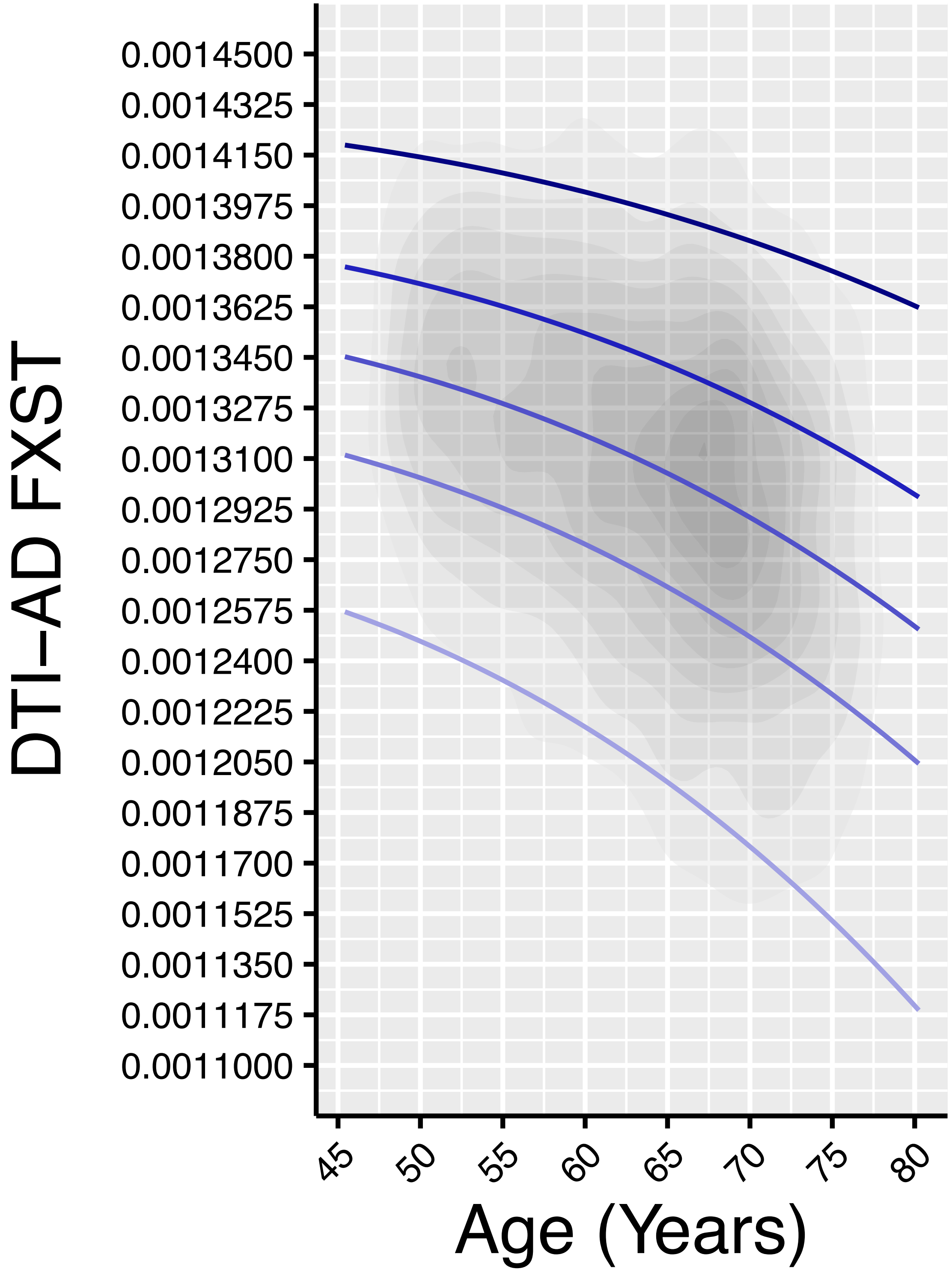

Figure S204. Full size normative centile reference curves calculated for the fornix (*crus*) / *stria terminalis* tract for DTI-AD in males. Solid colored lines, ordered from lightest to darkest, indicate the following centiles: 5th, 25th, 50th, 75th, 95th. Gray overlay reflects kernel density (darker=greater degree of data point overlap). FXST = fornix (*crus*) / *stria terminalis.*

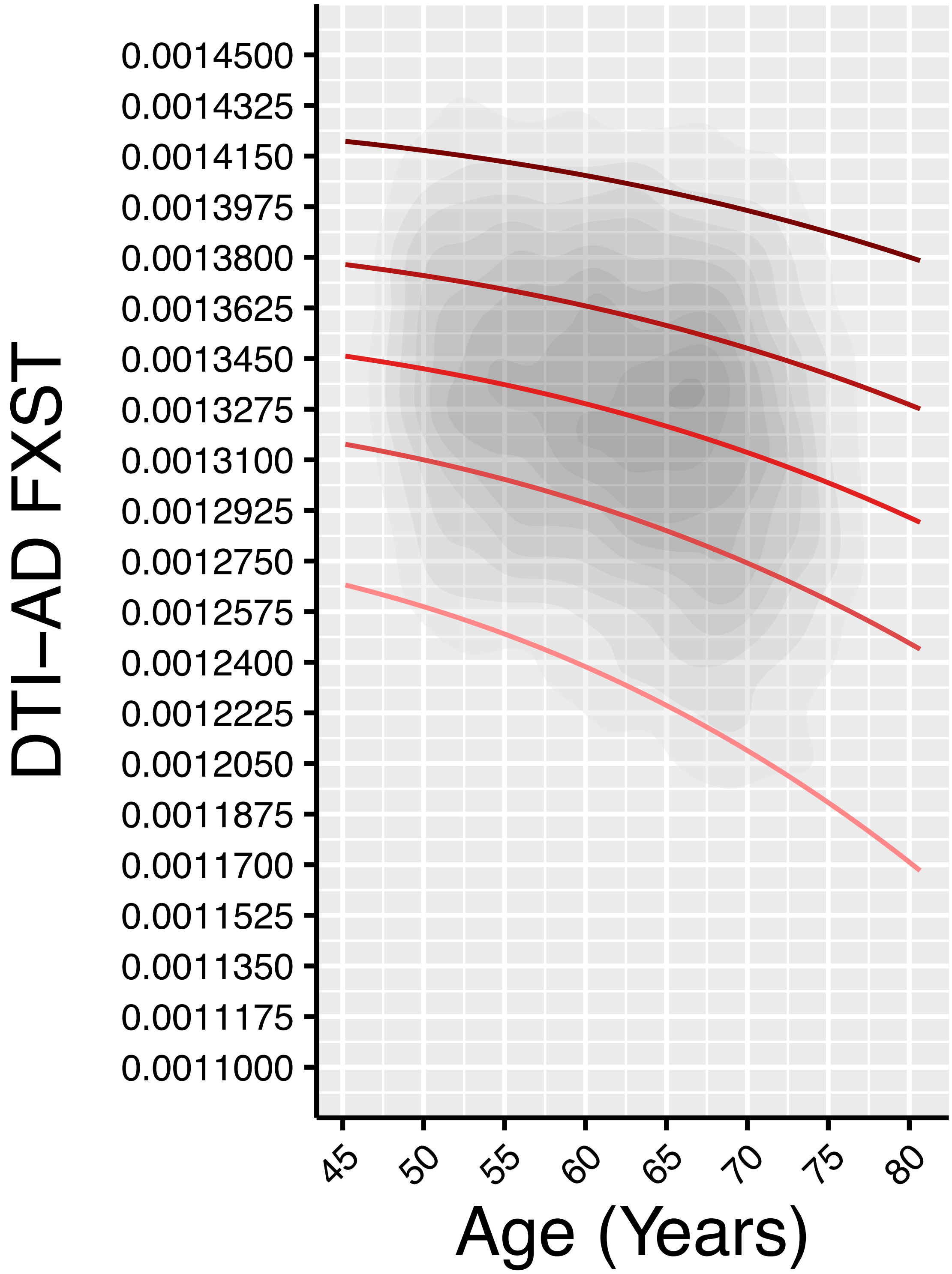

Figure S205. Full size normative centile reference curves calculated for the fornix (*crus*) / *stria terminalis* tract for DTI-AD in females. Solid colored lines, ordered from lightest to darkest, indicate the following centiles: 5th, 25th, 50th, 75th, 95th. Gray overlay reflects kernel density (darker=greater degree of data point overlap). FXST = fornix (*crus*) / *stria terminalis.*

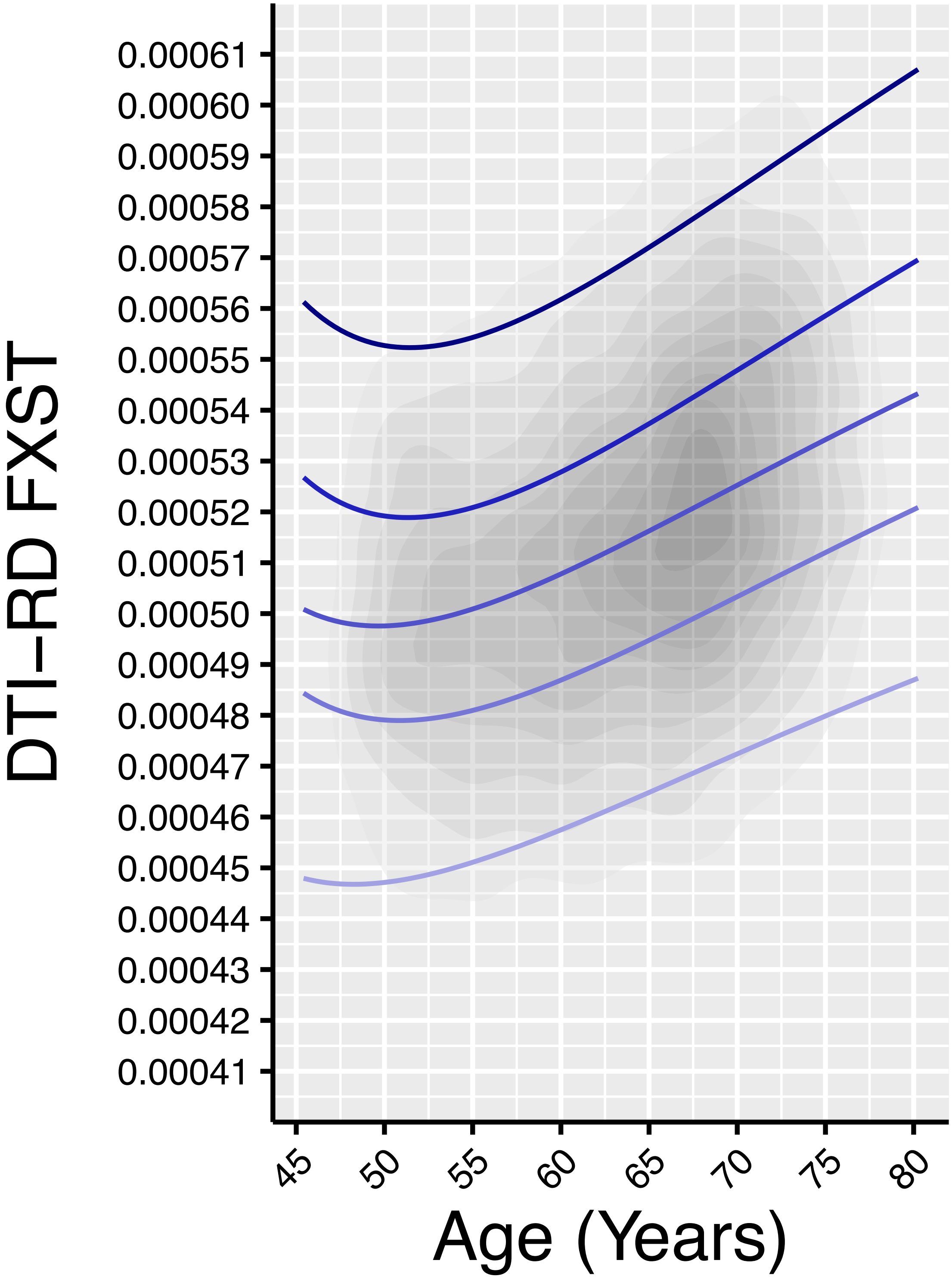

Figure S206. Full size normative centile reference curves calculated for the fornix (*crus*) / *stria terminalis* tract for DTI-RD in males. Solid colored lines, ordered from lightest to darkest, indicate the following centiles: 5th, 25th, 50th, 75th, 95th. Gray overlay reflects kernel density (darker=greater degree of data point overlap). FXST = fornix (*crus*) / *stria terminalis.*

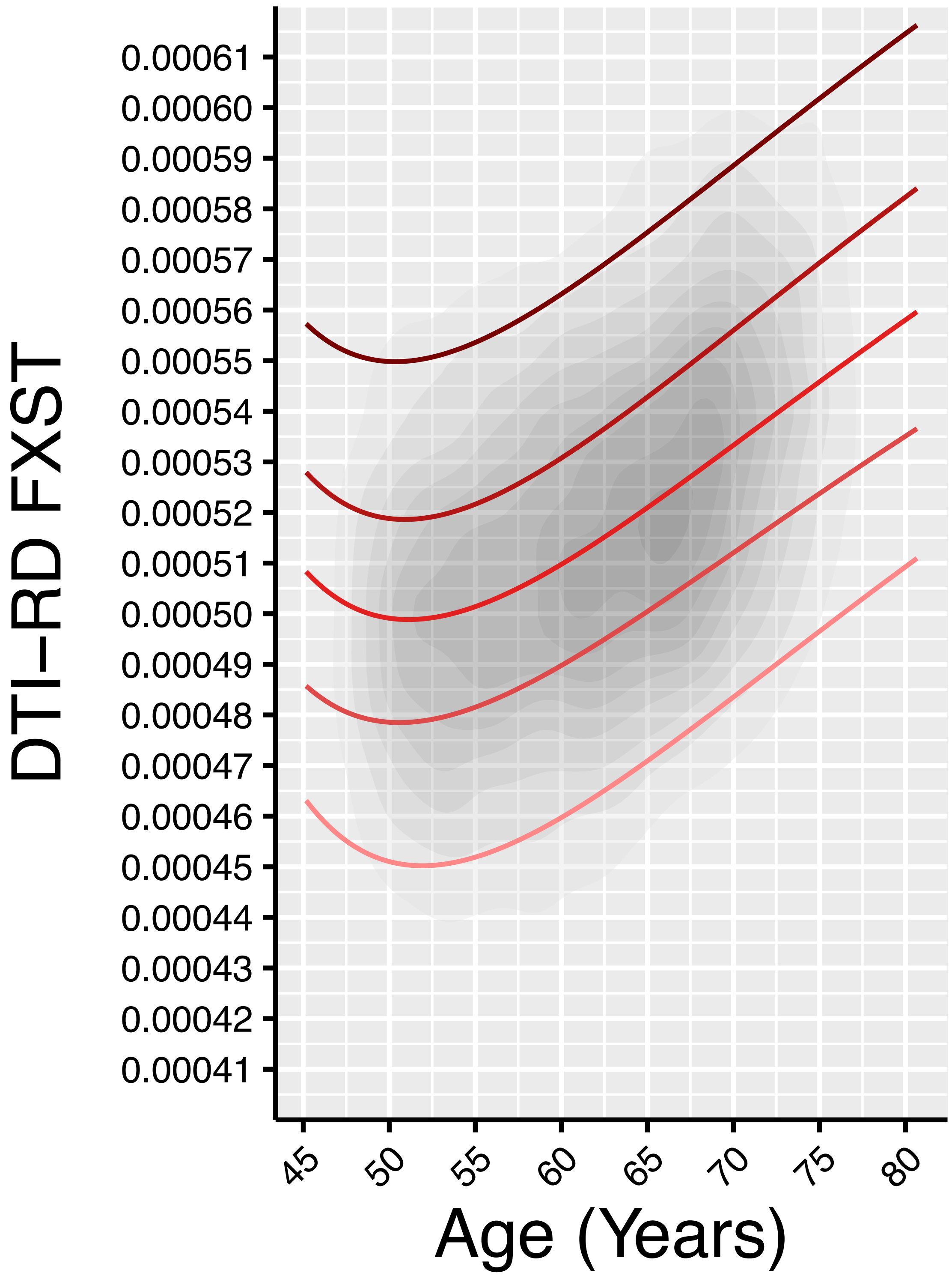

Figure S207. Full size normative centile reference curves calculated for the fornix (*crus*) / *stria terminalis* tract for DTI-RD in females. Solid colored lines, ordered from lightest to darkest, indicate the following centiles: 5th, 25th, 50th, 75th, 95th. Gray overlay reflects kernel density (darker=greater degree of data point overlap). FXST = fornix (*crus*) / *stria terminalis.*

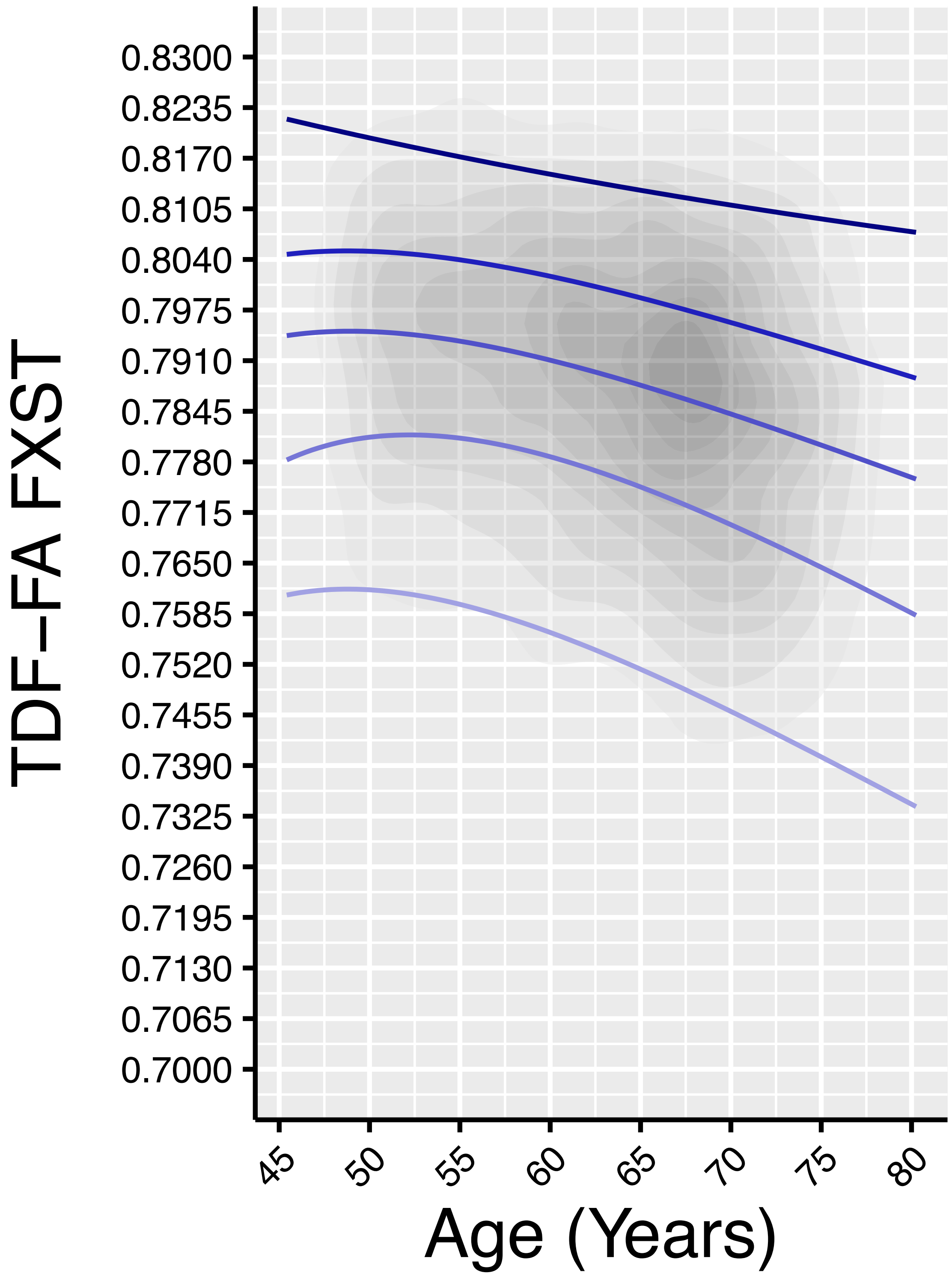

Figure S208. Full size normative centile reference curves calculated for the fornix (*crus*) / *stria terminalis* tract for TDF-FA in males. Solid colored lines, ordered from lightest to darkest, indicate the following centiles: 5th, 25th, 50th, 75th, 95th. Gray overlay reflects kernel density (darker=greater degree of data point overlap). FXST = fornix (*crus*) / *stria terminalis.*

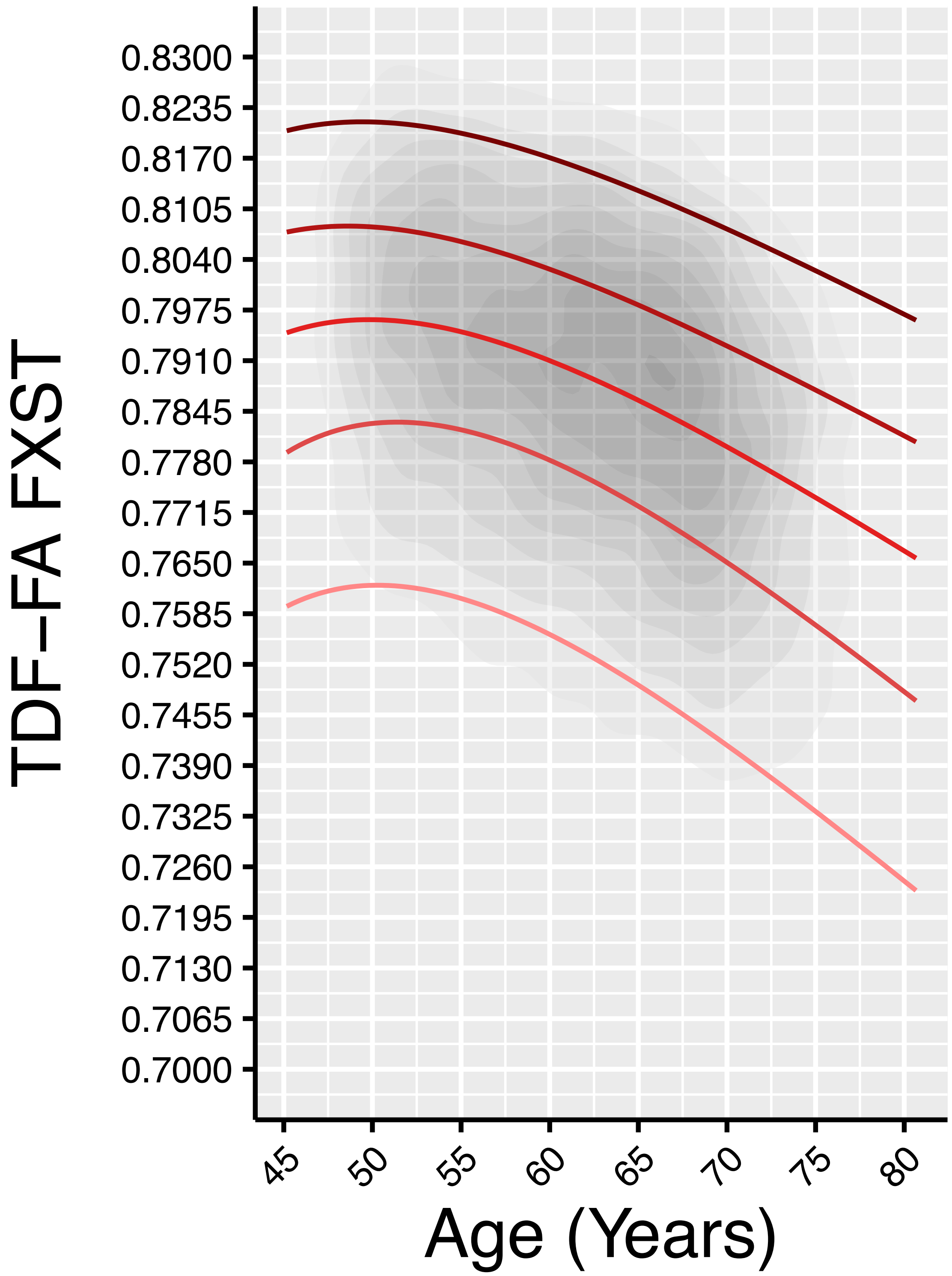

Figure S209. Full size normative centile reference curves calculated for the fornix (*crus*) / *stria terminalis* tract for TDF-FA in females. Solid colored lines, ordered from lightest to darkest, indicate the following centiles: 5th, 25th, 50th, 75th, 95th. Gray overlay reflects kernel density (darker=greater degree of data point overlap). FXST = fornix (*crus*) / *stria terminalis.*

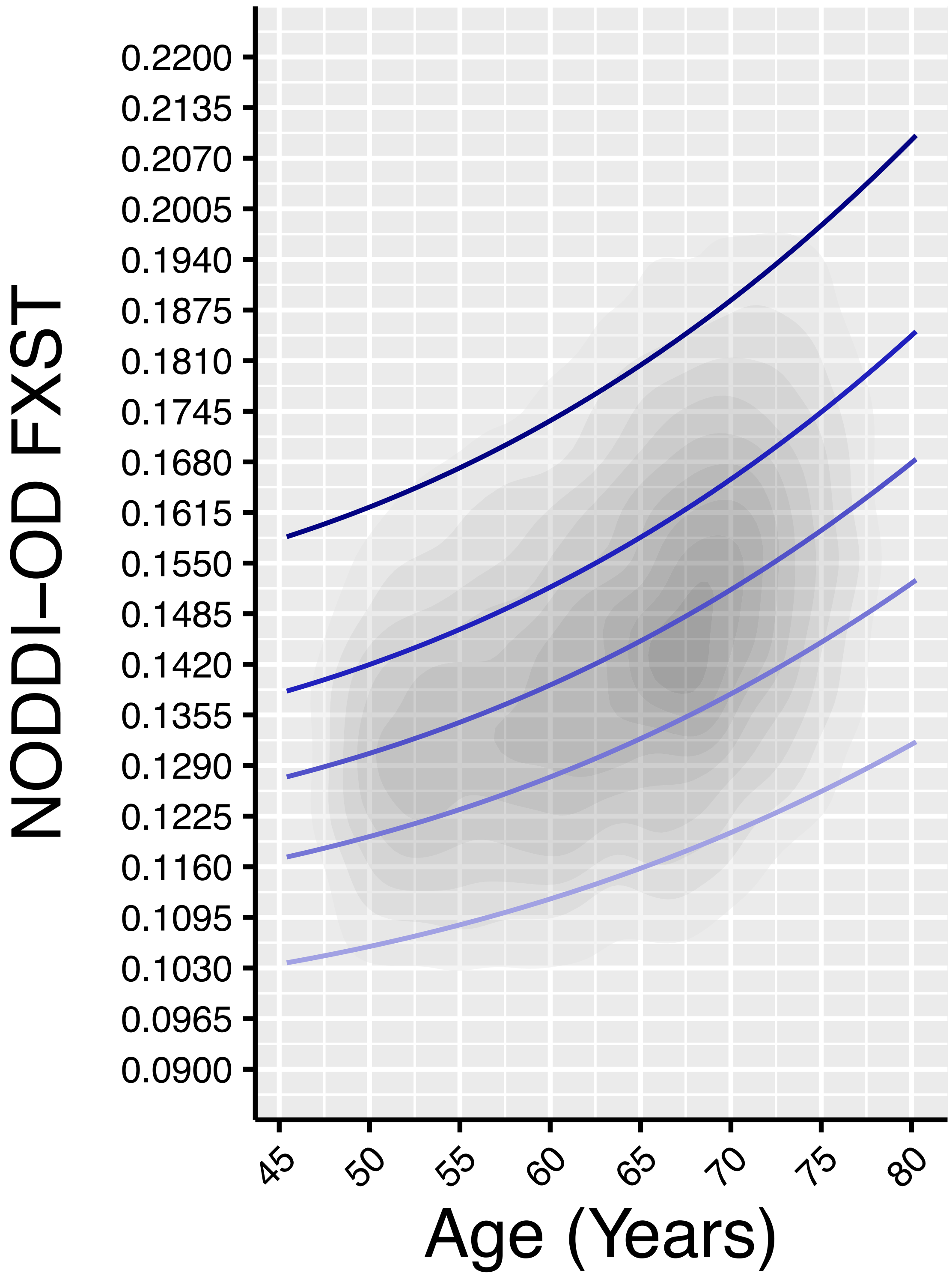

Figure S210. Full size normative centile reference curves calculated for the fornix (*crus*) / *stria terminalis* tract for NODDI-OD in males. Solid colored lines, ordered from lightest to darkest, indicate the following centiles: 5th, 25th, 50th, 75th, 95th. Gray overlay reflects kernel density (darker=greater degree of data point overlap). FXST = fornix (*crus*) / *stria terminalis.*

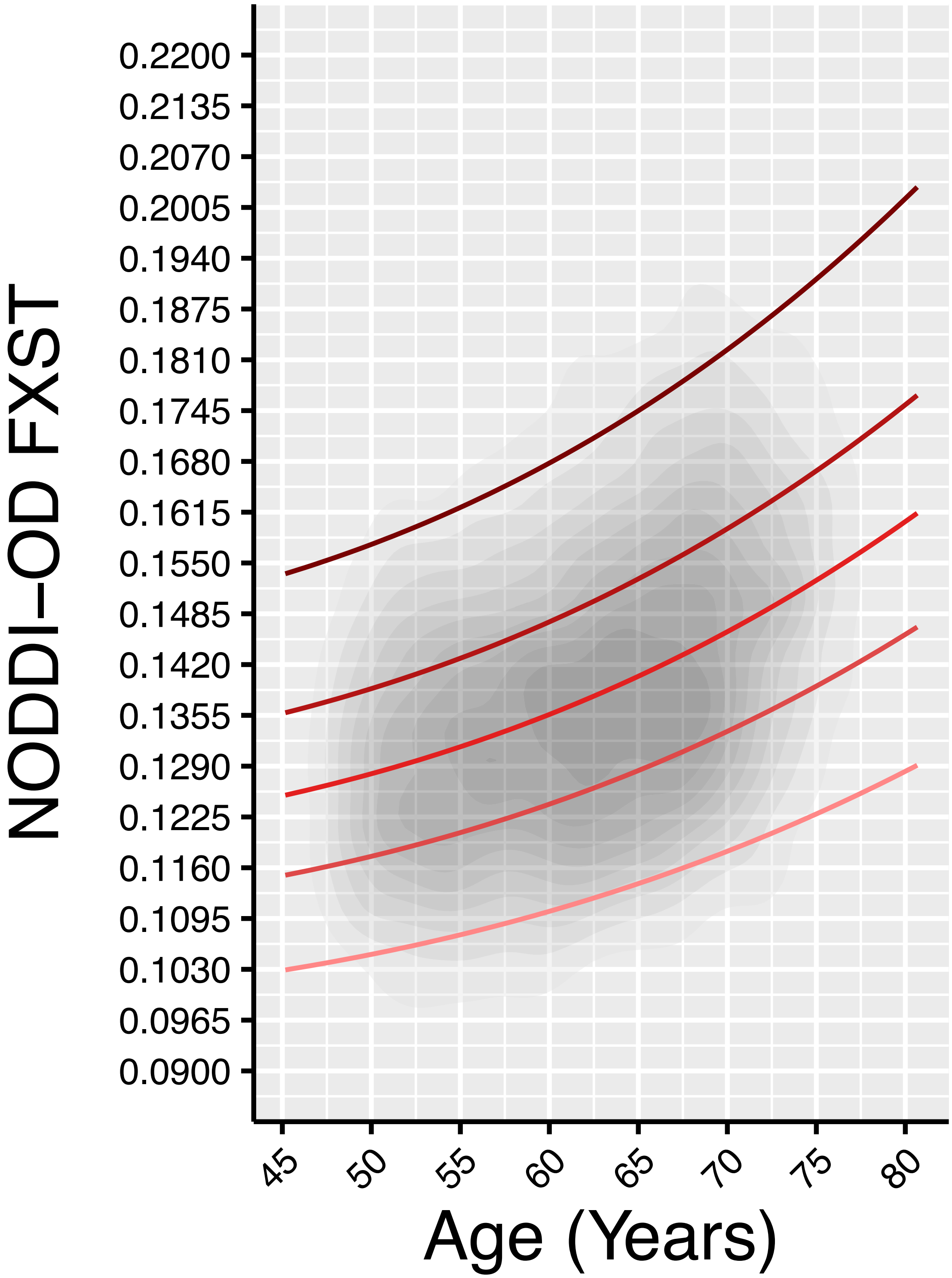

Figure S211. Full size normative centile reference curves calculated for the fornix (*crus*) / *stria terminalis* tract for NODDI-OD in females. Solid colored lines, ordered from lightest to darkest, indicate the following centiles: 5th, 25th, 50th, 75th, 95th. Gray overlay reflects kernel density (darker=greater degree of data point overlap). FXST = fornix (*crus*) / *stria terminalis.*

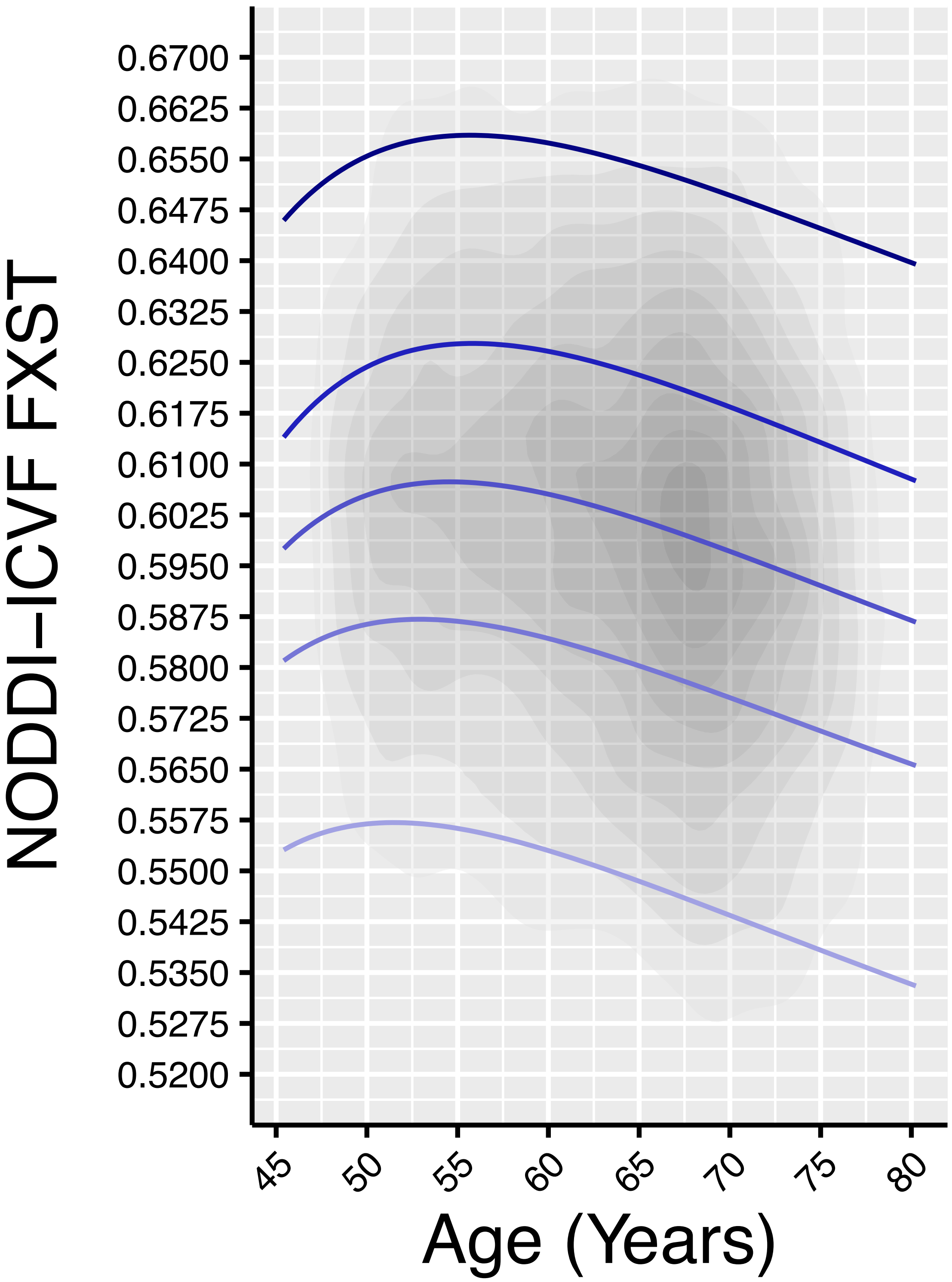

Figure S212. Full size normative centile reference curves calculated for the fornix (*crus*) / *stria terminalis* tract for NODDI-ICVF in males. Solid colored lines, ordered from lightest to darkest, indicate the following centiles: 5th, 25th, 50th, 75th, 95th. Gray overlay reflects kernel density (darker=greater degree of data point overlap). FXST = fornix (*crus*) / *stria terminalis.*

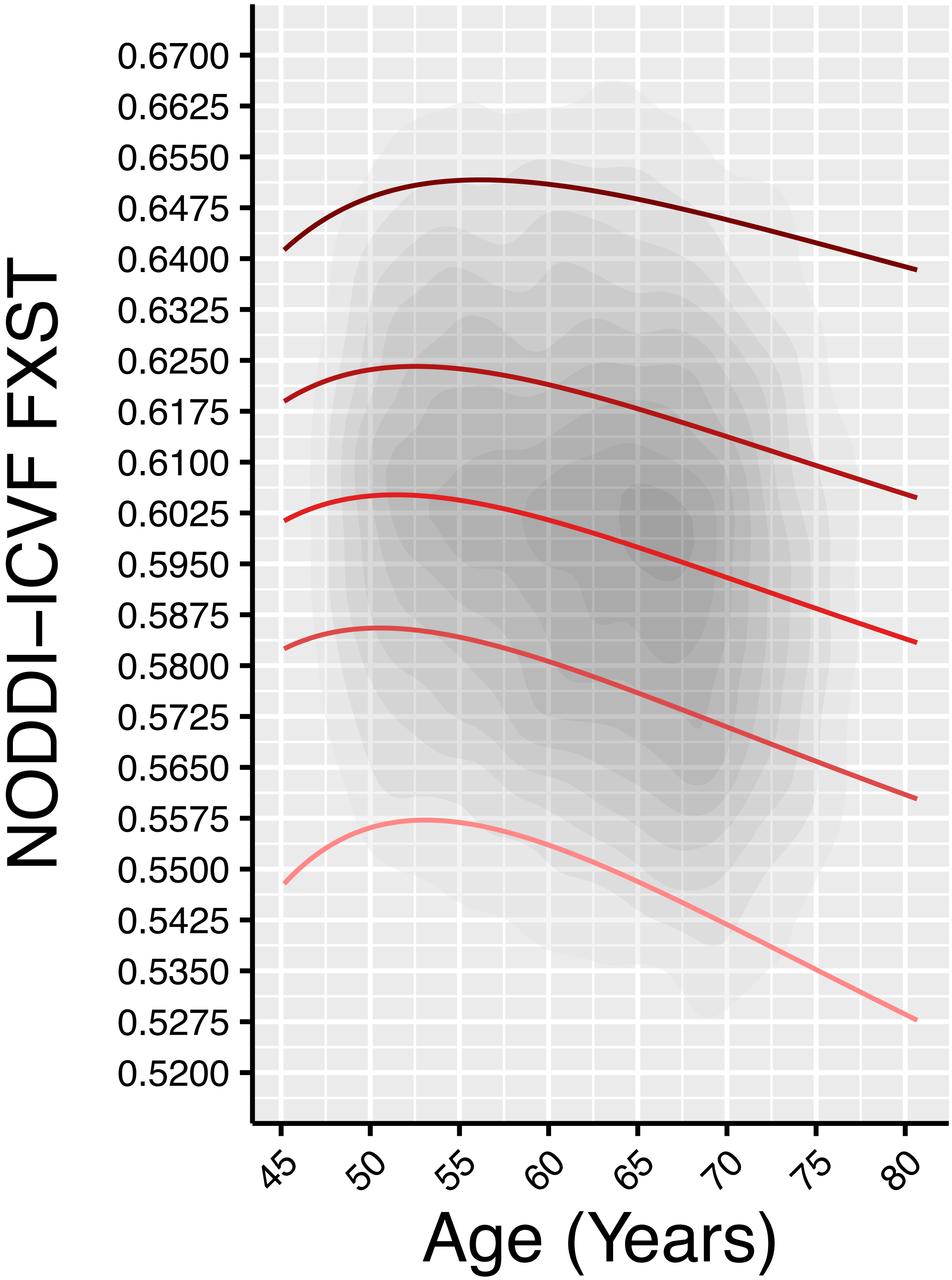

Figure S213. Full size normative centile reference curves calculated for the fornix (*crus*) / *stria terminalis* tract for NODDI-ICVF in females. Solid colored lines, ordered from lightest to darkest, indicate the following centiles: 5th, 25th, 50th, 75th, 95th. Gray overlay reflects kernel density (darker=greater degree of data point overlap). FXST = fornix (*crus*) / *stria terminalis.*

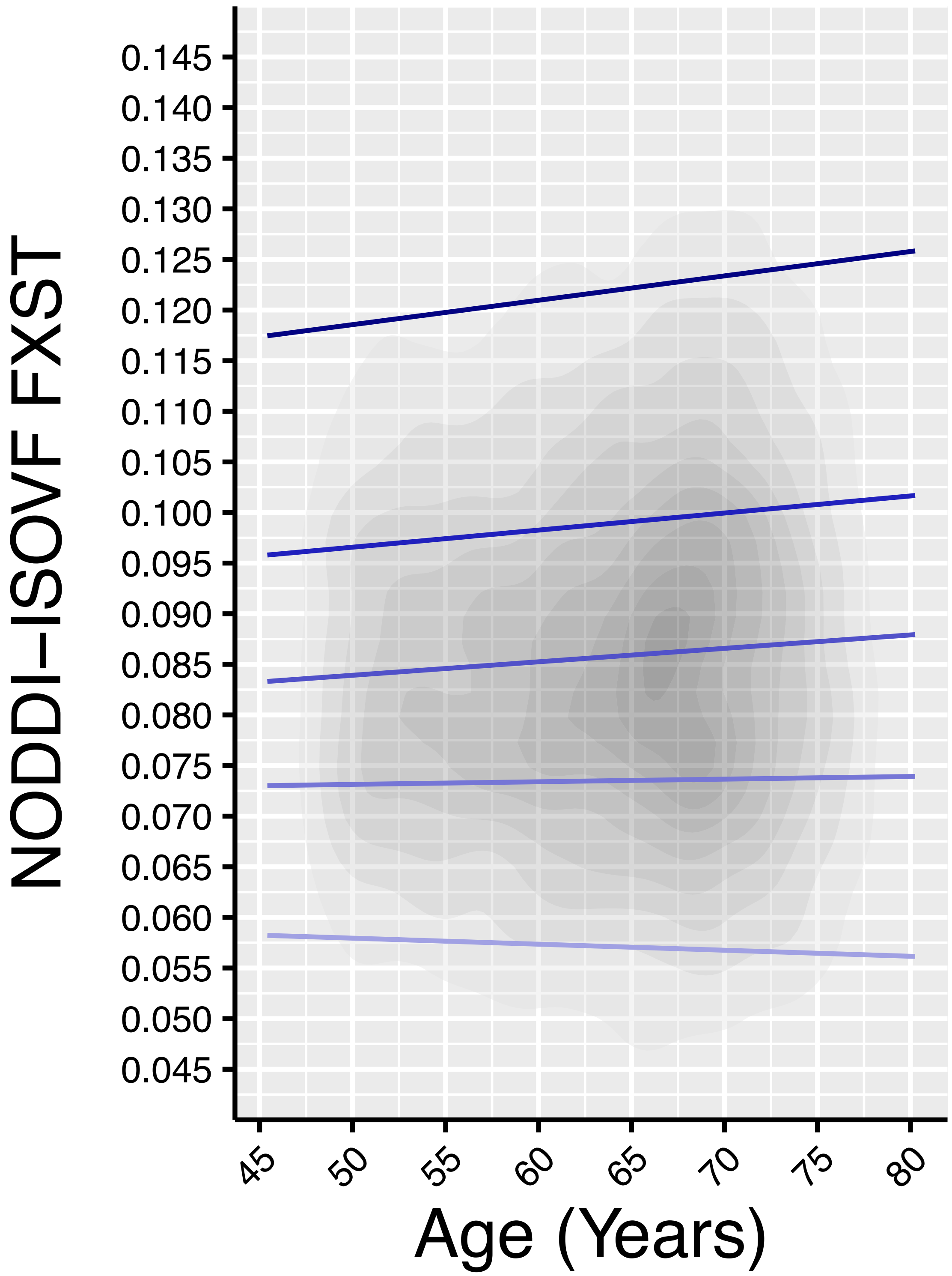

Figure S214. Full size normative centile reference curves calculated for the fornix (*crus*) / *stria terminalis* tract for NODDI-ISOVF in males. Solid colored lines, ordered from lightest to darkest, indicate the following centiles: 5th, 25th, 50th, 75th, 95th. Gray overlay reflects kernel density (darker=greater degree of data point overlap). FXST = fornix (*crus*) / *stria terminalis.*

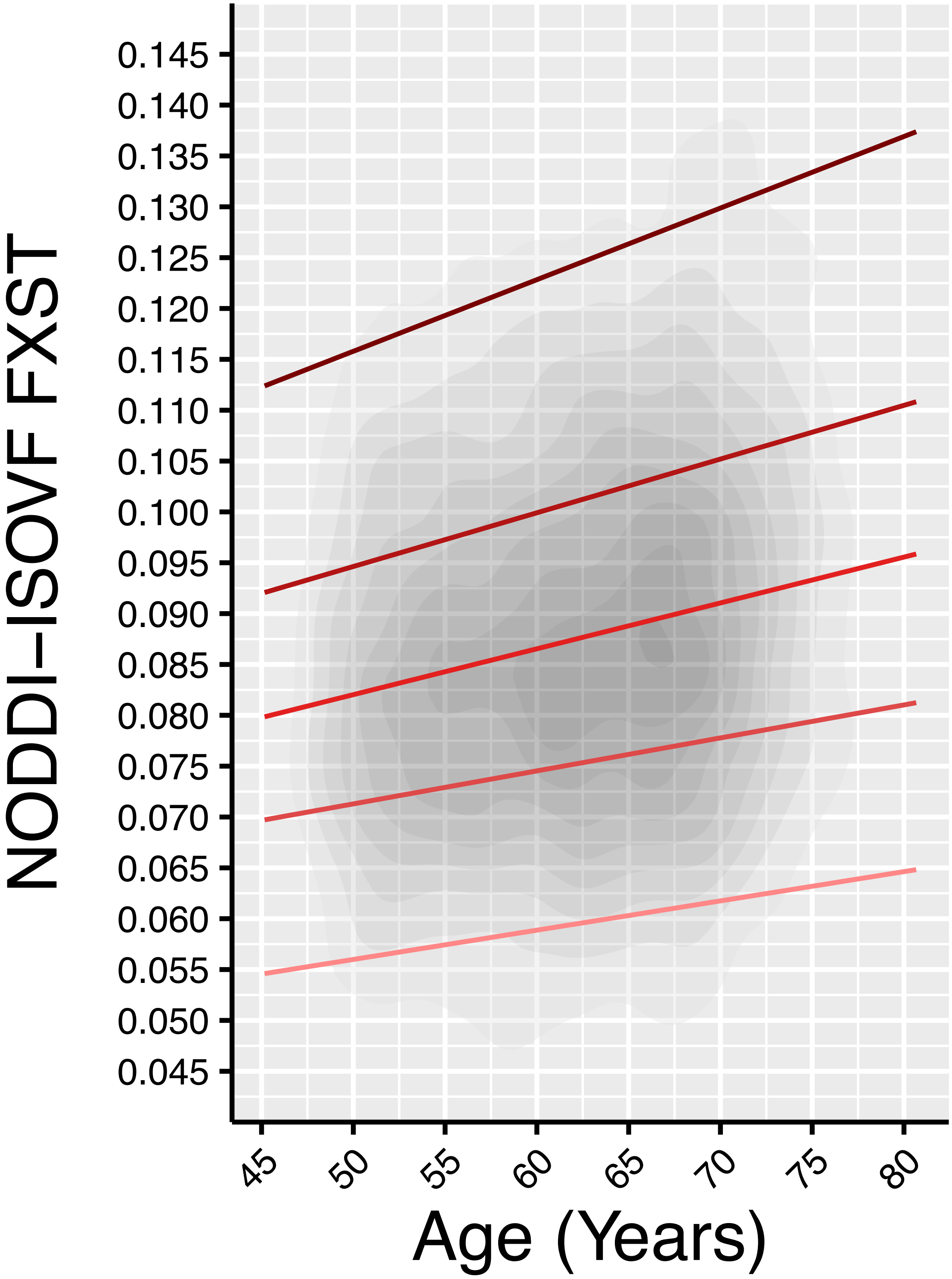

Figure S215. Full size normative centile reference curves calculated for the fornix (*crus*) / *stria terminalis* tract for NODDI-ISOVF in females. Solid colored lines, ordered from lightest to darkest, indicate the following centiles: 5th, 25th, 50th, 75th, 95th. Gray overlay reflects kernel density (darker=greater degree of data point overlap). FXST = fornix (*crus*) / *stria terminalis.*

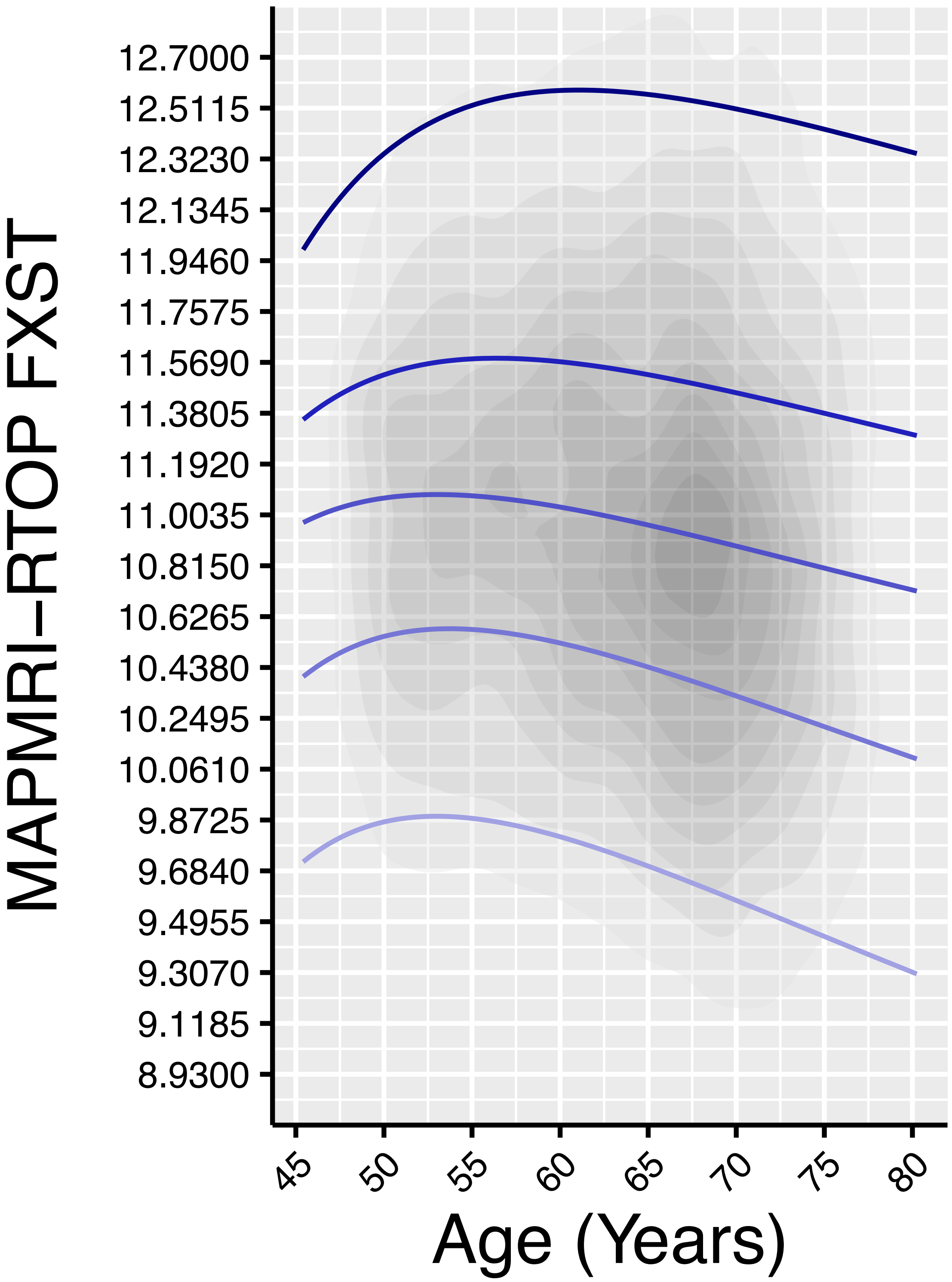

Figure S216. Full size normative centile reference curves calculated for the fornix (*crus*) / *stria terminalis* tract for MAPMRI-RTOP in males. Solid colored lines, ordered from lightest to darkest, indicate the following centiles: 5th, 25th, 50th, 75th, 95th. Gray overlay reflects kernel density (darker=greater degree of data point overlap). FXST = fornix (*crus*) / *stria terminalis.*

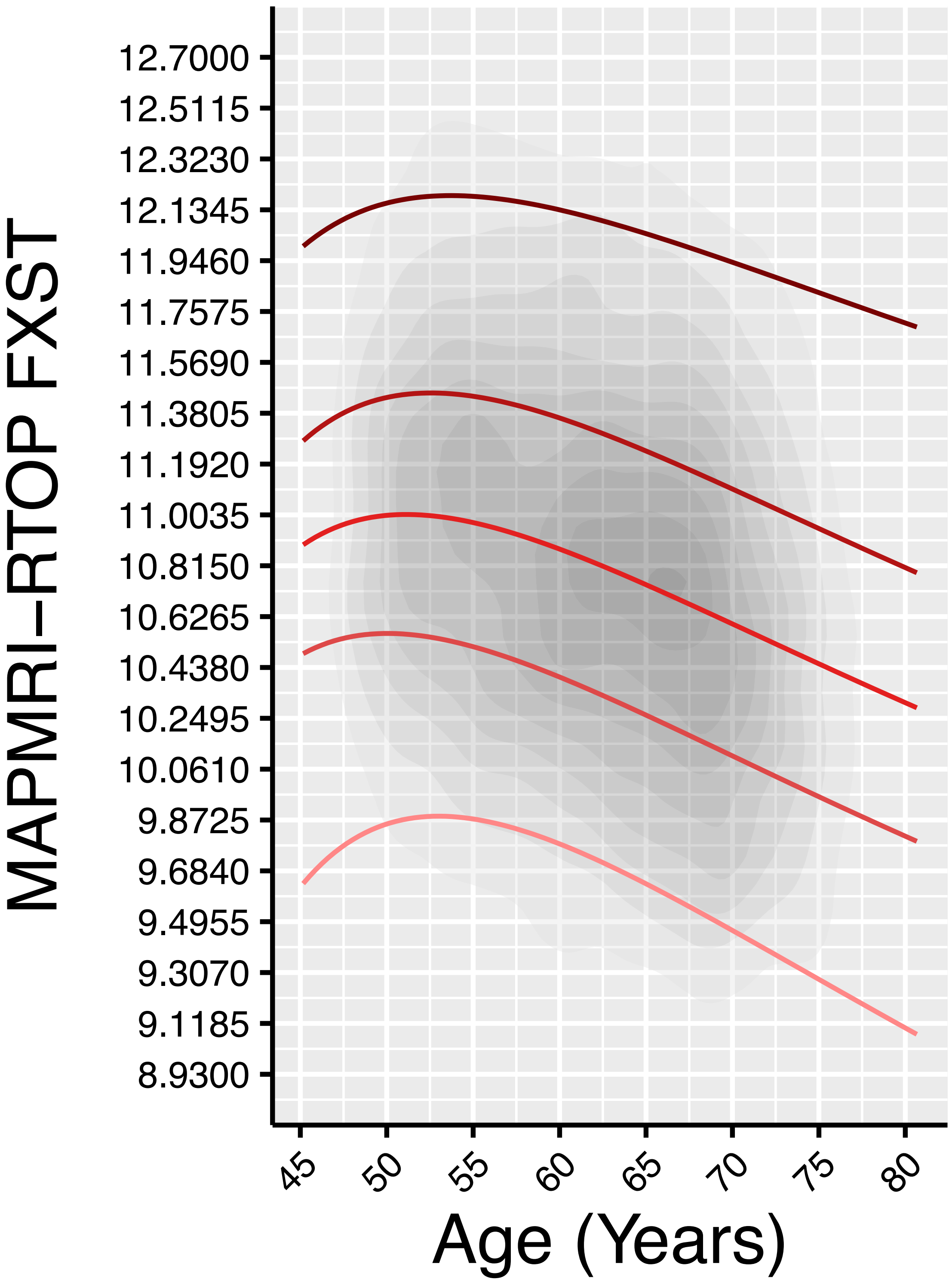

Figure S217. Full size normative centile reference curves calculated for the fornix (*crus*) / *stria terminalis* tract for MAPMRI-RTOP in females. Solid colored lines, ordered from lightest to darkest, indicate the following centiles: 5th, 25th, 50th, 75th, 95th. Gray overlay reflects kernel density (darker=greater degree of data point overlap). FXST = fornix (*crus*) / *stria terminalis.*

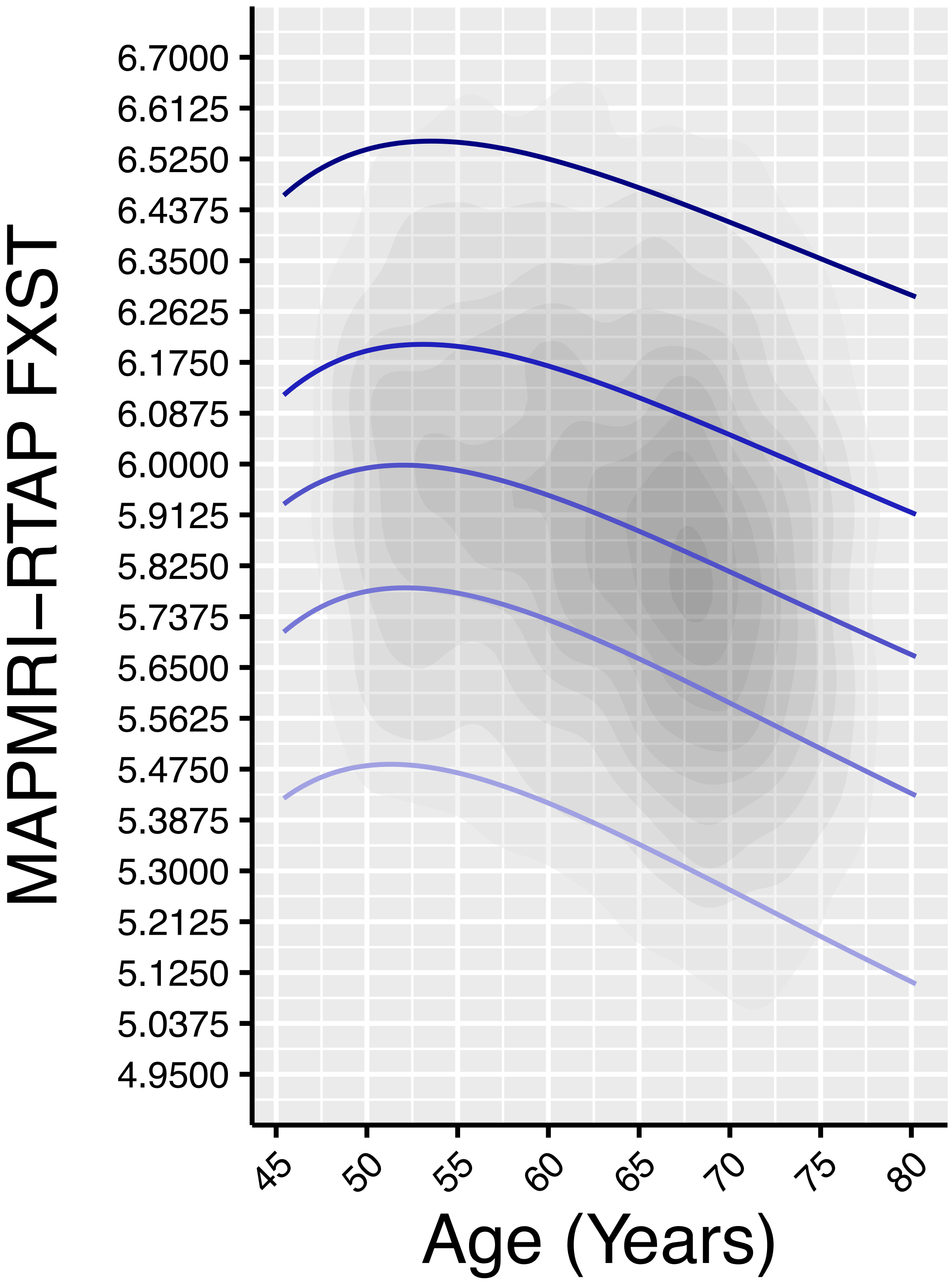

Figure S218. Full size normative centile reference curves calculated for the fornix (*crus*) / *stria terminalis* tract for MAPMRI-RTAP in males. Solid colored lines, ordered from lightest to darkest, indicate the following centiles: 5th, 25th, 50th, 75th, 95th. Gray overlay reflects kernel density (darker=greater degree of data point overlap). FXST = fornix (*crus*) / *stria terminalis.*

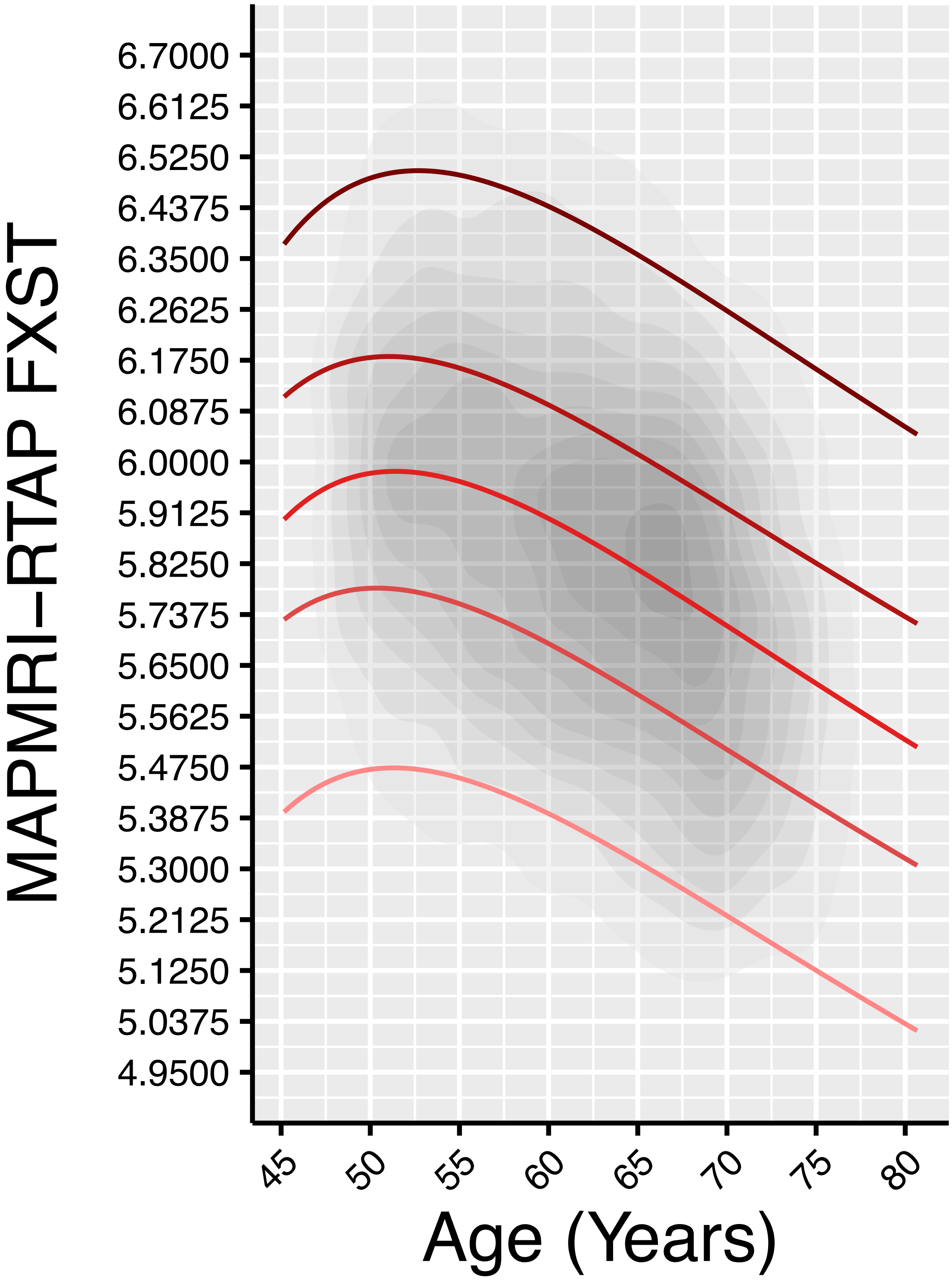

Figure S219. Full size normative centile reference curves calculated for the fornix (*crus*) / *stria terminalis* tract for MAPMRI-RTAP in females. Solid colored lines, ordered from lightest to darkest, indicate the following centiles: 5th, 25th, 50th, 75th, 95th. Gray overlay reflects kernel density (darker=greater degree of data point overlap). FXST = fornix (*crus*) / *stria terminalis.*

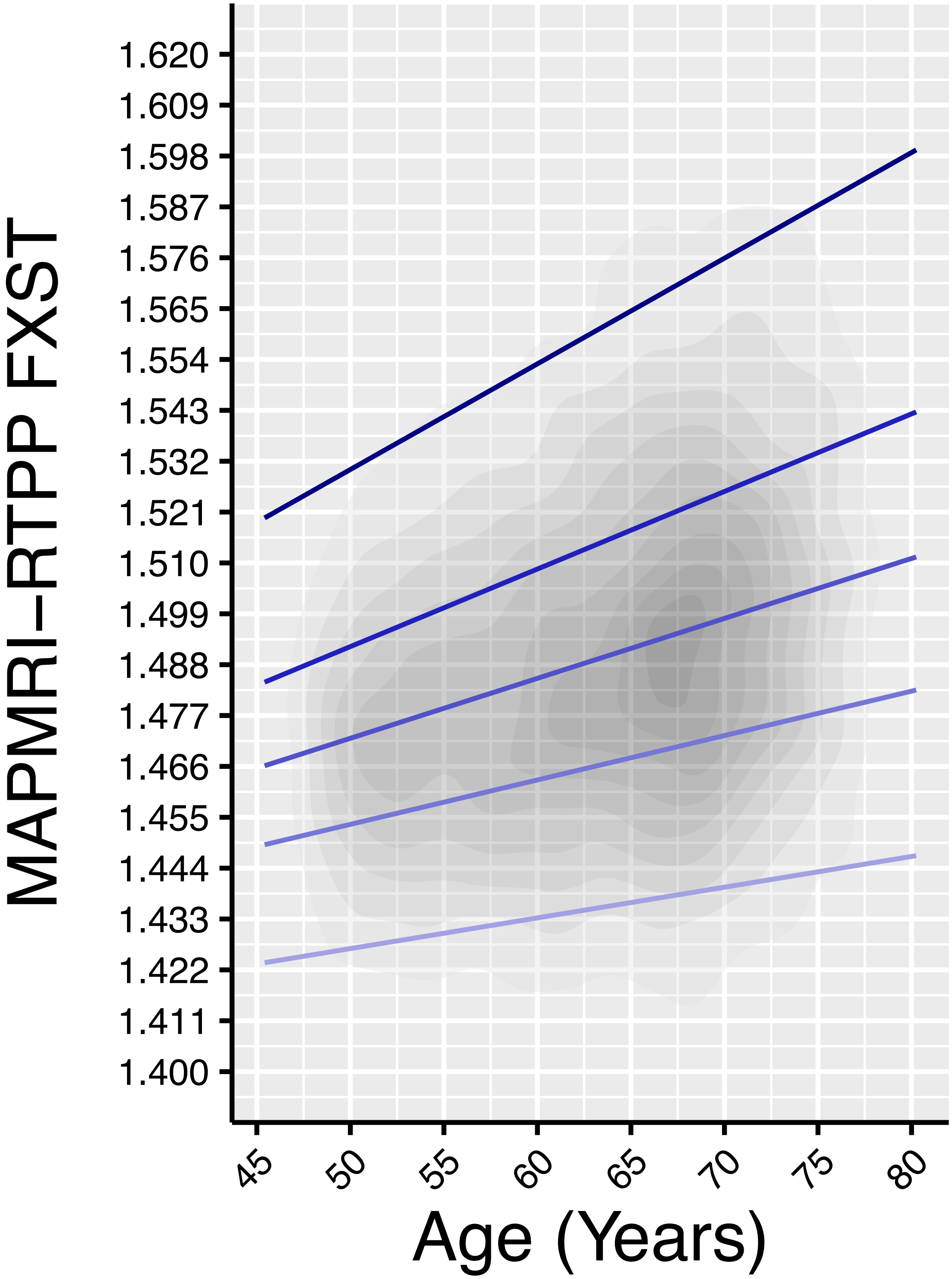

Figure S220. Full size normative centile reference curves calculated for the fornix (*crus*) / *stria terminalis* tract for MAPMRI-RTPP in males. Solid colored lines, ordered from lightest to darkest, indicate the following centiles: 5th, 25th, 50th, 75th, 95th. Gray overlay reflects kernel density (darker=greater degree of data point overlap). FXST = fornix (*crus*) / *stria terminalis.*

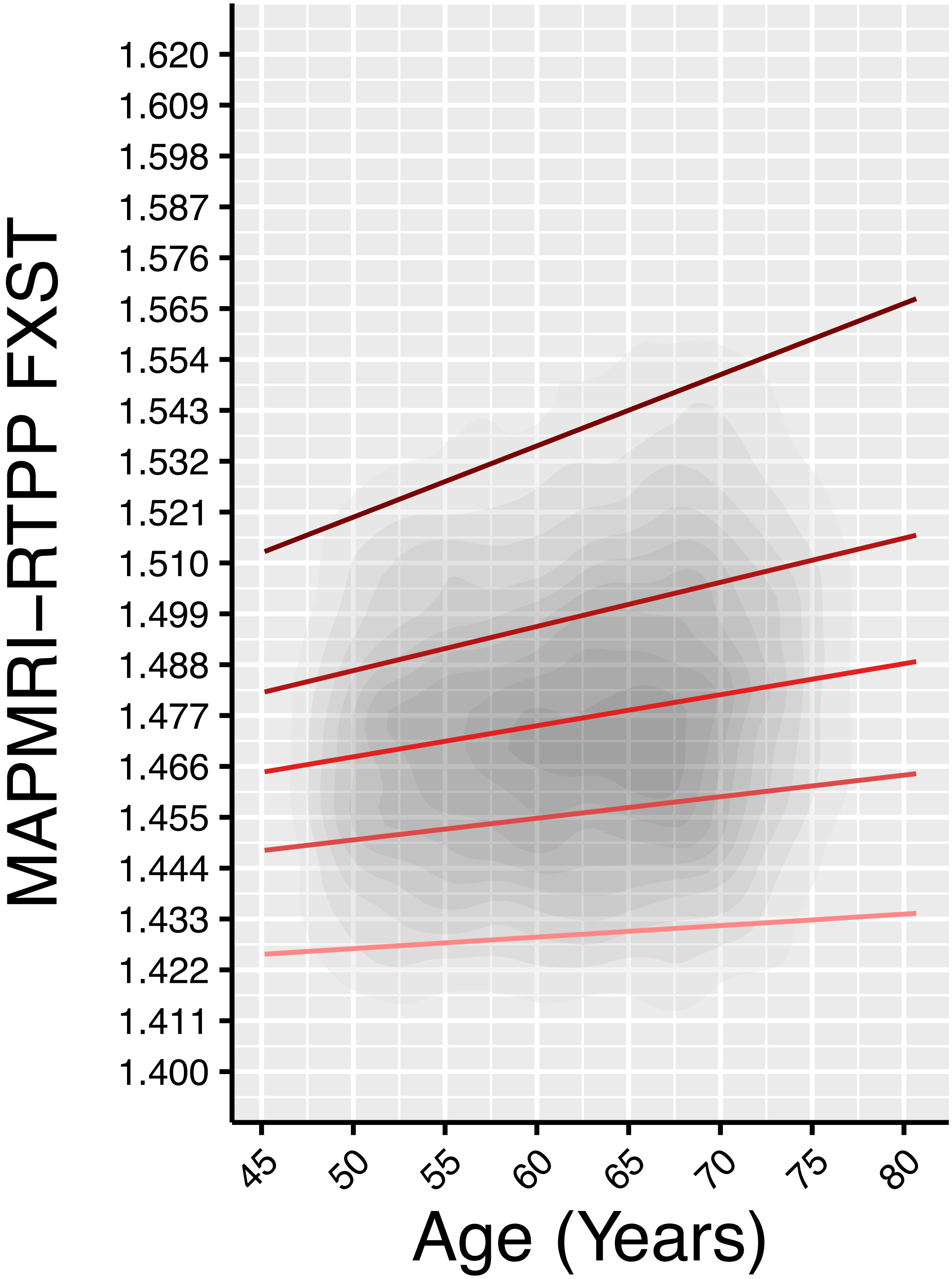

Figure S221. Full size normative centile reference curves calculated for the fornix (*crus*) / *stria terminalis* tract for MAPMRI-RTPP in females. Solid colored lines, ordered from lightest to darkest, indicate the following centiles: 5th, 25th, 50th, 75th, 95th. Gray overlay reflects kernel density (darker=greater degree of data point overlap). FXST = fornix (*crus*) / *stria terminalis.*

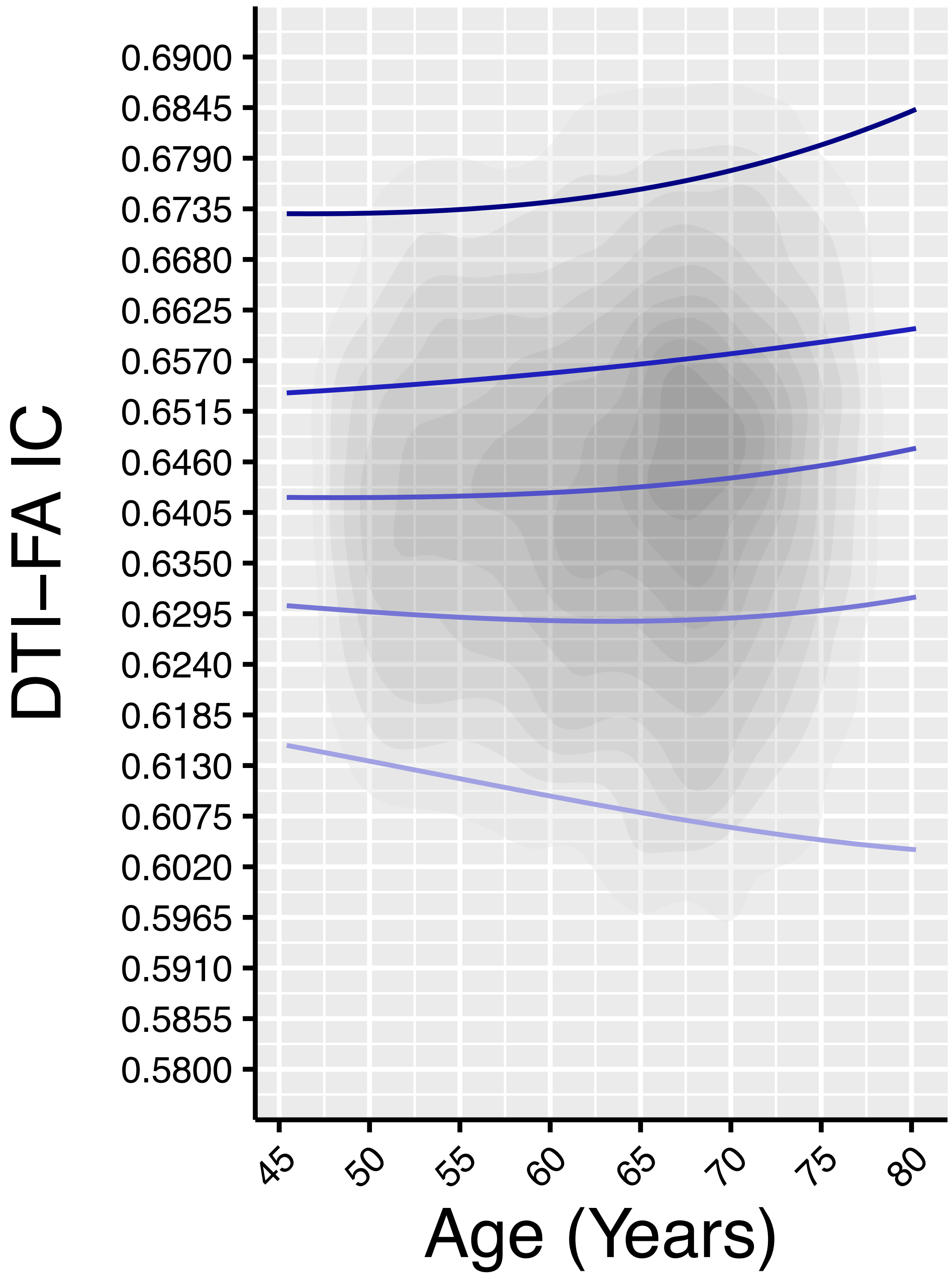

Figure S222. Full size normative centile reference curves calculated for the internal capsule tract for DTI-FA in males. Solid colored lines, ordered from lightest to darkest, indicate the following centiles: 5th, 25th, 50th, 75th, 95th. Gray overlay reflects kernel density (darker=greater degree of data point overlap). IC = internal capsule.

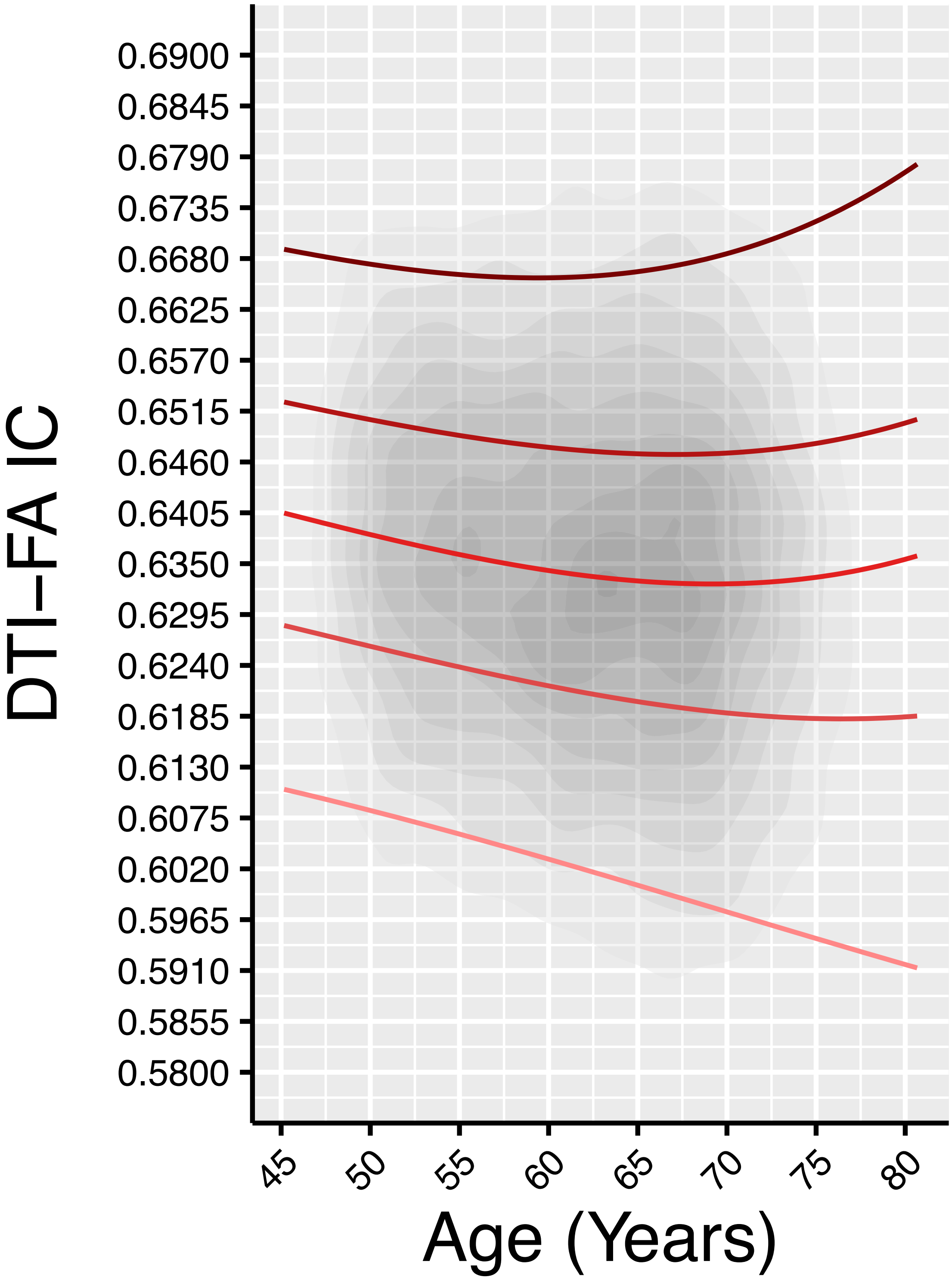

Figure S223. Full size normative centile reference curves calculated for the internal capsule tract for DTI-FA in females. Solid colored lines, ordered from lightest to darkest, indicate the following centiles: 5th, 25th, 50th, 75th, 95th. Gray overlay reflects kernel density (darker=greater degree of data point overlap). IC = internal capsule.

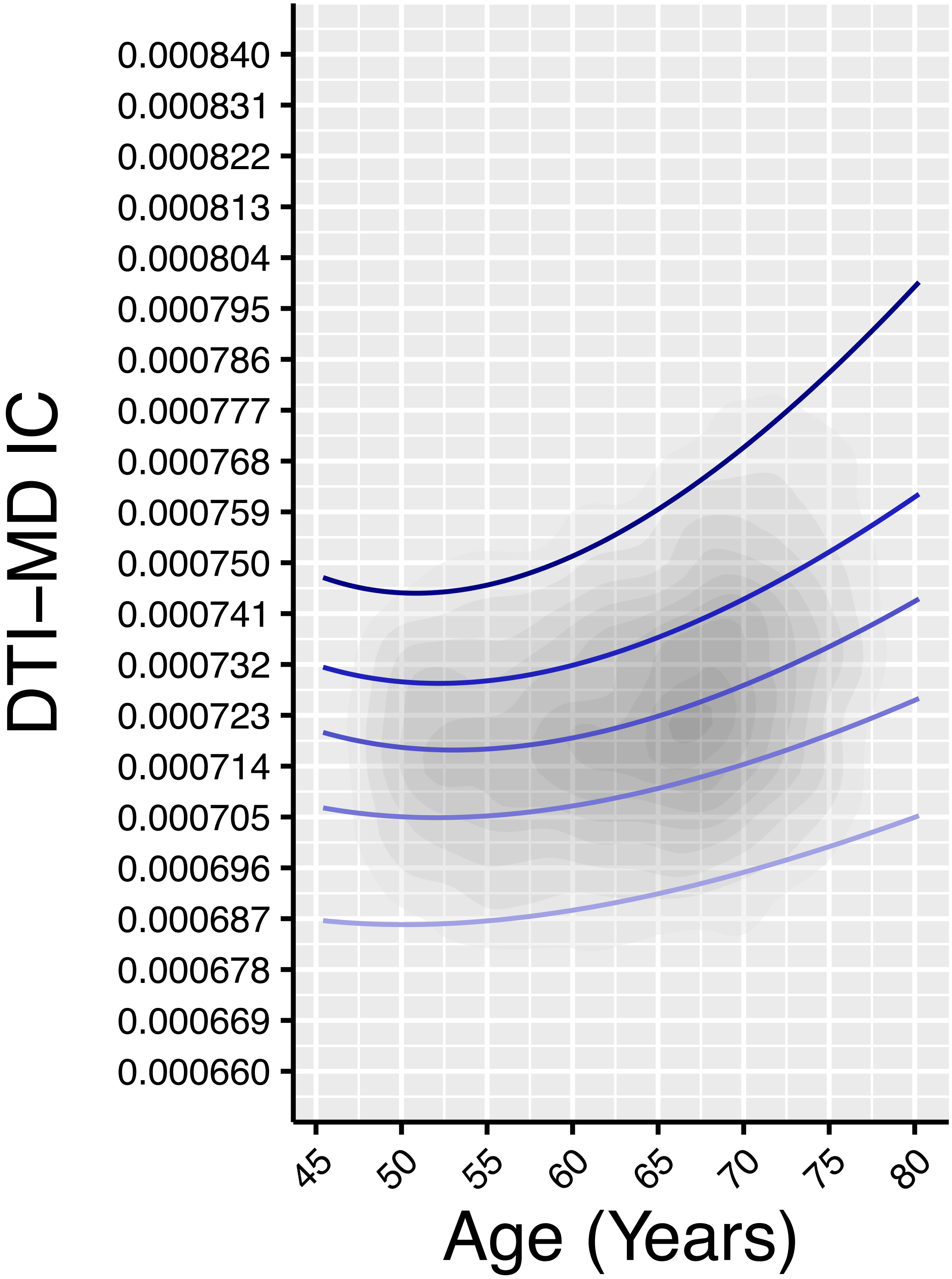

Figure S224. Full size normative centile reference curves calculated for the internal capsule tract for DTI-MD in males. Solid colored lines, ordered from lightest to darkest, indicate the following centiles: 5th, 25th, 50th, 75th, 95th. Gray overlay reflects kernel density (darker=greater degree of data point overlap). IC = internal capsule.

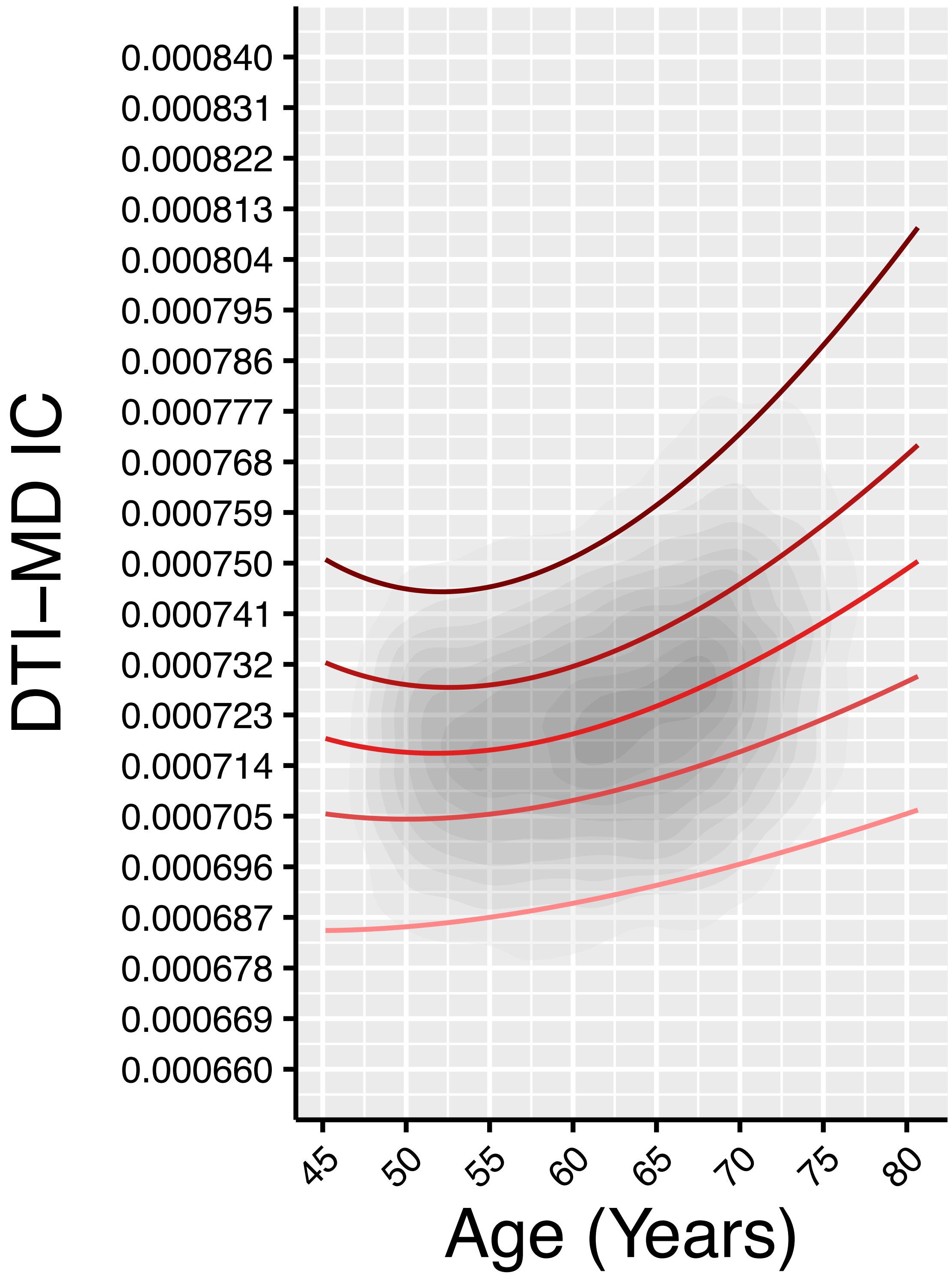

Figure S225. Full size normative centile reference curves calculated for the internal capsule tract for DTI-MD in females. Solid colored lines, ordered from lightest to darkest, indicate the following centiles: 5th, 25th, 50th, 75th, 95th. Gray overlay reflects kernel density (darker=greater degree of data point overlap). IC = internal capsule.

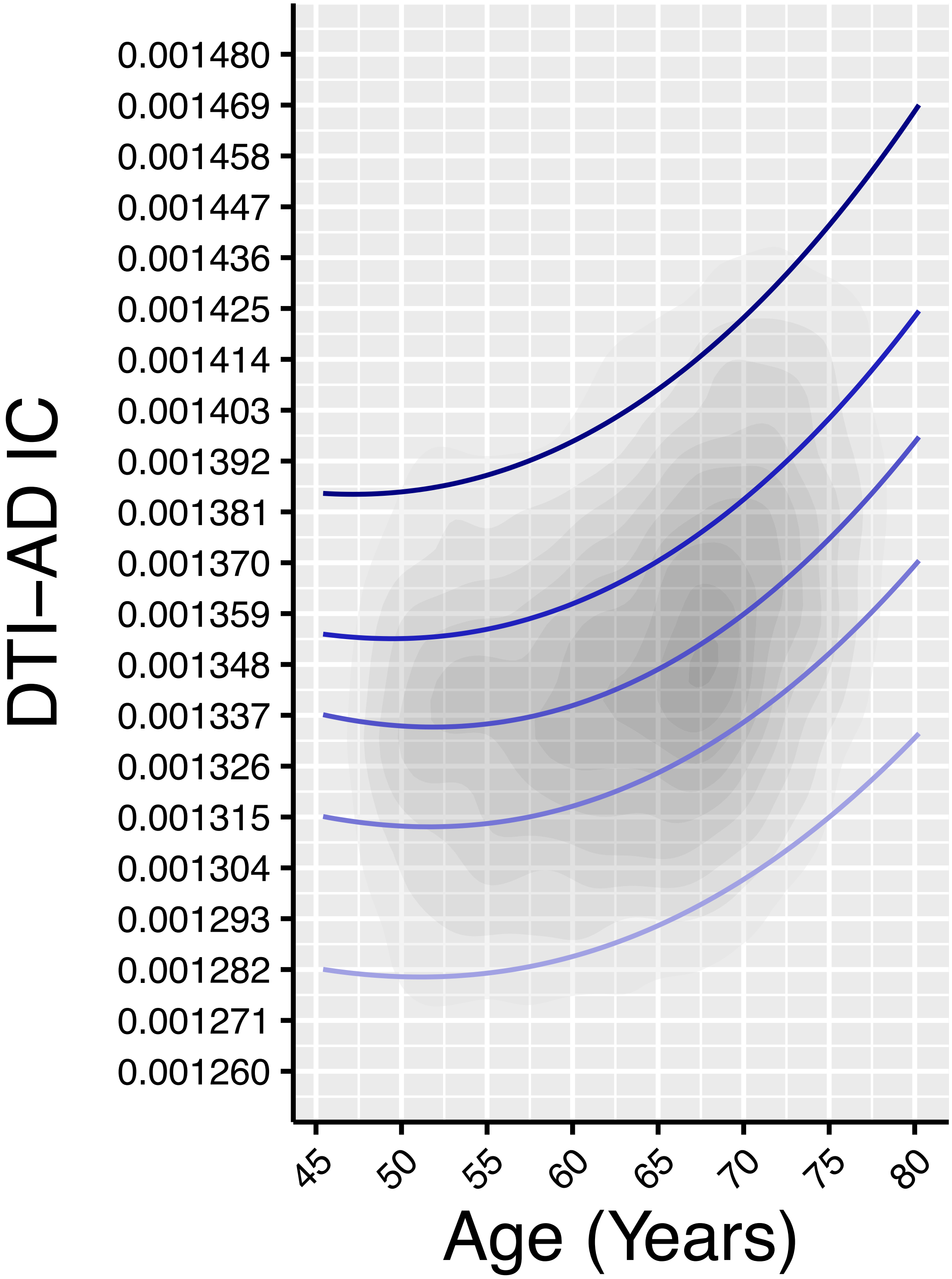

Figure S226. Full size normative centile reference curves calculated for the internal capsule tract for DTI-AD in males. Solid colored lines, ordered from lightest to darkest, indicate the following centiles: 5th, 25th, 50th, 75th, 95th. Gray overlay reflects kernel density (darker=greater degree of data point overlap). IC = internal capsule.

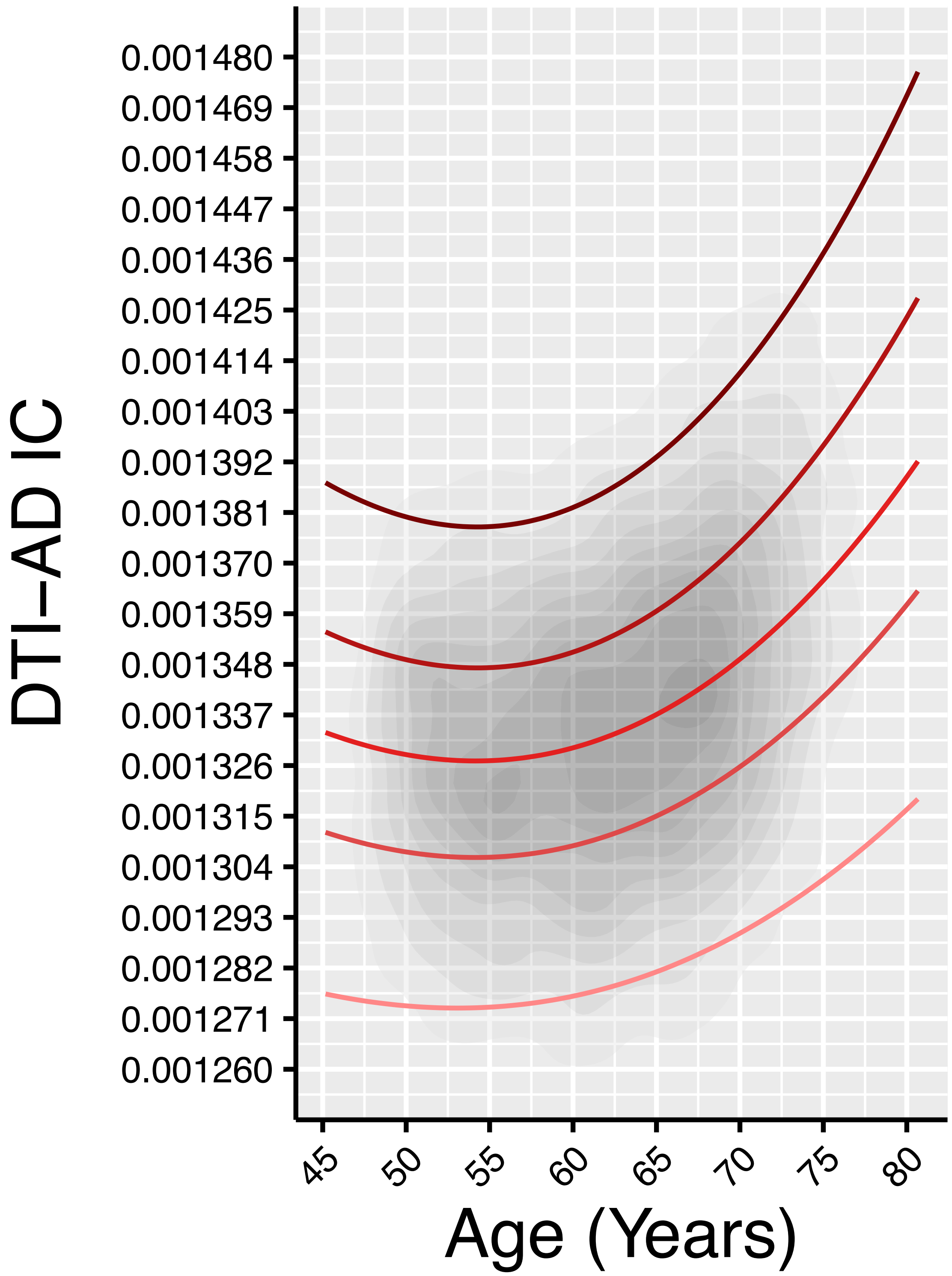

Figure S227. Full size normative centile reference curves calculated for the internal capsule tract for DTI-AD in females. Solid colored lines, ordered from lightest to darkest, indicate the following centiles: 5th, 25th, 50th, 75th, 95th. Gray overlay reflects kernel density (darker=greater degree of data point overlap). IC = internal capsule.

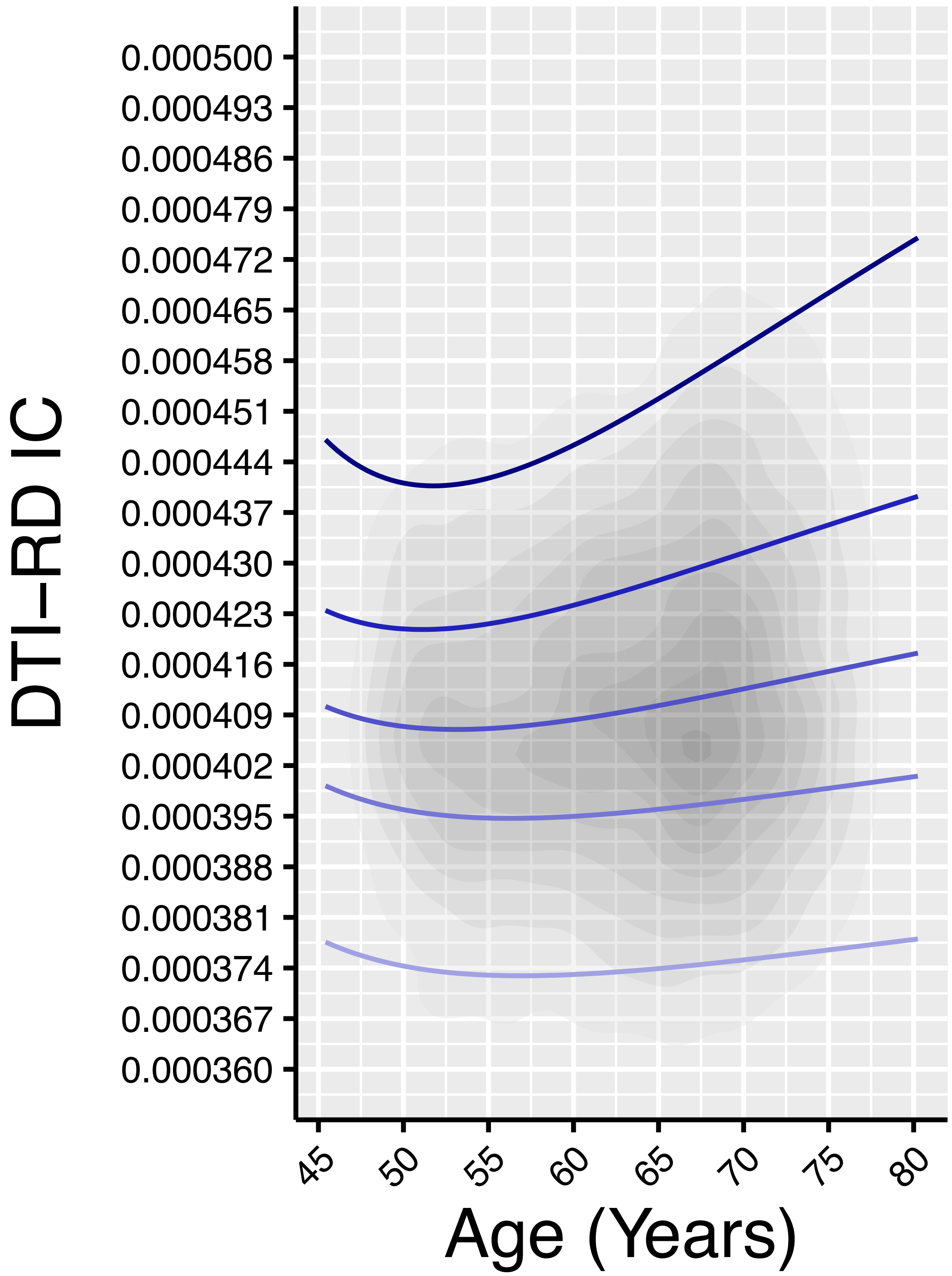

Figure S228. Full size normative centile reference curves calculated for the internal capsule tract for DTI-RD in males. Solid colored lines, ordered from lightest to darkest, indicate the following centiles: 5th, 25th, 50th, 75th, 95th. Gray overlay reflects kernel density (darker=greater degree of data point overlap). IC = internal capsule.

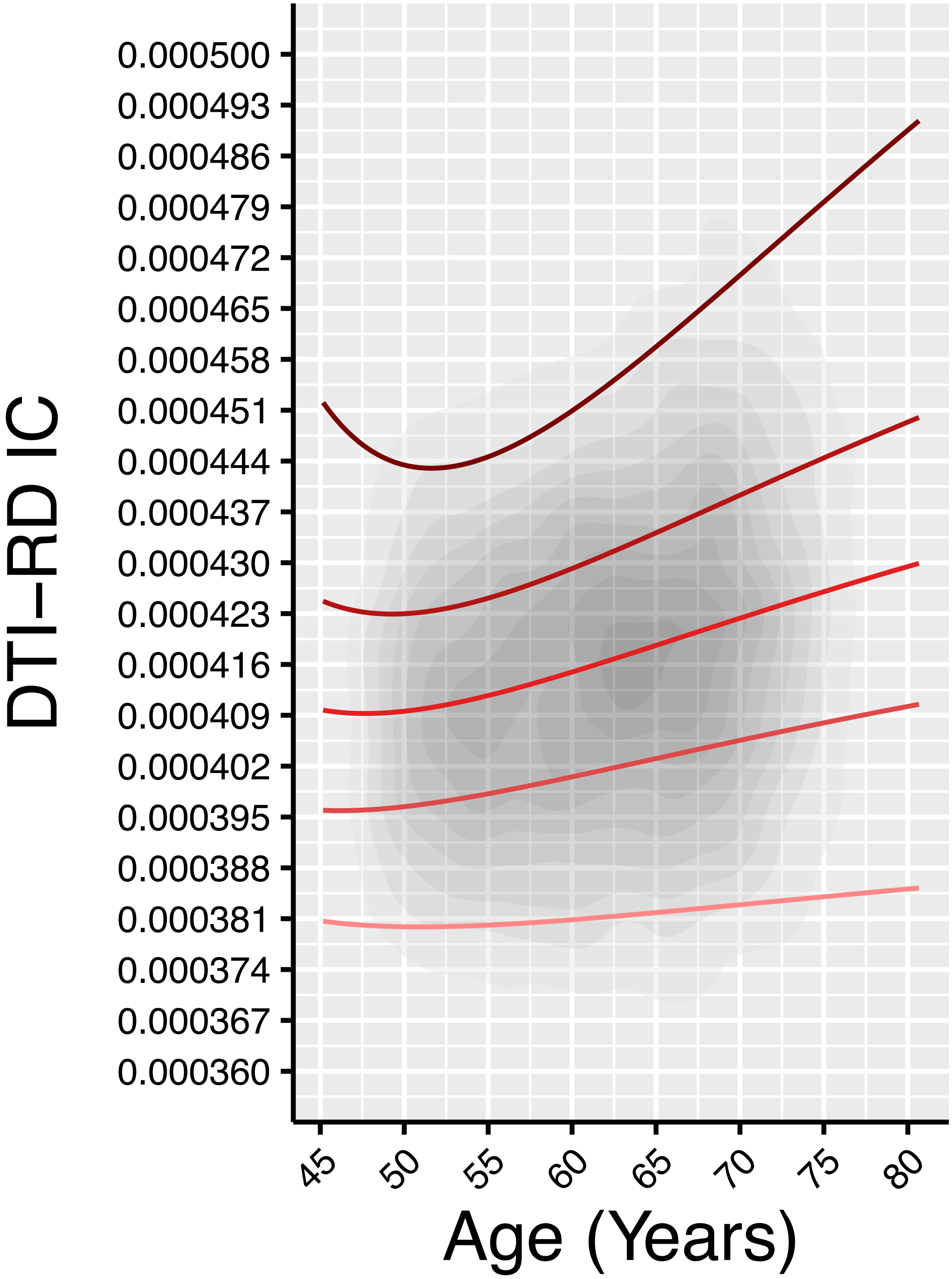

Figure S229. Full size normative centile reference curves calculated for the internal capsule tract for DTI-RD in females. Solid colored lines, ordered from lightest to darkest, indicate the following centiles: 5th, 25th, 50th, 75th, 95th. Gray overlay reflects kernel density (darker=greater degree of data point overlap). IC = internal capsule.

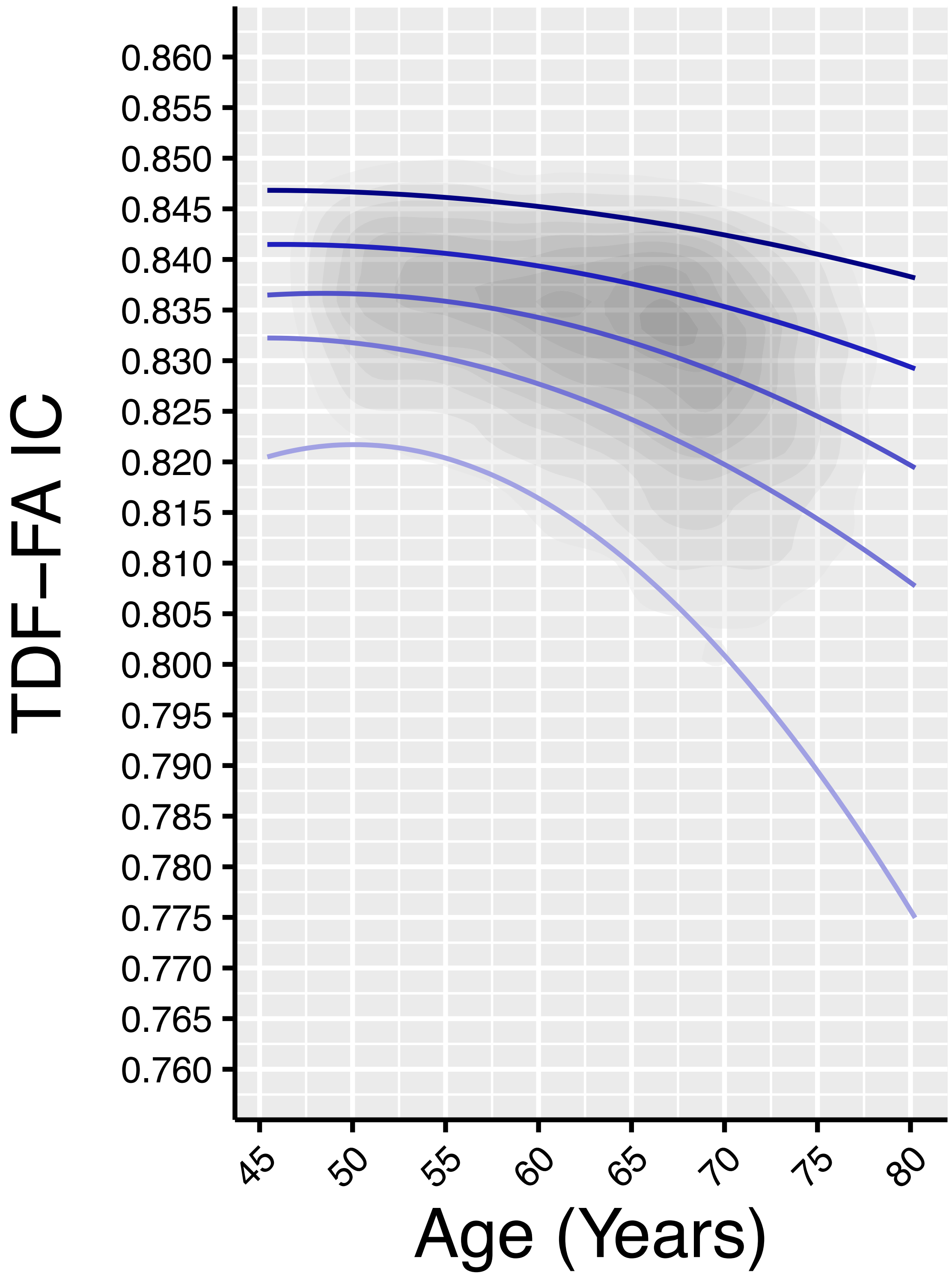

Figure S230. Full size normative centile reference curves calculated for the internal capsule tract for TDF-FA in males. Solid colored lines, ordered from lightest to darkest, indicate the following centiles: 5th, 25th, 50th, 75th, 95th. Gray overlay reflects kernel density (darker=greater degree of data point overlap). IC = internal capsule.

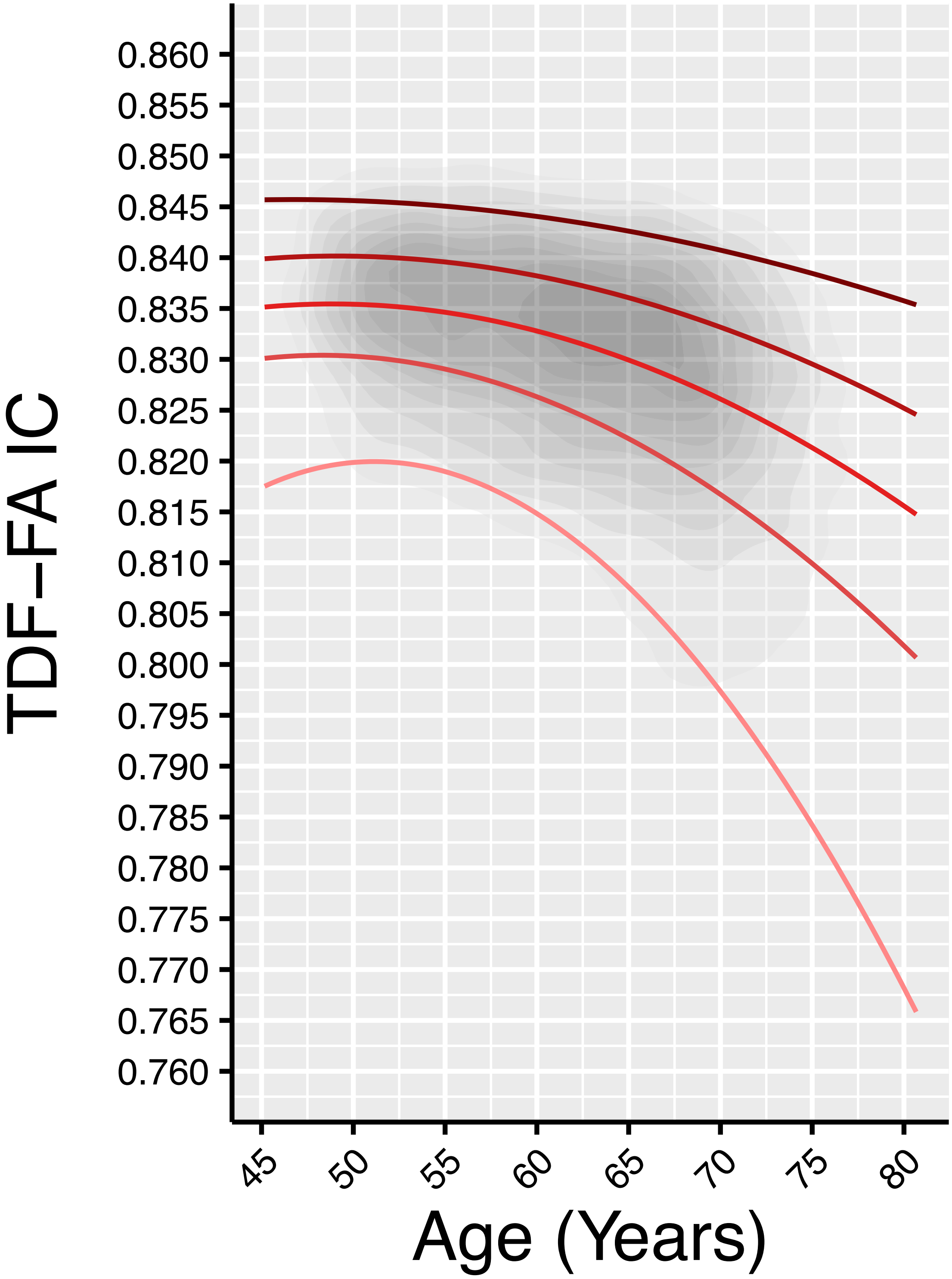

Figure S231. Full size normative centile reference curves calculated for the internal capsule tract for TDF-FA in females. Solid colored lines, ordered from lightest to darkest, indicate the following centiles: 5th, 25th, 50th, 75th, 95th. Gray overlay reflects kernel density (darker=greater degree of data point overlap). IC = internal capsule.

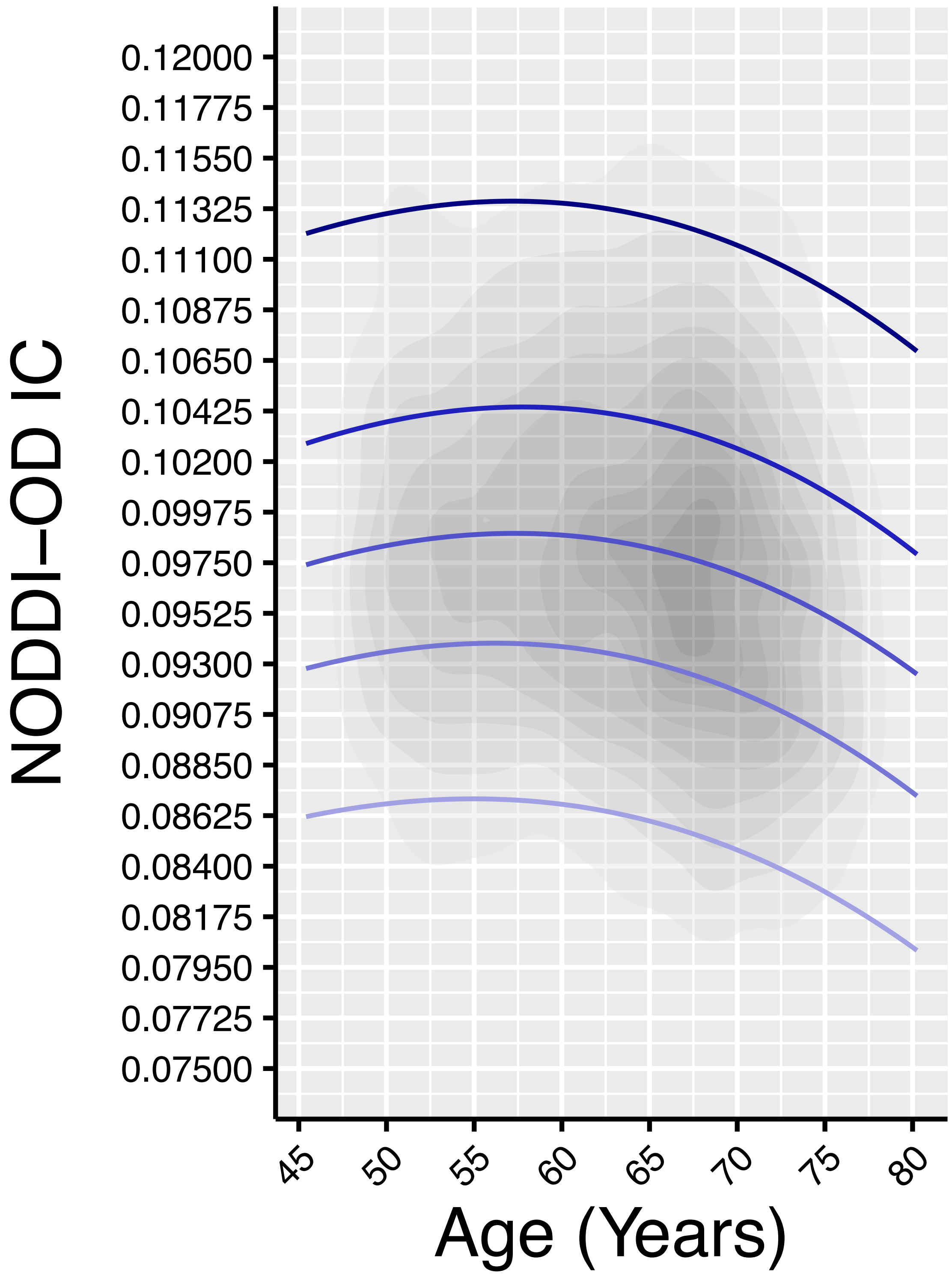

Figure S232. Full size normative centile reference curves calculated for the internal capsule tract for NODDI-OD in males. Solid colored lines, ordered from lightest to darkest, indicate the following centiles: 5th, 25th, 50th, 75th, 95th. Gray overlay reflects kernel density (darker=greater degree of data point overlap). IC = internal capsule.

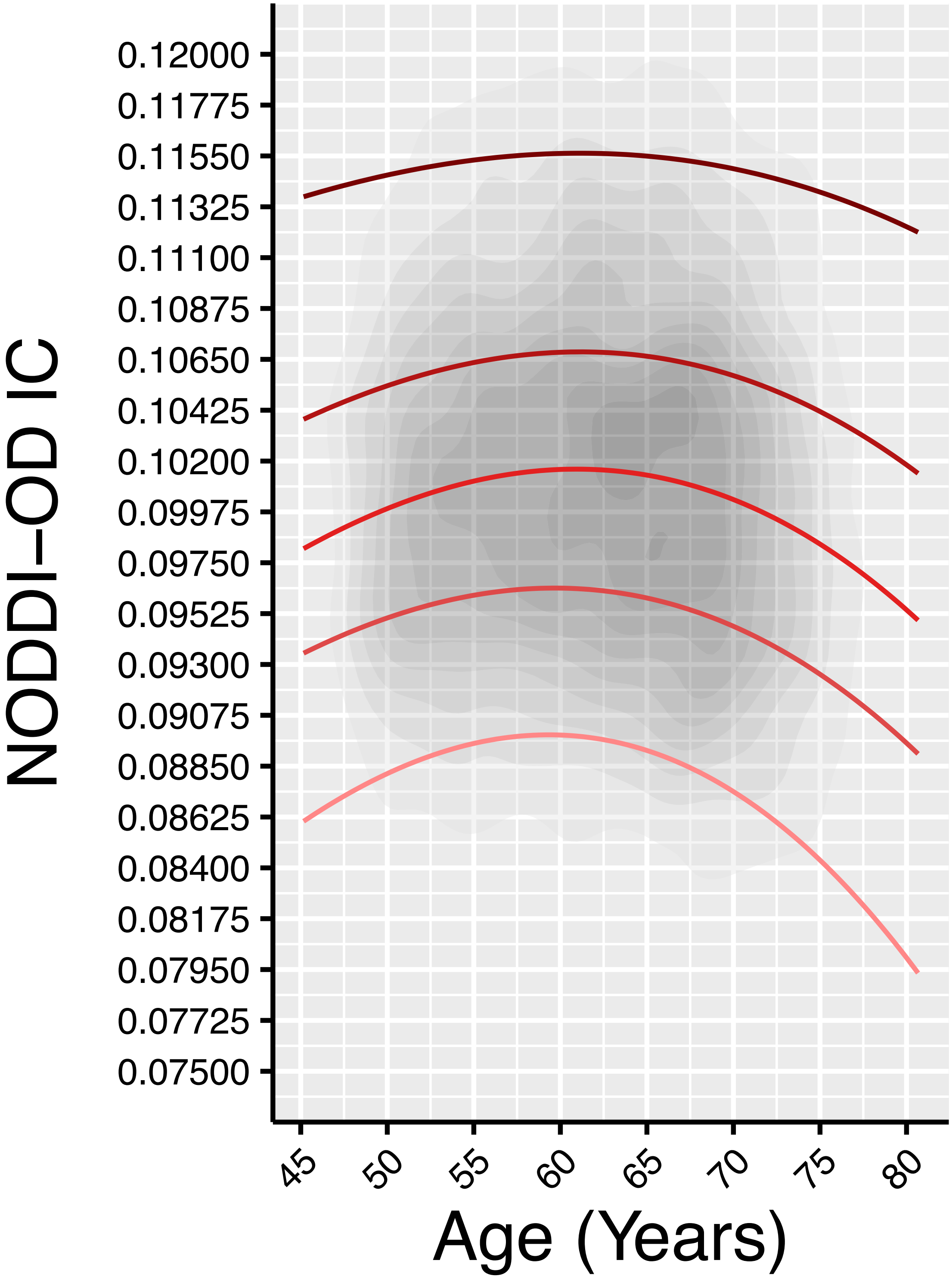

Figure S233. Full size normative centile reference curves calculated for the internal capsule tract for NODDI-OD in females. Solid colored lines, ordered from lightest to darkest, indicate the following centiles: 5th, 25th, 50th, 75th, 95th. Gray overlay reflects kernel density (darker=greater degree of data point overlap). IC = internal capsule.

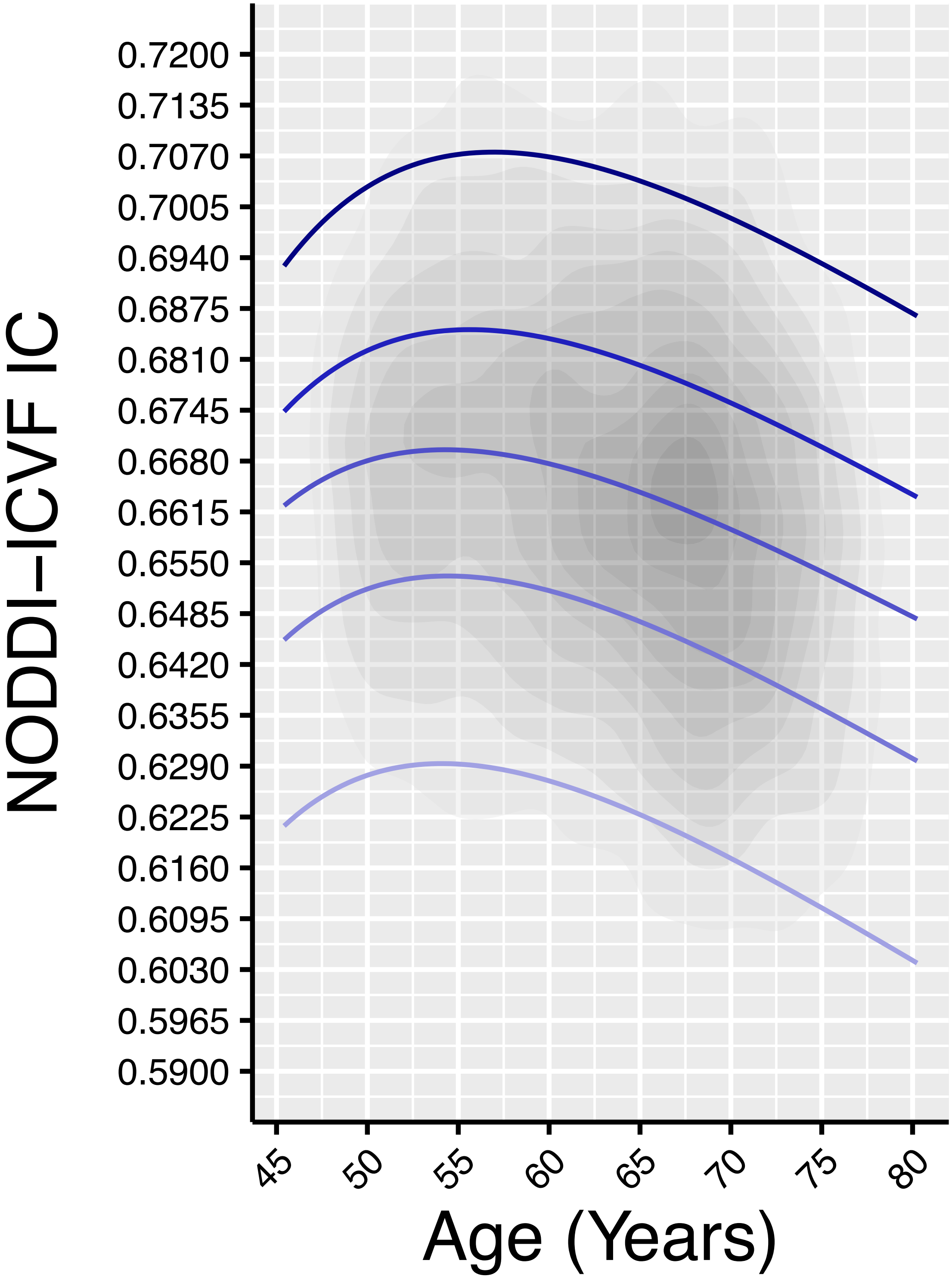

Figure S234. Full size normative centile reference curves calculated for the internal capsule tract for NODDI-ICVF in males. Solid colored lines, ordered from lightest to darkest, indicate the following centiles: 5th, 25th, 50th, 75th, 95th. Gray overlay reflects kernel density (darker=greater degree of data point overlap). IC = internal capsule.

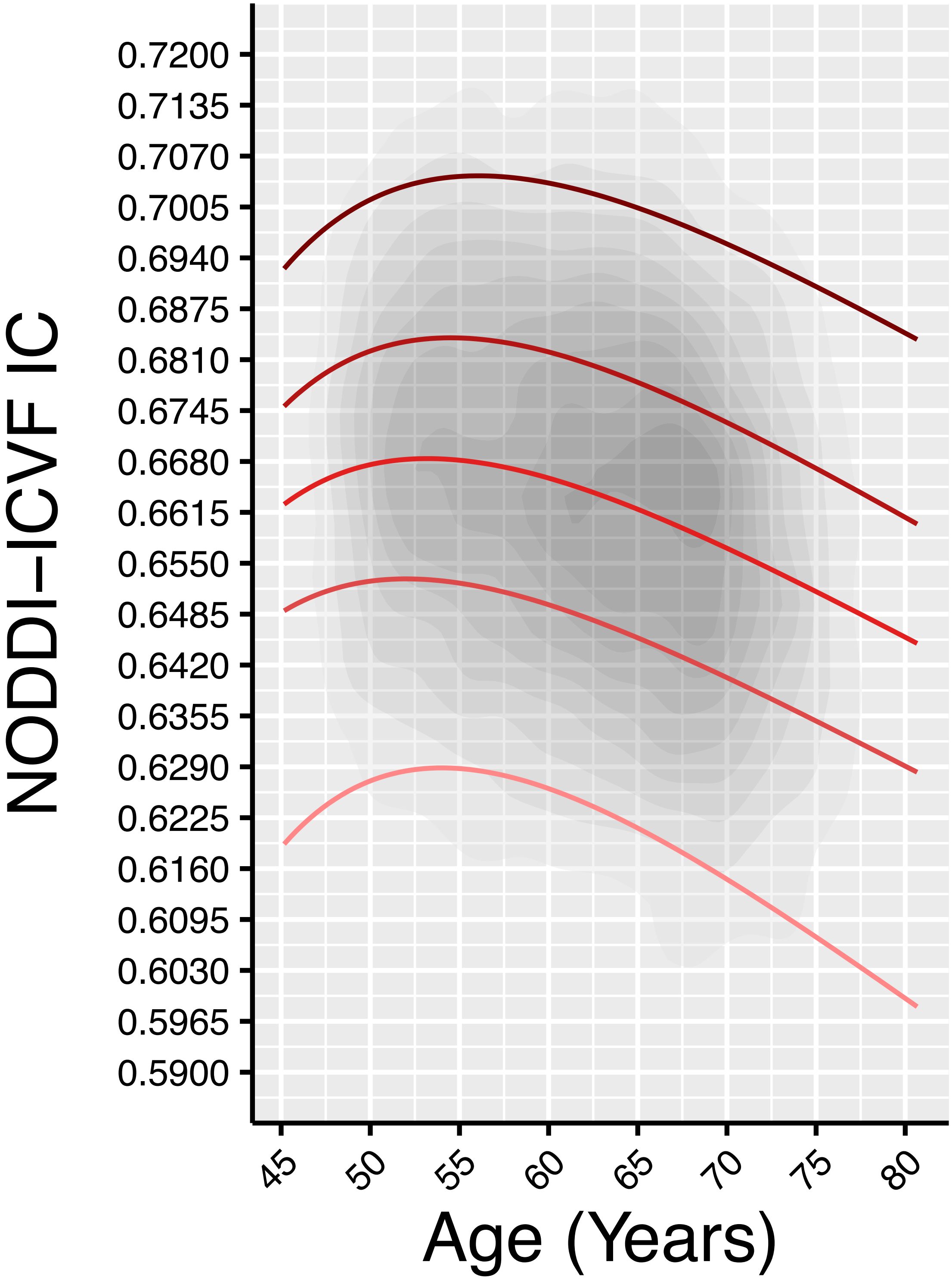

Figure S235. Full size normative centile reference curves calculated for the internal capsule tract for NODDI-ICVF in females. Solid colored lines, ordered from lightest to darkest, indicate the following centiles: 5th, 25th, 50th, 75th, 95th. Gray overlay reflects kernel density (darker=greater degree of data point overlap). IC = internal capsule.

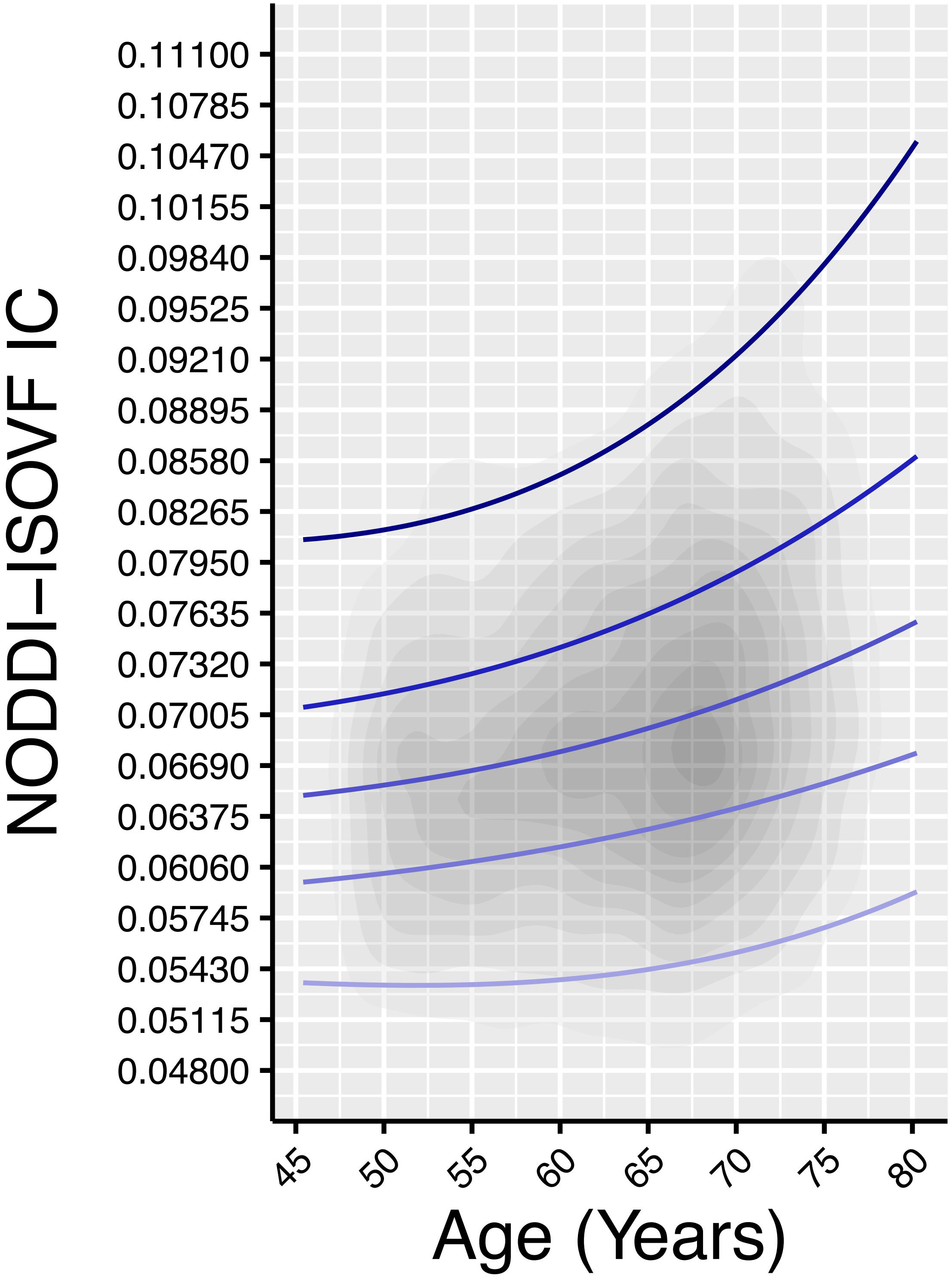

Figure S236. Full size normative centile reference curves calculated for the internal capsule tract for NODDI-ISOVF in males. Solid colored lines, ordered from lightest to darkest, indicate the following centiles: 5th, 25th, 50th, 75th, 95th. Gray overlay reflects kernel density (darker=greater degree of data point overlap). IC = internal capsule.

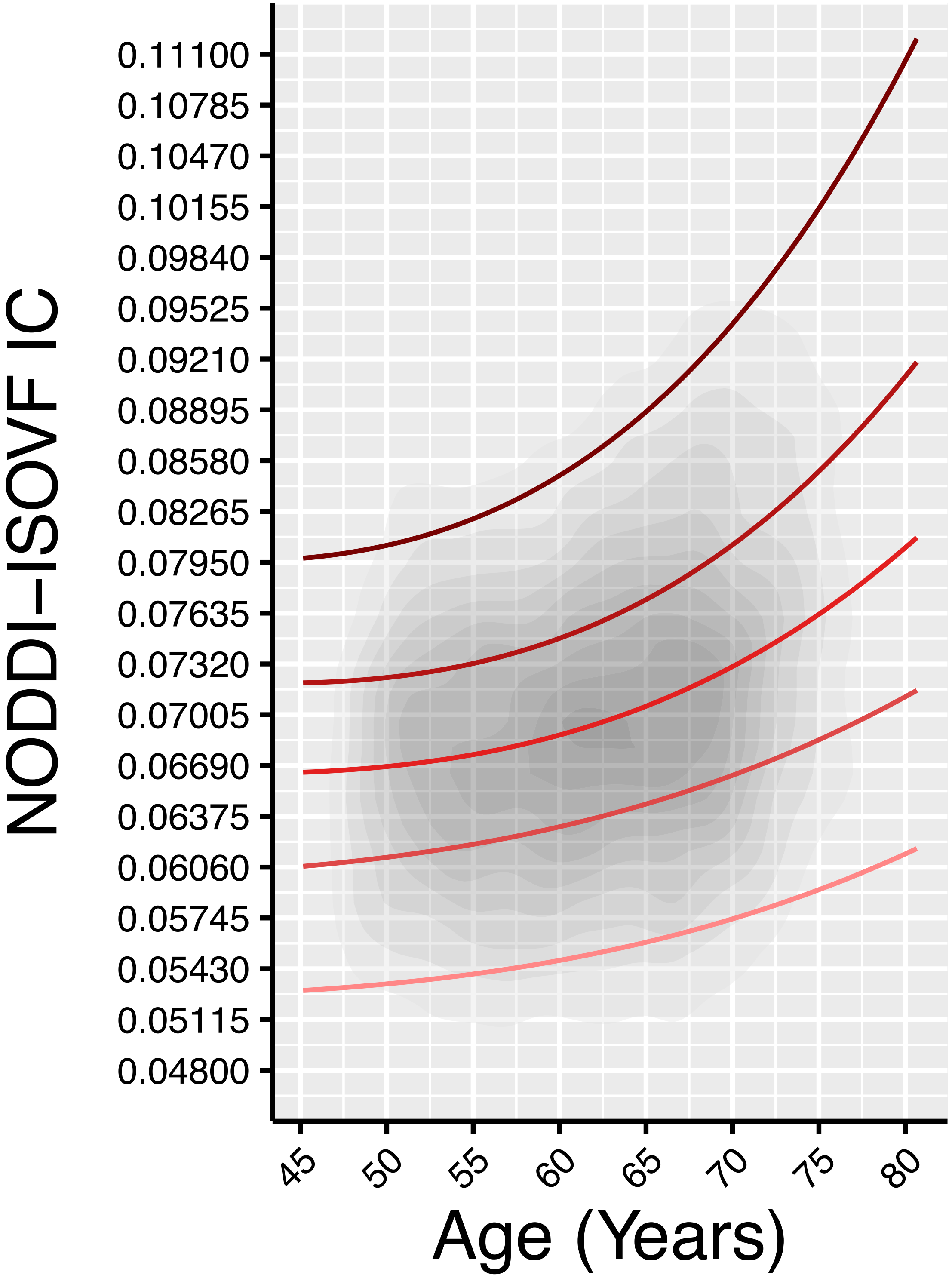

Figure S237. Full size normative centile reference curves calculated for the internal capsule tract for NODDI-ISOVF in females. Solid colored lines, ordered from lightest to darkest, indicate the following centiles: 5th, 25th, 50th, 75th, 95th. Gray overlay reflects kernel density (darker=greater degree of data point overlap). IC = internal capsule.

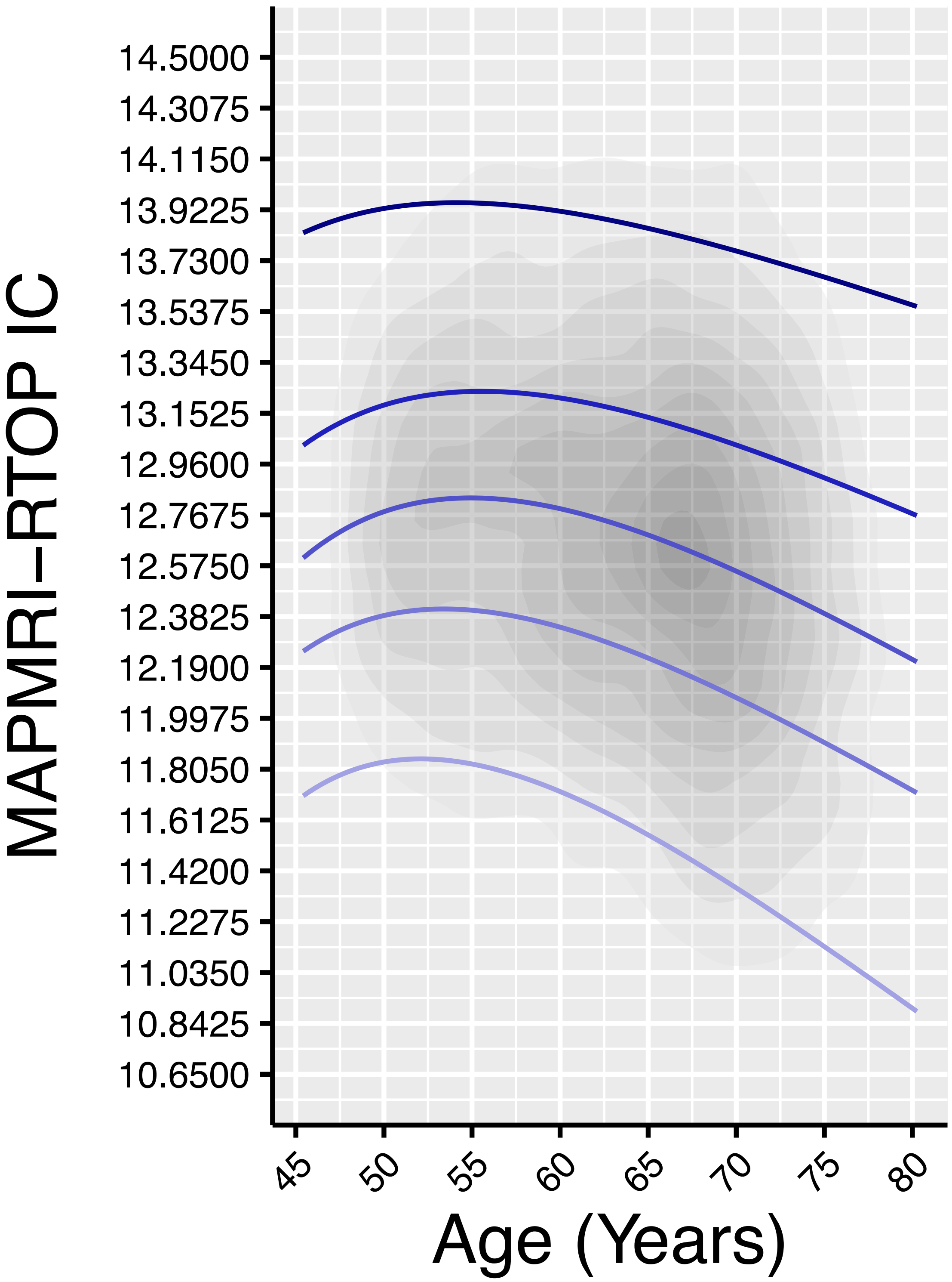

Figure S238. Full size normative centile reference curves calculated for the internal capsule tract for MAPMRI-RTOP in males. Solid colored lines, ordered from lightest to darkest, indicate the following centiles: 5th, 25th, 50th, 75th, 95th. Gray overlay reflects kernel density (darker=greater degree of data point overlap). IC = internal capsule.

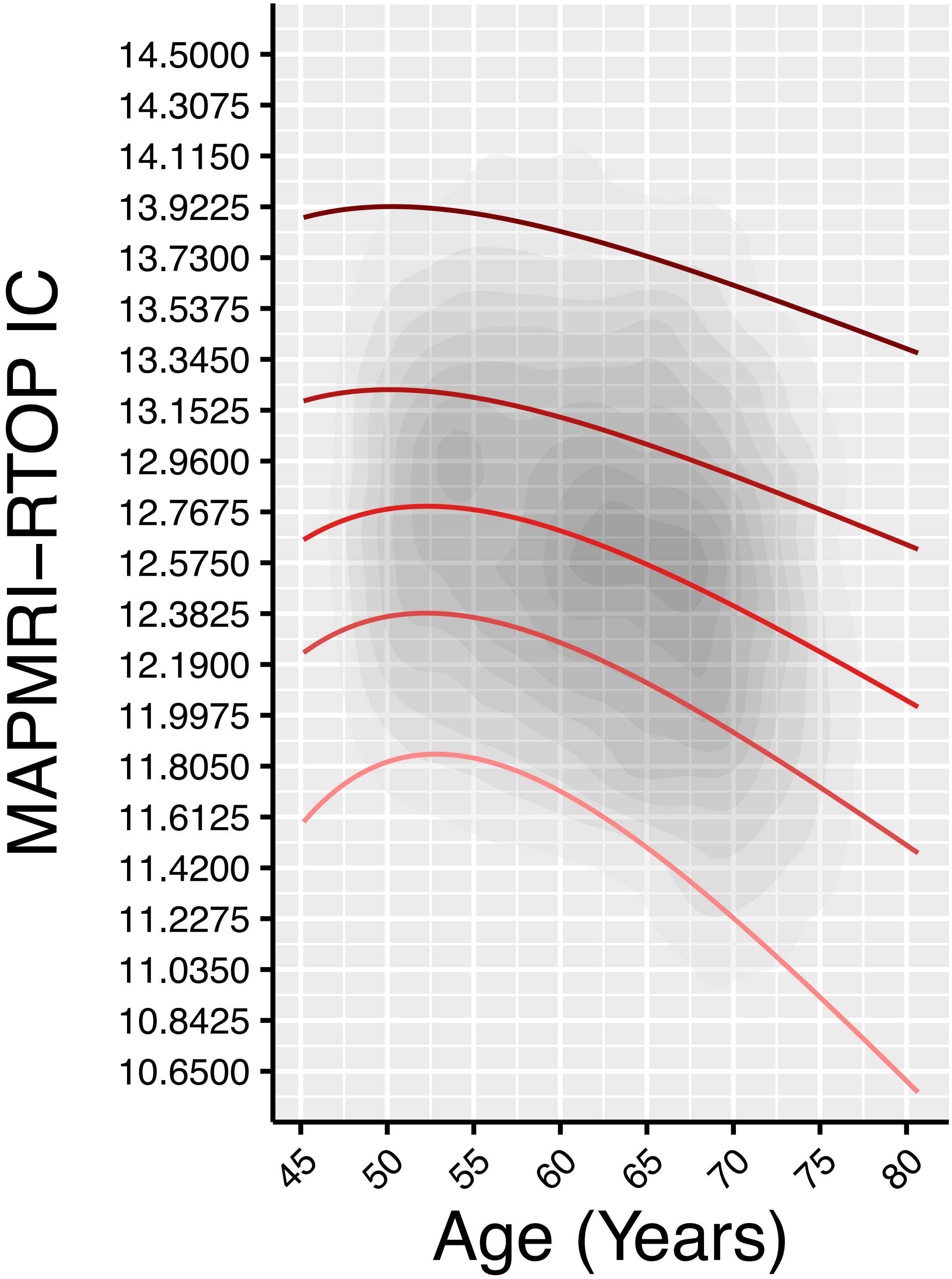

Figure S239. Full size normative centile reference curves calculated for the internal capsule tract for MAPMRI-RTOP in females. Solid colored lines, ordered from lightest to darkest, indicate the following centiles: 5th, 25th, 50th, 75th, 95th. Gray overlay reflects kernel density (darker=greater degree of data point overlap). IC = internal capsule.

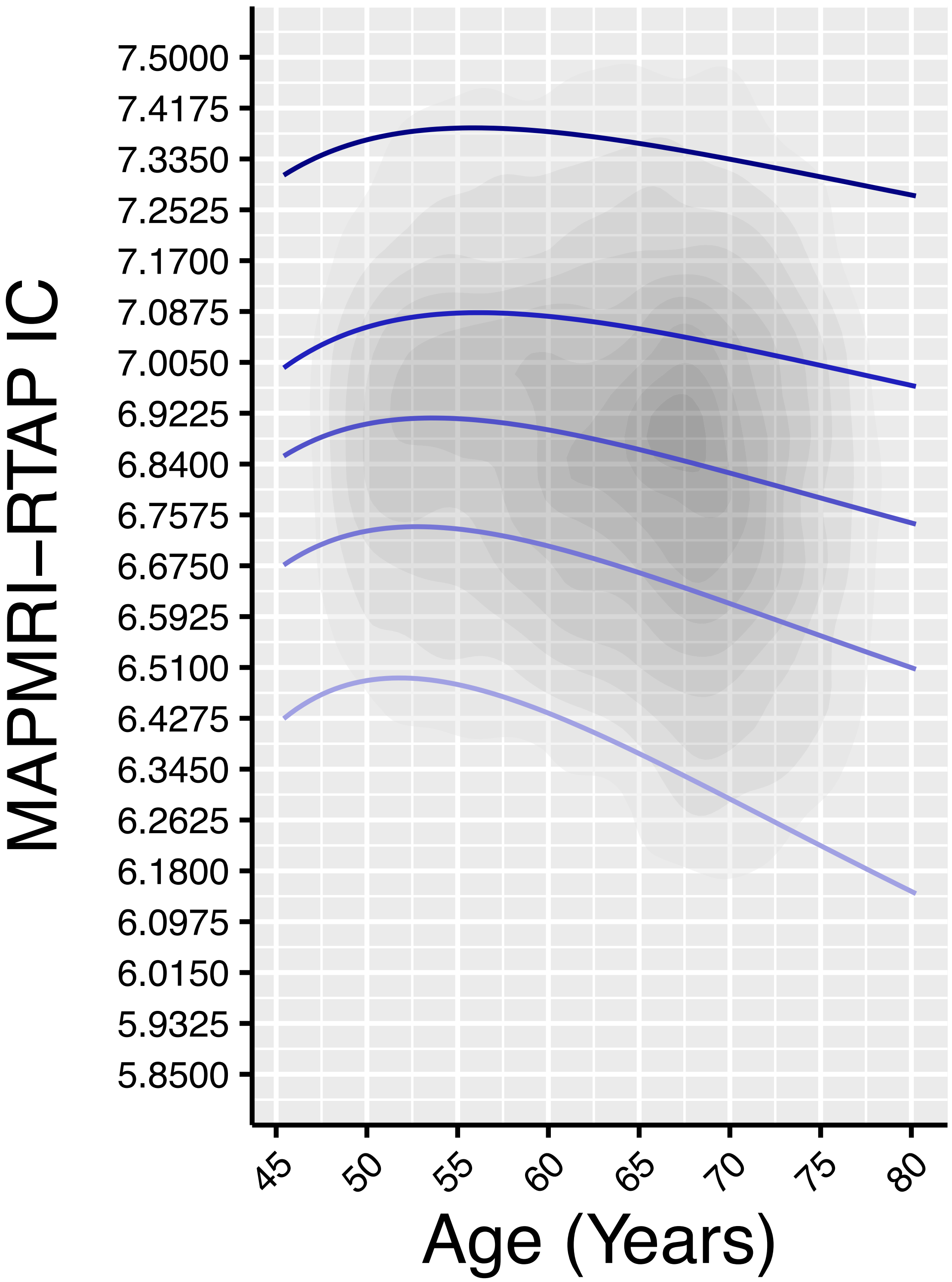

Figure S240. Full size normative centile reference curves calculated for the internal capsule tract for MAPMRI-RTAP in males. Solid colored lines, ordered from lightest to darkest, indicate the following centiles: 5th, 25th, 50th, 75th, 95th. Gray overlay reflects kernel density (darker=greater degree of data point overlap). IC = internal capsule.

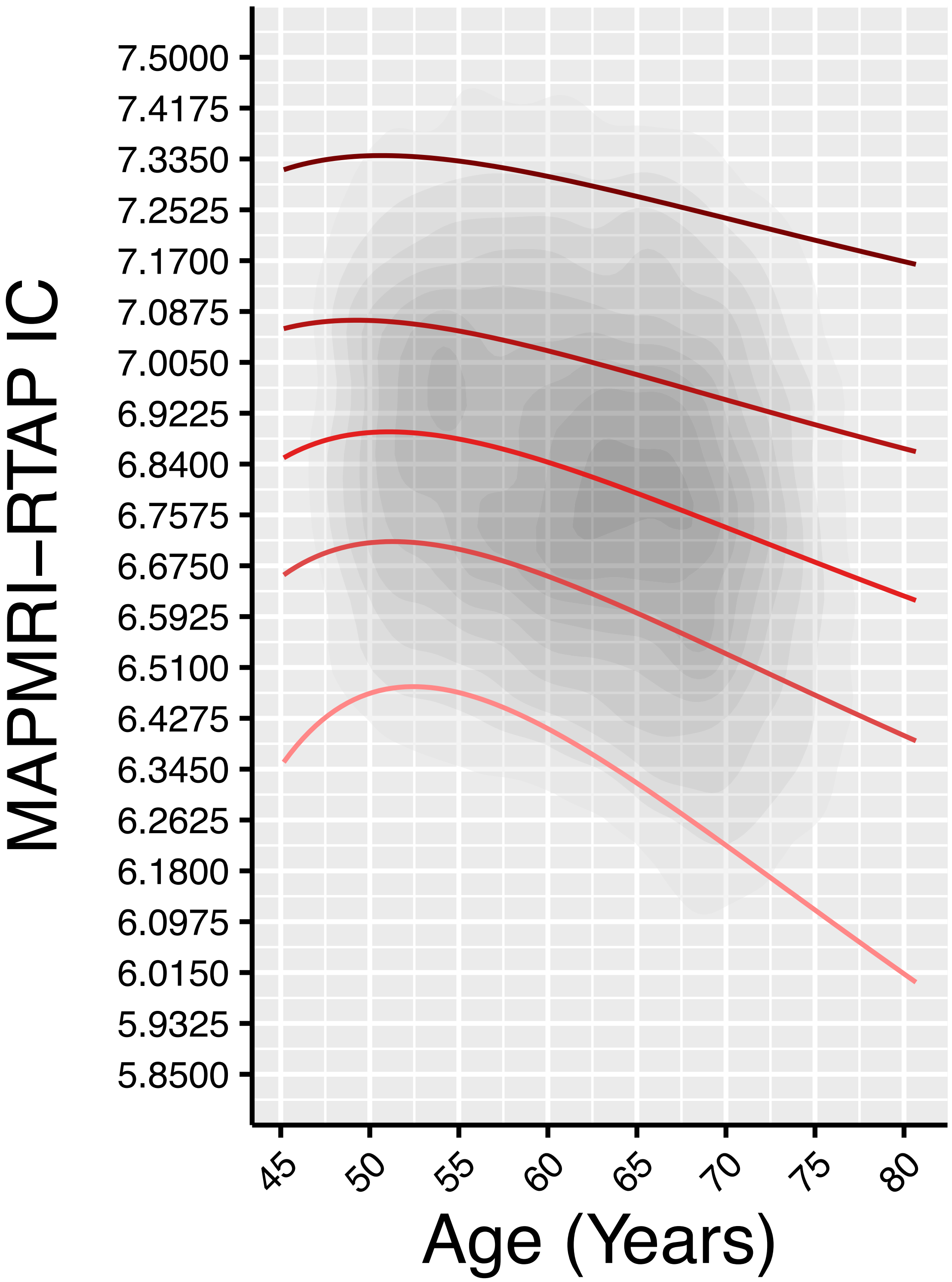

Figure S241. Full size normative centile reference curves calculated for the internal capsule tract for MAPMRI-RTAP in females. Solid colored lines, ordered from lightest to darkest, indicate the following centiles: 5th, 25th, 50th, 75th, 95th. Gray overlay reflects kernel density (darker=greater degree of data point overlap). IC = internal capsule.

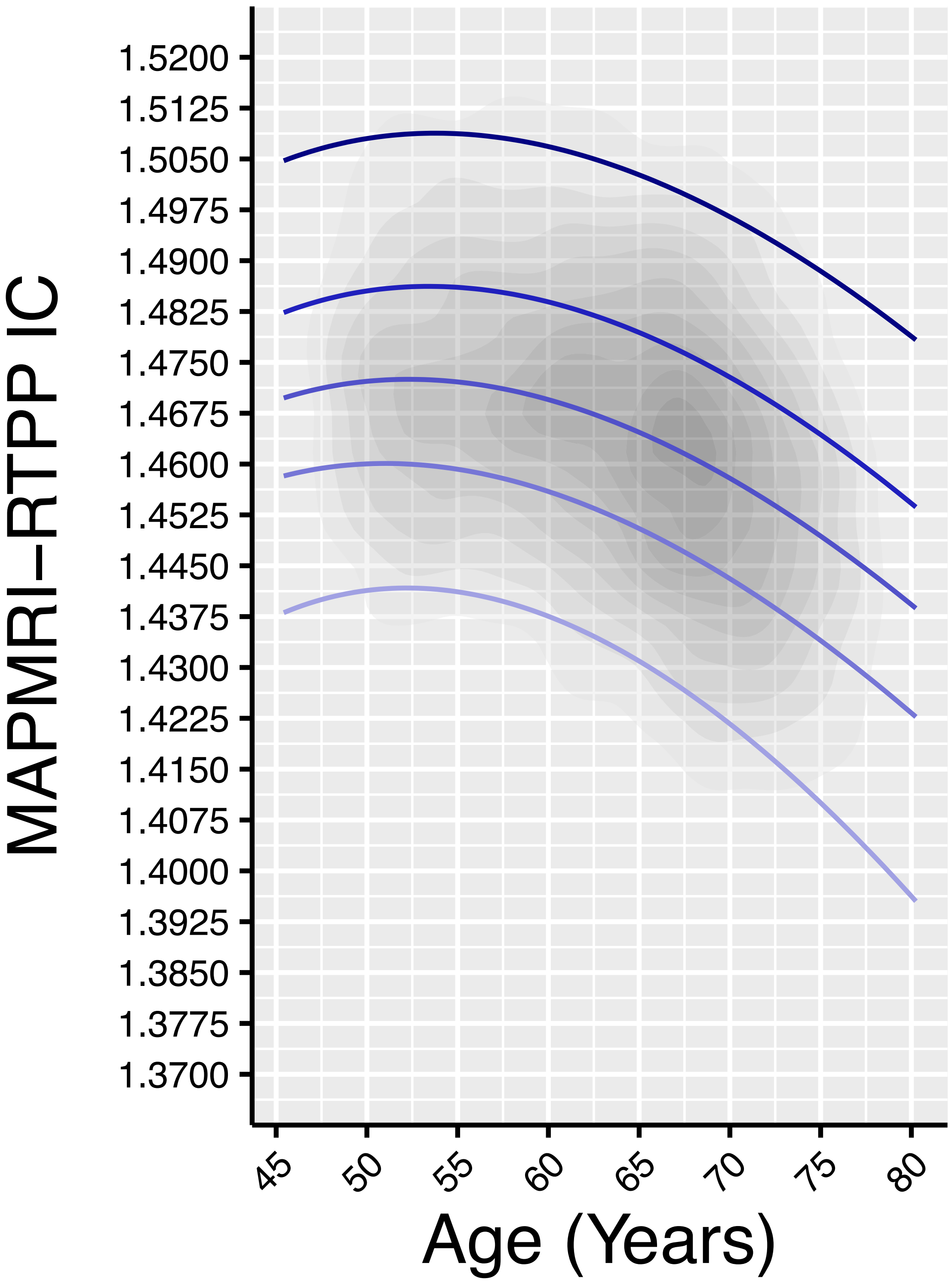

Figure S242. Full size normative centile reference curves calculated for the internal capsule tract for MAPMRI-RTPP in males. Solid colored lines, ordered from lightest to darkest, indicate the following centiles: 5th, 25th, 50th, 75th, 95th. Gray overlay reflects kernel density (darker=greater degree of data point overlap). IC = internal capsule.

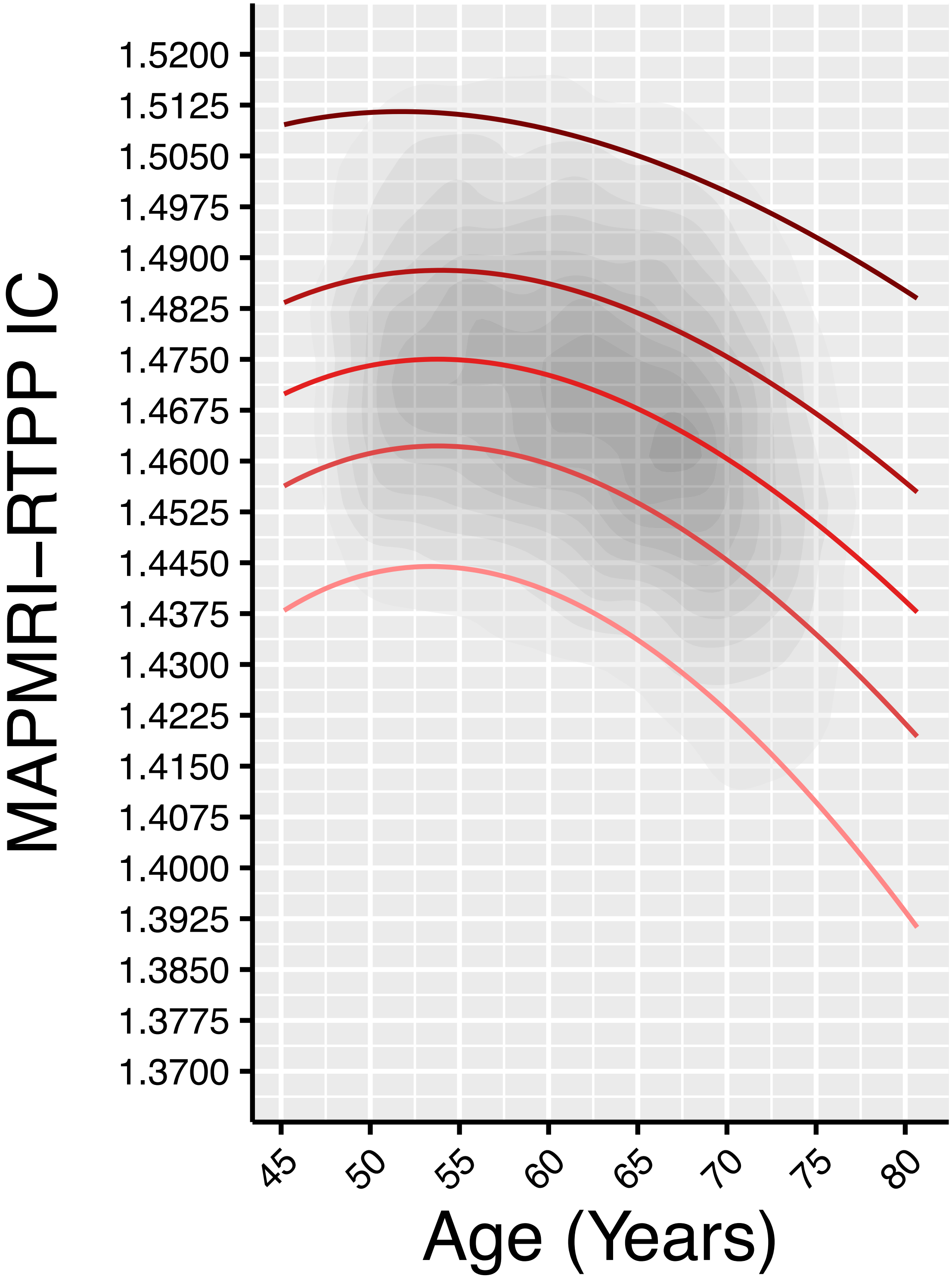

Figure S243. Full size normative centile reference curves calculated for the internal capsule tract for MAPMRI-RTPP in females. Solid colored lines, ordered from lightest to darkest, indicate the following centiles: 5th, 25th, 50th, 75th, 95th. Gray overlay reflects kernel density (darker=greater degree of data point overlap). IC = internal capsule.

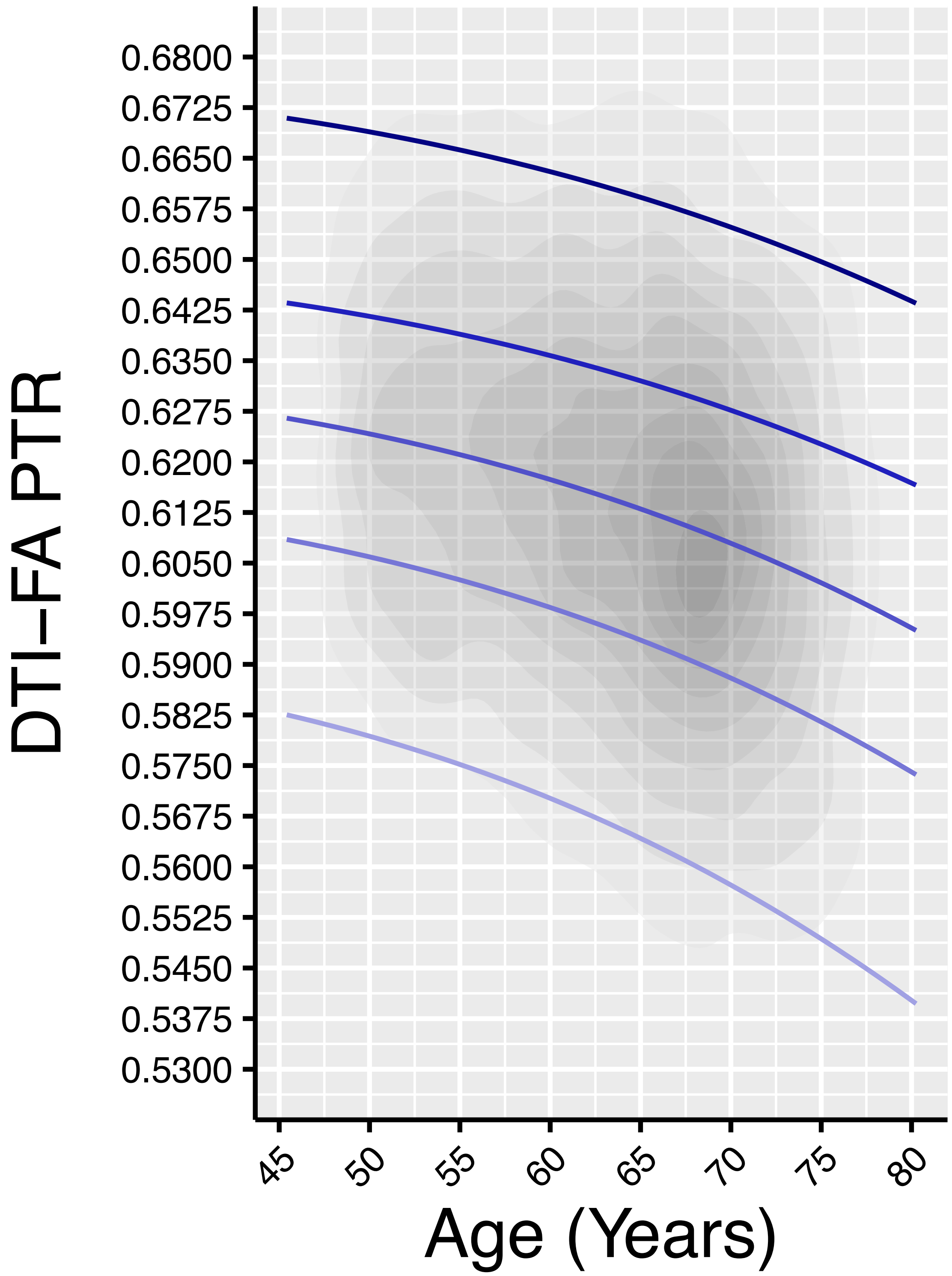

Figure S244. Full size normative centile reference curves calculated for the posterior thalamic radiation tract for DTI-FA in males. Solid colored lines, ordered from lightest to darkest, indicate the following centiles: 5th, 25th, 50th, 75th, 95th. Gray overlay reflects kernel density (darker=greater degree of data point overlap). PTR = posterior thalamic radiation.

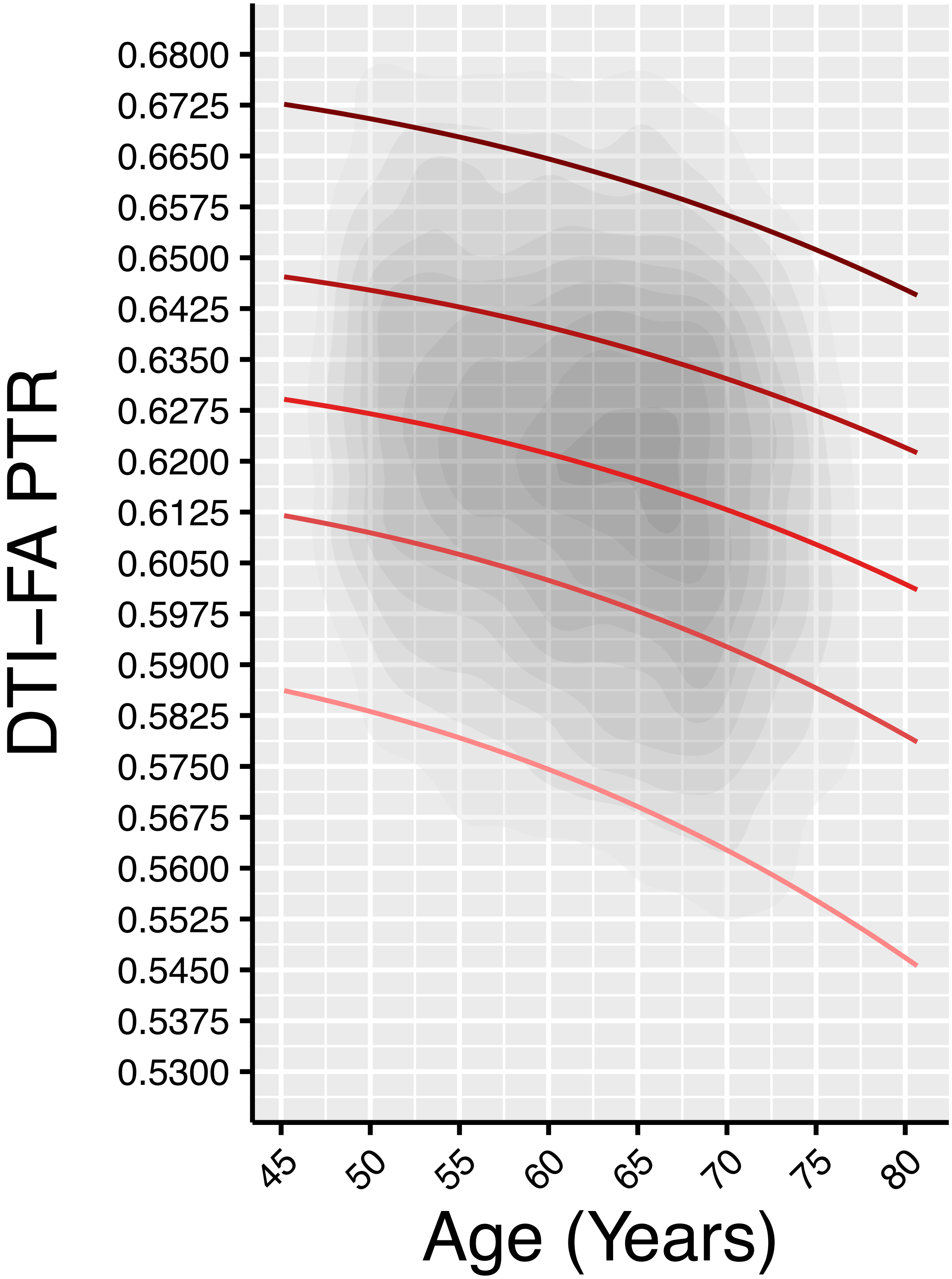

Figure S245. Full size normative centile reference curves calculated for the posterior thalamic radiation tract for DTI-FA in females. Solid colored lines, ordered from lightest to darkest, indicate the following centiles: 5th, 25th, 50th, 75th, 95th. Gray overlay reflects kernel density (darker=greater degree of data point overlap). PTR = posterior thalamic radiation.

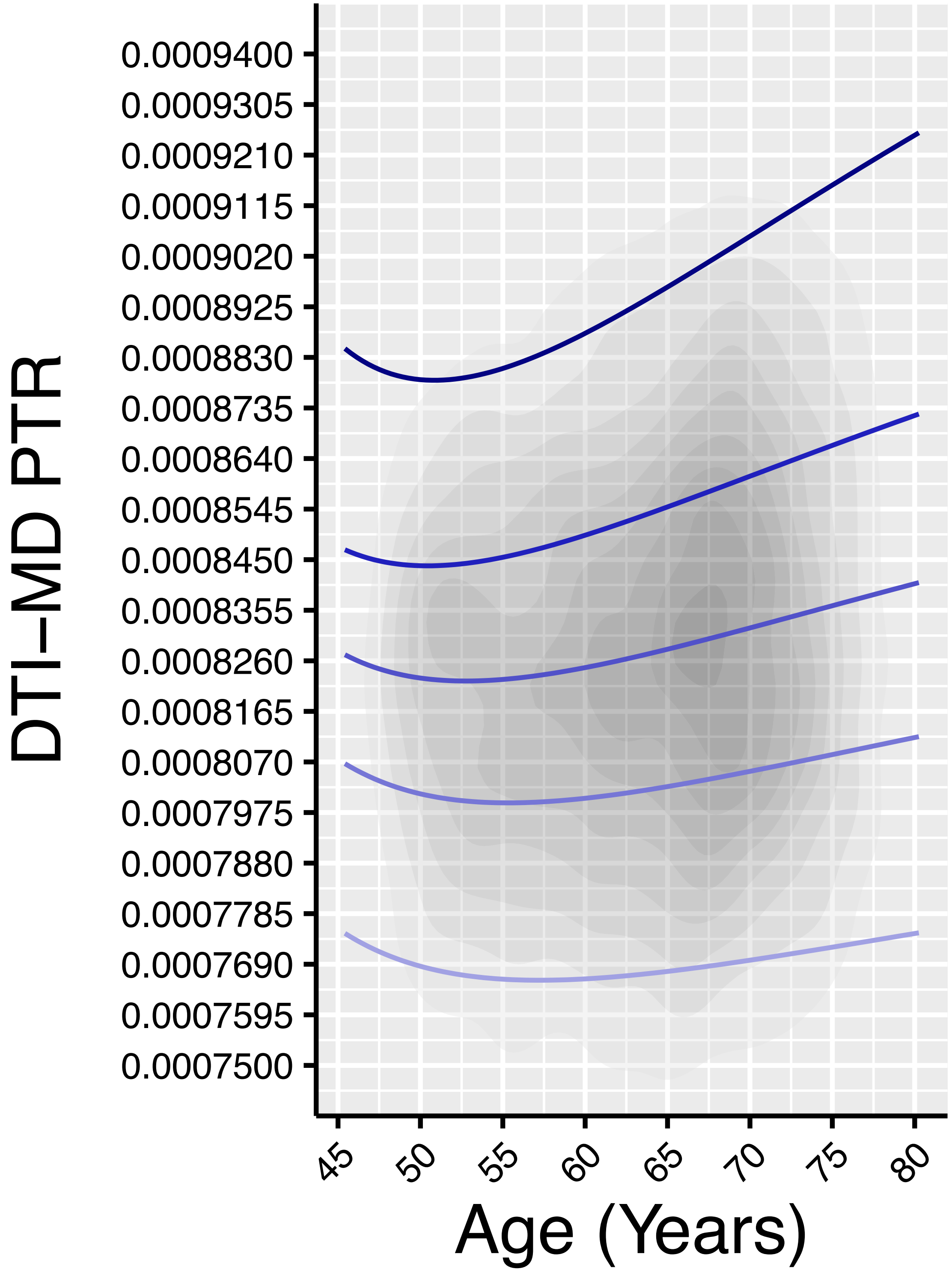

Figure S246. Full size normative centile reference curves calculated for the posterior thalamic radiation tract for DTI-MD in males. Solid colored lines, ordered from lightest to darkest, indicate the following centiles: 5th, 25th, 50th, 75th, 95th. Gray overlay reflects kernel density (darker=greater degree of data point overlap). PTR = posterior thalamic radiation.

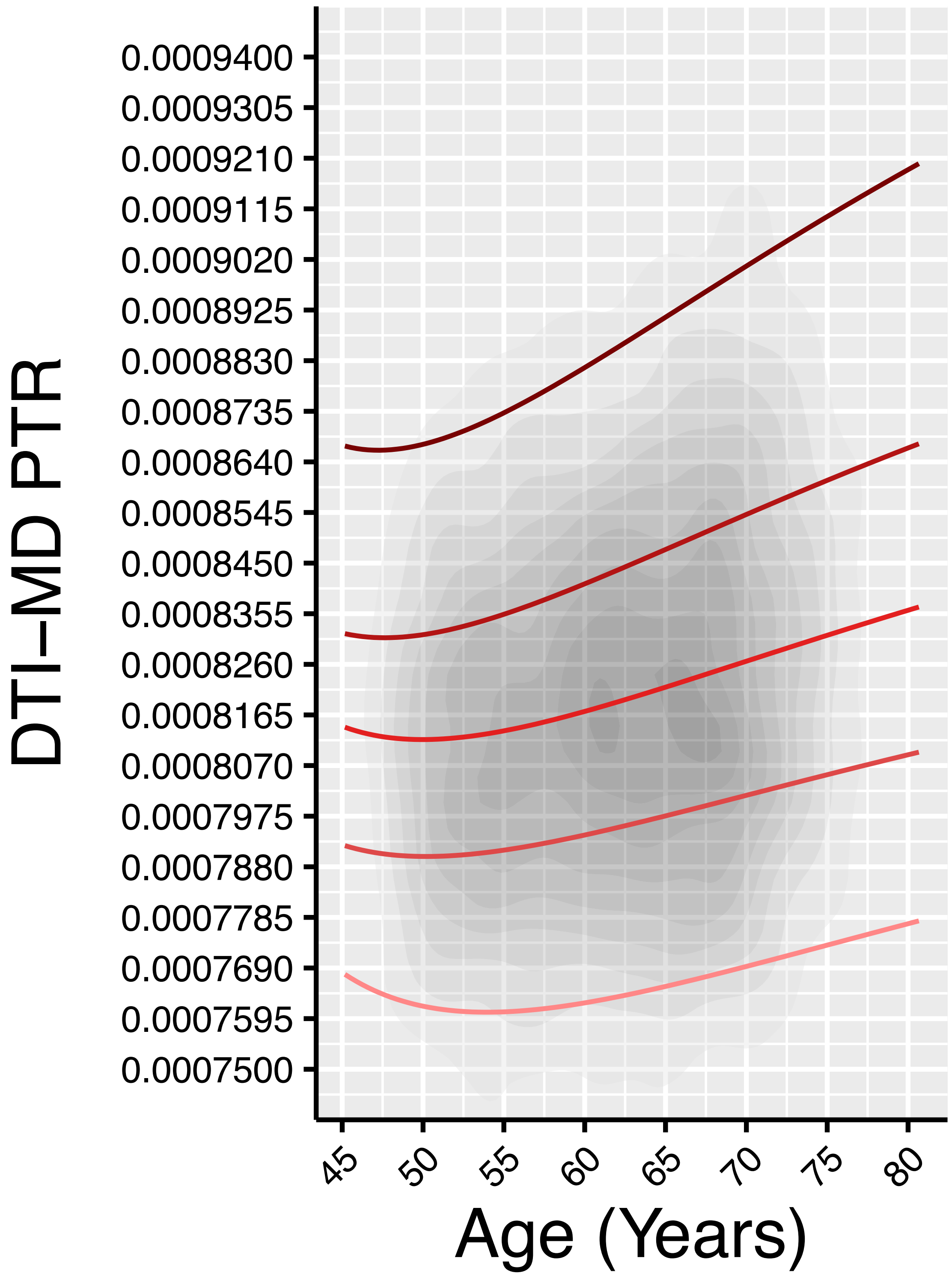

Figure S247. Full size normative centile reference curves calculated for the posterior thalamic radiation tract for DTI-MD in females. Solid colored lines, ordered from lightest to darkest, indicate the following centiles: 5th, 25th, 50th, 75th, 95th. Gray overlay reflects kernel density (darker=greater degree of data point overlap). PTR = posterior thalamic radiation.

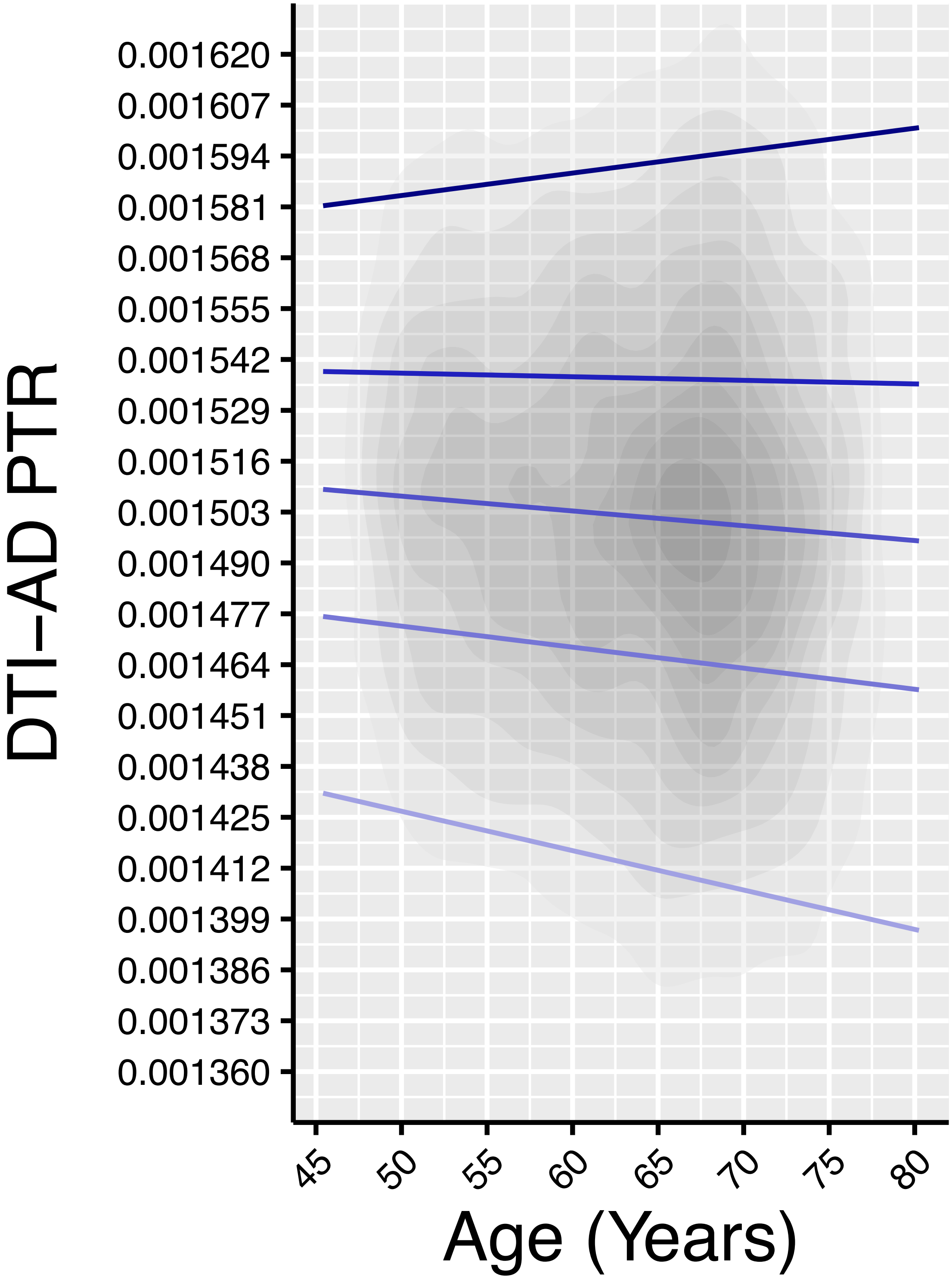

Figure S248. Full size normative centile reference curves calculated for the posterior thalamic radiation tract for DTI-AD in males. Solid colored lines, ordered from lightest to darkest, indicate the following centiles: 5th, 25th, 50th, 75th, 95th. Gray overlay reflects kernel density (darker=greater degree of data point overlap). PTR = posterior thalamic radiation.

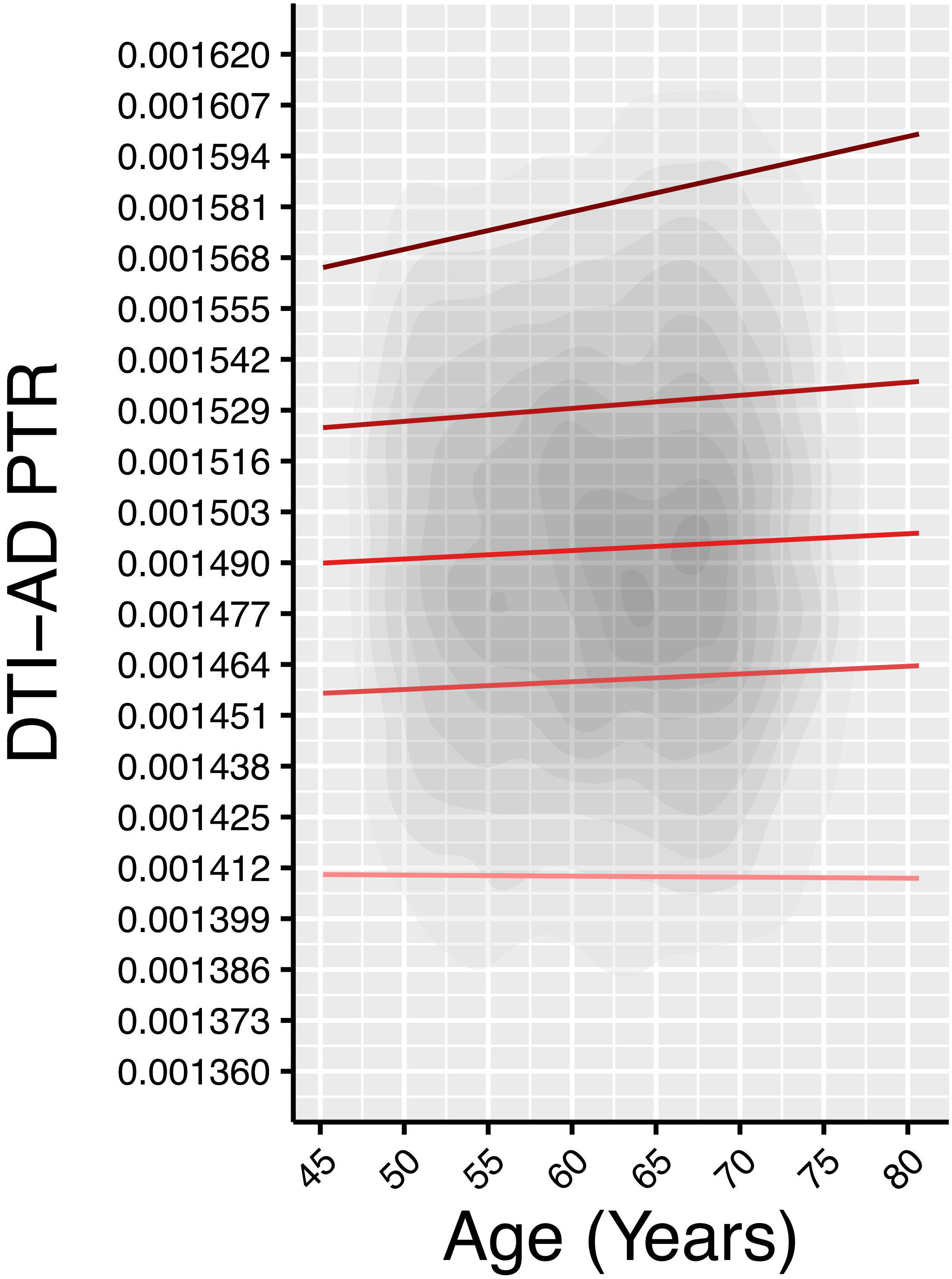

Figure S249. Full size normative centile reference curves calculated for the posterior thalamic radiation tract for DTI-AD in females. Solid colored lines, ordered from lightest to darkest, indicate the following centiles: 5th, 25th, 50th, 75th, 95th. Gray overlay reflects kernel density (darker=greater degree of data point overlap). PTR = posterior thalamic radiation.

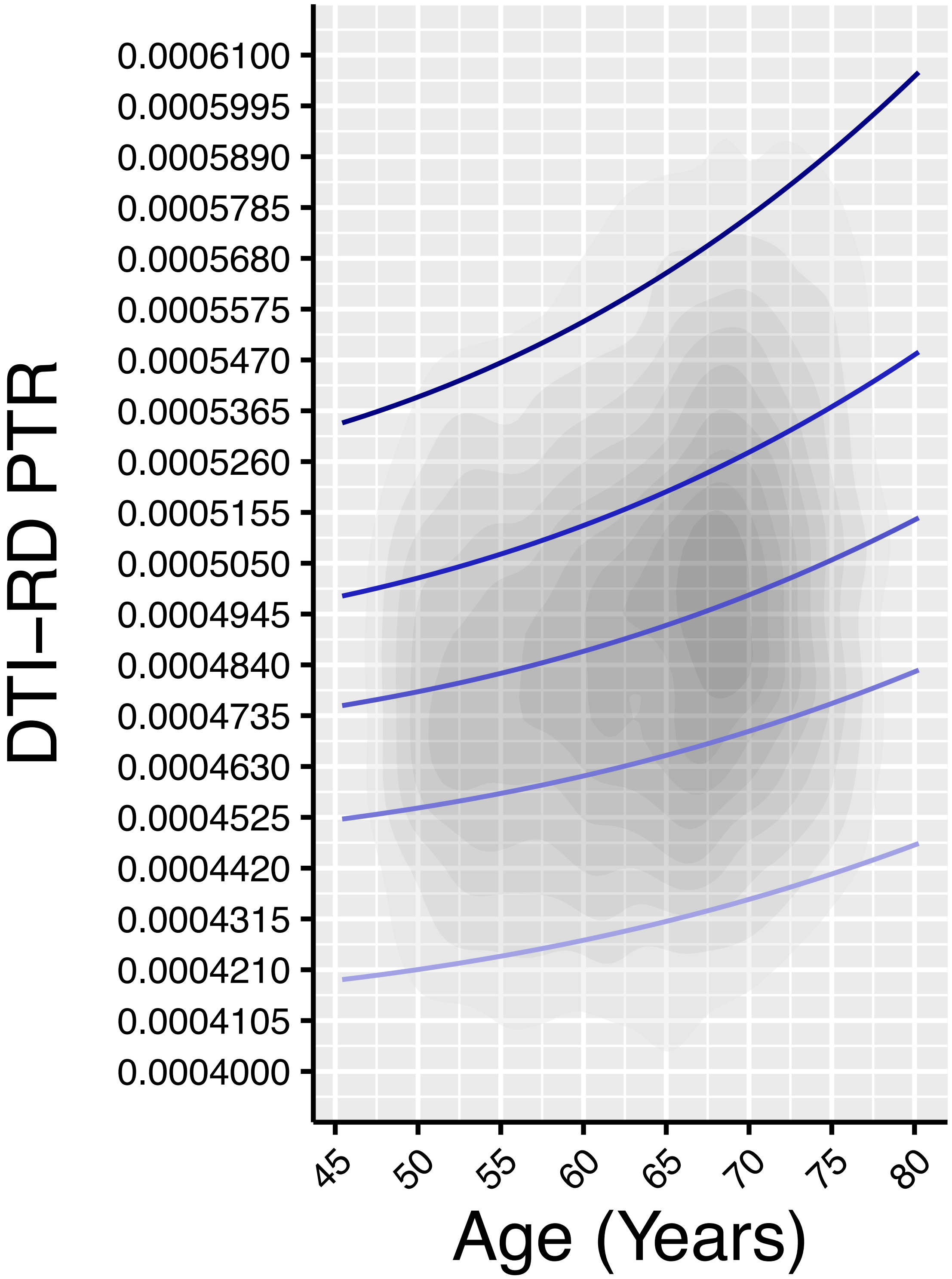

Figure S250. Full size normative centile reference curves calculated for the posterior thalamic radiation tract for DTI-RD in males. Solid colored lines, ordered from lightest to darkest, indicate the following centiles: 5th, 25th, 50th, 75th, 95th. Gray overlay reflects kernel density (darker=greater degree of data point overlap). PTR = posterior thalamic radiation.

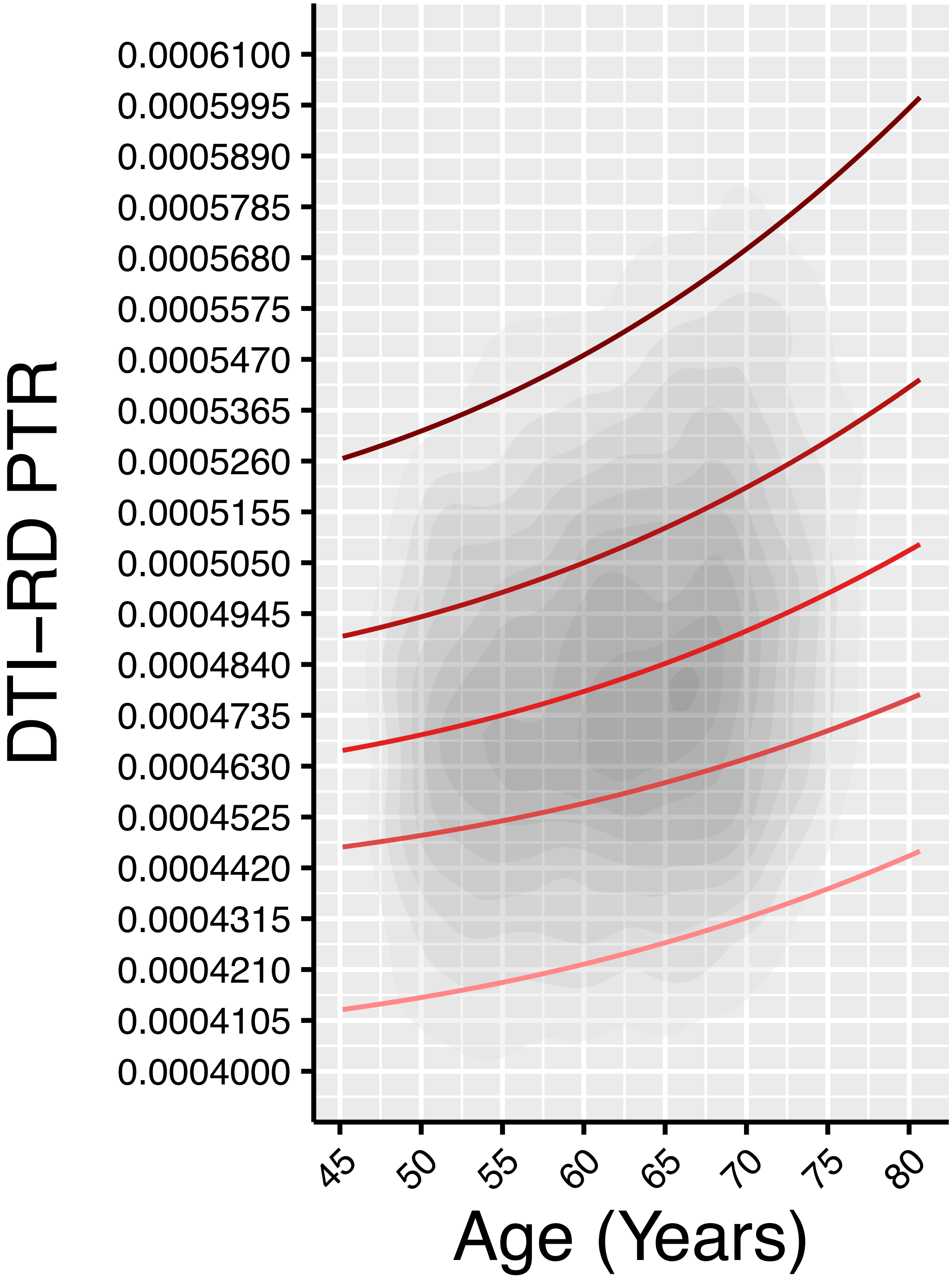

Figure S251. Full size normative centile reference curves calculated for the posterior thalamic radiation tract for DTI-RD in females. Solid colored lines, ordered from lightest to darkest, indicate the following centiles: 5th, 25th, 50th, 75th, 95th. Gray overlay reflects kernel density (darker=greater degree of data point overlap). PTR = posterior thalamic radiation.

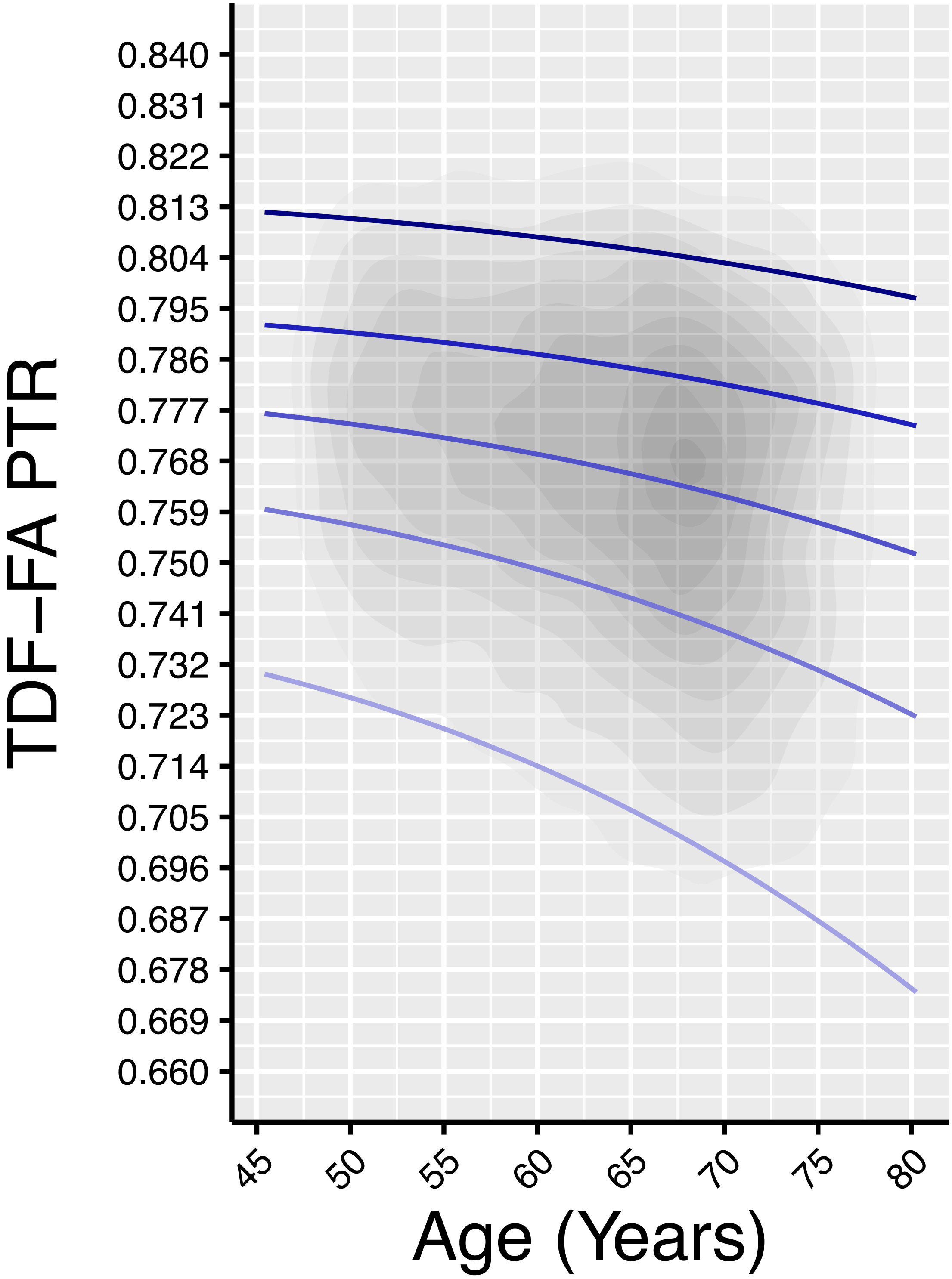

Figure S252. Full size normative centile reference curves calculated for the posterior thalamic radiation tract for TDF-FA in males. Solid colored lines, ordered from lightest to darkest, indicate the following centiles: 5th, 25th, 50th, 75th, 95th. Gray overlay reflects kernel density (darker=greater degree of data point overlap). PTR = posterior thalamic radiation.

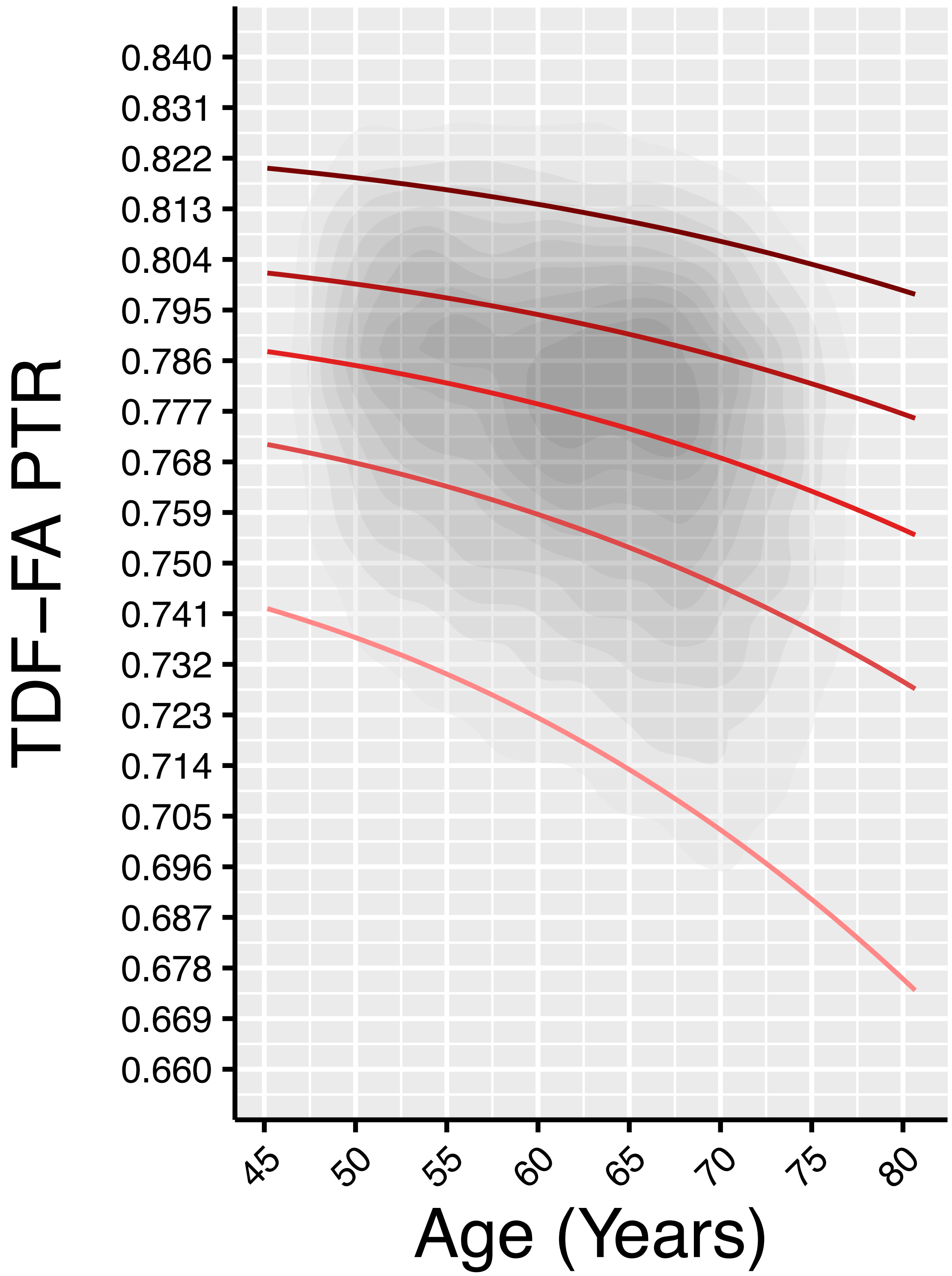

Figure S253. Full size normative centile reference curves calculated for the posterior thalamic radiation tract for TDF-FA in females. Solid colored lines, ordered from lightest to darkest, indicate the following centiles: 5th, 25th, 50th, 75th, 95th. Gray overlay reflects kernel density (darker=greater degree of data point overlap). PTR = posterior thalamic radiation.

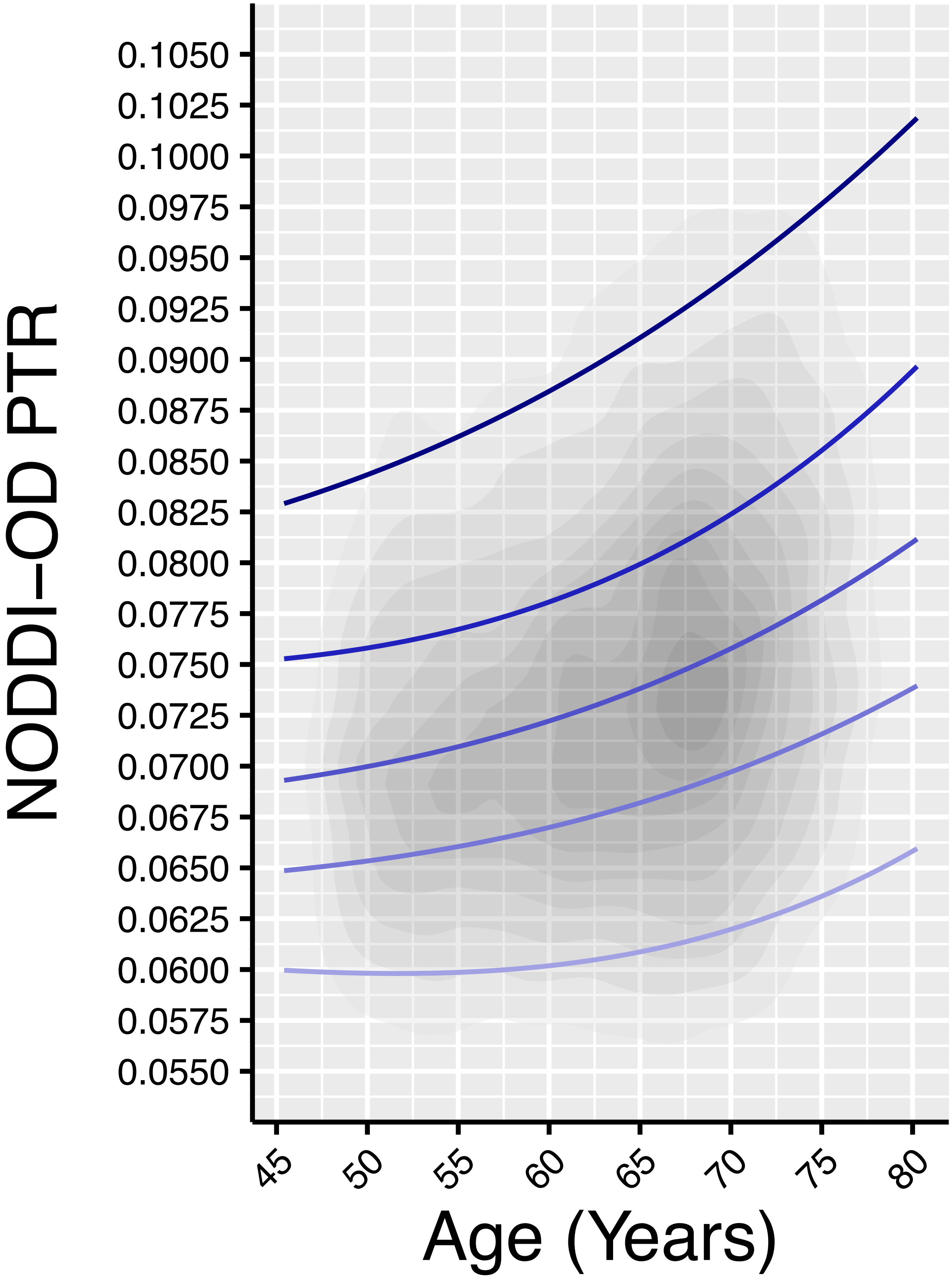

Figure S254. Full size normative centile reference curves calculated for the posterior thalamic radiation tract for NODDI-OD in males. Solid colored lines, ordered from lightest to darkest, indicate the following centiles: 5th, 25th, 50th, 75th, 95th. Gray overlay reflects kernel density (darker=greater degree of data point overlap). PTR = posterior thalamic radiation.

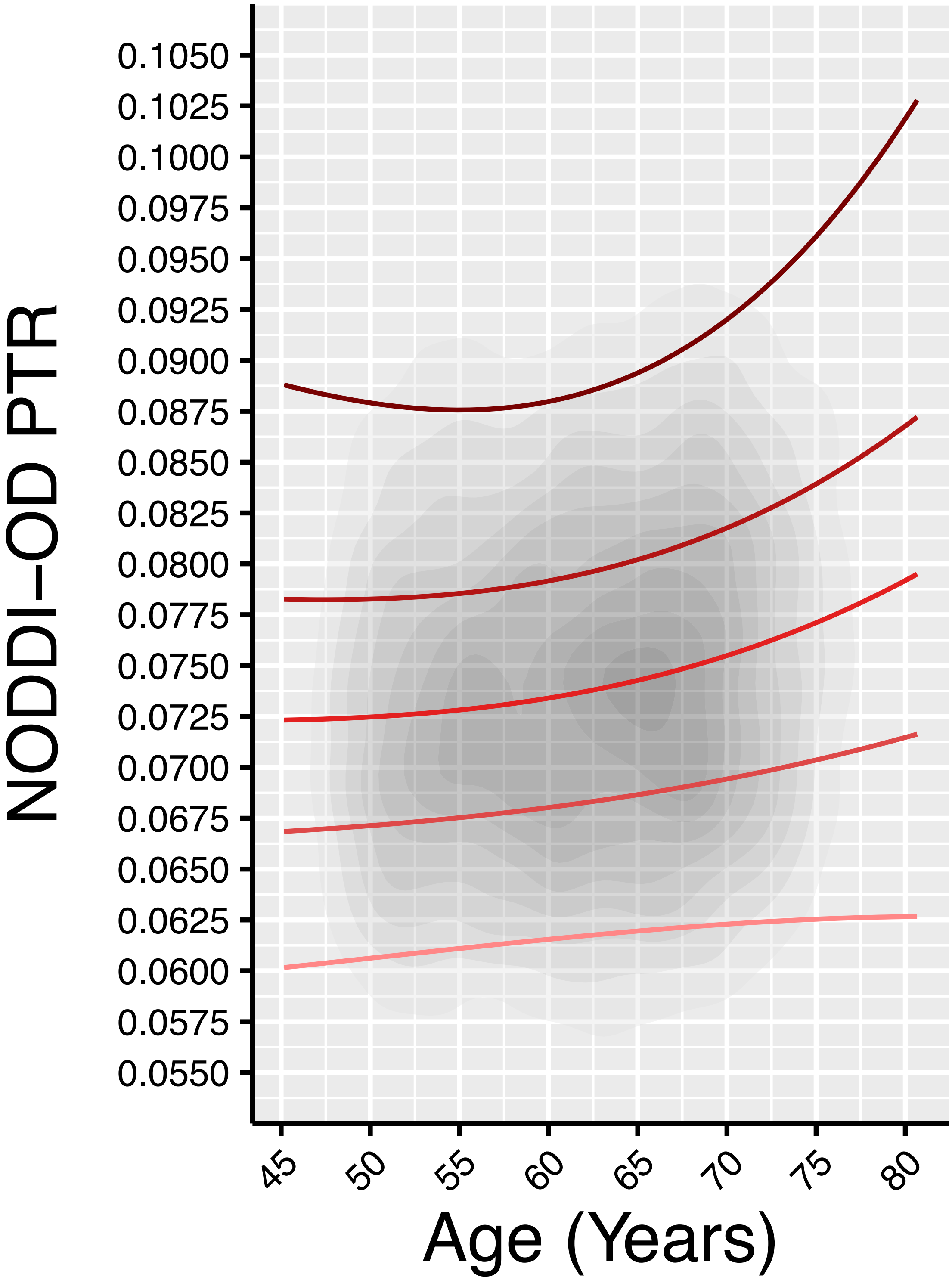

Figure S255. Full size normative centile reference curves calculated for the posterior thalamic radiation tract for NODDI-OD in females. Solid colored lines, ordered from lightest to darkest, indicate the following centiles: 5th, 25th, 50th, 75th, 95th. Gray overlay reflects kernel density (darker=greater degree of data point overlap). PTR = posterior thalamic radiation.

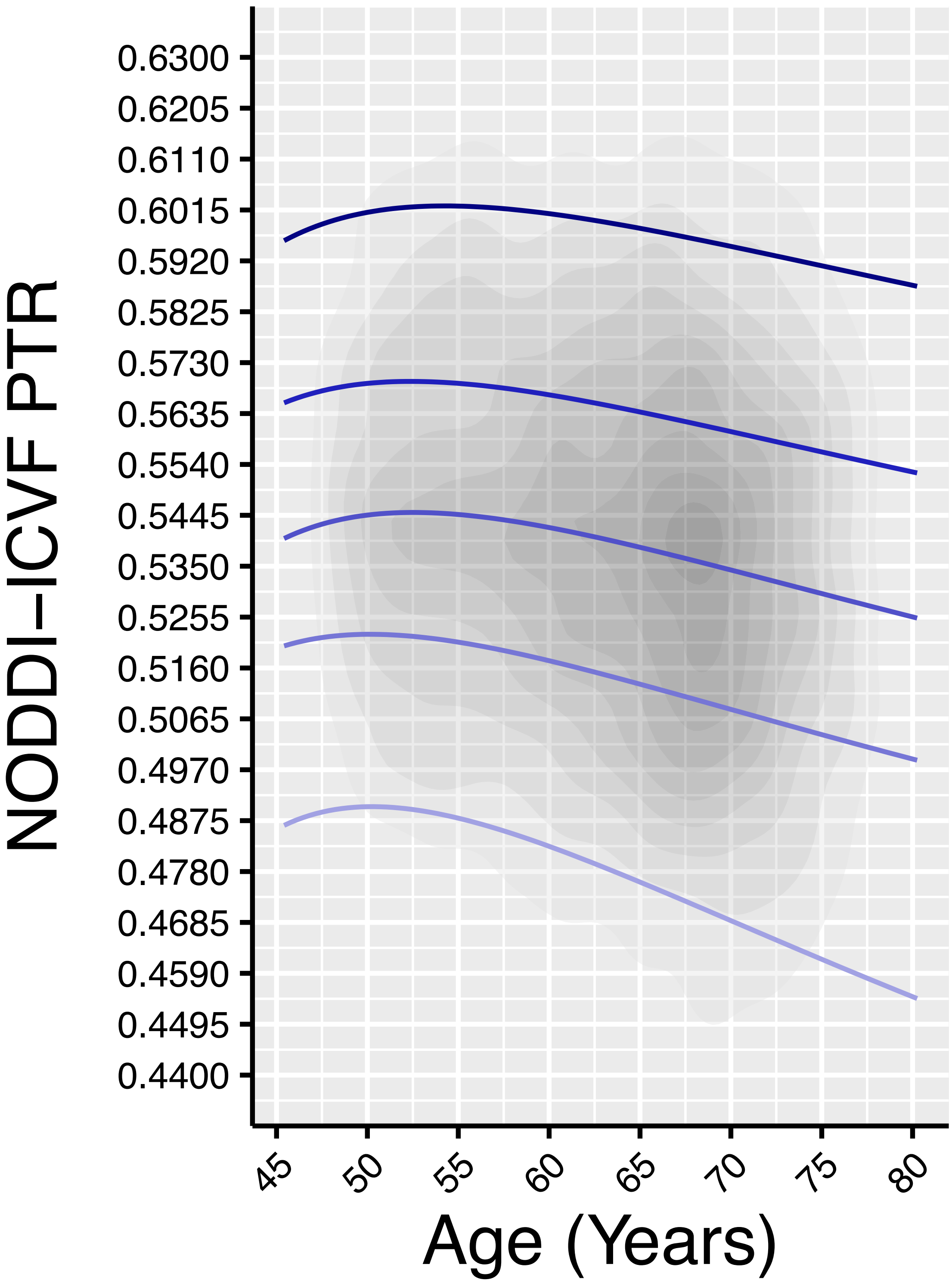

Figure S256. Full size normative centile reference curves calculated for the posterior thalamic radiation tract for NODDI-ICVF in males. Solid colored lines, ordered from lightest to darkest, indicate the following centiles: 5th, 25th, 50th, 75th, 95th. Gray overlay reflects kernel density (darker=greater degree of data point overlap). PTR = posterior thalamic radiation.

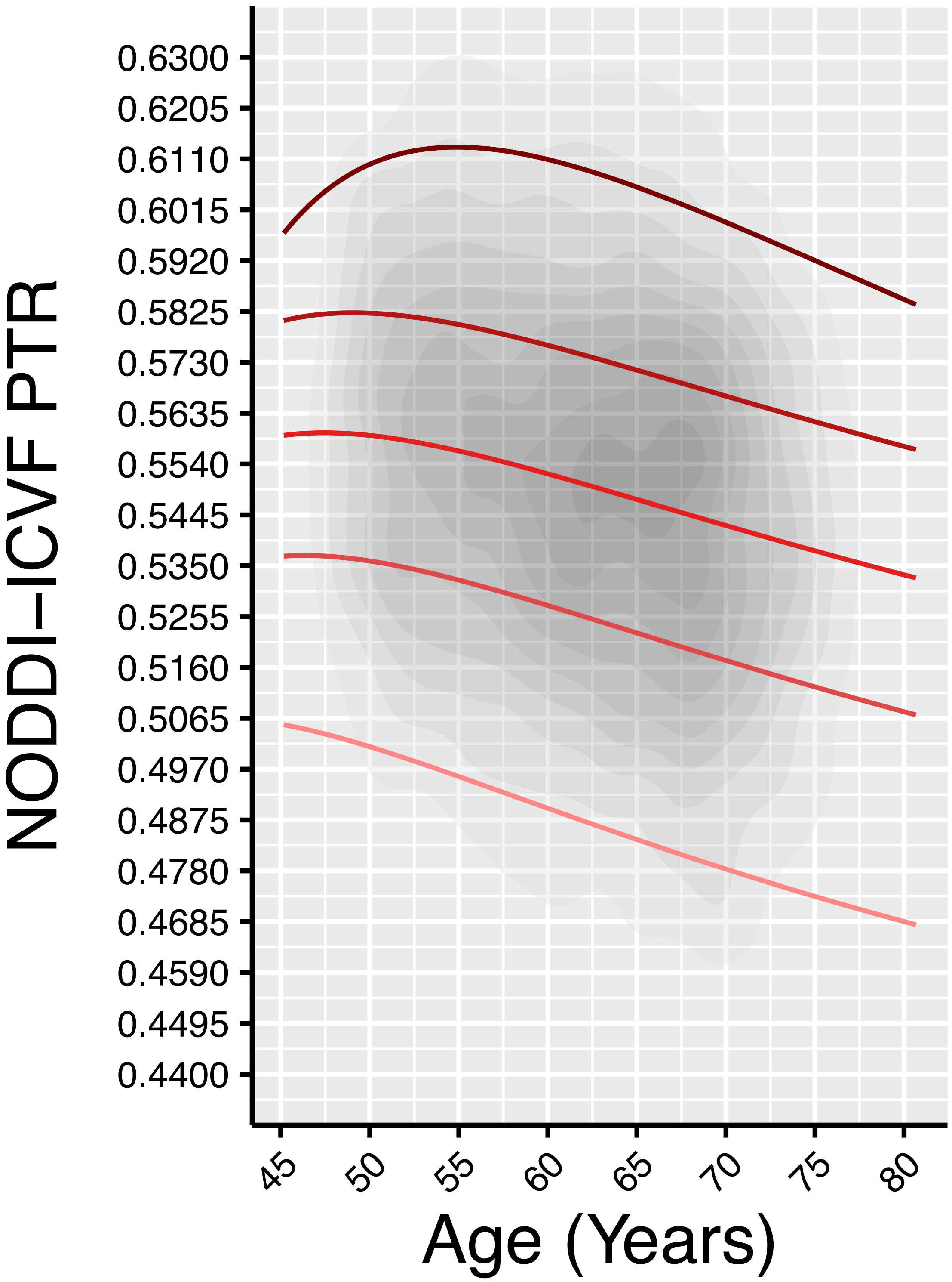

Figure S257. Full size normative centile reference curves calculated for the posterior thalamic radiation tract for NODDI-ICVF in females. Solid colored lines, ordered from lightest to darkest, indicate the following centiles: 5th, 25th, 50th, 75th, 95th. Gray overlay reflects kernel density (darker=greater degree of data point overlap). PTR = posterior thalamic radiation.

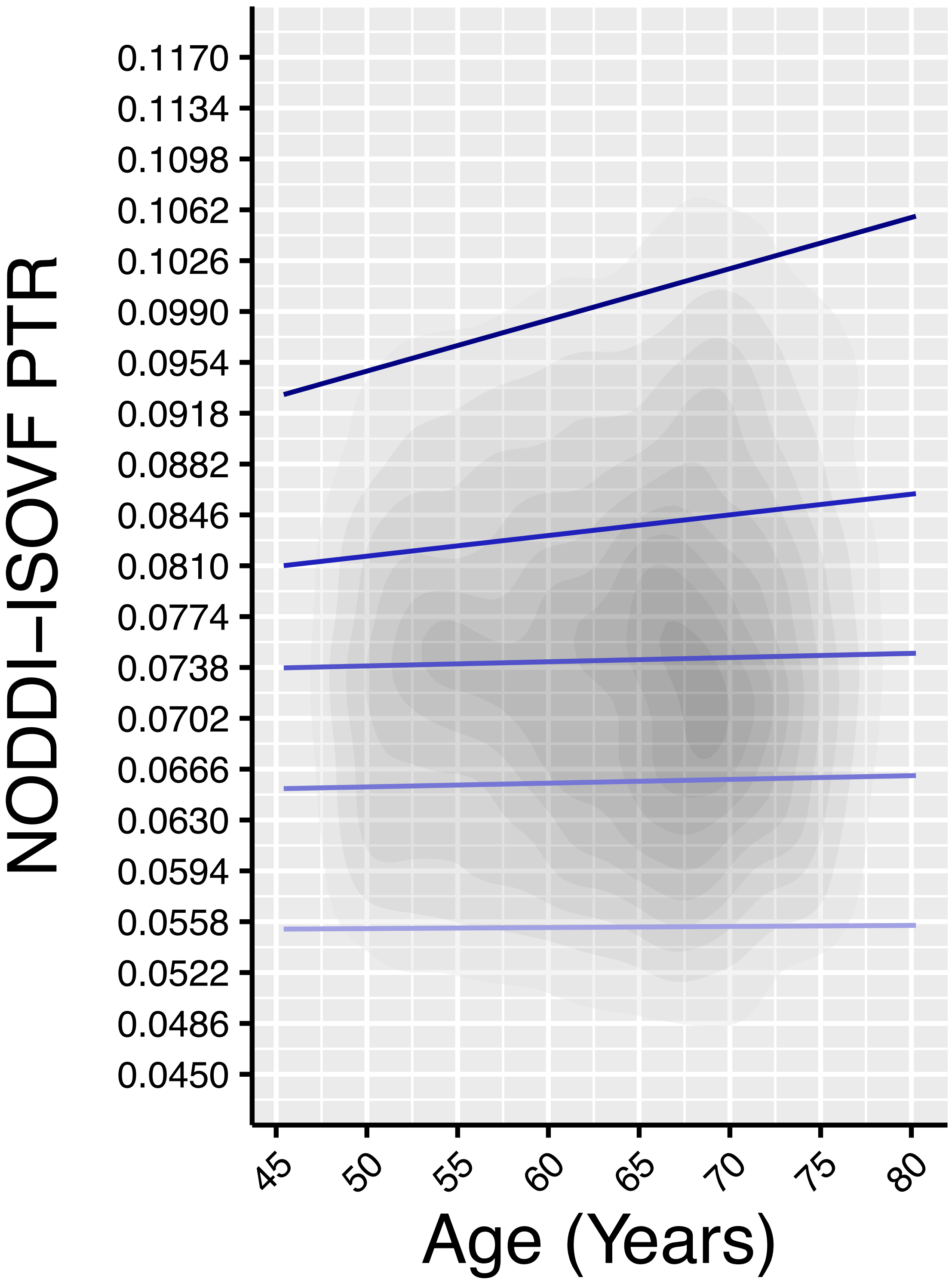

Figure S258. Full size normative centile reference curves calculated for the posterior thalamic radiation tract for NODDI-ISOVF in males. Solid colored lines, ordered from lightest to darkest, indicate the following centiles: 5th, 25th, 50th, 75th, 95th. Gray overlay reflects kernel density (darker=greater degree of data point overlap). PTR = posterior thalamic radiation.

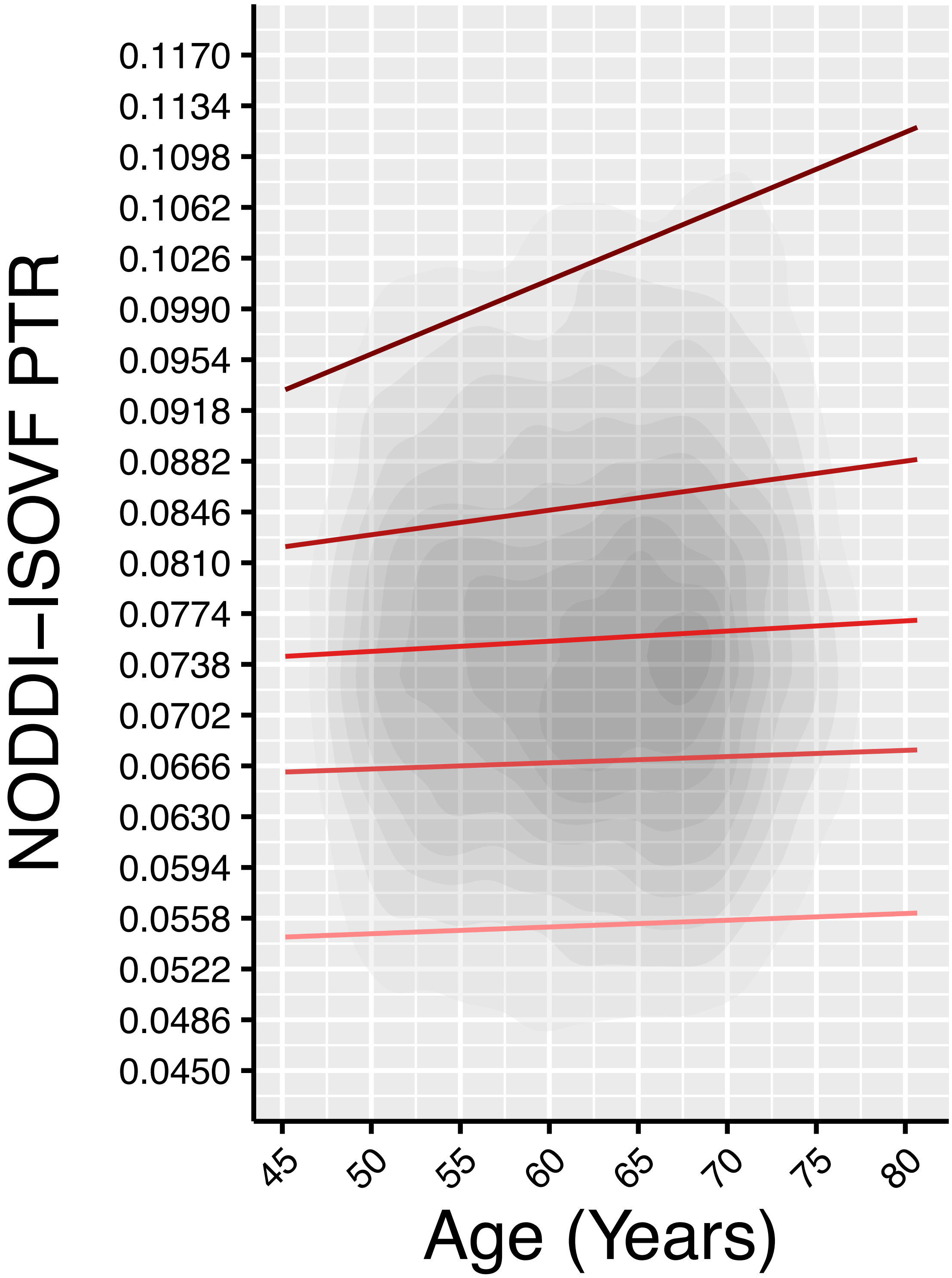

Figure S259. Full size normative centile reference curves calculated for the posterior thalamic radiation tract for NODDI-ISOVF in females. Solid colored lines, ordered from lightest to darkest, indicate the following centiles: 5th, 25th, 50th, 75th, 95th. Gray overlay reflects kernel density (darker=greater degree of data point overlap). PTR = posterior thalamic radiation.

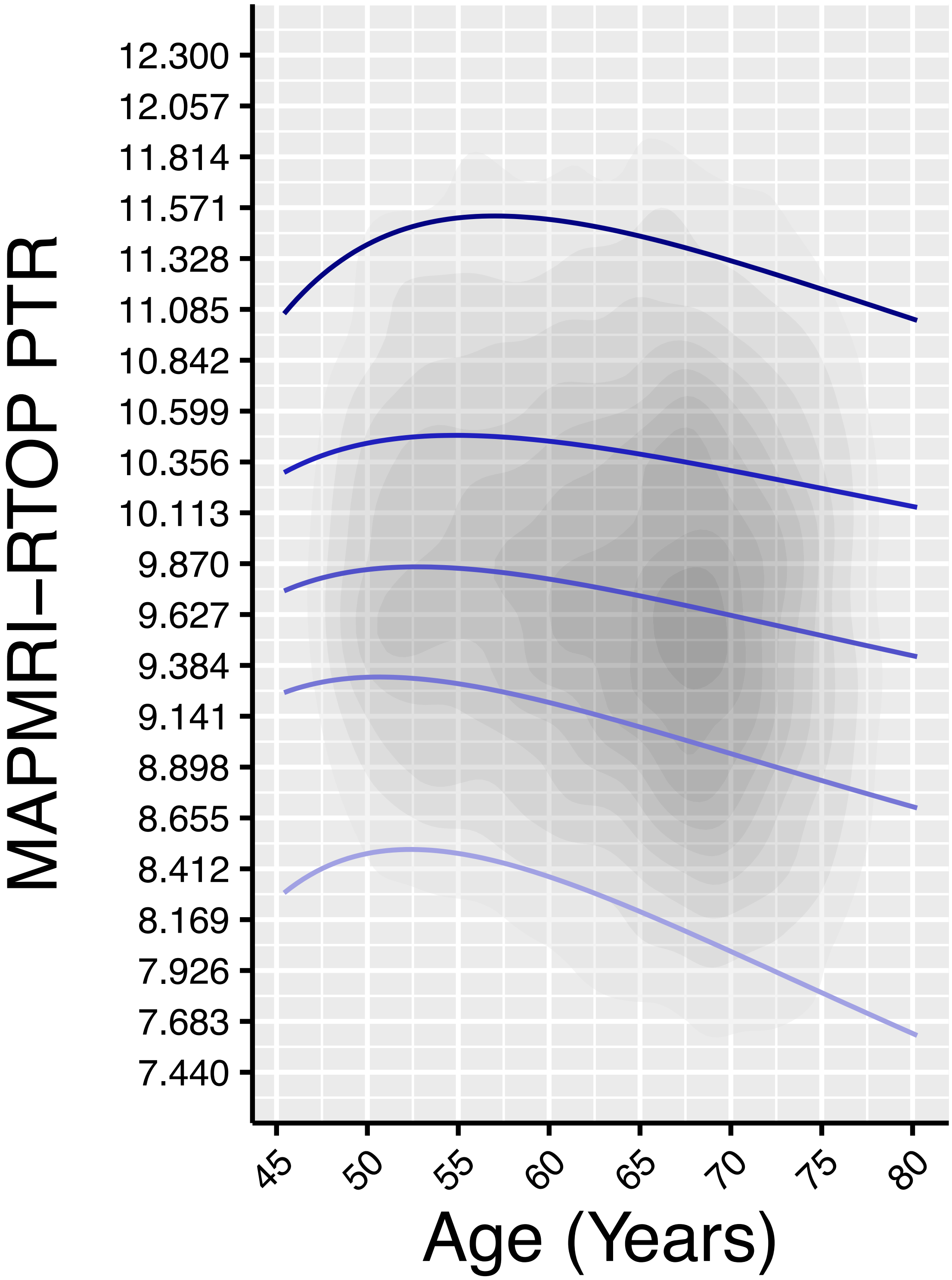

Figure S260. Full size normative centile reference curves calculated for the posterior thalamic radiation tract for MAPMRI-RTOP in males. Solid colored lines, ordered from lightest to darkest, indicate the following centiles: 5th, 25th, 50th, 75th, 95th. Gray overlay reflects kernel density (darker=greater degree of data point overlap). PTR = posterior thalamic radiation.

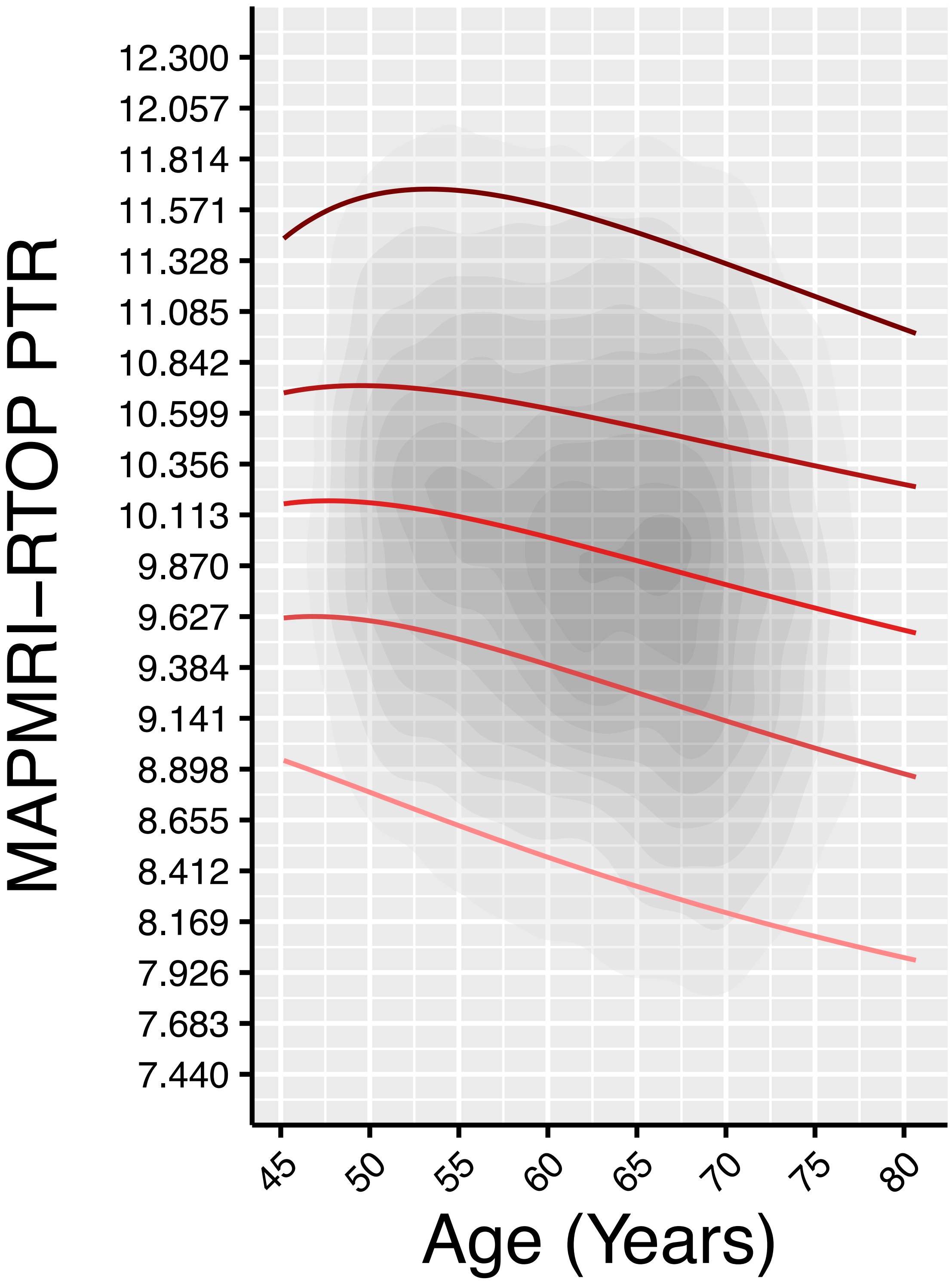

Figure S261. Full size normative centile reference curves calculated for the posterior thalamic radiation tract for MAPMRI-RTOP in females. Solid colored lines, ordered from lightest to darkest, indicate the following centiles: 5th, 25th, 50th, 75th, 95th. Gray overlay reflects kernel density (darker=greater degree of data point overlap). PTR = posterior thalamic radiation.

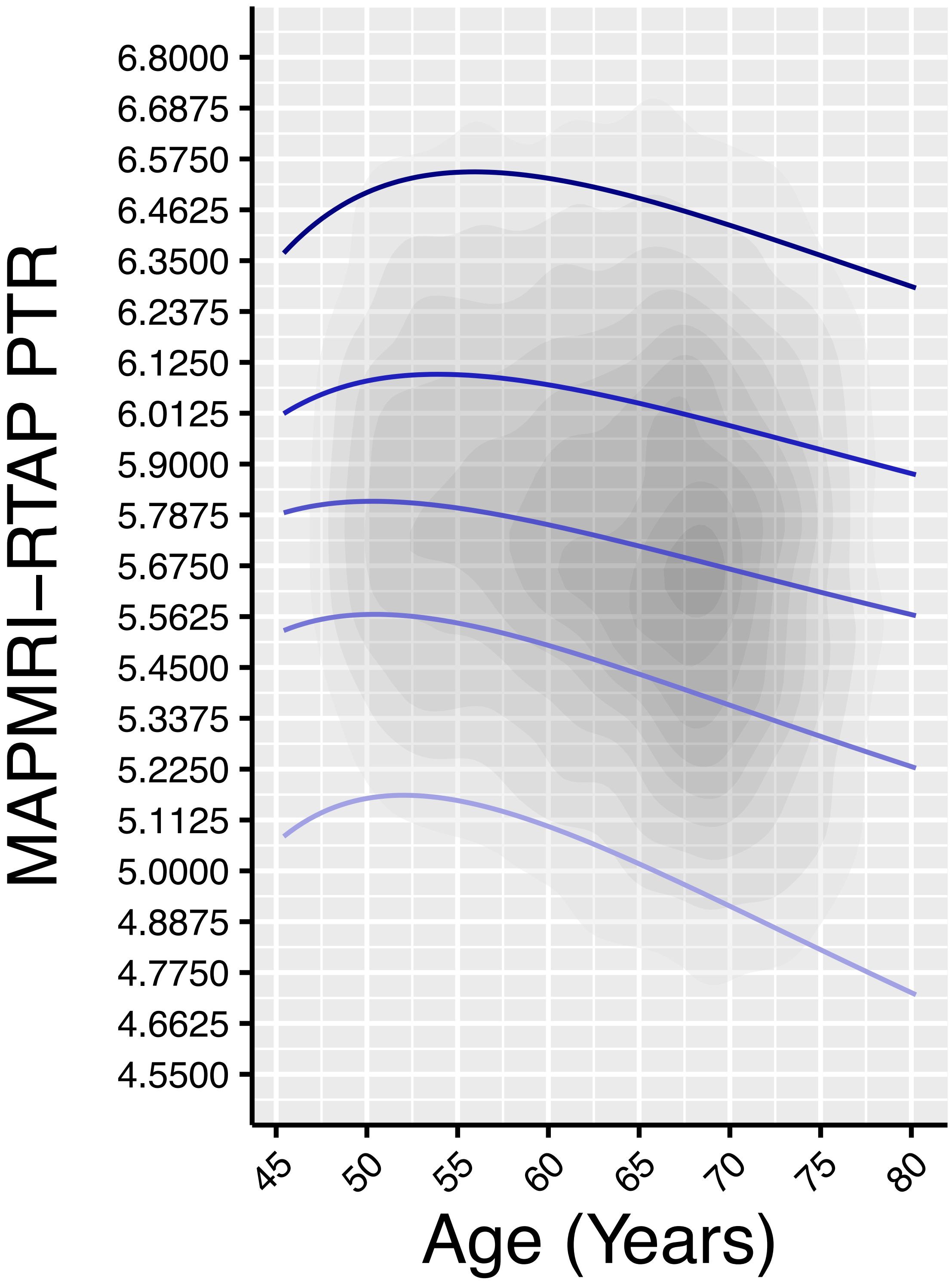

Figure S262. Full size normative centile reference curves calculated for the posterior thalamic radiation tract for MAPMRI-RTAP in males. Solid colored lines, ordered from lightest to darkest, indicate the following centiles: 5th, 25th, 50th, 75th, 95th. Gray overlay reflects kernel density (darker=greater degree of data point overlap). PTR = posterior thalamic radiation.

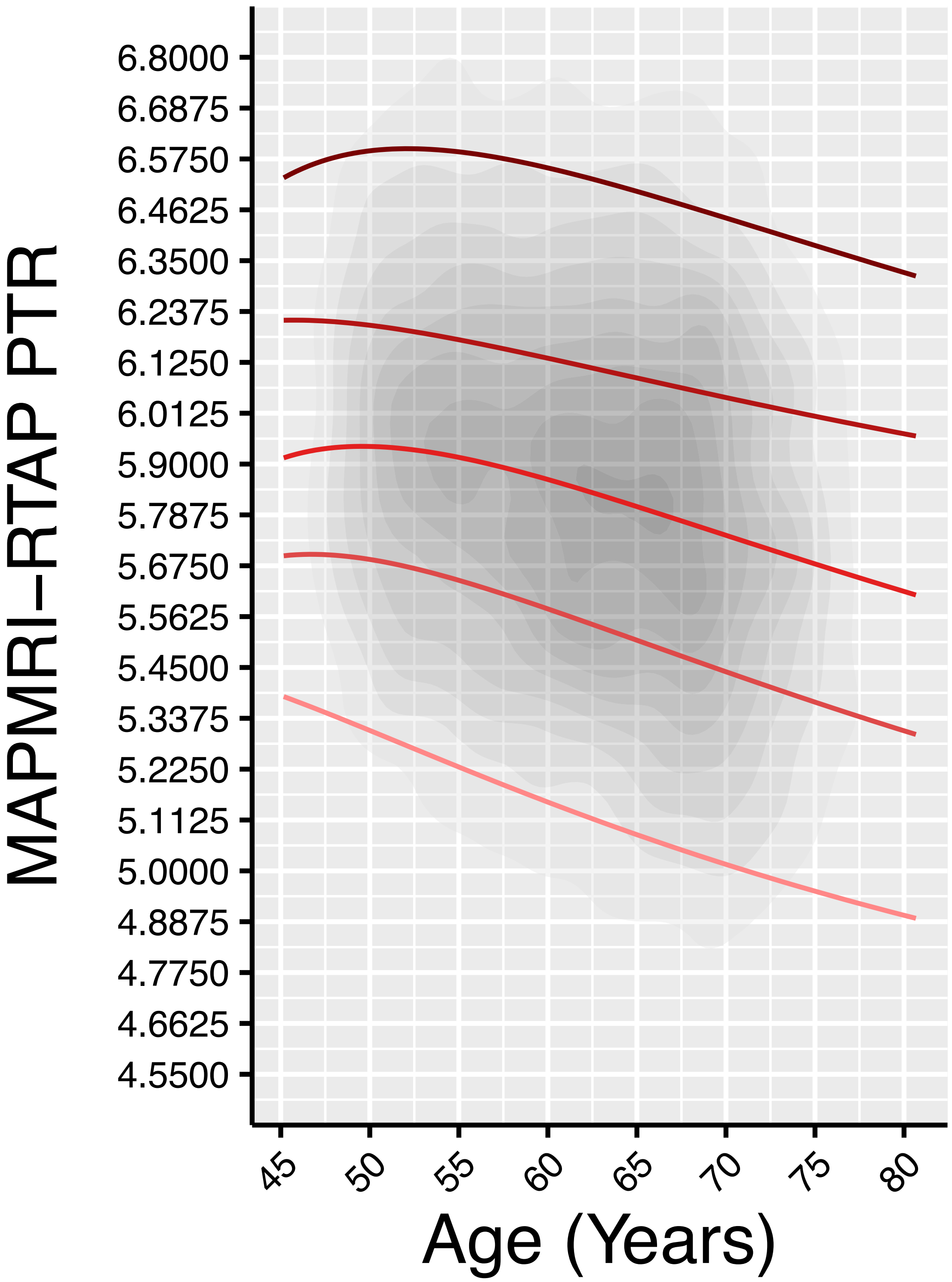

Figure S263. Full size normative centile reference curves calculated for the posterior thalamic radiation tract for MAPMRI-RTAP in females. Solid colored lines, ordered from lightest to darkest, indicate the following centiles: 5th, 25th, 50th, 75th, 95th. Gray overlay reflects kernel density (darker=greater degree of data point overlap). PTR = posterior thalamic radiation.

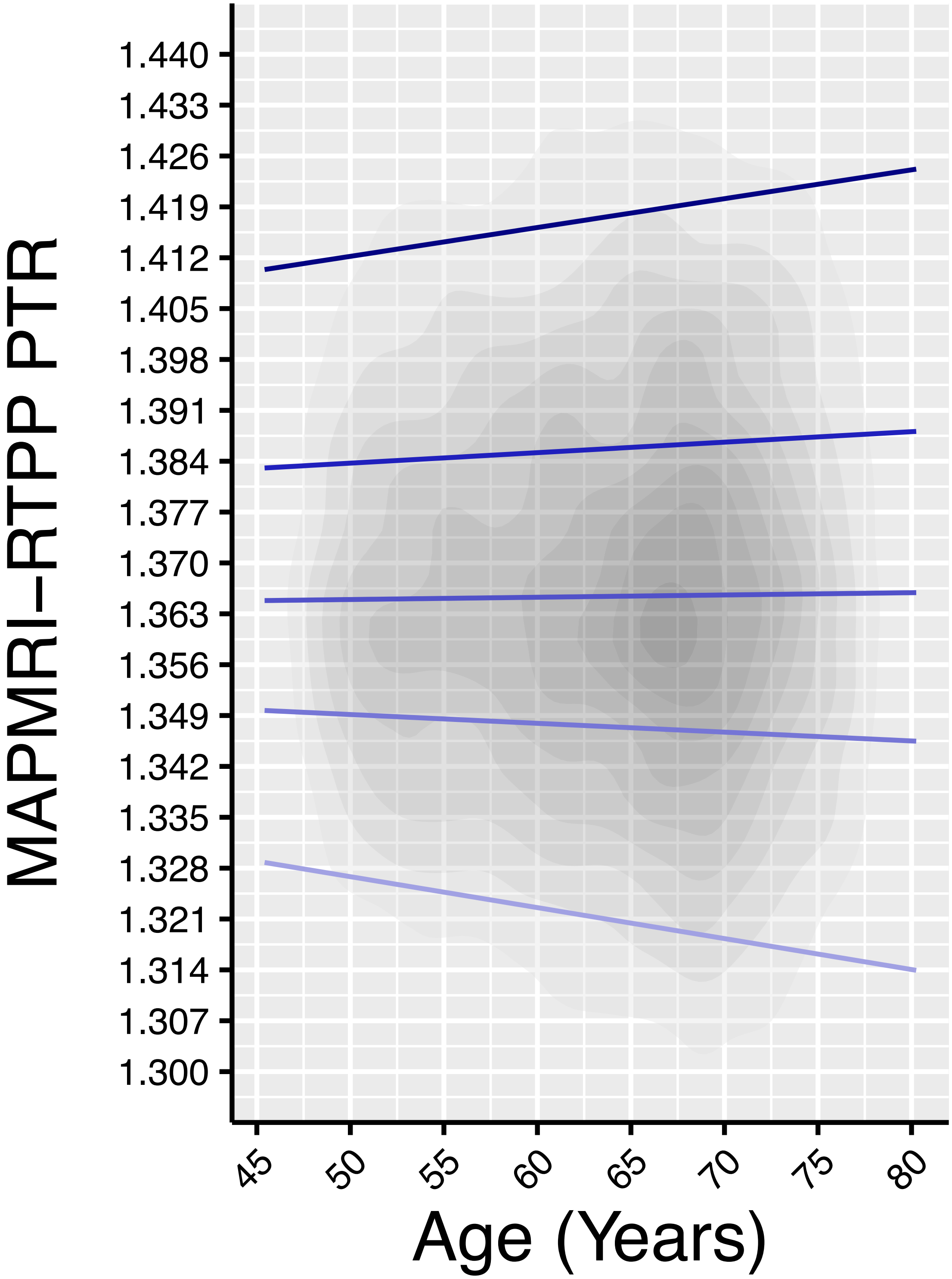

Figure S264. Full size normative centile reference curves calculated for the posterior thalamic radiation tract for MAPMRI-RTPP in males. Solid colored lines, ordered from lightest to darkest, indicate the following centiles: 5th, 25th, 50th, 75th, 95th. Gray overlay reflects kernel density (darker=greater degree of data point overlap). PTR = posterior thalamic radiation.

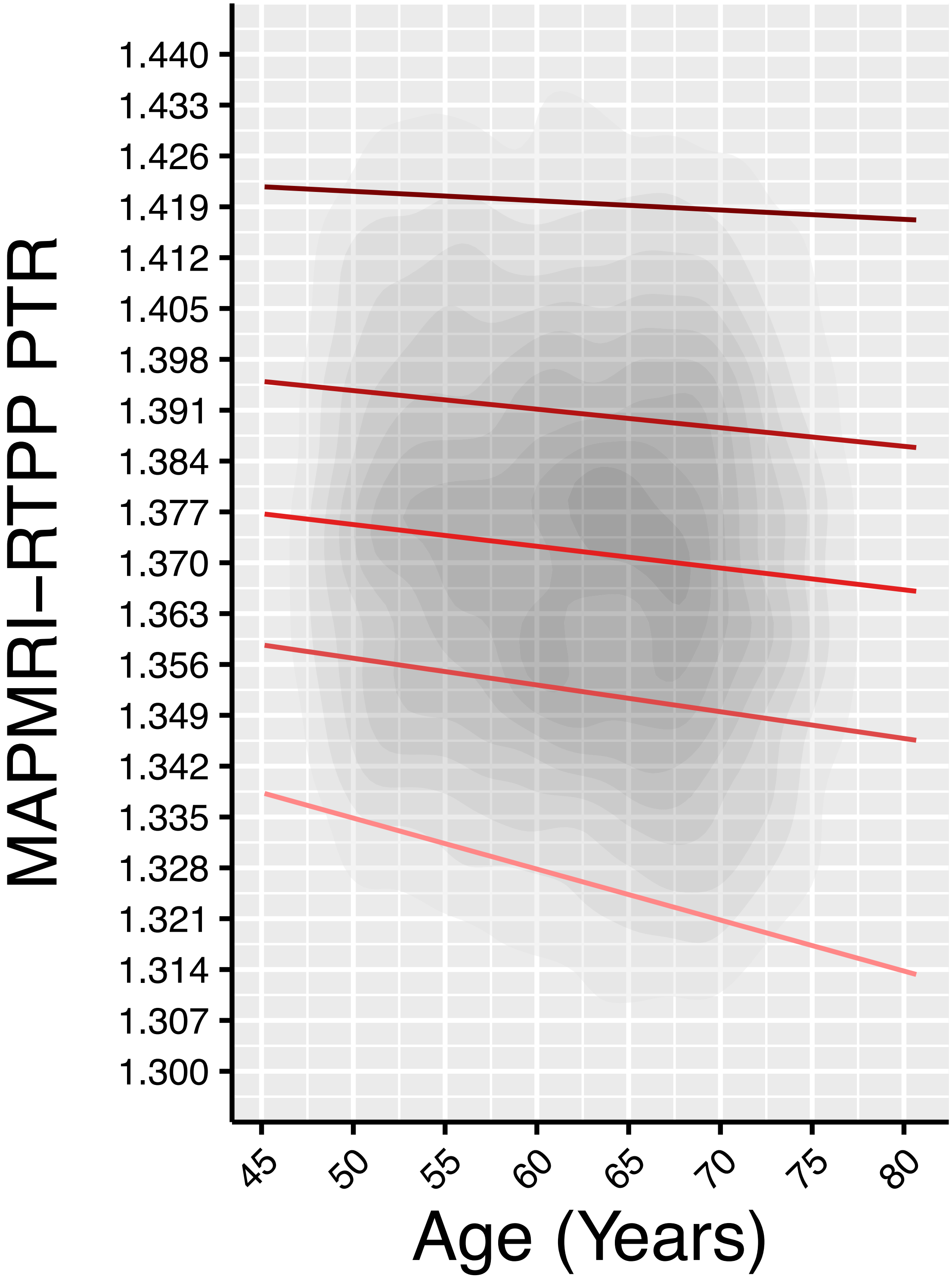

Figure S265. Full size normative centile reference curves calculated for the posterior thalamic radiation tract for MAPMRI-RTPP in females. Solid colored lines, ordered from lightest to darkest, indicate the following centiles: 5th, 25th, 50th, 75th, 95th. Gray overlay reflects kernel density (darker=greater degree of data point overlap). PTR = posterior thalamic radiation.

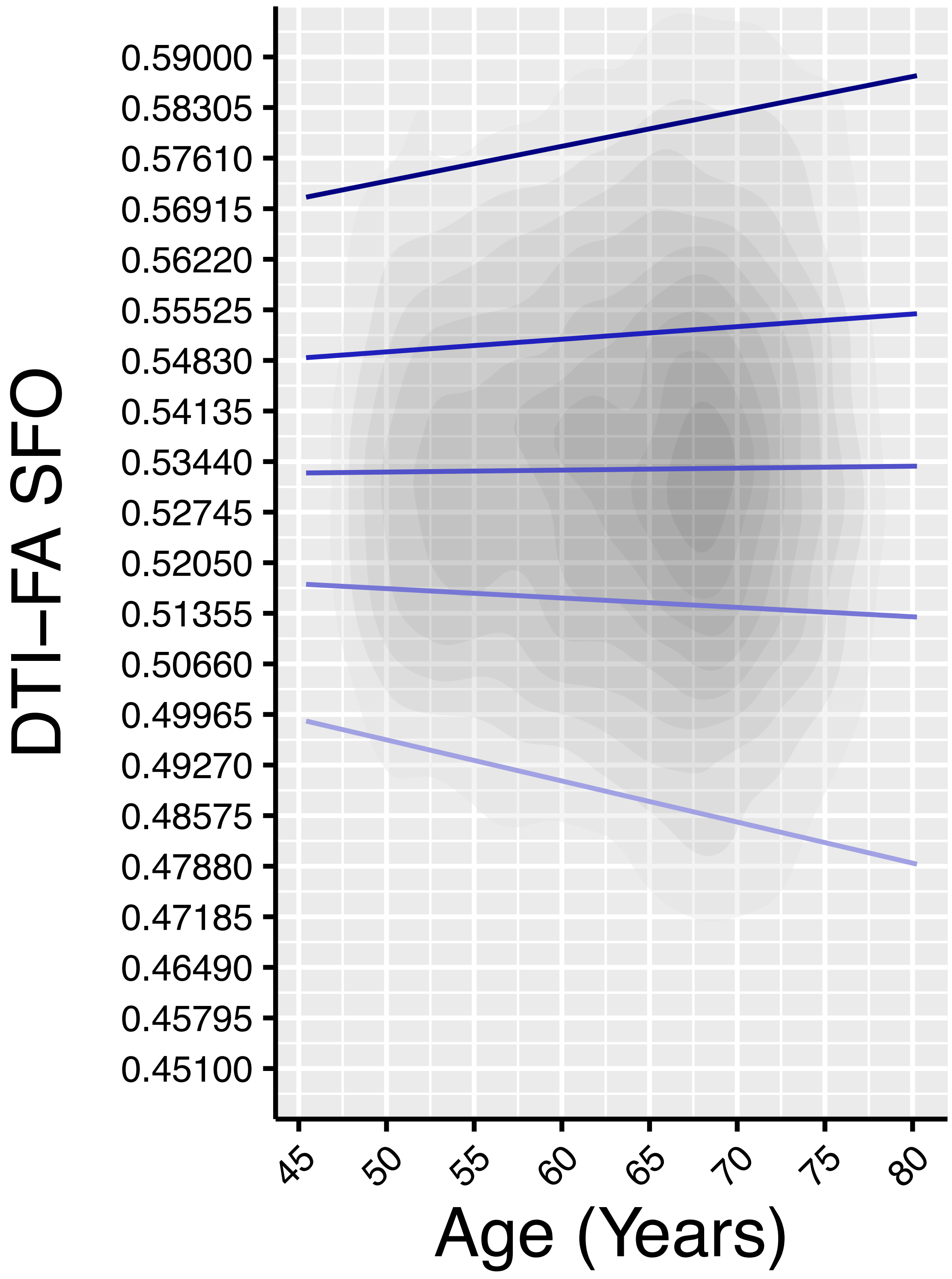

Figure S266. Full size normative centile reference curves calculated for the superior fronto-occpital fasciculus tract for DTI-FA in males. Solid colored lines, ordered from lightest to darkest, indicate the following centiles: 5th, 25th, 50th, 75th, 95th. Gray overlay reflects kernel density (darker=greater degree of data point overlap). SFO = superior fronto-occpital fasciculus.

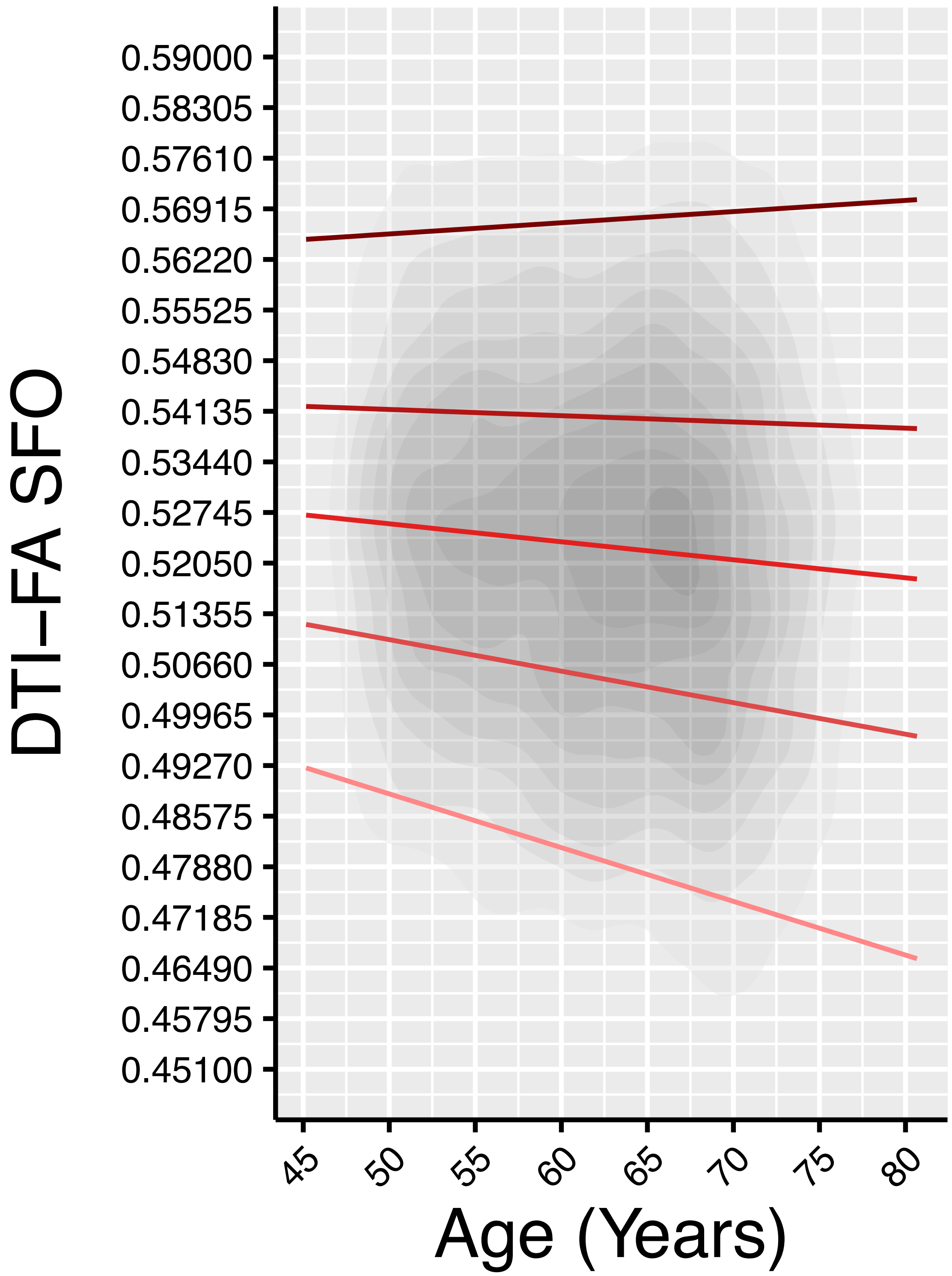

Figure S267. Full size normative centile reference curves calculated for the superior fronto-occpital fasciculus tract for DTI-FA in females. Solid colored lines, ordered from lightest to darkest, indicate the following centiles: 5th, 25th, 50th, 75th, 95th. Gray overlay reflects kernel density (darker=greater degree of data point overlap). SFO = superior fronto-occpital fasciculus.

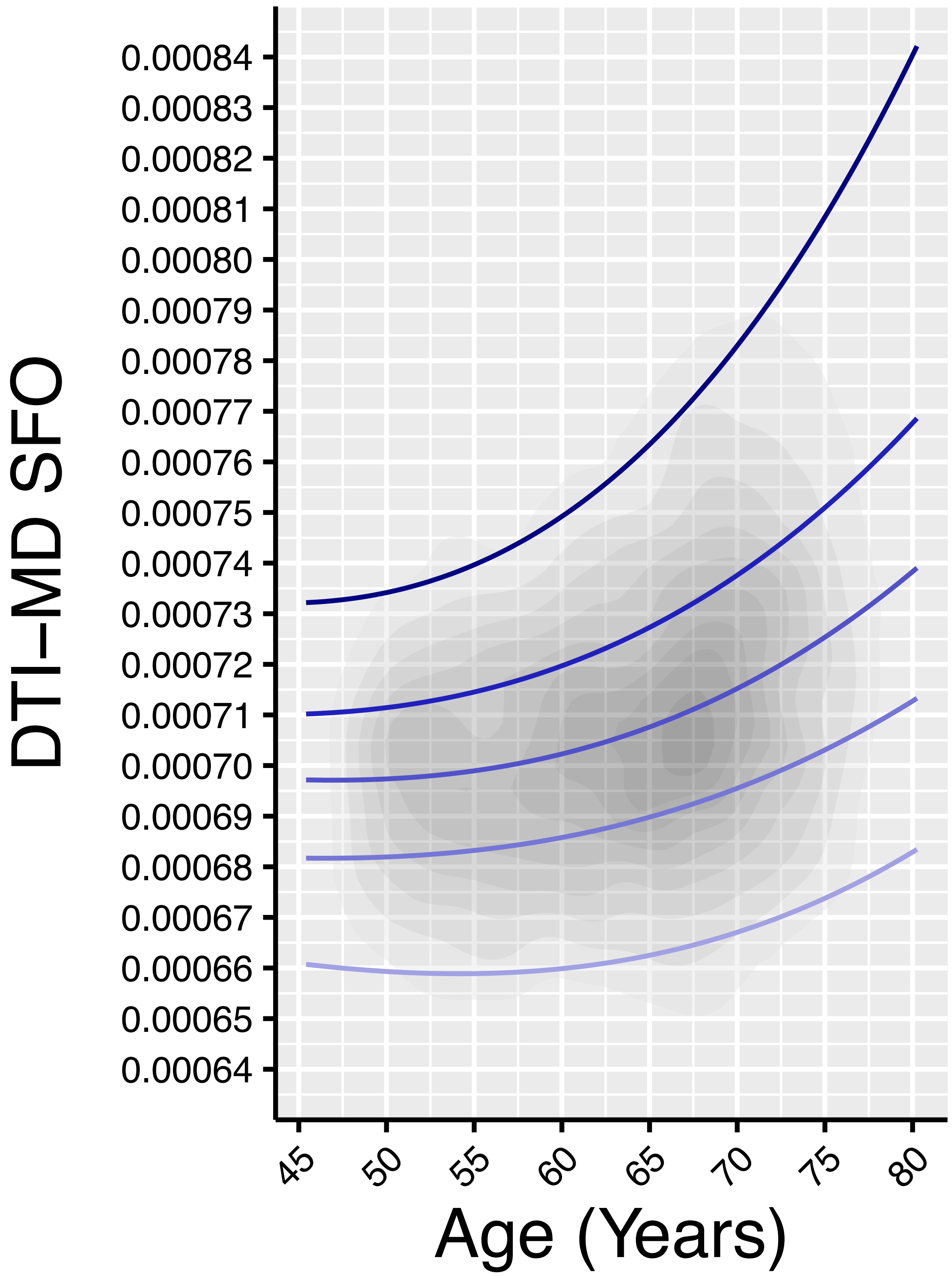

Figure S268. Full size normative centile reference curves calculated for the superior fronto-occpital fasciculus tract for DTI-MD in males. Solid colored lines, ordered from lightest to darkest, indicate the following centiles: 5th, 25th, 50th, 75th, 95th. Gray overlay reflects kernel density (darker=greater degree of data point overlap). SFO = superior fronto-occpital fasciculus.

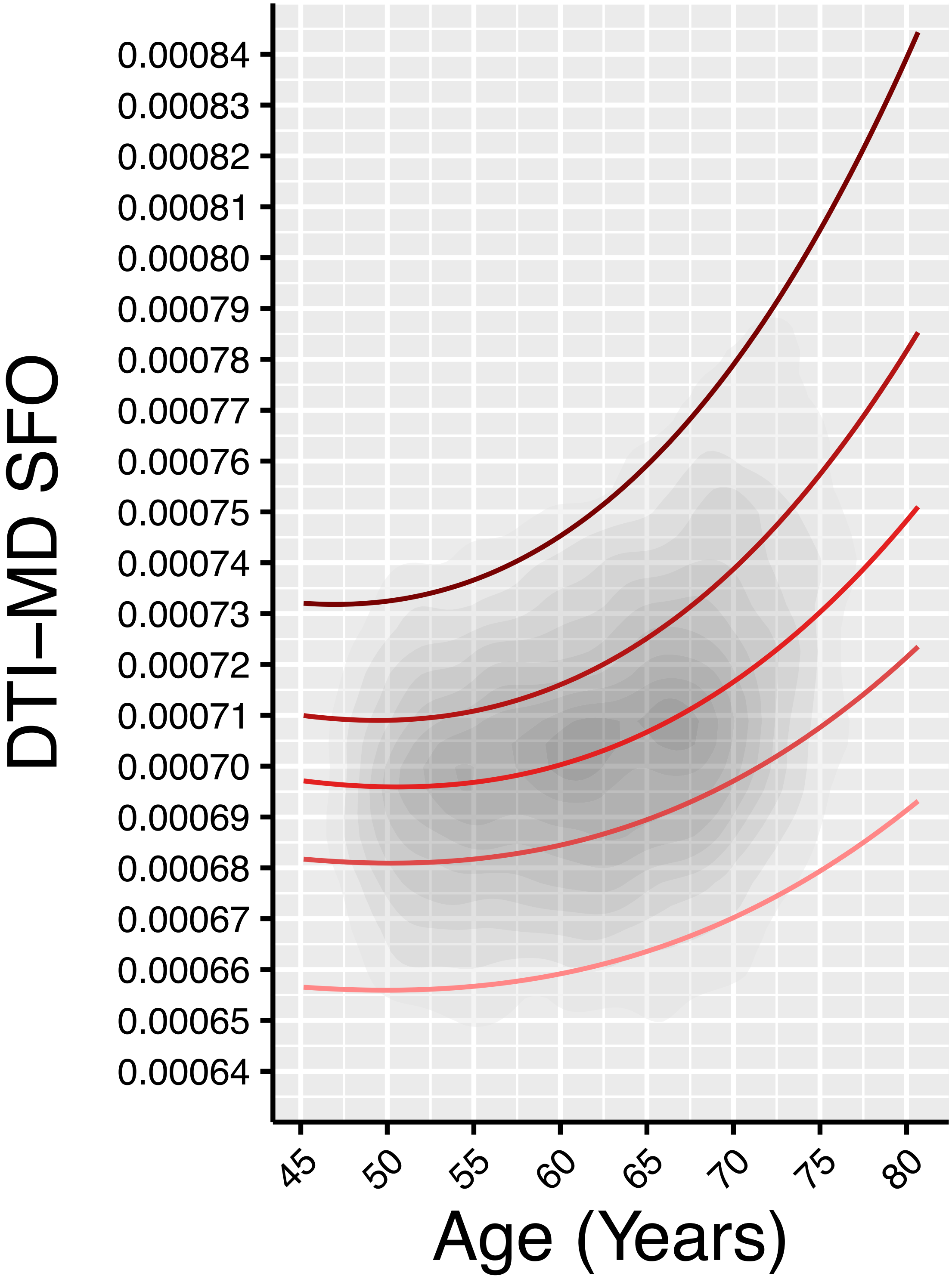

Figure S269. Full size normative centile reference curves calculated for the superior fronto-occpital fasciculus tract for DTI-MD in females. Solid colored lines, ordered from lightest to darkest, indicate the following centiles: 5th, 25th, 50th, 75th, 95th. Gray overlay reflects kernel density (darker=greater degree of data point overlap). SFO = superior fronto-occpital fasciculus.

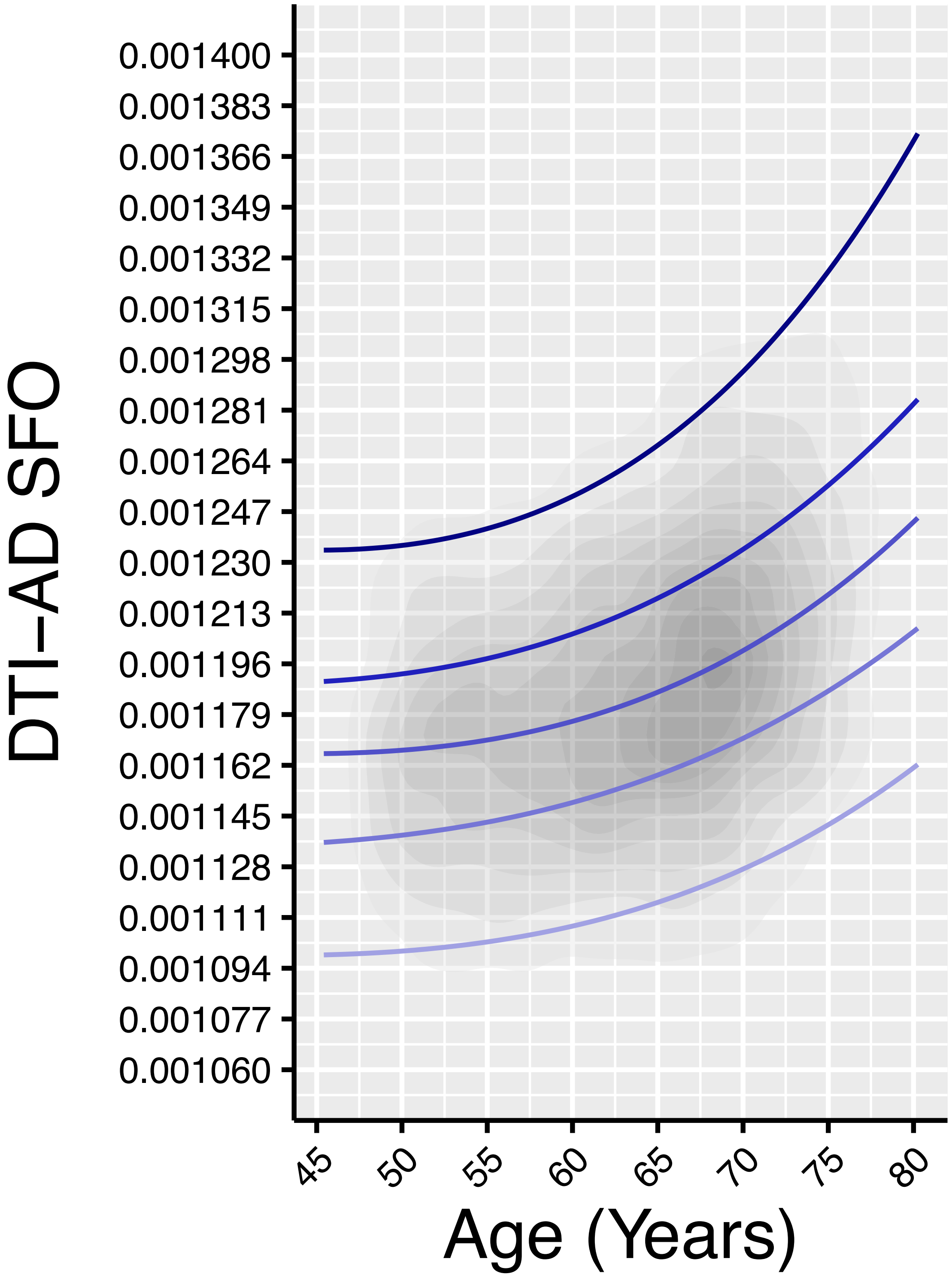

Figure S270. Full size normative centile reference curves calculated for the superior fronto-occpital fasciculus tract for DTI-AD in males. Solid colored lines, ordered from lightest to darkest, indicate the following centiles: 5th, 25th, 50th, 75th, 95th. Gray overlay reflects kernel density (darker=greater degree of data point overlap). SFO = superior fronto-occpital fasciculus.

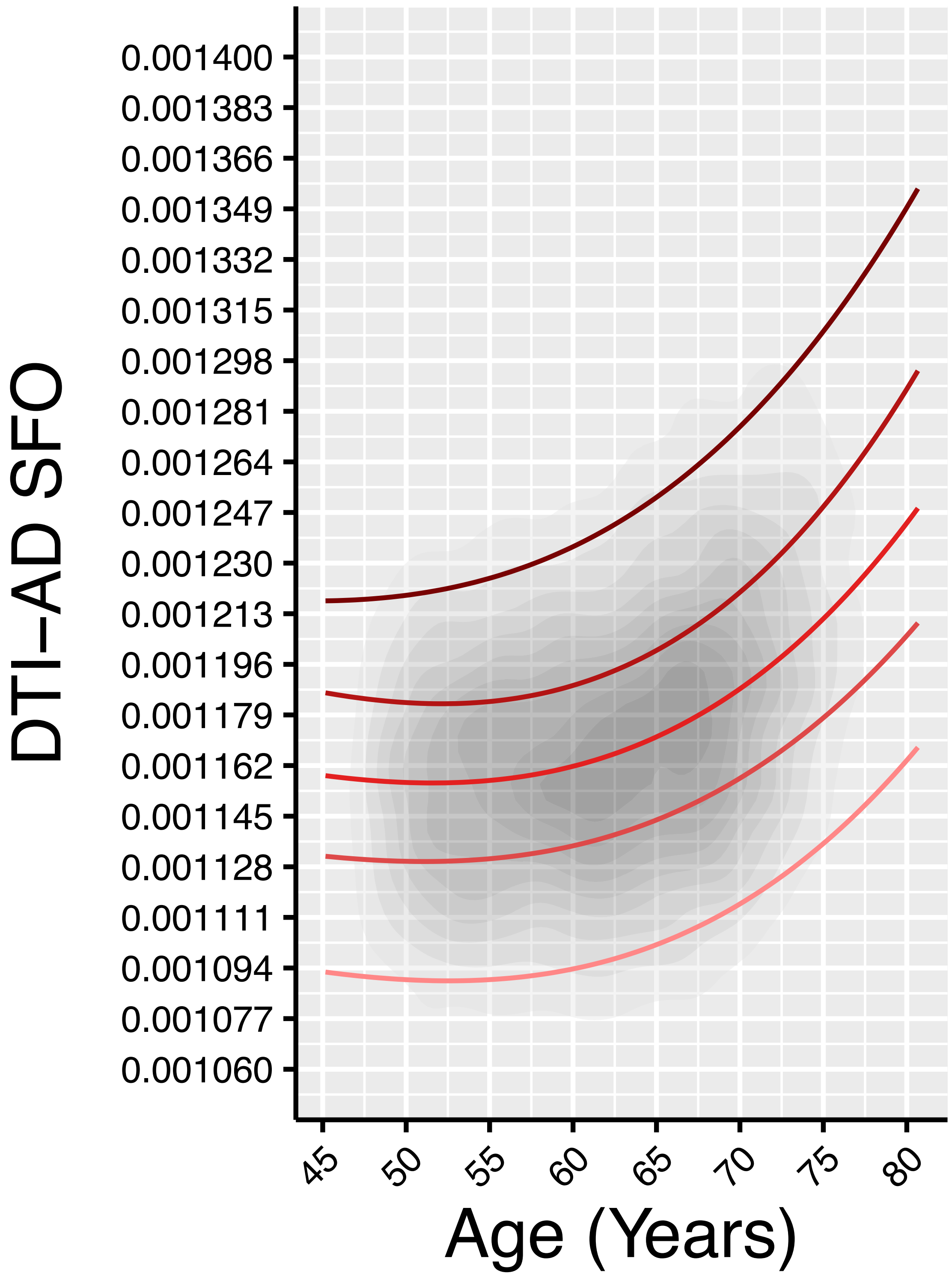

Figure S271. Full size normative centile reference curves calculated for the superior fronto-occpital fasciculus tract for DTI-AD in females. Solid colored lines, ordered from lightest to darkest, indicate the following centiles: 5th, 25th, 50th, 75th, 95th. Gray overlay reflects kernel density (darker=greater degree of data point overlap). SFO = superior fronto-occpital fasciculus.

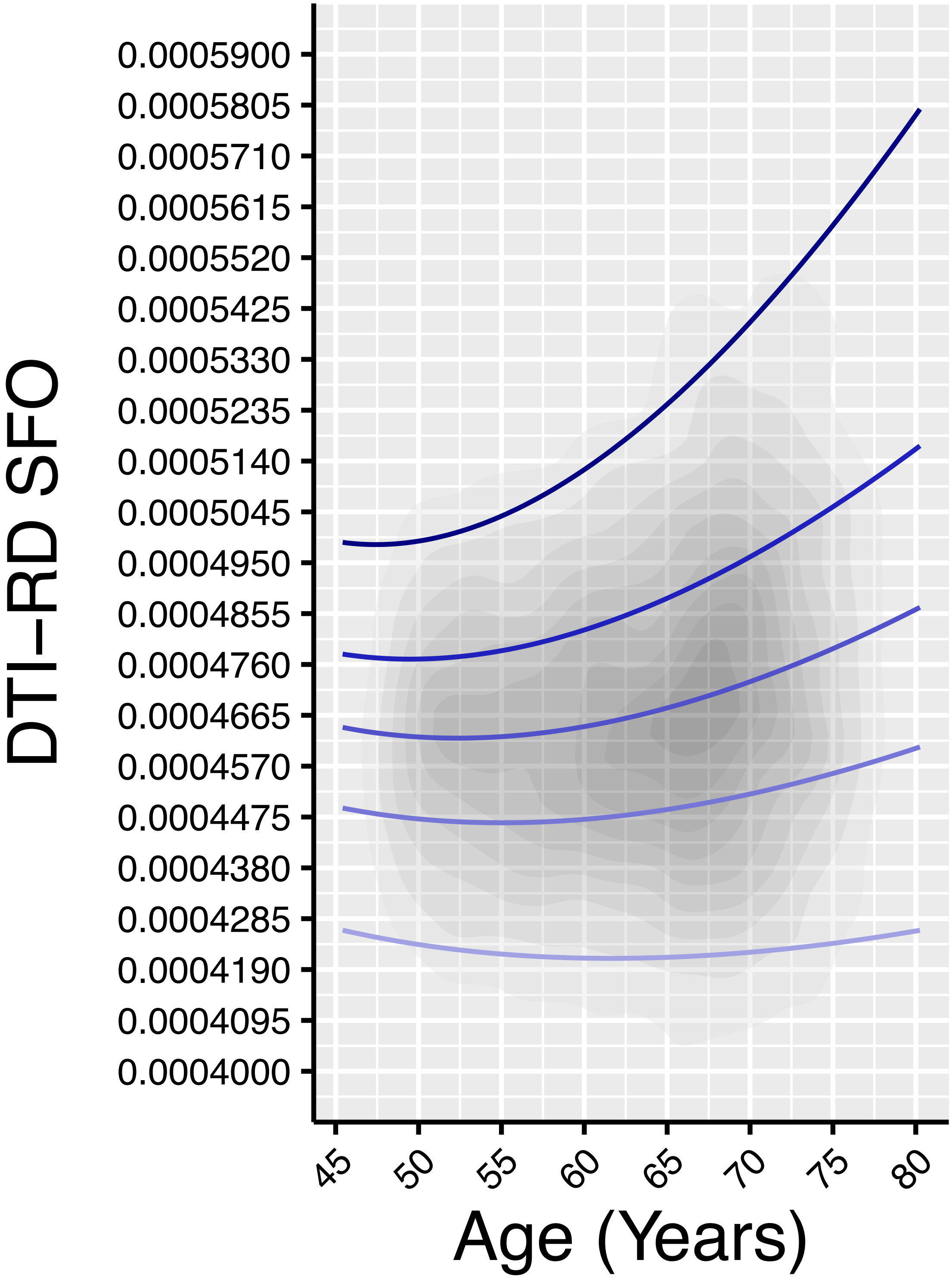

Figure S272. Full size normative centile reference curves calculated for the superior fronto-occpital fasciculus tract for DTI-RD in males. Solid colored lines, ordered from lightest to darkest, indicate the following centiles: 5th, 25th, 50th, 75th, 95th. Gray overlay reflects kernel density (darker=greater degree of data point overlap). SFO = superior fronto-occpital fasciculus.

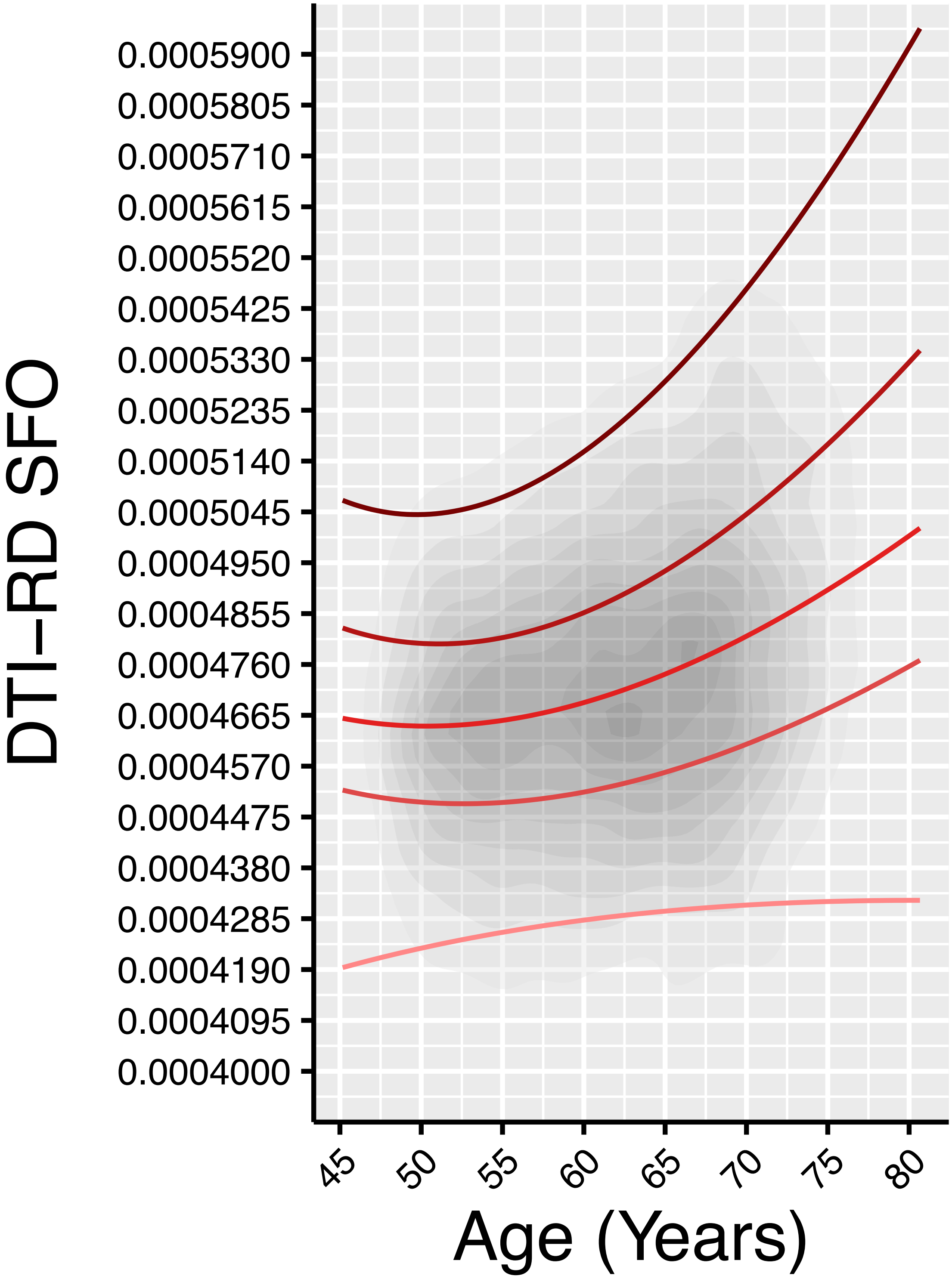

Figure S273. Full size normative centile reference curves calculated for the superior fronto-occpital fasciculus tract for DTI-RD in females. Solid colored lines, ordered from lightest to darkest, indicate the following centiles: 5th, 25th, 50th, 75th, 95th. Gray overlay reflects kernel density (darker=greater degree of data point overlap). SFO = superior fronto-occpital fasciculus.

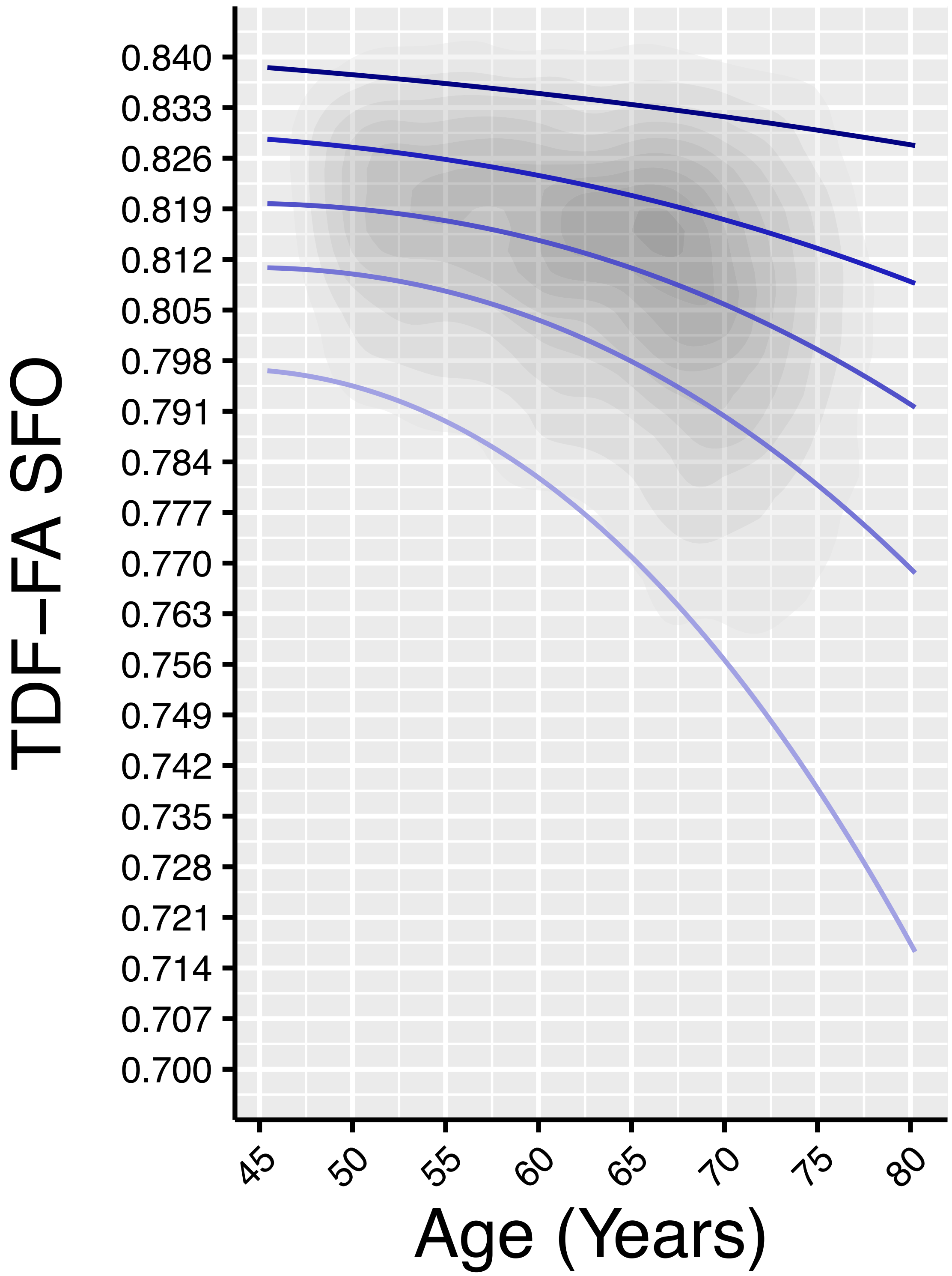

Figure S274. Full size normative centile reference curves calculated for the superior fronto-occpital fasciculus tract for TDF-FA in males. Solid colored lines, ordered from lightest to darkest, indicate the following centiles: 5th, 25th, 50th, 75th, 95th. Gray overlay reflects kernel density (darker=greater degree of data point overlap). SFO = superior fronto-occpital fasciculus.

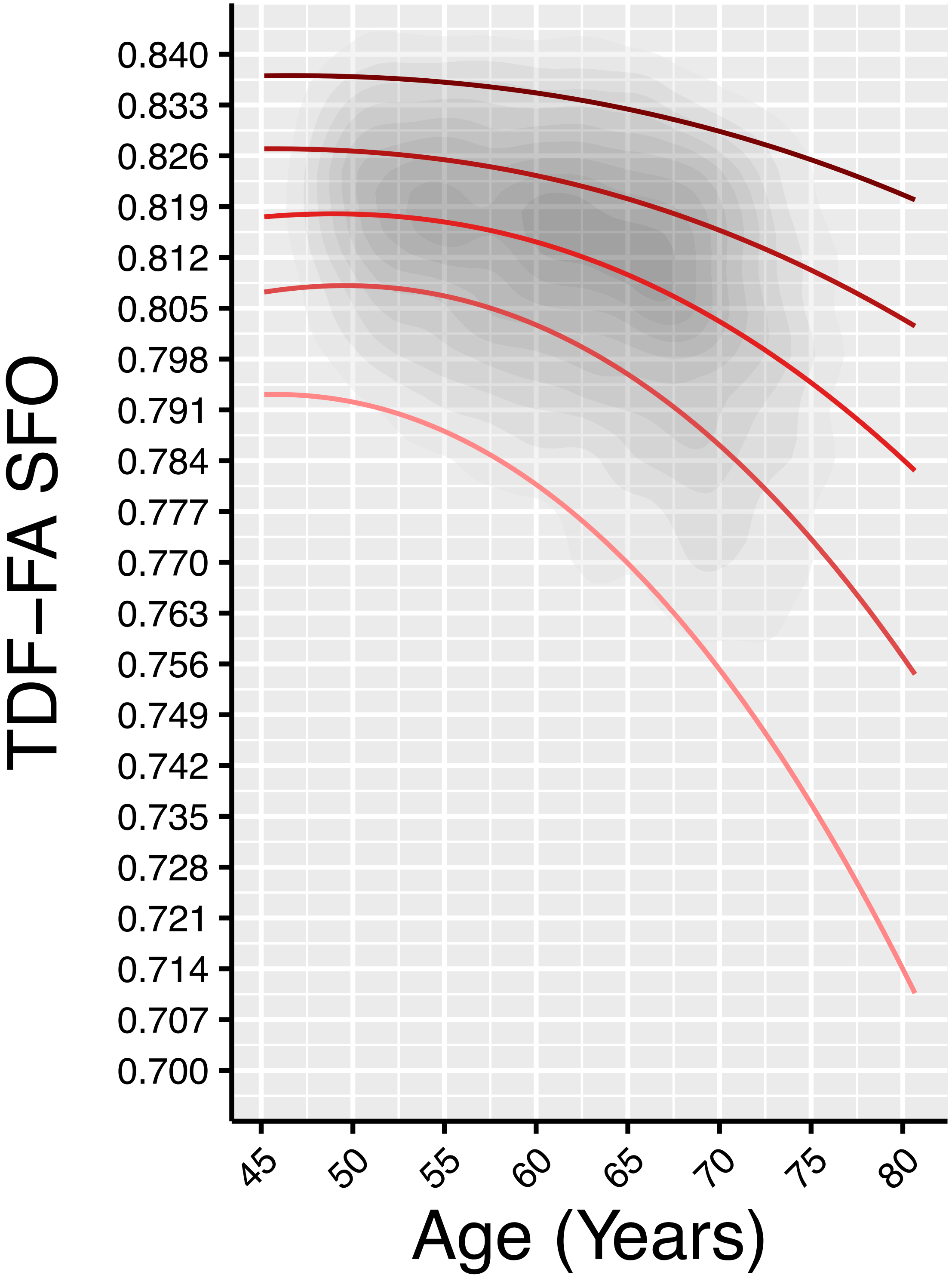

Figure S275. Full size normative centile reference curves calculated for the superior fronto-occpital fasciculus tract for TDF-FA in females. Solid colored lines, ordered from lightest to darkest, indicate the following centiles: 5th, 25th, 50th, 75th, 95th. Gray overlay reflects kernel density (darker=greater degree of data point overlap). SFO = superior fronto-occpital fasciculus.

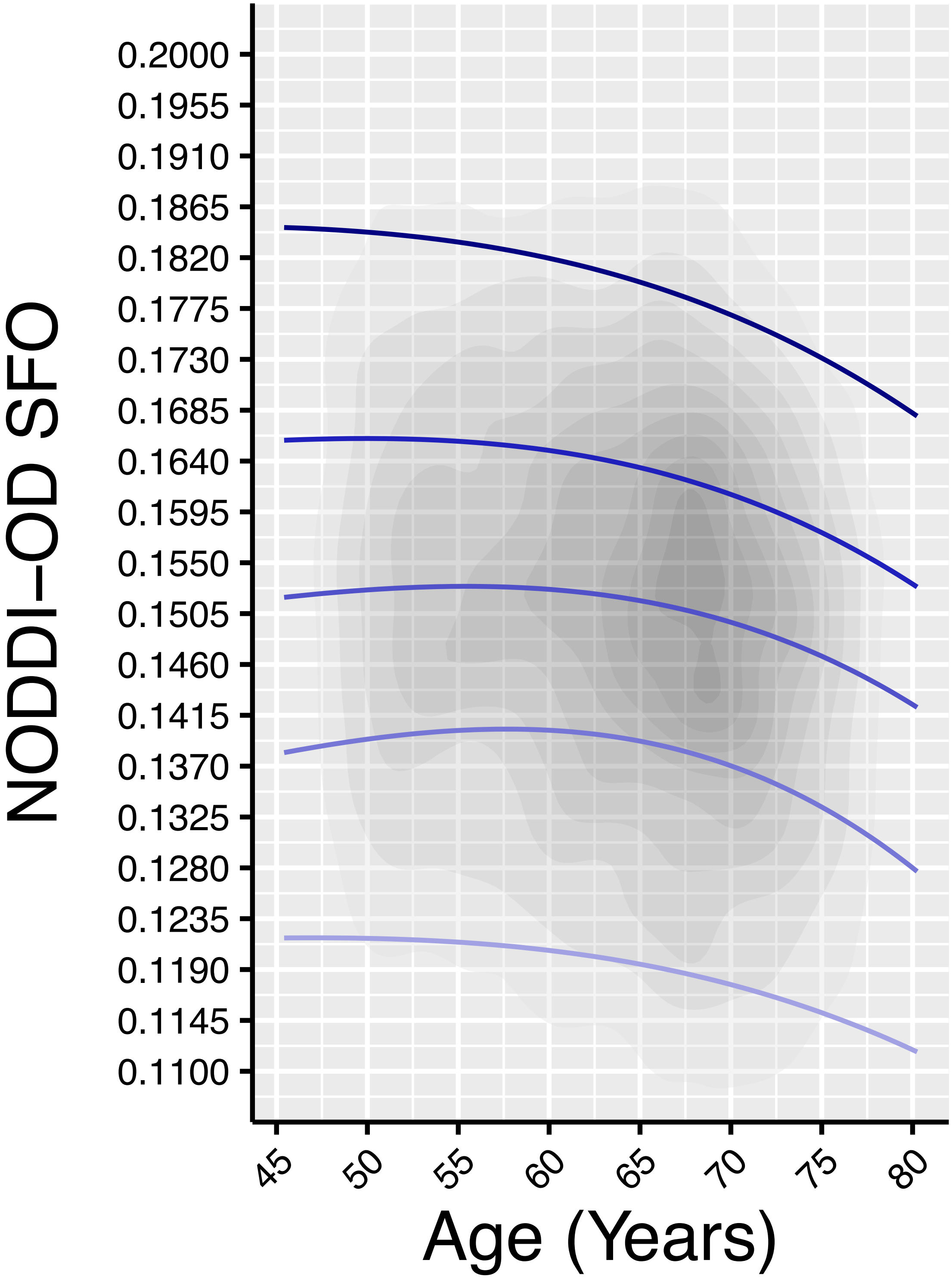

Figure S276. Full size normative centile reference curves calculated for the superior fronto-occpital fasciculus tract for NODDI-OD in males. Solid colored lines, ordered from lightest to darkest, indicate the following centiles: 5th, 25th, 50th, 75th, 95th. Gray overlay reflects kernel density (darker=greater degree of data point overlap). SFO = superior fronto-occpital fasciculus.

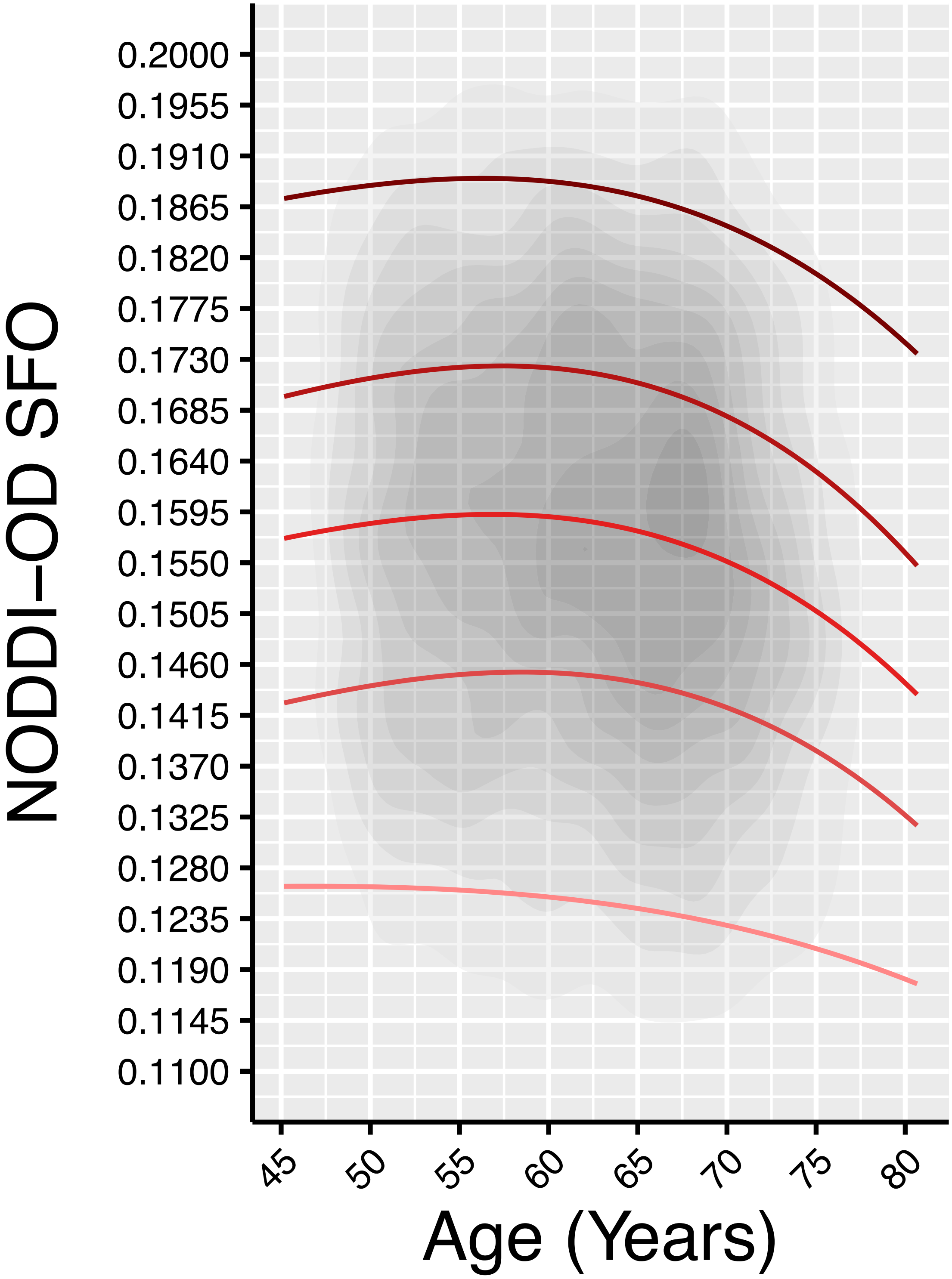

Figure S277. Full size normative centile reference curves calculated for the superior fronto-occpital fasciculus tract for NODDI-OD in females. Solid colored lines, ordered from lightest to darkest, indicate the following centiles: 5th, 25th, 50th, 75th, 95th. Gray overlay reflects kernel density (darker=greater degree of data point overlap). SFO = superior fronto-occpital fasciculus.

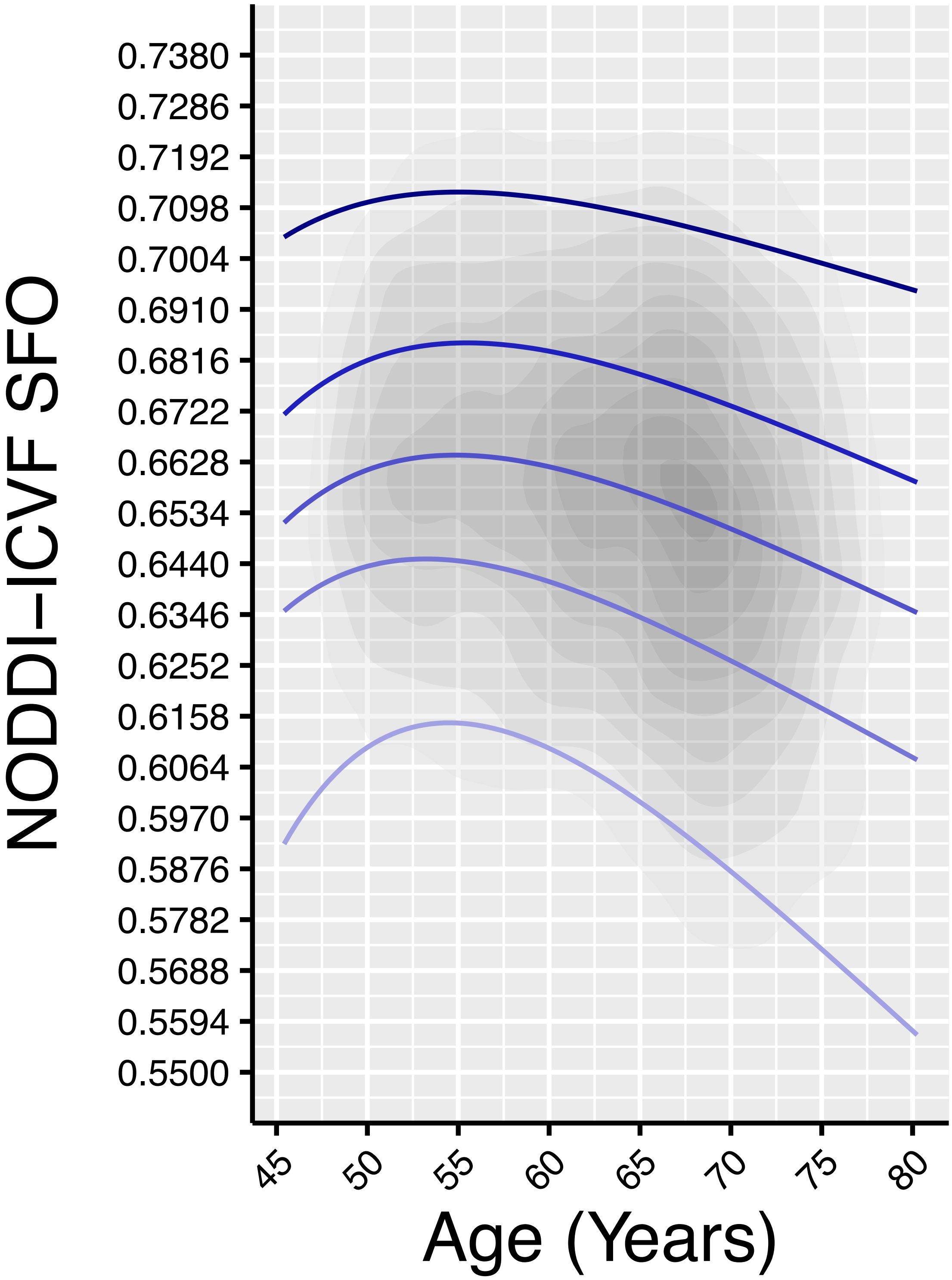

Figure S278. Full size normative centile reference curves calculated for the superior fronto-occpital fasciculus tract for NODDI-ICVF in males. Solid colored lines, ordered from lightest to darkest, indicate the following centiles: 5th, 25th, 50th, 75th, 95th. Gray overlay reflects kernel density (darker=greater degree of data point overlap). SFO = superior fronto-occpital fasciculus.

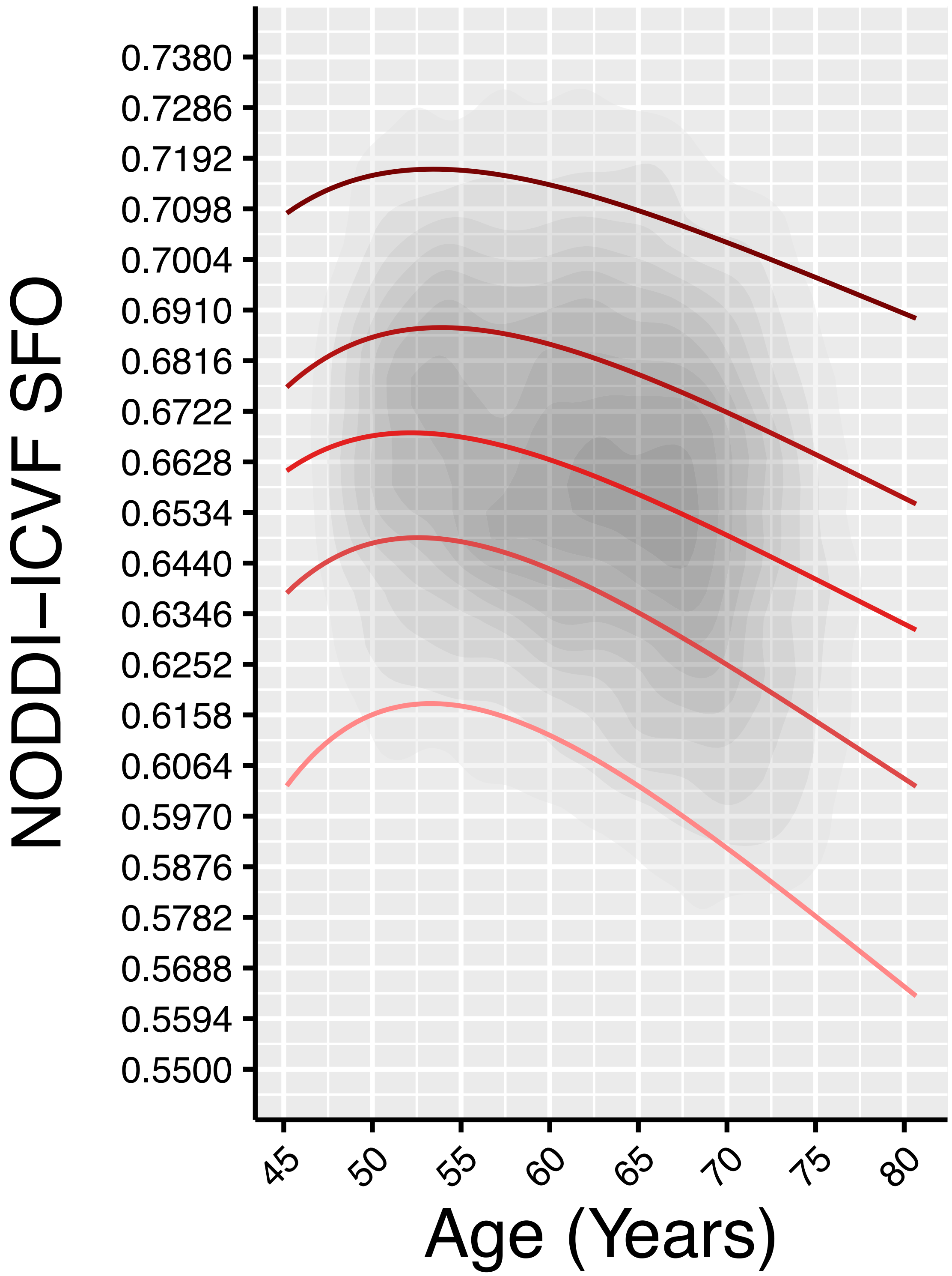

Figure S279. Full size normative centile reference curves calculated for the superior fronto-occpital fasciculus tract for NODDI-ICVF in females. Solid colored lines, ordered from lightest to darkest, indicate the following centiles: 5th, 25th, 50th, 75th, 95th. Gray overlay reflects kernel density (darker=greater degree of data point overlap). SFO = superior fronto-occpital fasciculus.

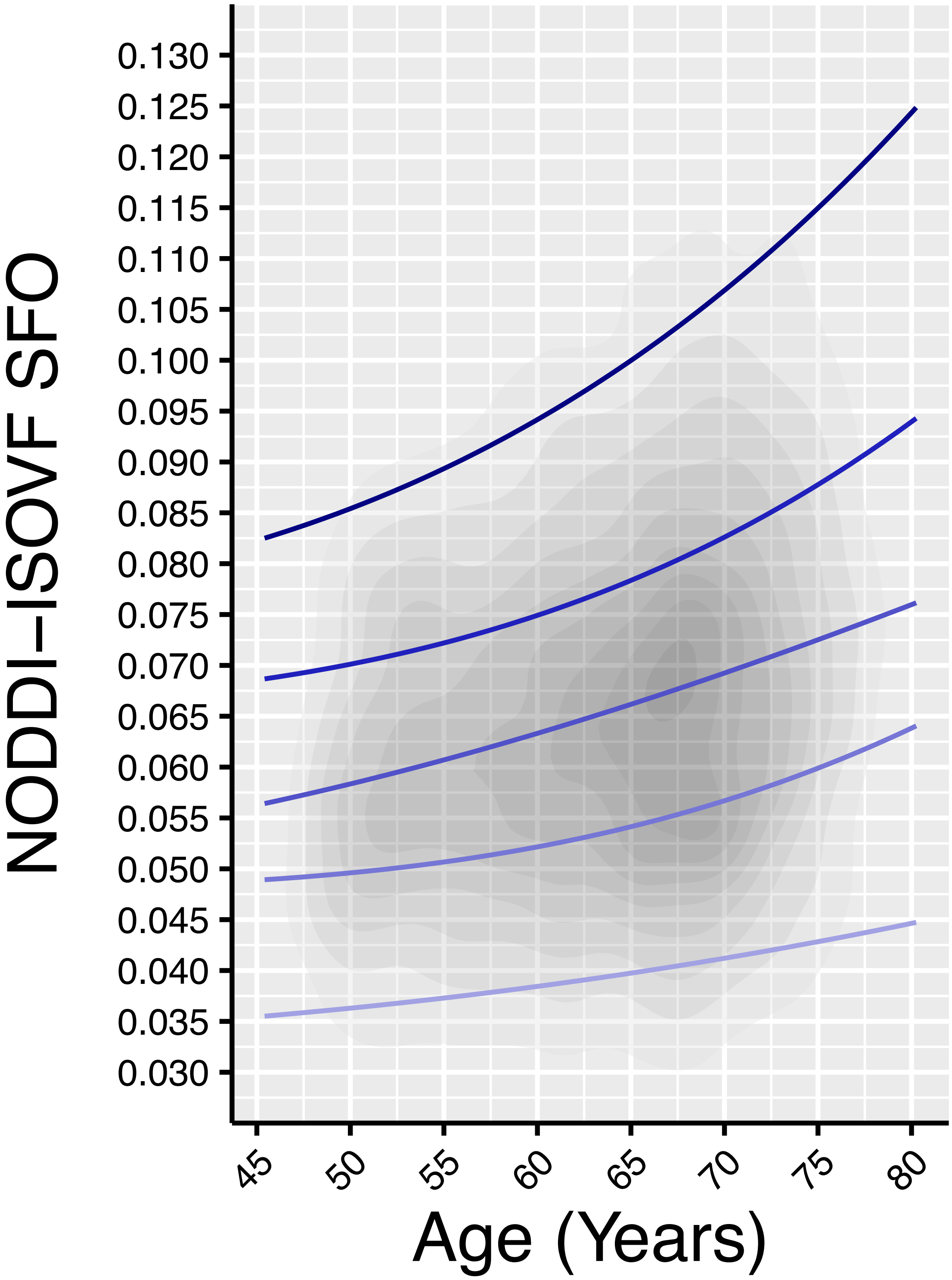

Figure S280. Full size normative centile reference curves calculated for the superior fronto-occpital fasciculus tract for NODDI-ISOVF in males. Solid colored lines, ordered from lightest to darkest, indicate the following centiles: 5th, 25th, 50th, 75th, 95th. Gray overlay reflects kernel density (darker=greater degree of data point overlap). SFO = superior fronto-occpital fasciculus.

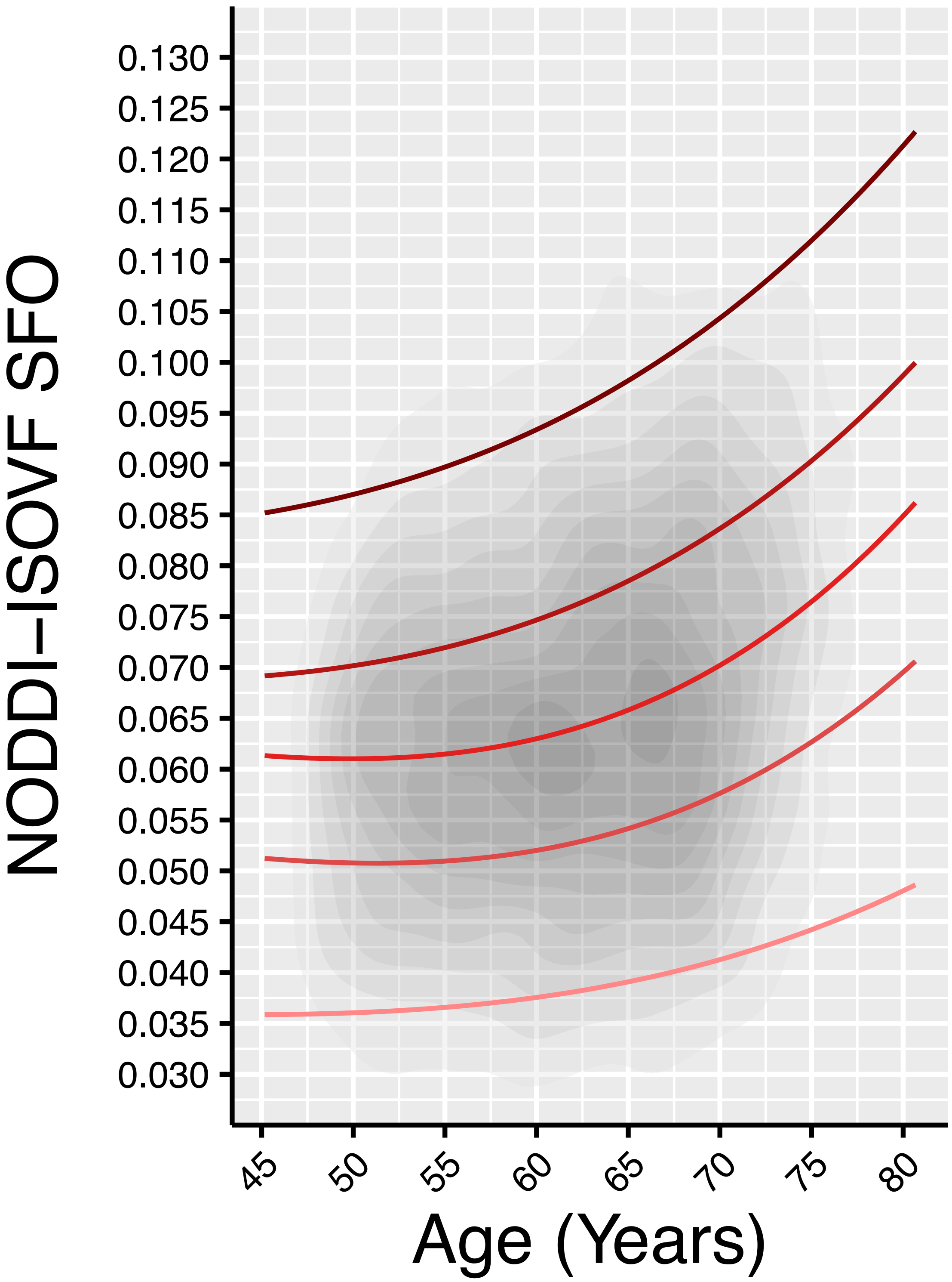

Figure S281. Full size normative centile reference curves calculated for the superior fronto-occpital fasciculus tract for NODDI-ISOVF in females. Solid colored lines, ordered from lightest to darkest, indicate the following centiles: 5th, 25th, 50th, 75th, 95th. Gray overlay reflects kernel density (darker=greater degree of data point overlap). SFO = superior fronto-occpital fasciculus.

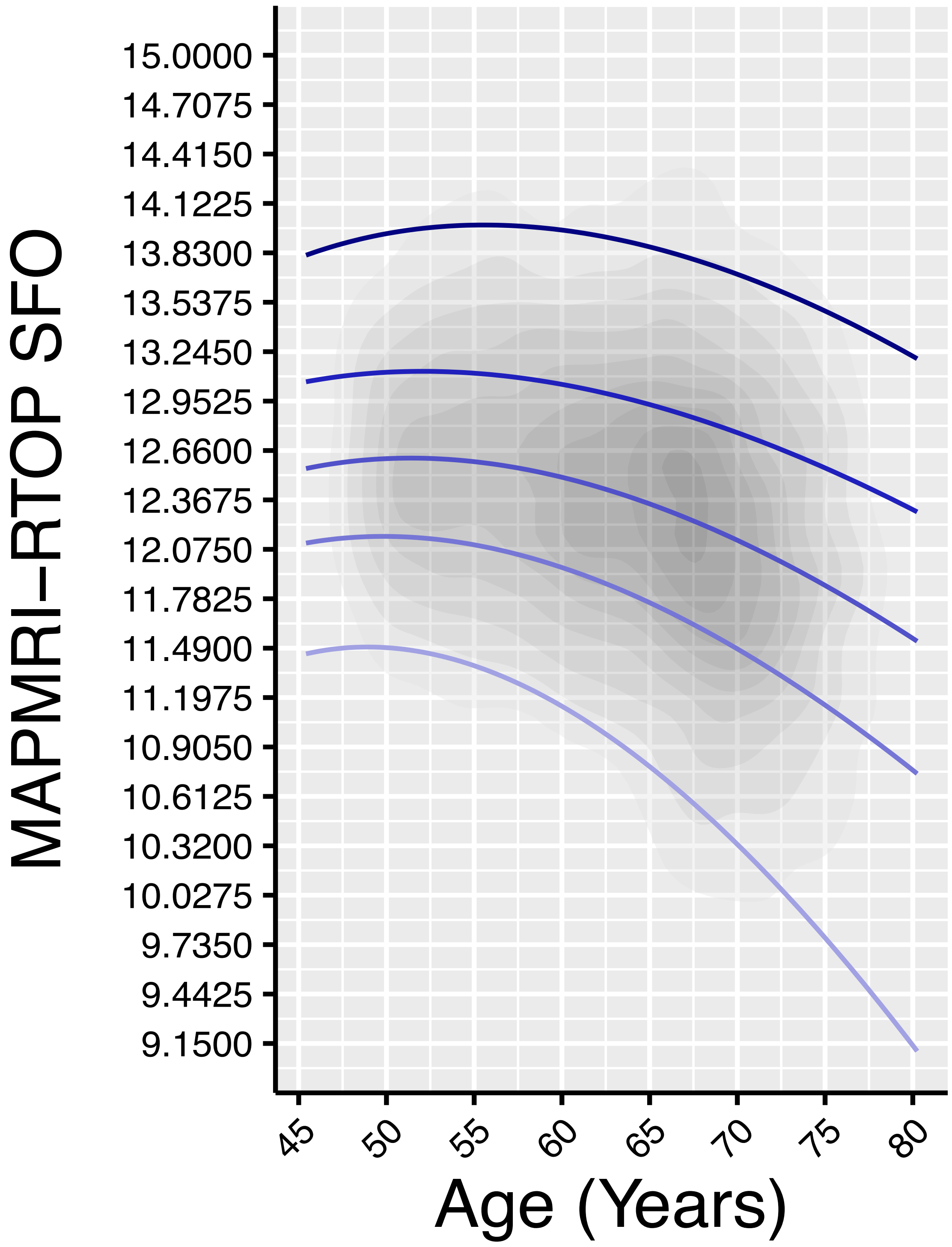

Figure S282. Full size normative centile reference curves calculated for the superior fronto-occpital fasciculus tract for MAPMRI-RTOP in males. Solid colored lines, ordered from lightest to darkest, indicate the following centiles: 5th, 25th, 50th, 75th, 95th. Gray overlay reflects kernel density (darker=greater degree of data point overlap). SFO = superior fronto-occpital fasciculus.

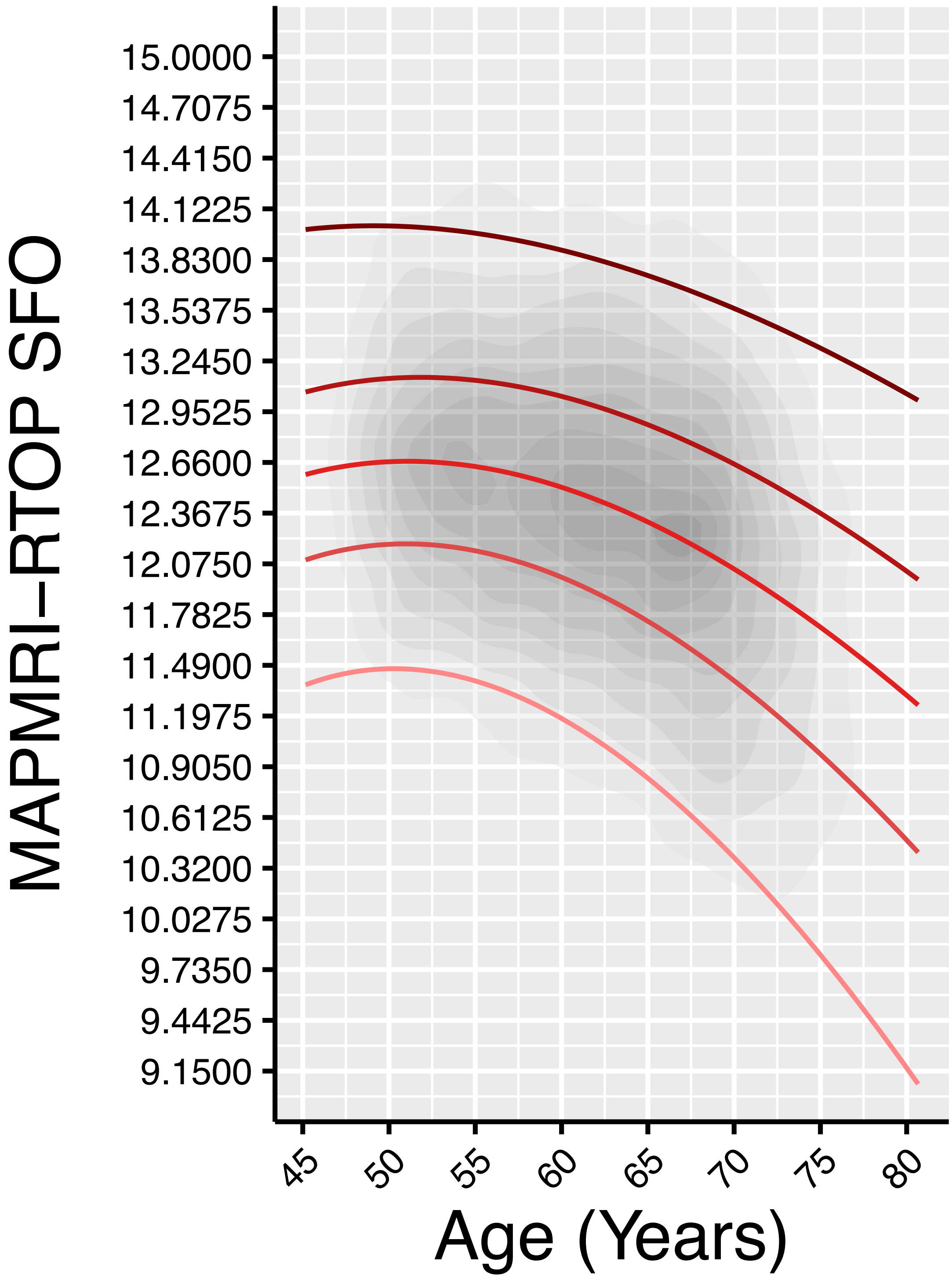

Figure S283. Full size normative centile reference curves calculated for the superior fronto-occpital fasciculus tract for MAPMRI-RTOP in females. Solid colored lines, ordered from lightest to darkest, indicate the following centiles: 5th, 25th, 50th, 75th, 95th. Gray overlay reflects kernel density (darker=greater degree of data point overlap). SFO = superior fronto-occpital fasciculus.

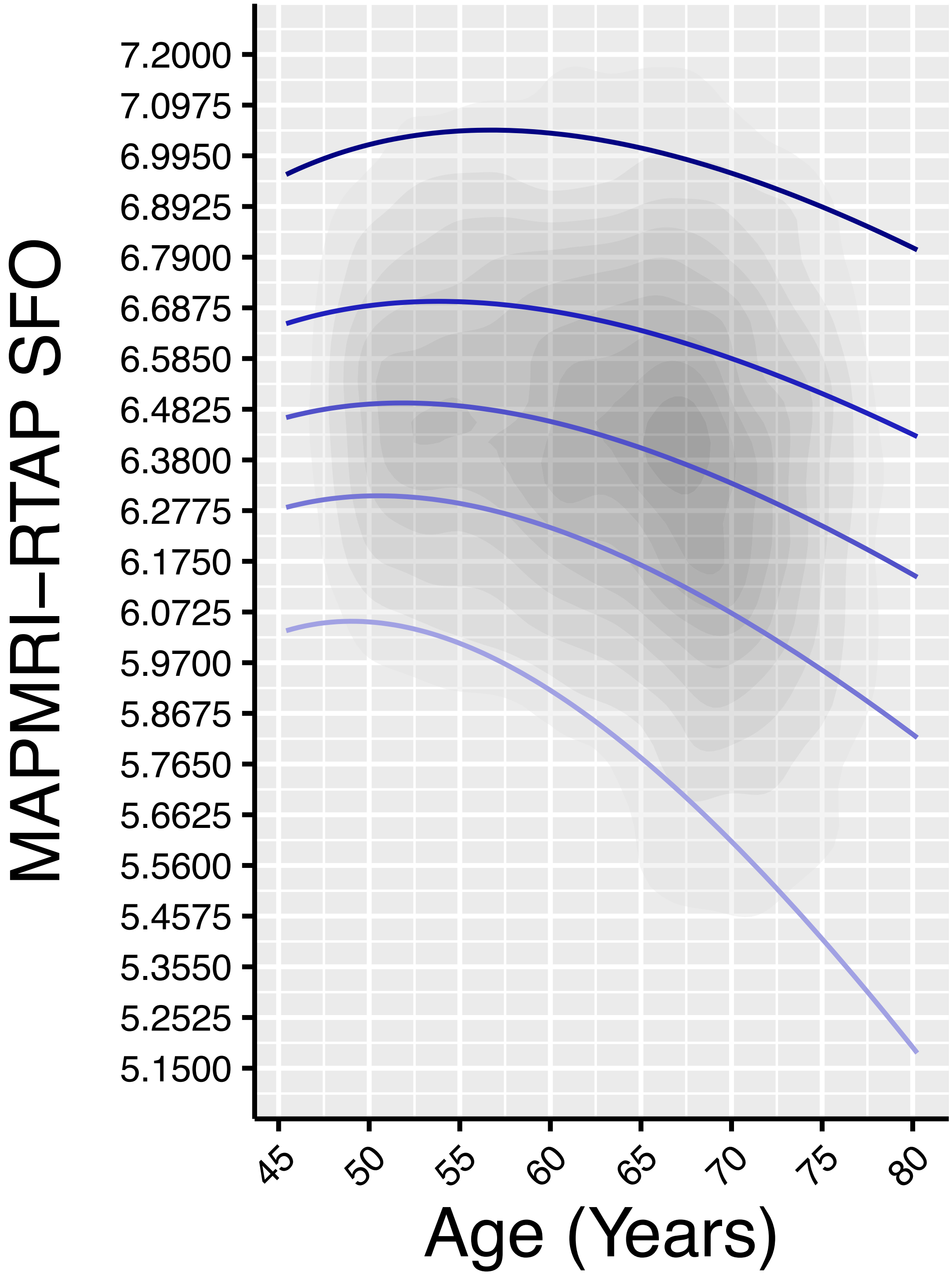

Figure S284. Full size normative centile reference curves calculated for the superior fronto-occpital fasciculus tract for MAPMRI-RTAP in males. Solid colored lines, ordered from lightest to darkest, indicate the following centiles: 5th, 25th, 50th, 75th, 95th. Gray overlay reflects kernel density (darker=greater degree of data point overlap). SFO = superior fronto-occpital fasciculus.

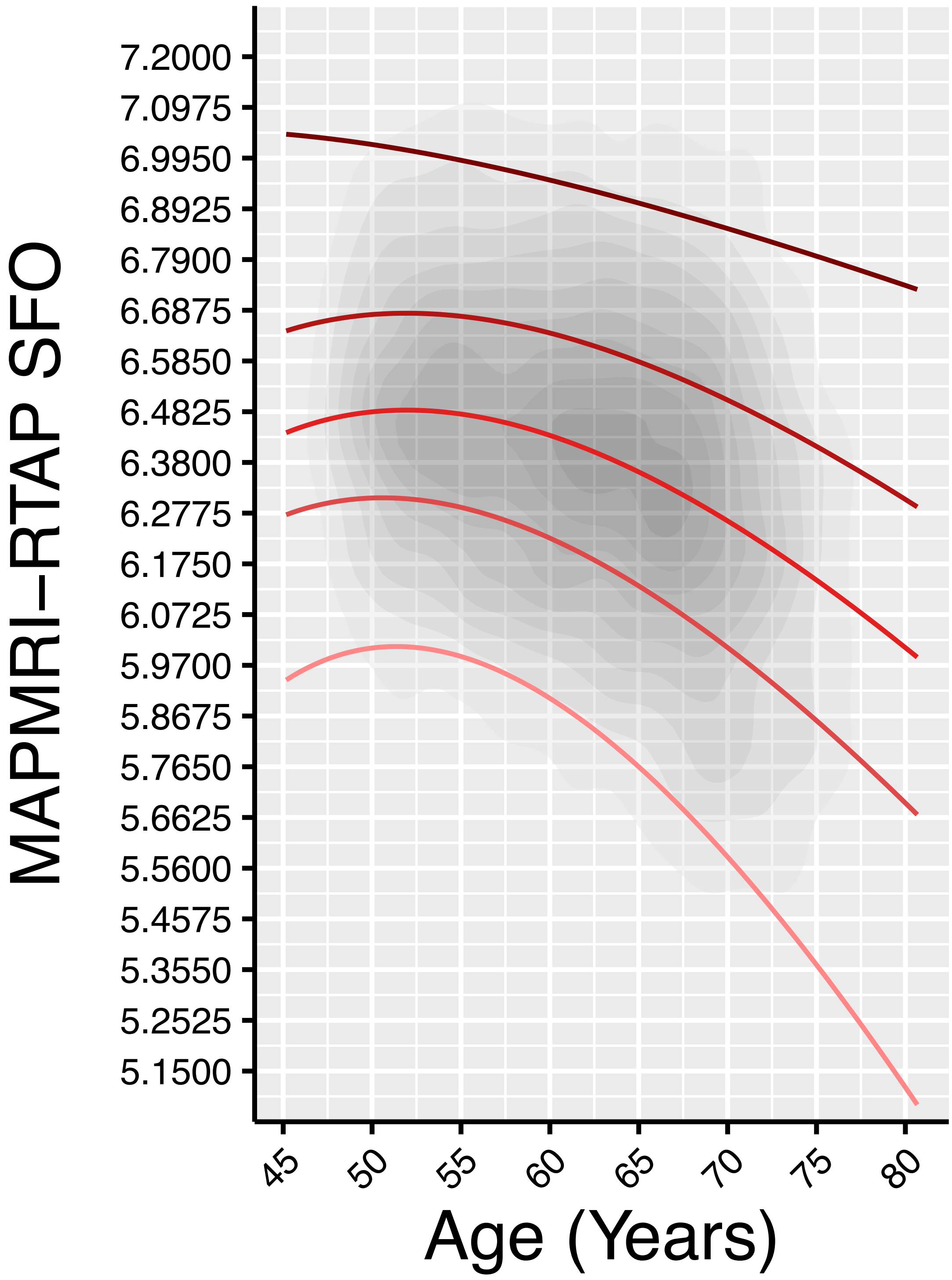

Figure S285. Full size normative centile reference curves calculated for the superior fronto-occpital fasciculus tract for MAPMRI-RTAP in females. Solid colored lines, ordered from lightest to darkest, indicate the following centiles: 5th, 25th, 50th, 75th, 95th. Gray overlay reflects kernel density (darker=greater degree of data point overlap). SFO = superior fronto-occpital fasciculus.

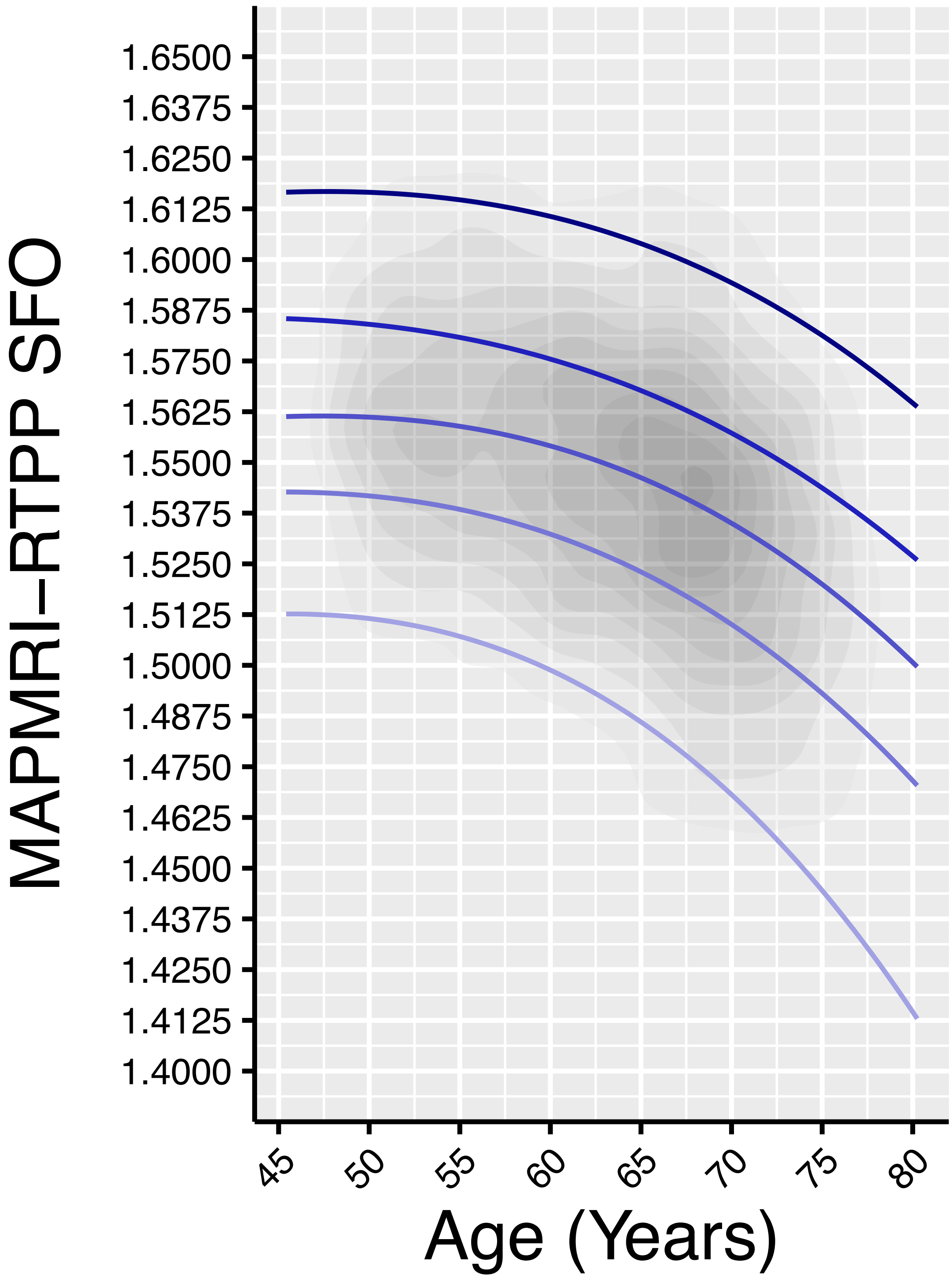

Figure S286. Full size normative centile reference curves calculated for the superior fronto-occpital fasciculus tract for MAPMRI-RTPP in males. Solid colored lines, ordered from lightest to darkest, indicate the following centiles: 5th, 25th, 50th, 75th, 95th. Gray overlay reflects kernel density (darker=greater degree of data point overlap). SFO = superior fronto-occpital fasciculus.

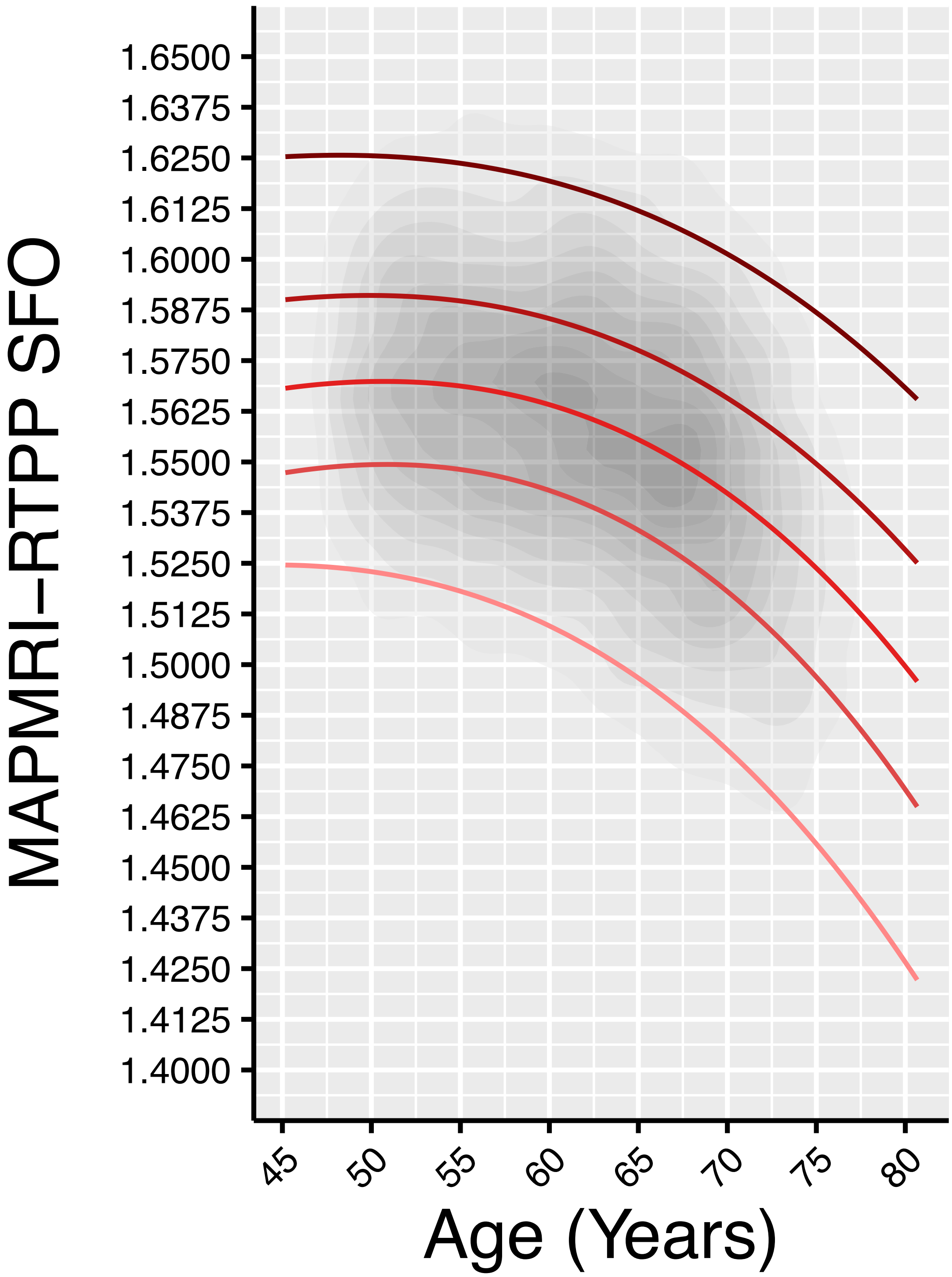

Figure S287. Full size normative centile reference curves calculated for the superior fronto-occpital fasciculus tract for MAPMRI-RTPP in females. Solid colored lines, ordered from lightest to darkest, indicate the following centiles: 5th, 25th, 50th, 75th, 95th. Gray overlay reflects kernel density (darker=greater degree of data point overlap). SFO = superior fronto-occpital fasciculus.

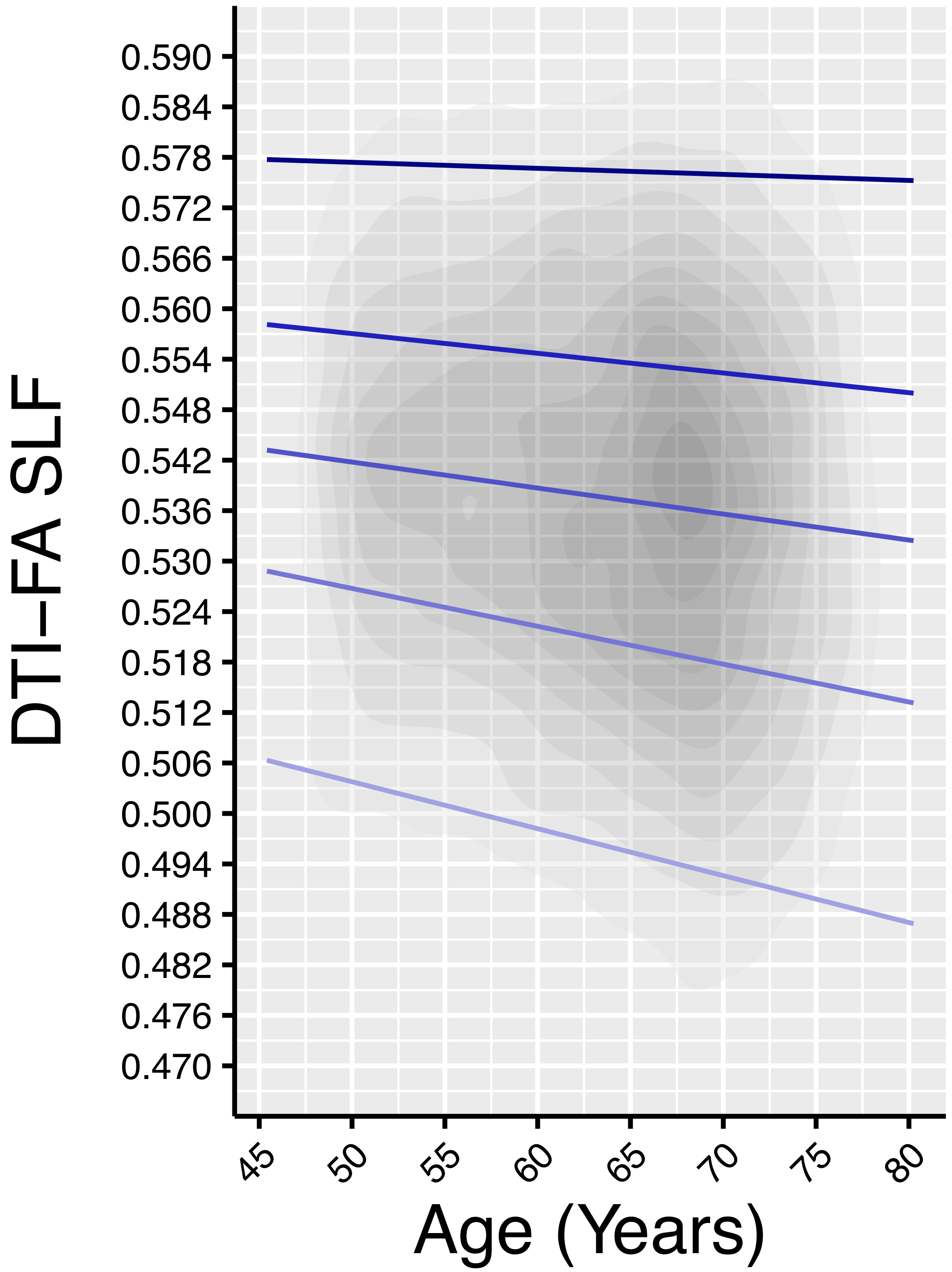

Figure S288. Full size normative centile reference curves calculated for the superior longitudinal fasciculus tract for DTI-FA in males. Solid colored lines, ordered from lightest to darkest, indicate the following centiles: 5th, 25th, 50th, 75th, 95th. Gray overlay reflects kernel density (darker=greater degree of data point overlap). SLF = superior longitudinal fasciculus.

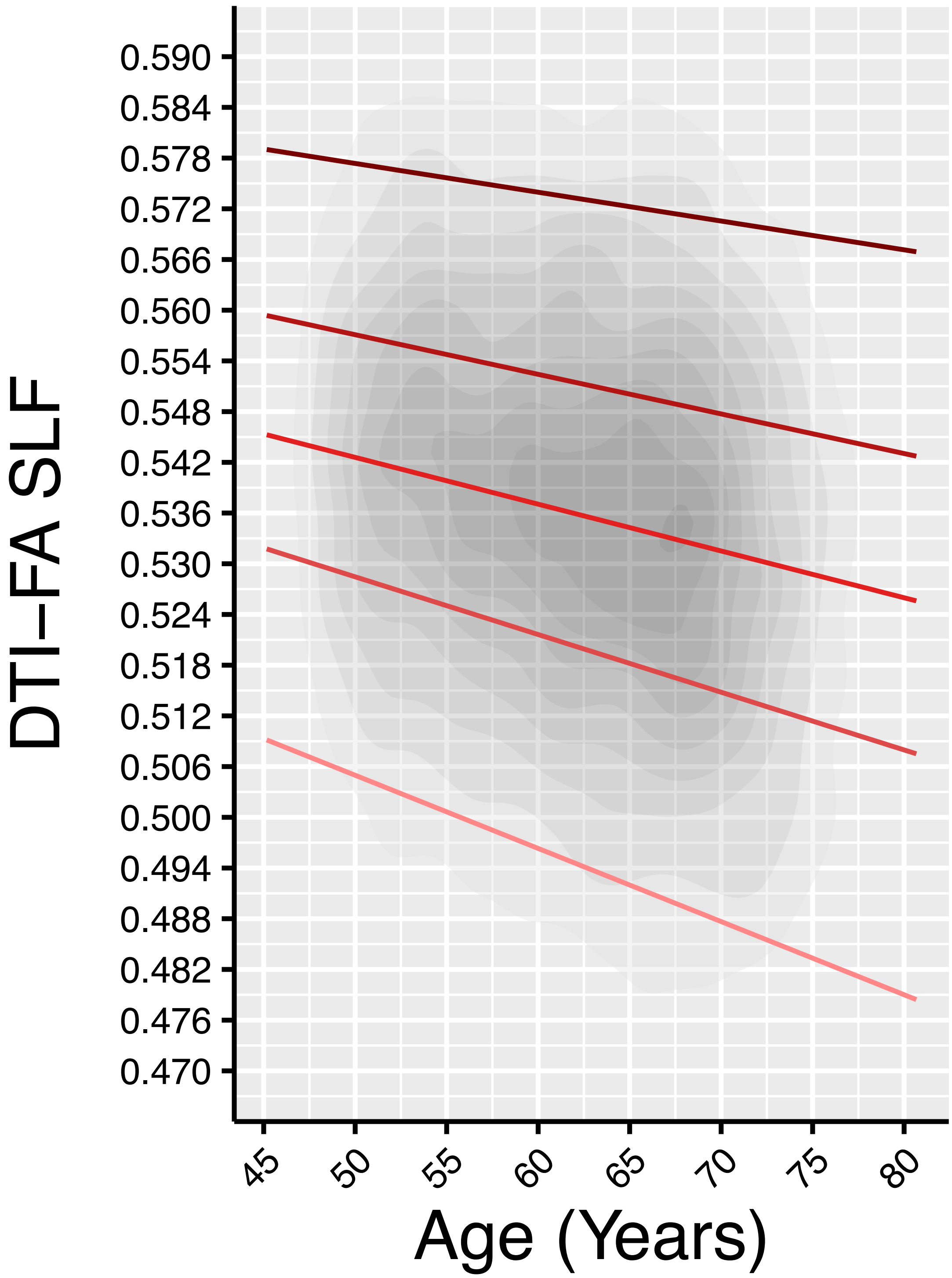

Figure S289. Full size normative centile reference curves calculated for the superior longitudinal fasciculus tract for DTI-FA in females. Solid colored lines, ordered from lightest to darkest, indicate the following centiles: 5th, 25th, 50th, 75th, 95th. Gray overlay reflects kernel density (darker=greater degree of data point overlap). SLF = superior longitudinal fasciculus.

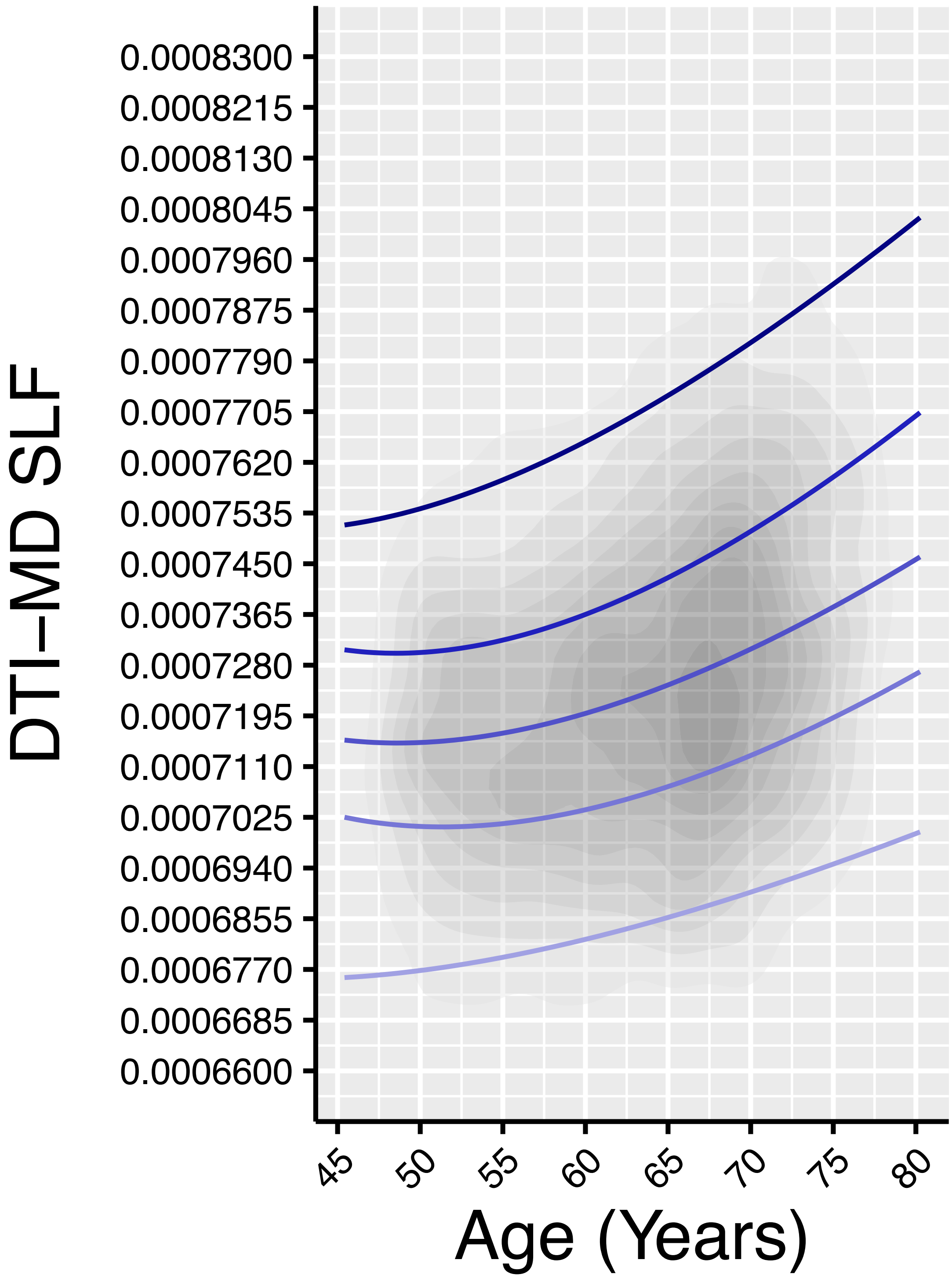

Figure S290. Full size normative centile reference curves calculated for the superior longitudinal fasciculus tract for DTI-MD in males. Solid colored lines, ordered from lightest to darkest, indicate the following centiles: 5th, 25th, 50th, 75th, 95th. Gray overlay reflects kernel density (darker=greater degree of data point overlap). SLF = superior longitudinal fasciculus.

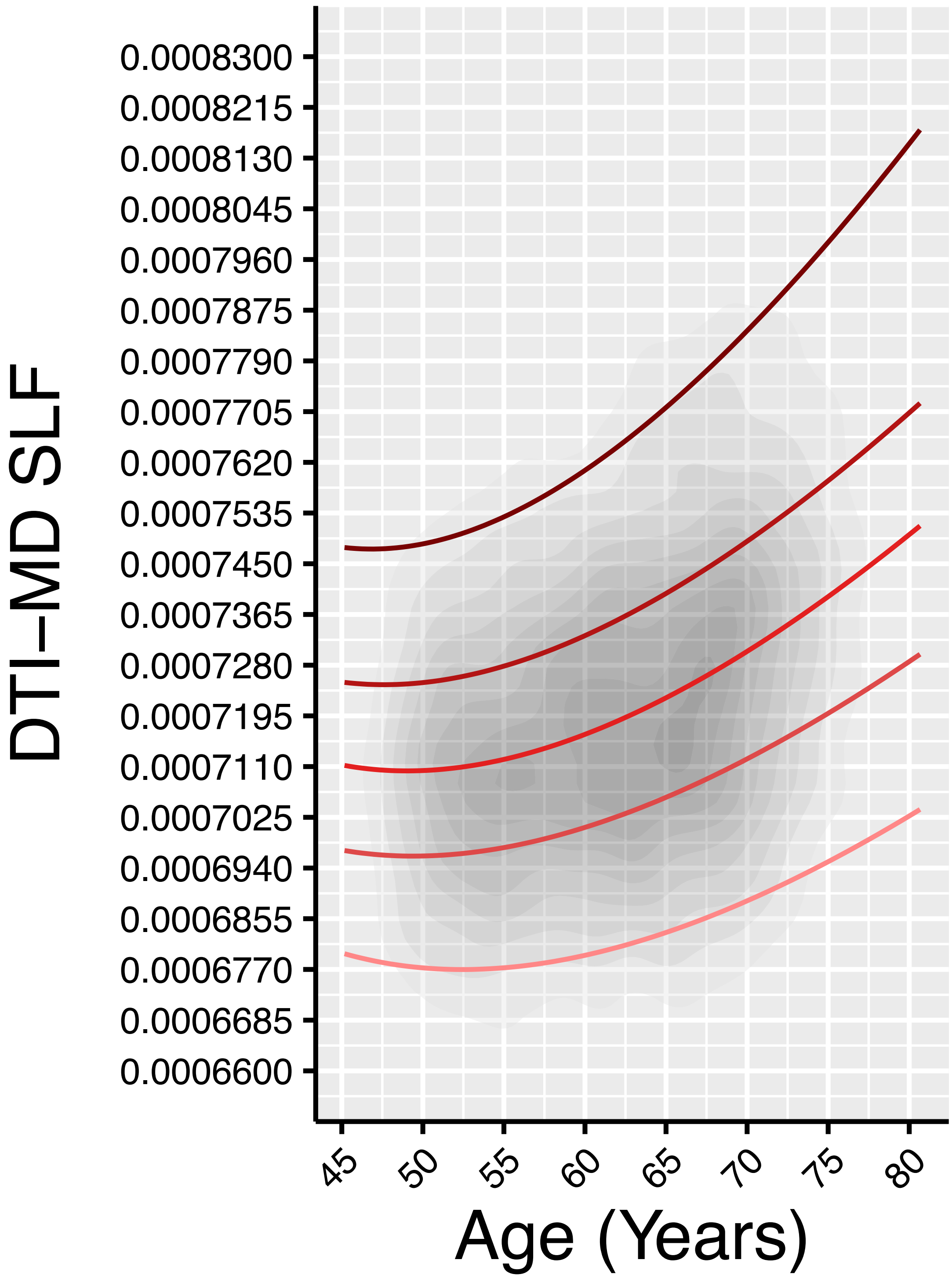

Figure S291. Full size normative centile reference curves calculated for the superior longitudinal fasciculus tract for DTI-MD in females. Solid colored lines, ordered from lightest to darkest, indicate the following centiles: 5th, 25th, 50th, 75th, 95th. Gray overlay reflects kernel density (darker=greater degree of data point overlap). SLF = superior longitudinal fasciculus.

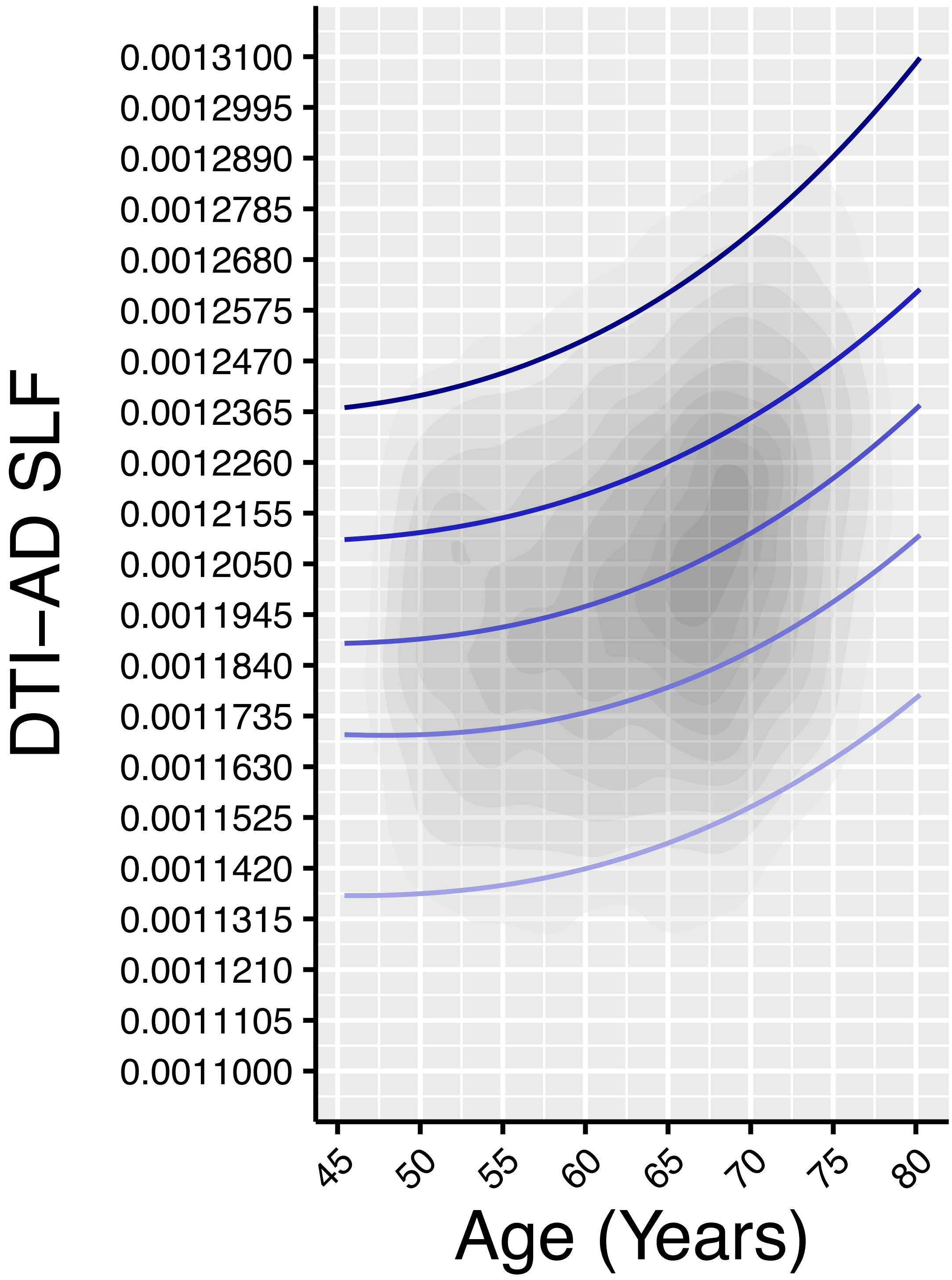

Figure S292. Full size normative centile reference curves calculated for the superior longitudinal fasciculus tract for DTI-AD in males. Solid colored lines, ordered from lightest to darkest, indicate the following centiles: 5th, 25th, 50th, 75th, 95th. Gray overlay reflects kernel density (darker=greater degree of data point overlap). SLF = superior longitudinal fasciculus.

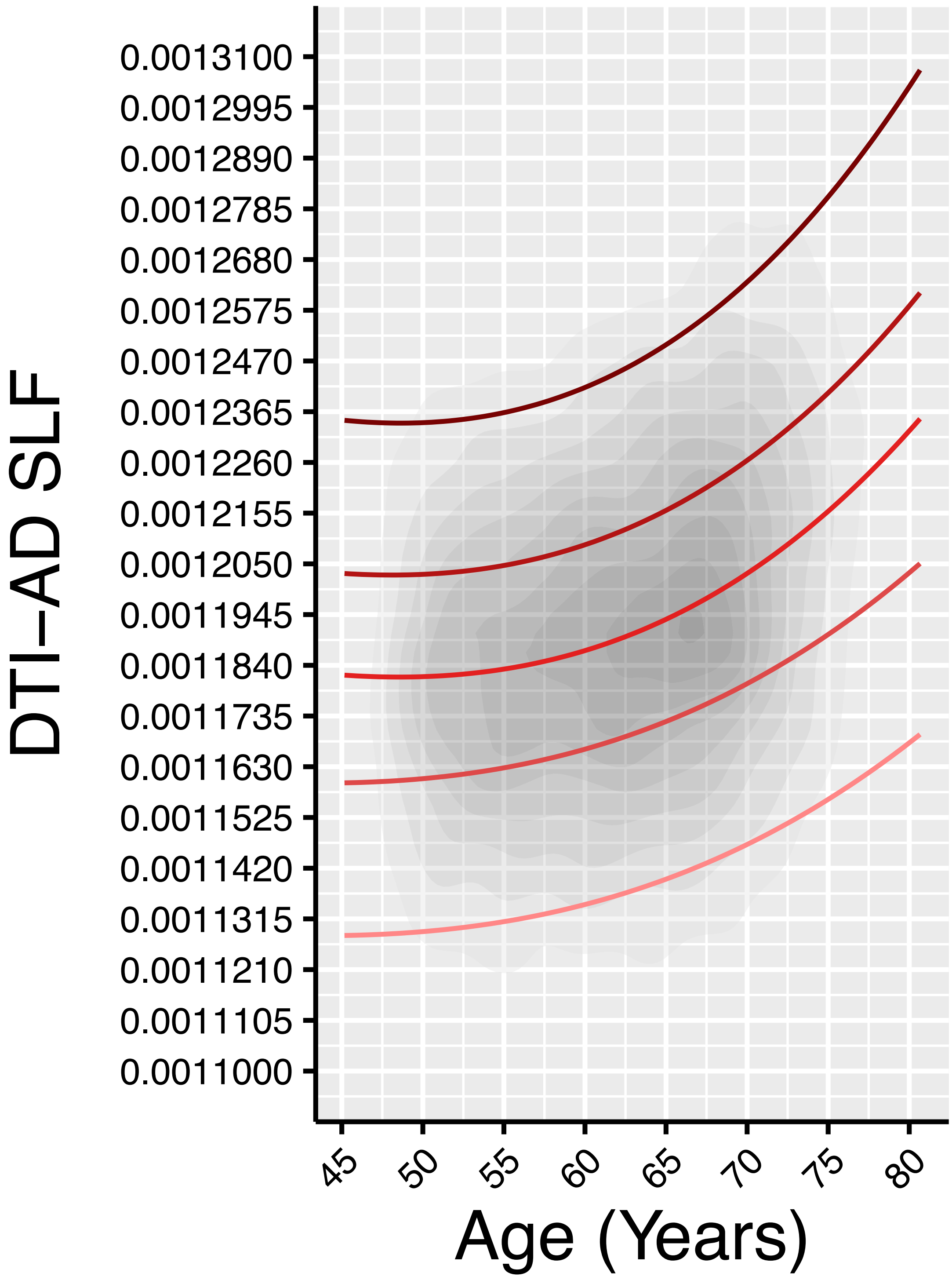

Figure S293. Full size normative centile reference curves calculated for the superior longitudinal fasciculus tract for DTI-AD in females. Solid colored lines, ordered from lightest to darkest, indicate the following centiles: 5th, 25th, 50th, 75th, 95th. Gray overlay reflects kernel density (darker=greater degree of data point overlap). SLF = superior longitudinal fasciculus.

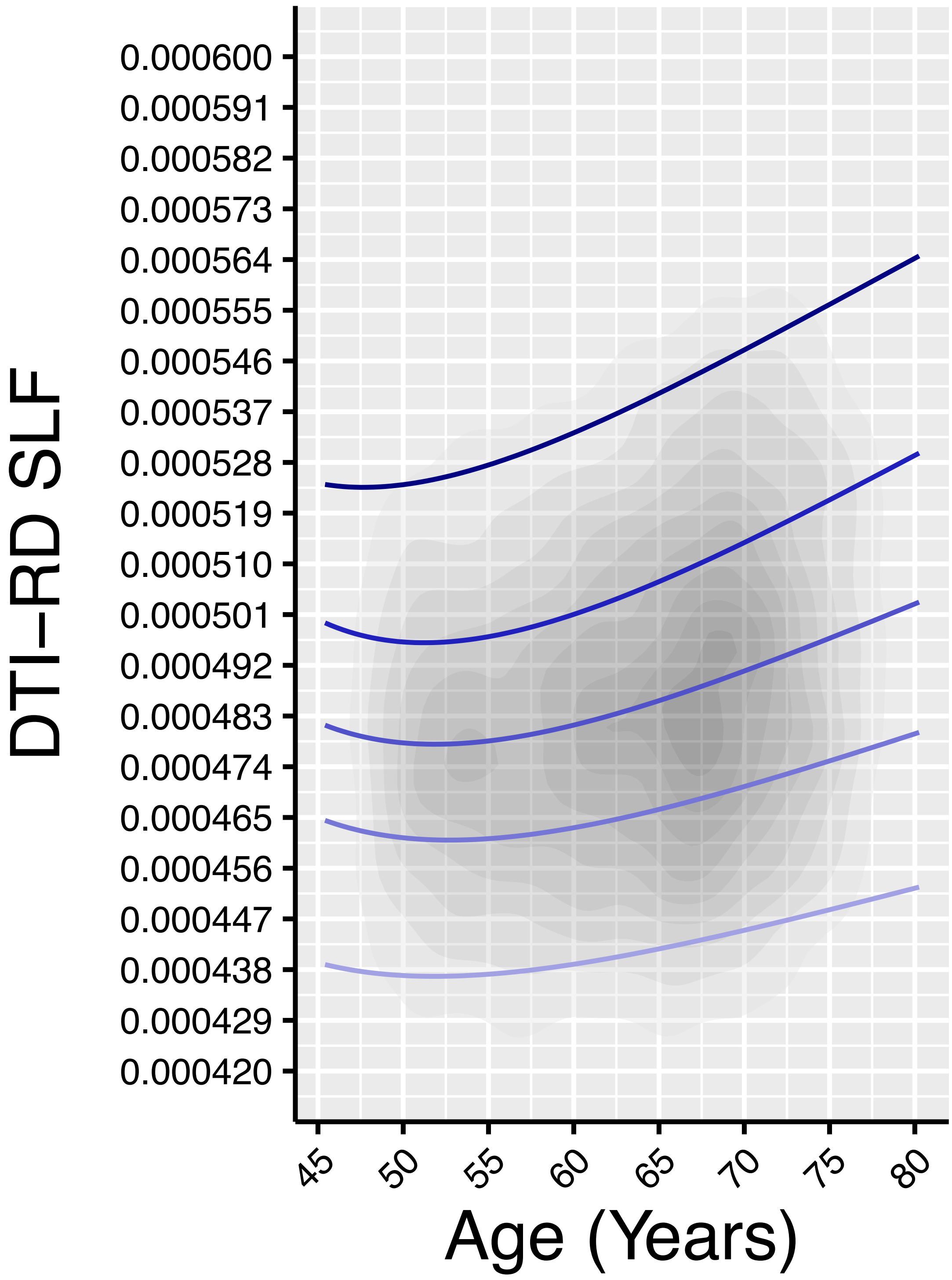

Figure S294. Full size normative centile reference curves calculated for the superior longitudinal fasciculus tract for DTI-RD in males. Solid colored lines, ordered from lightest to darkest, indicate the following centiles: 5th, 25th, 50th, 75th, 95th. Gray overlay reflects kernel density (darker=greater degree of data point overlap). SLF = superior longitudinal fasciculus.

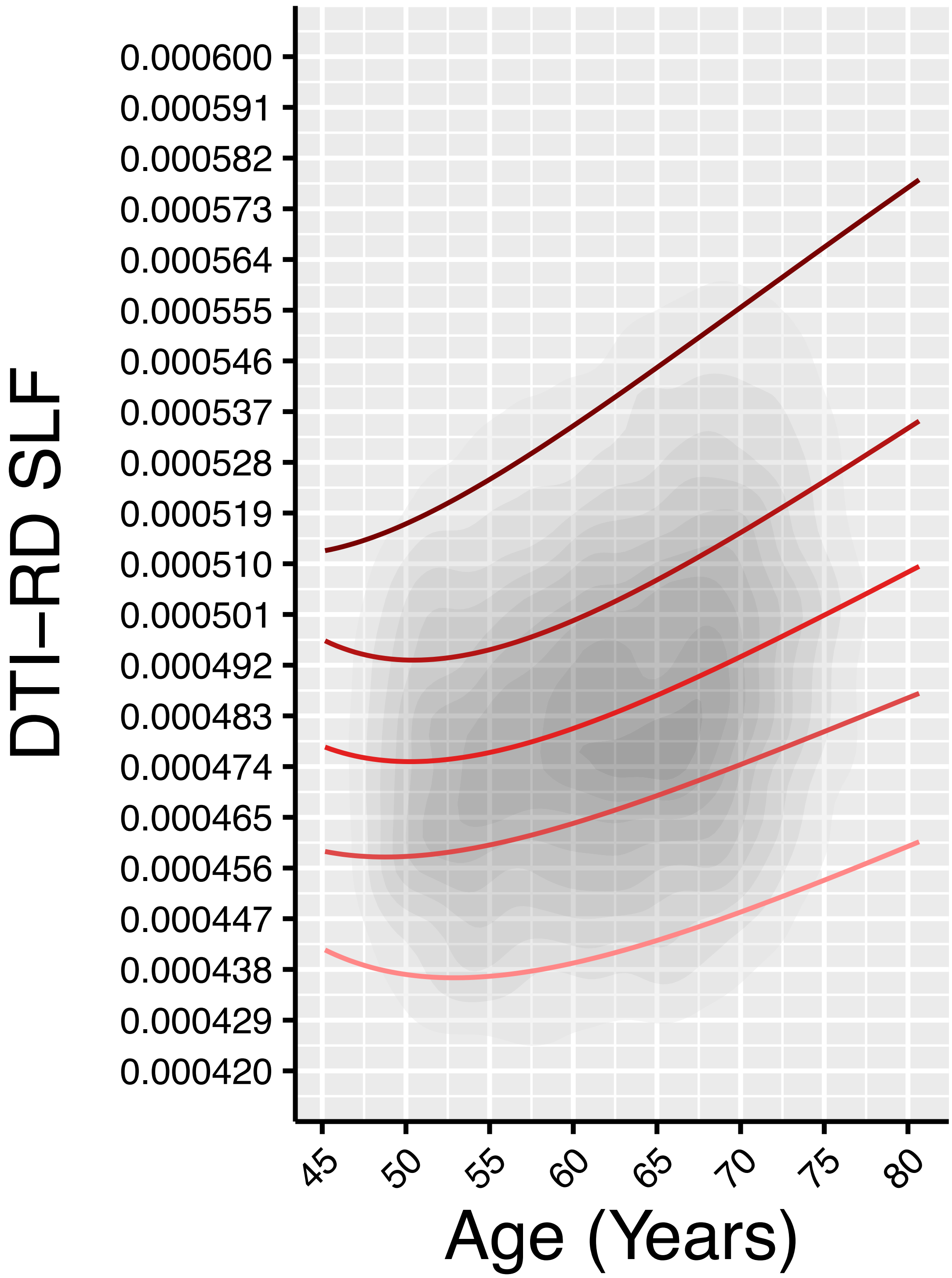

Figure S295. Full size normative centile reference curves calculated for the superior longitudinal fasciculus tract for DTI-RD in females. Solid colored lines, ordered from lightest to darkest, indicate the following centiles: 5th, 25th, 50th, 75th, 95th. Gray overlay reflects kernel density (darker=greater degree of data point overlap). SLF = superior longitudinal fasciculus.

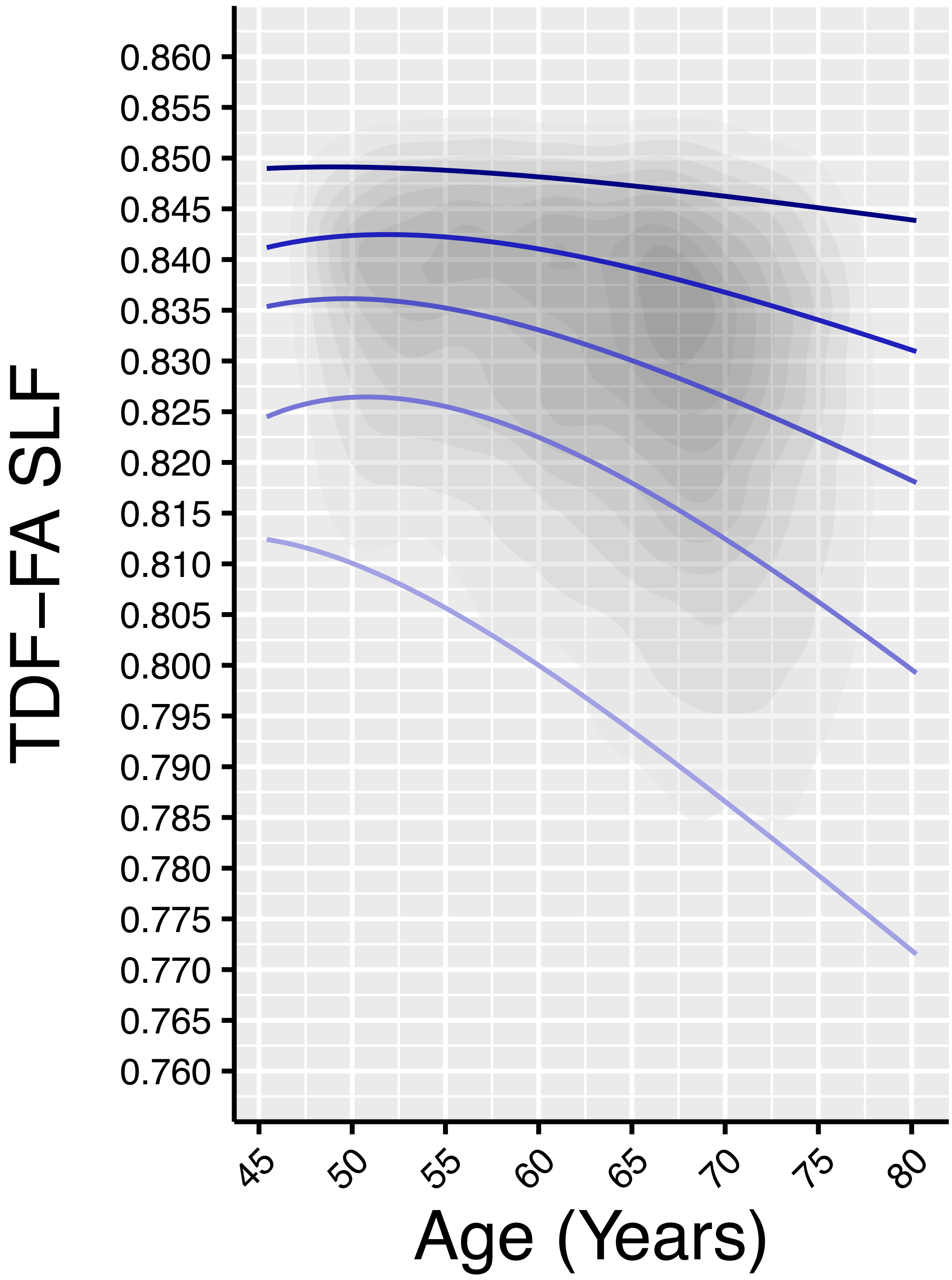

Figure S296. Full size normative centile reference curves calculated for the superior longitudinal fasciculus tract for TDF-FA in males. Solid colored lines, ordered from lightest to darkest, indicate the following centiles: 5th, 25th, 50th, 75th, 95th. Gray overlay reflects kernel density (darker=greater degree of data point overlap). SLF = superior longitudinal fasciculus.

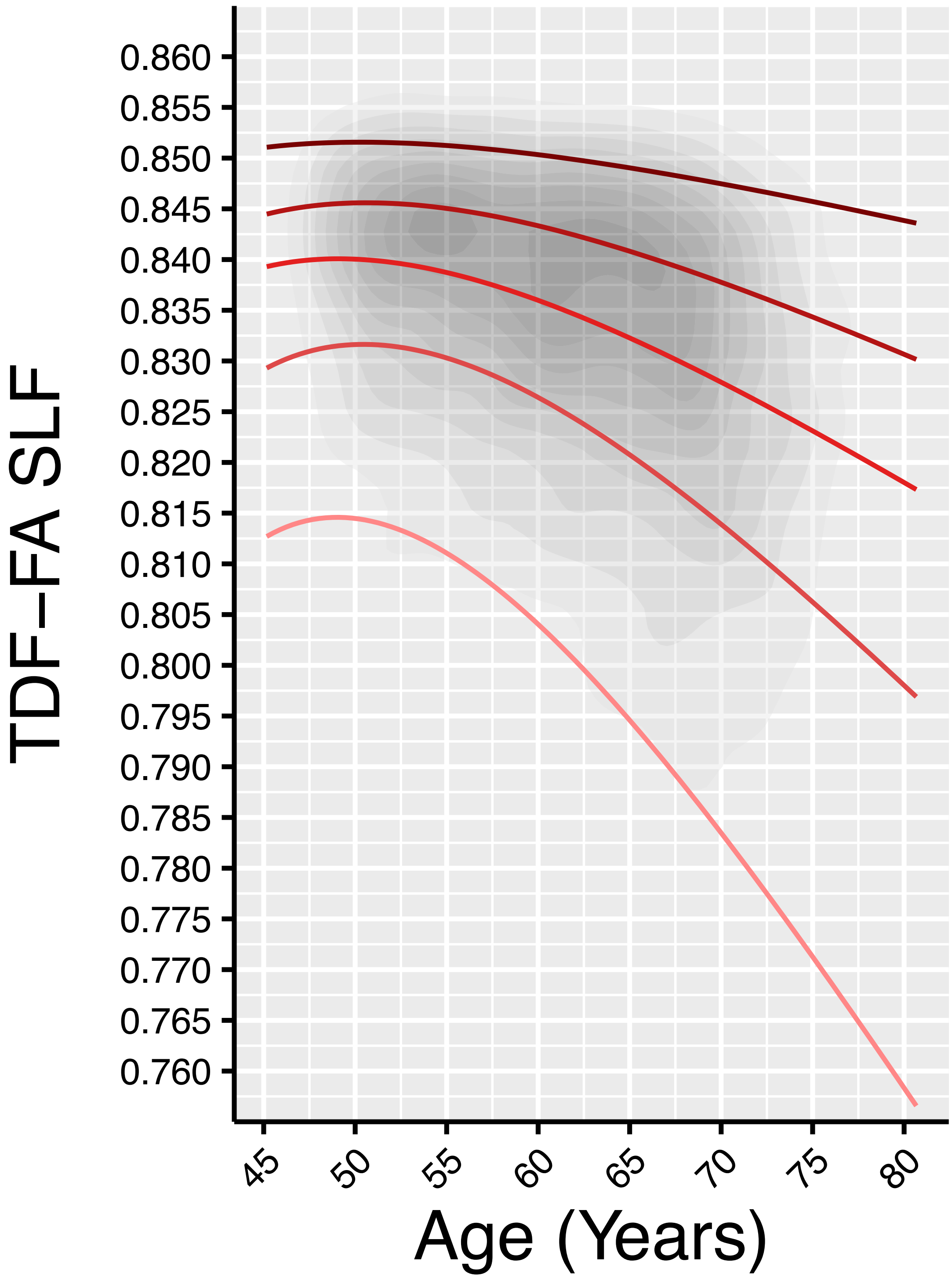

Figure S297. Full size normative centile reference curves calculated for the superior longitudinal fasciculus tract for TDF-FA in females. Solid colored lines, ordered from lightest to darkest, indicate the following centiles: 5th, 25th, 50th, 75th, 95th. Gray overlay reflects kernel density (darker=greater degree of data point overlap). SLF = superior longitudinal fasciculus.

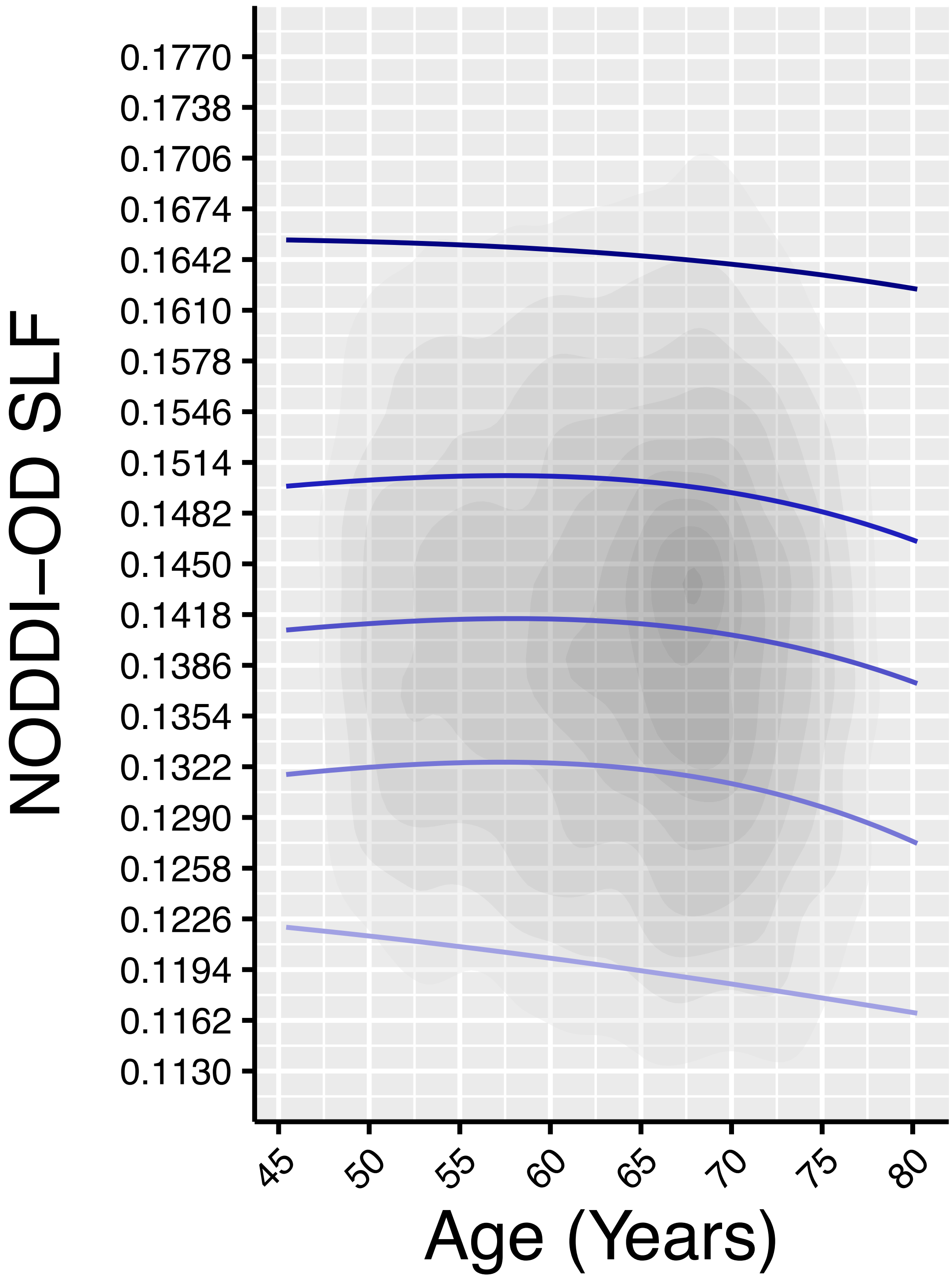

Figure S298. Full size normative centile reference curves calculated for the superior longitudinal fasciculus tract for NODDI-OD in males. Solid colored lines, ordered from lightest to darkest, indicate the following centiles: 5th, 25th, 50th, 75th, 95th. Gray overlay reflects kernel density (darker=greater degree of data point overlap). SLF = superior longitudinal fasciculus.

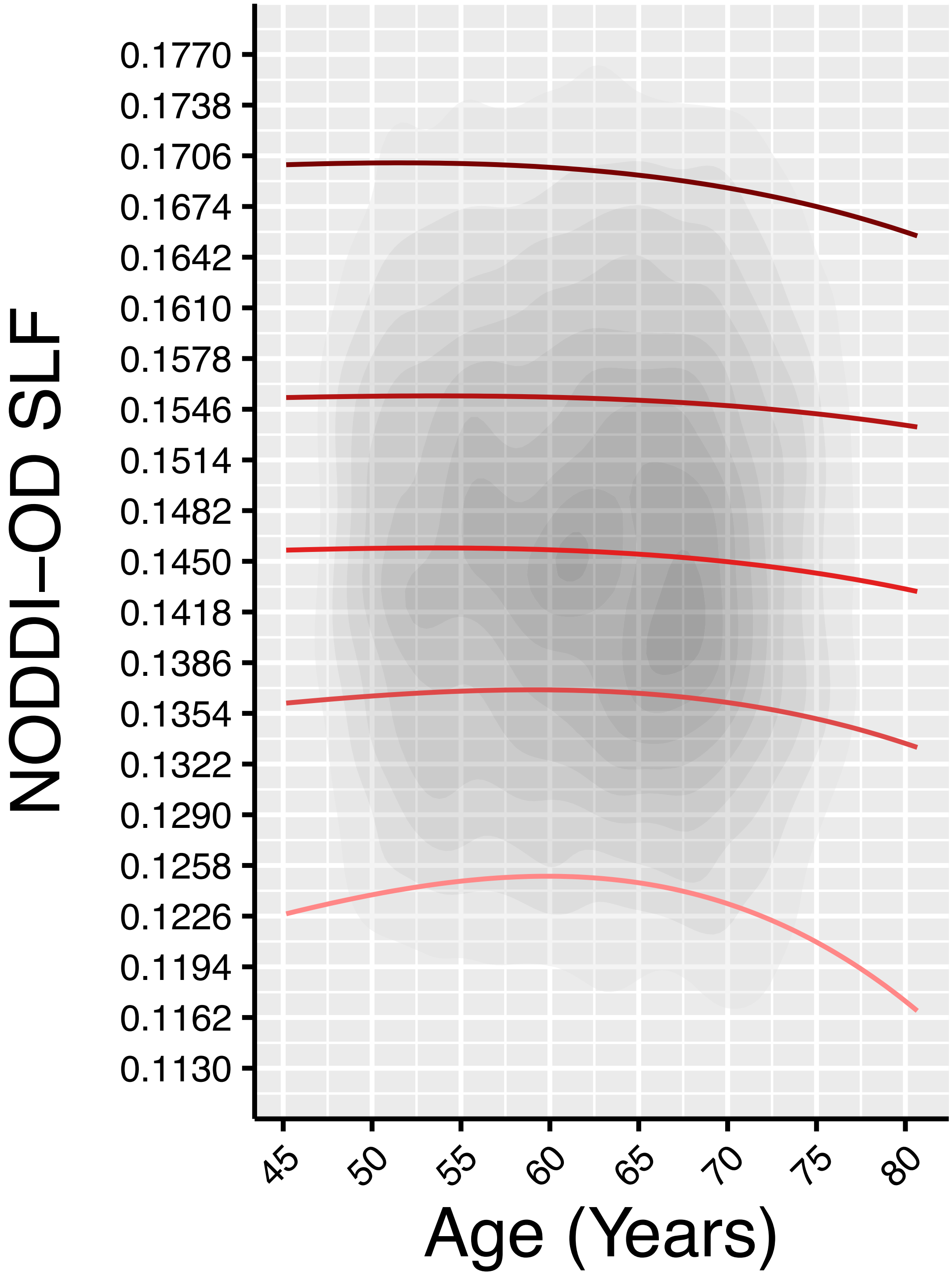

Figure S299. Full size normative centile reference curves calculated for the superior longitudinal fasciculus tract for NODDI-OD in females. Solid colored lines, ordered from lightest to darkest, indicate the following centiles: 5th, 25th, 50th, 75th, 95th. Gray overlay reflects kernel density (darker=greater degree of data point overlap). SLF = superior longitudinal fasciculus.

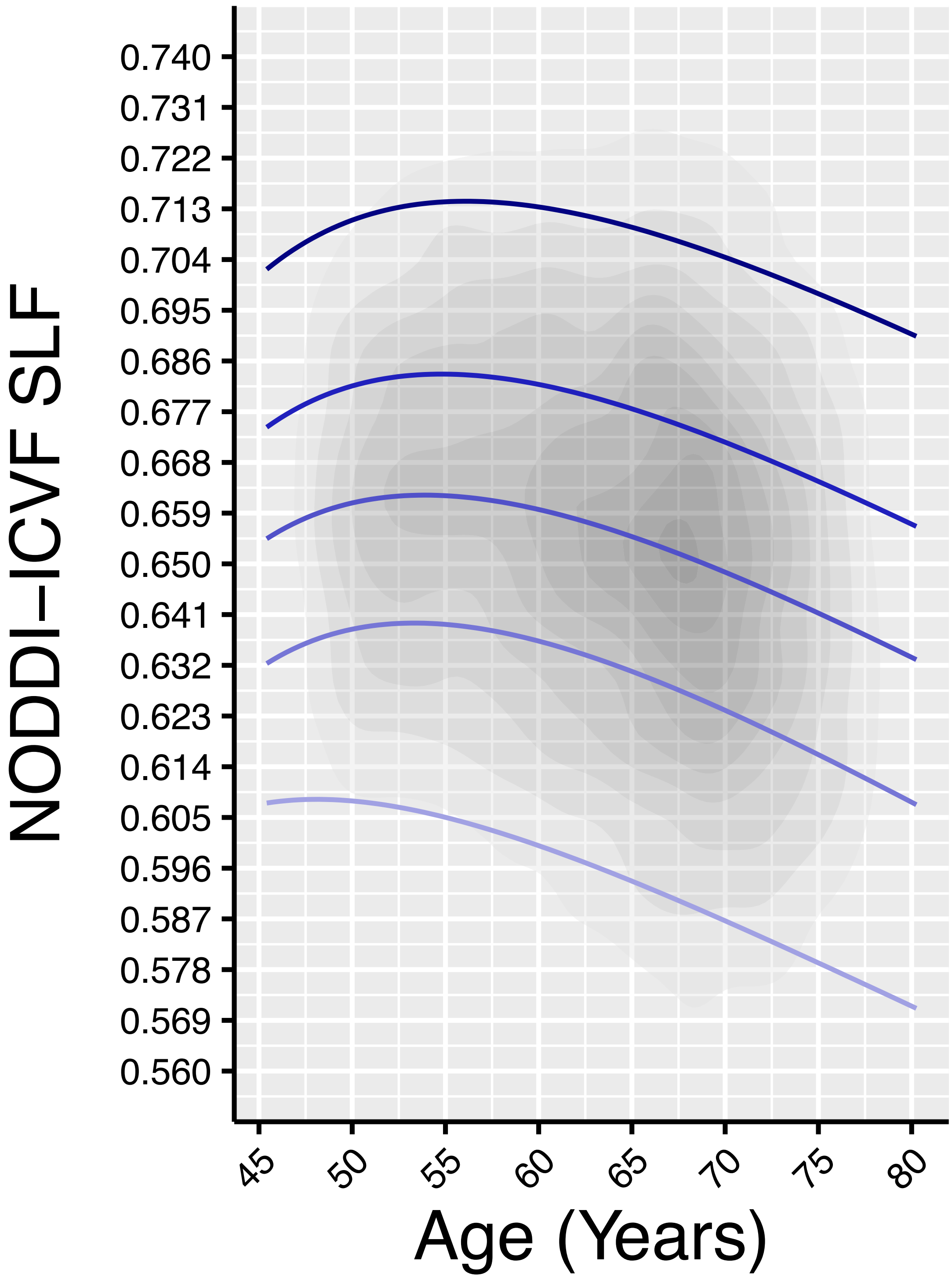

Figure S300. Full size normative centile reference curves calculated for the superior longitudinal fasciculus tract for NODDI-ICVF in males. Solid colored lines, ordered from lightest to darkest, indicate the following centiles: 5th, 25th, 50th, 75th, 95th. Gray overlay reflects kernel density (darker=greater degree of data point overlap). SLF = superior longitudinal fasciculus.

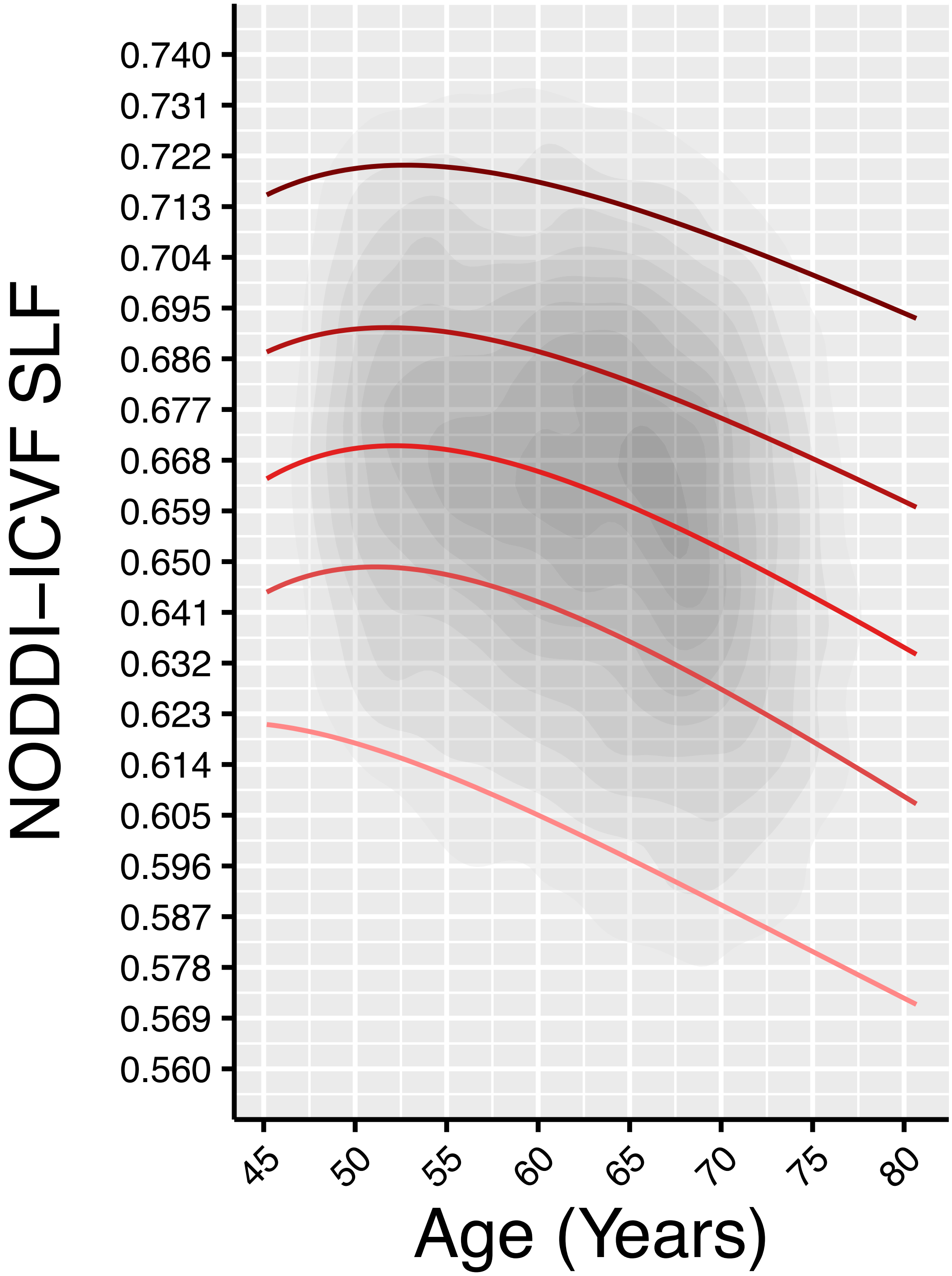

Figure S301. Full size normative centile reference curves calculated for the superior longitudinal fasciculus tract for NODDI-ICVF in females. Solid colored lines, ordered from lightest to darkest, indicate the following centiles: 5th, 25th, 50th, 75th, 95th. Gray overlay reflects kernel density (darker=greater degree of data point overlap). SLF = superior longitudinal fasciculus.

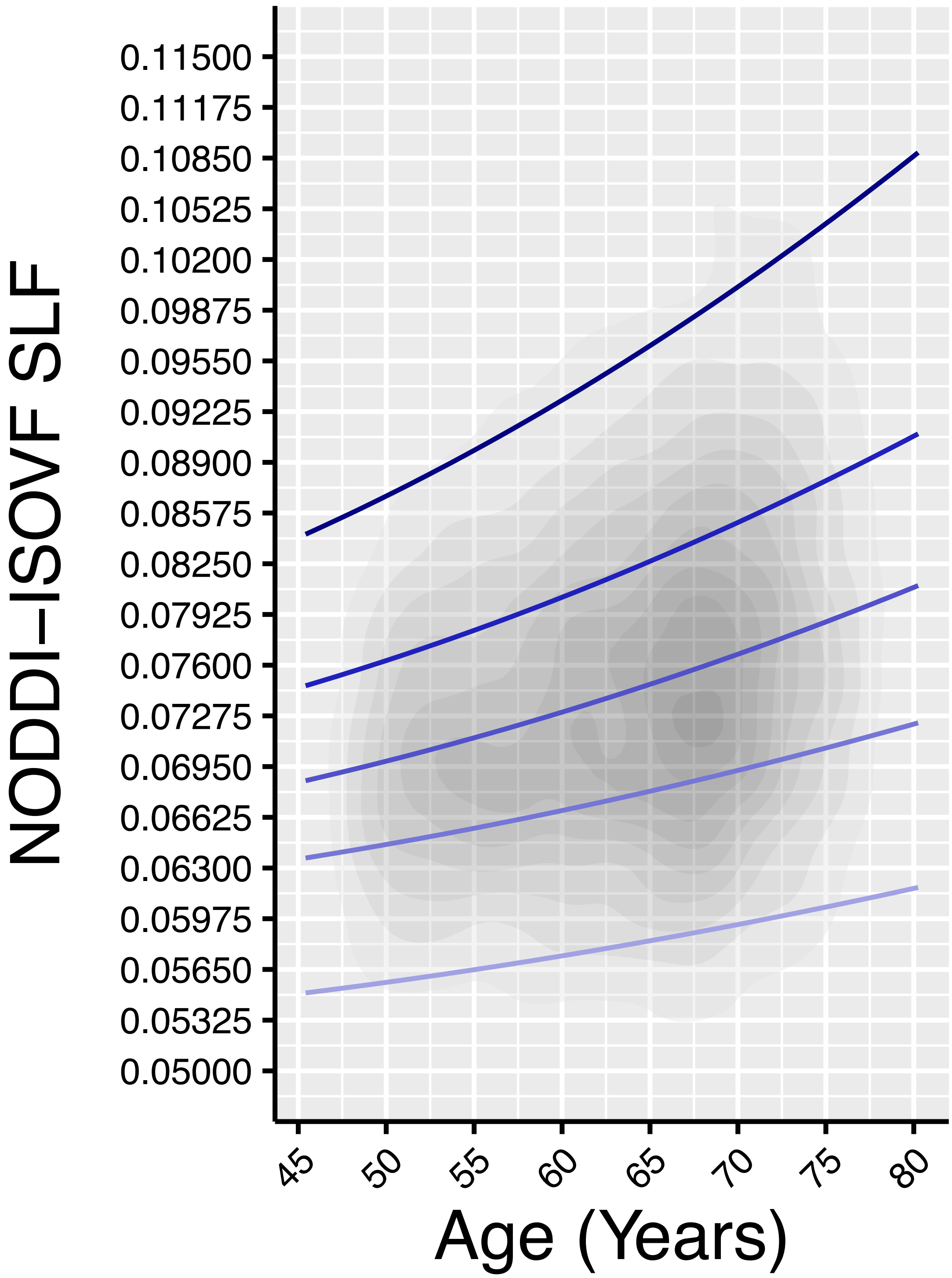

Figure S302. Full size normative centile reference curves calculated for the superior longitudinal fasciculus tract for NODDI-ISOVF in males. Solid colored lines, ordered from lightest to darkest, indicate the following centiles: 5th, 25th, 50th, 75th, 95th. Gray overlay reflects kernel density (darker=greater degree of data point overlap). SLF = superior longitudinal fasciculus.

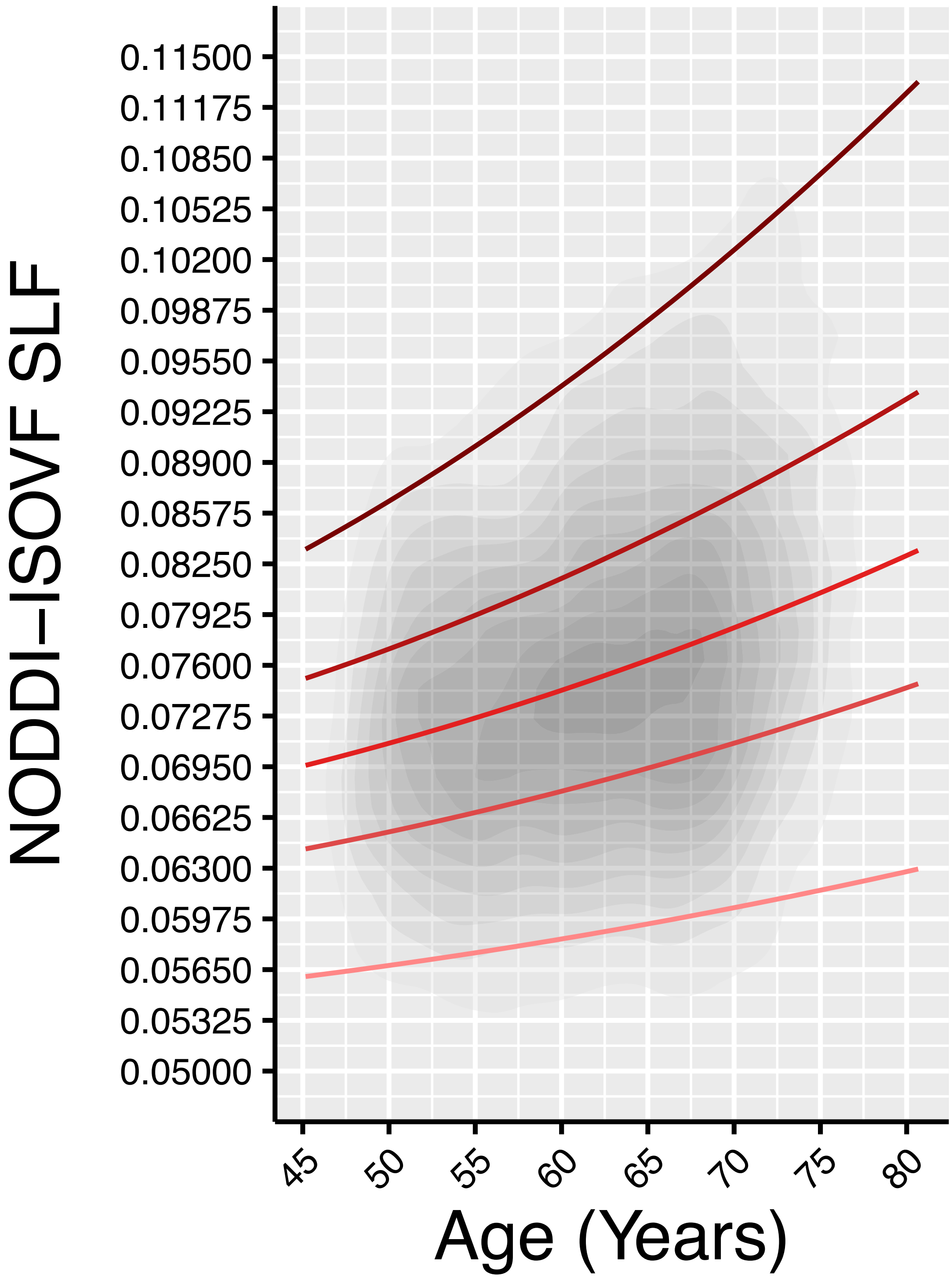

Figure S303 . Full size normative centile reference curves calculated for the superior longitudinal fasciculus tract for NODDI-ISOVF in females. Solid colored lines, ordered from lightest to darkest, indicate the following centiles: 5th, 25th, 50th, 75th, 95th. Gray overlay reflects kernel density (darker=greater degree of data point overlap). SLF = superior longitudinal fasciculus.

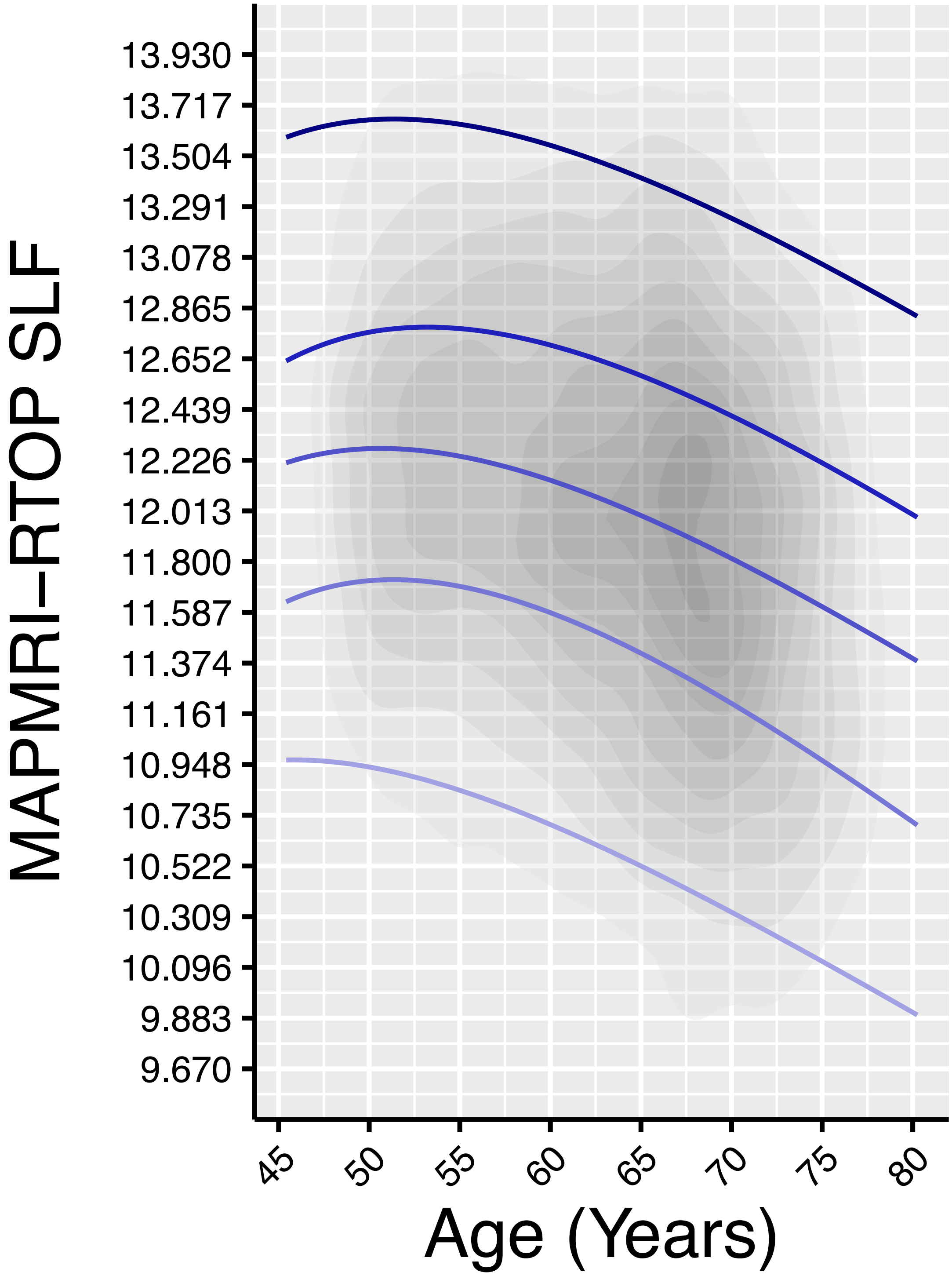

Figure S304. Full size normative centile reference curves calculated for the superior longitudinal fasciculus tract for MAPMRI-RTOP in males. Solid colored lines, ordered from lightest to darkest, indicate the following centiles: 5th, 25th, 50th, 75th, 95th. Gray overlay reflects kernel density (darker=greater degree of data point overlap). SLF = superior longitudinal fasciculus.

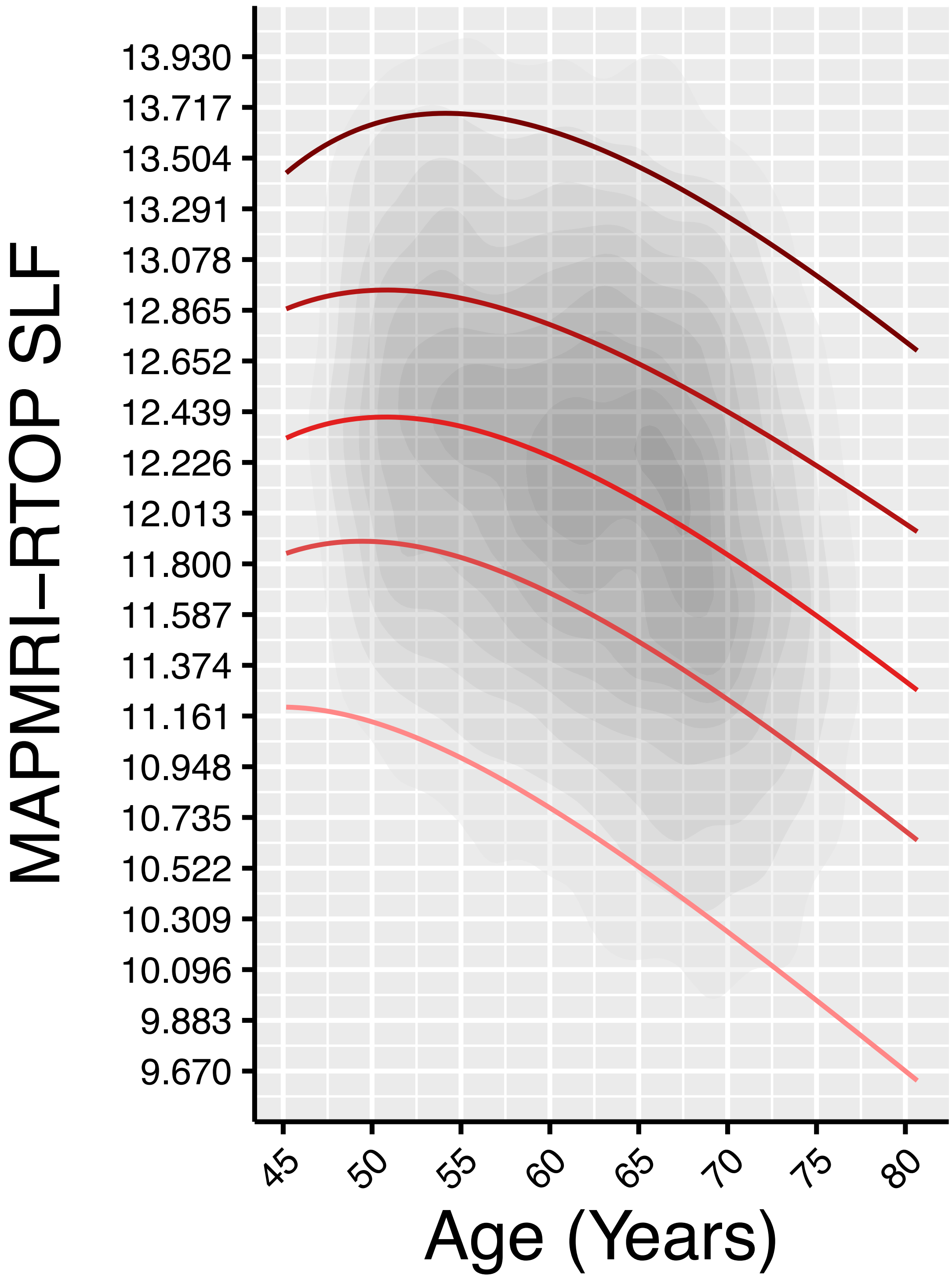

Figure S305. Full size normative centile reference curves calculated for the superior longitudinal fasciculus tract for MAPMRI-RTOP in females. Solid colored lines, ordered from lightest to darkest, indicate the following centiles: 5th, 25th, 50th, 75th, 95th. Gray overlay reflects kernel density (darker=greater degree of data point overlap). SLF = superior longitudinal fasciculus.

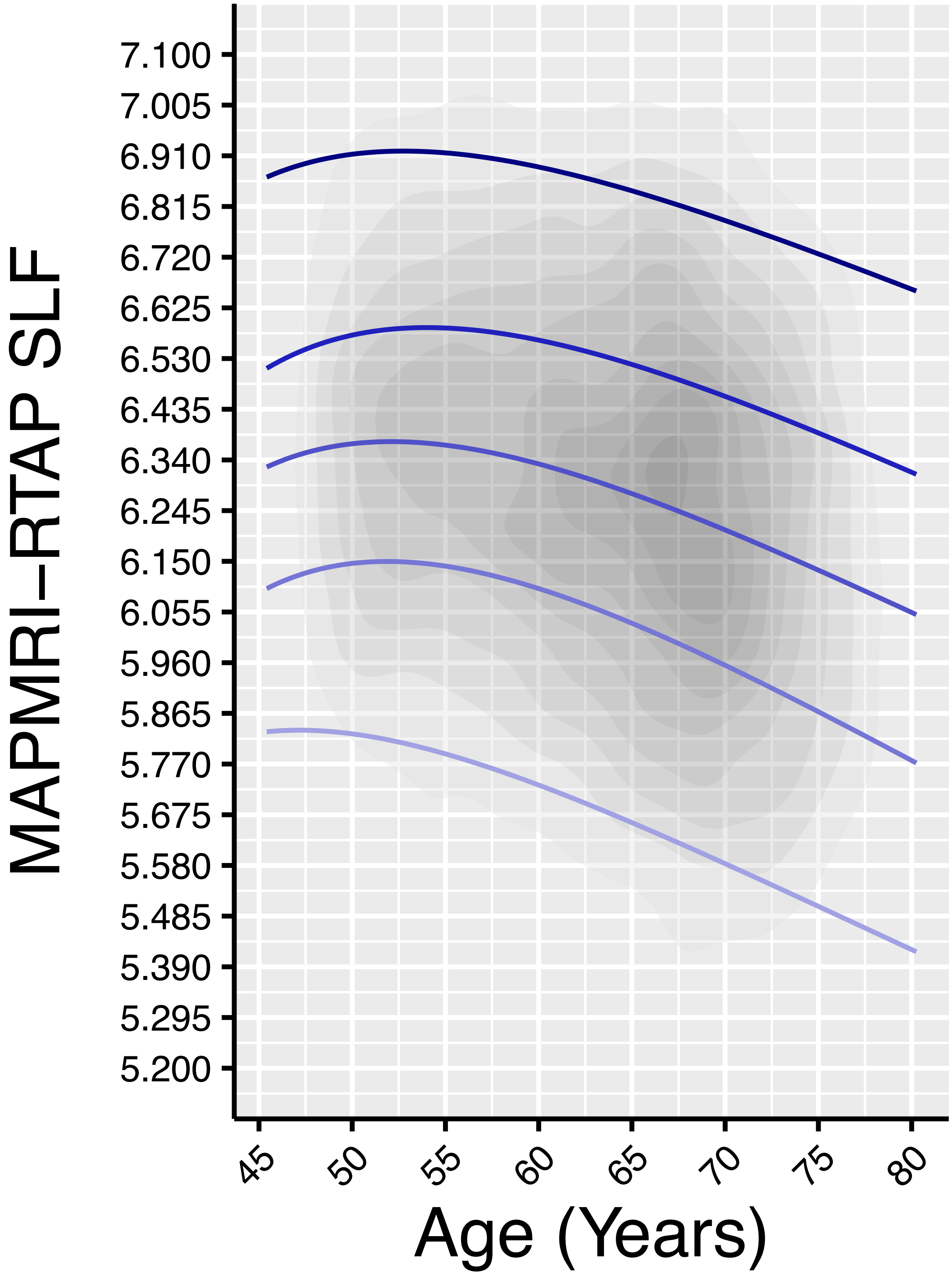

Figure S306. Full size normative centile reference curves calculated for the superior longitudinal fasciculus tract for MAPMRI-RTAP in males. Solid colored lines, ordered from lightest to darkest, indicate the following centiles: 5th, 25th, 50th, 75th, 95th. Gray overlay reflects kernel density (darker=greater degree of data point overlap). SLF = superior longitudinal fasciculus.

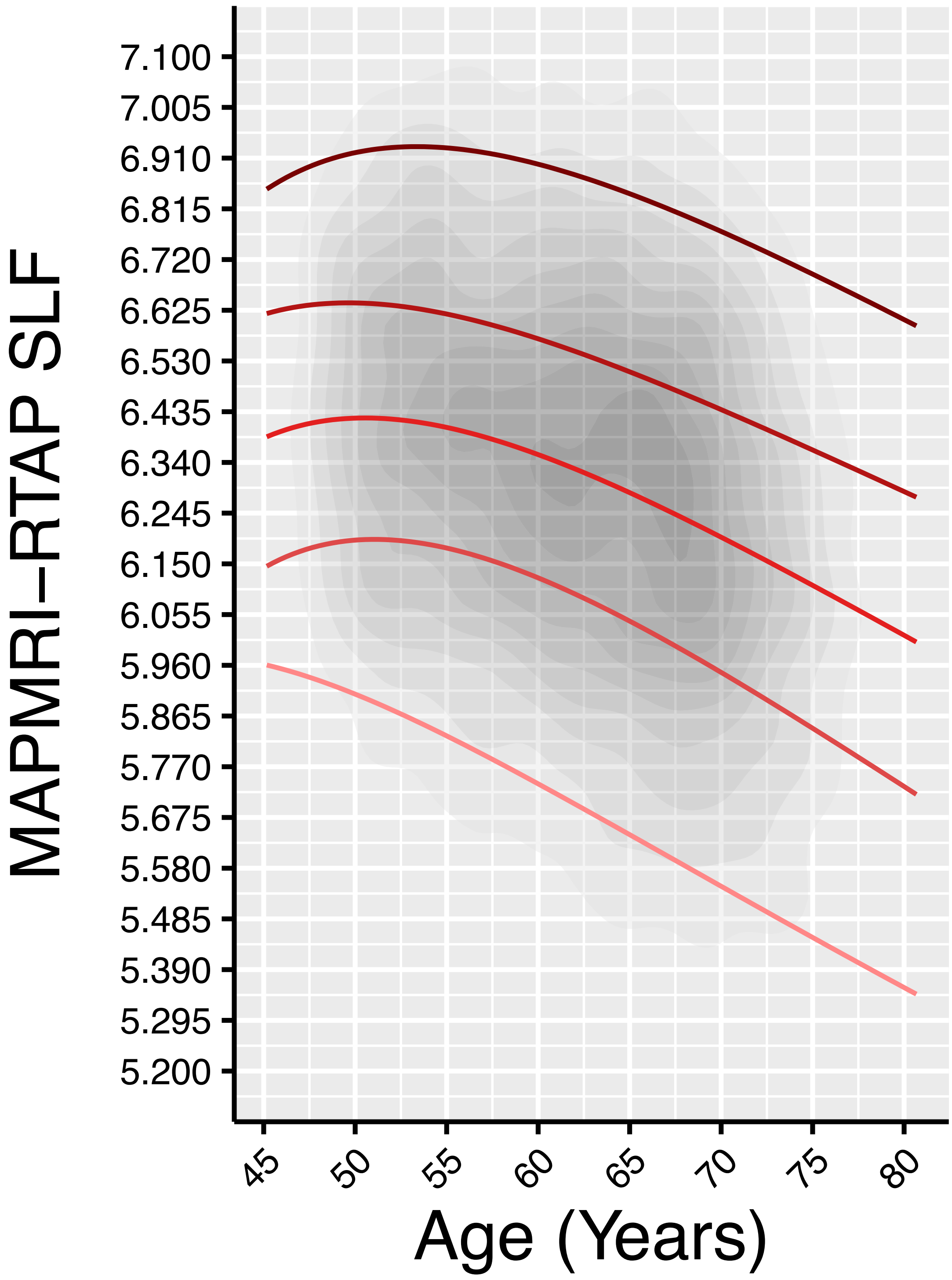

Figure S307. Full size normative centile reference curves calculated for the superior longitudinal fasciculus tract for MAPMRI-RTAP in females. Solid colored lines, ordered from lightest to darkest, indicate the following centiles: 5th, 25th, 50th, 75th, 95th. Gray overlay reflects kernel density (darker=greater degree of data point overlap). SLF = superior longitudinal fasciculus.

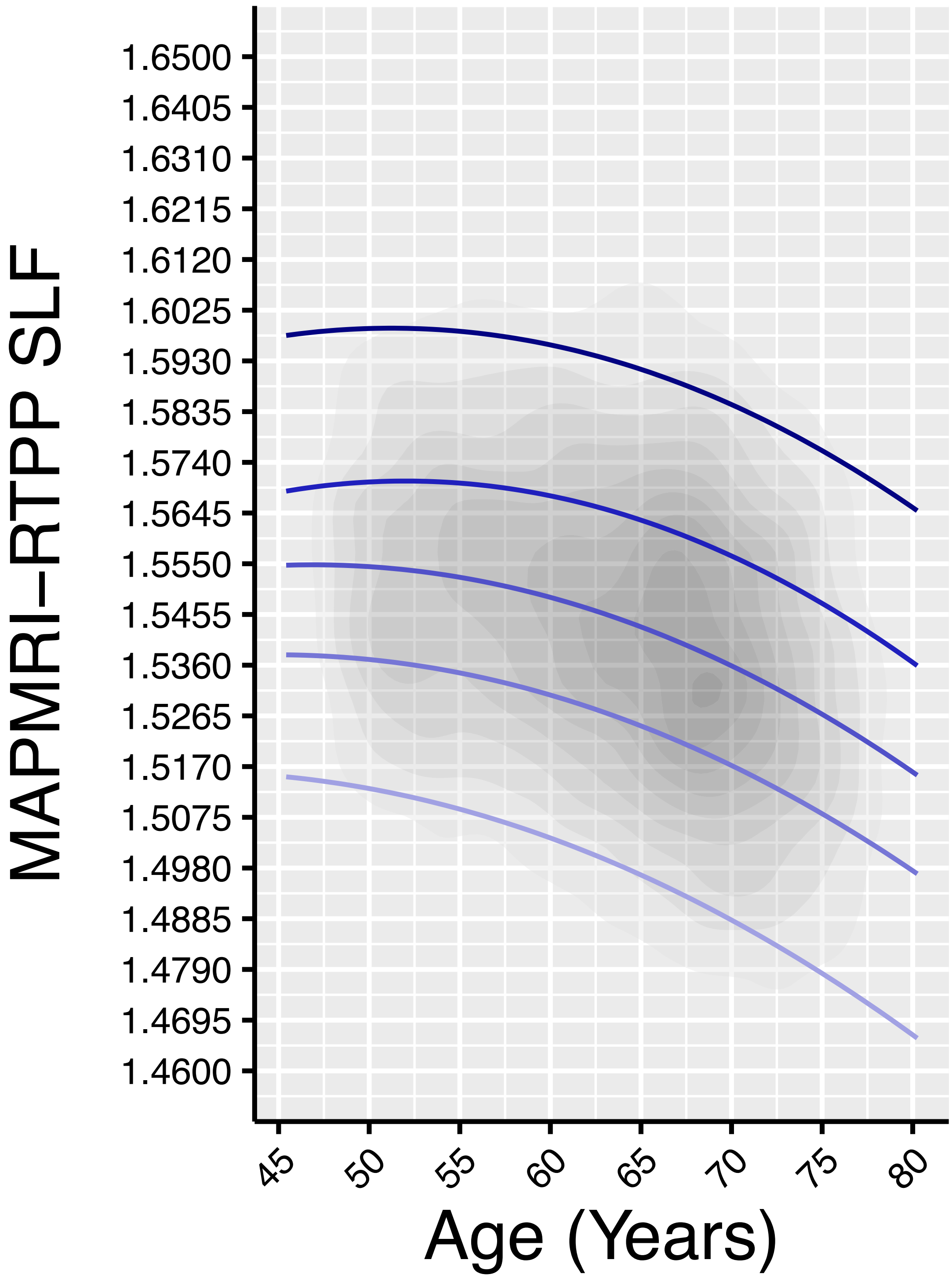

Figure S308. Full size normative centile reference curves calculated for the superior longitudinal fasciculus tract for MAPMRI-RTPP in males. Solid colored lines, ordered from lightest to darkest, indicate the following centiles: 5th, 25th, 50th, 75th, 95th. Gray overlay reflects kernel density (darker=greater degree of data point overlap). SLF = superior longitudinal fasciculus.

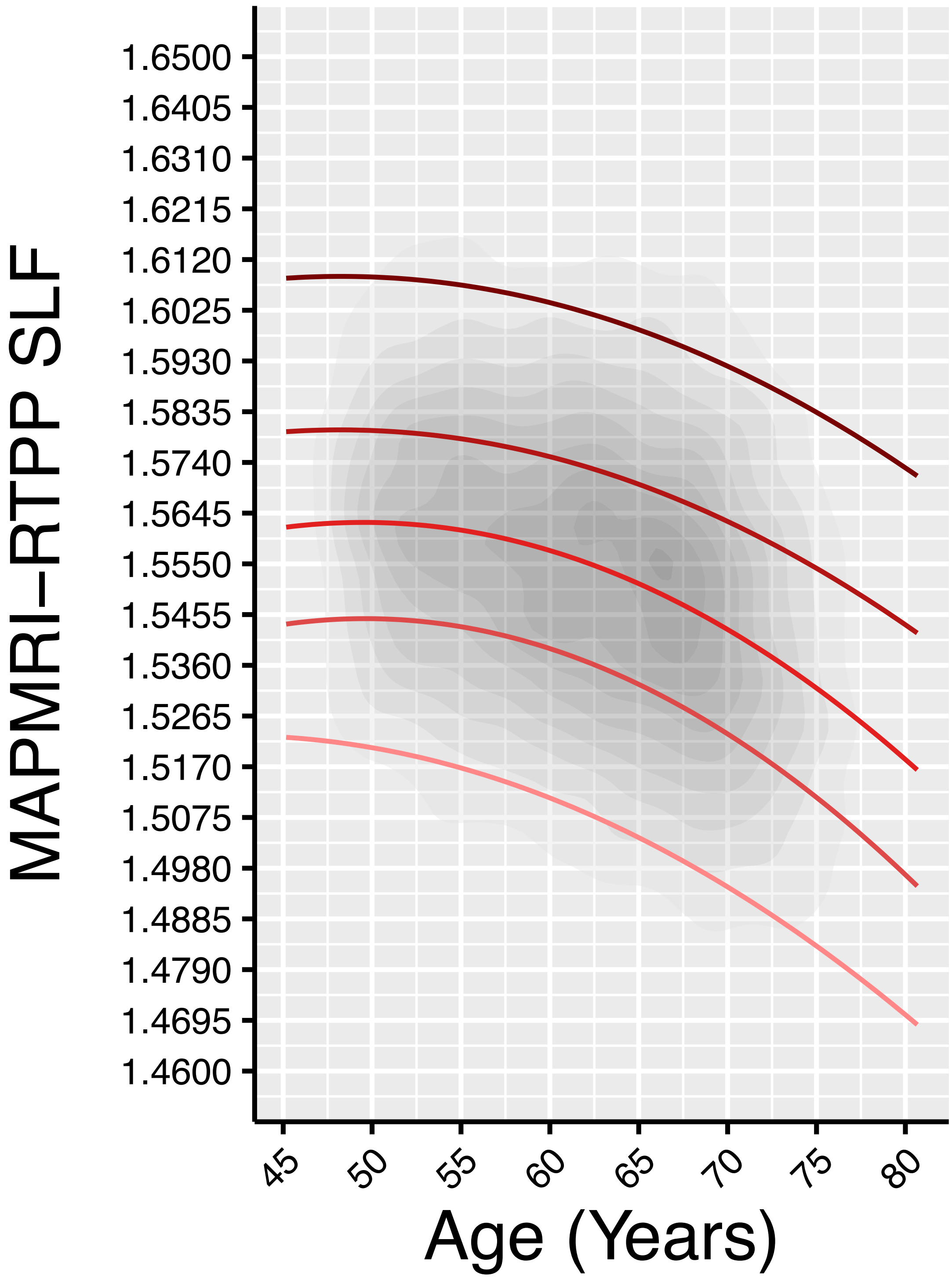

Figure S309. Full size normative centile reference curves calculated for the superior longitudinal fasciculus tract for MAPMRI-RTPP in females. Solid colored lines, ordered from lightest to darkest, indicate the following centiles: 5th, 25th, 50th, 75th, 95th. Gray overlay reflects kernel density (darker=greater degree of data point overlap). SLF = superior longitudinal fasciculus.

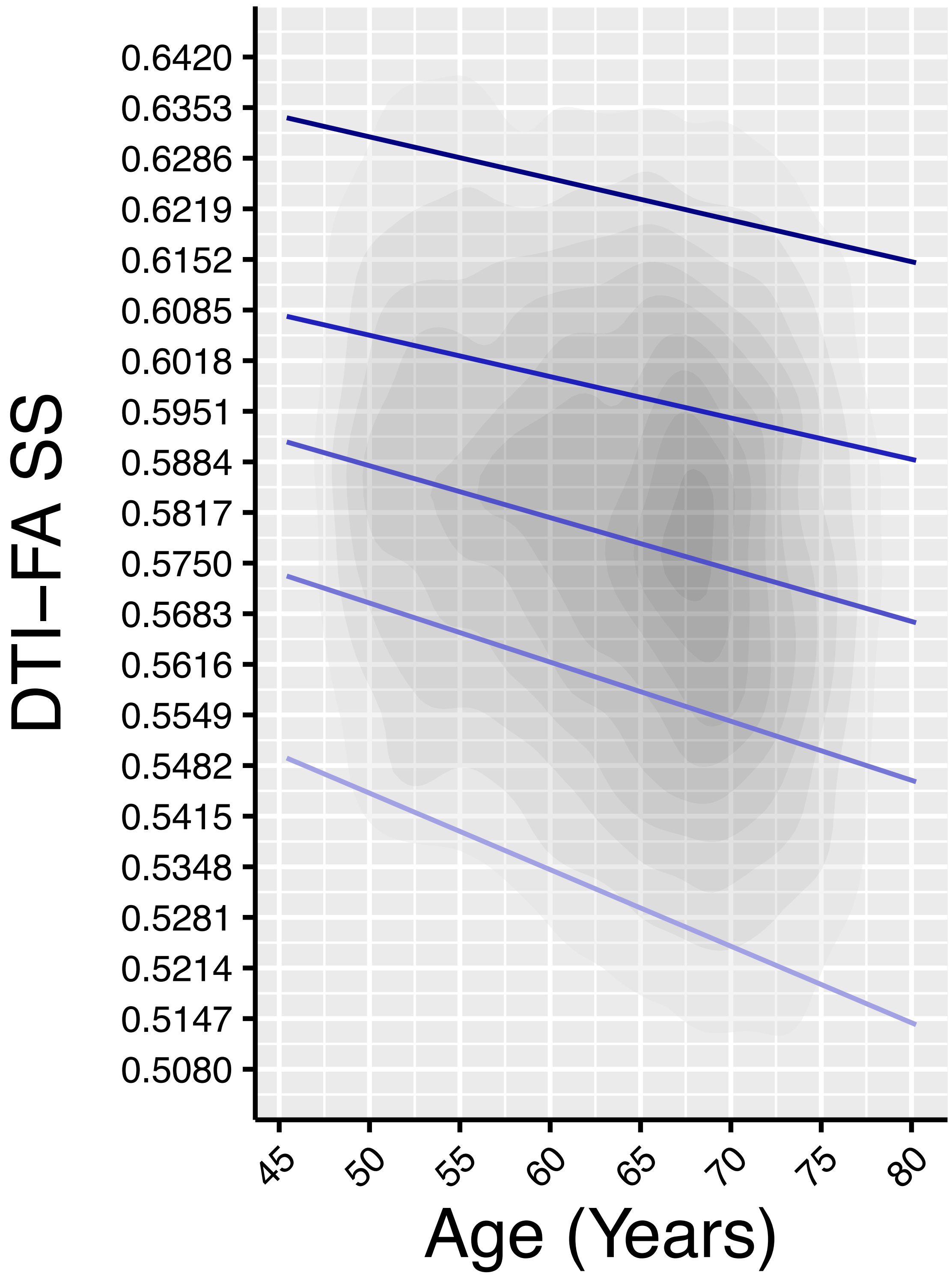

Figure S310. Full size normative centile reference curves calculated for the *sagittal stratum* tract for DTI-FA in males. Solid colored lines, ordered from lightest to darkest, indicate the following centiles: 5th, 25th, 50th, 75th, 95th. Gray overlay reflects kernel density (darker=greater degree of data point overlap). SS = *sagittal stratum.*

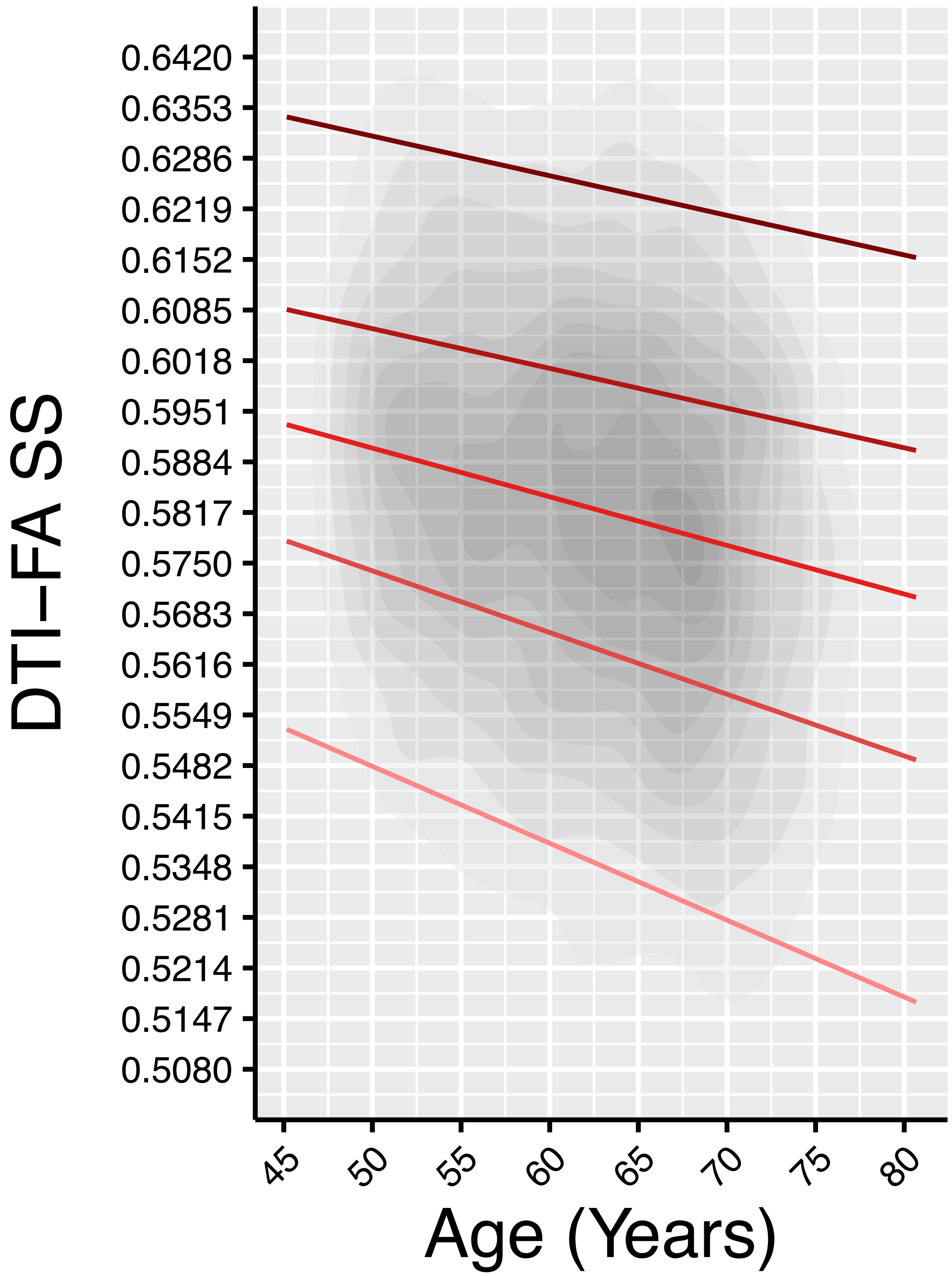

Figure S311. Full size normative centile reference curves calculated for the *sagittal stratum* tract for DTI-FA in females. Solid colored lines, ordered from lightest to darkest, indicate the following centiles: 5th, 25th, 50th, 75th, 95th. Gray overlay reflects kernel density (darker=greater degree of data point overlap). SS = *sagittal stratum.*

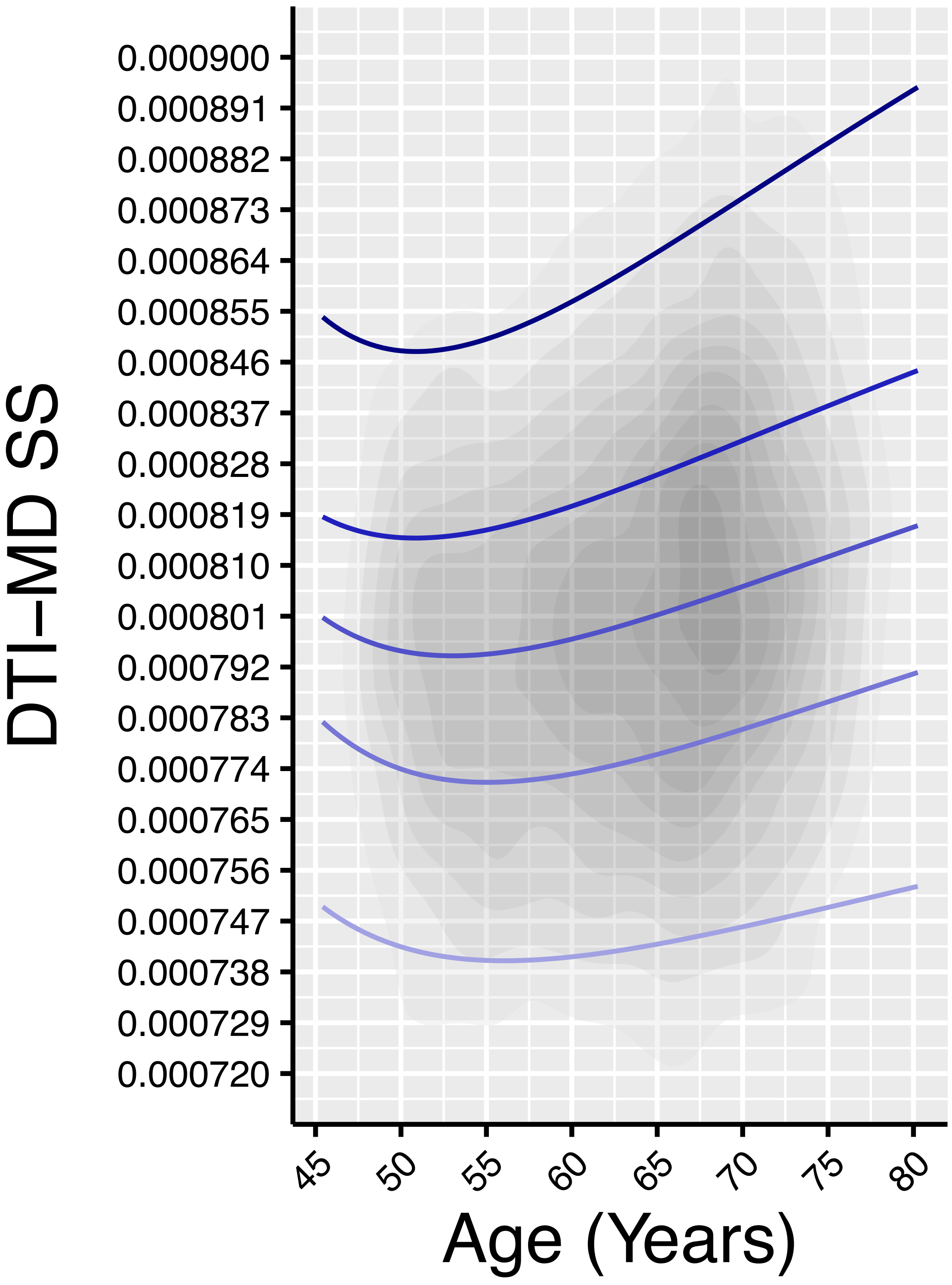

Figure S312. Full size normative centile reference curves calculated for the *sagittal stratum* tract for DTI-MD in males. Solid colored lines, ordered from lightest to darkest, indicate the following centiles: 5th, 25th, 50th, 75th, 95th. Gray overlay reflects kernel density (darker=greater degree of data point overlap). SS = *sagittal stratum.*

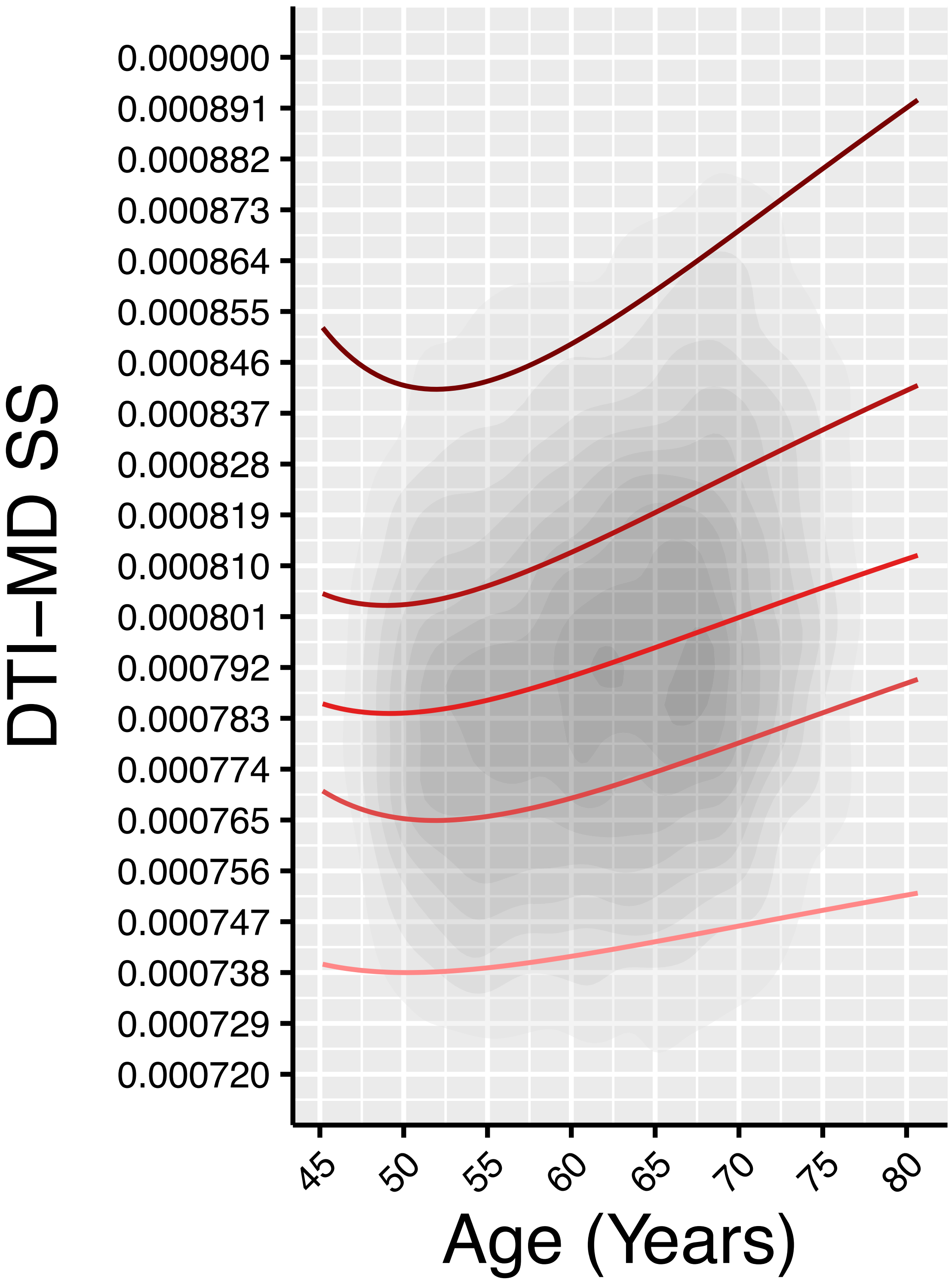

Figure S313. Full size normative centile reference curves calculated for the *sagittal stratum* tract for DTI-MD in females. Solid colored lines, ordered from lightest to darkest, indicate the following centiles: 5th, 25th, 50th, 75th, 95th. Gray overlay reflects kernel density (darker=greater degree of data point overlap). SS = *sagittal stratum.*

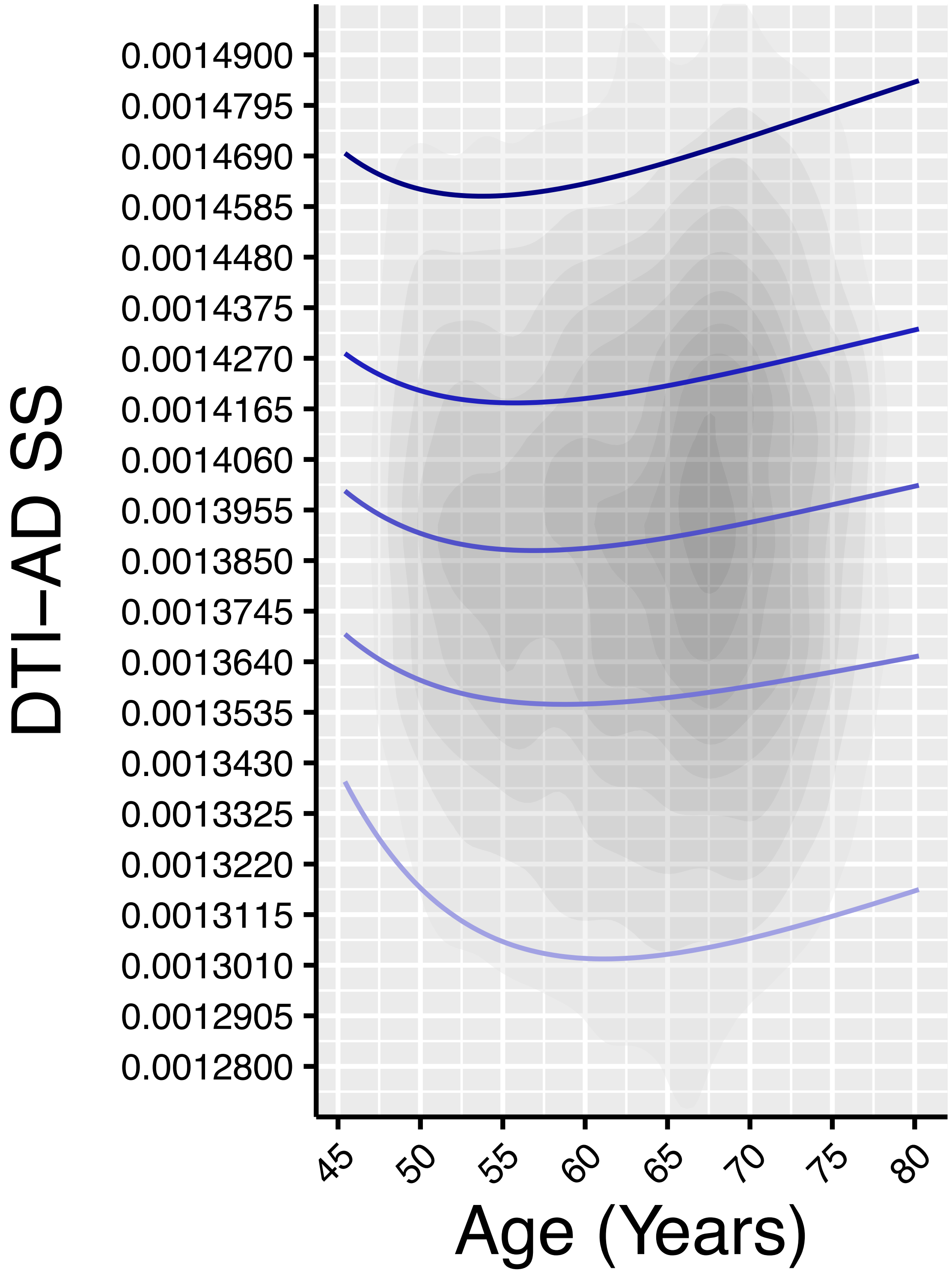

Figure S314. Full size normative centile reference curves calculated for the *sagittal stratum* tract for DTI-AD in males. Solid colored lines, ordered from lightest to darkest, indicate the following centiles: 5th, 25th, 50th, 75th, 95th. Gray overlay reflects kernel density (darker=greater degree of data point overlap). SS = *sagittal stratum.*

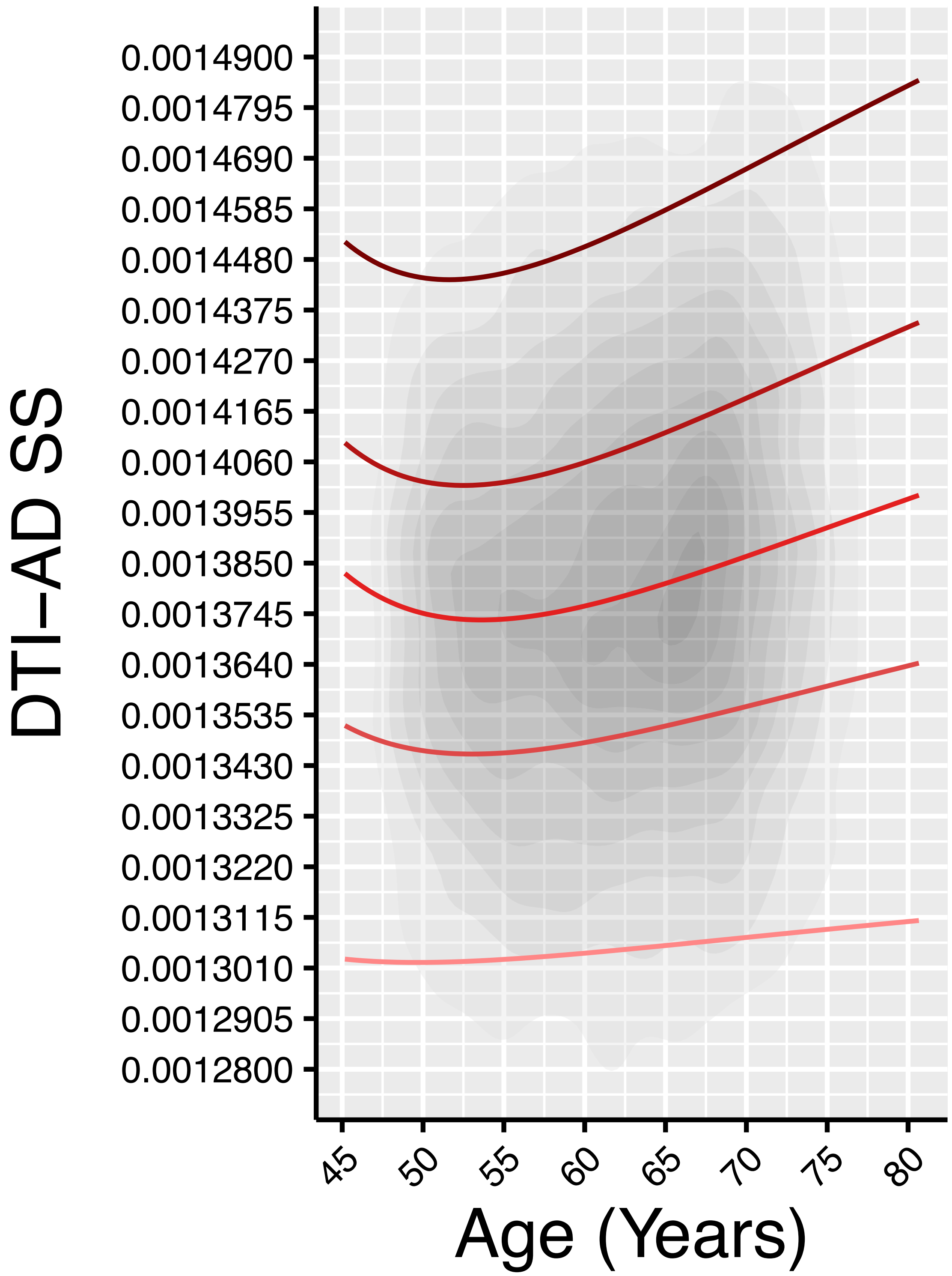

Figure S315. Full size normative centile reference curves calculated for the *sagittal stratum* tract for DTI-AD in females. Solid colored lines, ordered from lightest to darkest, indicate the following centiles: 5th, 25th, 50th, 75th, 95th. Gray overlay reflects kernel density (darker=greater degree of data point overlap). SS = *sagittal stratum.*

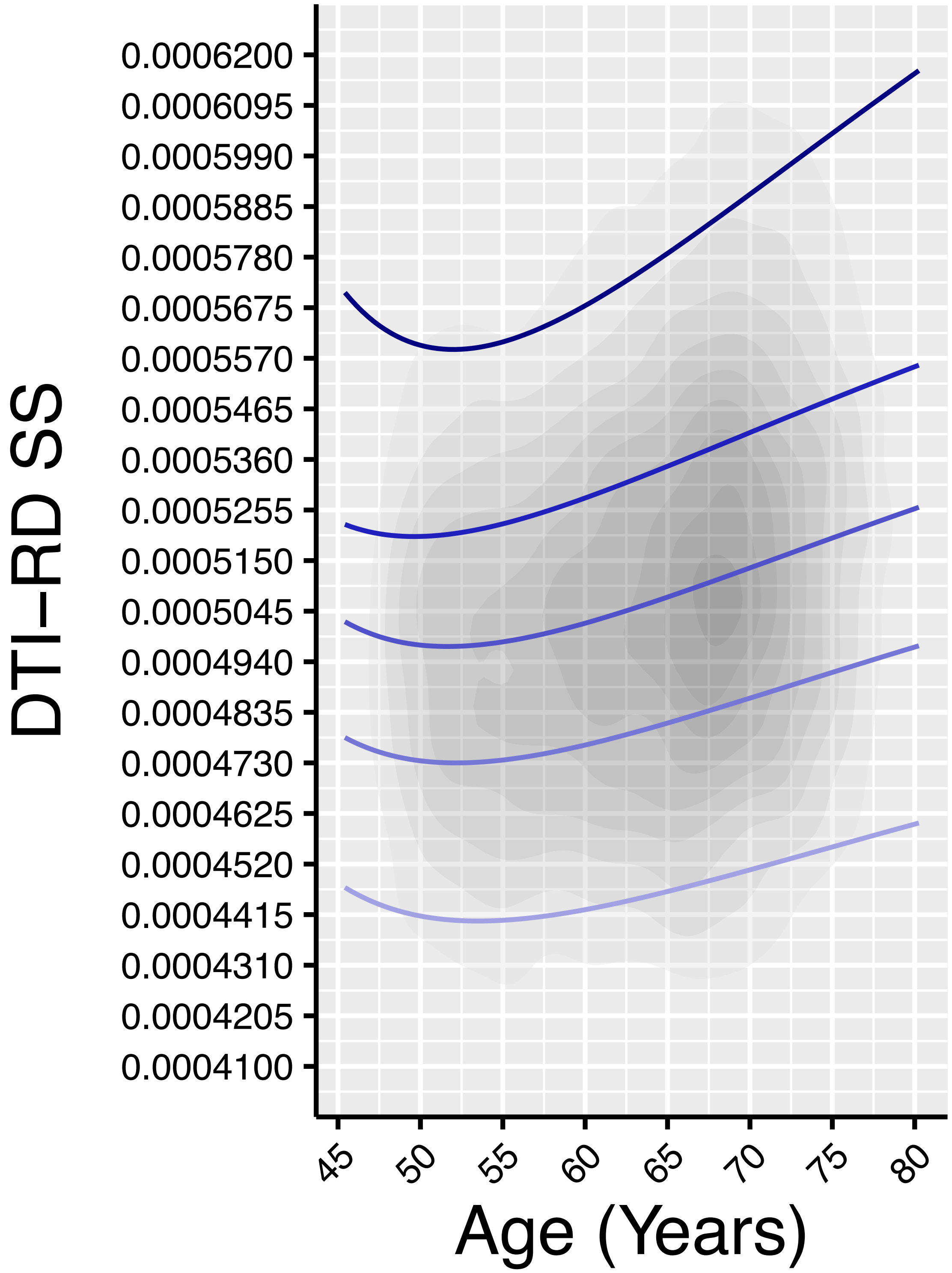

Figure S316. Full size normative centile reference curves calculated for the *sagittal stratum* tract for DTI-RD in males. Solid colored lines, ordered from lightest to darkest, indicate the following centiles: 5th, 25th, 50th, 75th, 95th. Gray overlay reflects kernel density (darker=greater degree of data point overlap). SS = *sagittal stratum.*

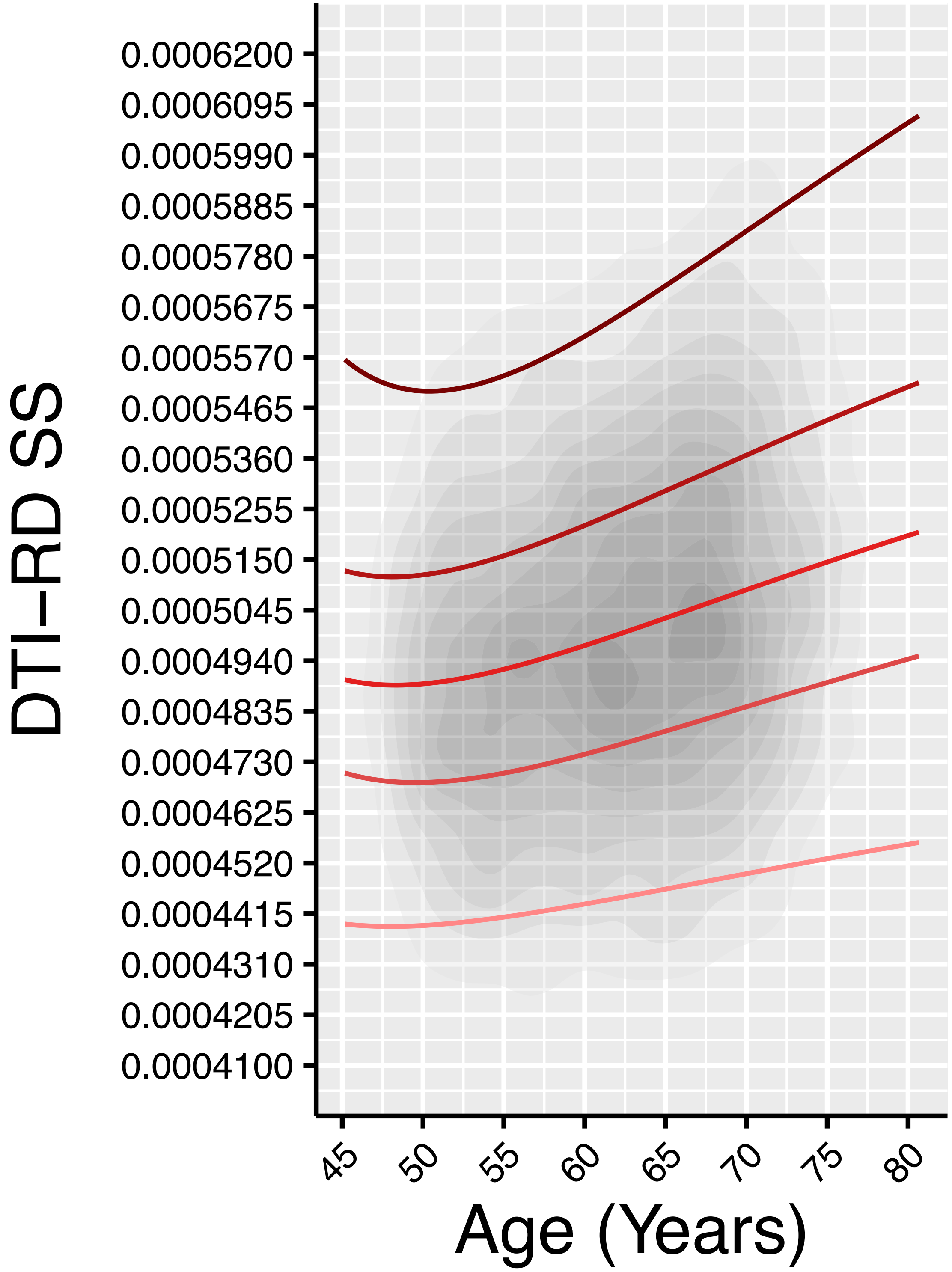

Figure S317. Full size normative centile reference curves calculated for the *sagittal stratum* tract for DTI-RD in females. Solid colored lines, ordered from lightest to darkest, indicate the following centiles: 5th, 25th, 50th, 75th, 95th. Gray overlay reflects kernel density (darker=greater degree of data point overlap). SS = *sagittal stratum.*

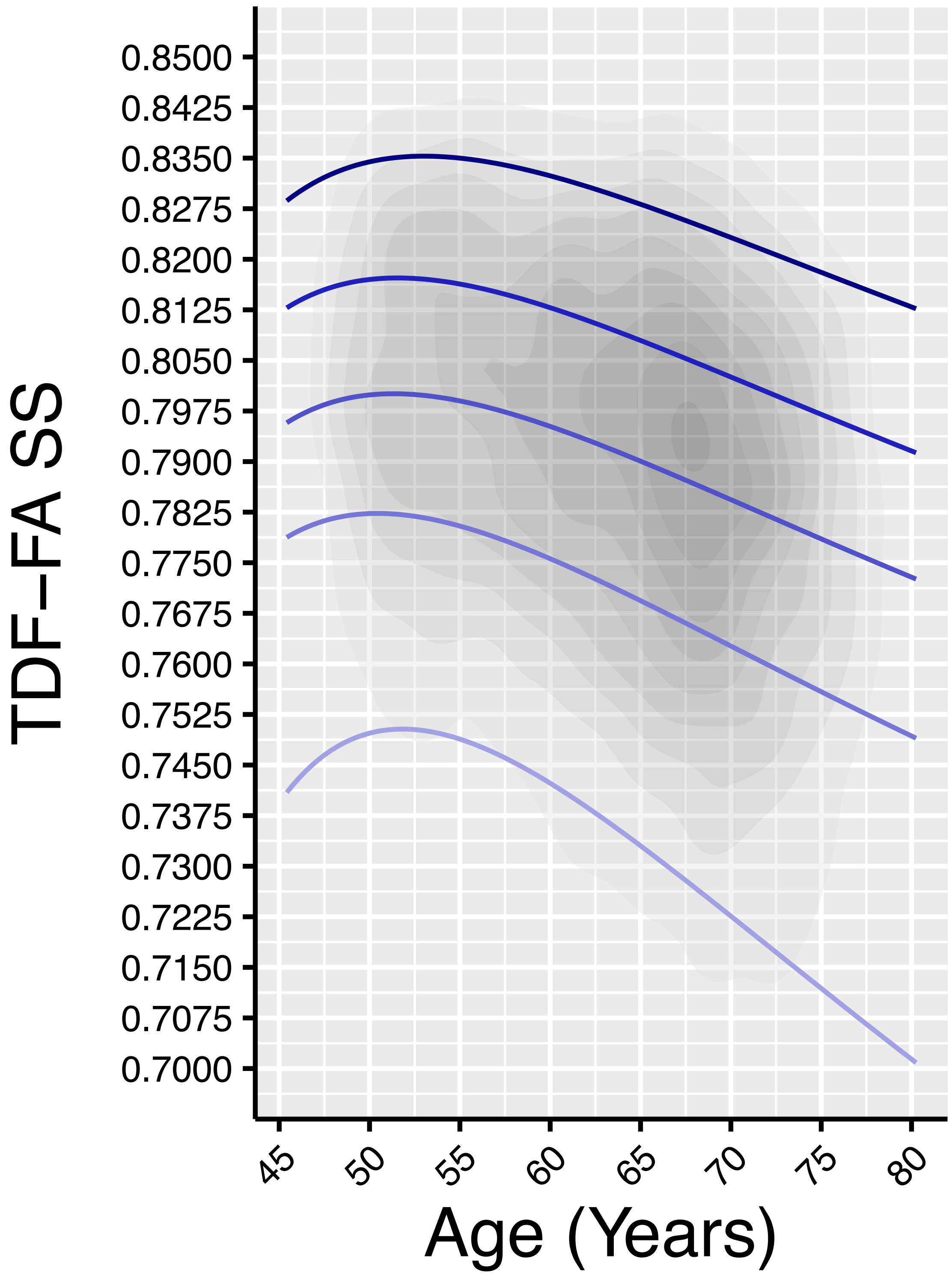

Figure S318. Full size normative centile reference curves calculated for the *sagittal stratum* tract for TDF-FA in males. Solid colored lines, ordered from lightest to darkest, indicate the following centiles: 5th, 25th, 50th, 75th, 95th. Gray overlay reflects kernel density (darker=greater degree of data point overlap). SS = *sagittal stratum.*

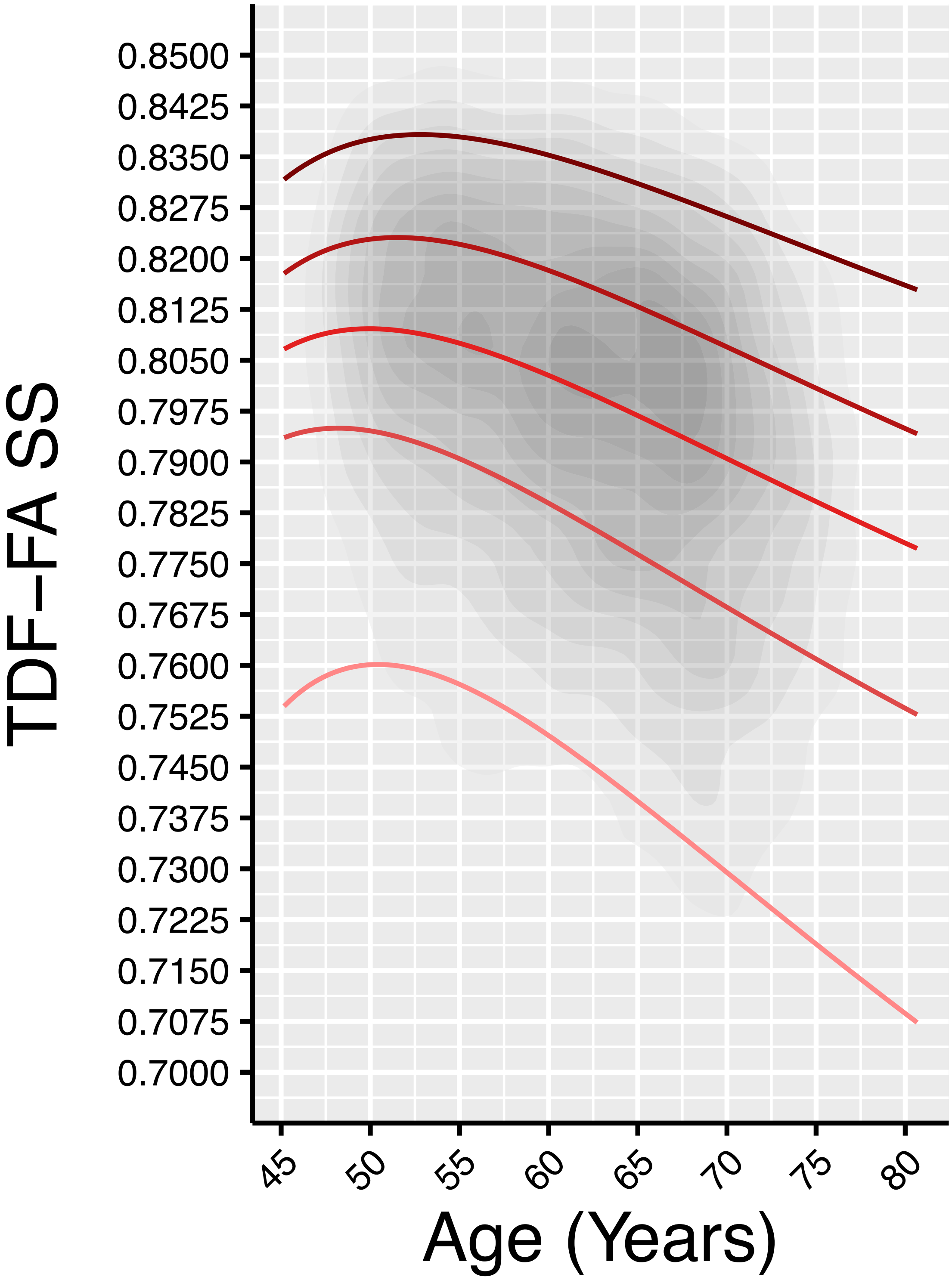

Figure S319. Full size normative centile reference curves calculated for the *sagittal stratum* tract for TDF-FA in females. Solid colored lines, ordered from lightest to darkest, indicate the following centiles: 5th, 25th, 50th, 75th, 95th. Gray overlay reflects kernel density (darker=greater degree of data point overlap). SS = *sagittal stratum.*

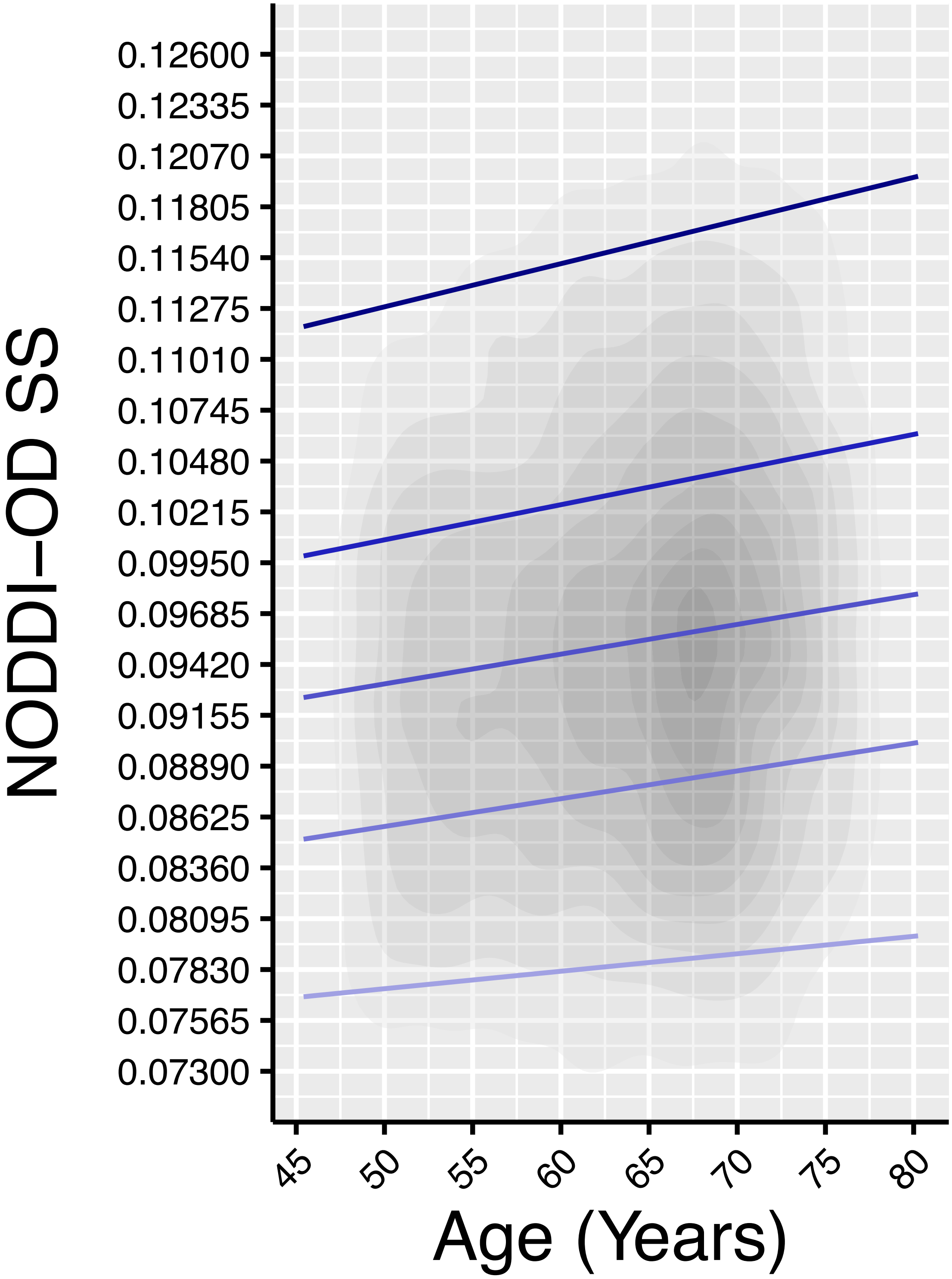

Figure S320. Full size normative centile reference curves calculated for the *sagittal stratum* tract for NODDI-OD in males. Solid colored lines, ordered from lightest to darkest, indicate the following centiles: 5th, 25th, 50th, 75th, 95th. Gray overlay reflects kernel density (darker=greater degree of data point overlap). SS = *sagittal stratum.*

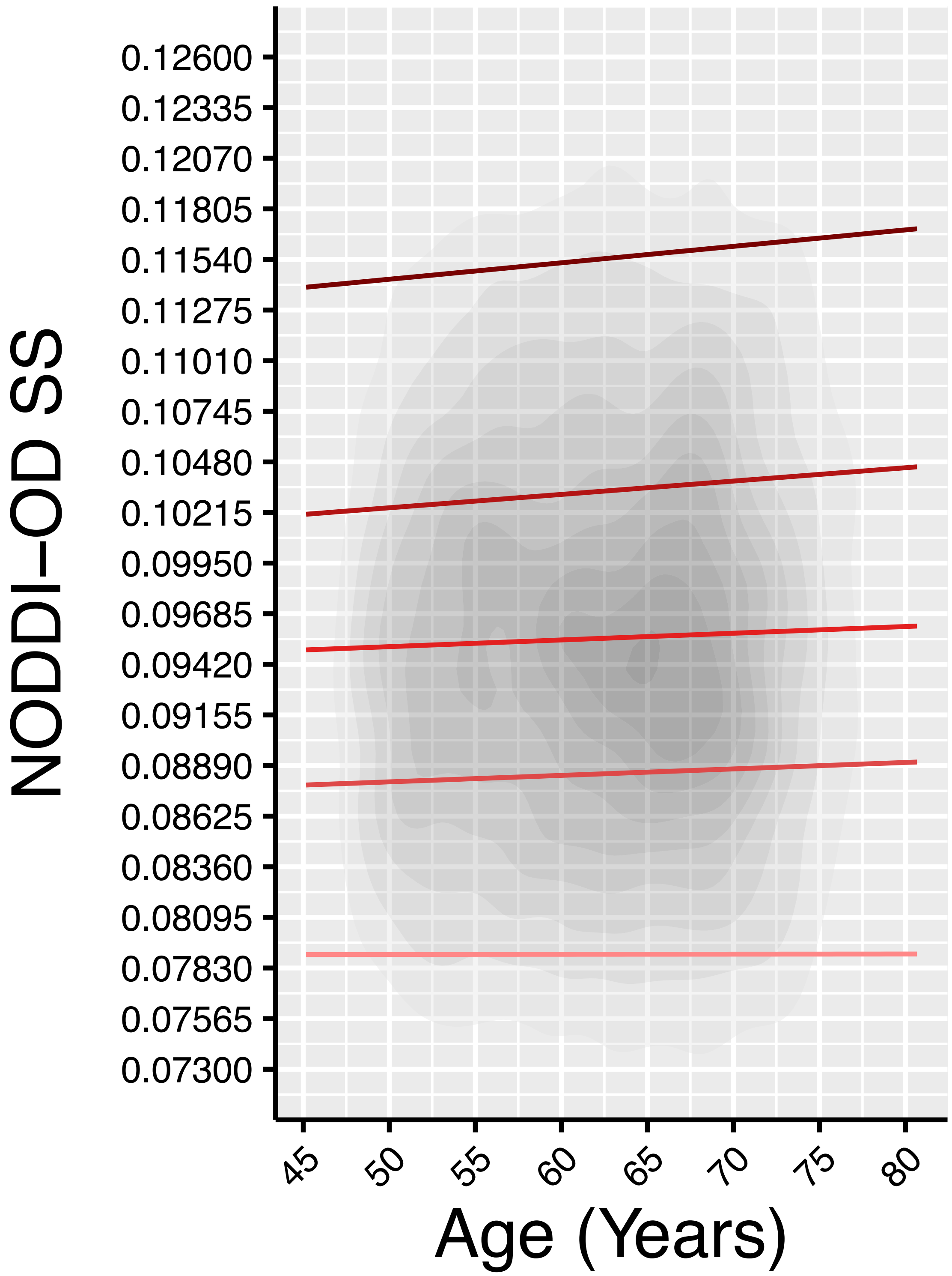

Figure S321. Full size normative centile reference curves calculated for the *sagittal stratum* tract for NODDI-OD in females. Solid colored lines, ordered from lightest to darkest, indicate the following centiles: 5th, 25th, 50th, 75th, 95th. Gray overlay reflects kernel density (darker=greater degree of data point overlap). SS = *sagittal stratum.*

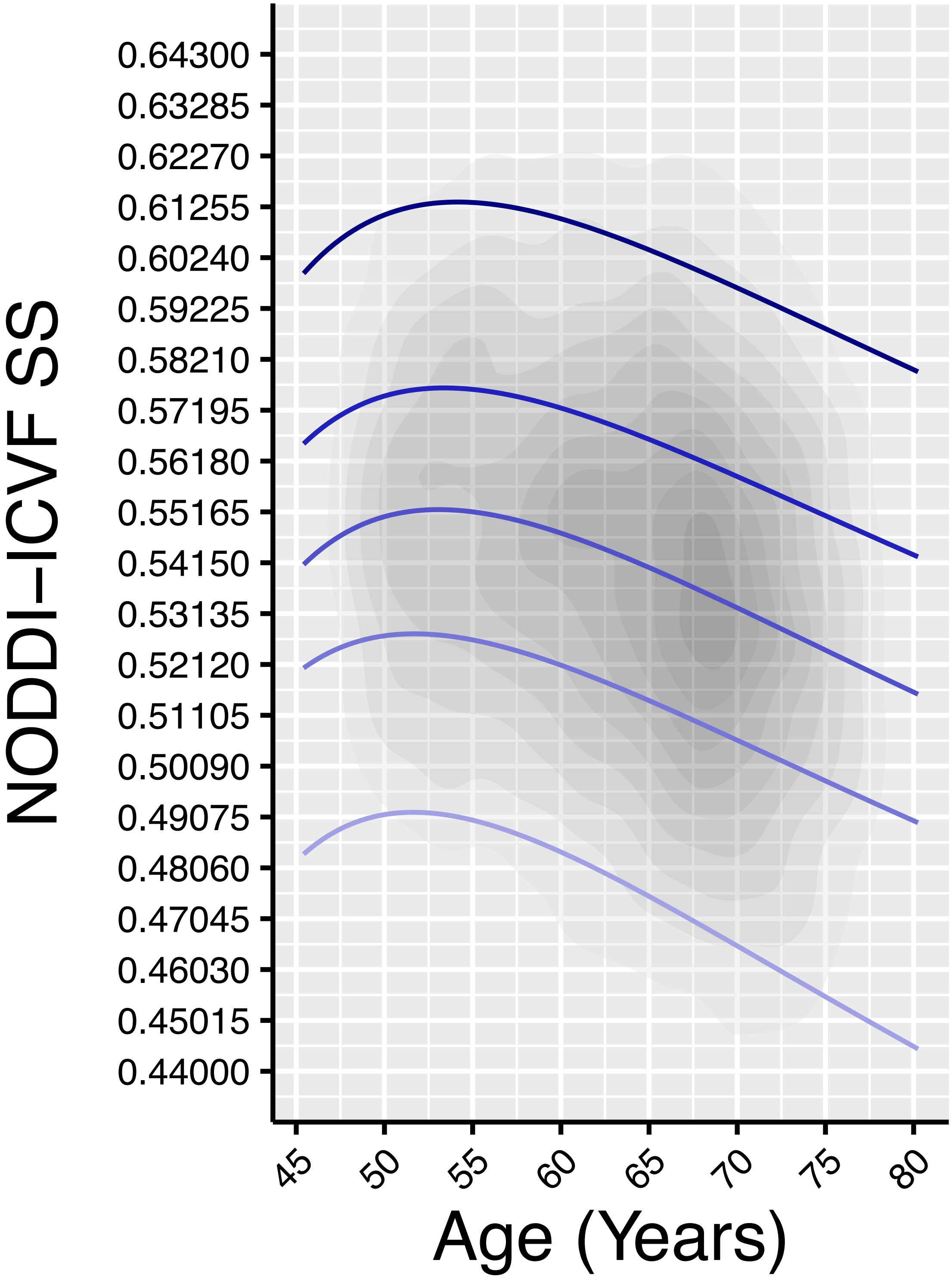

Figure S322. Full size normative centile reference curves calculated for the *sagittal stratum* tract for NODDI-ICVF in males. Solid colored lines, ordered from lightest to darkest, indicate the following centiles: 5th, 25th, 50th, 75th, 95th. Gray overlay reflects kernel density (darker=greater degree of data point overlap). SS = *sagittal stratum.*

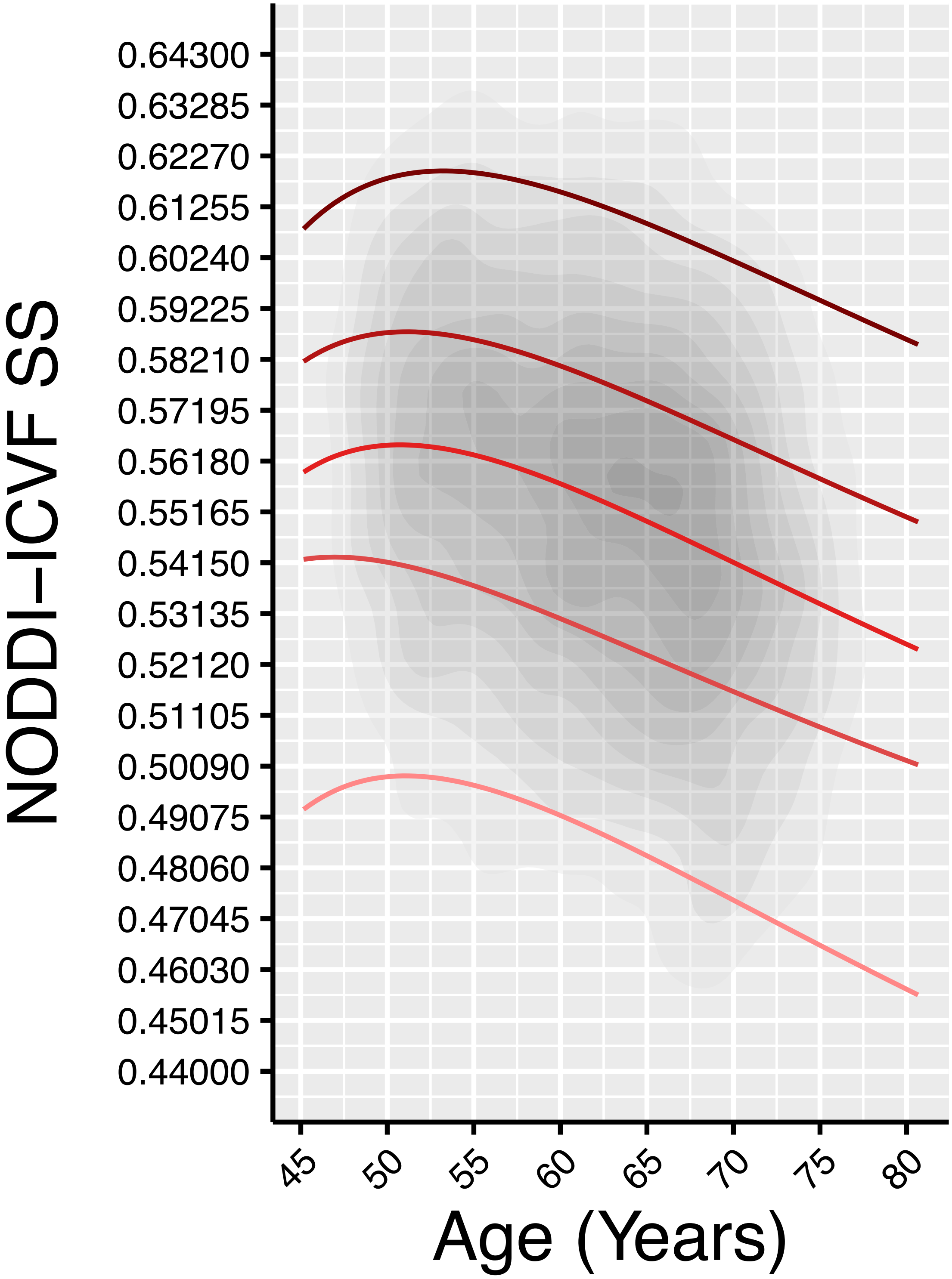

Figure S323. Full size normative centile reference curves calculated for the *sagittal stratum* tract for NODDI-ICVF in females. Solid colored lines, ordered from lightest to darkest, indicate the following centiles: 5th, 25th, 50th, 75th, 95th. Gray overlay reflects kernel density (darker=greater degree of data point overlap). SS = *sagittal stratum.*

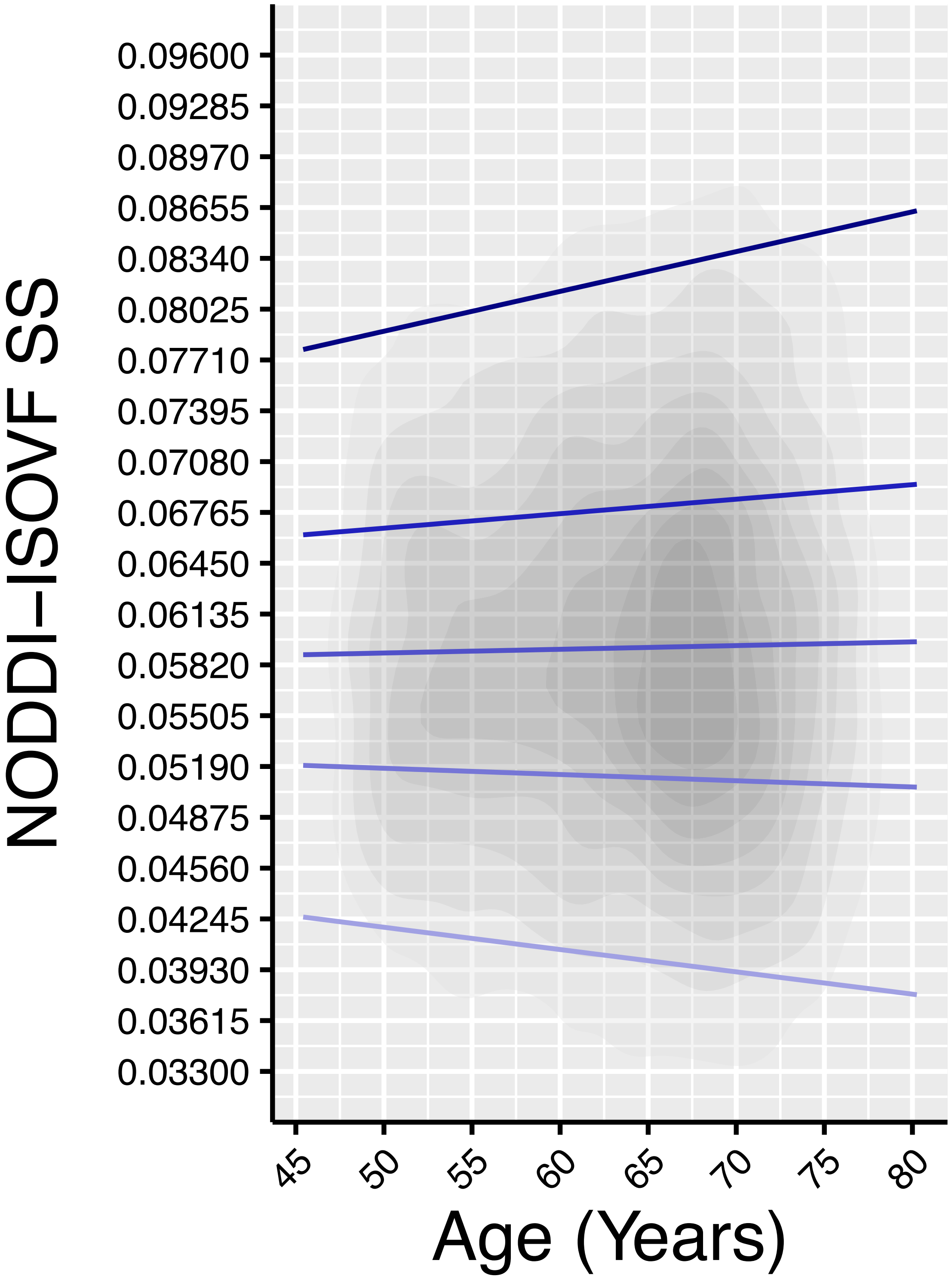

Figure S324. Full size normative centile reference curves calculated for the *sagittal stratum* tract for NODDI-ISOVF in males. Solid colored lines, ordered from lightest to darkest, indicate the following centiles: 5th, 25th, 50th, 75th, 95th. Gray overlay reflects kernel density (darker=greater degree of data point overlap). SS = *sagittal stratum.*

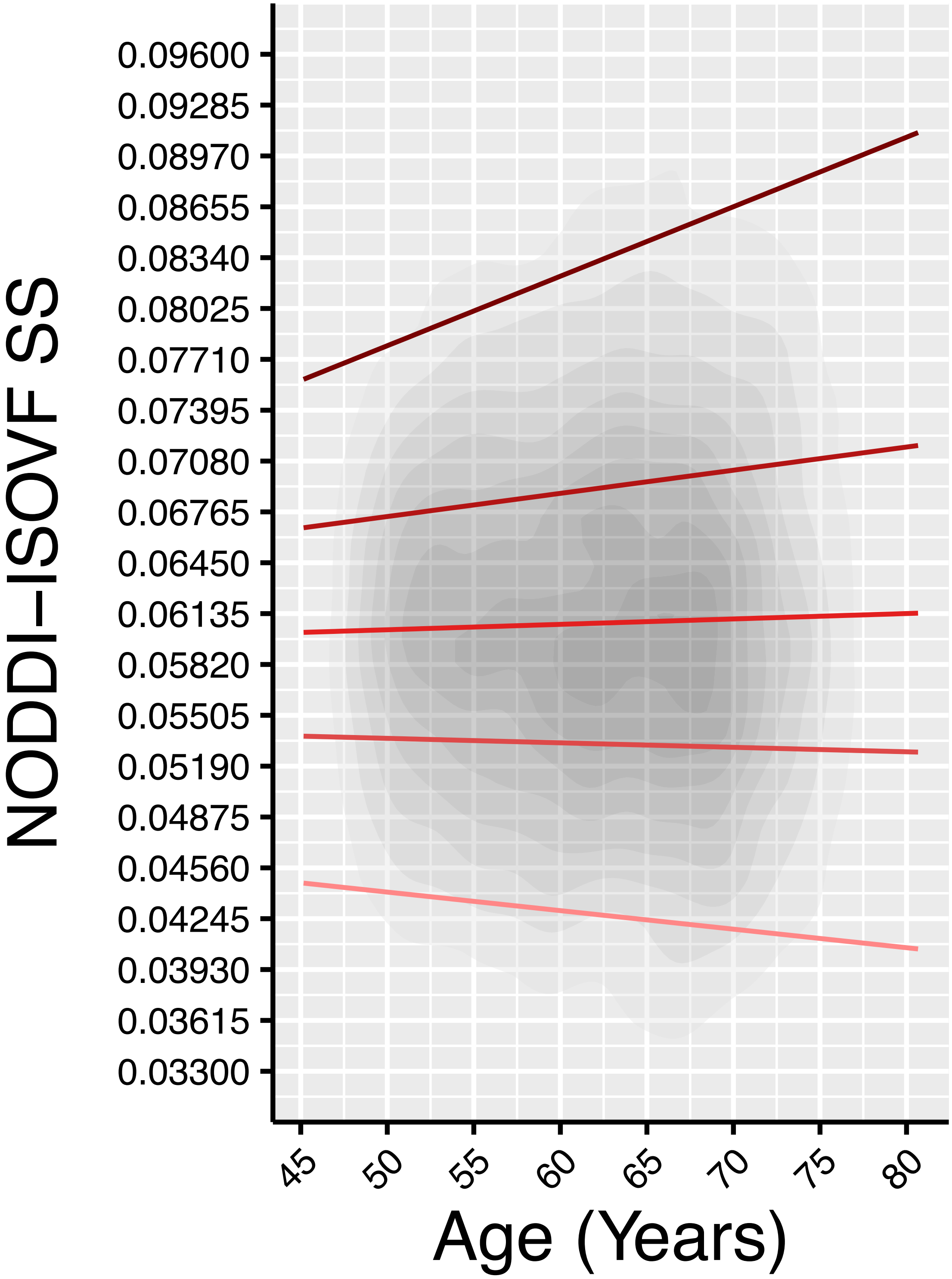

Figure S325. Full size normative centile reference curves calculated for the *sagittal stratum* tract for NODDI-ISOVF in females. Solid colored lines, ordered from lightest to darkest, indicate the following centiles: 5th, 25th, 50th, 75th, 95th. Gray overlay reflects kernel density (darker=greater degree of data point overlap). SS = *sagittal stratum.*

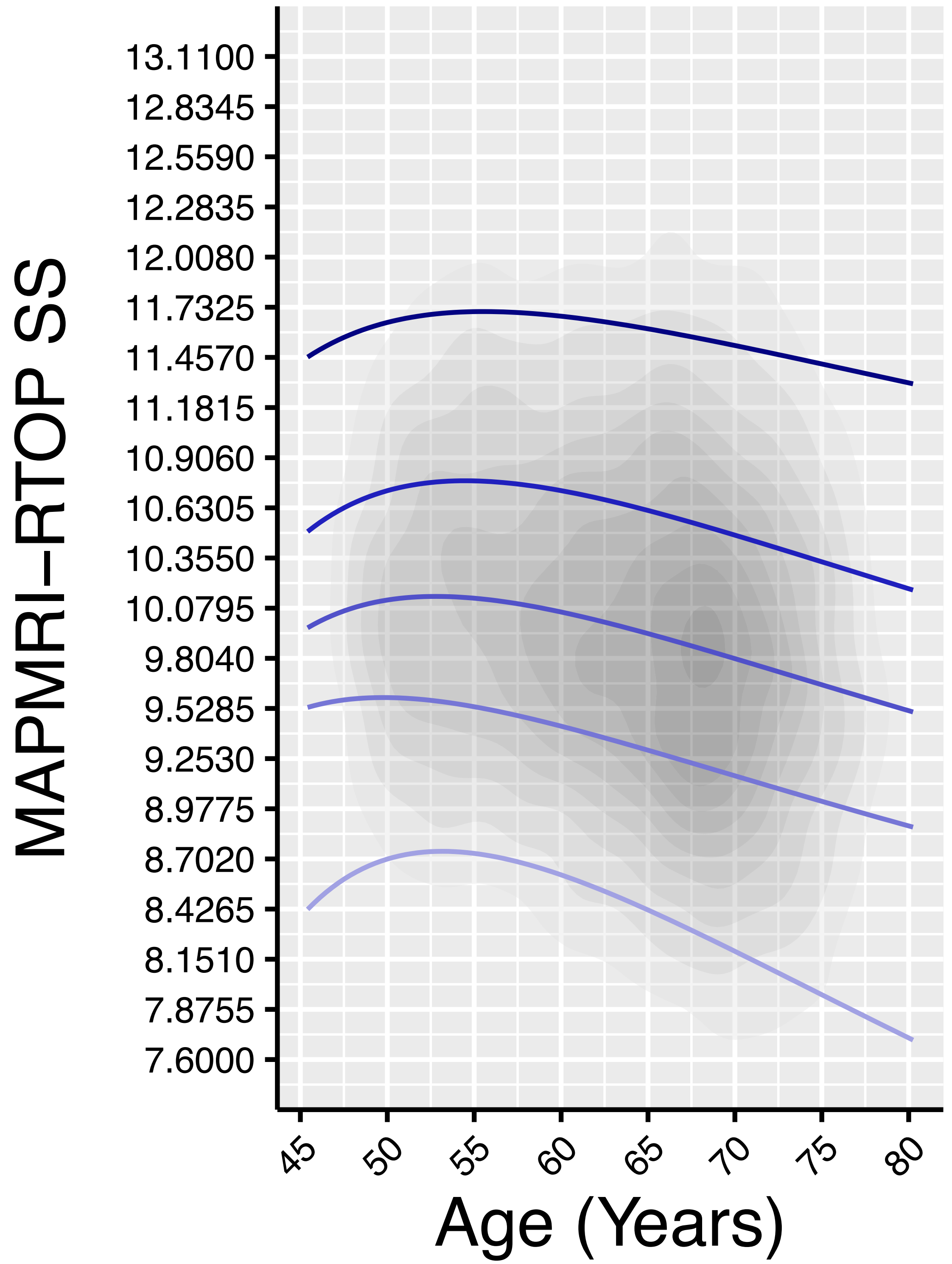

Figure S326. Full size normative centile reference curves calculated for the *sagittal stratum* tract for MAPMRI-RTOP in males. Solid colored lines, ordered from lightest to darkest, indicate the following centiles: 5th, 25th, 50th, 75th, 95th. Gray overlay reflects kernel density (darker=greater degree of data point overlap). SS = *sagittal stratum.*

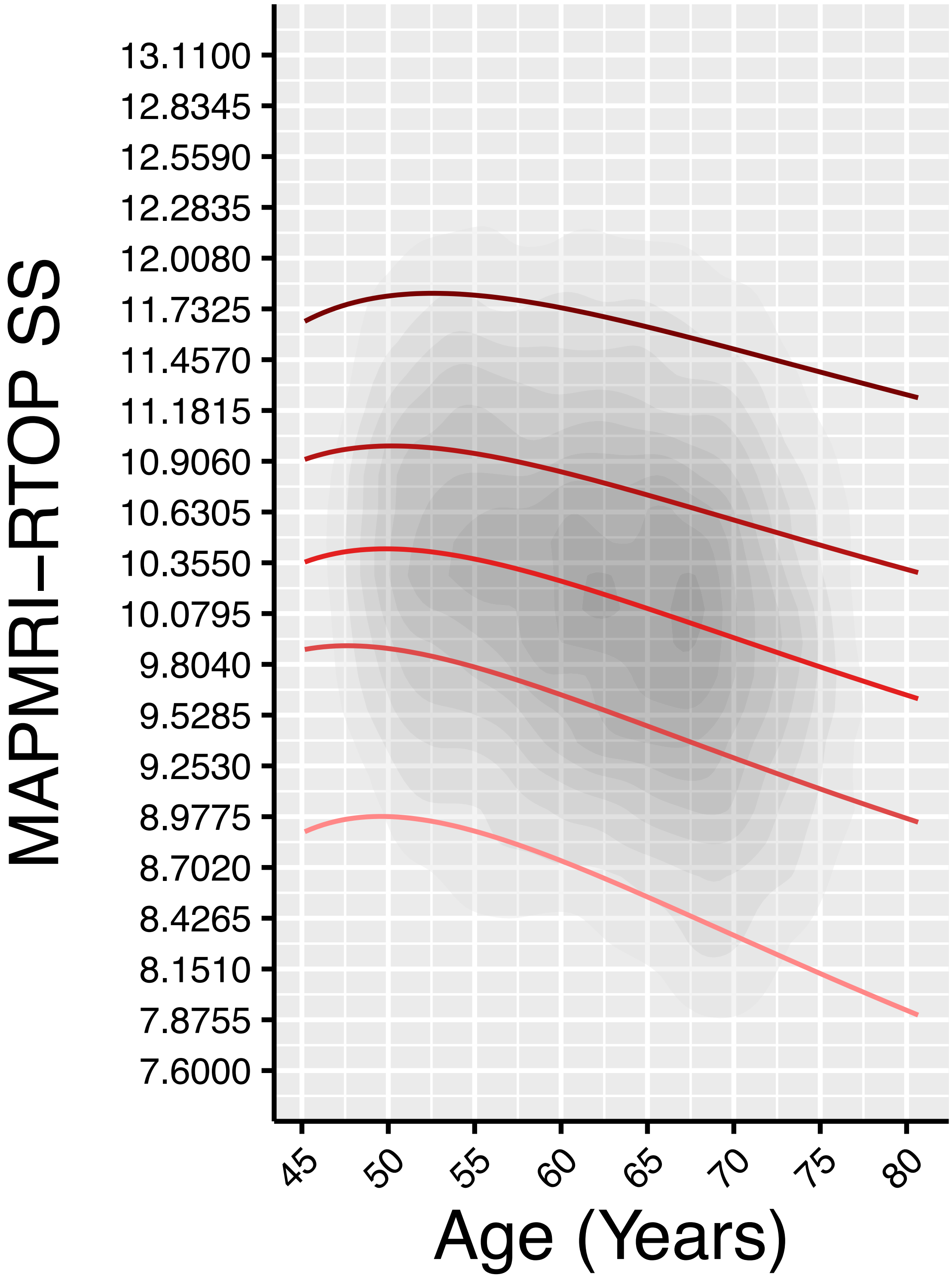

Figure S327. Full size normative centile reference curves calculated for the *sagittal stratum* tract for MAPMRI-RTOP in females. Solid colored lines, ordered from lightest to darkest, indicate the following centiles: 5th, 25th, 50th, 75th, 95th. Gray overlay reflects kernel density (darker=greater degree of data point overlap). SS = *sagittal stratum.*

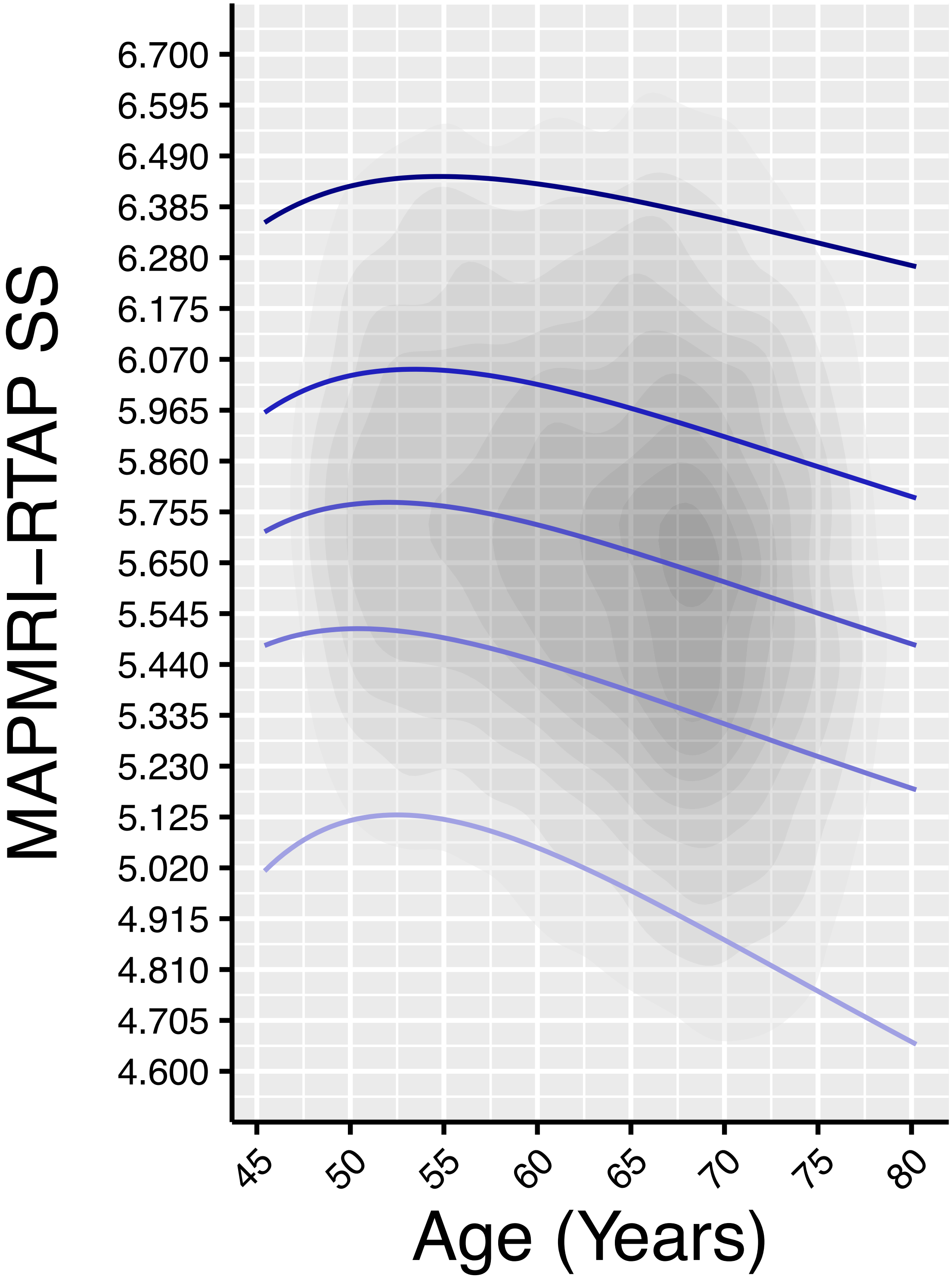

Figure S328. Full size normative centile reference curves calculated for the *sagittal stratum* tract for MAPMRI-RTAP in males. Solid colored lines, ordered from lightest to darkest, indicate the following centiles: 5th, 25th, 50th, 75th, 95th. Gray overlay reflects kernel density (darker=greater degree of data point overlap). SS = *sagittal stratum.*

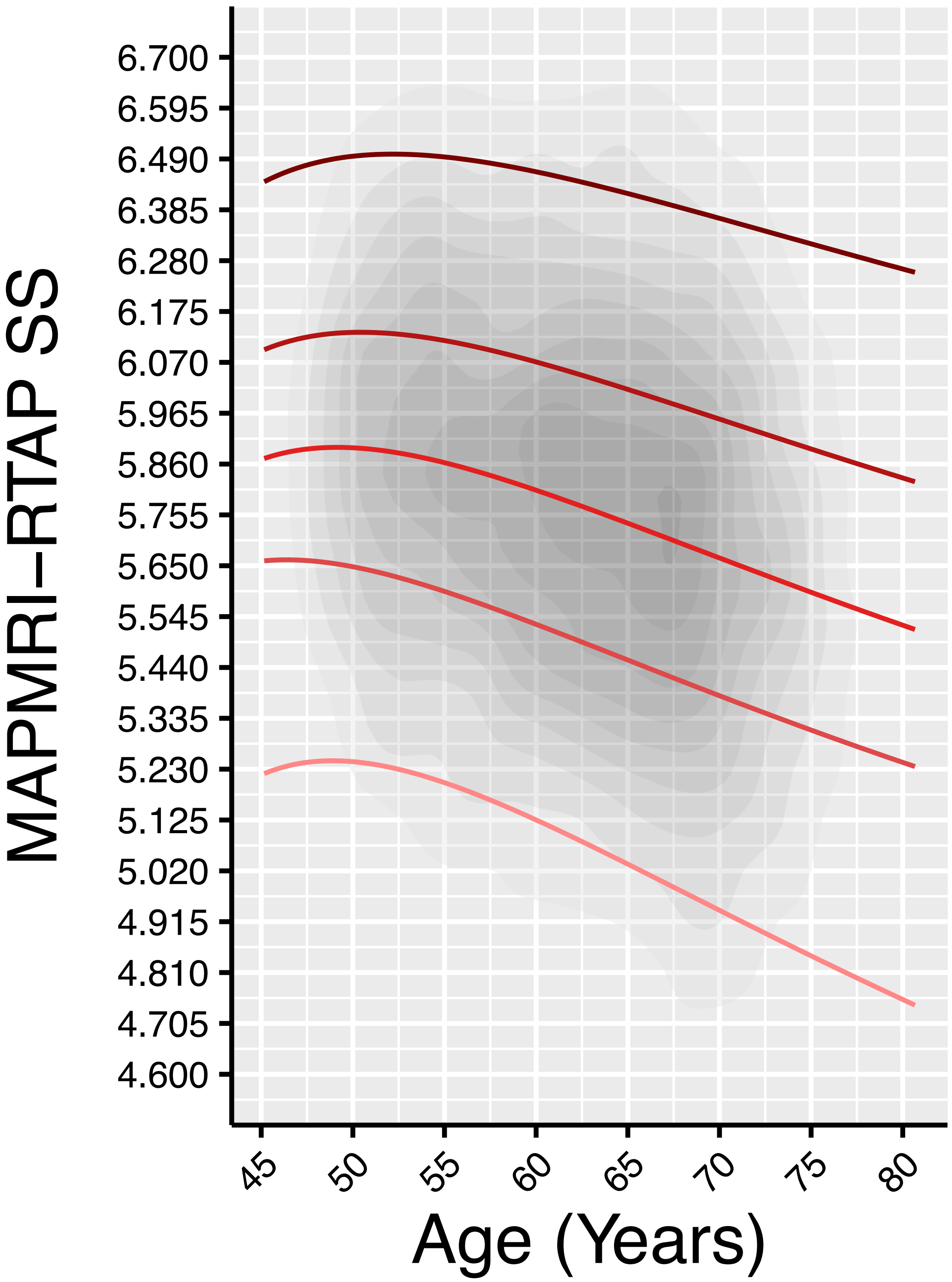

Figure S329. Full size normative centile reference curves calculated for the *sagittal stratum* tract for MAPMRI-RTAP in females. Solid colored lines, ordered from lightest to darkest, indicate the following centiles: 5th, 25th, 50th, 75th, 95th. Gray overlay reflects kernel density (darker=greater degree of data point overlap). SS = *sagittal stratum.*

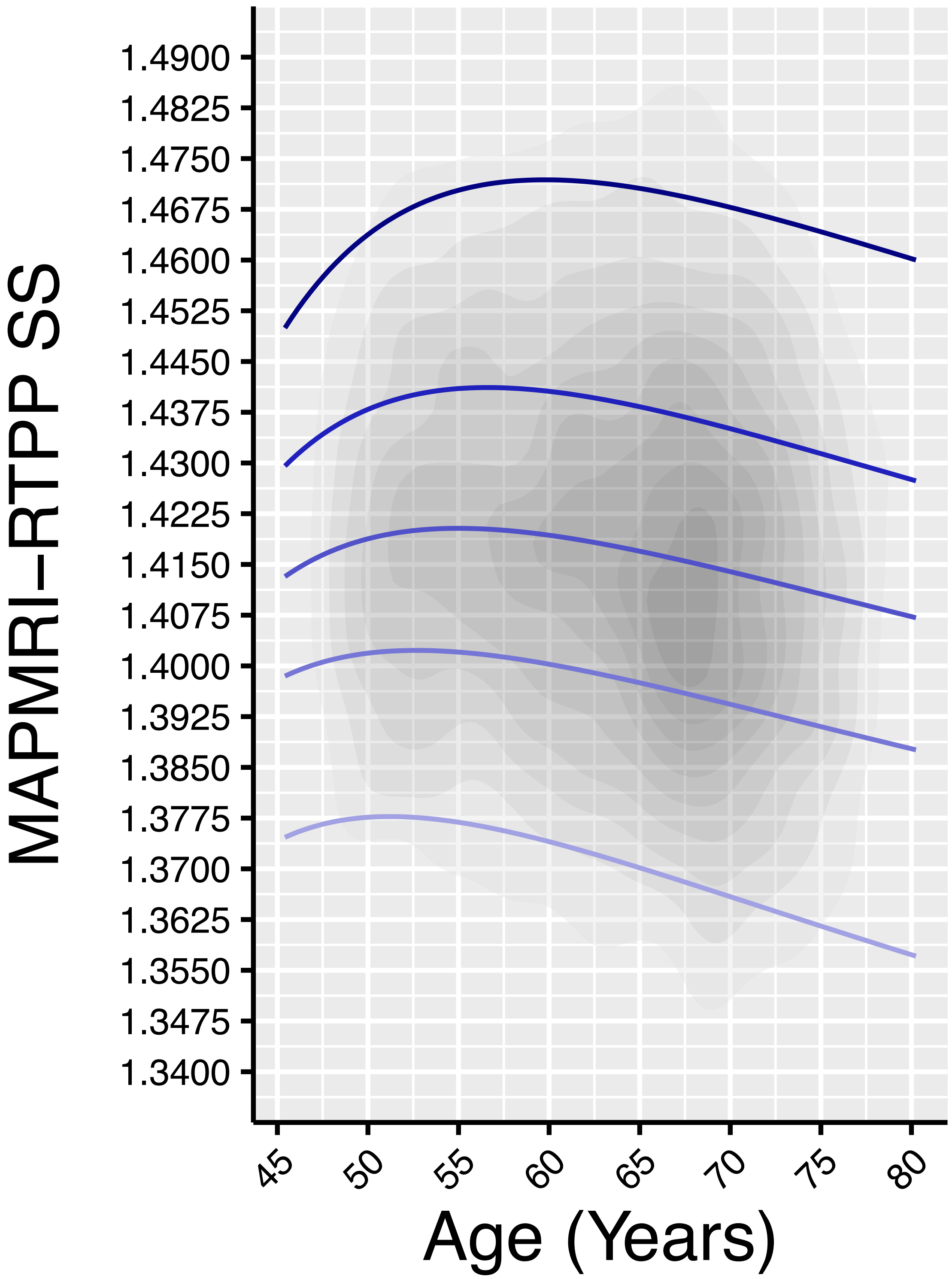

Figure S330. Full size normative centile reference curves calculated for the *sagittal stratum* tract for MAPMRI-RTPP in males. Solid colored lines, ordered from lightest to darkest, indicate the following centiles: 5th, 25th, 50th, 75th, 95th. Gray overlay reflects kernel density (darker=greater degree of data point overlap). SS = *sagittal stratum.*

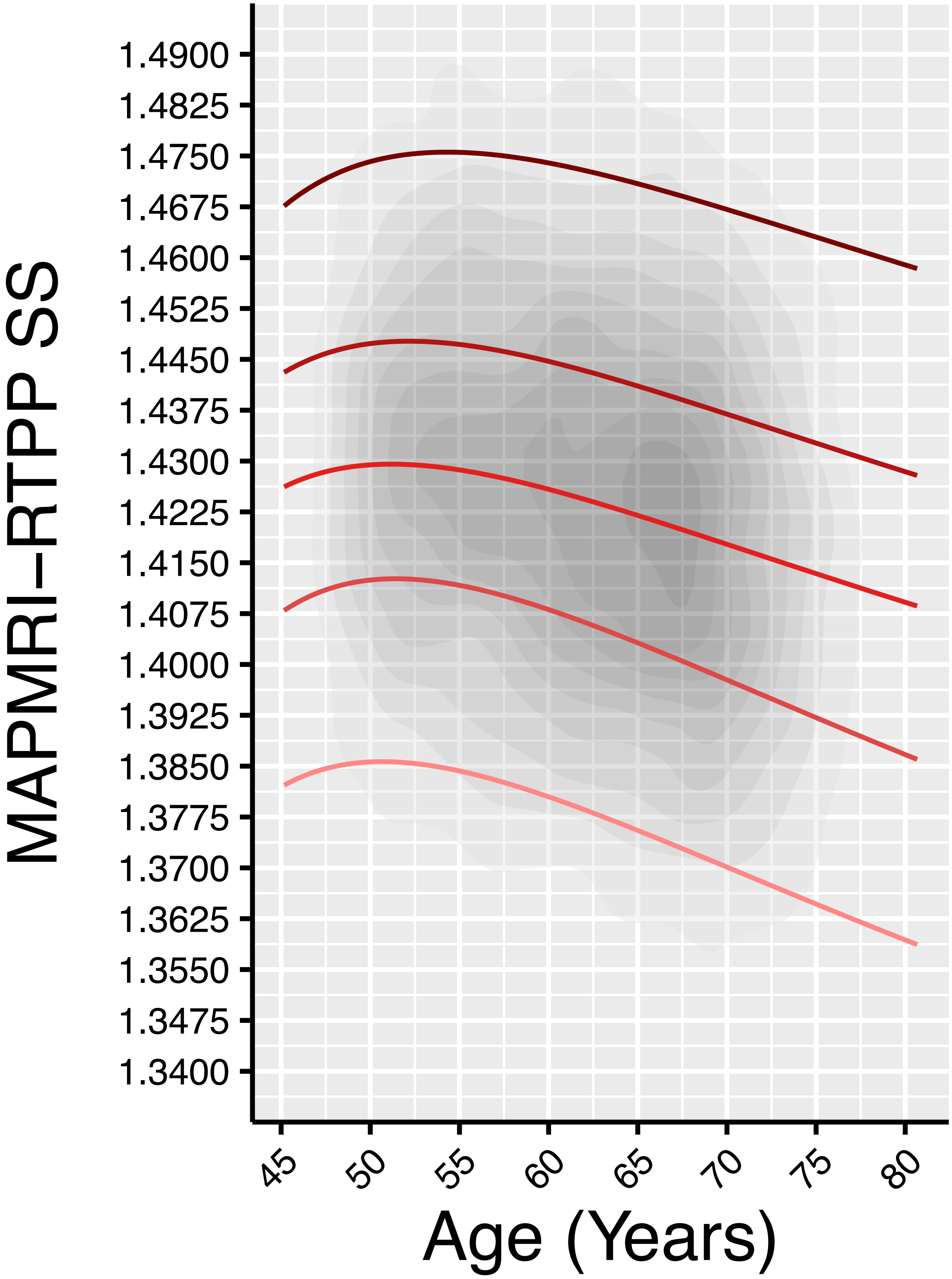

Figure S331. Full size normative centile reference curves calculated for the *sagittal stratum* tract for MAPMRI-RTPP in females. Solid colored lines, ordered from lightest to darkest, indicate the following centiles: 5th, 25th, 50th, 75th, 95th. Gray overlay reflects kernel density (darker=greater degree of data point overlap). SS = *sagittal stratum.*

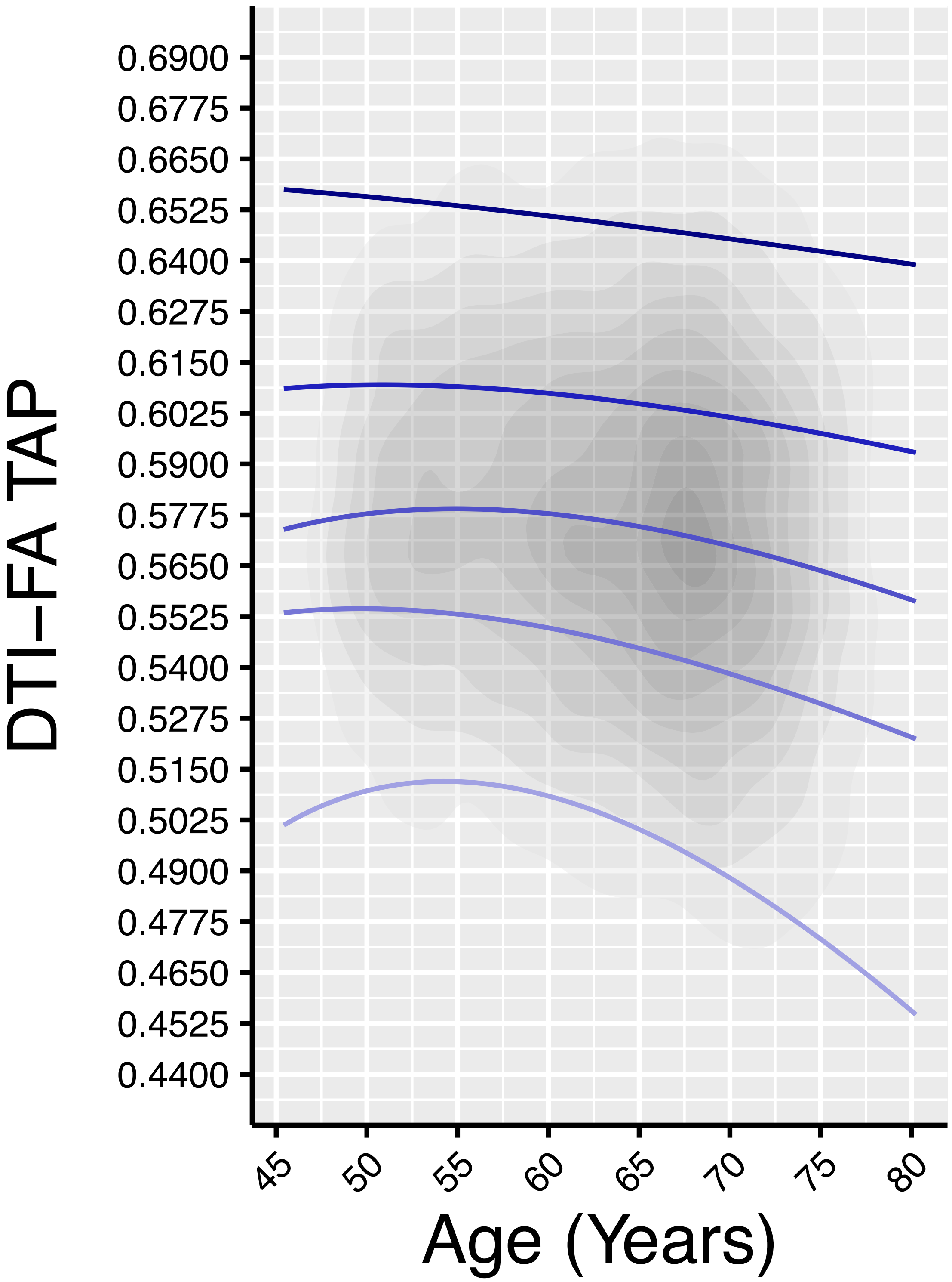

Figure S332. Full size normative centile reference curves calculated for the tapetum tract for DTI-FA in males. Solid colored lines, ordered from lightest to darkest, indicate the following centiles: 5th, 25th, 50th, 75th, 95th. Gray overlay reflects kernel density (darker=greater degree of data point overlap). TAP = tapetum.

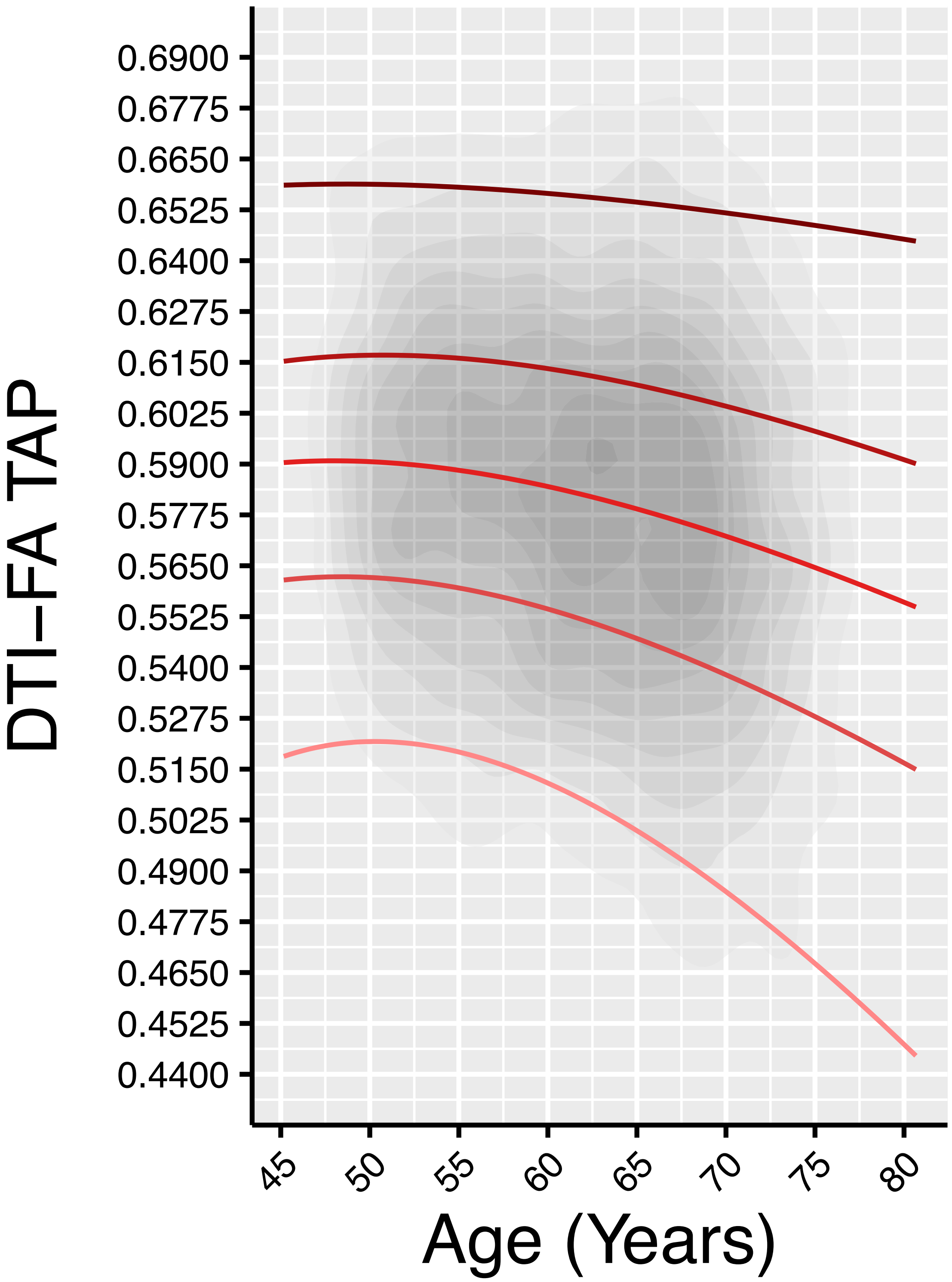

Figure S333. Full size normative centile reference curves calculated for the tapetum tract for DTI-FA in females. Solid colored lines, ordered from lightest to darkest, indicate the following centiles: 5th, 25th, 50th, 75th, 95th. Gray overlay reflects kernel density (darker=greater degree of data point overlap). TAP = tapetum.

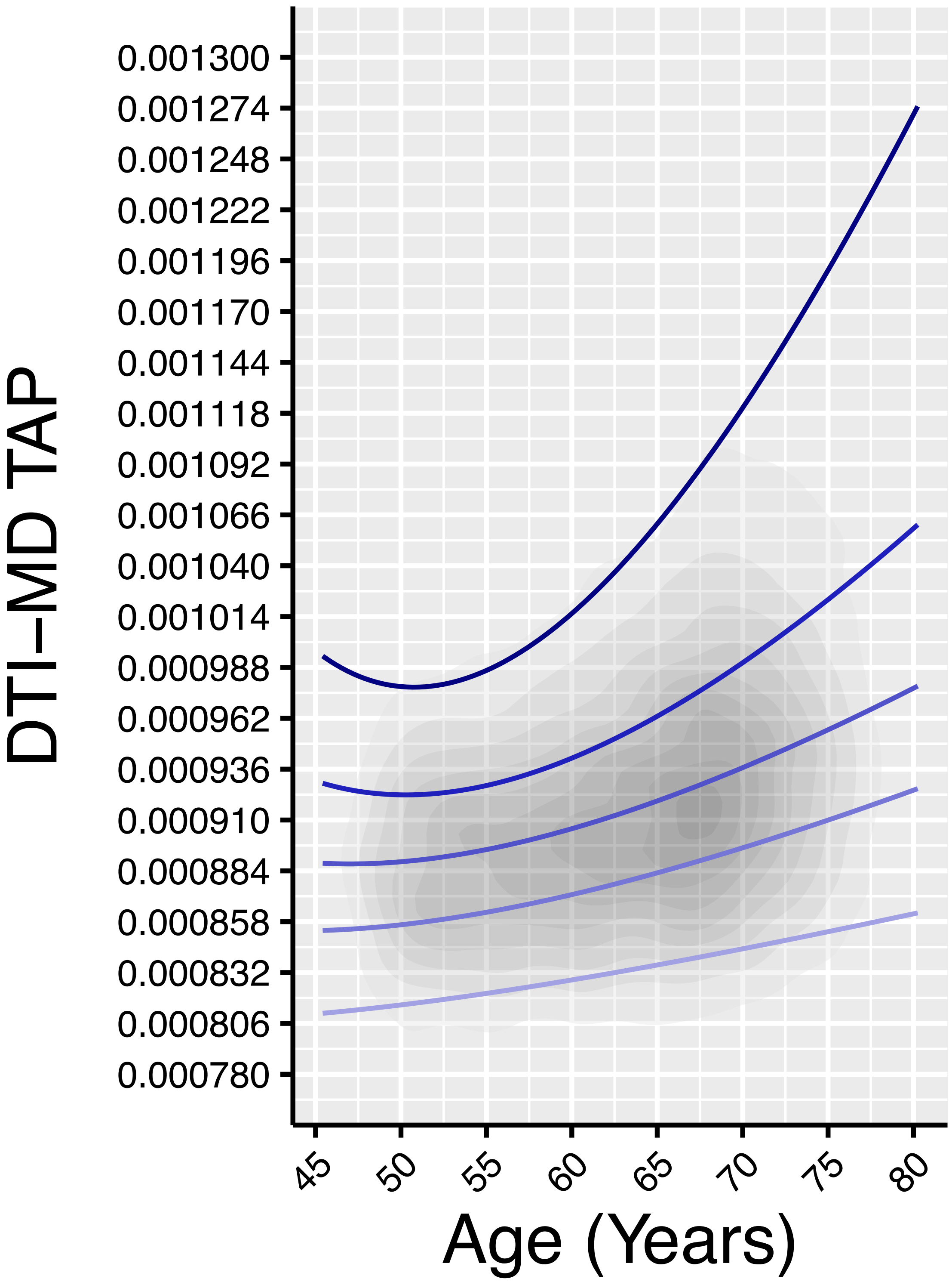

Figure S334. Full size normative centile reference curves calculated for the tapetum tract for DTI-MD in males. Solid colored lines, ordered from lightest to darkest, indicate the following centiles: 5th, 25th, 50th, 75th, 95th. Gray overlay reflects kernel density (darker=greater degree of data point overlap). TAP = tapetum.

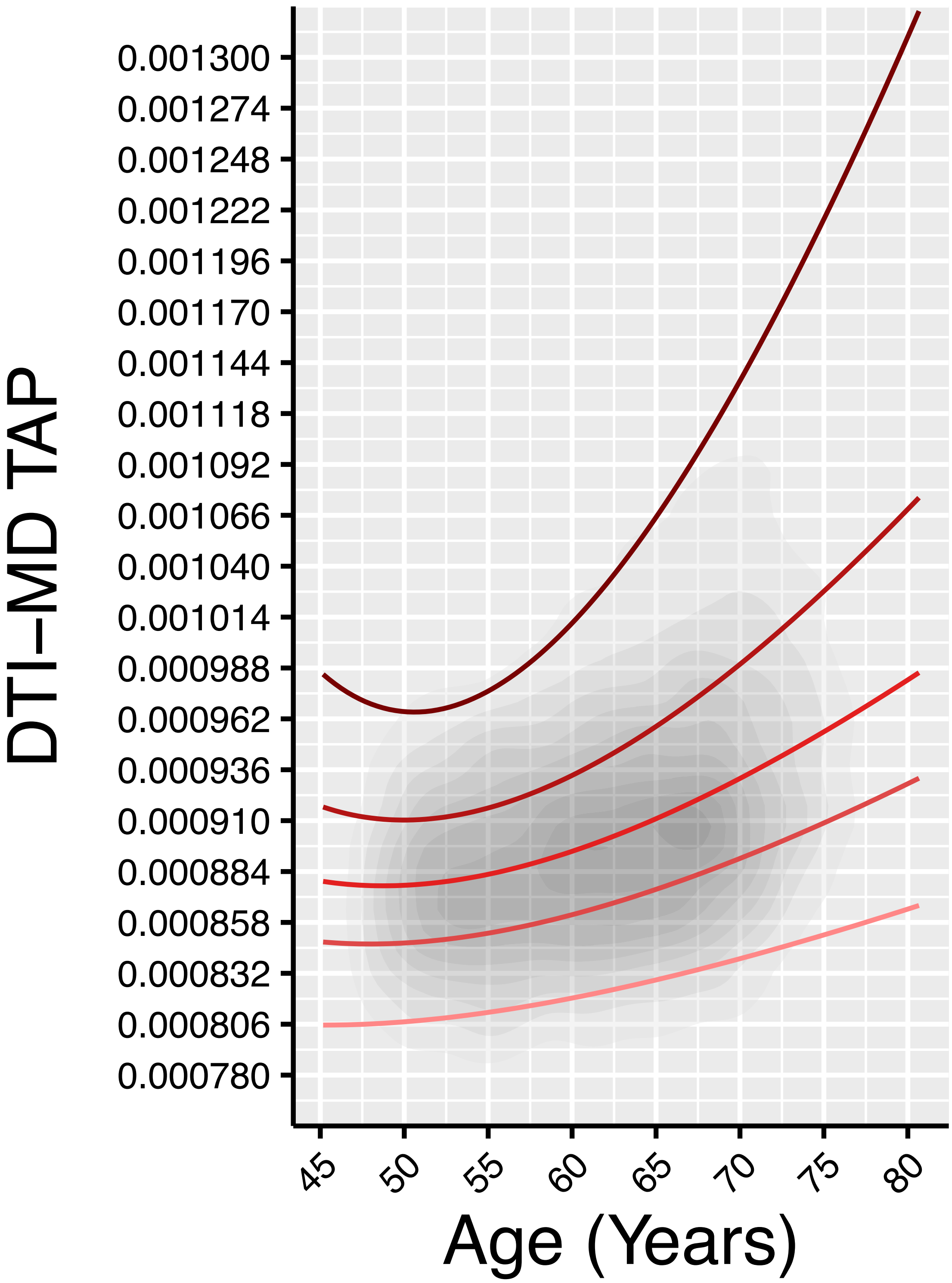

Figure S335. Full size normative centile reference curves calculated for the tapetum tract for DTI-MD in females. Solid colored lines, ordered from lightest to darkest, indicate the following centiles: 5th, 25th, 50th, 75th, 95th. Gray overlay reflects kernel density (darker=greater degree of data point overlap). TAP = tapetum.

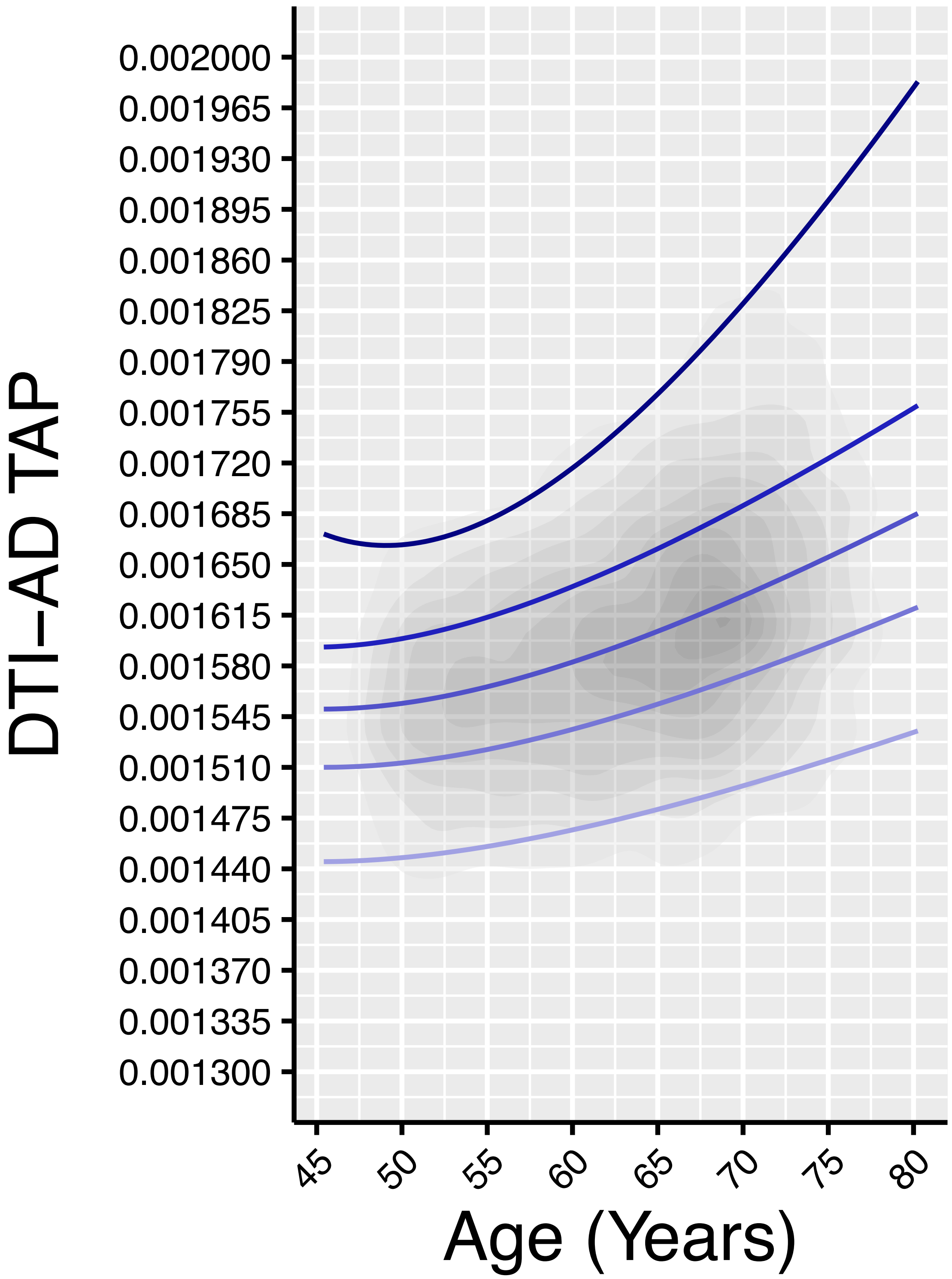

Figure S336. Full size normative centile reference curves calculated for the tapetum tract for DTI-AD in males. Solid colored lines, ordered from lightest to darkest, indicate the following centiles: 5th, 25th, 50th, 75th, 95th. Gray overlay reflects kernel density (darker=greater degree of data point overlap). TAP = tapetum.

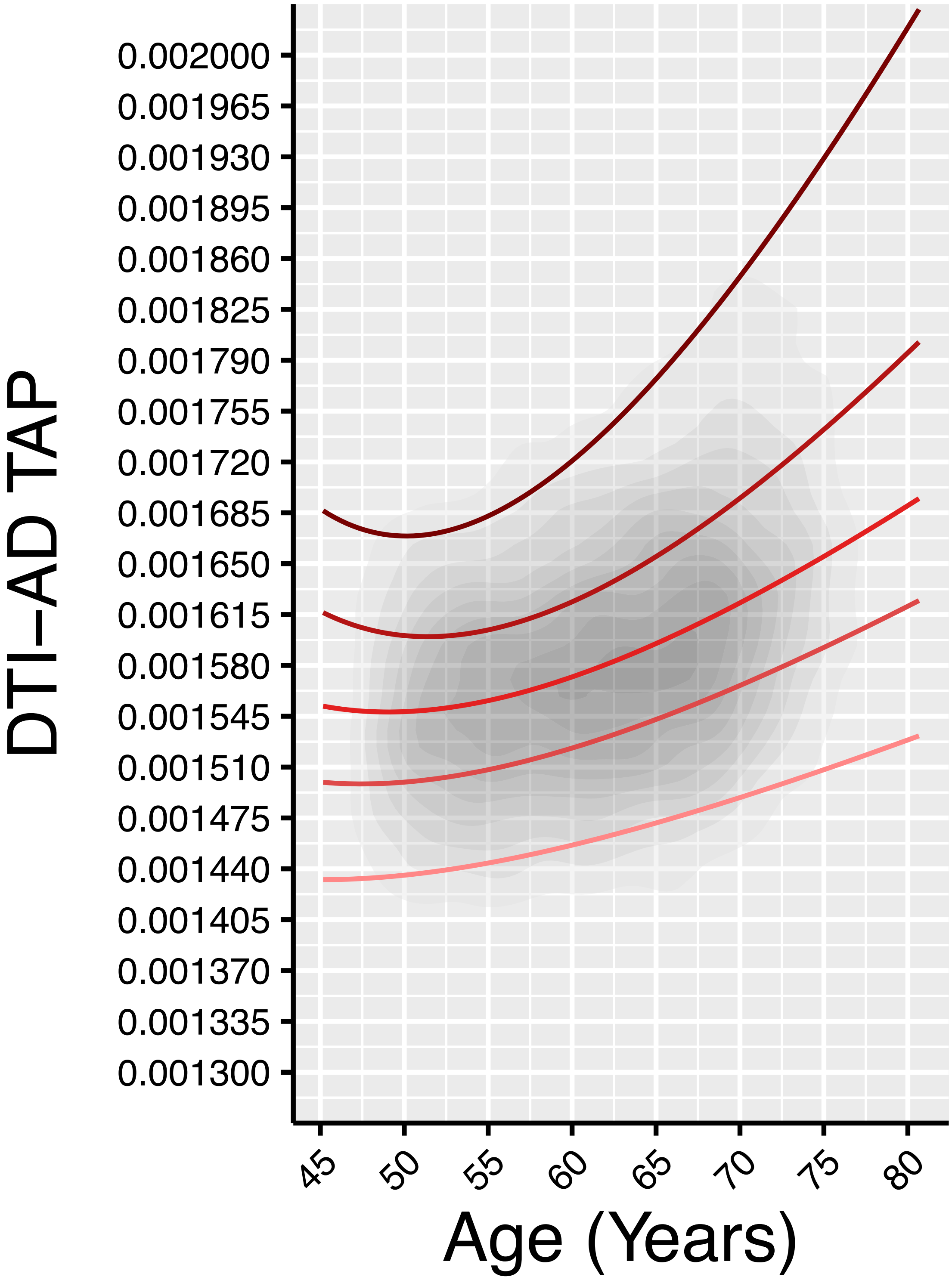

Figure S337. Full size normative centile reference curves calculated for the tapetum tract for DTI-AD in females. Solid colored lines, ordered from lightest to darkest, indicate the following centiles: 5th, 25th, 50th, 75th, 95th. Gray overlay reflects kernel density (darker=greater degree of data point overlap). TAP = tapetum.

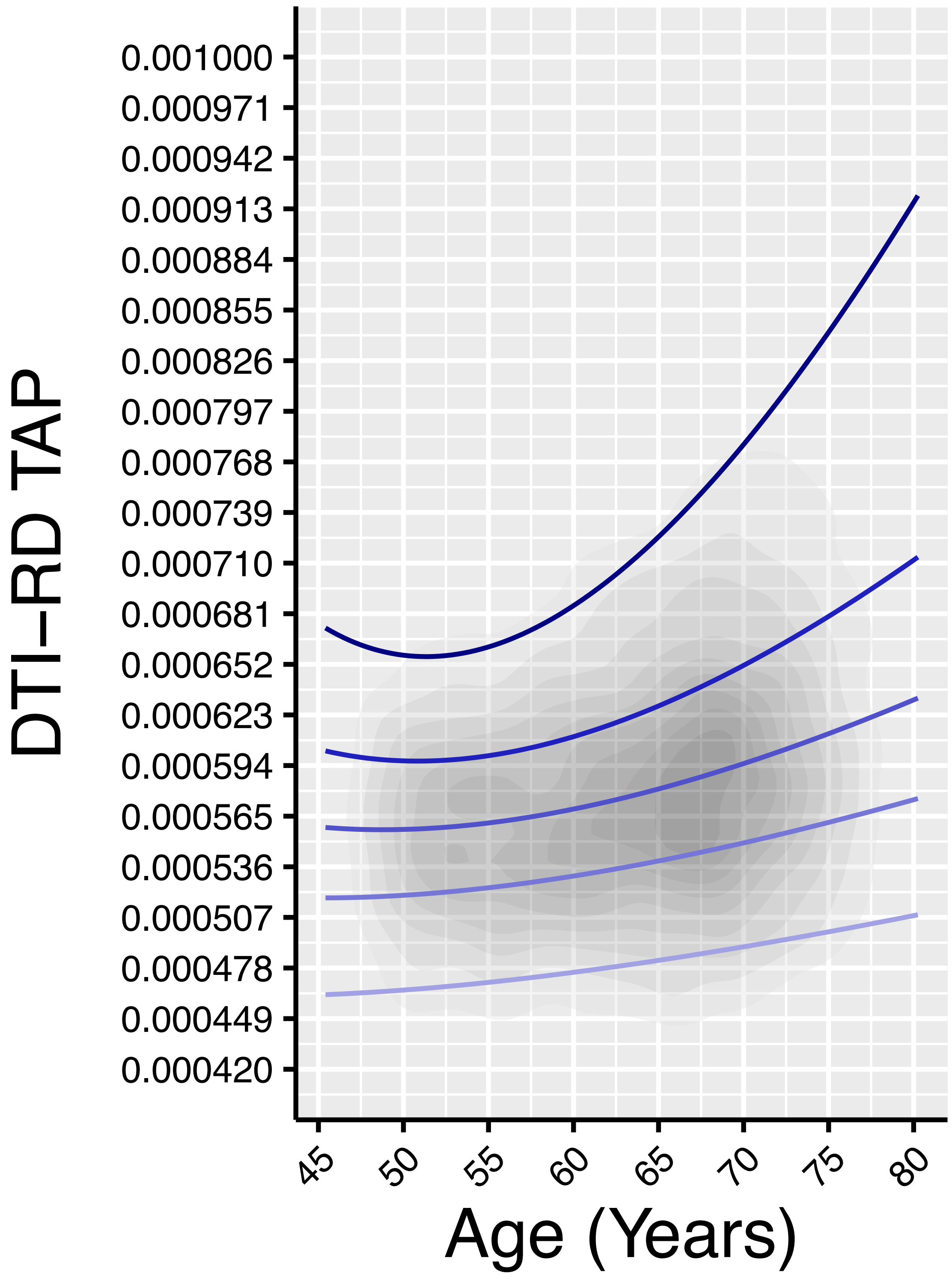

Figure S338. Full size normative centile reference curves calculated for the tapetum tract for DTI-RD in males. Solid colored lines, ordered from lightest to darkest, indicate the following centiles: 5th, 25th, 50th, 75th, 95th. Gray overlay reflects kernel density (darker=greater degree of data point overlap). TAP = tapetum.

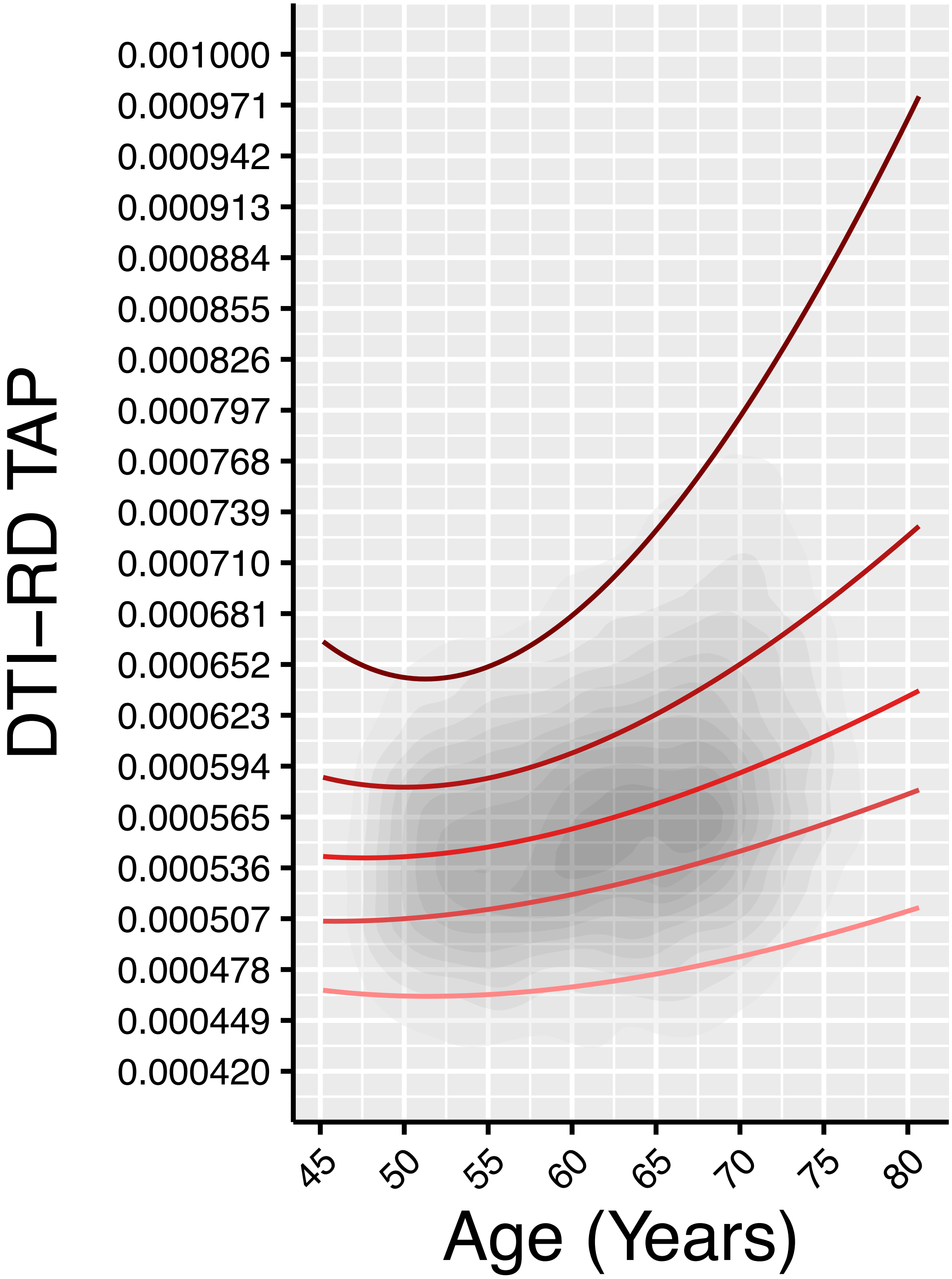

Figure S339. Full size normative centile reference curves calculated for the tapetum tract for DTI-RD in females. Solid colored lines, ordered from lightest to darkest, indicate the following centiles: 5th, 25th, 50th, 75th, 95th. Gray overlay reflects kernel density (darker=greater degree of data point overlap). TAP = tapetum.

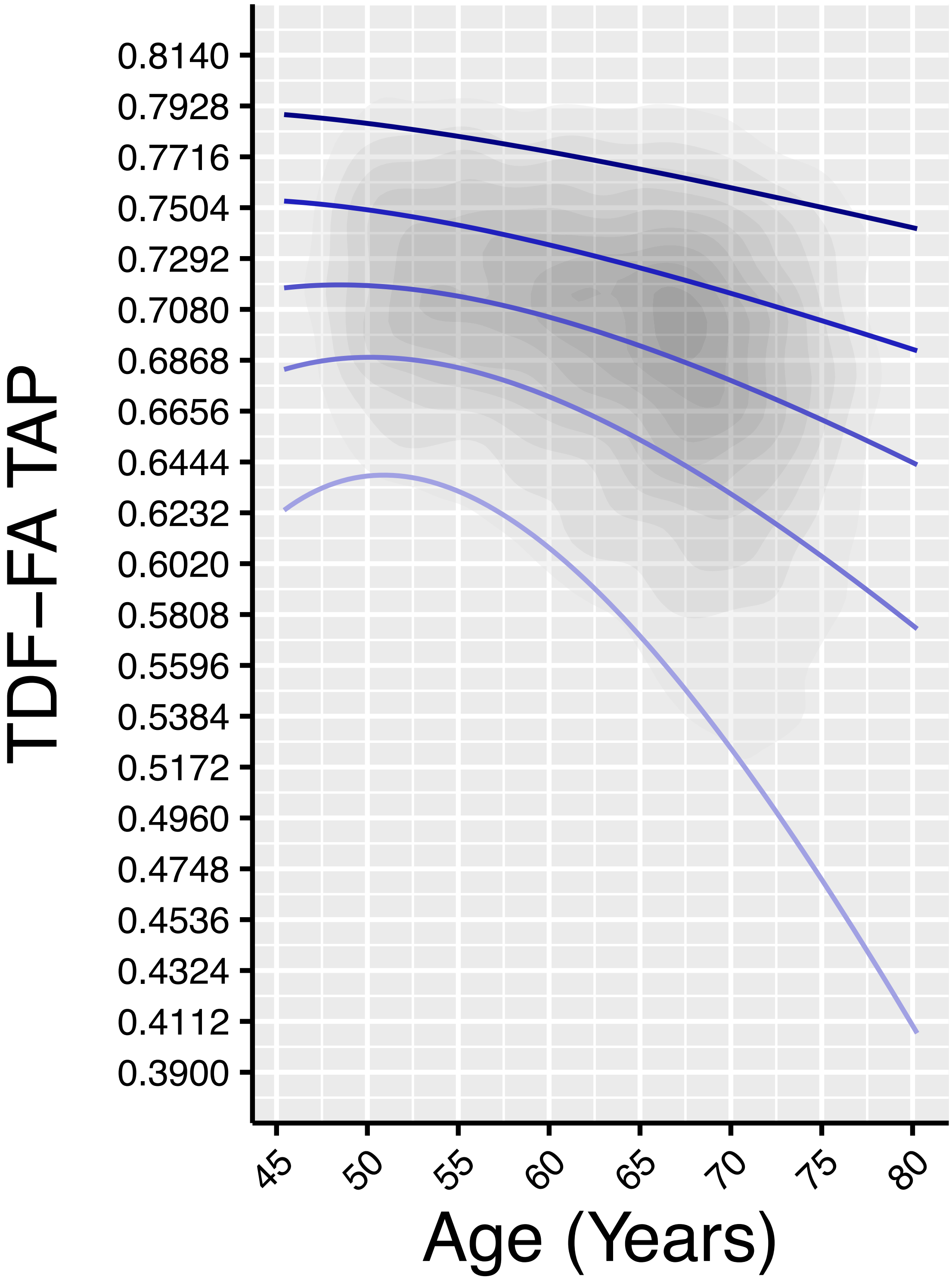

Figure S340. Full size normative centile reference curves calculated for the tapetum tract for TDF-FA in males. Solid colored lines, ordered from lightest to darkest, indicate the following centiles: 5th, 25th, 50th, 75th, 95th. Gray overlay reflects kernel density (darker=greater degree of data point overlap). TAP = tapetum.

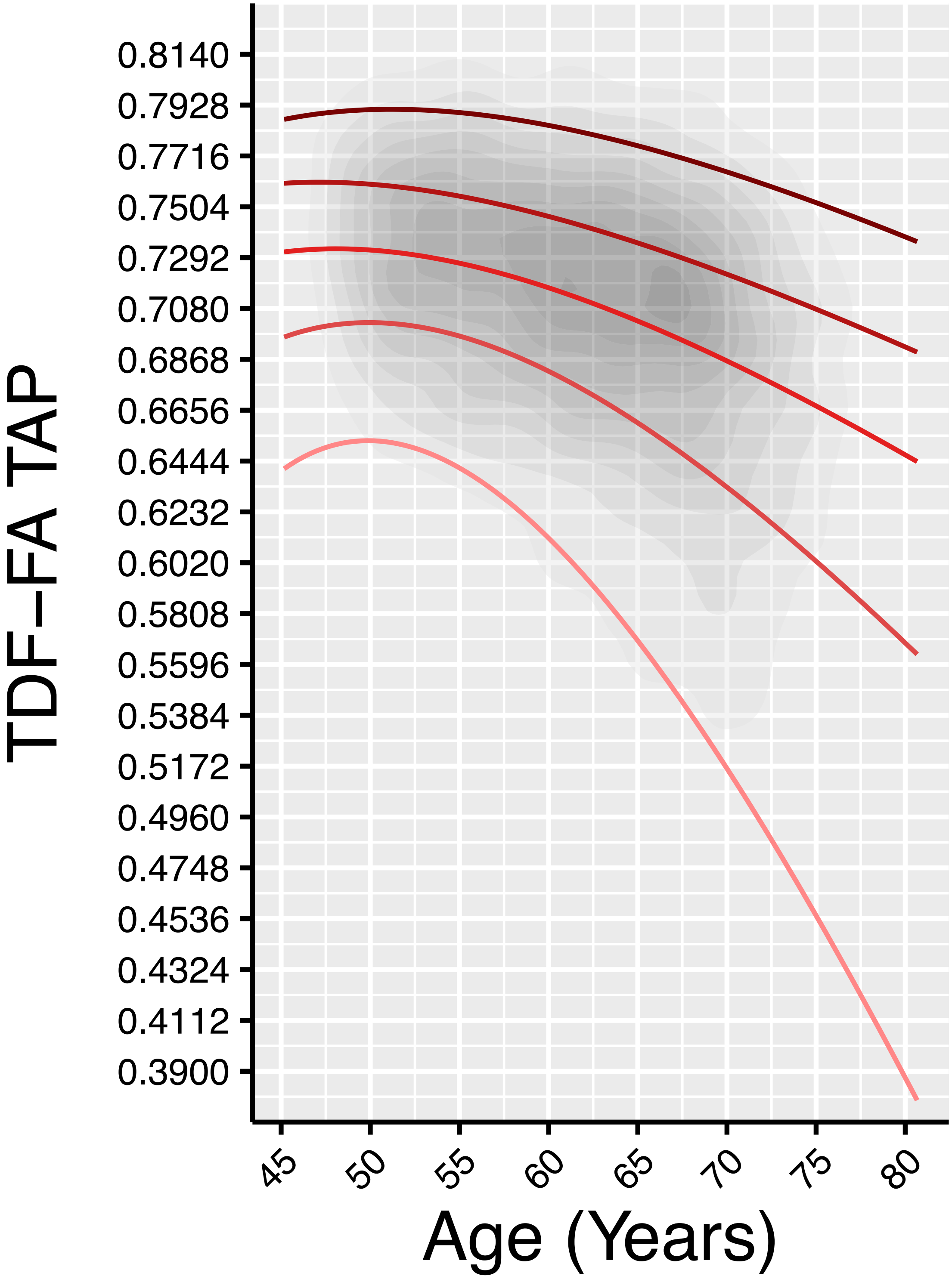

Figure S341. Full size normative centile reference curves calculated for the tapetum tract for TDF-FA in females. Solid colored lines, ordered from lightest to darkest, indicate the following centiles: 5th, 25th, 50th, 75th, 95th. Gray overlay reflects kernel density (darker=greater degree of data point overlap). TAP = tapetum.

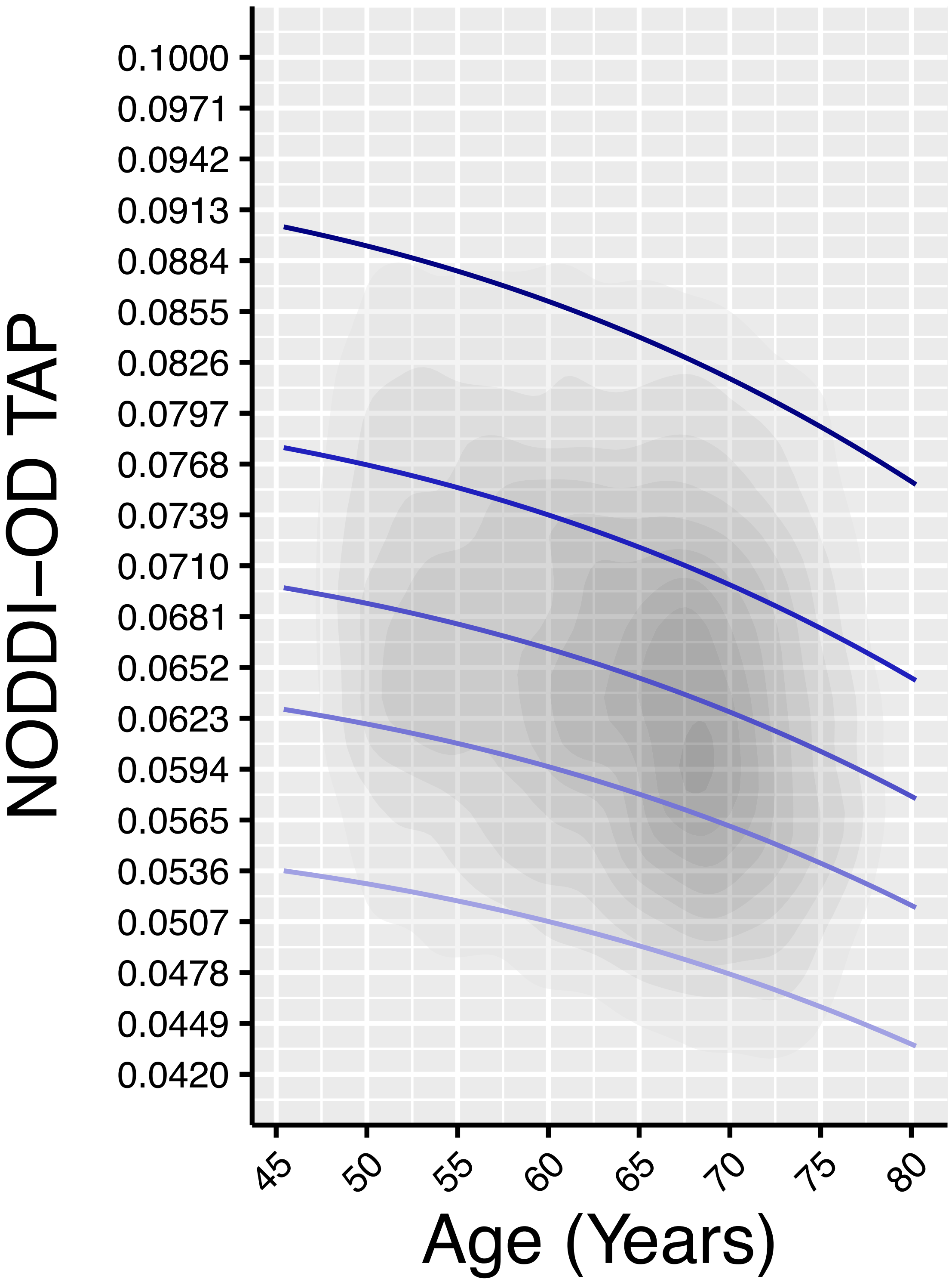

Figure S342. Full size normative centile reference curves calculated for the tapetum tract for NODDI-OD in males. Solid colored lines, ordered from lightest to darkest, indicate the following centiles: 5th, 25th, 50th, 75th, 95th. Gray overlay reflects kernel density (darker=greater degree of data point overlap). TAP = tapetum.

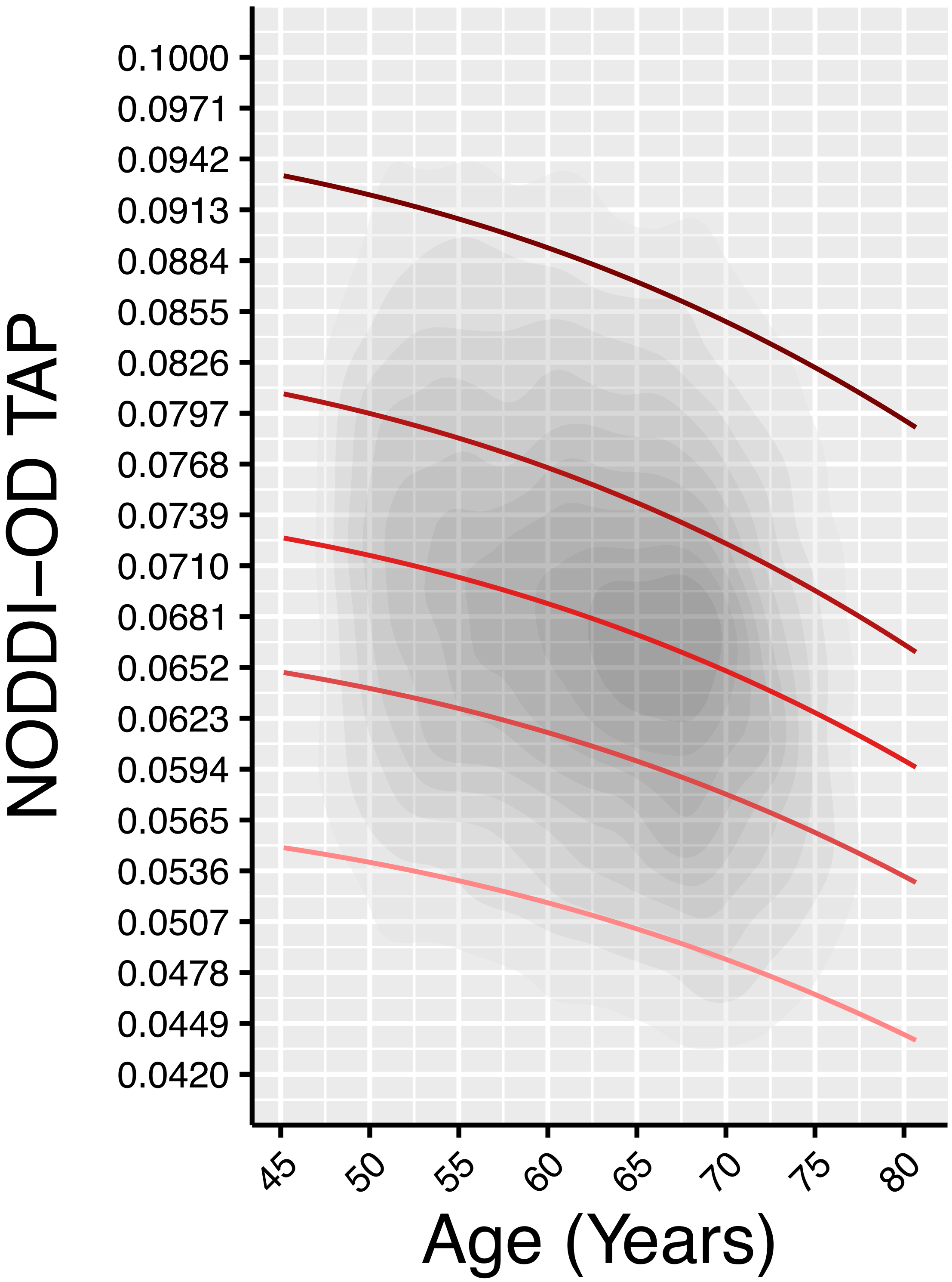

Figure S343. Full size normative centile reference curves calculated for the tapetum tract for NODDI-OD in females. Solid colored lines, ordered from lightest to darkest, indicate the following centiles: 5th, 25th, 50th, 75th, 95th. Gray overlay reflects kernel density (darker=greater degree of data point overlap). TAP = tapetum.

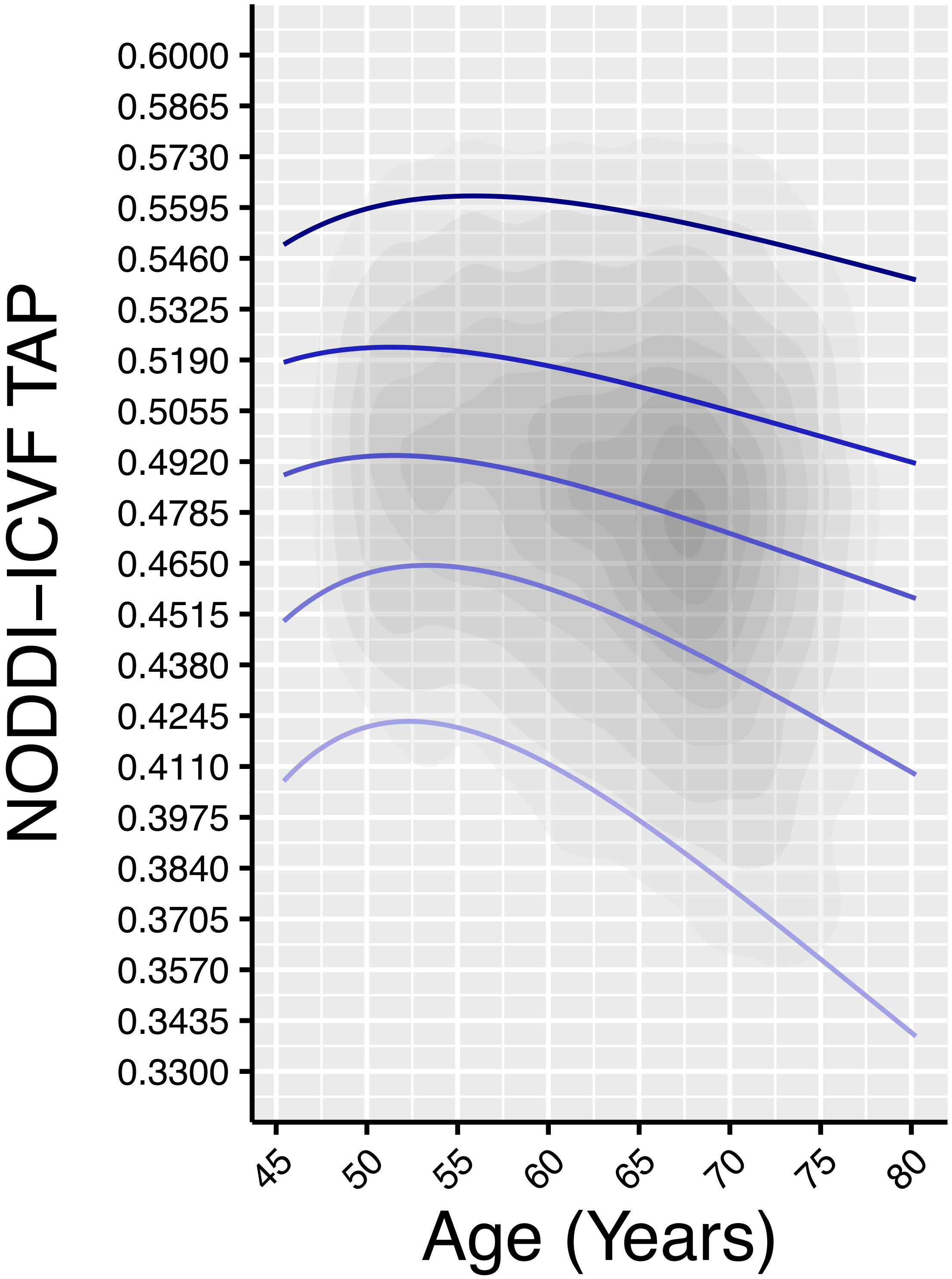

Figure S344. Full size normative centile reference curves calculated for the tapetum tract for NODDI-ICVF in males. Solid colored lines, ordered from lightest to darkest, indicate the following centiles: 5th, 25th, 50th, 75th, 95th. Gray overlay reflects kernel density (darker=greater degree of data point overlap). TAP = tapetum.

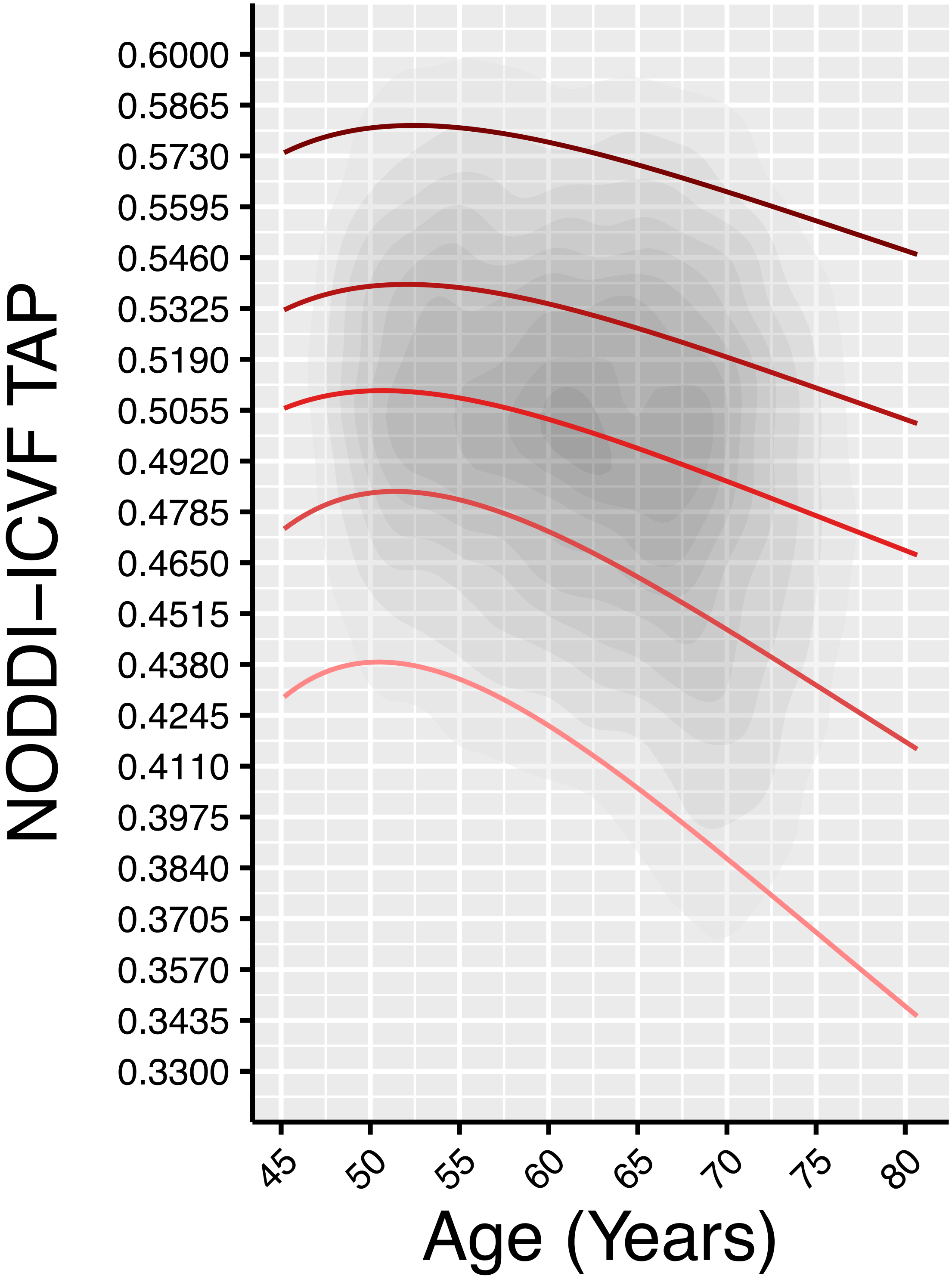

Figure S345. Full size normative centile reference curves calculated for the tapetum tract for NODDI-ICVF in females. Solid colored lines, ordered from lightest to darkest, indicate the following centiles: 5th, 25th, 50th, 75th, 95th. Gray overlay reflects kernel density (darker=greater degree of data point overlap). TAP = tapetum.

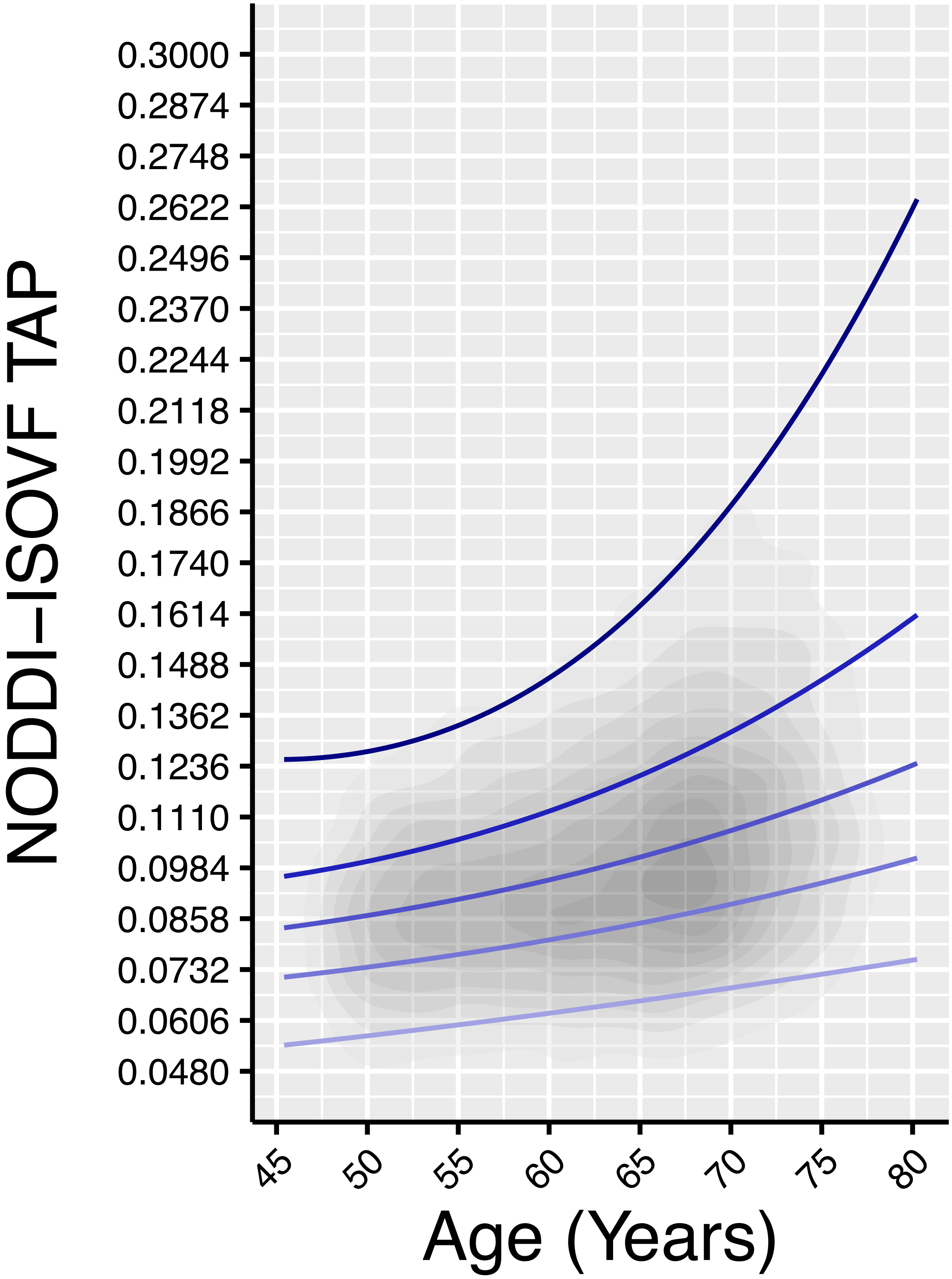

Figure S346. Full size normative centile reference curves calculated for the tapetum tract for NODDI-ISOVF in males. Solid colored lines, ordered from lightest to darkest, indicate the following centiles: 5th, 25th, 50th, 75th, 95th. Gray overlay reflects kernel density (darker=greater degree of data point overlap). TAP = tapetum.

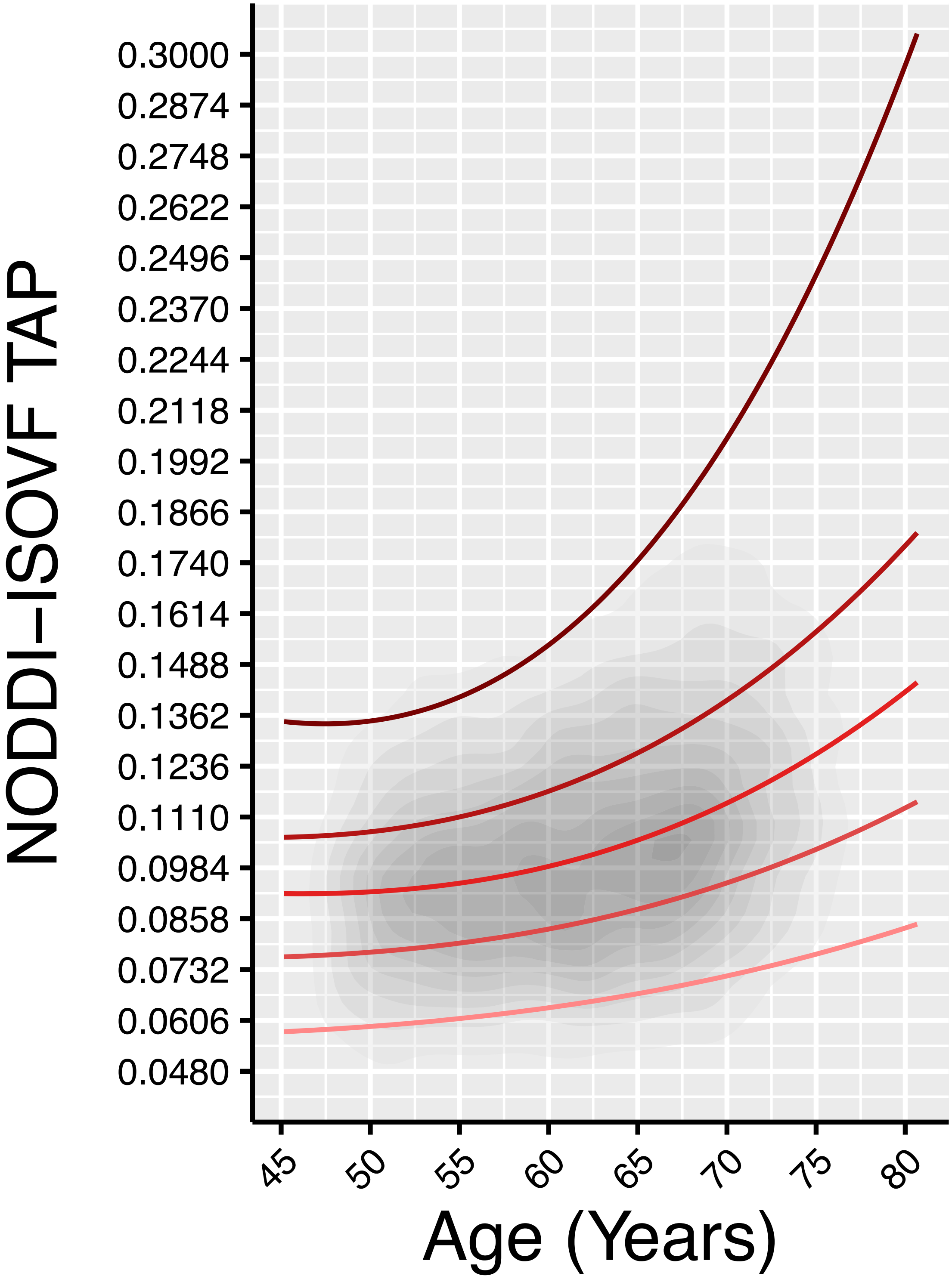

Figure S347. Full size normative centile reference curves calculated for the tapetum tract for NODDI-ISOVF in females. Solid colored lines, ordered from lightest to darkest, indicate the following centiles: 5th, 25th, 50th, 75th, 95th. Gray overlay reflects kernel density (darker=greater degree of data point overlap). TAP = tapetum.

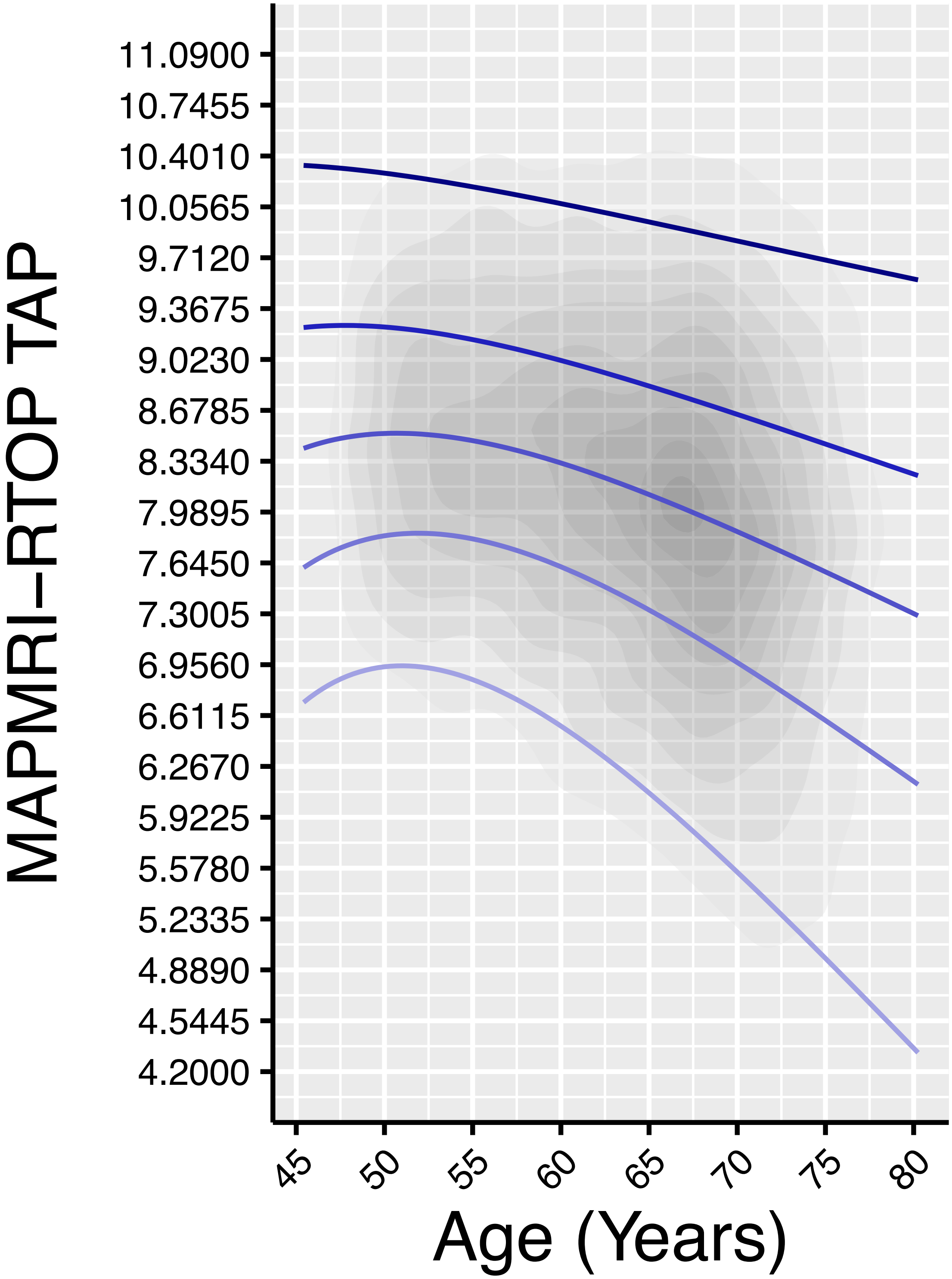

Figure S348. Full size normative centile reference curves calculated for the tapetum tract for MAPMRI-RTOP in males. Solid colored lines, ordered from lightest to darkest, indicate the following centiles: 5th, 25th, 50th, 75th, 95th. Gray overlay reflects kernel density (darker=greater degree of data point overlap). TAP = tapetum.

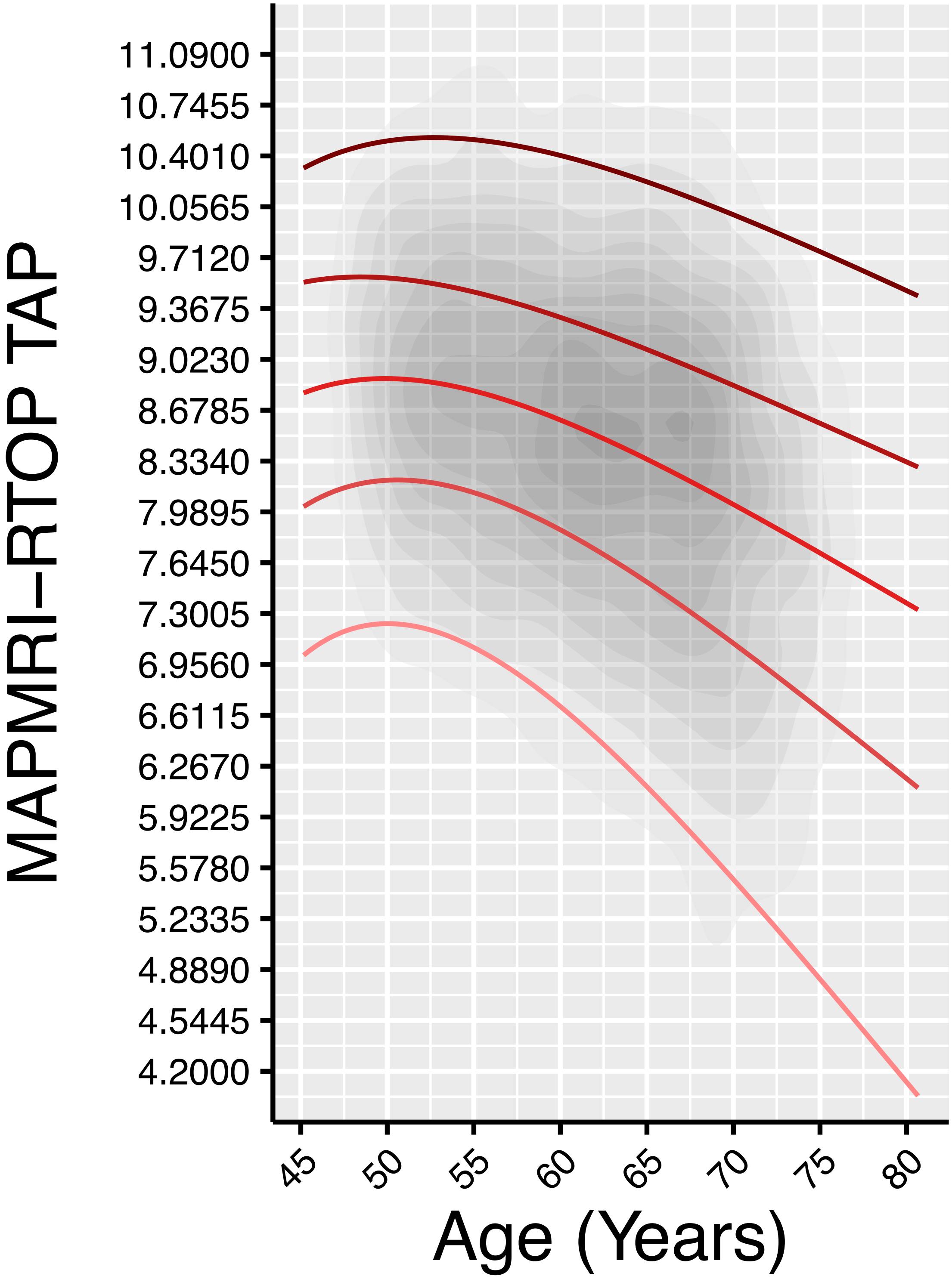

Figure S349. Full size normative centile reference curves calculated for the tapetum tract for MAPMRI-RTOP in females. Solid colored lines, ordered from lightest to darkest, indicate the following centiles: 5th, 25th, 50th, 75th, 95th. Gray overlay reflects kernel density (darker=greater degree of data point overlap). TAP = tapetum.

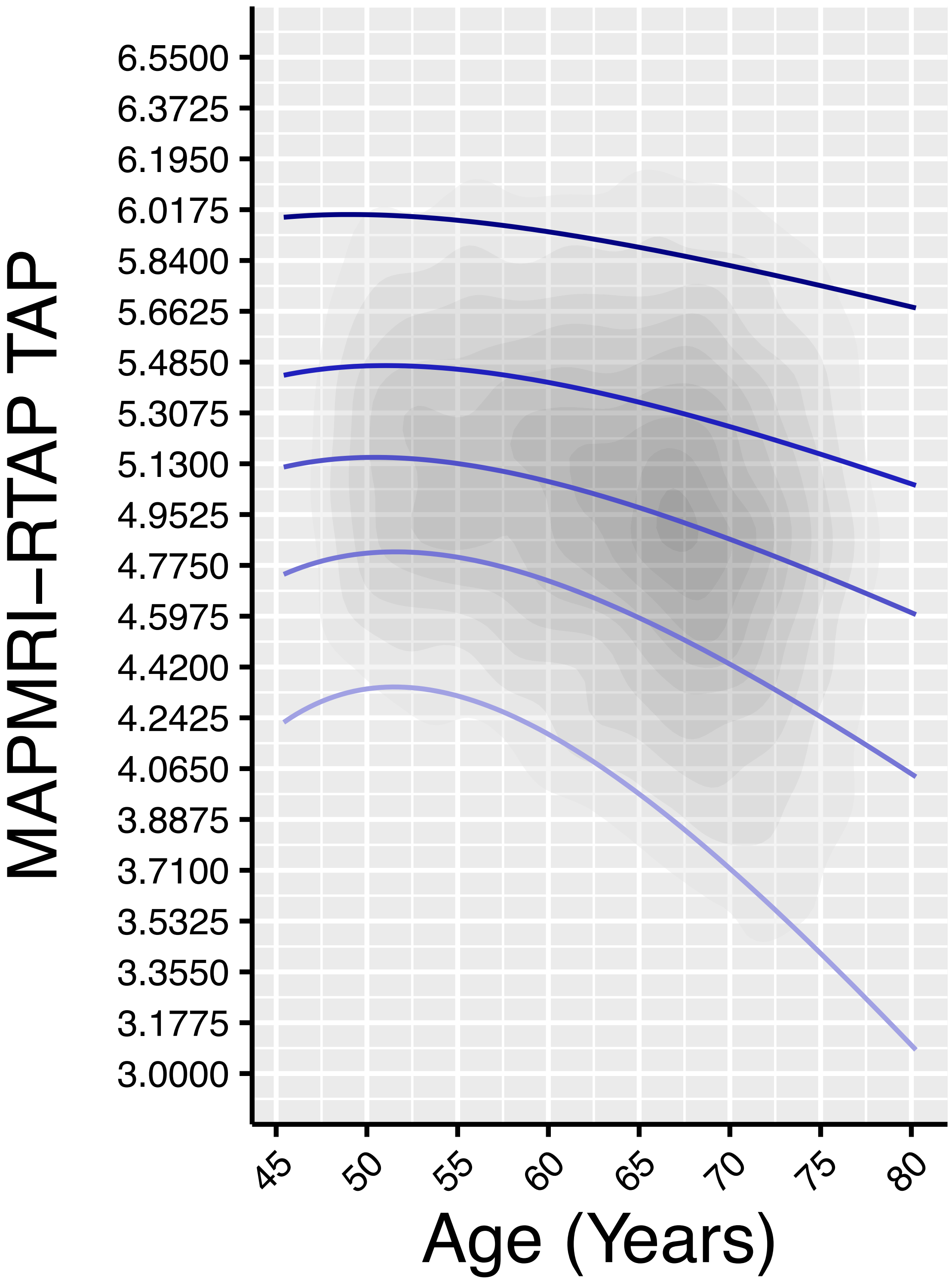

Figure S350. Full size normative centile reference curves calculated for the tapetum tract for MAPMRI-RTAP in males. Solid colored lines, ordered from lightest to darkest, indicate the following centiles: 5th, 25th, 50th, 75th, 95th. Gray overlay reflects kernel density (darker=greater degree of data point overlap). TAP = tapetum.

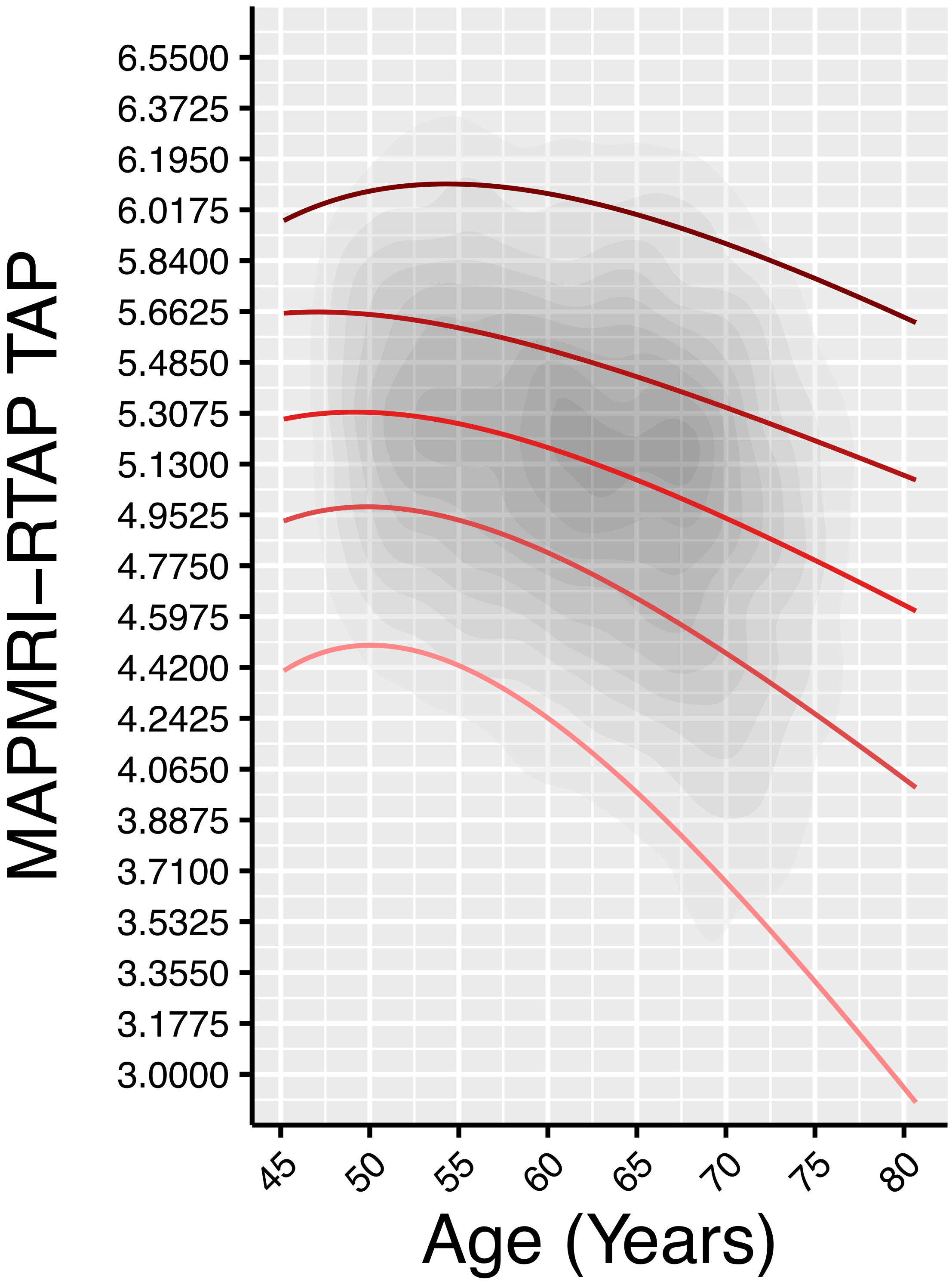

Figure S351. Full size normative centile reference curves calculated for the tapetum tract for MAPMRI-RTAP in females. Solid colored lines, ordered from lightest to darkest, indicate the following centiles: 5th, 25th, 50th, 75th, 95th. Gray overlay reflects kernel density (darker=greater degree of data point overlap). TAP = tapetum.

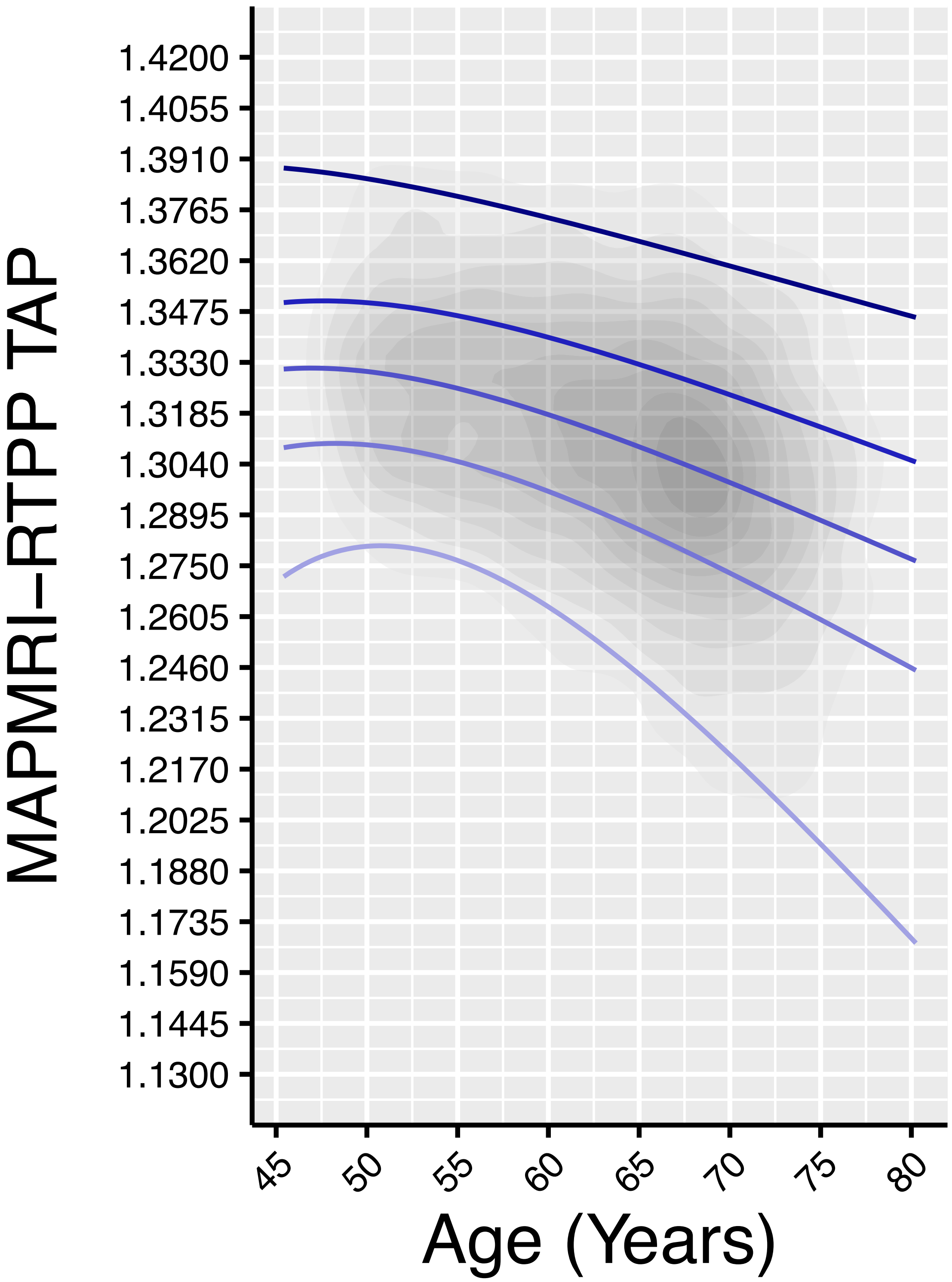

Figure S352. Full size normative centile reference curves calculated for the tapetum tract for MAPMRI-RTPP in males. Solid colored lines, ordered from lightest to darkest, indicate the following centiles: 5th, 25th, 50th, 75th, 95th. Gray overlay reflects kernel density (darker=greater degree of data point overlap). TAP = tapetum.

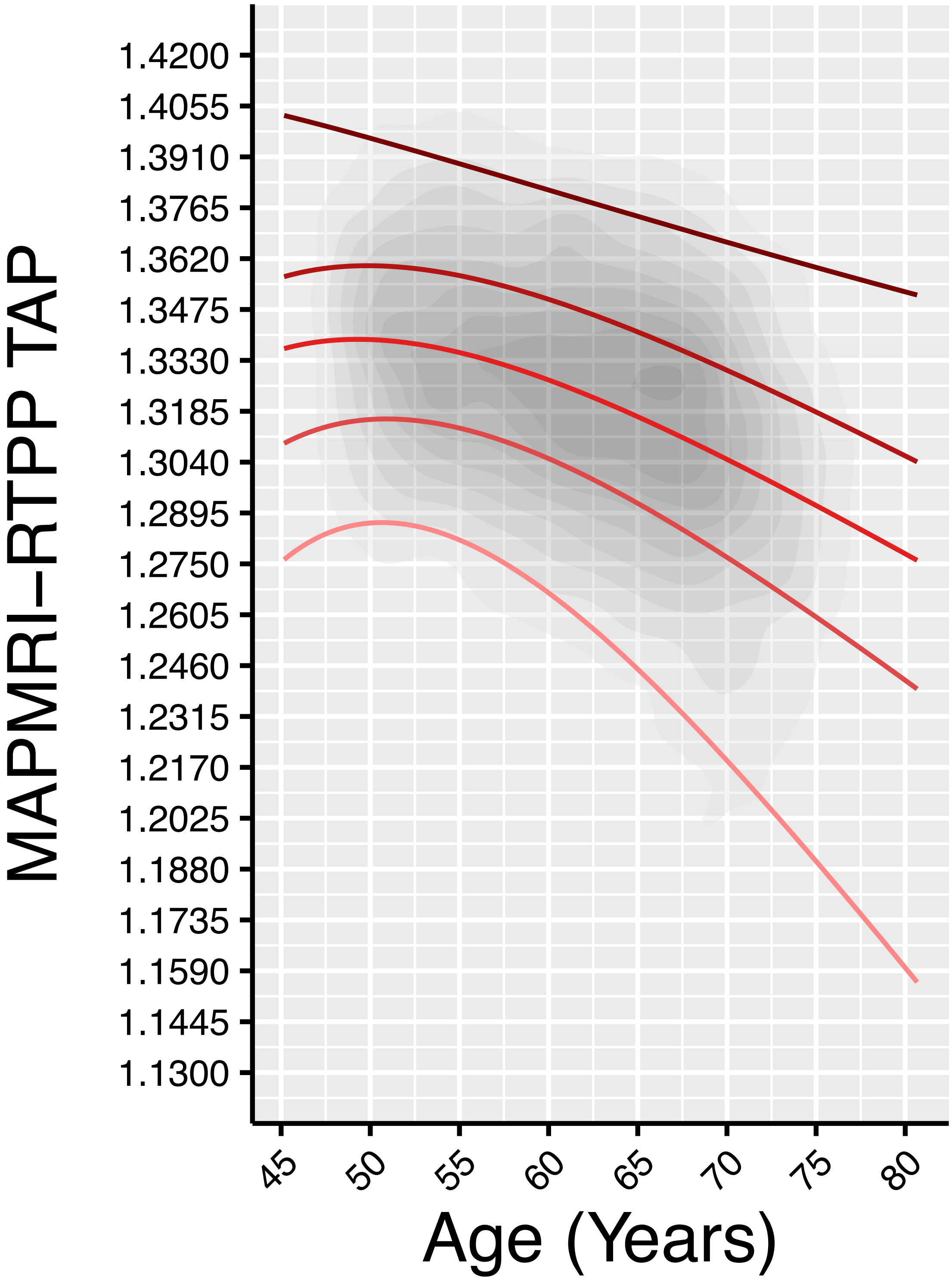

Figure S353. Full size normative centile reference curves calculated for the tapetum tract for MAPMRI-RTPP in females. Solid colored lines, ordered from lightest to darkest, indicate the following centiles: 5th, 25th, 50th, 75th, 95th. Gray overlay reflects kernel density (darker=greater degree of data point overlap). TAP = tapetum.

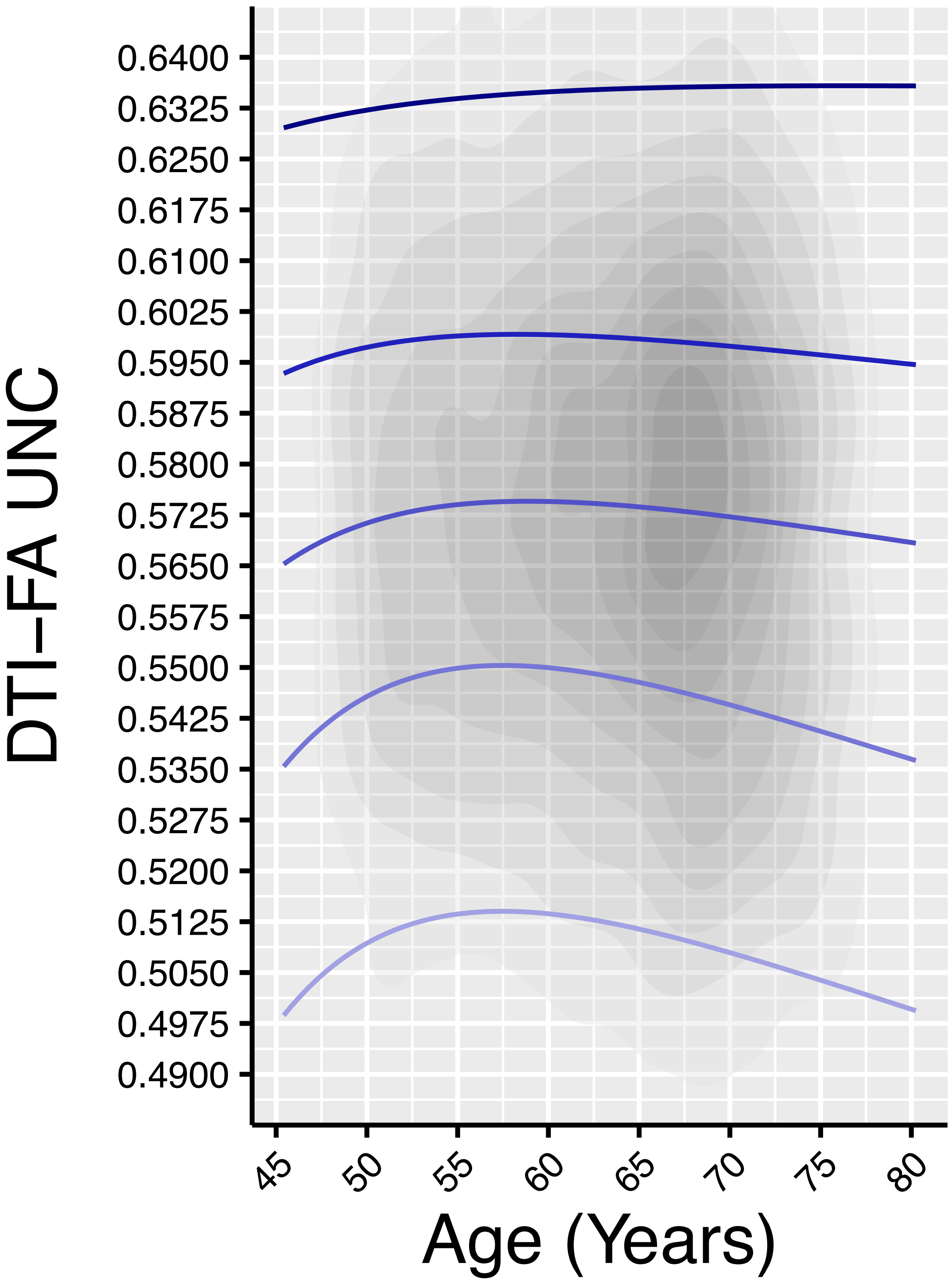

Figure S354. Full size normative centile reference curves calculated for the uncinate fasciculus tract for DTI-FA in males. Solid colored lines, ordered from lightest to darkest, indicate the following centiles: 5th, 25th, 50th, 75th, 95th. Gray overlay reflects kernel density (darker=greater degree of data point overlap). UNC = uncinate fasciculus.

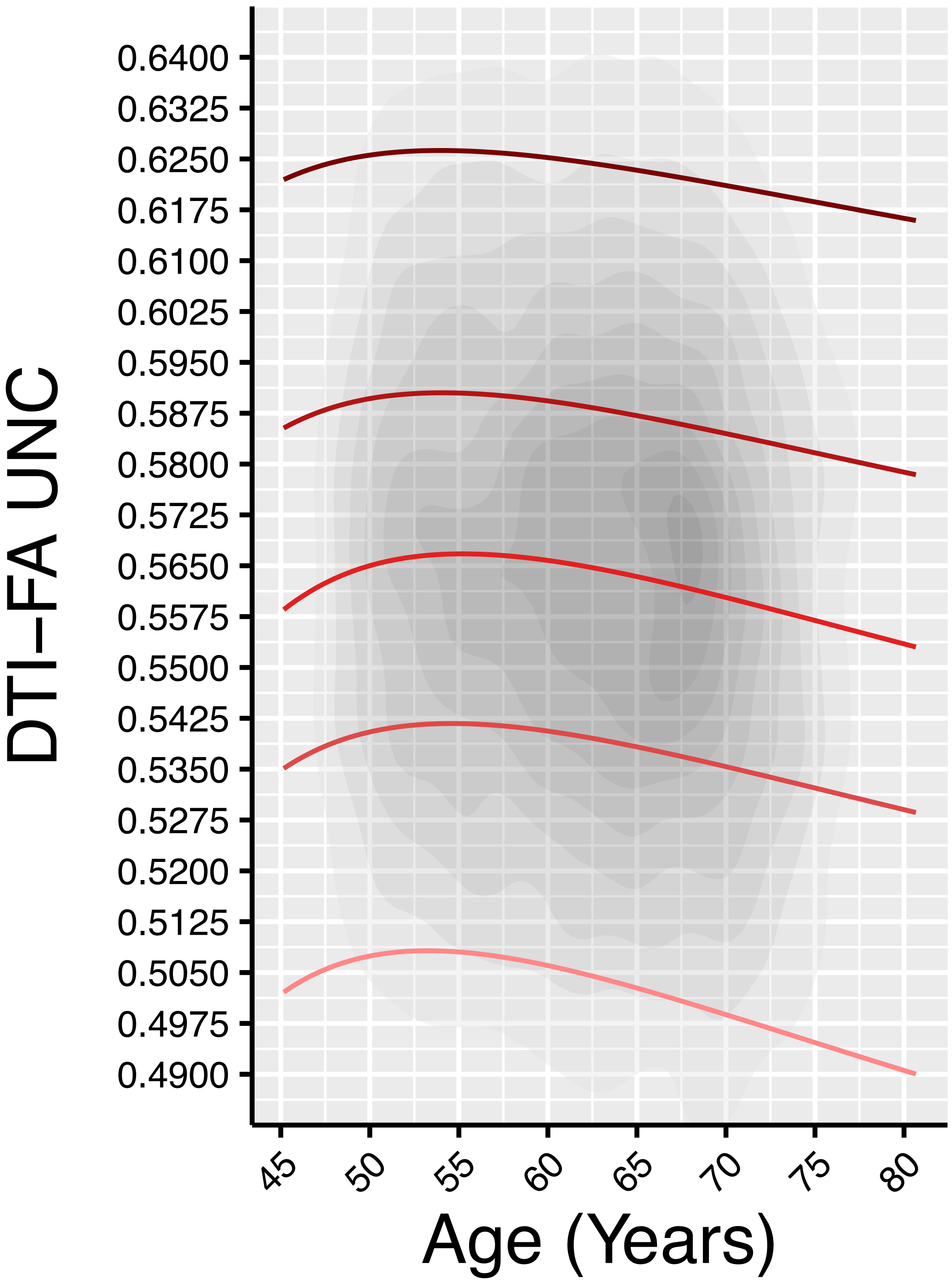

Figure S355. Full size normative centile reference curves calculated for the uncinate fasciculus tract for DTI-FA in females. Solid colored lines, ordered from lightest to darkest, indicate the following centiles: 5th, 25th, 50th, 75th, 95th. Gray overlay reflects kernel density (darker=greater degree of data point overlap). UNC = uncinate fasciculus.

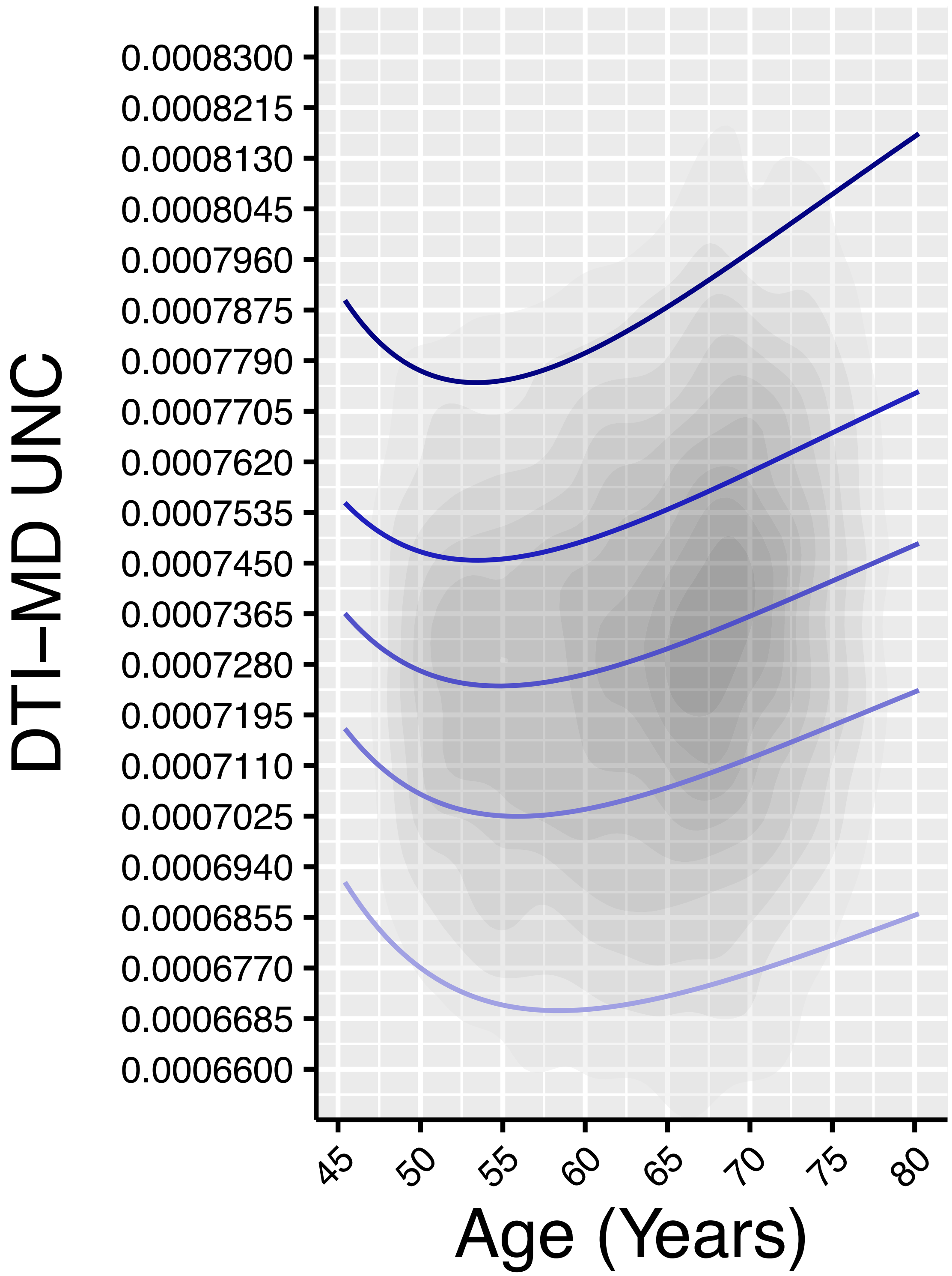

Figure S356. Full size normative centile reference curves calculated for the uncinate fasciculus tract for DTI-MD in males. Solid colored lines, ordered from lightest to darkest, indicate the following centiles: 5th, 25th, 50th, 75th, 95th. Gray overlay reflects kernel density (darker=greater degree of data point overlap). UNC = uncinate fasciculus.

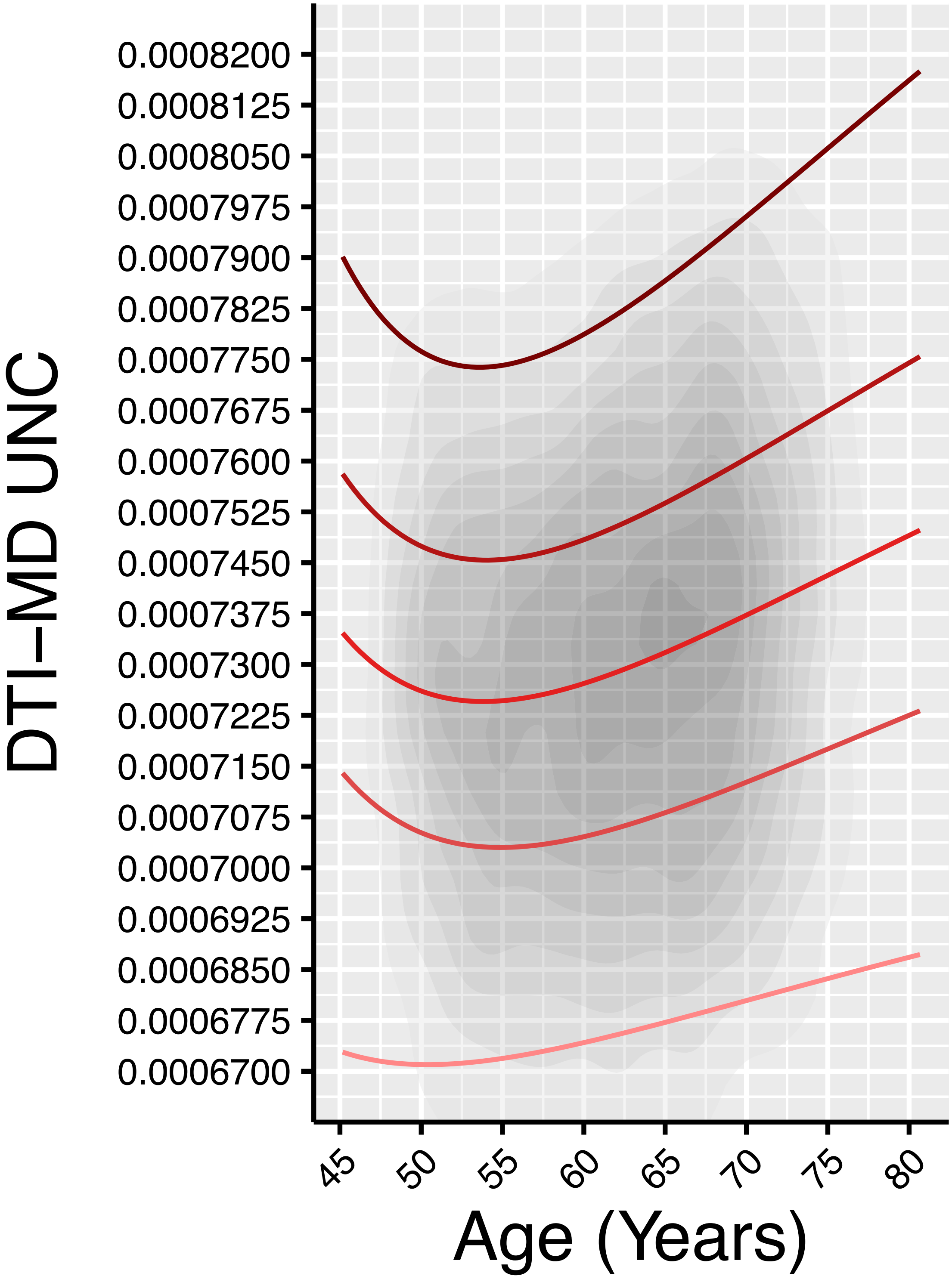

Figure S357. Full size normative centile reference curves calculated for the uncinate fasciculus tract for DTI-MD in females. Solid colored lines, ordered from lightest to darkest, indicate the following centiles: 5th, 25th, 50th, 75th, 95th. Gray overlay reflects kernel density (darker=greater degree of data point overlap). UNC = uncinate fasciculus.

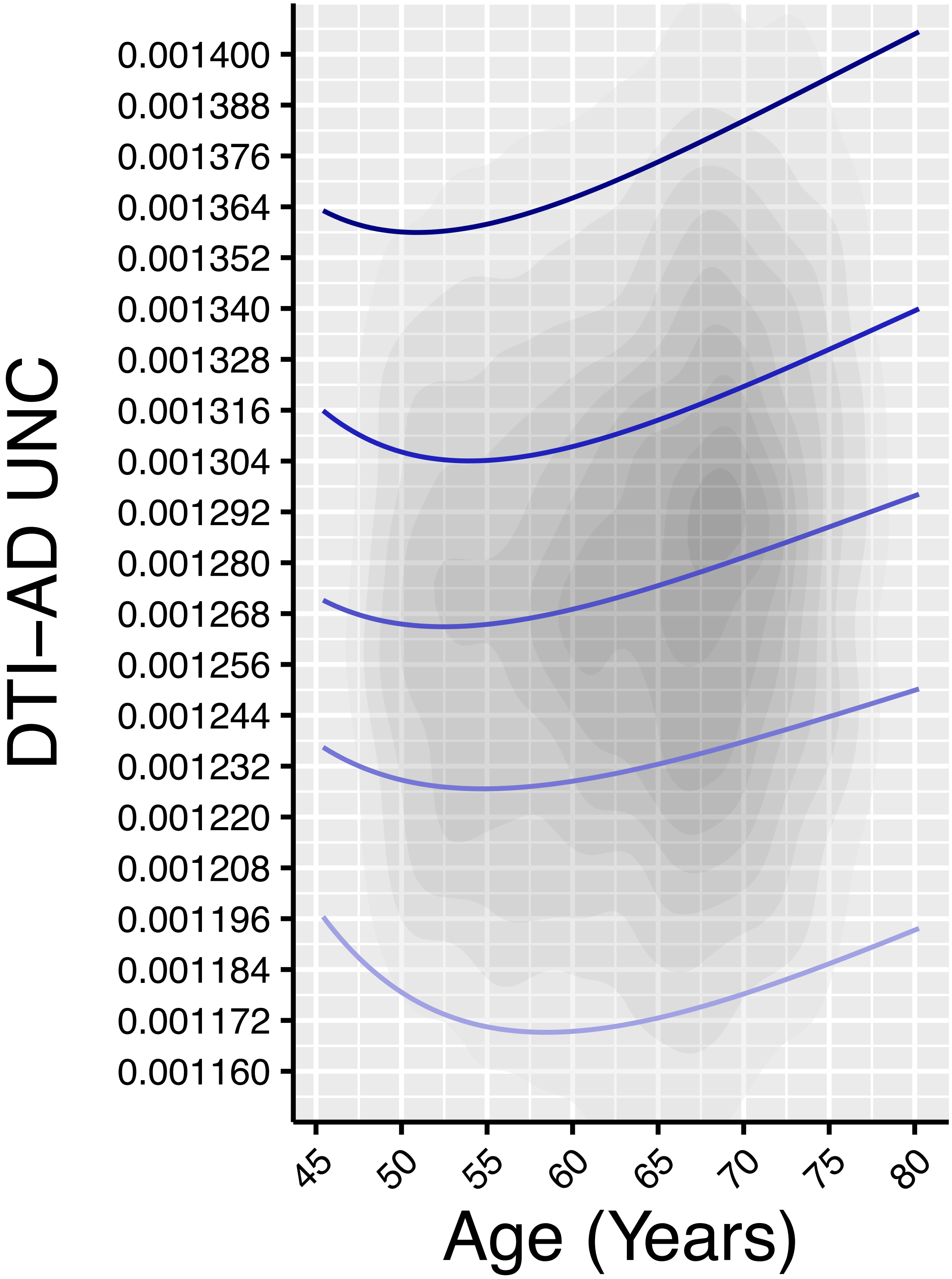

Figure S358. Full size normative centile reference curves calculated for the uncinate fasciculus tract for DTI-AD in males. Solid colored lines, ordered from lightest to darkest, indicate the following centiles: 5th, 25th, 50th, 75th, 95th. Gray overlay reflects kernel density (darker=greater degree of data point overlap). UNC = uncinate fasciculus.

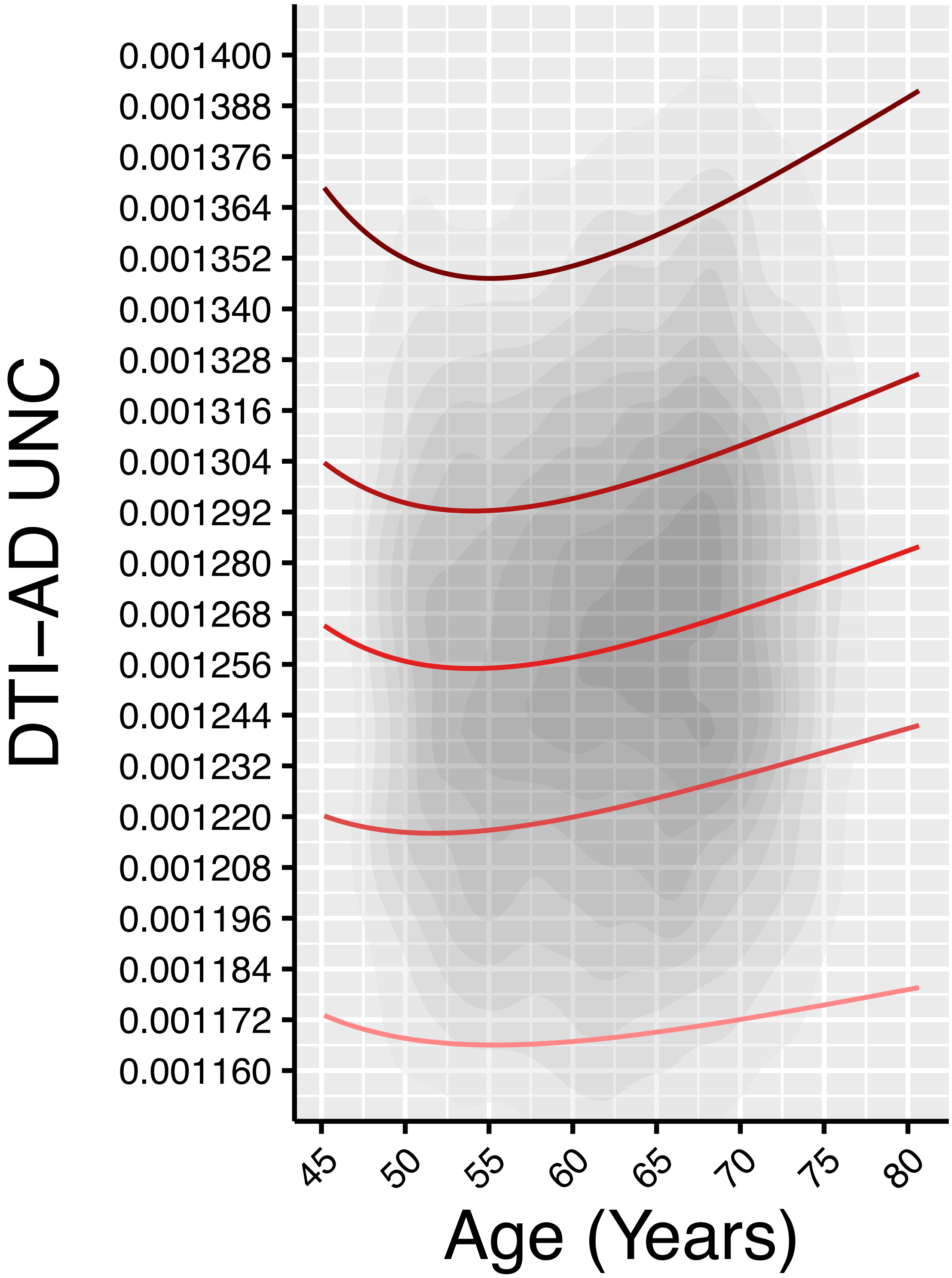

Figure S359. Full size normative centile reference curves calculated for the uncinate fasciculus tract for DTI-AD in females. Solid colored lines, ordered from lightest to darkest, indicate the following centiles: 5th, 25th, 50th, 75th, 95th. Gray overlay reflects kernel density (darker=greater degree of data point overlap). UNC = uncinate fasciculus.

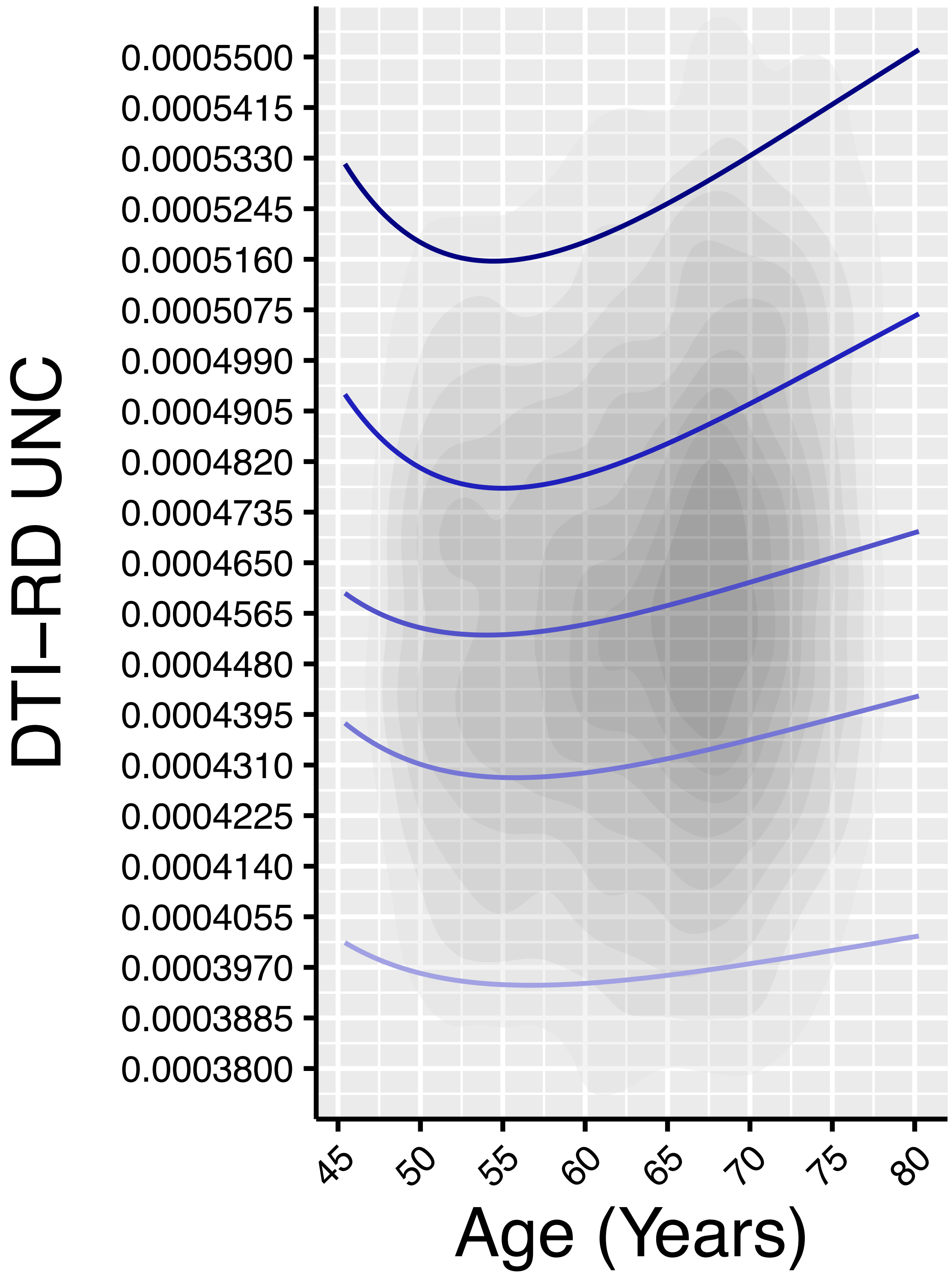

Figure S360. Full size normative centile reference curves calculated for the uncinate fasciculus tract for DTI-RD in males. Solid colored lines, ordered from lightest to darkest, indicate the following centiles: 5th, 25th, 50th, 75th, 95th. Gray overlay reflects kernel density (darker=greater degree of data point overlap). UNC = uncinate fasciculus.

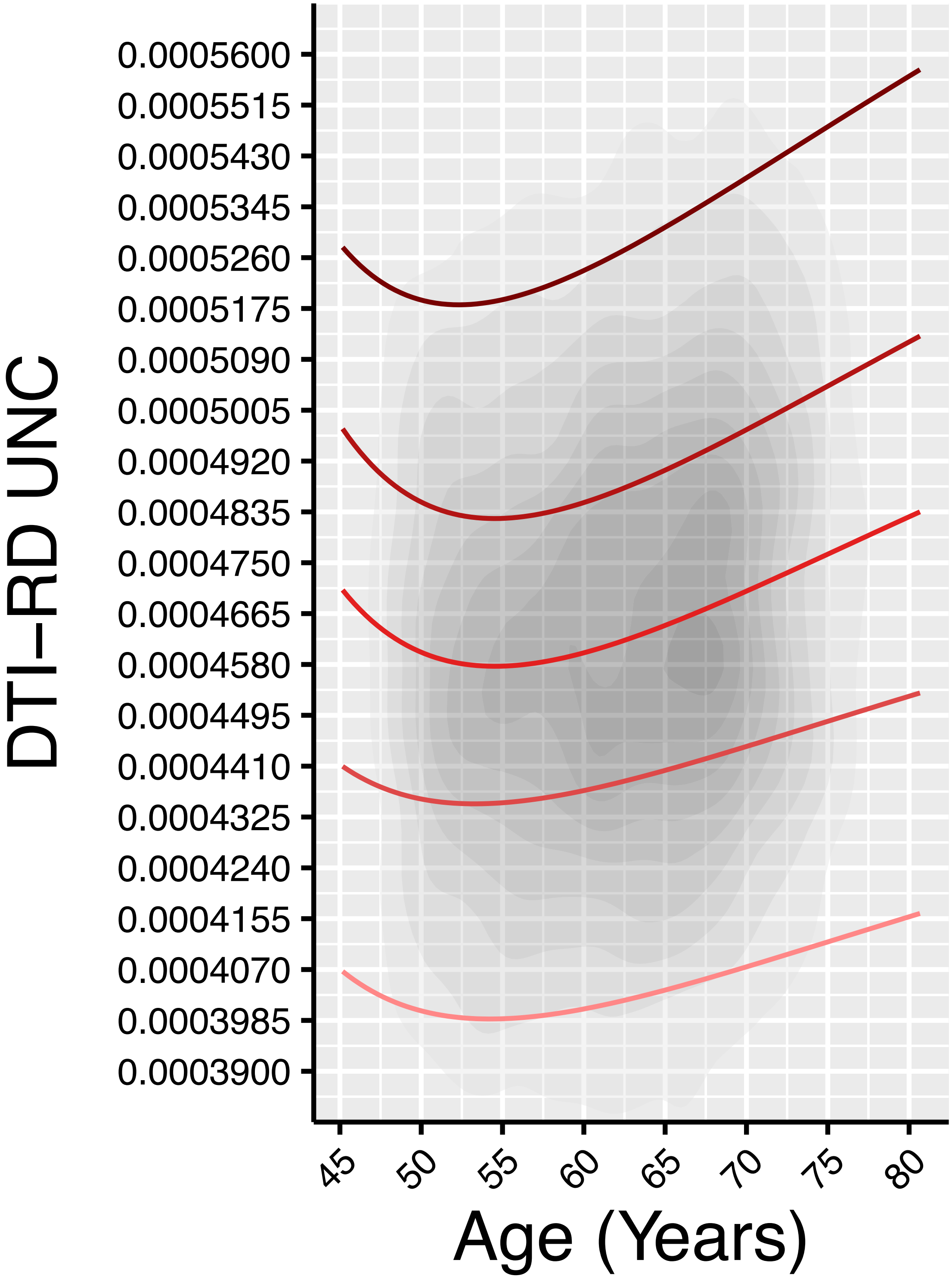

Figure S361. Full size normative centile reference curves calculated for the uncinate fasciculus tract for DTI-RD in females. Solid colored lines, ordered from lightest to darkest, indicate the following centiles: 5th, 25th, 50th, 75th, 95th. Gray overlay reflects kernel density (darker=greater degree of data point overlap). UNC = uncinate fasciculus.

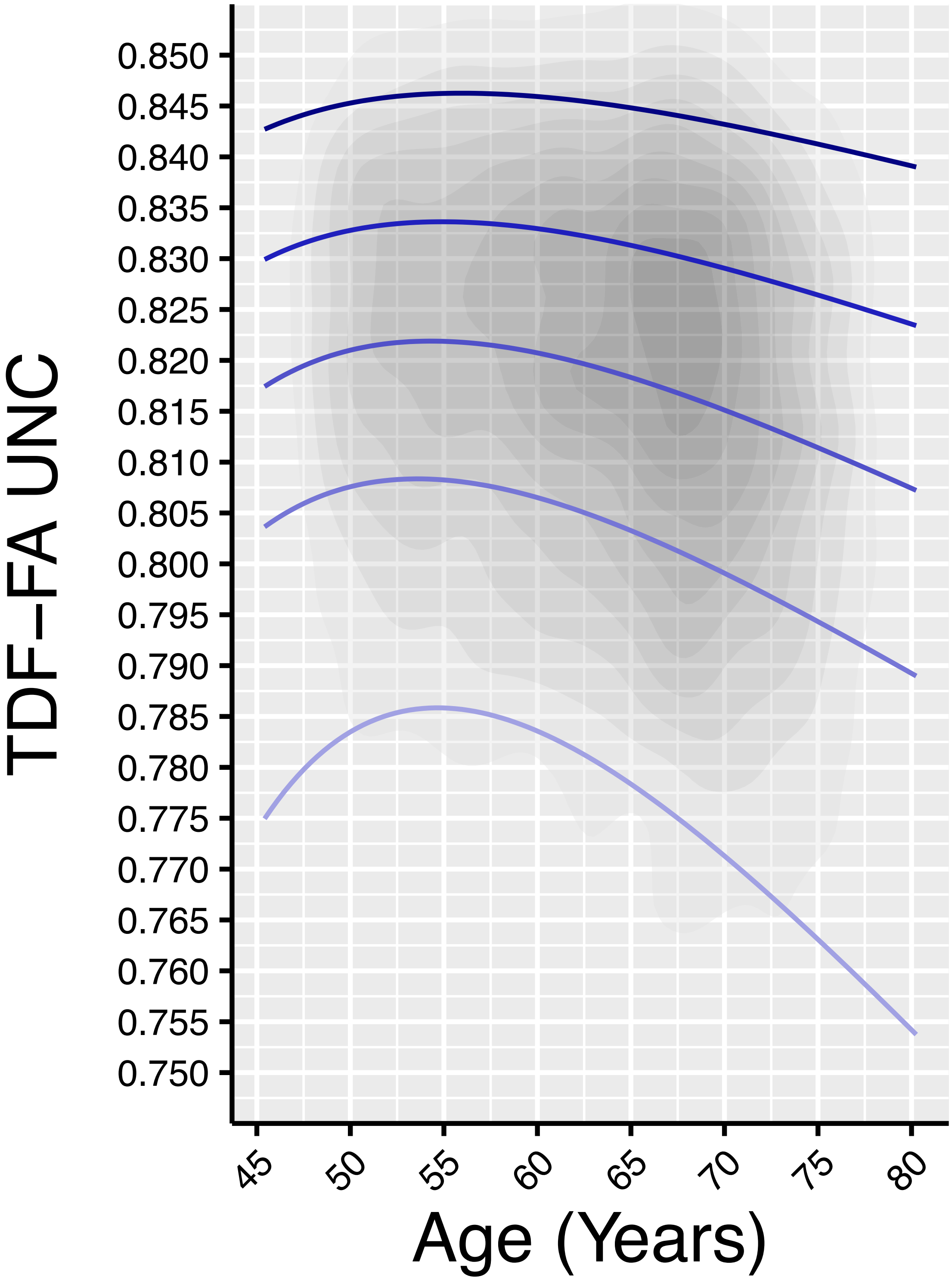

Figure S362. Full size normative centile reference curves calculated for the uncinate fasciculus tract for TDF-FA in males. Solid colored lines, ordered from lightest to darkest, indicate the following centiles: 5th, 25th, 50th, 75th, 95th. Gray overlay reflects kernel density (darker=greater degree of data point overlap). UNC = uncinate fasciculus.

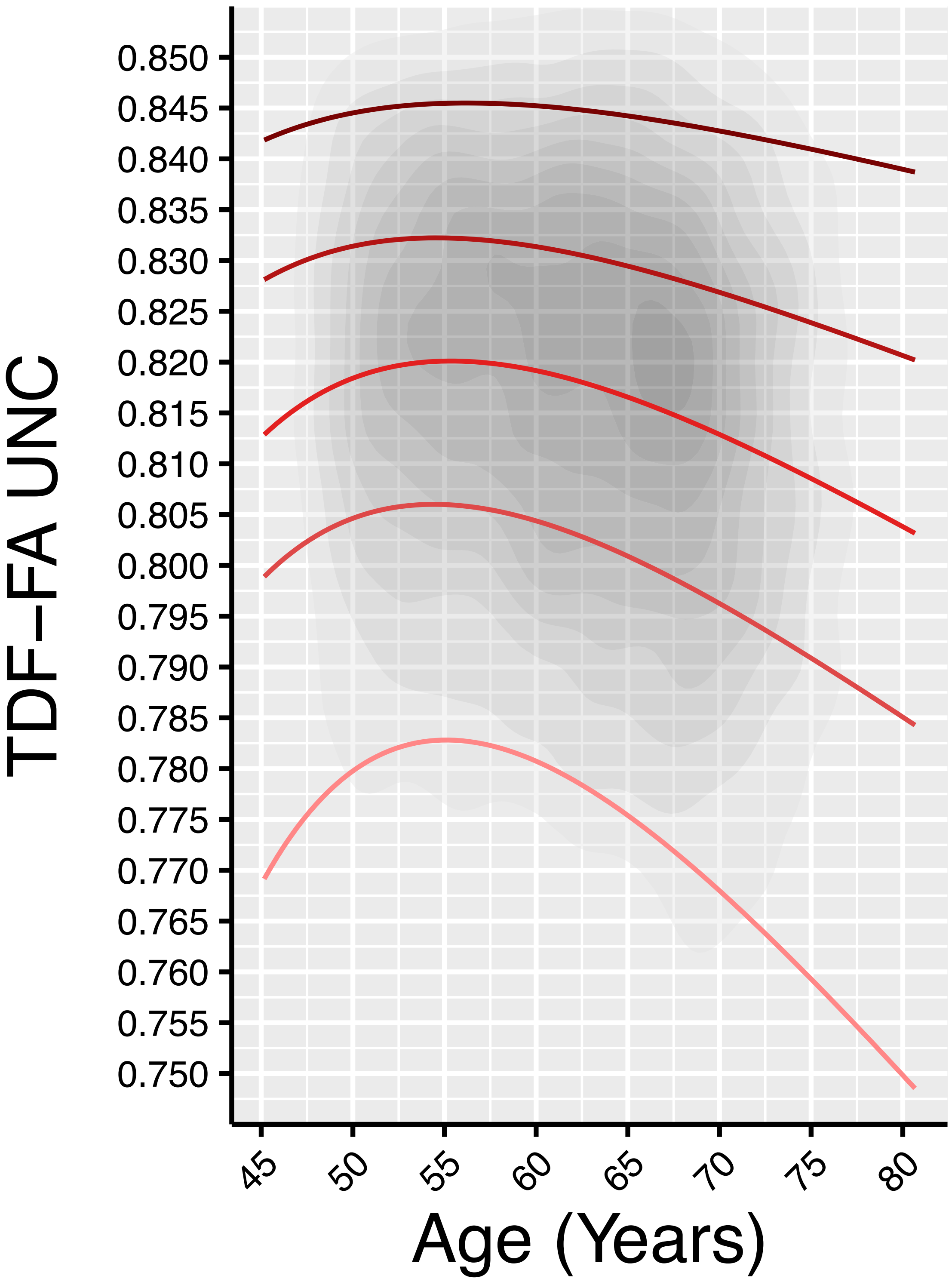

Figure S363. Full size normative centile reference curves calculated for the uncinate fasciculus tract for TDF-FA in females. Solid colored lines, ordered from lightest to darkest, indicate the following centiles: 5th, 25th, 50th, 75th, 95th. Gray overlay reflects kernel density (darker=greater degree of data point overlap). UNC = uncinate fasciculus.

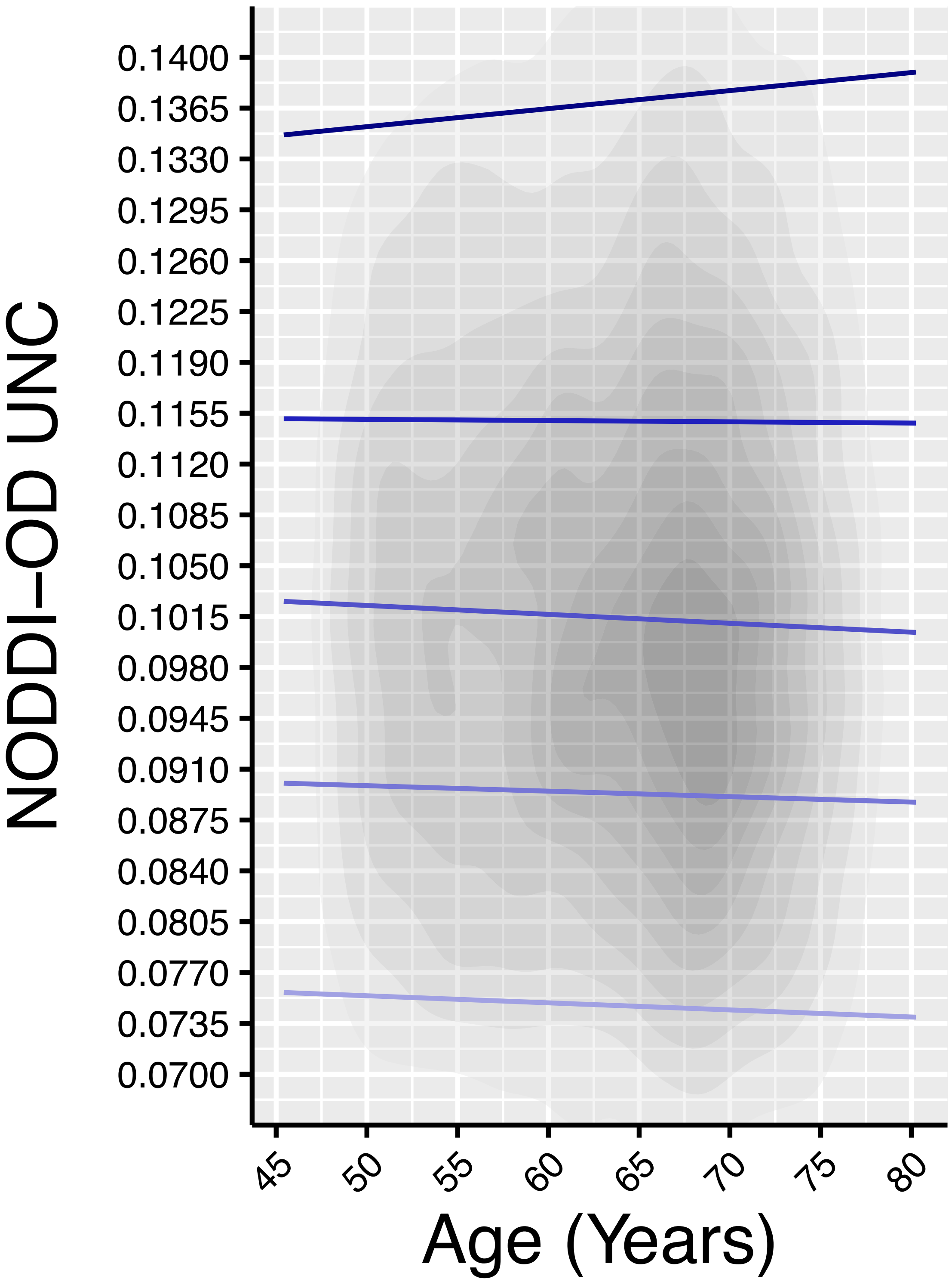

Figure S364. Full size normative centile reference curves calculated for the uncinate fasciculus tract for NODDI-OD in males. Solid colored lines, ordered from lightest to darkest, indicate the following centiles: 5th, 25th, 50th, 75th, 95th. Gray overlay reflects kernel density (darker=greater degree of data point overlap). UNC = uncinate fasciculus.

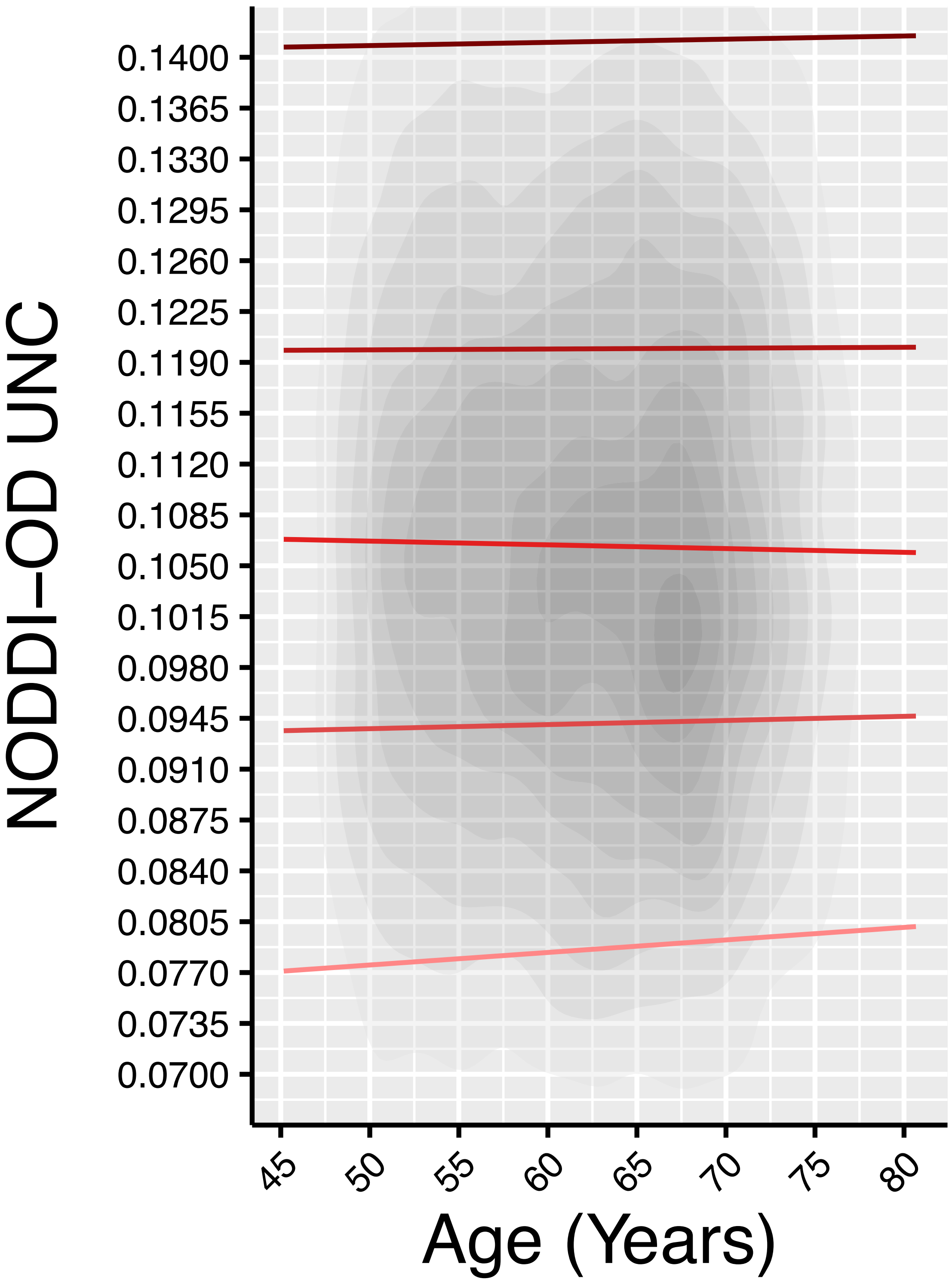

Figure S365. Full size normative centile reference curves calculated for the uncinate fasciculus tract for NODDI-OD in females. Solid colored lines, ordered from lightest to darkest, indicate the following centiles: 5th, 25th, 50th, 75th, 95th. Gray overlay reflects kernel density (darker=greater degree of data point overlap). UNC = uncinate fasciculus.

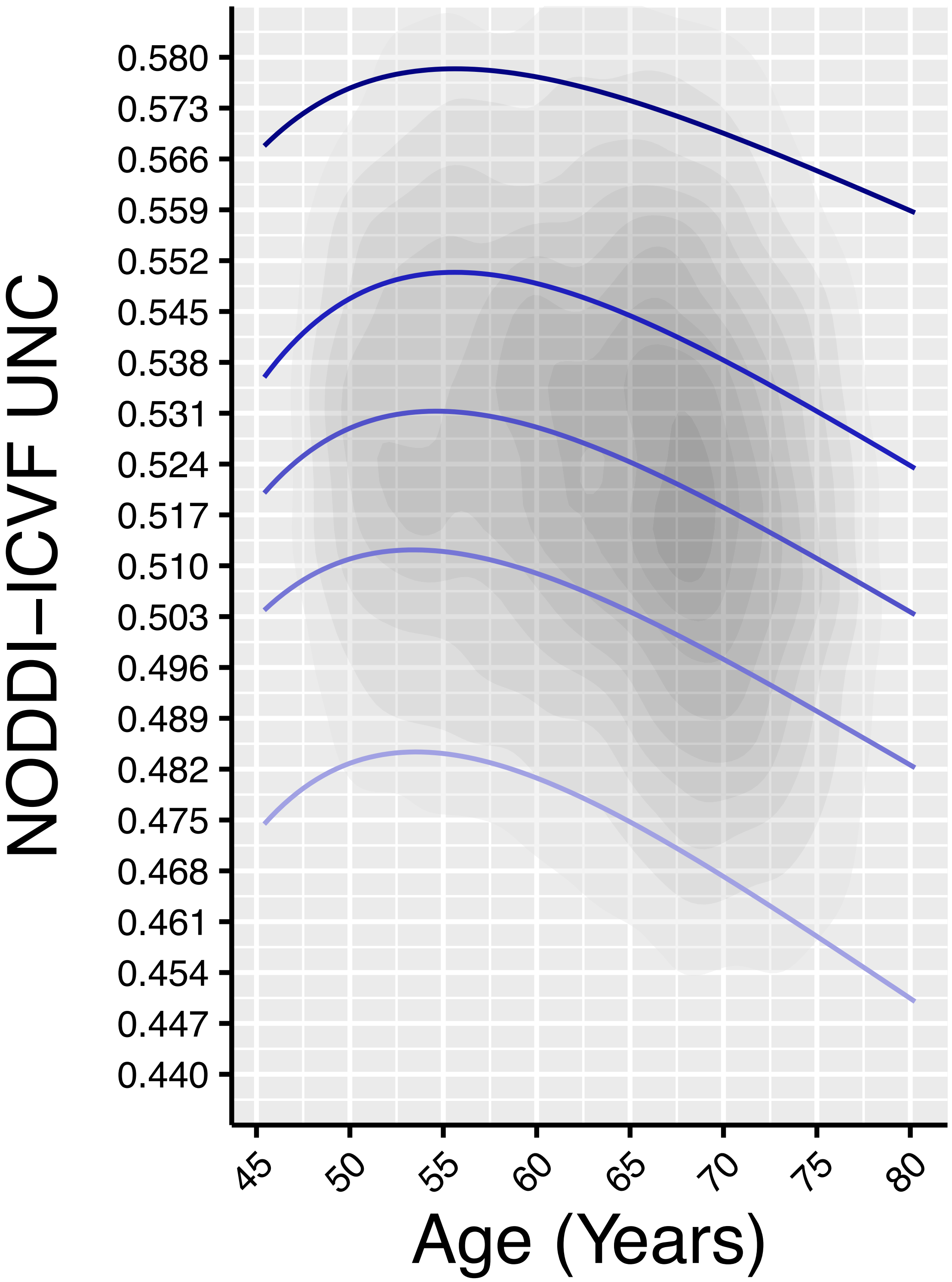

Figure S366. Full size normative centile reference curves calculated for the uncinate fasciculus tract for NODDI-ICVF in males. Solid colored lines, ordered from lightest to darkest, indicate the following centiles: 5th, 25th, 50th, 75th, 95th. Gray overlay reflects kernel density (darker=greater degree of data point overlap). UNC = uncinate fasciculus.

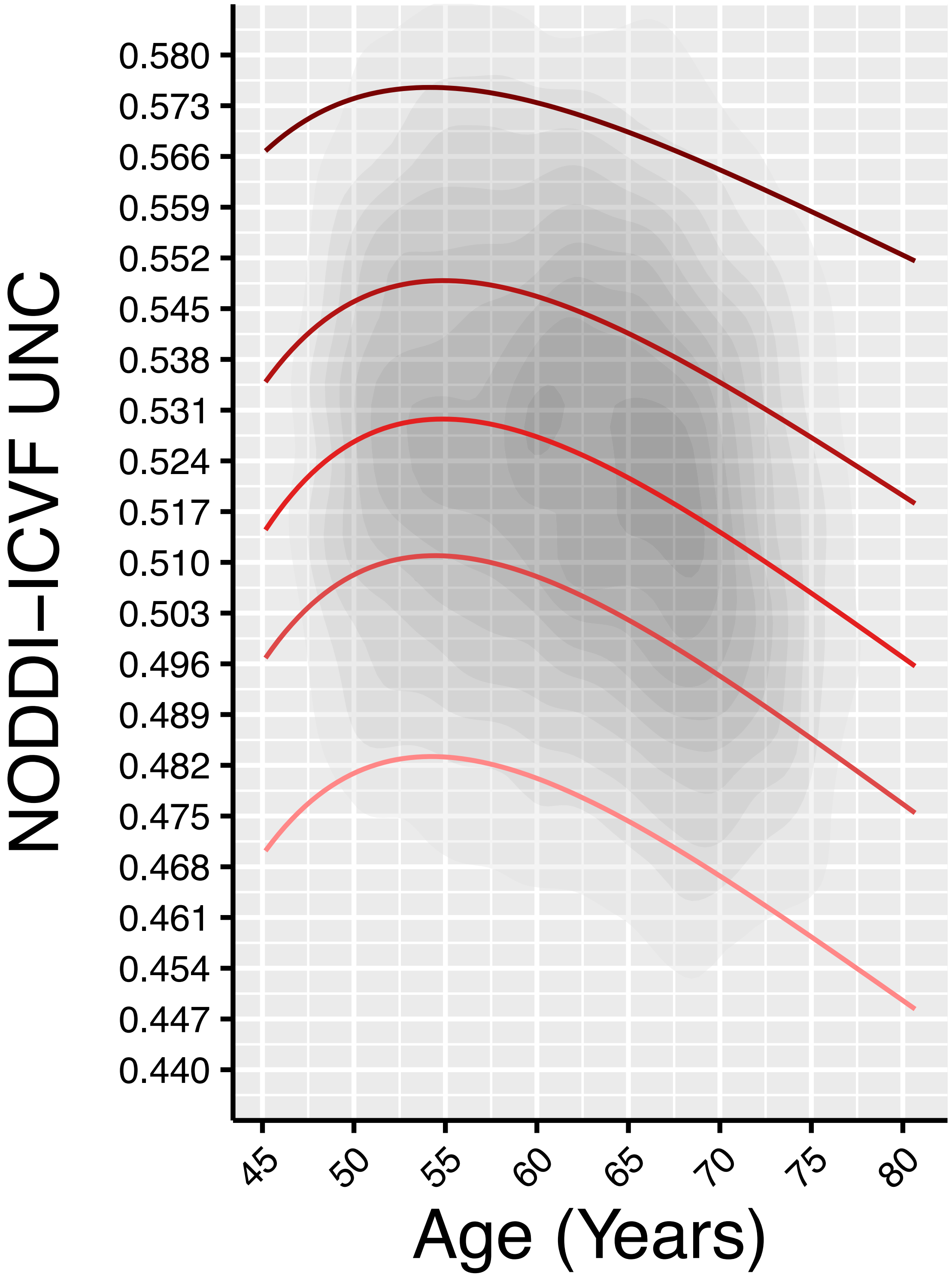

Figure S367. Full size normative centile reference curves calculated for the uncinate fasciculus tract for NODDI-ICVF in females. Solid colored lines, ordered from lightest to darkest, indicate the following centiles: 5th, 25th, 50th, 75th, 95th. Gray overlay reflects kernel density (darker=greater degree of data point overlap). UNC = uncinate fasciculus.

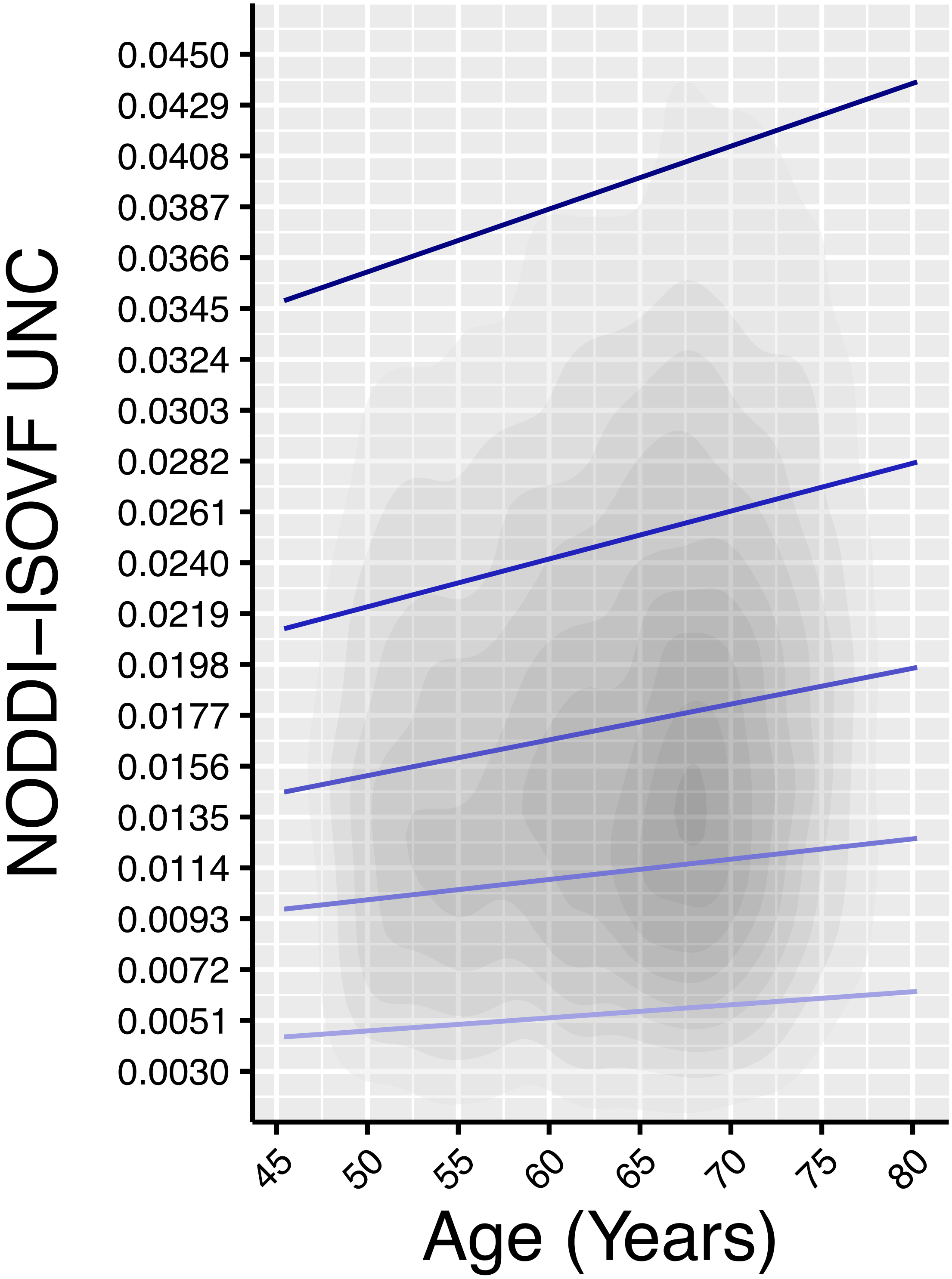

Figure S368. Full size normative centile reference curves calculated for the uncinate fasciculus tract for NODDI-ISOVF in males. Solid colored lines, ordered from lightest to darkest, indicate the following centiles: 5th, 25th, 50th, 75th, 95th. Gray overlay reflects kernel density (darker=greater degree of data point overlap). UNC = uncinate fasciculus.

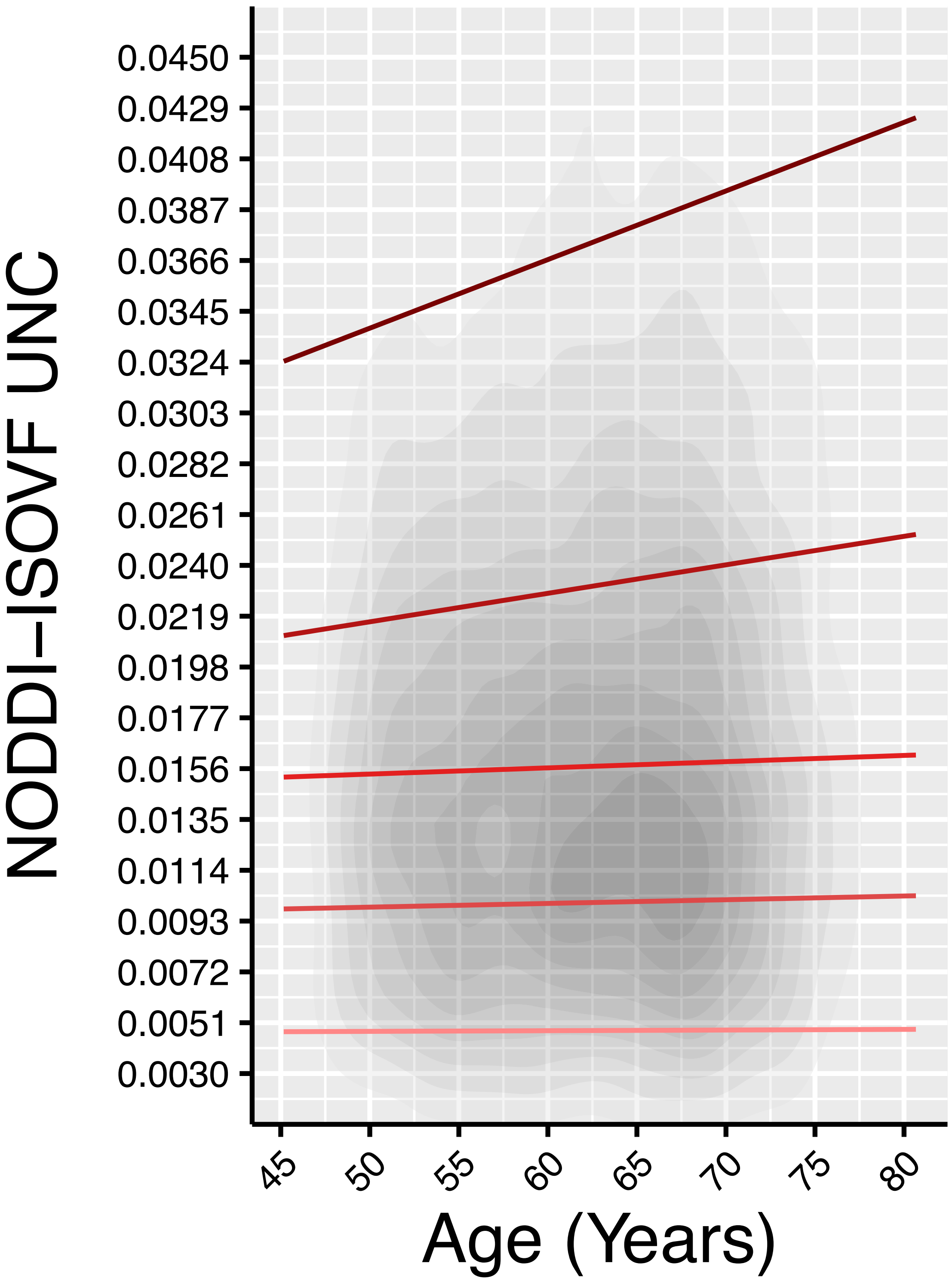

Figure S369. Full size normative centile reference curves calculated for the uncinate fasciculus tract for NODDI-ISOVF in females. Solid colored lines, ordered from lightest to darkest, indicate the following centiles: 5th, 25th, 50th, 75th, 95th. Gray overlay reflects kernel density (darker=greater degree of data point overlap). UNC = uncinate fasciculus.

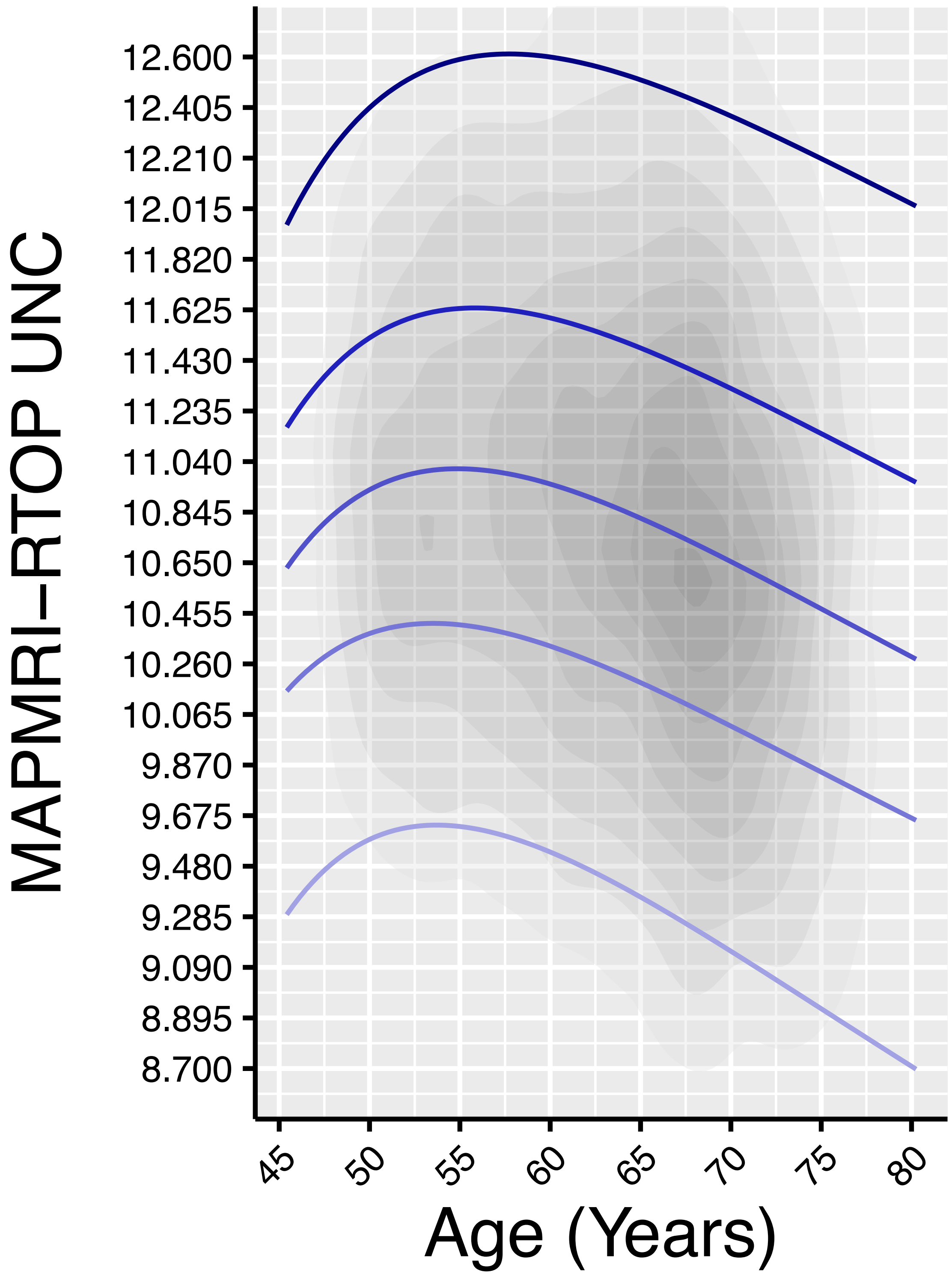

Figure S370. Full size normative centile reference curves calculated for the uncinate fasciculus tract for MAPMRI-RTOP in males. Solid colored lines, ordered from lightest to darkest, indicate the following centiles: 5th, 25th, 50th, 75th, 95th. Gray overlay reflects kernel density (darker=greater degree of data point overlap). UNC = uncinate fasciculus.

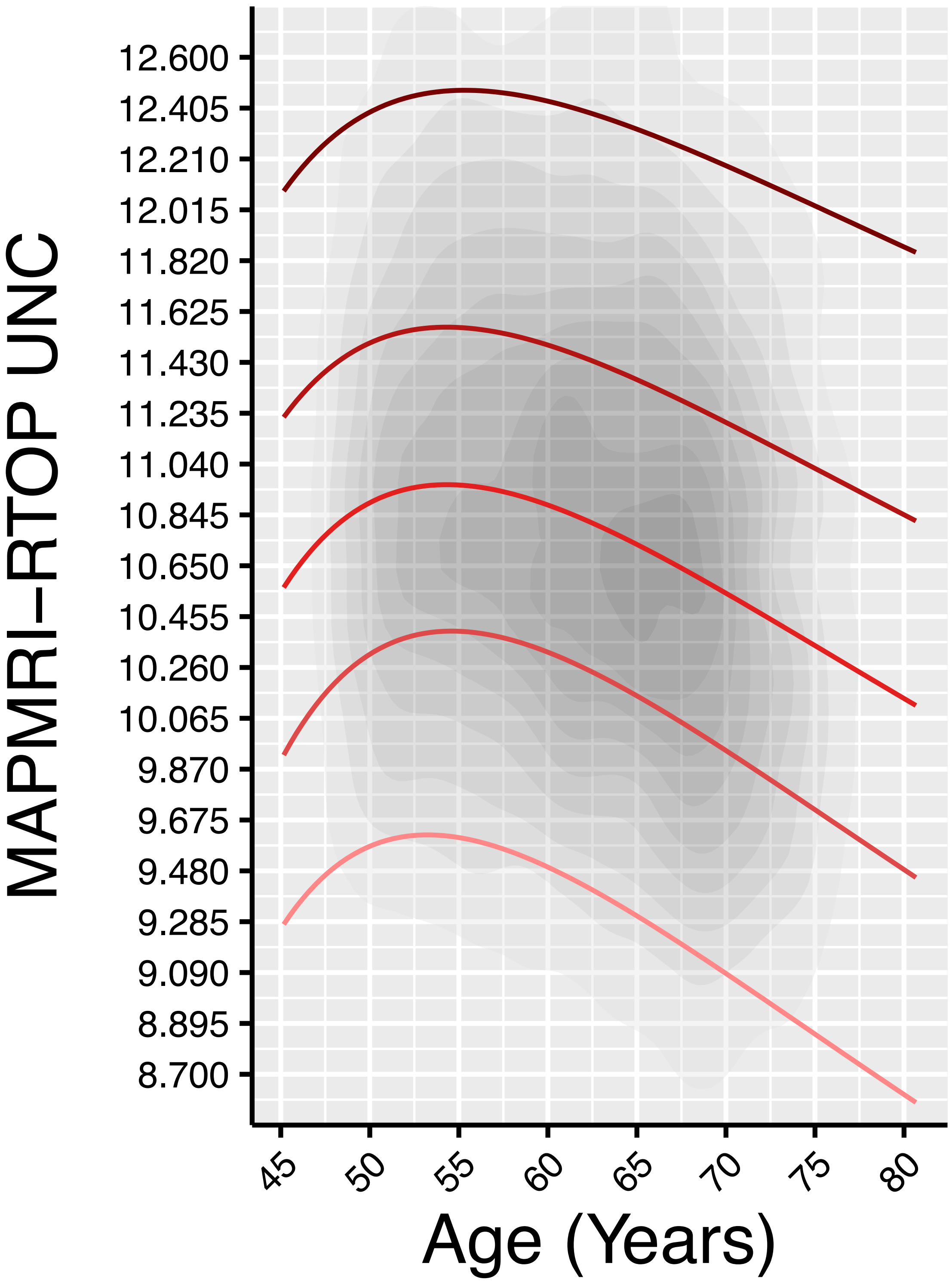

Figure S371. Full size normative centile reference curves calculated for the uncinate fasciculus tract for MAPMRI-RTOP in females. Solid colored lines, ordered from lightest to darkest, indicate the following centiles: 5th, 25th, 50th, 75th, 95th. Gray overlay reflects kernel density (darker=greater degree of data point overlap). UNC = uncinate fasciculus.

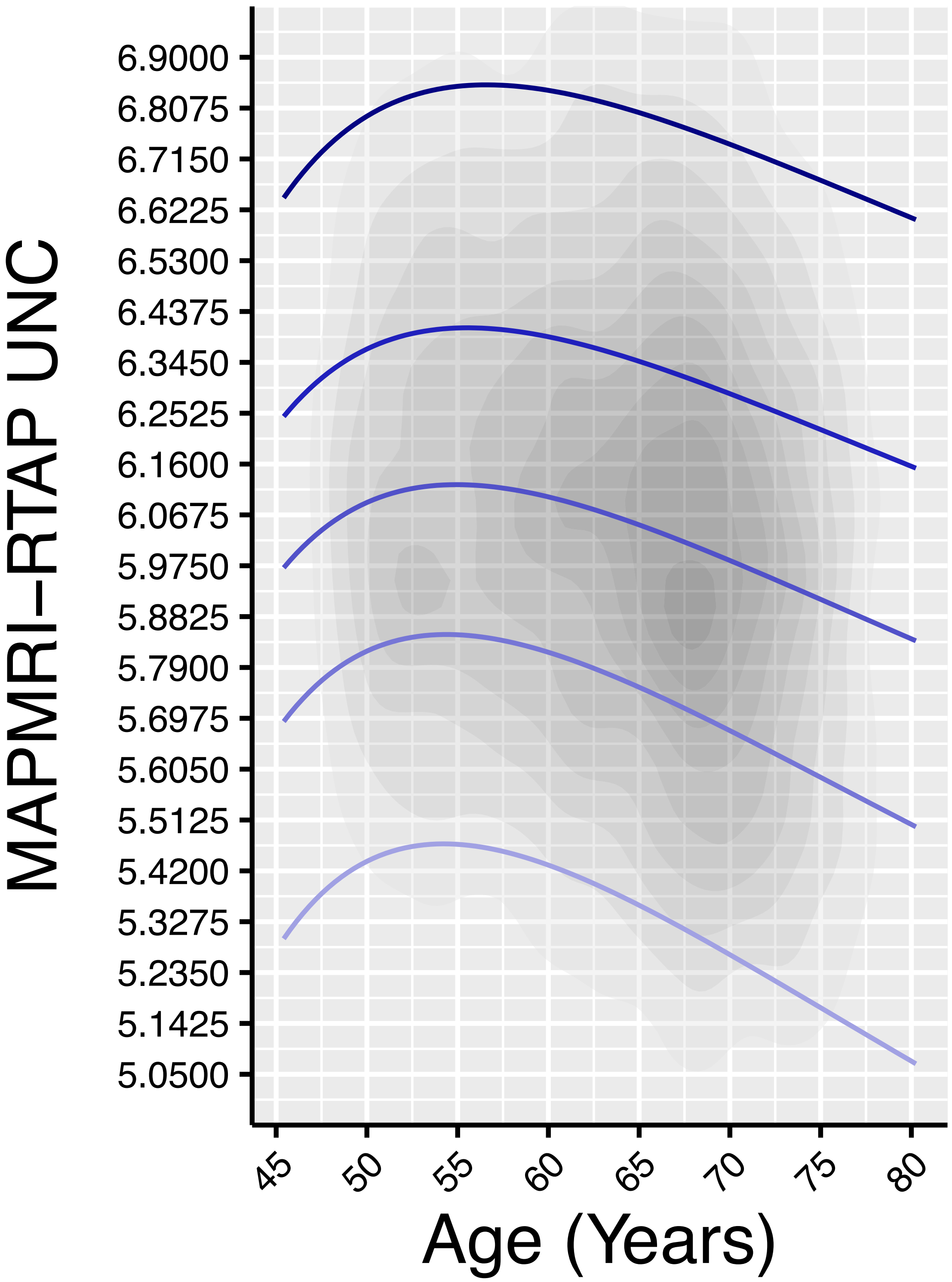

Figure S372. Full size normative centile reference curves calculated for the uncinate fasciculus tract for MAPMRI-RTAP in males. Solid colored lines, ordered from lightest to darkest, indicate the following centiles: 5th, 25th, 50th, 75th, 95th. Gray overlay reflects kernel density (darker=greater degree of data point overlap). UNC = uncinate fasciculus.

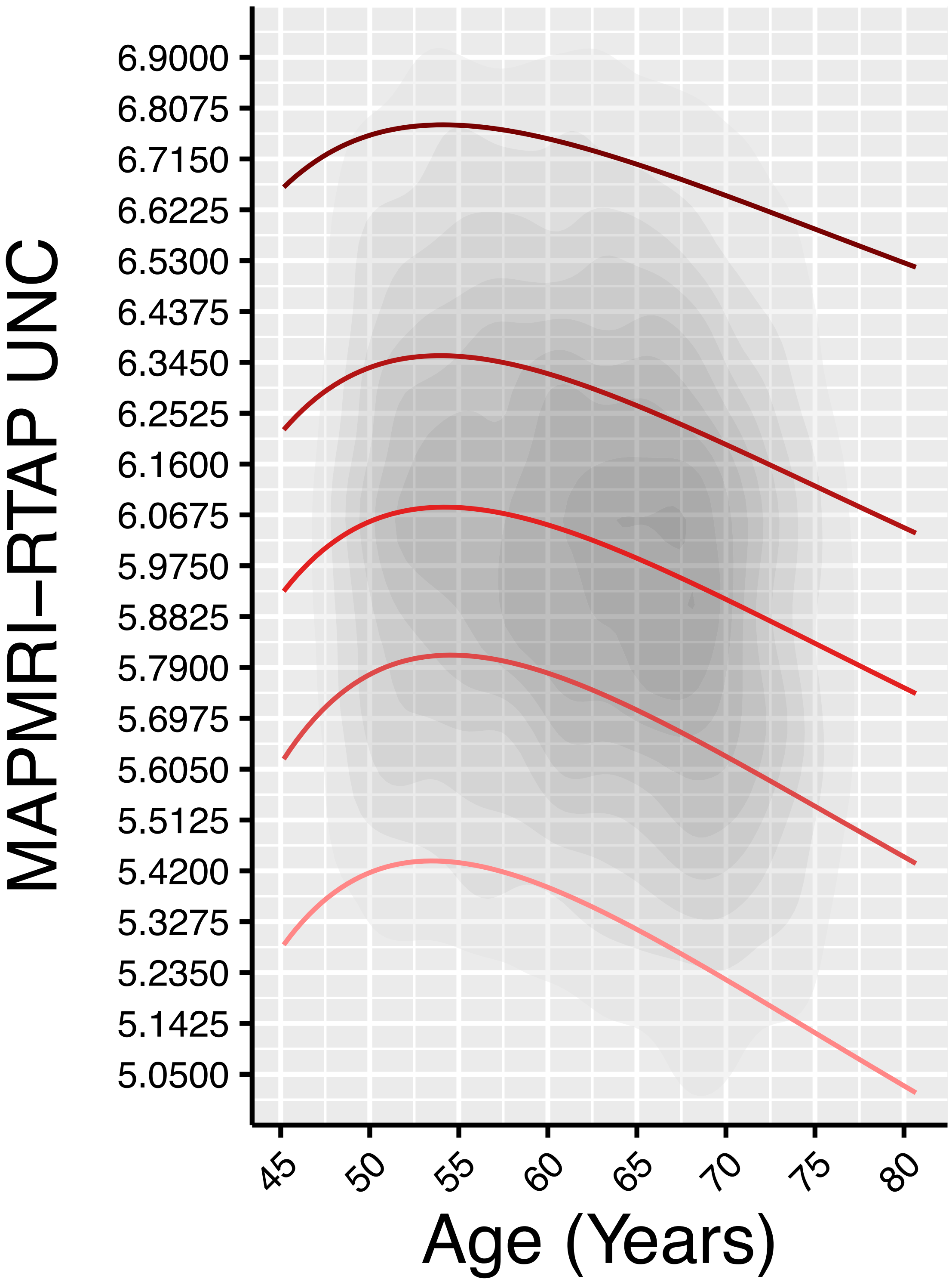

Figure S373. Full size normative centile reference curves calculated for the uncinate fasciculus tract for MAPMRI-RTAP in females. Solid colored lines, ordered from lightest to darkest, indicate the following centiles: 5th, 25th, 50th, 75th, 95th. Gray overlay reflects kernel density (darker=greater degree of data point overlap). UNC = uncinate fasciculus.

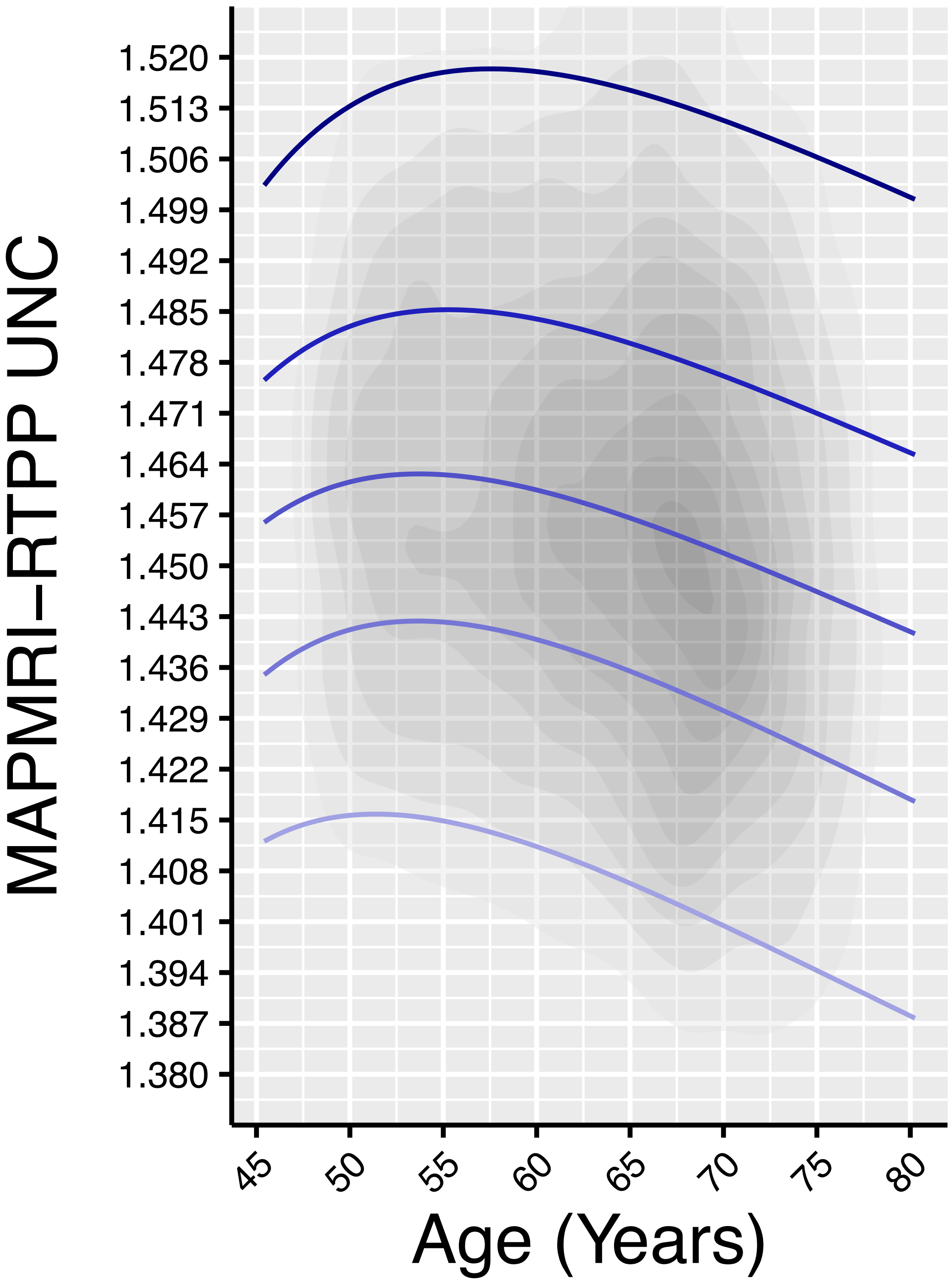

Figure S374. Full size normative centile reference curves calculated for the uncinate fasciculus tract for MAPMRI-RTPP in males. Solid colored lines, ordered from lightest to darkest, indicate the following centiles: 5th, 25th, 50th, 75th, 95th. Gray overlay reflects kernel density (darker=greater degree of data point overlap). UNC = uncinate fasciculus.

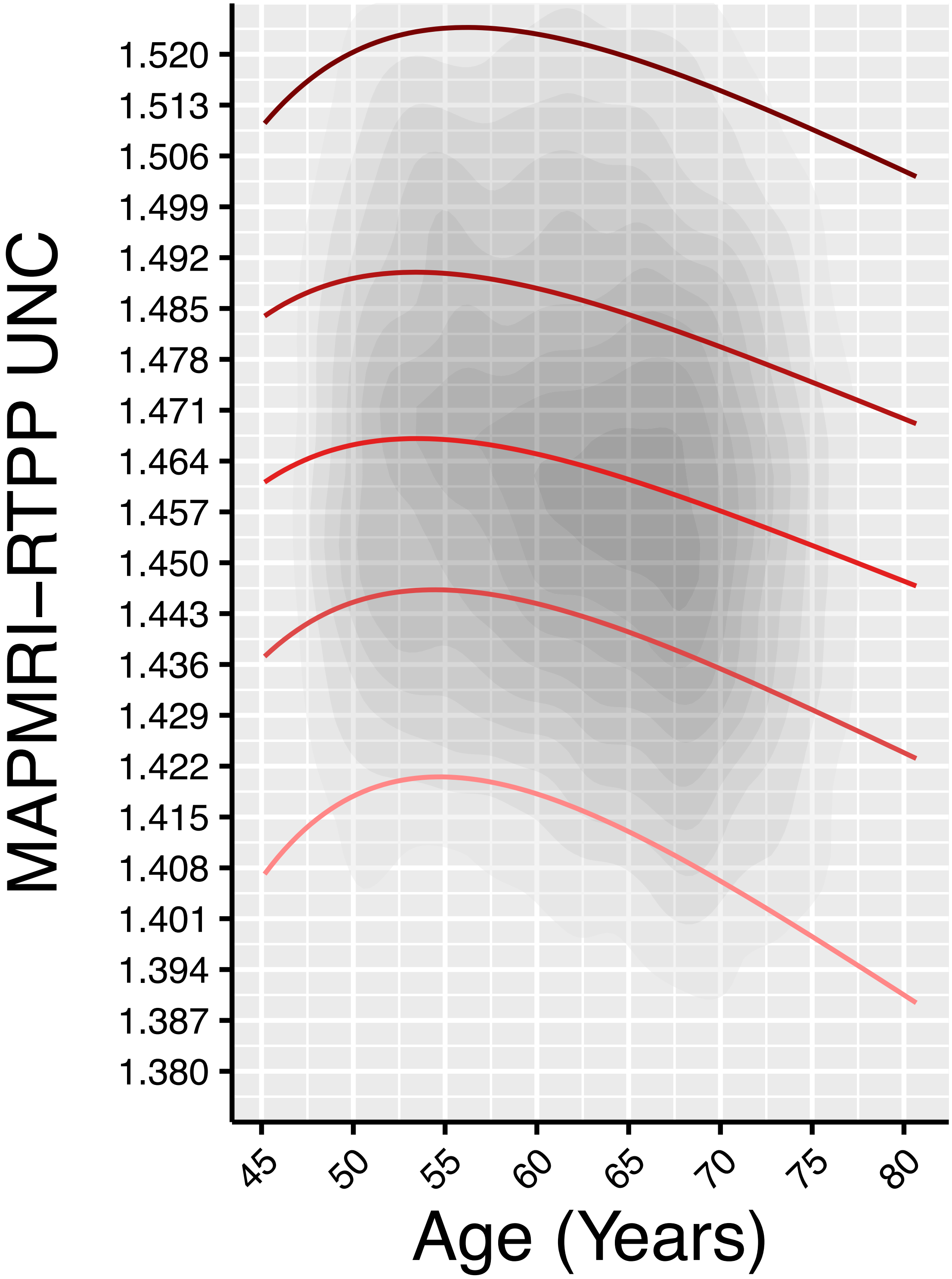

Figure S375. Full size normative centile reference curves calculated for the uncinate fasciculus tract for MAPMRI-RTPP in females. Solid colored lines, ordered from lightest to darkest, indicate the following centiles: 5th, 25th, 50th, 75th, 95th. Gray overlay reflects kernel density (darker=greater degree of data point overlap). UNC = uncinate fasciculus.

Supplementary References

Alfaro-Almagro, F., Jenkinson, M., Bangerter, N. K., Andersson, J. L. R., Griffanti, L., Douaud, G., . . . Smith, S. M. (2018). Image processing and Quality Control for the first 10,000 brain imaging datasets from UK Biobank. *Neuroimage, 166*, 400-424. doi:10.1016/j.neuroimage.2017.10.034

Andersson, J. L., & Sotiropoulos, S. N. (2015). Non-parametric representation and prediction of single- and multi-shell diffusion-weighted MRI data using Gaussian processes. *Neuroimage, 122*, 166-176. doi:10.1016/j.neuroimage.2015.07.067

Andersson, J. L. R., & Sotiropoulos, S. N. (2016). An integrated approach to correction for off-resonance effects and subject movement in diffusion MR imaging. *Neuroimage, 125*, 1063-1078. doi:10.1016/j.neuroimage.2015.10.019

Avants, B. B., Epstein, C. L., Grossman, M., & Gee, J. C. (2008). Symmetric diffeomorphic image registration with cross-correlation: evaluating automated labeling of elderly and neurodegenerative brain. *Med Image Anal, 12*(1), 26-41. doi:10.1016/j.media.2007.06.004

Basser, P. J., Mattiello, J., & Lebihan, D. (1994). Mr Diffusion Tensor Spectroscopy and Imaging. *Biophysical Journal, 66*(1), 259-267. doi:Doi 10.1016/S0006-3495(94)80775-1

Beck, D., de Lange, A. G., Maximov, II, Richard, G., Andreassen, O. A., Nordvik, J. E., & Westlye, L. T. (2020). White matter microstructure across the adult lifespan: A mixed longitudinal and cross-sectional study using advanced diffusion models and brain-age prediction. *Neuroimage, 224*, 117441. doi:10.1016/j.neuroimage.2020.117441

Benjamini, Y., & Hochberg, Y. (1995). Controlling the False Discovery Rate - a Practical and Powerful Approach to Multiple Testing. *Journal of the Royal Statistical Society Series B-Statistical Methodology, 57*(1), 289-300. Retrieved from <Go to ISI>://WOS:A1995QE45300017

Bycroft, C., Freeman, C., Petkova, D., Band, G., Elliott, L. T., Sharp, K., . . . Marchini, J. (2017). Genome-wide genetic data on ~500,000 UK Biobank participants. *bioRxiv*, 166298. doi:10.1101/166298

Daducci, A., Canales-Rodriguez, E. J., Zhang, H., Dyrby, T. B., Alexander, D. C., & Thiran, J. P. (2015). Accelerated Microstructure Imaging via Convex Optimization (AMICO) from diffusion MRI data. *Neuroimage, 105*, 32-44. doi:10.1016/j.neuroimage.2014.10.026

Dima, D., Papachristou, E., Modabbernia, A., Doucet, G. E., Agartz, I., Aghajani, M., . . . Frangou, S. (2020). Subcortical Volume Trajectories across the Lifespan: Data from 18,605 healthy individuals aged 3-90 years. *bioRxiv*, 2020.2005.2005.079475. doi:10.1101/2020.05.05.079475

Fick, R. H. J., Wassermann, D., Caruyer, E., & Deriche, R. (2016). MAPL: Tissue microstructure estimation using Laplacian-regularized MAP-MRI and its application to HCP data. *Neuroimage, 134*, 365-385. doi:10.1016/j.neuroimage.2016.03.046

Frangou, S., Modabbernia, A., Doucet, G. E., Papachristou, E., Williams, S. C., Agartz, I., . . . Dima, D. (2020). Cortical Thickness Trajectories across the Lifespan: Data from 17,075 healthy individuals aged 3-90 years. *bioRxiv*, 2020.2005.2005.077834. doi:10.1101/2020.05.05.077834

Garyfallidis, E., Brett, M., Amirbekian, B., Rokem, A., van der Walt, S., Descoteaux, M., . . . Dipy, C. (2014). Dipy, a library for the analysis of diffusion MRI data. *Front Neuroinform, 8*, 8. doi:10.3389/fninf.2014.00008

Jahanshad, N., Kochunov, P. V., Sprooten, E., Mandl, R. C., Nichols, T. E., Almasy, L., . . . Glahn, D. C. (2013). Multi-site genetic analysis of diffusion images and voxelwise heritability analysis: a pilot project of the ENIGMA-DTI working group. *Neuroimage, 81*, 455-469. doi:10.1016/j.neuroimage.2013.04.061

Jahanshad, N., & Thompson, P. M. (2017). Multimodal neuroimaging of male and female brain structure in health and disease across the life span. *J Neurosci Res, 95*(1-2), 371-379. doi:10.1002/jnr.23919

Jones, D. K. (2008). Studying connections in the living human brain with diffusion MRI. *Cortex, 44*(8), 936-952. doi:10.1016/j.cortex.2008.05.002

Leow, A. D., Zhu, S., Zhan, L., McMahon, K., de Zubicaray, G. I., Meredith, M., . . . Thompson, P. M. (2009). The tensor distribution function. *Magn Reson Med, 61*(1), 205-214. doi:10.1002/mrm.21852

Miller, K. L., Alfaro-Almagro, F., Bangerter, N. K., Thomas, D. L., Yacoub, E., Xu, J., . . . Smith, S. M. (2016). Multimodal population brain imaging in the UK Biobank prospective epidemiological study. *Nat Neurosci, 19*(11), 1523-1536. doi:10.1038/nn.4393

Mori, S., Oishi, K., Jiang, H., Jiang, L., Li, X., Akhter, K., . . . Mazziotta, J. (2008). Stereotaxic white matter atlas based on diffusion tensor imaging in an ICBM template. *Neuroimage, 40*(2), 570-582. doi:10.1016/j.neuroimage.2007.12.035

Nir, T. M., Jahanshad, N., Villalon-Reina, J. E., Isaev, D., Zavaliangos-Petropulu, A., Zhan, L., . . . Alzheimer's Diseaase Neuroimaginng, I. (2017). Fractional anisotropy derived from the diffusion tensor distribution function boosts power to detect Alzheimer's disease deficits. *Magn Reson Med, 78*(6), 2322-2333. doi:10.1002/mrm.26623

Ozarslan, E., Koay, C. G., Shepherd, T. M., Komlosh, M. E., Irfanoglu, M. O., Pierpaoli, C., & Basser, P. J. (2013). Mean apparent propagator (MAP) MRI: a novel diffusion imaging method for mapping tissue microstructure. *Neuroimage, 78*, 16-32. doi:10.1016/j.neuroimage.2013.04.016

Pines, A. R., Cieslak, M., Larsen, B., Baum, G. L., Cook, P. A., Adebimpe, A., . . . Satterthwaite, T. D. (2020). Leveraging multi-shell diffusion for studies of brain development in youth and young adulthood. *Dev Cogn Neurosci, 43*, 100788. doi:10.1016/j.dcn.2020.100788

R Core Team (2016). *R: A language and environment for statistical computing*. Vienna, Austria: R Foundation for Statistical Computing.

Royston, P., & Altman, D. (1994). Regression using fractional polynomials of continuous covariates: parsimonious parametric modelling. *Applied Statistics, 43*(3), 429-467.

Royston, P., & Sauerbrei, W. (2004). A new approach to modelling interactions between treatment and continuous covariates in clinical trials by using fractional polynomials. *Stat Med, 23*(16), 2509-2525. doi:10.1002/sim.1815

Salminen, L. E., Wilcox, R. R., Zhu, A. H., Riedel, B. C., Ching, C. R. K., Rashid, F., . . . Jahanshad, N. (2019). Altered Cortical Brain Structure and Increased Risk for Disease Seen Decades After Perinatal Exposure to Maternal Smoking: A Study of 9000 Adults in the UK Biobank. *Cereb Cortex, 29*(12), 5217-5233. doi:10.1093/cercor/bhz060

Smith, S. M., Jenkinson, M., Johansen-Berg, H., Rueckert, D., Nichols, T. E., Mackay, C. E., . . . Behrens, T. E. (2006). Tract-based spatial statistics: voxelwise analysis of multi-subject diffusion data. *Neuroimage, 31*(4), 1487-1505. doi:10.1016/j.neuroimage.2006.02.024

Townsend, P., Phillimore, P., & Beattie, A. (1988). *Health and Deprivation: Inequality and the North*: Routledge.

Zhang, H., Schneider, T., Wheeler-Kingshott, C. A., & Alexander, D. C. (2012). NODDI: practical in vivo neurite orientation dispersion and density imaging of the human brain. *Neuroimage, 61*(4), 1000-1016. doi:10.1016/j.neuroimage.2012.03.072
